# Biomimetic Synthesis of Chejuenolides A–C by a Cryptic Lactone-Based Macrocyclization: Stereochemical Implications in Biosynthesis

**DOI:** 10.1021/acscentsci.2c01096

**Published:** 2023-01-04

**Authors:** Bingbing Zhang, Kuan Zheng, Ran Hong

**Affiliations:** † CAS Key Laboratory of Synthetic Chemistry of Natural Substances, Center for Excellence in Molecular Synthesis, Shanghai Institute of Organic Chemistry, Chinese Academy of Sciences, Shanghai 200032, PR China; ‡ University of Chinese Academy of Sciences, Beijing 100049, PR China

## Abstract

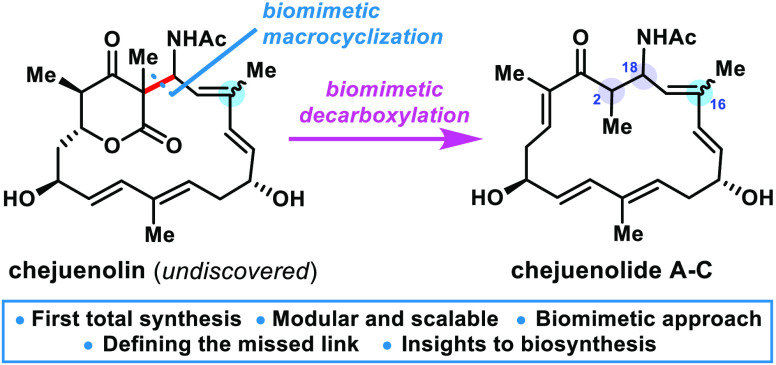

A hypothetical Mannich macrocyclization in the biosynthesis of chejuenolides A–C served as the basis for the synthetic design herein. Using a lactone-based linear precursor constructed via a tactic sequence of aldol–Julia–aldol reactions on a gram scale, the biomimetic total synthesis and structural validation of chejuenolides A–C were successfully achieved for the first time. The β-oxo-δ-lactone unit in the macrocyclized adducts was fragile and readily converted to a series of C2/C18-diastereoisomers via a decarboxylation and protonation pathway. Stereochemical identification of the biosynthetic precursor (O3P2) confirmed structural adherence to the given macrocycles and previously clarified lankacidins. Moreover, the stereovariants of the linear precursor designed for the macrocyclization event highlighted the unparalleled impact of using this biomimetic approach to determine the stereoselectivity in the proposed enzymatic reaction by reviving the lost or unstable intermediate.

## Abstract 2

Biomimetic synthesis emulates the (proposed) biosynthetic pathway and reveals the chemical logic of molecular construction, providing a mechanistic guidance for the stereochemical refinement and the discovery of latent natural products.

## Introduction

Macrocyclic natural products often exhibit remarkable biological activities, and many of them and their derivatives are used to treat various cancers and pathogens.^1,2^ The fascinating chemical architectures, such as macrolides, macrolactams, and polypeptides, inspire innovative drug design and inventive chemical probes to illustrate biological pathways.^3−5^ A growing interest in protein–protein interactions (PPIs), which are prevalent difficult-to-drug targets, necessitates the development of new sophisticated ligands with multiple noncovalent interactions.^6,7^ Macrocycles are rationally designed to rigidify a flexible linear fragment into a well-defined conformation and to comprise multiple interactions that alleviate entropic penalties during protein–ligand interaction.^8^ In the past decades, numerous impressive synthesis methods have been devoted to efficiently constructing macrocycles, including dehydration-reagent-based macrocyclization (macrolactonization^9^ and macrolactamization^10^) and transition-metal-catalyzed metathesis,^11,12^ among others.^13−15^

**Scheme 1:**
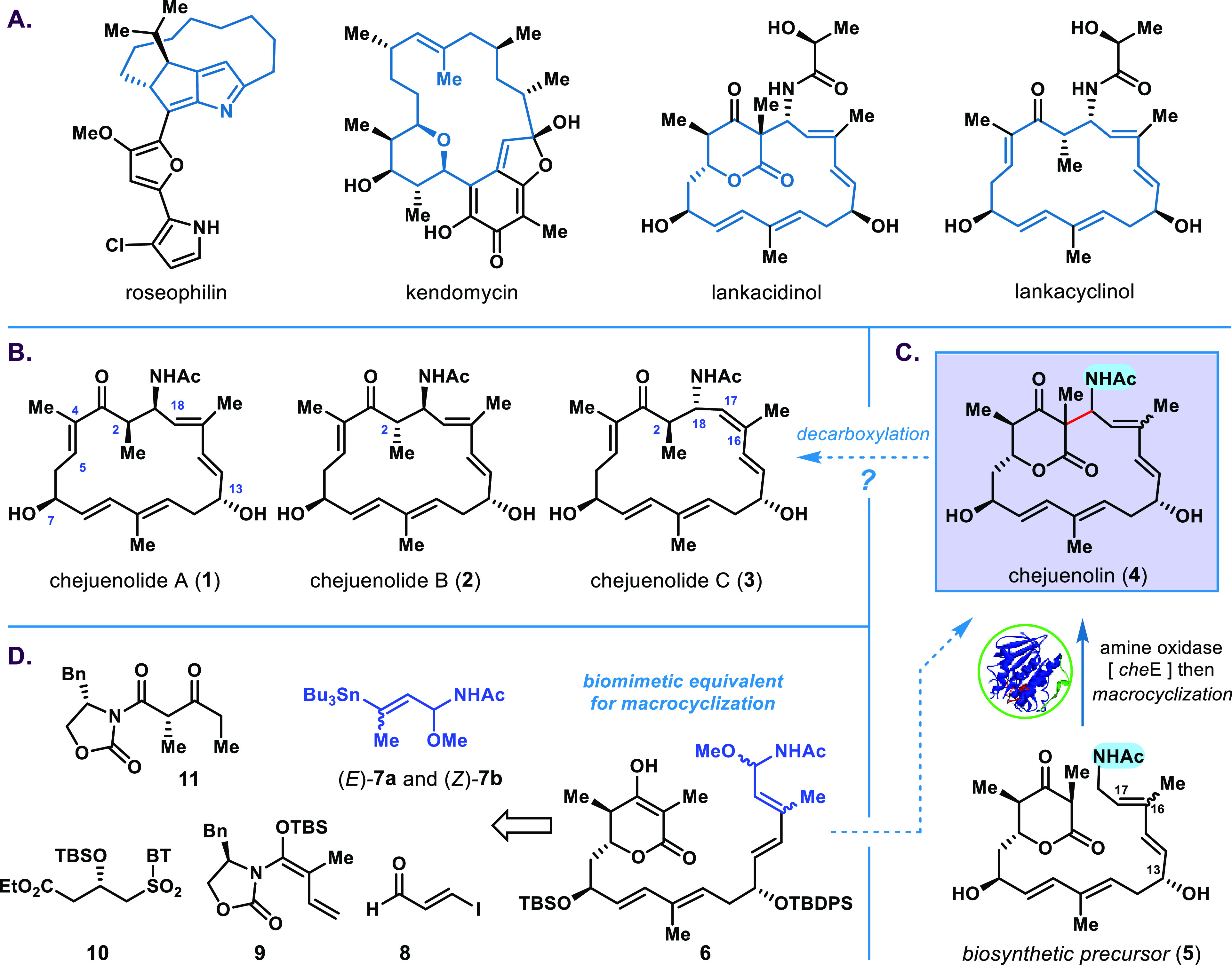
(A) Represented Macrocycles with the All-Carbon Framework; (B) Chemical Structures of Chejuenolides A–C; (C) A Possible Biosynthesis Involving a Cryptic Lactone; (D) Biomimetic Approach and Starting Fragments (**7**–**11**)

Nature has evolved intriguing pathways to produce macrocycles without an embedded ester or amide module, such as roseophilin, kendomycin, and lankacidins (Scheme 1A).^16−18^ The construction of all-carbon macrocycles is generally challenging, yet inspiring for the development of novel and effective strategies.^19−21^ Chejuenolides A and B were isolated from the Gram-negative marine bacterium *Hahella chejuensis* by Oh and co-workers (2008).^22^ Both secondary metabolites contain 17-membered carbocyclic tetraenes and exhibit weak inhibitory activity for protein tyrosine phosphatase 1B (PTP1B), a novel drug development target for the treatment of type-2 diabetes and related metabolic syndromes (Scheme 1B).^23,24^ Comparing the structures of chejuenolides and lankacidins^18^ revealed an identical carbon framework and geometric alkenes, indicating that the biosynthetic pathways are closely related each other.^25^ Recent interpretation of a biosynthetic gene cluster revealed that the *che* PKSs have a similar modular structure to the *lkc* PKSs that produce lankacidin C. Additionally, the gene inactivation experiment confirmed that *cheE* is an amine oxidase responsible for oxidative macrocyclization.^26^ However, an acetamide group instead of a 2-hydroxypropamide branch was observed at C18, along with the inversed stereogenic center at C13 and the missing β-oxo-δ-lactone (Scheme 1C). Moreover, no congeners of chejuenolides containing the β-oxo-δ-lactone subunit (denoted as chejuenolin (**4**)) have ever been characterized, and such a lactone form has yet to be chemically and biosynthetically validated. Interestingly, an unprecedented *cis*-alkene-embedded chejuenolide C (ΔC_16_–C_17_) was also isolated from the same marine bacteria, posing a challenge in the origin of the double bond geometry in the biosynthetic machinery.^27^ These structural characteristics have emerged as an appealing quest for chemical synthesis to identify the proposed lactone intermediate and clarify the stereochemical control derived from various modules to reveal the capacity of the key Mannich-type macrocyclization.

## Results

### Synthetic Plan

To coincide with the proposed biosynthesis of chejuenolides, a Mannich-based macrocyclization is designed as the key step to refine the stereochemistry at C2 and C18 (Scheme 1D). This strategy offers the following attractive and complementary advantages by (1) offering an insightful understanding of the reactivity of the acetylated imine, (2) elevating the hypothetical intermediates (i.e., **4**) to execute the feasibility of δ-lactone in the final product, and (3) providing mechanistic insights into the origin of stereochemical control in the two newly generated stereocenters (C2 and C18). Retro-synthetically, the linear precursor **6** is virtually assembled by two strategic aldol reactions to establish the chiral centers at C7 and C13. Following the established protocol^28^ for preparing fragments **7**–**11**, vinylogous Mukaiyama aldol, modified Julia olefination, and Evans keto imide *anti*-aldol reactions have been identified as the preferred methods for allying these motifs. Thus, the thermo-regulated macrocyclization of **6** is highlighted to provide a cyclophane-type structure, and the subsequent decarboxylation and protonation will yield chejuenolides A (**1**) and B (**2**), where the two stereoisomers differ in the configuration only at C2. Moreover, (*Z*)-*N*,*O*-acetal **7b** is projected for the implementation of the *cis*-trisubstituted double bond at C_16_–C_17_ in chejuenolide C (**3**).

### Total Synthesis

**Scheme 2:**
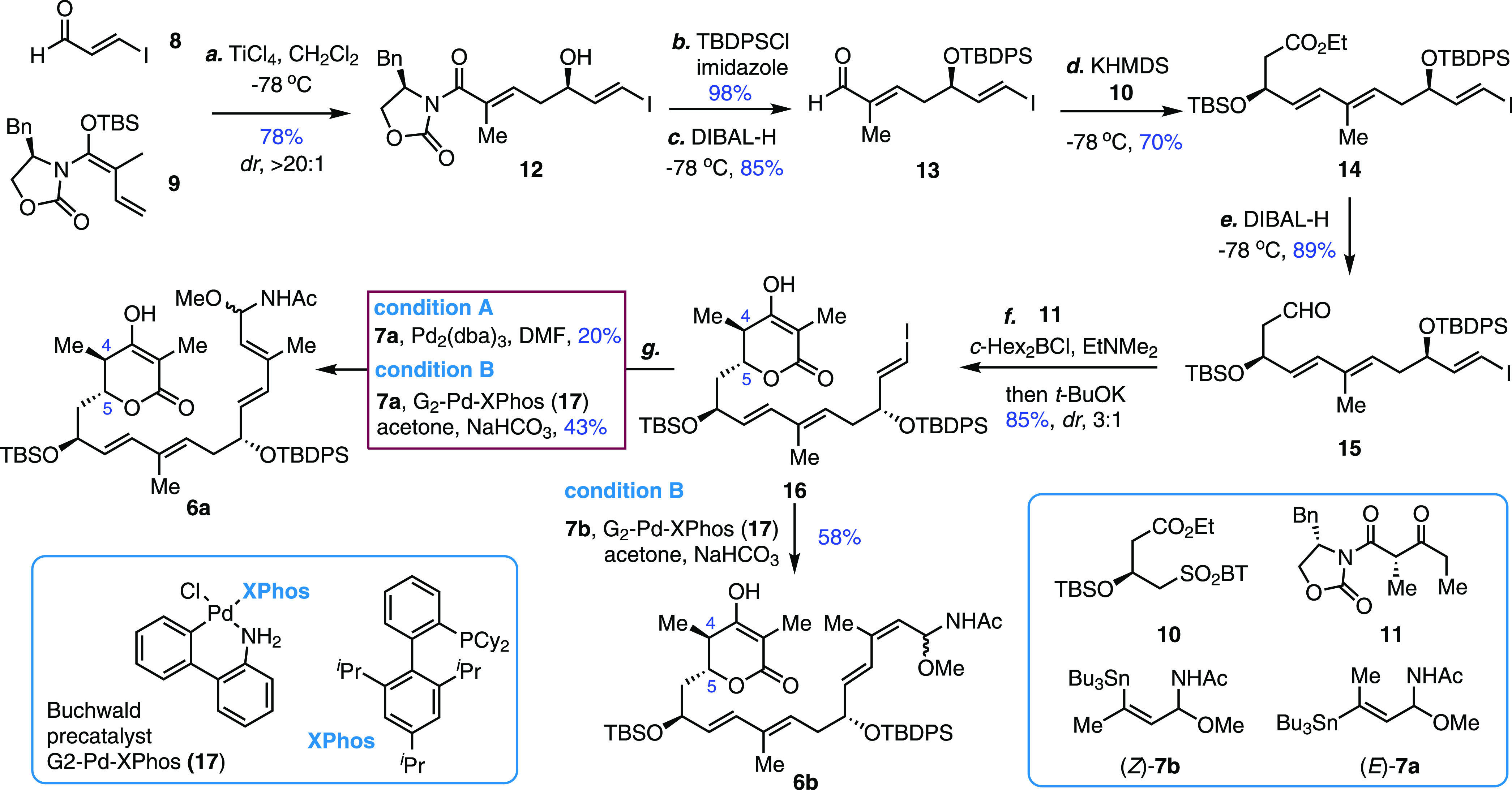
Modular Construction of the Linear Precursors **6a** and **6b** Bn, benzyl; TBS, *tert*-butyldimethylsilyl; TBDPS, *tert*-butyldiphenylsilyl; DIBAL-H, diisobutylalumium hydride; BT, benzothiazolyl; Pd_2_(dba)_3_, tris(dibentris(dibenzylideneacetone)dipalladium.

At the outset, the preparation of acetal **7a** containing an acetyl group presented an unanticipated challenge in terms of stability for the fragment synthesis. A judicious optimization of reaction parameters was essential to obtain the required stannylated *N*,*O*-acetals **7a** and **7b** in gram scale with good isolated yields (60% and 47%, respectively).^29^ Meanwhile, a stereodefined vinyl iodide **16** was constructed via a five-step sequence, including the Mukaiyama aldol reaction,^30^ modified Julia olefination,^31^ and Evans aldol reaction (Scheme 2).^32^ Subsequent application of palladium-catalyzed Stille coupling (condition A)^28^ of (*E*)-*N*,*O*-acetal **7a** with **16** delivered the linear polyene **6a** in 20% isolated yield. The yield of subsequent optimization was considerably increased (43%) when the Buchwald precatalyst G2–Pd–XPhos (**17**) (condition B)^33^ was used to inhibit the severe decomposition of *N*,*O*-acetal. Under optimal conditions, the coupling reaction of (*Z*)-*N*,*O*-acetal **7b** also proceeded smoothly to generate (*Z*)-trisubstituted alkene **6b** with a 58% yield without epimerization. The easily scalable protocol allowed us to accumulate sufficient material for crucial cyclization.

**Scheme 3:**
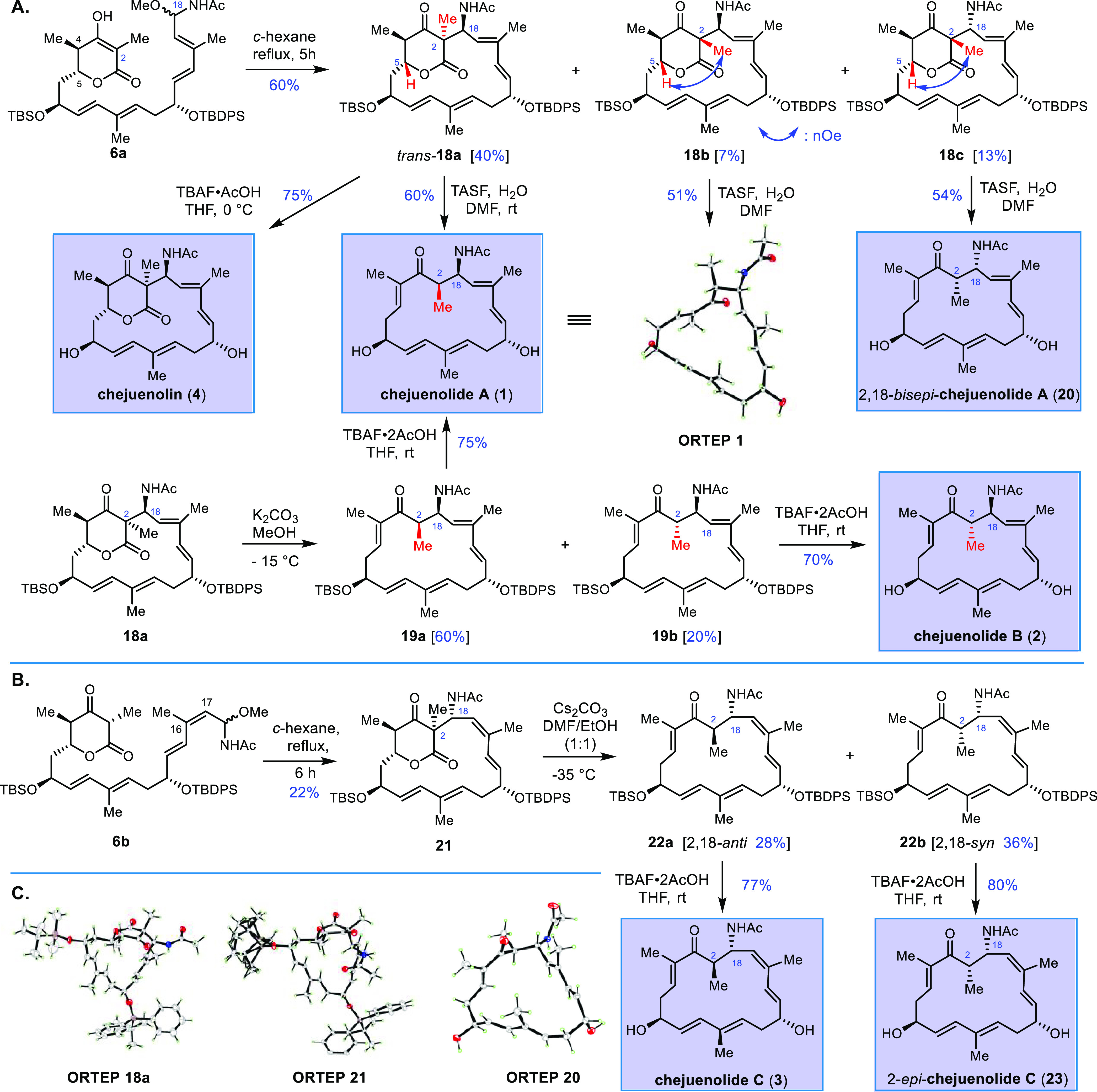
Macrocyclization, Structural Determination, and Synthesis of Chejuenolides A (**1**), B (**2**), and C (**3**), Chejuenolin (**4**), and Other Stereoisomers TASF, tris(dimethylamino)-sulfonium difluorotrimethylsilicate (IV); DMF, *N*,*N*-dimethylformamide; TBAF, tetrabutyl-ammonium fluoride.

The imine precursor **6a** was immersed in refluxing cyclohexane for 5 h, and three macrocycles were identified with a combined yield of 60% (Scheme 3A). The predominant stereoisomer **18a** (40% yield) was determined to be a 2,5-*trans*-δ-lactone owing to the absence of a characteristic NOE cross-peak between C5–H and C2–Me, and that was further unambiguously confirmed by X-ray analysis (Scheme 3C). Accordingly, the absolute configuration of the two newly formed stereocenters in **18a** was determined to be (2*R*,18*S*). The other two minor macrocycles **18b** and **18c** (ratio 1:2) had the NOE cross-peak between C5–H and C2–Me to confirm the 2,5-*cis*-δ-lactone unit; however, it was not possible to determine the stereochemistry at C18 at this stage. Regarding the diastereoselectivity of the Mannich reaction at C2, the ratio of 2:1 (*trans*-/*cis*-, **18a**/(**18b** + **18c**)) exhibited a reversed preference compared to the previous diastereomeric ratio (*trans*-/*cis*-, 1:3) in the lankacidin synthesis,^28^ indicating the profound effect on stereochemistry in the cyclization event by tailoring the tether, such as the acetyl group on N atom and the stereogenic center at C13 in **6a**.

To streamline the synthesis of chejuenolides A and B, lactone **18a** was first treated with TASF in DMF–H_2_O at ambient temperature. Decarboxylation and global deprotection proceeded smoothly, and chejuenolide A was isolated at a yield of 60% as the sole macrocyclic product. The NMR, optical rotation, and polarity of the synthesized sample match those in the literature.^22^ Moreover, a single crystal of chejuenolide A was unambiguously verified via X-ray analysis to assign the (2*R*,18*S*)-configuration in the Mannich adduct **18a**. We anticipated that, after decarboxylation, a diastereoselective protonation of the proposed enolate should be forged under milder conditions and that may lead to chejuenolide B. Accordingly, treatment of **18a** with K_2_CO_3_ in MeOH at −15 °C afforded decarboxylation and the isolation of two products **19a** and **19b** with a combined yield of 80%. The subsequent removal of the silyl groups proceeded stereospecifically to generate chejuenolides A (**1**) and B (**2**) with yields of 75% and 70%, respectively. The successful two-step protocol indicates that a conformational preference is essential for the kinetically controlled generation of chejuenolide B. To determine the propensity of lactone, after exposure of **18a** to anhydrous and neutral conditions, TBAF·AcOH in THF produced a global desilylated product **4** in which the δ-lactone ring remained intact, representing the first isolation of a hypothetical lactone-based chejuenolin (**4**). However, a slight increase in the reaction temperature (0 °C → 23 °C) or exposure to TBAF·2AcOH in THF afforded an extremely low yield (∼6%) of **4**. We also noticed the simple decomposition of **4** at room temperature in a pure form or solution (CDCl_3_). These scenarios demonstrate the fragility of δ-lactone in chejuenolide congeners under mild conditions, demanding a future careful reharvesting from natural resources.

With the aforementioned workflow, we turned to determine the stereochemistry of **18b** and **18c**. After treatment with TASF in DMF–H_2_O, **18b** and **18c** were converted to chejuenolide A (**1**) and 2,18-*epi*-chejuenolide A (**20**) with yields of 51% and 54%, respectively. A single crystal of 2,18-*epi*-chejuenolide A (**20**) verified the *syn*-configuration of C2 and C18 following the decarboxylation–protonation cascade. Therefore, the absolute configuration of C18 in **18b** was determined to be *S* and *R* in **18c**.

The successful application of macrocyclization of **6a** encouraged us to investigate the capacity of the lactone-based strategy with the *cis*-alkene-embedded **6b**. A major product **21** was isolated with a 22% yield and was the only cyclized product identified to contain a 2,5-*trans*-lactone unit. Several side products, including acyclic structures derived from [1,5]-H-shift and 6π-electrocyclization of imine, predominated,^29^ suggesting a conformational detraction derived from the twisted *E*,*Z*-diene geometry of the C14–C17 motif (Scheme 3B). The absolute configurations at C2 and C18 were unambiguously determined to be (2*R*,18*R*) by X-ray analysis.^29^ K_2_CO_3_ in MeOH slowed the corresponding macrocycle relative to the previous condition. Extensive examination revealed that Cs_2_CO_3_ in DMF–EtOH is an effective decarboxylation agent, and the corresponding *anti*-isomer **22a** was converted into chejuenolide C (**3**) in 77% yield by TBAF·2AcOH in THF. The sample was identical to the data reported in the literature.^27^ The slightly higher yield of *syn*-isomer **22b** from decarboxylation was also easily converted into 2-*epi*-chejuenolide C (**23**) in a yield of 80%. The generally favorable *syn*-isomers **23** and **20** (via **18c**) could represent undiscovered natural products as minor ingredients in the fermentation.^34^

### Stereoisomers in Macrocyclization

The fugitive nature of δ-lactone in macrocyclic chejuenolides makes it difficult to demonstrate the stereochemical refinement of such biosynthetic intermediates. To formulate a detailed scheme to clarify the path toward decarboxylated products, we decided to create a stereoisomer of the C4 and C5 positions for the macrocyclization and assess the viability of a conformational change to chejuenolide A or B.

**Scheme 4:**
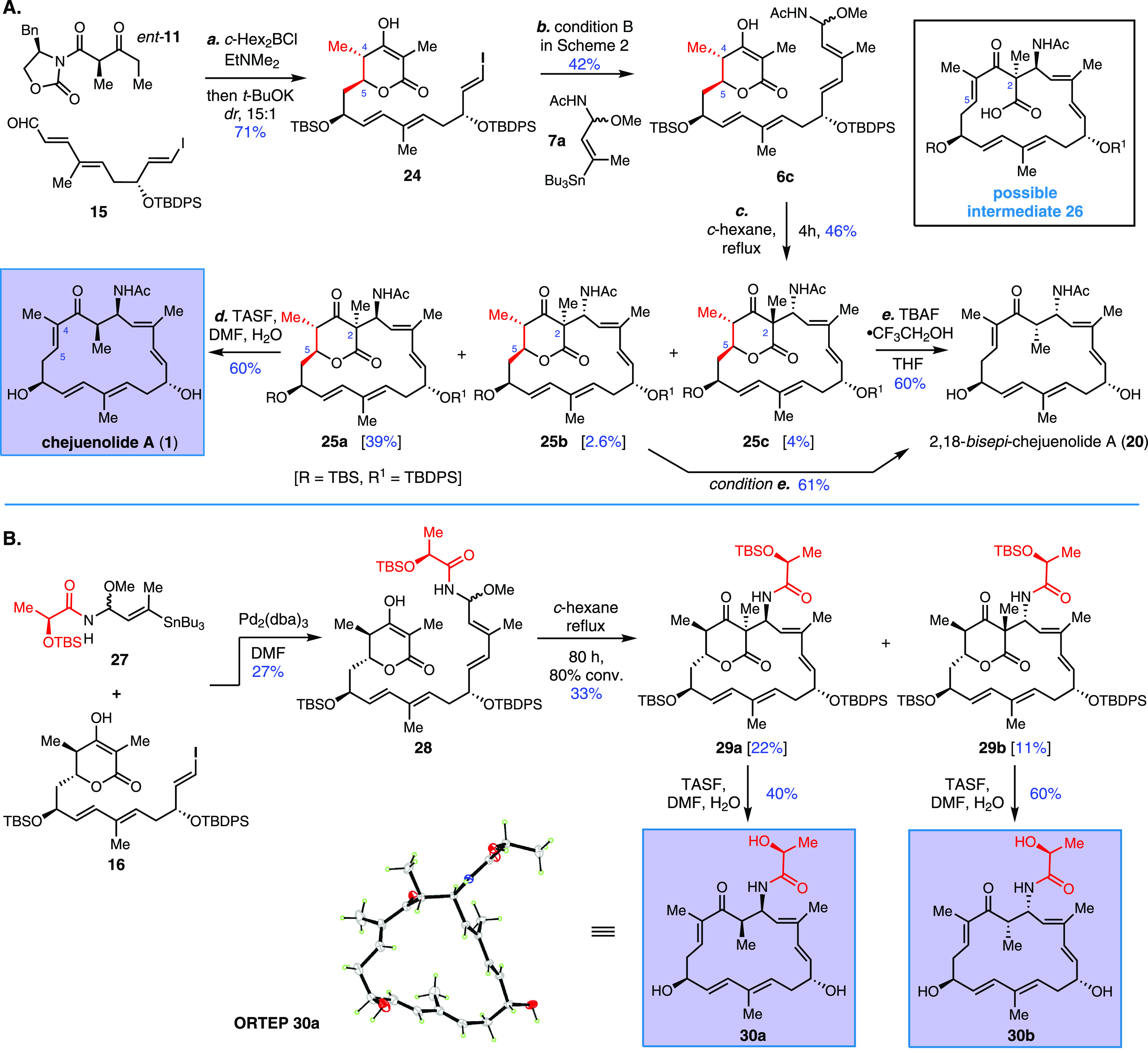
Syntheses of Chejuenolide A (**1**) and Its Stereoisomers and Analogues

The alternative Evans keto *anti*-aldol reaction of aldehyde **15** with *ent*-**11** generated (4*S*,5*S*)-aldol adduct **24** with excellent yield (71%) and diastereoselectivity (*dr* 15/1). Subsequent Stille coupling with **7a** by the modified protocol yielded the linear acetal **6c** (Scheme 4A). To our delight, the programmed macrocyclization remained effective for the hybrid precursor and successfully delivered three cyclized products with a total yield of 46%. With a characteristic NOE cross-peak of C2–Me and C5–H, the predominant macrocycle **25a** (39%) was assigned as the 2,5-*cis*-isomer, which is the opposite of the major stereoisomer derived from **6a**. Interestingly, the diastereomer **25a** was also viable to obtain chejuenolides A and B in high yield when a two-step protocol (i, K_2_CO_3_/MeOH; ii, TBAF·2AcOH) was applied. Moreover, chejuenolide A (**1**) could only be obtained using TASF. These results suggested the stereochemistry at C4 and C5 is not crucial for the formation of chejuenolides A and B; thus, the formation of the C4–C5 double bond via elimination occurs likely before the decarboxylation and protonation cascade, as shown in the inserted intermediate **26** in Scheme 4A. The vulnerability of ketoacid **26** at room temperature under mildly basic conditions (such as K_2_CO_3_ or TBAF) provides additional evidence that ketonic decarboxylation^35^ is energetically feasible under physiological conditions. Both minor macrocycles **25b** and **25c** were readily converted into the identical 2,18-*bisepi*-chejuenolide A (**20**), and the C18 position was thus assigned to be the *R*-configuration. Accordingly, the relative configuration of the corresponding macrocycles **25b/c** was determined based on the correlation of the NOE cross-peak between the C2–Me and C5–H. The current biomimetic synthesis of chejuenolides A–C highlights a sustainable approach to access the side chain modification for the discovery of novel antibacterial agents, which had previously been met with limited success through the preparation of ester derivatives that relied on biotransformation or tedious synthetic detours.^36,37^

To elucidate the functional group dependence in cyclization, we designed a hybrid substance **28**(29) with the lactamide group found in lankacidin derivatives (Scheme 4B). The stereochemistry of compound **28** is almost identical to that of the biosynthetic precursor toward lankacidins,^25^ except for the opposite configuration at C13. The macrocyclization of the linear hybrid **28** yielded two cyclized products, **29a** and **29b**, with a combined yield of 33% favoring the 2,5-*trans*-lactone isomer. Taken together with the cyclization of **6a**, the diastereoselectivity trend indicates that the remote stereogenic center at C13 plays a crucial role in the conformation preorganization of δ-lactone and in situ generated imine. Further X-ray analysis of the decarboxylated product **30a** (TASF, DMF–H_2_O, 40% yield) correlated with the absolute configuration of **29a**. The NOE correlation between C5–H and C2–Me confirmed **29b** and its decarboxylated derivative **30b** as (2*S*,18*R*) and (2*S*,18*R*), respectively.

### Determination of the Biosynthetic Precursor for Chejuenolides

An acyclic precursor O3P2 for biogenetic cyclization was identified from the disruption of an amine oxidase gene in a culture of the mutant strain.^38^ All four stereogenic centers lacked assigned stereochemistry, necessitating validation via total synthesis. Although the stereochemistry assignment can be confidently correlated with the detailed gene information on ketoreductase in polyketides,^39^ chemical synthesis remains a reliable approach to determine all possible stereoisomers of complex substances. Moreover, in our most recent study, a similar linear precursor LC-KA05-2 in the proposed biosynthesis of lankacidins raised doubts owing to the distant stereogenic centers of the linear structure.^40^ Therefore, it is crucial to define the stereochemistry of δ-lactone as an important unit at the early stage of chejuenolide biosynthesis.

**Scheme 5:**
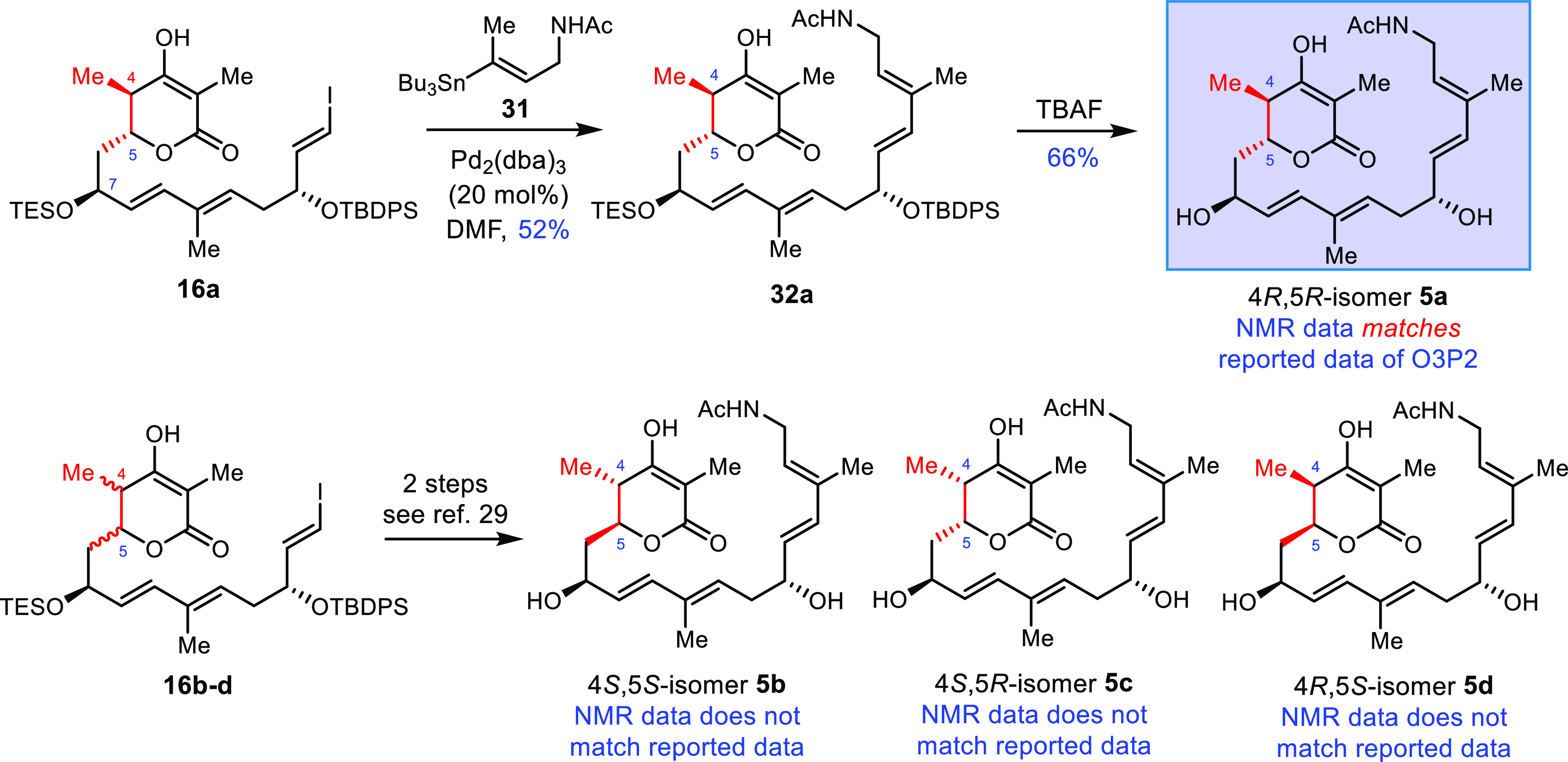
Stereochemical Determination of the Biosynthetic Precursor O3P2

The synthesis began with vinyl iodide **16a**, a surrogate for the TBS group on C7–OH in compound **16** (Scheme 5). To comprehensively verify the stereocenters at C4 and C5, all four diastereoisomers of C4 and C5 were synthesized using *anti*-aldol and *syn*-aldol protocols with moderate to excellent selectivity and good yields.^29^ Through palladium-catalyzed Stille coupling, four linear stereoisomers **32a**–**d** were isolated and the subsequent removal of two silyl groups released O3P2 and its congeners (**5a** and **5b**–**d**). When compared to the original data,^38^ compound **5a** was found to be identical, providing the elusive stereochemistry identification of C4 and C5 in the δ-lactone ring, which is consistent with the proposed structure of chejuenolin (**4**) and that of lanckacidins. The confirmation of the biosynthetic precursor further validates the stewardship of the gene cluster in the early stage of chejuenolide biosynthesis.

## Discussion

### Conformational Effect in Macrocyclization

**Scheme 6:**
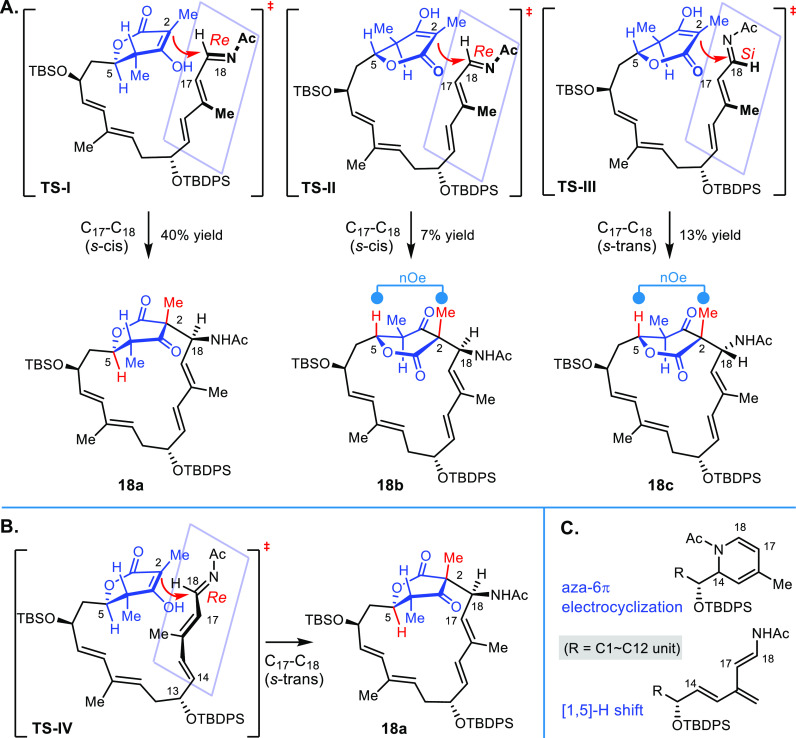
Stereochemical Rationale in the Macrocyclization of the Linear Precursor **6a**

Amino oxidase (*lkc*E)-enabled macrocyclization in lankacidin biosynthesis was a long-term goal and was recently revealed as a unique dual function of amide oxidase and macrocylase by Gruez, Weissman, and co-workers.^41^ However, the detailed mechanism for conformational preference-based stereochemical control remains elusive. In our previous biomimetic synthesis of lankacidins, the stereochemical diversification of *iso*lankacidinol and other congeners raised concerns regarding stereospecificity in enzymatic reactions.^42^ Together with various stereoisomers of lankacidins,^28^ the diastereoselective synthesis of the δ-lactone-embedded chejuenolides implicates that energetically comparable conformers may be involved in macrocyclization during biosynthesis. The predominantly configured C18 in chejuenolides may be attributed to the *s-cis* 1-azatriene motif, which subsequently adopts the *Re*-addition of Mannich addition (Scheme 6A). This energetically favorable conformer may also be responsible for the formation of 1,2-dihydropyridine and [1,5]-H shift products during macrocyclization (the key structural motifs shown in Scheme 6C).^29,43^ The stereoisomer for C2 is correlated to the conformer of δ-lactone in which a visually outside-orientated enol form (**TS-I**) gives **18a** and an inside form (**TS-II**) to **18b**. Accordingly, the cyclization event of an inside enol form toward the *Si*-Mannich addition (**TS-III**) ensures the generation of macrocycle **18c**. Moreover, the remote stereocenter at C13 has a significant impact on the conformation preference to regulate the stereochemical outcome in the cyclization of **6a** and the C4,C5-diastereoisomer **6c**. The linear polyene **6b** containing a *Z*-alkene (ΔC_16_–C_17_) is literally congested for conformational preorganization in the macrocyclization to give chejuenolide C (**3**) and 2-*epi*-chejuenolide C (**26**), and the latter structure has not yet been discovered from natural resources.

Alternatively, the side products derived from electrocyclization and hydride shift might not exclude an alternative conformer that features an *s*-cis of the C15–C16 bond and an *s*-trans of the C17–C18 bond, resulting in the identical stereochemical outcome of compound **18a** via the *Re*-facial Mannich addition (Scheme 6B). The conformational preference^44^ between **TS-I** and **TS-IV** remains unclear at this stage, and that demands further experimental and theoretical investigations.

### Biosynthetic Implication

The macrocycles derived from the aforementioned Mannich-based cyclization were susceptible to decarboxylation after the removal of the corresponding silyl groups. Most of them were not stable enough for instrumental characterization at ambient temperature, except for lactone **4** (half-life time: *t*_1/2_, ∼120 h at 0 °C), because of the absence of a lactamide-derived hydrogen bond at C18 as in lankacidin antibiotics. Although we are unable to establish a complete landscape for decarboxylation and protonation, the product distribution suggests that chejuenolide B (**2**) is only isolable under kinetic control, whereas the formation of chejuenolide A (**1**) is thermodynamically favored, suggesting that the given decarboxylation and protonation likely occurred with minimal enzyme participation.^45−47^

Differing from the stereochemical outcome from macrocyclization in the synthesis of lankacidins,^28^ the formation of **18a**, **21**, and **29a** (substituents at C2 vs C5) as the dominated *trans*-stereoisomer (correlation of C2–Me and C5–H) and (18*S*)-Mannich adduct **18a** suggest an alternative conformational preference in the ring-forming event. The stereochemical outcome in lankacidin biosynthesis as well as the possible stereoisomers identified within the trajectory of biomimetic synthesis of chejuenolides may suggest coevolution of the cyclase with the product stereochemistry. This notion was also spotlighted on the stereoisomers derived from thio-Michael cyclization in lanthipeptide biosynthesis.^48^ Although simulation of the reactive site in the enzyme is not possible owing to the lack of structural information of the amino oxidase (*cheE*) co-crystallized with any cyclized product,^41^ the two carbonyl groups in the β-oxo-δ-lactone ring may be interconvertible through noncovalent interactions with essential amino residues. Moreover, the possible orientation of the acetyl or lactyl group by the respective amino residues (i.e., arginine in *Lkc*E^41^) in the active site of the enzyme would require a large entropic contribution for the conformational change, posing an intriguing challenge for future mechanistic research.

The consistency of the absolute configuration in the δ-lactone ring in the linear precursors O3P2^38^ to LC-KA05-2 for lankacidins corresponds to the common gene information at the early stage of biosynthesis in various hosts. Although the phylogenetic tree pattern of the designated species has yet to be elucidated, the given substrate promiscuity in the fascinating macrocyclization holds the potential in developing a novel approach to access other polyene macrocycles that complements those derived from genome mining.^49,50^ Moreover, the macrocyclization of C4 and C5 stereoisomers and the implementation of tunable modules in the precursor enhance the capacity of the biomimetic approach to the synthesis of complex molecules.

In conclusion, the first synthesis and confirmation of the absolute configurations of chejuenolides A–C have been achieved via biomimetic macrocyclization. The propensity for decarboxylation of the cryptic lactone may represent a novel strategy for gaining access to diverse polyene macrocycles with editable modules and functional groups, providing a novel chemical space for future biological research. Moreover, the stereochemistry of the proposed biosynthetic precursor was clarified through modular synthesis to demonstrate the consistency of the absolute configurations at C4 and C5 in lanckacidins and chejuenolides, indicating a similarity in the biosynthesis of the two polyketide macrocycles. The biomimetic strategy has the additional advantage of fostering an undiscovered reservoir of incidental congeners along the biogenesis pathway. However, the stereoselectivity of macrocyclization may imply an underestimated challenge in the conformational discrimination of the given substrate by enzymes in the biosynthetic machinery. These efforts provide an intriguing chemical basis for future biosynthetic studies to reveal the unique macrocyclization in the context of the development of novel structures for medical applications.

## Supporting Information Available

Experimental procedures, characterization data, and structural assignments (PDF)
Crystallographic information for **18a** (CIF)
Crystallographic information for chejuenolide A (**1**) (CIF)
Crystallographic information for 2,18-*bisepi*-chejuenolide A (**20**) (CIF)
Crystallographic information for **21** (CIF)
Crystallographic information for **30a** (CIF)

## Supplementary Material

oc2c01096_si_001.pdf

oc2c01096_si_002.cif

oc2c01096_si_003.cif

oc2c01096_si_004.cif

oc2c01096_si_005.cif

oc2c01096_si_006.cif
