# Supplementary material for: Biomimetic Synthesis of Chejuenolides A–C by a Cryptic Lactone-Based Macrocyclization: Stereochemical Implications in Biosynthesis
Source: ACS Cent Sci. 2023 Jan 4;9(1):84–92. doi: 10.1021/acscentsci.2c01096 (PMC9881209; doi:10.1021/acscentsci.2c01096)
Supplement: Supplementary file 1 — oc2c01096_si_001.pdf [file oc2c01096_si_001.pdf]

Supporting Information for

Biomimetic Synthesis of Chejuenolides A-C by A Cryptic Lactone-based Macrocyclization: Stereochemical Implication in Biosynthesis

Bingbing Zhang,[1,2,#] Kuan Zheng,[1,#] and Ran Hong[1,2,*]

[1] CAS Key Laboratory of Synthetic Chemistry of Natural Substances, Center for Excellence in Molecular Synthesis, Shanghai Institute of Organic Chemistry, Chinese Academy of Sciences, Shanghai 200032

[2] University of Chinese Academy of Sciences, Beijing 101419, China

# These authors contributed equally to this work.

* Correspondence: rhong@sioc.ac.cn

Contents

1. General Procedures ········· S2
2. Experimental Procedures and Compound Characterizations ········· S3
3. NMR Comparisons of the Reported O3P2 and Synthetic Stereoisomers ········· S42
4. NMR Comparisons of the Reported and Synthetic chejuenolide A, B, and C ········· S50
5. X-Ray Crystallographic Information ········· S56
6. ^{1}H and ^{13}C NMR Spectra of Compounds ········· S61

1. General procedures

All reactions were conducted in oven-dried glassware fitted with rubber septa and magnetically stirred under N_2 atmosphere unless otherwise noted. Air-sensitive reagents and solutions were transferred via syringe or cannula, and were introduced to the reaction vessels through rubber septa. Solids were added under inert gas counter flow or were dissolved in appropriate solvents. All the solvents were dried using standard procedure and distilled before use. All reagents were purchased at the highest commercial quality and used without further purification, unless otherwise stated. Yields refer to chromatographically and spectroscopically homogeneous material. Reactions were monitored by thin layer chromatography (TLC) supplied by Yantai Jiangyou Silicon Material Company (China). Visualization was accomplished with UV light or basic aqueous potassium permangante ($KMnO_4$). Chromatography was achieved using forced flow (flash chromatography) of the indicated solvent system on 230-400 mesh silica gel (Silicycle® flash F60) unless otherwise noted. Preparative thin layer chromatography separations were carried out on E. Merck silica gel plates (0.25 or 0.50 mm, 60F–254). Optical rotations were measured on a Jasco P–1030 series digital polarimeter using a 100 mm path-length cell at 589 nm. Infrared spectra were recorded as thin films on NaCl plates on a Perkin–Elmer 983 or Digital FT-IR spectrometer and are reported in frequencies of absorption given in reciprocal centimeters (cm^{-1}). ^{1}H NMR and ^{13}C-NMR spectra were recorded on Varian mercury–400, Bruker AM–400, and Bruker AV–500 spectrometers. Chemical shifts for ^{1}H and ^{13}C NMR spectra are expressed in parts per million (ppm, δ scale) downfield from $SiMe_4$ relative to the residual ^{1}H and ^{13}C signals of the solvent ($CDCl_3$: δ 7.26, 77.16 ppm; CD_3OD: δ 3.31, 49.00 ppm; C_6D_6: δ 7.16, 128.1; DMSO-d_6: δ 2.50, 39.52 ppm; acetone-d6: δ 2.05, 29.84, 206.26) and the multiplicities are presented as follows: s = singlet, d = doublet, t = triplet, m = multiplet or unresolved, q = quartet, br = broad, app = apparent. 2D NMR techniques such as homonuclear correlation spectroscopy (COSY), heteronuclear single quantum coherence (HSQC) and heteronuclear multiple bond coherence (HMBC) were used to assist signal assignment. Nuclear Overhauser enhancement spectroscopy (NOESY) was conducted for further elucidation of 3D structures of the products. High-resolution mass spectra (HRMS) were acquired through the National Center for Organic Mass Spectrometry in Shanghai, Shanghai Institute of Organic Chemistry (SIOC, CAS) and determined on a Thermo Fisher Scientific LTQ FT Ultra mass spectrometer with DART (Direct Analysis in Real Time) ionization performed in positive mode.

2. Experimental Procedures and Compound Characterizations.

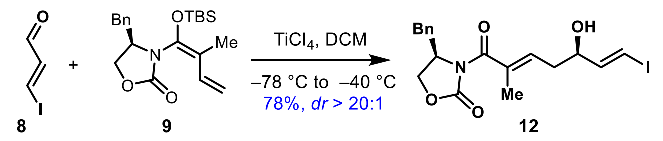

To a solution of (*E*)-3-iodoacrolein **8** (3.49 g, 19.2 mmol, 2.0 equiv) in CH_2Cl_2 (50 mL) was added $TiCl_4$ (1.0 M in CH_2Cl_2, 9.6 mL, 9.6 mmol, 1.0 equiv) at −78 °C. The resulting light yellow mixture was stirred for 30 min and a solution of vinylketene silyl *N,O*-acetal **9** (3.60 g, 9.6 mmol, 1.0 equiv) in CH_2Cl_2 (50 mL) was added. Stirring was continued for 36 hours at the same temperature before the reaction mixture was warmed up to −40 °C, stirred for further 10 h, and quenched with a mixed solution of saturated Rochelle Salt (*aq.*, 70 mL) and $NaHCO_3$ (*aq.*, 70 mL) in the dark. Upon warming to room temperature, the leaf green-colored organic layer was separated and the aqueous phase was briefly extracted with CH_2Cl_2 (2 x 40 mL). The combined organics were dried over Na_2SO_4, filtered and *immediately* concentrated under vacuum. Purification by flash column chromatography on silica gel (petroleum ether/EtOAc: 20/1 to 3/1) gave alcohol **12** (3.30 g, 78%) as a pale-yellow sticky oil, which easily solidified by exposure to a solvent mixture of *n*-pentane and diethyl ether (10/1, v/v). The enantiomer of **12** was reported in our previous work[1] with a specific optical rotation of $[\alpha]_D^{20} = -28.4$ (c = 1.13 in $CHCl_3$).

Data for alcohol **12**: $[\alpha]_D^{25}$ = +28.9 (c = 1.06 in $CHCl_3$); **^{1}H NMR** (400 MHz, DMSO-d_6): δ = 7.34-7.20 (m, 4H), 6.60 (dd, J = 14.4, 5.6 Hz, 1H), 6.39 (dd, J = 14.5, 1.3 Hz, 1H), 5.98 (tq, J = 7.1, 1.3 Hz, 1H), 4.69 (m, 1H), 4.36 (t, J = 8.5 Hz, 1H), 4.19 (dd, J = 8.7, 4.7 Hz, 1H), 4.07 (m, 1H), 3.07 (dd, J = 13.5, 3.5 Hz, 1H), 2.97 (dd, J = 13.5, 7.8 Hz, 1H), 2.27 (m, 2H), 1.78 (d, J = 1.5 Hz, 3H) ppm; **^{13}C NMR** (100 MHz, DMSO-d_6): δ = 170.9, 152.8, 149.1, 135.6, 132.8, 132.3, 129.5, 128.6, 126.9, 77.8, 71.9, 66.3, 54.6, 36.4, 35.2, 13.7 ppm; **IR** (thin film): ν_{max} = 3495, 2963, 2920, 1782, 1683, 1352, 1302, 1262, 1213, 1095, 1032, 801, 704 cm^{-1}; **HRMS-DART** (*m/z*): calcd. for $C_{18}H_{21}O_4NI$ $[M + H]^+$: 442.0510, found: 442.0511.

Reference

1. Zheng, K.; Shen, D. F.; Hong, R. *J. Am. Chem. Soc.* **2017**, *139*, 12939-12942.

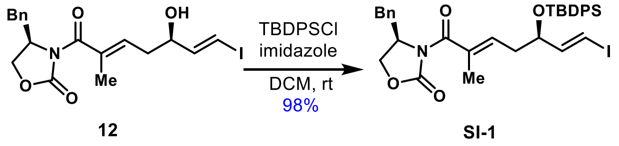

To a solution of **12** (2.43 g, 5.5 mmol, 1.0 equiv) and imidazole (1.14 g, 16.5 mmol, 3.0 equiv) in CH_2Cl_2 (20 mL) was added TBDPSCl (1.71 g, 6.1 mmol, 1.1 equiv) at 0 °C. The reaction mixture was warmed to room temperature, stirred for 5 hours, and carefully quenched with saturated *aq*. $NaHCO_3$ (50 mL), After extraction with CH_2Cl_2 (2 × 25 mL), the combined extracts were dried over Na_2SO_4, and concentrated. The residue oil was purified by flash column chromatography on silica gel (petroleum ether/EtOAc: 20/1 to 10/1) to give silyl ether **SI-1** (3.66 g, 98% yield) as a colorless oil. The enantiomer of **SI-1** was reported in our previous work[1] with a specific optical rotation of $[\alpha]_D^{21}$ = +5.3 (*c* = 0.69 in $CHCl_3$).

Data for silyl ether **SI-1**: $[\alpha]_D^{25}$ = −6.1 (*c* = 0.70 in $CHCl_3$); **^{1}H NMR** (400 MHz, $CDCl_3$): δ = 7.67-7.61 (m, 4H), 7.45-7.27 (m, 9H), 7.19 (m, 2H), 6.55 (dd, *J* = 14.4 Hz, 6.8 Hz, 1H), 6.08 (d, *J* = 14.4 Hz, 1H), 5.95 (t, *J* = 7.2 Hz, 1H), 4.67 (m, 1H), 4.23-4.18 (m, 2H), 4.12 (dd, *J* = 8.8 Hz, 5.2 Hz, 1H), 3.32 (dd, *J* = 13.6 Hz, 3.2 Hz, 1H), 2.76 (dd, *J* = 13.6 Hz, 9.6 Hz, 1H), 2.33 (m, 2H), 1.73 (s, 3H), 1.57 (s, 6H), 1.07 (s, 9H) ppm; **^{13}C NMR** (100 MHz, $CDCl_3$): δ = 171.4, 152.8, 147.2, 135.8 (2 peaks), 135.1, 133.5, 133.1 (2 peaks), 133.0, 129.9 (2 peaks), 129.4, 128.9, 127.7 (2 peaks), 127.3, 77.9, 74.6, 66.4, 55.3, 37.5, 36.1, 27.0, 19.3, 13.7 ppm; **IR** (thin film): ν_{max} = 2961, 2928, 2856, 1790, 1683, 1261, 1111, 749, 703 cm^{-1}; **HRMS-DART** (*m/z*): calcd. for $C_{34}H_{39}O_4NISi$ $[M + H]^+$: 680.1688, found: 680.1691.

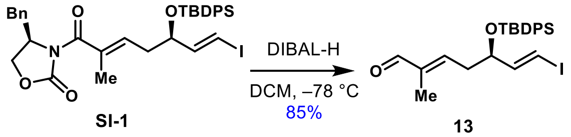

To a vigorously stirred solution of **SI-1** (2.85 g, 4.2 mmol, 1.0 equiv) in CH_2Cl_2 (45 mL) was added dropwise DIBAL-H (1.5 M in toluene, 5.6 mL, 8.4 mmol, 2.0 equiv) within 10 min at −78 °C. Upon complete conversion of the starting material (ca. 1 hour), the reaction mixture was carefully quenched with methanol (2.5 mL), saturated Rochelle salt (*aq*., 60

mL) and ether (50 mL), respectively. The resultant slurry was vigorously stirred for 6 hours at room temperature. The separated aqueous layer was extracted with ether (30 mL) and then the combined organic layers were dried over Na_2SO_4 and concentrated. Purification by silica gel flash column chromatography (petroleum ether/EtOAc: 10/1) gave aldehyde **13** (1.80 g, 85% yield) as a colorless oil. The enantiomer of **13** was reported in our previous work[1] with a specific optical rotation of $[\alpha]_D^{21} = -34.2$ (c = 1.19 in $CHCl_3$).

Data for *O*-TBDPS protected enal **13**: $[\alpha]_D^{25} = +34.9$ (c = 1.11 in $CHCl_3$); **^{1}H NMR** (500 MHz, DMSO-d_6): δ = 9.28 (s, 1H), 7.60-7.55 (m, 4H), 7.49-7.40 (m, 6H), 6.58 (dd, J = 14.5, 6.6 Hz, 1H), 6.51 (m, 1H), 6.27 (dd, J = 14.5, 1.0 Hz, 1H), 4.39 (m, 1H), 2.58-2.44 (m, 2H), 1.51 (d, J = 1.3 Hz, 3H), 1.00 (s, 9H) ppm; **^{13}C NMR** (125 MHz, DMSO-d_6): δ = 195.1, 149.4, 146.9, 140.3, 135.4 (2 peaks), 132.9, 132.8, 130.1, 130.0, 127.9, 127.8, 79.5, 74.3, 36.1, 26.7, 18.9, 9.1 ppm; **IR** (thin film): ν_{max} = 2960, 2901, 2857, 2718, 1690, 1427, 1261, 1111, 941, 821, 702 cm^{-1}; **HRMS-DART** (*m/z*): calcd. for $C_{24}H_{33}O_2NISi$ $[M + NH_4]^+$: 522.1320, found: 522.1320.

Reference

1. Zheng, K.; Shen, D. F.; Hong, R. *J. Am. Chem. Soc.* **2017**, *139*, 12939-12942.

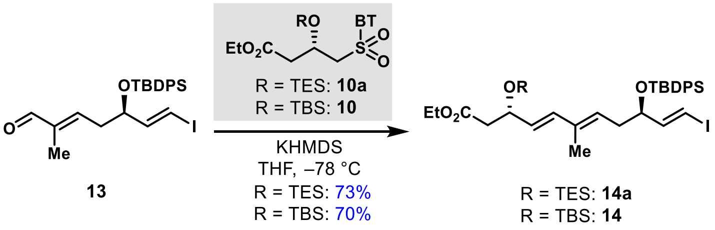

A solution of enal **13** (685 mg, 1.35 mmol, 1.0 equiv) and *O*-TES-protected BT-sulfone fragment **10a** (836 mg, 2.03 mmol, 1.5 equiv) in THF (10 mL) was cooled to −78 °C. KHMDS (1.0 M in THF, 2.3 mL, 2.30 mmol, 1.7 equiv) was added dropwise and the orange solution was stirred for 1 hour. Saturated NH_4Cl (*aq.*, 20 mL) was added to quench the reaction. The resultant mixture was warmed to room temperature, diluted with H_2O (10 mL) and extracted with EtOAc (25 mL). The combined organic extracts were dried over Na_2SO_4, filtered and concentrated *in vacuo*. The crude residue was purified by flash column

chromatography on silica gel (petroleum ether/EtOAc: 80/1 to 20/1) to afford C7-*O*-TES-protected ester **14a** (721 mg, 73% yield) as a pale-yellow oil. When *O*-TBS-protected sulfone fragment **10** was utilized under the same Barbier-type conditions, the corresponding C7-*O*-TBS-protected ester **14** can be obtained in an isolated yield of 70%.

Data for C7-*O*-TES-protected ester **14a**: $[\alpha]_D^{25}$ = −5.5 (*c* = 0.29 in $CHCl_3$); **^{1}H NMR** (500 MHz, $CDCl_3$): δ = 7.66-7.61 (m, 4H), 7.45-7.36 (m, 10H), 6.46 (dd, *J* = 14.4, 6.5 Hz, 1H), 6.14 (d, *J* = 15.6 Hz, 1H), 5.98 (d, *J* = 14.4 Hz, 1H), 5.52 (dd, *J* = 15.6, 7.2 Hz, 1H), 5.34 (t, *J* = 7.5 Hz, 1H), 4.62 (m, 1H), 4.17-4.07 (m, 3H), 2.56 (dd, *J* = 14.4, 8.1 Hz, 1H), 2.43 (dd, *J* = 14.5, 5.3 Hz, 1H), 2.34-2.23 (m, 2H), 1.54 (s, 3H), 1.25 (t, *J* = 7.1 Hz, 3H), 1.06 (s, 9H), 0.93 (t, *J* = 7.9 Hz, 9H), 0.57 (q, *J* = 7.9 Hz, 6H) ppm; **^{13}C NMR** (125 MHz, $CDCl_3$): δ = 171.3, 147.9, 136.0, 135.3, 134.9, 133.8, 133.5, 129.9 (2 peaks), 129.2, 127.8, 127.7, 127.2, 77.1, 75.6, 70.9, 60.5, 44.3, 36.3, 27.1, 19.4, 14.4, 12.6, 6.9, 5.0 ppm; **IR** (thin film): ν_{max} = 2957, 2935, 2876, 1738, 1606, 1463, 1428, 1370, 1262, 1236, 1161, 1112, 1079, 966, 944, 822, 740, 702, 613, 508 cm^{-1}; **HRMS-DART** (*m/z*): calcd. for $C_{36}H_{57}O_4NISi_2$ $[M + NH_4]^+$: 750.2865, found: 750.2866.

Data for C7-*O*-TBS-protected ester **14**: $[\alpha]_D^{25}$ = −2.3 (*c* = 0.97 in $CHCl_3$); **^{1}H NMR** (400 MHz, $CDCl_3$): δ = 7.66-7.60 (m, 4H), 7.45-7.35 (m, 6H), 6.46 (dd, *J* = 14.4, 6.6 Hz, 1H), 6.13 (d, *J* = 15.6 Hz, 1H), 5.97 (d, *J* = 14.4 Hz, 1H), 5.50 (dd, *J* = 15.6, 7.0 Hz, 1H), 5.34 (t, *J* = 7.6 Hz, 1H), 4.62 (td, *J* = 7.5, 4.9 Hz, 1H), 4.18-4.06 (m, 3H), 2.54 (dd, *J* = 14.4, 8.3 Hz, 1H), 2.42 (dd, *J* = 14.4, 5.0 Hz, 1H), 2.35-2.21 (m, 2H), 1.54 (s, 3H), 1.25 (t, *J* = 7.2 Hz, 3H), 1.05 (s, 9H), 0.86 (s, 9H), 0.04 (s, 3H), 0.01 (s, 3H) ppm; **^{13}C NMR** (100 MHz, $CDCl_3$): δ = 171.2, 147.9, 136.0 (2 peaks), 135.2, 134.8, 133.8, 133.5, 129.9 (2 peaks), 129.3, 127.7 (2 peaks), 127.1, 77.1, 75.6, 71.1, 60.4, 44.3, 36.3, 27.1, 25.9, 19.4, 18.2, 14.4, 12.6, -4.1, -4.9 ppm; **IR** (thin film): ν_{max} = 2956, 2930, 2894, 2857, 1738, 1472, 1428, 1362, 1257, 1161, 1112, 1079, 966, 940, 836, 777, 739, 702, 613, 507, 490 cm^{-1}; **HRMS-DART** (*m/z*): calcd. for $C_{36}H_{57}O_4NISi_2$ $[M + NH_4]^+$: 750.2865, found: 750.2860.

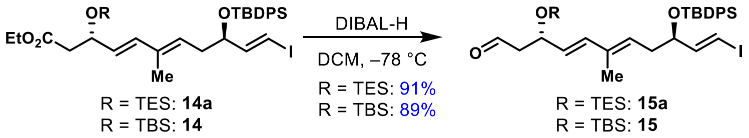

To a solution of C7-*O*-TES-protected ester **14a** (630 mg, 0.86 mmol, 1.0 equiv) in CH_2Cl_2 (10 mL) was added DIBAL-H (1.5 M in toluene, 0.7 mL, 1.03 mmol, 1.2 equiv) at −78 °C. The reaction mixture was quenched upon completion (ca. 30 min) by careful addition of methanol (0.5 mL), followed by a saturated Rochelle Salt (*aq*., 15 mL) and ether (20 mL). After 2 hours of vigorous stirring, the layers were separated and the aqueous layer was extracted with ether (20 mL). The combined organic extracts were dried over Na_2SO_4, filtered and concentrated. The obtained oily residue was purified by silica gel flash column chromatography (petroleum ether/EtOAc: 20/1) to give C7-*O*-TES-protected aldehyde **15a** (540 mg, 91% yield) as a pale-yellow oil. When *O*-TBS-protected substrate **14** was used, the corresponding C7-*O*-TBS-protected aldehyde **15** can be obtained in an isolated yield of 89%.

Data for C7-*O*-TES-protected aldehyde **15a**: $[\alpha]_D^{25}$ = −7.7 (*c* = 1.36 in $CHCl_3$); **^{1}H NMR** (500 MHz, DMSO-d_6): δ = 9.64 (t, *J* = 2.4 Hz, 1H), 7.59-7.55 (m, 4H), 7.48-7.40 (m, 6H), 6.49 (dd, *J* = 14.5, 6.7 Hz, 1H), 6.19 (d, *J* = 15.6 Hz, 1H), 6.14 (d, *J* = 14.5 Hz, 1H), 5.58 (dd, *J* = 15.6, 6.6 Hz, 1H), 5.40 (t, *J* = 7.4 Hz, 1H), 4.76 (app q, *J* = 6.2 Hz, 1H), 4.21 (app q, *J* = 6.1 Hz, 1H), 2.55-2.53 (m, 2H), 2.34-2.22 (m, 2H), 1.53 (s, 3H), 0.99 (s, 9H), 0.87 (t, *J* = 7.9 Hz, 9H), 0.53 (q, *J* = 7.9 Hz, 6H) ppm; **^{13}C NMR** (125 MHz, DMSO-d_6): δ = 202.0, 147.5, 135.4, 134.5, 134.1, 133.1, 133.0, 130.0, 129.9, 129.0, 127.8 (2 peaks), 127.3, 78.6, 75.2, 68.7, 51.4, 35.5, 26.7, 18.9, 12.4, 6.7, 4.4 ppm; IR (thin film): *v*max = 2955, 2876, 1726, 1427, 1111, 1076, 1006, 740, 702 cm^{-1}; **HRMS-DART** (*m/z*): calcd. for $C_{34}H_{53}O_3NISi_2$ $[M + NH_4]^+$: 706.2603, found: 706.2603.

Data for C7-*O*-TBS-protected aldehyde: **15**: $[\alpha]_D^{25}$ = +3.8 (*c* = 0.55 in $CHCl_3$); **^{1}H NMR** (400 MHz, $CDCl_3$): δ = 9.77 (t, *J* = 2.5 Hz, 1H), 7.65-7.60 (m, 4H), 7.45-7.35 (m, 6H), 6.46 (dd, *J* = 14.4, 6.6 Hz, 1H), 6.15 (d, *J* = 15.6 Hz, 1H), 5.98 (d, *J* = 14.4 Hz, 1H), 5.52 (dd, *J* = 15.6, 6.7 Hz, 1H), 5.36 (t, *J* = 7.5 Hz, 1H), 4.69 (td, *J* = 7.1, 4.8 Hz, 1H), 4.12 (q, *J* = 6.4 Hz, 1H), 2.63 (ddd, *J* = 15.6, 7.3, 2.8 Hz, 1H), 2.51 (ddd, *J* = 15.6, 4.8, 2.2 Hz, 1H),

2.36-2.22 (m, 2H), 1.55 (s, 3H), 1.05 (s, 9H), 0.87 (s, 9H), 0.06 (s, 3H), 0.03 (s, 3H) ppm; **^{13}C NMR** (100 MHz, $CDCl_3$): δ = 201.8, 147.8, 136.0 (2 peaks), 135.1, 135.0, 133.8, 133.5, 129.9 (2 peaks), 128.9, 127.7 (2 peaks), 127.6, 75.6, 69.7, 51.9, 36.3, 27.1, 25.9, 19.4, 18.2, 12.7, -4.0, -4.8 ppm; **IR** (thin film): ν_{max} = 2958, 2929, 2857, 1727, 1606, 1471, 1463, 1428, 1362, 1260, 1105, 1082, 1025, 966, 939, 836, 823, 802, 779, 740, 702, 613, 509, 488 cm^{-1}; **HRMS-DART** (*m/z*): calcd. for $C_{34}H_{53}O_3NISi_2$ $[M + NH_4]^+$: 706.2603, found: 706.2601.

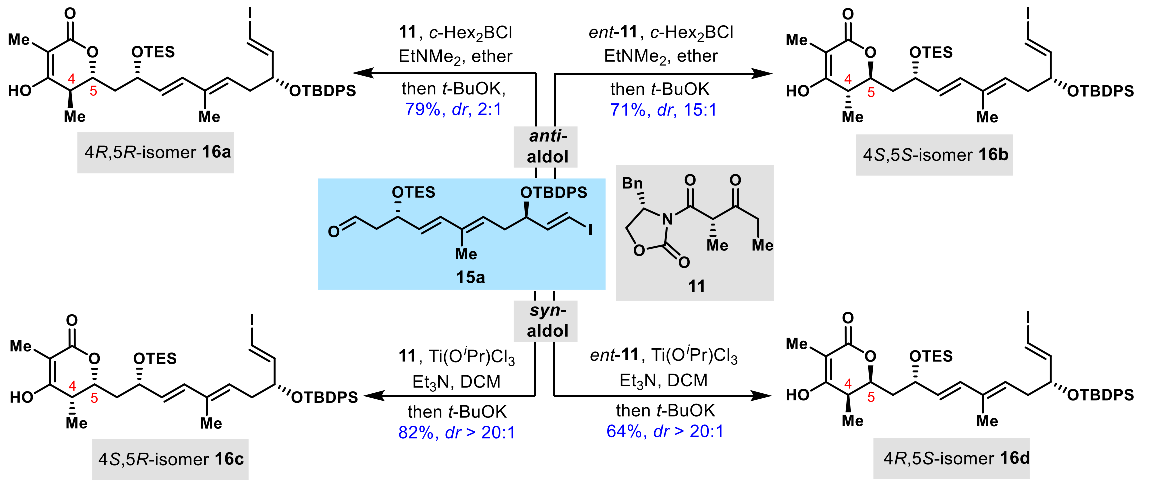

A solution of imide **11** (608 mg, 2.1 mmol, 1.2 equiv) in Et_2O (13 mL) was cooled to 0 °C. *c*-Hex_2BCl (1.0 M in hexane, 2.3 mL, 2.3 mmol, 1.3 equiv) and $EtNMe_2$ (170 mg, 2.3 mmol, 1.3 equiv) were added sequentially and the suspension was stirred at 0 °C for 2 h. After cooling to −78 °C, a freshly prepared solution of *O*-TES-protected aldehyde **15a** (1.20 g, 1.74 mmol, 1.0 equiv) in ether (8 mL) was added dropwise and additional ether (2 mL) was used to rinse the residue **15a** into the reaction. The reaction mixture was stirred at −78 °C for 18 h and −40 °C for 24 h, diluted with ether (8 mL) and quenched by sequential addition of methanol (8 mL) and phosphate buffer (*aq*., pH = 7, 8 mL). The separated aqueous layer was extracted with CH_2Cl_2 (2 x 20 mL) and the combined organic layers were dried over Na_2SO_4 and concentrated. Purification of the crude oil by flash column chromatography (petroleum ether/EtOAc: 20/1 to 8/1) afforded a mixture of *anti*-aldol adducts (*dr* = 2:1) and β-keto imide **11** (1.66 g in total, with a mole ratio of ca. 3:1 as indicated by ^{1}H NMR spectrum). This product mixture was dissolved in THF (60 mL) and treated with *t*-BuOK (1.0 M in THF, 1.7 mL, 1.7 mmol) at 0 °C *under oxygen-free atmosphere*. The reaction mixture was stirred at the same temperature for 1 h and quenched

with half-saturated NH_4Cl (*aq.*, 60 mL). After extraction with ether (2 x 30 mL), the combined organics were washed with brine (20 mL), dried over Na_2SO_4 and concentrated *in vacuo*. The residue was purified column chromatography (petroleum ether/EtOAc: 15/1) to give a ca. 2:1 diastereomeric mixture of lactones (Total: 1.10 g, 79% isolated yield from aldehyde **15a** over 2 steps). Analytically-pure samples of major diastereomer **16a** and minor diastereomer **16b** could be obtained by preparative TLC (petroleum ether/CH_2Cl_2, twice development). The minor 4*S*,5*S*-lactone isomer **16b** can also be diastereoselectively synthesized (*dr* = 15:1) in 71% overall yield following a similar two-step protocol using boron enolate prepared from *ent*-**11**.

Data for the major 4*R*,5*R*-lactone isomer **16a**: **$[\alpha]_D^{25}$** = +9.1 (*c* = 1.07 in $CHCl_3$); **^{1}H NMR** (500 MHz, CD_3OD): δ = 7.65-7.60 (m, 4H), 7.46-7.37 (m, 6H), 6.49 (dd, *J* = 14.5, 7.0 Hz, 1H), 6.20 (d, *J* = 15.7 Hz, 1H), 6.02 (dd, *J* = 14.5, 1.0 Hz, 1H), 5.51 (dd, *J* = 15.6, 7.6 Hz, 1H), 5.39 (t, *J* = 7.6 Hz, 1H), 4.39 (td, *J* = 8.0, 4.9 Hz, 1H), 4.19 (q, *J* = 6.1 Hz, 1H), 4.10 (m, 1H), 2.47 (m, 1H), 2.38 (m, 1H), 2.26 (m, 1H), 2.10 (ddd, *J* = 14.0, 9.3, 4.9 Hz, 1H), 1.75 (ddd, *J* = 13.4, 8.5, 4.6 Hz, 1H), 1.71 (d, *J* = 0.9 Hz, 3H), 1.63 (d, *J* = 1.2 Hz, 3H), 1.22 (d, *J* = 7.1 Hz, 3H), 1.04 (s, 9H), 0.94 (t, *J* = 8.0 Hz, 9H), 0.59 (q, *J* = 7.9 Hz, 6H) ppm; **^{13}C NMR** (125 MHz, CD_3OD): δ = 171.2, 170.6, 149.3, 137.3, 137.1, 136.3, 134.8, 134.7, 131.1, 131.0, 130.0, 129.0, 128.8, 128.7, 98.0, 79.1, 77.6, 77.1, 72.2, 43.1, 38.1, 37.2, 27.5, 20.1, 16.7, 13.0, 8.6, 7.2, 5.8 ppm; **IR** (thin film): ν_{max} = 3392, 3071, 2957, 2933, 2886, 2858, 1760, 1716, 1668, 1428, 1392, 1362, 1325, 1262, 1111, 1020, 968, 941, 822, 740, 703, 613, 508 cm^{-1}; **HRMS-DART** (*m/z*): calcd. for $C_{40}H_{61}O_5NISi_2$ $[M + NH_4]^+$: 818.3127, found: 818.3125.

Data for the minor 4*S*,5*S*-lactone isomer **16b**: **$[\alpha]_D^{25}$** = +10.8 (*c* = 0.84 in $CHCl_3$); **^{1}H NMR** (500 MHz, CD_3OD): δ = 7.65-7.60 (m, 4H), 7.46-7.36 (m, 6H), 6.49 (dd, *J* = 14.5, 6.9 Hz, 1H), 6.11 (d, *J* = 15.8 Hz, 1H), 6.03 (d, *J* = 14.5 Hz, 1H), 5.51 (dd, *J* = 15.6, 7.3 Hz, 1H), 5.35 (t, *J* = 7.5 Hz, 1H), 4.44 (m, 1H), 4.30 (ddd, *J* = 10.0, 4.5, 3.0 Hz, 1H), 4.18 (m, 1H), 2.43 (m, 1H), 2.36 (m, 1H), 2.26 (m, 1H), 1.83 (ddd, *J* = 14.2, 10.0, 2.9 Hz, 1H), 1.72 (d, *J* = 1.0 Hz, 3H), 1.71 (m, 1H), 1.59 (d, *J* = 1.2 Hz, 3H), 1.25 (d, *J* = 7.1 Hz, 3H), 1.04 (s, 9H), 0.96 (t, *J* = 8.0 Hz, 9H), 0.62 (q, *J* = 7.9 Hz, 6H) ppm; **^{13}C NMR** (125 MHz, CD_3OD):

δ = 171.2, 170.6, 149.3, 137.1, 137.0, 136.3, 136.0, 134.8, 134.6, 131.1, 131.0 (2 peaks), 128.8, 128.7, 128.6, 98.1, 78.6, 77.5, 77.1, 71.3, 43.7, 38.4, 37.1, 27.5, 20.1, 16.4, 12.9, 8.7, 7.3, 5.9 ppm; **IR** (thin film): ν_{max} = 3171, 3071, 2956, 2937, 2876, 1766, 1725, 1651, 1456, 1428, 1391, 1362, 1326, 1261, 1233, 1112, 1009, 967, 942, 822, 803, 741, 703, 613, 506 cm^{-1}; **HRMS-DART** (*m/z*): calcd. for $C_{40}H_{61}O_5NISi_2$ $[M + NH_4]^+$: 818.3127, found: 818.3128.

A solution of $TiCl_4$ (1.0 mol/L in CH_2Cl_2, 1.2 mL, 1.2 mmol, 1.2 equiv) in CH_2Cl_2 (6.5 mL) was cooled to 0 °C and treated with $Ti(O^iPr)_4$ (116 mg, 0.4 mmol, 0.12 mL, 0.4 equiv). The mixture was stirred for 15 min, at which time a solution of β-keto imide **11** (0.67 g, 2.3 mmol, 1.5 equiv) in CH_2Cl_2 (4.0 mL) was added dropwise at the same temperature. To the resultant yellow suspension was further added Et_3N (163 mg, 0.22 mL, 1.6 mmol, 1.6 equiv) and the obtained dark red solution was stirred for 2 h at the same temperature before cooling to −78 °C. A solution of *O*-TES-protected aldehyde **15a** (689 mg, 1.0 mmol, 1.0 equiv) in CH_2Cl_2 (5.0 mL) was slowly cannulated into the abovementioned enolate solution. After 4 h of stirring, the reaction was quenched by saturated NH_4Cl (*aq*., 15 mL) at −78 °C and the mixture was neutralized by pouring into a vigorously stirred, saturated $NaHCO_3$ (*aq*., 50 mL). The separated aqueous layer was extracted with CH_2Cl_2 (2 x 25 mL) and the combined organic extracts were dried over Na_2SO_4 and concentrated *in vacuo*. Chromatography purification (petroleum ether/EtOAc: 10/1) of the residue yielded a mixture of *syn*-aldol adduct (*dr* > 20:1) and keto imide **11** (948 mg in total). This intermediate was dissolved in THF (30 mL) and the solution was cooled to 0 °C. Then, *t*-BuOK (1.0 M in THF, 1.1 mL, 1.1 mmol) was added dropwise and stirring was continued for 1 h. Reaction was quenched by saturated NH_4Cl (*aq*., 20 mL) and H_2O (5 mL). Phases were separated and the aqueous layer was extracted with ether (20 mL). The combined organic layers were dried over Na_2SO_4 and concentrated. The residue oil was applied to chromatography on silica gel (petroleum ether/EtOAc: 6/1) to give 4*S*,5*R*-lactone **16c** (726 mg, contains ca. 9.6 w.t.% inseparable β-keto imide **11** as indicated by NMR analysis) with an overall yield of 82% (2 steps from aldehyde **15a**). The remaining 4*R*,5*S*-lactone isomer **16d** can also be diastereoselectively synthesized (*dr* > 20:1) in 64% overall yield following a similar same scale two-step protocol using titanium enolate prepared from *ent*-**11**.

Data for the 4*S*,5*R*-lactone isomer **16c**: ^{1}H NMR (500 MHz, CD_3OD, an inseparable 3.4:1 mixture with β-keto imide **11**, *the resonances of which are labelled with single asterisk*): δ = 7.63-7.59 (m, 4H), 7.43-7.35 (m, 6H), 7.33*-7.20* (m, 1.6H), 6.48 (dd, *J* = 14.5, 6.9 Hz, 1H), 6.16 (d, *J* = 15.6 Hz, 1H), 6.00 (dd, *J* = 14.5, 0.9 Hz, 1H), 5.49 (dd, *J* = 15.6, 7.7 Hz, 1H), 5.36 (t, *J* = 7.5 Hz, 1H), 4.77* (dd, *J* = 8.2, 3.2 Hz, 0.33H), 4.59* (q, *J* = 7.3 Hz, 0.33H), 4.42 (td, *J* = 7.9, 4.8 Hz, 1H), 4.31* (t, *J* = 8.7 Hz, 0.33H), 4.30 (m, 1H), 4.25* (dd, *J* = 9.1, 3.1 Hz, 0.33H), 4.18 (m, 1H), 3.15* (dd, *J* = 13.5, 3.3 Hz, 0.33H), 2.94* (dd, *J* = 13.6, 8.3 Hz, 0.33H), 2.66* (qd, *J* = 7.3, 3.2 Hz, 0.66H), 2.42-2.33 (m, 2H), 2.24 (m, 1H), 2.05 (ddd, *J* = 14.0, 9.1, 5.1 Hz, 1H), 1.68 (m, 1H), 1.67 (s, 3H), 1.64 (s, 3H), 1.38* (d, *J* = 7.3 Hz, 1H), 1.12 (d, *J* = 7.1 Hz, 3H), 1.02 (s, 9H), 1.01* (t, *J* = 7.2 Hz, 1H), 0.96 (t, *J* = 7.9 Hz, 9H), 0.62 (q, *J* = 7.9 Hz, 6H) ppm; ^{13}C NMR (125 MHz, CD_3OD, a mixture with **11**, *the resonances of which are labelled with single asterisk*): δ = 210.3*, 173.9, 172.0, 171.8*, 155.6*, 149.4, 137.5, 137.1, 136.8* 136.2, 134.7 (2 peaks), 131.0, 130.9, 130.6*, 130.1, 129.8*, 129.3, 128.8, 128.7, 128.2*, 98.0, 77.7, 76.1, 72.3, 67.9*, 56.3*, 54.1*, 40.8, 38.3*, 37.8, 37.2, 34.8*, 27.5, 20.1, 13.3*, 13.0, 11.2, 8.8, 7.8*, 7.2, 5.9 ppm; **HRMS-DART** (*m/z*): calcd. for $C_{40}H_{61}O_5NISi_2$ $[M + NH_4]^+$: 818.3127, found: 818.3130.

Data for the 4*R*,5*S*-lactone isomer **16d**: ^{1}H NMR (500 MHz, CD_3OD, an inseparable 2.9:1 mixture with β-keto imide *ent*-**11**, *the resonances of which are labelled with single asterisk*): δ = 7.66-7.60 (m, 4H), 7.46-7.37 (m, 6H), 7.33*-7.20* (m, 1.6H), 6.48 (dd, *J* = 14.5, 6.9 Hz, 1H), 6.17 (d, *J* = 15.7 Hz, 1H), 6.01 (dd, *J* = 14.5, 1.0 Hz, 1H), 5.56 (dd, *J* = 15.6, 7.2 Hz, 1H), 5.40 (t, *J* = 7.3 Hz, 1H), 4.77* (tt, *J* = 8.2, 3.2 Hz, 0.33H), 4.59* (q, *J* = 7.3 Hz, 0.33H), 4.55 (dt, *J* = 10.2, 2.9 Hz, 1H), 4.47 (m, 1H) 4.31* (t, *J* = 8.7 Hz, 0.33H), 4.25* (dd, *J* = 9.1, 3.1 Hz, 0.33H), 4.18 (m, 1H), 3.15* (dd, *J* = 13.5, 3.3 Hz, 0.33H), 2.94* (dd, *J* = 13.6, 8.3 Hz, 0.33H), 2.66* (qd, *J* = 7.3, 3.2 Hz, 0.66H), 2.40-2.25 (m, 3H), 1.83 (ddd, *J* = 14.2, 10.2, 2.9 Hz, 1H), 1.72 (d, *J* = 0.7 Hz, 3H), 1.61 (d, *J* = 1.3 Hz, 3H), 1.58 (m, 1H), 1.38* (d, *J* = 7.3 Hz, 1H), 1.10 (d, *J* = 7.1 Hz, 3H), 1.05 (s, 9H), 1.01* (t, *J* = 7.2 Hz, 1H), 0.93 (t, *J* = 7.9 Hz, 9H), 0.60 (q, *J* = 7.9 Hz, 6H) ppm; ^{13}C NMR (125 MHz, CD_3OD, a mixture with *ent*-**11** *the resonances of which are labelled with single asterisk*): δ = 210.3*, 174.0, 172.0, 171.8*, 155.6*, 149.3, 137.0 (2 peaks), 136.8*, 136.4, 136.0*, 134.8, 134.6, 131.2, 131.0 (2 peaks), 130.6*, 129.8*, 128.8, 128.7, 128.5, 128.2*, 98.0, 77.6, 77.1, 75.4,

70.8, 67.9*, 56.3*, 54.1*, 41.3, 38.3*, 38.2, 37.1, 34.8*, 27.5, 20.1, 13.3*, 12.9, 11.2, 8.7, 7.9*, 7.2, 5.8 ppm; **HRMS-DART** (*m/z*): calcd. for $C_{40}H_{61}O_5NISi_2$ $[M + NH_4]^+$: 818.3127, found: 818.3127.

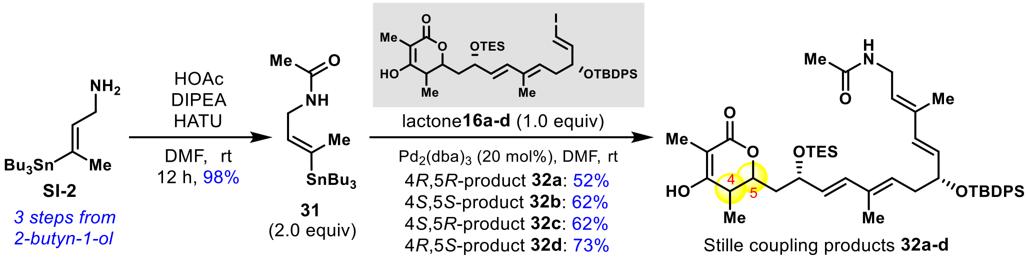

To a stirred solution of acetic acid (443 mg, 7.22 mmol, 1.3 equiv) in DMF (45 mL) at room temperature was sequentially added DIPEA (3.77 g, 28.86 mmol, 5.2 equiv) and HATU (2.77 g, 7.22 mmol, 1.3 equiv). The reaction mixture was stirred for 40 min before a solution of allylic amine **SI-2** (2.00 g, 5.55 mmol, 1.0 equiv) in DMF (15 mL) was added. Upon completion (ca. 12 h), the reaction mixture was partitioned between CH_2Cl_2 (80 mL) and phosphate buffer (*aq*., pH = 7, 80 mL). The separated organic layer was subsequently washed by H_2O (3 x 40 mL), brine (50 mL), dried over Na_2SO_4 and concentrated. The residue was then chromatographied on a silica gel column (petroleum ether/EtOAc: 15/1 to 5/1) to provide amide **31** (2.19 g, 98% yield) as a colorless oil.

Data for vinylstannane amide **31**: **^{1}H NMR** (400 MHz, $CDCl_3$): δ = 6.15 (br s, 1H), 5.56 (tq, J = 6.4, 1.8 Hz, $^3J_{Sn\text{-}H}$ = 66.8 Hz, 1H), 3.86 (app t, J = 5.8 Hz, 2H), 1.91 (s, 3H), 1.80 (s, $^3J_{Sn\text{-}H}$ = 44.6 Hz, 3H), 1.44-1.37 (m, 6H), 1.28-1.19 (m, 6H), 0.83-0.79 (m, 15H) ppm; **^{13}C NMR** (100 MHz, $CDCl_3$): δ = 170.0, 142.8, 135.5, 37.3, 29.1, 27.3, 23.1, 19.3, 13.7, 9.0 ppm; **IR** (thin film): ν_{max} = 3286, 3078, 2957, 2926, 2871, 2853, 1652, 1554, 1463, 1375, 1285, 1072, 875, 688, 662, 599 cm^{-1}; **HRMS-DART** (*m/z*): calcd. for $C_{18}H_{38}ON^{112}Sn$ $[M + H]^+$: 396.1996, found: 396.1995.

A solution of 4*R*,5*R*-vinyl iodide lactone **16a** (120 mg, 0.15 mmol, 1.0 equiv) and vinylstannane **31** (121 mg, 0.30 mmol, 2.0 equiv) in anhydrous DMF (3.5 mL) was degassed by three freeze-vacuum-thaw cycles. $Pd_2(dba)_3$ (28 mg, 0.03 mmol, 0.2 equiv) was added and the resultant dark brown solution was vigorously stirred for 36 h at room

temperature before partitioning between brine (30 mL) and EtOAc (30 mL). The separated aqueous layer was extracted with EtOAc (2 x 30 mL). The combined organic extracts were dried over Na_2SO_4 followed by filtration through a short pad of silica gel. The filtrate was concentrated and the remaining DMF in the residue was completely removed by high vacuum. Purification by flash column chromatography (petroleum ether/EtOAc: 4/1 to 1/2) furnished 4*R*,5*R*-amide lactone **32a** (61 mg, 52% yield) as a white foam.

Data for 4*R*,5*R*-amide lactone **32a**: $[\alpha]_D^{23}$ = +2.6 (*c* = 0.21 in $CHCl_3$); ^{1}**H NMR** (400 MHz, CD_3OD): δ = 7.66-7.61 (m, 4H), 7.44-7.33 (m, 6H), 6.20 (d, *J* = 15.6 Hz, 1H), 5.87 (d, *J* = 15.6 Hz, 1H), 5.64 (dd, *J* = 15.6, 6.8 Hz, 1H), 5.49 (dd, *J* = 15.6, 7.6 Hz, 1H), 5.45 (t, *J* = 7.4 Hz, 1H), 5.26 (t, *J* = 6.8 Hz, 1H), 4.39 (m, 1H), 4.28 (q, *J* = 6.4 Hz, 1H), 4.09 (m, 1H), 3.84 (d, *J* = 6.8 Hz, 2H), 2.49-2.40 (m, 2H), 2.29 (m, 1H), 2.09 (ddd, *J* = 14.0, 9.2, 5.0 Hz, 1H), 1.92 (s, 3H), 1.75 (m, 1H), 1.71 (s, 3H), 1.66 (s, 3H), 1.64 (s, 3H), 1.22 (d, *J* = 7.2 Hz, 3H), 1.03 (s, 9H), 0.94 (t , *J* = 7.6 Hz, 9H), 0.59 (q, *J* = 7.6 Hz, 6H) ppm; ^{13}**C NMR** (100 MHz, CD_3OD): δ = 173.0, 171.4, 170.6, 137.5, 137.1 (2 peaks), 136.8, 135.7, 135.4, 135.2, 132.3, 130.9, 130.7, 130.1, 129.6, 128.7, 128.5, 128.2, 97.9, 79.1, 75.7, 72.3, 43.1, 38.3 (2 peaks), 38.2, 27.6, 22.5, 20.1, 16.7, 12.9, 12.5, 8.6, 7.2, 5.8 ppm; **IR** (thin film): ν_{max} = 3292, 2963, 2929, 2877, 2857, 1762, 1723, 1651, 1552, 1458, 1428, 1390, 1361, 1261, 1111, 1078, 1017, 967, 800, 740, 703, 611, 505 cm^{-1}; **HRMS-DART** (*m/z*): calcd. for $C_{46}H_{67}O_6NNaSi_2$ $[M + Na]^+$: 808.4399, found: 808.4386.

When 4*S*,5*S*-vinyl iodide lactone **16b** (64 mg, 0.08 mmol) was used as starting material in the above-mentioned coupling protocol, long-chian 4*S*,5*S*-amide lactone **32b** (39 mg, 62% yield) can be obtained as a white foam. Data for 4*S*,5*S*-amide lactone **32b**: $[\alpha]_D^{24}$ = +4.6 (*c* = 0.59 in $CHCl_3$); ^{1}**H NMR** (400 MHz, CD_3OD): δ = 7.65-7.61 (m, 4H), 7.43-7.32 (m, 6H), 6.10 (d, *J* = 15.6 Hz, 1H), 5.88 (d, *J* = 15.6 Hz, 1H), 5.63 (dd, *J* = 15.6, 6.8 Hz, 1H), 5.49 (dd, *J* = 15.6, 7.2 Hz, 1H), 5.41 (t, *J* = 7.2 Hz, 1H), 5.27 (t, *J* = 6.8 Hz, 1H), 4.43 (m, 1H), 4.32-4.25 (m, 2H), 3.84 (d, *J* = 6.8 Hz, 2H), 2.46-2.35 (m, 2H), 2.29 (m, 1H), 1.93 (s, 3H), 1.83 (ddd, *J* = 14.2, 10.0, 2.8 Hz, 1H), 1.73 (s, 3H), 1.69 (m, 1H), 1.66 (s, 3H), 1.59 (s, 3H), 1.24 (d, *J* = 6.8 Hz, 3H), 1.03 (s, 9H), 0.95 (t , *J* = 7.6 Hz, 9H), 0.62 (q, *J* = 7.6 Hz, 6H) ppm; ^{13}**C NMR** (100 MHz, CD_3OD): δ = 172.9, 171.2, 170.6, 137.1, 137.0, 136.7,

136.2, 135.6, 135.3, 135.2, 132.2, 130.8, 130.7, 130.6, 129.7, 128.6, 128.5, 128.2, 98.1, 78.6, 75.7, 71.3, 43.7, 38.4, 38.3, 38.2, 27.6, 22.5, 20.2, 16.5, 12.9, 12.6, 8.8, 7.3, 5.9 ppm; **IR** (thin film): ν_{max} = 3294, 2959, 2942, 2881, 1763, 1724, 1651, 1551, 1457, 1390, 1362, 1261, 1111, 1013, 967, 821, 800, 740, 703, 611, 506 cm^{-1}; **HRMS-DART** (*m/z*): calcd. for $C_{46}H_{67}O_6NNaSi_2$ $[M + Na]^+$: 808.4399, found: 808.4387.

When 4*S*,5*R*-vinyl iodide lactone **16c** (650 mg, as an inseparable mixture with β-keto imide **11**, 90.4 w.t.% purity) was used as starting material in the above-mentioned coupling protocol, long-chain 4*S*,5*R*-amide lactone **32c** (357 mg, 62% yield based on **11**) can be obtained as a white foam. Data for 4*S*,5*R*-amide lactone **32c**: $[\alpha]_D^{25}$ = −10.5 (*c* = 1.00 in $CHCl_3$); **^{1}H NMR** (500 MHz, CD_3OD): δ = 7.64-7.60 (m, 4H), 7.44-7.32 (m, 6H), 6.16 (d, *J* = 15.6 Hz, 1H), 5.85 (d, *J* = 15.5 Hz, 1H), 5.63 (dd, *J* = 15.7, 7.0 Hz, 1H), 5.48 (dd, *J* = 15.6, 7.8 Hz, 1H), 5.42 (t, *J* = 7.6 Hz, 1H), 5.25 (t, *J* = 6.9 Hz, 1H), 4.42 (td, *J* = 8.0, 5.0 Hz, 1H), 4.31-4.24 (m, 2H), 3.85-3.83 (m, 2H), 2.44 (dt, *J* = 14.7, 7.5 Hz, 1H), 2.35 (m, 1H), 2.25 (m, 1H), 2.05 (ddd, *J* = 14.0, 9.1, 5.1 Hz, 1H), 1.92 (s, 3H), 1.72-1.64 (m, 10H), 1.12 (d, *J* = 7.1 Hz, 3H), 1.01 (s, 9H), 0.96 (t, *J* = 7.9 Hz, 9H), 0.61 (q, *J* = 8.0 Hz, 6H) ppm; **^{13}C NMR** (125 MHz, CD_3OD): δ = 173.9, 173.0, 172.9, 172.0, 137.7, 137.1, 136.7, 135.6, 135.3 (2 peaks), 135.1, 132.3, 130.8, 130.7, 130.4, 129.7, 128.7, 128.5, 128.2, 98.0, 76.1, 75.7, 72.4, 40.8, 38.4, 38.3, 37.8, 27.6, 22.5 (2 peaks), 20.1, 13.0, 12.6, 11.2, 8.8, 7.2, 5.9 ppm; **IR** (thin film): ν_{max} = 3286, 3071, 2956, 2926, 2879, 2691, 1651, 1557, 1456, 1428, 1393, 1310, 1262, 1239, 1112, 1076, 999, 967, 822, 800, 767, 740, 703, 611, 505 cm^{-1}; HRMS-DART (*m/z*): calcd. for $C_{46}H_{67}O_6NNaSi_2$ $[M + Na]^+$: 808.4399, found: 808.4395.

When 4*R*,5*S*-vinyl iodide lactone **16d** (510 mg, as an inseparable mixture with β-keto imide *ent*-**11**, 88.9 w.t.% purity) was used as starting material in the above-mentioned coupling protocol, long-chain 4*R*,5*S*-amide lactone **32d** (320 mg, 73% yield based on **11**) can be obtained as a white foam. Data for 4*R*,5*S*-amide lactone **32d**: $[\alpha]_D^{25}$ = +7.3 (*c* = 0.86 in $CHCl_3$); **^{1}H NMR** (500 MHz, CD_3OD): δ = 7.67-7.62 (m, 4H), 7.45-7.33 (m, 6H), 6.16 (d, *J* = 15.8 Hz, 1H), 5.87 (d, *J* = 15.7 Hz, 1H), 5.63 (dd, *J* = 15.6, 7.0 Hz, 1H), 5.54 (dd, *J* = 15.6, 7.3 Hz, 1H), 5.45 (t, *J* = 7.4 Hz, 1H), 5.27 (t, *J* = 6.9 Hz, 1H), 4.55 (dt, *J* = 10.2, 3.0 Hz, 1H), 4.46 (m, 1H), 4.28 (m, 1H), 3.86-3.83 (m, 2H), 2.41 (m, 1H), 2.35-2.28 (m, 2H),

1.92 (s, 3H), 1.83 (ddd, J = 14.1, 10.1, 2.9 Hz, 1H), 1.72 (d, J = 0.6 Hz, 3H), 1.66 (br s, 3H), 1.61 (d, J = 1.2 Hz, 3H), 1.59 (ddd, J = 14.1, 10.1, 2.8 Hz, 1H), 1.10 (d, J = 7.1 Hz, 3H), 1.04 (s, 9H), 0.93 (t, J = 7.9 Hz, 9H), 0.59 (q, J = 8.0 Hz, 6H) ppm; **^{13}C NMR** (125 MHz, CD_3OD): δ = 174.1, 173.0, 172.9, 172.0, 137.1 (2 peaks), 136.7, 136.2, 135.7, 135.4, 135.3 (2 peaks), 132.2, 130.9, 130.8, 130.7, 129.7, 128.7, 128.5, 128.2 (2 peaks), 98.0, 75.7, 75.4, 70.9, 41.3, 38.4, 38.3, 38.2, 27.6, 22.5 (2 peaks), 20.1, 12.9, 12.6, 11.1, 8.7, 7.2, 5.8 ppm; **IR** (thin film): ν_{max} = 3283, 2956, 2935, 1652, 1558, 1456, 1428, 1391, 1311, 1295, 1261, 1112, 1083, 1007, 966, 821, 801, 767, 740, 703, 611, 506, 489 cm^{-1}; **HRMS-DART** (*m/z*): calcd. for $C_{46}H_{67}O_6NNaSi_2$ $[M + Na]^+$: 808.4399, found: 808.4389.

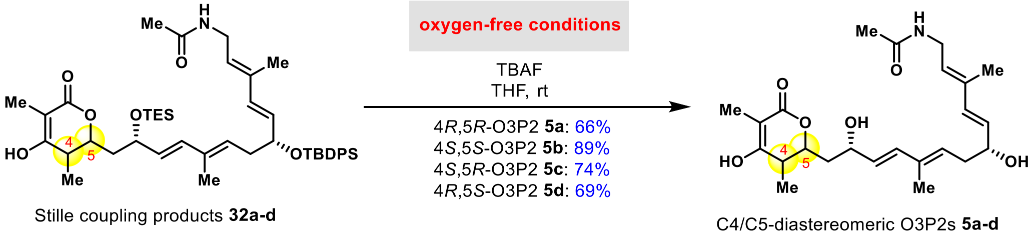

A solution of 4*R*,5*R*-amide lactone **32a** (157 mg, 0.2 mmol, 1.0 equiv) in THF (deoxygenated, 4.0 mL) was added dropwise TBAF (1.0 mol/L in THF, 0.8 mL, 0.8 mmol, 4.0 equiv) at room temperature under a positive pressure of argon. Saturated NH_4Cl (*aq.*, 20 mL) was added when the global desilylation was achieved as indicated by TLC (ca. 45 h) and layers were separated. The aqueous layer was extracted with EtOAc (3 x 20 mL) and the organic layers were combined and finally washed with brine (30 mL). The organics were dried over Na_2SO_4 and concentrated. Purification of the crude product mixture by silica gel flash column chromatography (CH_2Cl_2/MeOH: 20/1 to 15/1) provided 4*R*,5*R*-**O3P2** (**5a**) (57 mg, 66% yield) as a white foam. It was found that the ^{1}H and ^{13}C NMR data of this synthetic isomer *matches* those reported for the biosynthetic intermediate **O3P2**.

Data for 4*R*,5*R*-**O3P2** (**5a**): $[\alpha]_D^{23}$ = +60.1 (*c* = 0.99 in CH_3OH); **^{1}H NMR** (400 MHz, CD_3OD): δ = 6.29 (d, J = 15.6 Hz, 1H), 6.23 (d, J = 15.2 Hz, 1H), 5.69 (dd, J = 15.6, 6.4 Hz, 1H), 5.56 (dd, J = 15.4, 7.6 Hz, 1H), 5.54 (t, J = 7.2 Hz, 1H), 5.46 (t, J = 6.8 Hz, 1H), 4.28 (q, J = 6.4 Hz, 1H), 4.17 (m, 1H), 4.15 (m, 1H), 3.89 (d, J = 7.2 Hz, 2H), 2.50 (m, 1H), 2.44 (m, 1H), 2.40 (m, 1H), 2.08 (m, 1H), 1.93 (s, 3H), 1.79 (s, 3H), 1.78 (m, 1H),

1.77 (s, 3H), 1.72 (s, 3H), 1.25 (d, J = 7.2 Hz, 3H) ppm; **^{13}C NMR** (125 MHz, CD_3OD): δ = 173.0, 171.1, 170.6, 137.4, 136.9, 135.9, 135.2, 132.3, 129.7, 128.3, 98.0, 79.4, 73.3, 70.8, 42.0, 38.4, 38.0, 37.6, 22.5, 16.7, 12.9, 12.6, 8.6 ppm; **IR** (thin film): ν_{max} = 3321, 2972, 2933, 2890, 1755, 1716, 1657, 1549, 1456, 1392, 1326, 1287, 1264, 1117, 1088, 1047, 968, 880, 800, 765, 603 cm^{-1}; **HRMS-DART** (*m/z*): calcd. for $C_{24}H_{36}O_6N$ $[M + H]^+$: 434.2537, found: 434.2537.

When 4*S*,5*S*- amide lactone **32b** (370 mg, 0.47 mmol) was used as starting material in the above-mentioned global desilylation protocol (4 equiv of TBAF, 45 h), 4*S*,5*S*-**O3P2** (**5b**) (181 mg, 89% yield) can be obtained as a white foam. It was found that the ^{1}H and ^{13}C NMR data of this synthetic isomer *does not match* those reported for the natural product. Data for 4*S*,5*S*-**O3P2** (**5b**): $[\alpha]_D^{23}$ = −43.1 (*c* = 0.96 in CH_3OH); **^{1}H NMR** (400 MHz, CD_3OD): δ = 6.25 (d, J = 15.6 Hz, 1H), 6.22 (d, J = 15.6 Hz, 1H), 5.68 (dd, J = 15.6, 6.8 Hz, 1H), 5.59 (dd, J = 15.6, 6.8 Hz, 1H), 5.51 (t, J = 7.6 Hz, 1H), 5.46 (t, J = 6.8 Hz, 1H), 4.36 (m, 1H), 4.34 (m, 1H), 4.16 (q, J = 6.4 Hz, 1H), 3.89 (d, J = 6.8 Hz, 2H), 2.46 (m, 1H), 2.42 (m, 1H), 2.38 (m, 1H), 1.93 (s, 3H), 1.90 (ddd, J = 14.4, 10.4, 3.2 Hz, 1H), 1.79 (s, 3H), 1.74 (s, 3H), 1.72 (s, 3H), 1.71 (m, 1H), 1.27 (d, J = 7.2 Hz, 3H) ppm; **^{13}C NMR** (125 MHz, CD_3OD): δ = 173.0, 171.3, 170.7, 136.9, 136.0, 135.9, 135.2, 132.3, 130.7, 129.3, 128.3, 98.1, 78.8, 73.3, 69.5, 42.6, 38.5, 38.4, 37.5, 22.5, 16.6, 12.9, 12.6, 8.6 ppm; **IR** (thin film): ν_{max} = 3325, 2973, 2937, 2881, 1755, 1720, 1651, 1550, 1456, 1392, 1331, 1286, 1118, 1089, 1046, 967, 880, 767, 605 cm^{-1}; HRMS-DART (*m/z*): calcd. for $C_{24}H_{36}O_6N$ $[M + H]^+$: 434.2537, found: 434.2537.

When 4*S*,5*R*-amide lactone **32c** (280 mg, 0.36 mmol) was used as starting material in the above-mentioned global desilylation protocol (5 equiv of TBAF, 16 h), 4*S*,5*R*-**O3P2** (**5c**) (115 mg, 74% yield) can be obtained as a white foam. It was found that the ^{1}H and ^{13}C NMR data of this synthetic isomer *does not match* those reported for the natural product. Data for 4*S*,5*R*-**O3P2** (**5c**): $[\alpha]_D^{25}$ = −24.2 (*c* = 0.99 in CH_3OH); **^{1}H NMR** (500 MHz, CD_3OD): δ = 6.28 (d, J = 15.6 Hz, 1H), 6.23 (d, J = 15.7 Hz, 1H), 5.68 (dd, J = 15.7, 6.7 Hz, 1H), 5.57 (dd, J = 15.7, 7.5 Hz, 1H), 5.54 (t, J = 7.4 Hz, 1H), 5.46 (t, J = 6.9 Hz, 1H), 4.37 (ddd, J = 8.5, 5.2, 3.2 Hz, 1H), 4.29 (m, 1H), 4.16 (qd, J = 6.6, 1.2 Hz, 1H), 3.89 (d,

J = 7.0 Hz, 2H), 2.46-2.34 (m, 3H), 2.05 (ddd, J = 13.9, 8.6, 6.3 Hz, 1H), 1.93 (s, 3H), 1.79 (d, J = 1.1 Hz, 3H), 1.76 (d, J = 1.2 Hz, 3H), 1.70 (m, 1H), 1.70 (s, 3H), 1.12 (d, J = 7.0 Hz, 3H) ppm; **^{13}C NMR** (125 MHz, CD_3OD): δ = 174.2, 173.0, 172.2, 137.5, 136.9, 135.9, 135.2, 132.3, 129.9, 129.7, 128.3, 97.9, 76.4, 73.3, 70.6, 39.6, 38.4, 37.6, 37.5, 22.5, 12.9, 12.6, 11.0, 8.7 ppm; **IR** (thin film): ν_{max} = 3307, 2925, 2695, 1651, 1557, 1455, 1392, 1312, 1292, 1117, 1025, 994, 968, 882, 802, 768, 735, 702, 599 cm^{-1}; **HRMS-DART** (*m/z*): calcd. for $C_{24}H_{36}O_6N$ $[M + H]^+$: 434.2537, found: 434.2534.

When 4*R*,5*S*- amide lactone **32d** (250 mg, 0.32 mmol) was used as starting material in the above-mentioned global desilylation protocol (5 equiv of TBAF, 16 h), 4*R*,5*S*-**O3P2** (**5d**) (96 mg, 69% yield) can be obtained as a white foam. It was found that the ^{1}H and ^{13}C NMR data of this synthetic isomer *does not match* those reported for the natural product.

Data for 4*R*,5*S*-**O3P2** (**5d**): **$[\alpha]_D^{25}$** = +67.3 (*c* = 0.95 in CH_3OH); **^{1}H NMR** (500 MHz, CD_3OD): δ = 6.28 (d, J = 15.7 Hz, 1H), 6.23 (d, J = 15.7 Hz, 1H), 5.69 (dd, J = 15.8, 6.7 Hz, 1H), 5.64 (dd, J = 15.7, 6.7 Hz, 1H), 5.53 (t, J = 7.4 Hz, 1H), 5.46 (t, J = 7.1 Hz, 1H), 4.58 (dt, J = 9.7, 3.2 Hz, 1H), 4.36 (ddd, J = 10.0, 6.7, 3.1 Hz, 1H), 4.17 (app q, J = 6.6 Hz, 1H), 3.89 (d, J = 7.0 Hz, 2H), 2.47-2.34 (m, 3H), 1.93 (s, 3H), 1.89 (ddd, J = 14.1, 9.6, 3.2 Hz, 1H), 1.79 (d, J = 1.4 Hz, 3H), 1.76 (d, J = 1.3 Hz, 3H), 1.71 (s, 3H), 1.62 (ddd, J = 14.0, 10.0, 3.3 Hz, 1H), 1.12 (d, J = 7.0 Hz, 3H) ppm; **^{13}C NMR** (125 MHz, CD_3OD): δ = 174.2, 173.0, 172.2, 136.9, 136.1, 136.0, 135.2, 132.3, 130.7, 129.4, 128.3, 97.9, 75.8, 73.3, 69.2, 40.2, 38.4, 38.1, 37.5, 22.5, 12.9, 12.6, 11.1, 8.7 ppm; **IR** (thin film): ν_{max} = 3307, 2981, 2925, 2695, 1651, 1557, 1392, 1312, 1294, 1266, 1120, 1017, 994, 967, 885, 834, 799, 767, 736, 703, 605, 573 cm^{-1}; **HRMS-DART** (*m/z*): calcd. for $C_{24}H_{36}O_6N$ $[M + H]^+$: 434.2537, found: 434.2536.

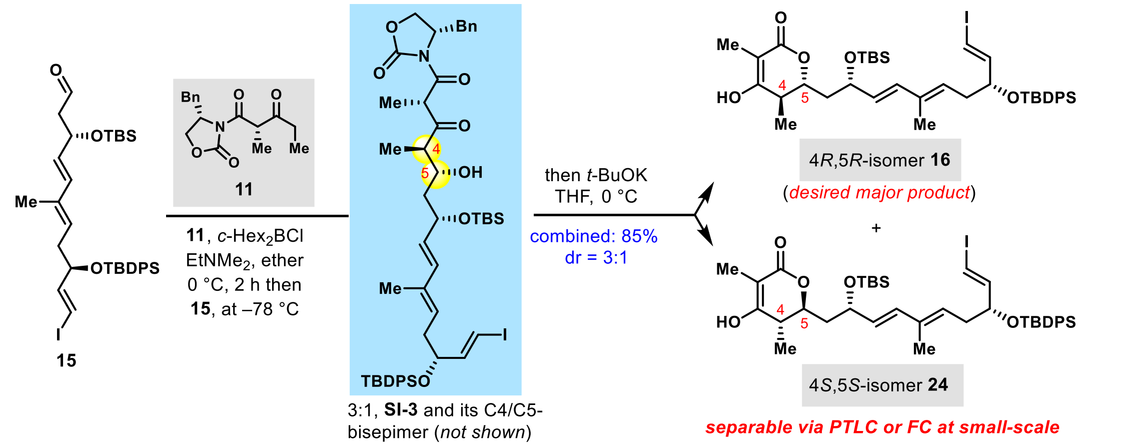

A solution of imide **11** (5.64 g, 19.5 mmol, 1.2 equiv) in ether (80 mL) was cooled to 0 °C. *c*-Hex$_2$BCl (1.0 M in hexane, 23.4 mL, 23.4 mmol, 1.5 equiv) and EtNMe$_2$ (2.5 mL, 23.4 mmol, 1.5 equiv) were added sequentially and the suspension was stirred at 0 °C for 2 h. After cooling to −78 °C, a solution of *O*-TBS-protected aldehyde **15** (10.86 g, 15.7 mmol, 1.0 equiv) in ether (40 mL) was added dropwise and additional ether (10 mL) was used to rinse the residue **15** into the reaction. The reaction mixture was stirred at −78 °C for 56 h, diluted with ether (40 mL) and quenched by sequential addition of methanol (40 mL) and phosphate buffer (*aq*., pH = 7, 40 mL). The separated aqueous layer was extracted with CH_2Cl_2 (3 x 60 mL) and the combined organic layers were dried over Na_2SO_4 and concentrated. Purification of the crude oil by flash column chromatography (petroleum ether/EtOAc: 20/1 to 6/1) afforded a mixture of *anti*-aldol adducts (**SI-3**/its 4,5-bisepimer = 3:1) and β-keto imide **11** (Total: 14.67 g, containing around 4.2 w.t.% imide as indicated by ^{1}H NMR spectrum). *For characterization purposes, an analytically-pure sample of major aldol adduct **SI-3** was obtained by preparative TLC (petroleum ether/CH_2Cl_2).* This diastereo-mixture was dissolved in THF (370 mL) and treated with *t*-BuOK (1.0 M in THF, 16.0 mL, 16 mmol) at 0 °C *under oxygen-free atmosphere*. The solution was stirred 0 °C for 1 h and quenched with half-saturated NH_4Cl (*aq*., 200 mL). After extraction with ether (3 x 100 mL), the combined organics were washed with brine (80 mL), dried over Na_2SO_4 and concentrated *in vacuo*. The residue was purified column chromatography (petroleum ether/EtOAc: 15/1) to give a ca. 3:1 diastereomeric mixture of lactones (Total: 10.69 g, 85% isolated yield from aldehyde **15**). Analytically-pure samples of major 4*R*,5*R*-diastereomer

16 and minor 4*S*,5*S*-diastereomer **24** could be obtained by preparative TLC (petroleum ether/CH_2Cl_2, twice development).

Data for major *anti*-aldol adduct **SI-3**: **$[\alpha]_D^{25}$** = +25.2 (*c* = 0.66 in $CHCl_3$); **^{1}H NMR** (500 MHz, $CDCl_3$): δ = 7.65-7.60 (m, 4H), 7.45-7.19 (m, 11H), 6.46 (dd, *J* = 14.4, 6.6 Hz, 1H), 6.05 (d, *J* = 15.6 Hz, 1H), 5.98 (dd, *J* = 14.4, 1.1 Hz, 1H), 5.44 (dd, *J* = 15.7, 7.6 Hz, 1H), 5.33 (t, *J* = 7.4 Hz, 1H), 4.88 (q, *J* = 7.2 Hz, 1H), 4.73 (ddt, *J* = 9.6, 7.9, 3.1 Hz, 1H), 4.39 (td, *J* = 8.1, 4.4 Hz, 1H), 4.23 (m, 1H), 4.16 (dd, *J* = 9.1, 2.8 Hz, 1H), 4.11 (m, 1H), 3.94 (ddt, *J* = 8.9, 7.0, 1.8 Hz, 1H), 3.60 (br, d, *J* = 2.1 Hz, 1H), 3.29 (dd, *J* = 13.4, 3.3 Hz, 1H), 2.84 (p, *J* = 7.0 Hz, 1H), 2.77 (dd, *J* = 13.4, 9.6 Hz, 1H), 2.34-2.22 (m, 2H), 1.68-1.52 (m, 5H), 1.48 (d, *J* = 7.3 Hz, 3H), 1.15 (d, *J* = 7.0 Hz, 3H), 1.05 (s, 9H), 0.87 (s, 9H), 0.07 (s, 3H), 0.01 (s, 3H) ppm; **^{13}C NMR** (125 MHz, $CDCl_3$): δ = 210.6, 171.0, 153.5, 147.9, 136.0 (2 peaks), 135.2 (2 peaks), 135.1, 133.8, 133.5, 130.0, 129.9, 129.8, 129.6, 129.1, 127.8, 127.7, 127.5, 127.4, 75.6, 75.4, 72.7, 66.4, 55.5, 52.7, 50.1, 41.4, 38.1, 36.3, 27.1, 26.0, 19.4, 18.1, 12.9, 12.7, -3.5, -4.6 ppm; **IR** (thin film): ν_{max} = 3508, 3070, 3029, 2959, 2929, 2857, 1783, 1716, 1698, 1605, 1471, 1455, 1428, 1391, 1360, 1259, 1213, 1111, 967, 940, 836, 823, 810, 702, 508 cm^{-1}; **HRMS-DART** (*m/z*): calcd. for $C_{50}H_{72}O_7N_2ISi_2$ $[M + NH_4]^+$: 995.3917, found: 995.3915.

Data for the major 4*R*,5*R*-lactone isomer **16**: **$[\alpha]_D^{25}$** = +11.6 (*c* = 0.92 in $CHCl_3$); **^{1}H NMR** (500 MHz, DMSO-d_6): δ = 10.41 (s, 1H), 7.58-7.54 (m, 4H), 7.47-7.39 (m, 6H), 6.50 (dd, *J* = 14.6, 6.8 Hz, 1H), 6.13 (d, *J* = 14.5 Hz, 1H), 6.10 (d, *J* = 15.5 Hz, 1H), 5.51 (dd, *J* = 15.6, 7.0 Hz, 1H), 5.35 (t, *J* = 7.5 Hz, 1H), 4.28 (m, 1H), 4.20 (m 1H), 4.02 (m, 1H), 2.45 (m, 1H), 2.33 (m, 1H), 2.23 (m, 1H), 1.93 (ddd, *J* = 13.9, 8.4, 5.8 Hz, 1H), 1.72 (ddd, *J* = 13.3, 7.6, 5.3 Hz, 1H), 1.61 (s, 3H), 1.56 (s, 3H), 1.14 (d, *J* = 6.9 Hz, 3H), 0.98 (s, 9H), 0.84 (s, 9H), 0.00 (s, 3H), -0.03 (s, 3H) ppm; **^{13}C NMR** (125 MHz, DMSO-d_6): δ = 168.2, 166.4, 147.5, 135.4, 134.7, 134.6, 133.1, 133.0, 129.9 (2 peaks), 129.1, 127.8, 127.7, 127.3, 96.2, 78.7, 76.5, 75.3, 70.4, 41.7, 35.9, 35.6, 26.8, 25.8, 18.9, 17.9, 16.6, 12.5, 8.7, -4.4, -4.8 ppm; **IR** (thin film): ν_{max} = 3136, 3071, 2954, 2929, 2857, 1765, 1724, 1651, 1471, 1462, 1428, 1391, 1362, 1258, 1111, 1074, 969, 938, 836, 821, 777, 740, 702, 613, 508,

488 cm^{-1}; **HRMS-DART** (*m/z*): calcd. for $C_{40}H_{61}O_5NISi_2$ [M + NH_4]$^+$: 818.3127, found: 818.3103.

Data for the minor 4*S*,5*S*-lactone isomer **24**: $[\alpha]_D^{25}$ = +7.6 (*c* = 1.05 in $CHCl_3$); **^{1}H NMR** (400 MHz, DMSO-*d*$_6$): δ = 10.40 (s, 1H), 7.59-7.54 (m, 4H), 7.47-7.37 (m, 6H), 6.48 (dd, *J* = 14.5, 6.6 Hz, 1H), 6.12 (d, *J* = 14.5 Hz, 1H), 6.08 (d, *J* = 15.7 Hz, 1H), 5.49 (dd, *J* = 15.7, 6.7 Hz, 1H), 5.35 (t, *J* = 7.3 Hz, 1H), 4.33 (m, 1H), 4.19 (m, 1H), 4.12 (m, 1H), 2.42 (m, 1H), 2.30 (m, 1H), 2.23 (m, 1H), 1.77 (m, 1H), 1.65 (m, 1H), 1.63 (s, 3H), 1.52 (s, 3H), 1.15 (d, *J* = 6.9 Hz, 3H), 0.98 (s, 9H), 0.85 (s, 9H), 0.04 (s, 3H), 0.02 (s, 3H) ppm; **^{13}C NMR** (100 MHz, DMSO-*d*$_6$): δ = 168.3, 166.4, 147.5, 135.3 (2 peaks), 134.5, 133.7, 133.1, 133.0, 130.0, 129.9, 129.8, 127.7 (2 peaks), 126.9, 96.3, 78.4, 76.0, 75.2, 69.4, 42.1, 36.4, 35.5, 26.7, 25.8, 18.9, 17.8, 16.2, 12.3, 8.8, -4.3, -4.9 ppm; **IR** (thin film): ν_{max} = 3185, 3071, 2954, 2929, 2857, 1760, 1723, 1659, 1471, 1462, 1427, 1389, 1361, 1259, 1111, 967, 940, 898, 836, 824, 778, 740, 702, 613, 508, 488 cm^{-1}; **HRMS-DART** (*m/z*): calcd. for $C_{40}H_{61}O_5NISi_2$ [M + NH_4]$^+$: 818.3127, found: 818.3122.

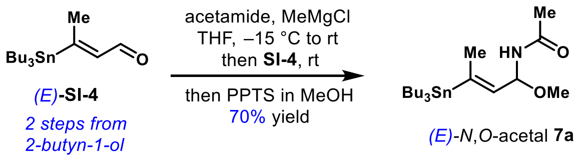

Methylmagnesium chloride (3.0 M in THF, 24.0 mL, 72 mmol, 3.5 equiv) was added dropwise to a stirred solution of acetamide (3.61 g, 60 mmol, 2.9 equiv) in THF (40 mL) at −15 °C. The slurry was warmed to room temperature, stirred for 15 min, and recooled to −15 °C before aldehyde **SI-4** (9.0 g, 25 mmol, 1.0 equiv) was added in one portion. After stirring for 17 h at room temperature, the reaction was quenched at −50 °C by addition of saturated $NaHCO_3$ (*aq.*, 80 mL) and partitioned between CH_2Cl_2 (100 mL) and H_2O (100 mL) while warming to room temperature. The organic layer was separated and the aqueous layer was extracted with CH_2Cl_2 (2 x 60 mL). The combined extracts were thoroughly dried over Na_2SO_4. The solvent of the filtrate was removed under reduced pressure at 20 °C and the residue oil was dissolved in anhydrous methanol (50 mL) and treated with pyridinium *p*-toluenesulfonate (130 mg, 0.5 mmol) at room temperature. Stirring was continued for 3 h and the reaction was quenched by saturated $NaHCO_3$ (*aq.*, 100 mL) at −50 °C and

partitioned between CH_2Cl_2 (100 mL) and H_2O (50 mL). After phase separation and extraction with CH_2Cl_2 (2 x 80 mL), the combined extracts were dried over Na_2SO_4 for 2 hours, filtered and concentrated. The residue was immediately purified by flash chromatography on silica gel (petroleum ether/EtOAc: 20/1 to 5/1) to afford *N*-acetyl *N,O*-acetal **7a** (7.60 g, 70% yield) as a light orange oil.

Data for *N*-acetyl *N,O*-acetal **7a**: **^{1}H NMR** (400 MHz, $CDCl_3$): δ = 5.86 (dd, *J* = 9.2, 6.4 Hz, 1H), 5.72 (br d, *J* = 9.2 Hz, 1H), 5.50 (dq, *J* = 6.4, 1.8 Hz, 1H), 3.35 (s, 3H), 2.03 (s, 3H), 1.93 (d, *J* = 1.8 Hz, 3H), 1.52-1.44 (m, 6H), 1.35-1.26 (m, 6H), 0.92-0.87 (m, 15H) ppm; **^{13}C NMR** (100 MHz, $CDCl_3$): δ = 170.0, 145.7, 136.6, 76.3, 54.6, 28.9, 27.2, 23.1, 20.0, 13.5, 9.0 ppm; **IR** (thin film): ν_{max} = 3288, 2957, 2927, 2872, 2853, 1661, 1538, 1464, 1375, 1268, 1130, 1112, 1068, 948, 940, 692, 669, 597 cm^{-1}; **HRMS-DART** (*m/z*): calcd. for $C_{18}H_{36}ON^{112}Sn$ $[M – OCH_3]^+$: 402.1819, found: 402.1809.

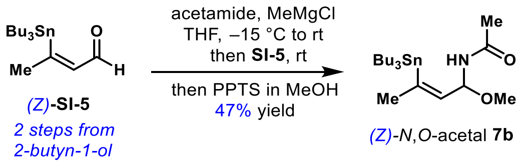

Methylmagnesium chloride (12.4 mL, 3M in THF, 37.2 mmol) was added dropwise to a vigorously stirred solution of acetamide (2.2 g, 37.2 mmol) in THF (50 mL) at 0 °C. The resulting mixture was stirred for 30 minutes at room temperature. Then the mixture was cooled to -15 °C, **SI-5** (6.7 g, 18.6 mmol) was added. After stirred for 1.5 hours at -15 °C, the reaction was quenched with saturated NH_4Cl (*aq.*, 200 mL), the resulting mixture was extracted with CH_2Cl_2 (100 mL x3), the combined organic layer was dried over anhydrous Na_2SO_4, filtered and concentrated, the crude hemiaminal was redissolved in MeOH (20 mL), PPTS (470 mg, 1.86 mmol) was added. The resulting mixture was stirred for 0.5 hour at room temperature and quenched by saturated $NaHCO_3$ (*aq.*, 50 mL), extracted with CH_2C_2 (50 mL x 3), the combined organic layer was dried over anhydrous Na_2SO_4, filtered and concentrated, the residue was purified by flash chromatography on silica gel

(petroleum ether/EtOAc = 5/1) to to afford *N*-acetyl *N*,*O*-acetal **7b** (3.80 g, 47% yield) as a colorless oil (unstable, transformed back into **SI-5** easily).

Data for *N*-acetyl *N*,*O*-acetal **7b**: **^{1}H NMR** (500 MHz, $CDCl_3$) δ 6.31 (d, *J* = 9.1 Hz, 1H), 5.95 (d, *J* = 6.3 Hz, 1H), 5.36 (d, *J* = 6.3 Hz, 1H), 3.25 (s, 3H), 1.96 (d, *J* = 8.7 Hz, 3H), 1.88 (s, 3H), 1.45 – 1.39 (m, 6H), 1.28 – 1.21 (m, 6H), 0.89 (t, *J* = 8.1Hz, 5H), 0.82 (t, *J* = 7.4 Hz, 10H) ppm; **^{13}C NMR** (125 MHz, $CDCl_3$) δ 170.0, 145.8, 136.3, 81.1, 54.9, 29.1, 27.4, 27.3, 23.3, 13.7, 10.5 ppm; **HRMS-ESI** (*m/z*): calcd. For $C_{46}H_{66}NO_6Si_2$ [M - OCH_3]$^+$: 402.1819, found: 402.1813.

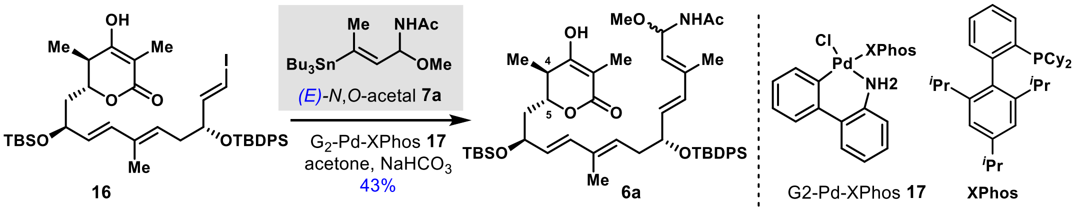

To a solution of lactone **16** (400 mg, 0.5 mmol) and (*E*)-*N,O*-acetal **7a** (260 mg, 0.6 mmol) in acetone (50 mL) under argon atmosphere was added $NaHCO_3$ (84 mg, 1 mmol) and Pd-G_2-XPhos (79 mg, 0.1 mmol), the resulting mixture was stirred for 2 days at room temperature and quenched by saturated KF (*aq*., 20 mL), diluted with brine (200 mL), the resulting mixture was extracted by ether (100 mL x 3), the combined organic layer was washed with brine (50 mL), dried over anhydrous Na_2SO_4, filtered and concentrated, the residue was purified by flash chromatography on silica (petroleum ether/acetone = 4/1) to afford the target compound **6a** (177 mg, 43%) as a yellow foam , which was unstable, submitted to next step directly.

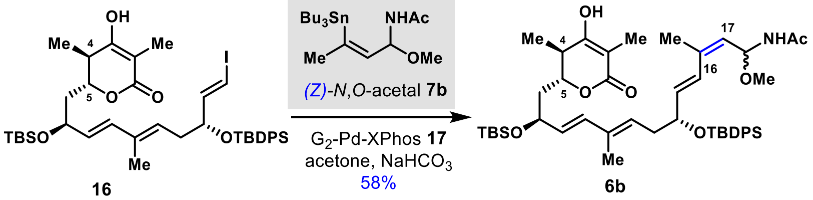

To a solution of **16** (1.45 g, 1.8mmol) and (*Z*)-*N,O*-acetal **7b** (1.1 g, 2.7 mmol) in acetone (130 mL) under argon atmosphere was added $NaHCO_3$ (168 mg, 2 mmol) and Pd-G_2-XPhos (456 mg, 0.58 mmol), the resulting mixture was stirred for 3 days at room temperature and quenched by brine (200 mL), the resulting mixture was extracted by ether

(100 mL x 3), the combined organic layer was washed with brine (50 mL), dried over anhydrous Na_2SO_4, filtered and concentrated, the residue was purified by column chromatography on silica (petroleum ether/acetone = 4/1) to afford the target compound **6b** (850 mg, 58%) as a yellow foam, which was not stable, submitted to next step directly.

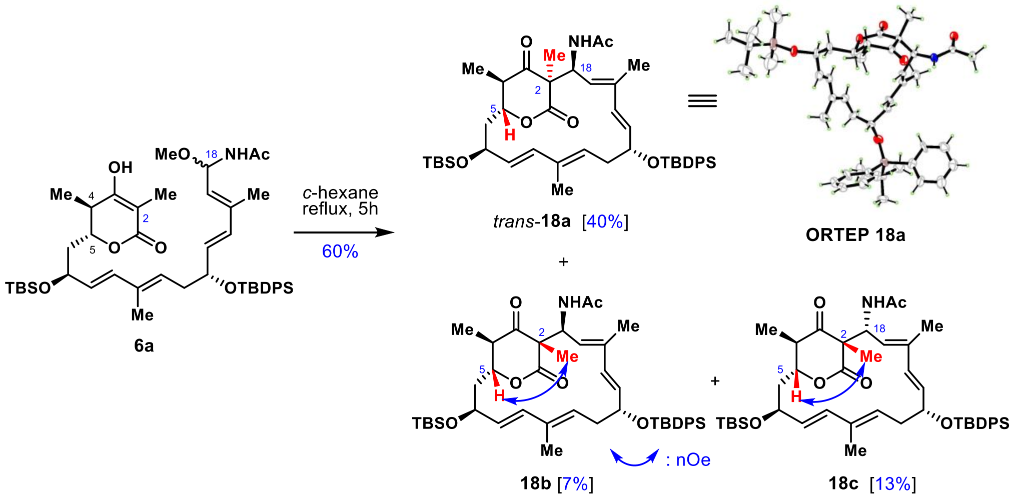

A solution of 1:1 diastereomeric mixture of *N*,*O*-acetal **6a** (170 mg, 0.21 mmol) in toluene (5 mL) was added to a refluxing cyclohexane (210 mL) by cannula transfer. The reaction mixture was stirred vigorously at reflux for 5 hours until the complete disappearance of starting material (monitored by TLC). The solution was cooled to ambient temperature and concentrated at vacuum to give a foam residue, which was purified by preparative TLC (petroleum ether / EtOAc = 2/1) to give the macrocycles **18a** (65 mg, 40% yield) as a white foam, **18b** (10 mg, 6.5% yield) as a white foam, **18c** (21 mg, 13% yield) as a white foam, respectively.

Data for **18a**: $[\alpha]_D^{25}$ = +34.9; **^{1}H NMR** (500 MHz, $CDCl_3$) δ 7.70 – 7.61 (m, 4H), 7.44 – 7.31 (m, 6H), 6.82 (d, *J* = 10.0 Hz, 1H), 6.03 (d, *J* = 15.6 Hz, 1H), 5.59 (dd, *J* = 15.6, 8.7 Hz, 1H), 5.40 (t, *J* = 10.4, 1H), 5.31 (d, *J* = 15.6, 1H), 5.17 (dd, *J* = 15.6, 7.3 Hz, 1H), 5.04 (t, *J* = 10.4 Hz, 1H), 4.80 (d, *J* = 10.7 Hz, 1H), 4.44 (td, *J* = 8.2, 4.4 Hz, 1H), 3.97 (td, *J* = 9.7, 6.0 Hz, 1H), 3.58 (td, *J* = 9.7, 2.0 Hz 1H), 2.71 (dq, *J* = 13.0, 6.5 Hz, 1H), 2.40 (ddd, *J* = 12.7, 8.5, 6.0 Hz, 1H), 2.18 (ddd, *J* = 12.7, 10.1, 8.5 Hz, 1H), 2.02 (s, 3H), 1.95 (ddd, *J* = 14.1, 10.2, 4.0 Hz, 1H), 1.81 (ddd, *J* = 11.2, 9.0, 2.2 Hz, 1H), 1.79 (d, *J* =

1.1 Hz, 3H), 1.50 (s, 3H), 1.27 (s, 3H), 1.04 (s, 9H), 0.84 (s, 9H), 0.01 (s, 3H), -0.01 (s, 3H) ppm; **^{13}C NMR** (125 MHz, $CDCl_3$) δ 210.7, 172.0, 169.3, 139.3, 137.0, 136.0, 135.9, 134.4, 134.4, 134.0, 133.9, 132.4, 129.8, 129.7, 129.4, 127.7, 127.6, 126.6, 125.2, 75.8, 75.3, 71.1, 59.2, 52.1, 45.8, 41.8, 36.2, 27.1, 26.0, 23.7, 23.3, 19.3, 18.3, 12.9, 12.1, 8.9, -4.2, -4.7 ppm; **IR** (thin film): υ_{max} = 3432, 2960, 2926, 2855, 1750, 1712, 1682, 1456, 1260, 1107 cm^{-1}; **HRMS-ESI** (*m/z*): calcd. For $C_{46}H_{66}NO_6Si_2$ $[M + H]^+$: 784.4423, found: 784.4423.

Data for **18b**: $[\alpha]_D^{25}$ = −61.2 (*c* = 1.04, $CHCl_3$); **^{1}H NMR** (600 MHz, $CDCl_3$) δ 7.73 – 7.59 (m, 4H), 7.39 (m, 6H), 6.62 (d, *J* = 10.0 Hz, 1H), 6.09 (d, *J* = 15.6 Hz, 1H), 6.04 (d, *J* = 15.5 Hz, 1H), 5.66 (dd, *J* = 15.5, 9.4 Hz, 1H), 5.59 (t, *J* = 10.4 Hz, 1H), 5.53 (t, *J* = 15.0 Hz 1H), 5.49 (dd, *J* = 15.6, 3.8 Hz 1H), 4.66 (d, *J* = 10.9 Hz, 1H), 4.62 (brs, 1H), 4.36 – 4.32 (m, 2H), 2.51 (qd, *J* = 13.4, 6.8 Hz, 1H), 2.30 (ddd, *J* = 15.2, 10.6, 2.3 Hz, 1H), 2.14 (dt, *J* = 15.4, 4.5 Hz, 1H), 2.11 – 2.09 (m, 2H), 2.03 (s, 3H), 1.86 (s, 3H), 1.45 (s, 3H), 1.42 (s, 3H), 1.29 (d, *J* = 6.7 Hz, 3H), 1.10 (s, 9H), 0.86 (s, 9H), 0.08 (s, 3H), 0.02 (s, 3H) ppm; **^{13}C NMR** (150 MHz, $CDCl_3$) δ 212.6, 170.2, 169.4, 139.3, 137.2, 136.4, 136.0, 134.4, 133.9, 133.7, 130.6, 129.9, 129.8, 129.6, 127.8, 127.7, 127.4, 123.8, 75.7, 717, 71.0, 57.3, 51.7, 46.6, 38.6, 34.9, 27.3, 26.0, 23.8, 21.2, 19.6, 18.2, 13.4, 12.6, 10.0, -3.6, -4.3 ppm; **IR** (thin film): υ_{max} = 3425, 3075, 3044, 2929, 2890, 2856, 1751, 1708, 1678, 1504, 1427, 1371, 1255 cm^{-1}; **HRMS-ESI** (*m/z*): calcd. For $C_{46}H_{66}NO_6Si_2$ $[M + H]^+$: 784.4423, found: 784.4433.

Data for **18c**: $[\alpha]_D^{25}$ = +61.0 (*c* = 0.30, $CDCl_3$) **^{1}H NMR** (400 MHz, $CDCl_3$) δ 7.71 – 7.55 (m, 4H), 7.45 – 7.30 (m, 6H), 6.29 (d, *J* = 10.0 Hz, 1H), 5.92 (d, *J* = 16.0 Hz, 1H), 5.55 (s, 1H), 5.51 (t, *J* = 10.0 Hz, 1H), 5.46 (s, 1H), 5.11 (dd, *J* = 16.1, 8.1 Hz, 1H), 4.80(t, *J* = 5.0 Hz, 1H), 4.77 (d, *J* = 10.0 Hz, 1H), 4.47 (td, *J* = 9.4, 5.1 Hz, 1H), 4.05 (dd, *J* = 10.3, 4.0 Hz, 1H), 3.34 (t, *J* = 10.9 Hz, 1H), 2.91 – 2.78 (m, 1H), 2.45 (ddd, *J* = 13.6, 11.2, 4.1 Hz, 1H), 2.31 – 2.23 (m, 1H), 2.02 (s, 3H), 2.02 – 1.96 (m, 1H), 1.93 (s, 3H), 1.65 – 1.54 (m, 1H), 1.52 (s, 3H), 1.29 (s, 3H), 1.03 (s, 9H), 0.96 (d, *J* = 6.7 Hz, 3H), 0.84 (d, *J* = 10.0 Hz, 9H), 0.02 (s, 3H), 0.01 (s, 3H) ppm; **^{13}C NMR** (100 MHz, $CDCl_3$) δ 208.6, 172.0, 169.0, 153.0, 142.0, 136.5, 136.0, 134.3, 134.0, 133.9, 129.7, 129.6, 127.8, 127.5, 127.5, 127.4,

120.0, 116.5, 76.6, 71.5, 70.9, 59.5, 51.7, 44.2, 41.3, 39.8, 27.1, 26.0, 25.0, 23.7, 19.2, 18.3, 16.3, 12.3, 9.0, -4.3, -4.7 ppm; **IR** (thin film): υ_{max} = 3447, 3075, 3044, 2955, 2929, 2856, 1752, 1714, 1490, 1251, 1120 cm^{-1}; **HRMS-ESI** (*m/z*): calcd. For $C_{46}H_{66}NO_6Si_2$ $[M + H]^+$: 784.4423, found: 784.4434.

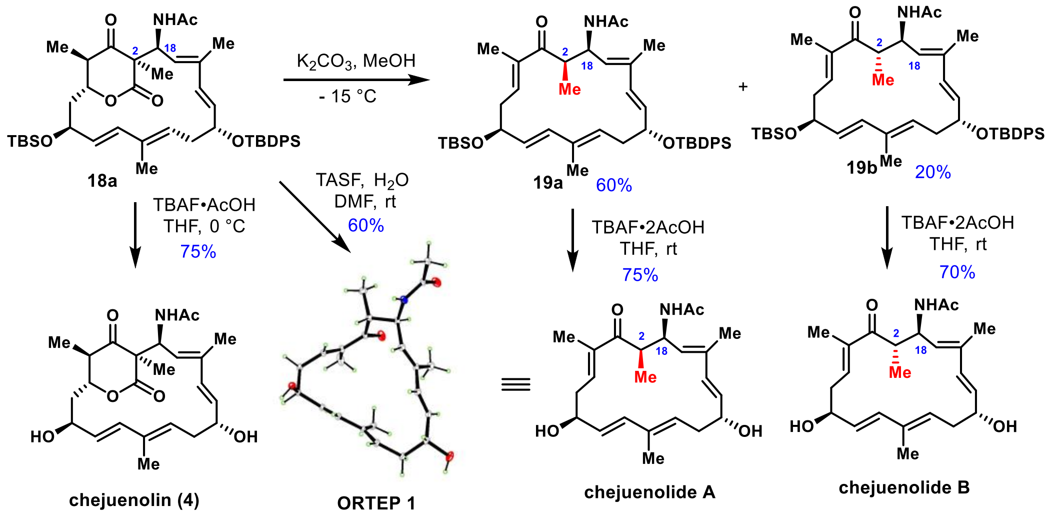

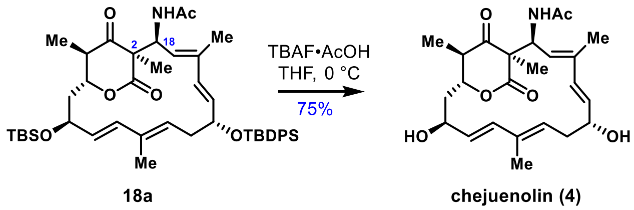

To a solution of the macrocycle compound **18a** (55 mg, 0.07 mmol) in THF (7 mL) was added TBAF•AcOH (2.8 mL, 1 M in THF, 40.0 equiv.) at 0 ℃. The resulting solution was stirred for 6 days at 0 ℃. The mixture was quenched by phosphate buffer (*aq*., pH = 7, 30 mL) and warmed to ambient temperature. The resulting mixture was extracted with ester acetate (30 mL x 3), the combined organic layer was dried over anhydrous Na_2SO_4, filtered and concentrated, the residue was purified by preparative TLC to afford the target compound **chejuenolin (4)** (22 mg, 75% yield) as white foam.

Data for **chejuenolin (4)**: $[\alpha]_D^{25}$ = −16.7 (*c* = 0.20, $CDCl_3$) **^{1}H NMR** (500 MHz, $CDCl_3$) δ 6.87 (d, *J* = 10.0 Hz, 1H), 6.21 (d, *J* = 15.6 Hz, 1H), 5.64 (d, *J* = 15.6 Hz, 1H), 5.52 (dd, *J*

= 15.5, 8.8 Hz, 1H), 5.41 (t, *J* = 10.3 Hz, 1H), 5.36 (dd, *J* = 15.6, 7.7 Hz, 1H), 5.24 (t, *J* = 8.2 Hz, 1H), 4.94 (d, *J* = 10.7 Hz, 1H), 4.58 (td, *J* = 8.4, 4.0 Hz, 1H), 4.58 (td, *J* = 9.2, 6.1 Hz, 1H), 3.67 (td, *J* = 10.0, 2.0 Hz, 1H), 2.78 (qd, *J* = 11.6, 6.5 Hz, 1H), 2.76 – 2.71 (m, 1H), 2.17 (ddd, *J* = 12.4, 10.4, 8.5 Hz, 1H), 2.10 (ddd, *J* = 14.6, 10.8, 4.4 Hz, 1H), 2.00 (s, 3H), 1.89 (ddd, *J* = 11.6, 9.4, 2.2 Hz, 1H), 1.83 (d, *J* = 1.1 Hz, 3H), 1.68 (s, 3H), 1.53 (s, 3H), 1.10 (d, *J* = 6.5 Hz, 3H) ppm; **^{13}C NMR** (125 MHz, $CDCl_3$) δ 210.6, 171.9, 169.5, 139.0, 138.2, 135.8, 134.1, 131.8, 129.9, 126.1, 126.0, 75.4, 74.5, 70.3, 59.1, 52.2, 45.9, 40.3, 35.1, 23.7, 23.3, 13.0, 12.6, 8.9 ppm; **IR** (thin film): υ_{max} = 3475, 2959, 2925, 2861, 1745, 1709, 1659, 1504, 1453, 1374, 1248 cm^{-1}; **HRMS-ESI** (*m/z*): calcd. For $C_{24}H_{33}NO_6Na$ $[M + Na]^+$: 454.2200, found: 454.2203.

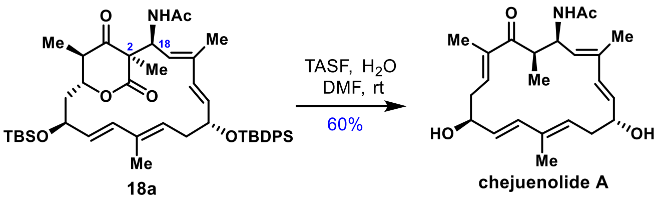

To a solution of macrocycle **18a** (34 mg, 0.04 mmol) in DMF (8 mL) was added TASF (180 mg, 0.65 mmol) and H_2O (32 µL, 1.7 mmol) dissolved in DMF (3 mL). The resulting mixture was stirred for 13 hours at room temperature, diluted with EtOAc (30 mL) and cooled to 0 ℃, during which time a mixture of brine (10 mL) and phosphate buffer (*aq*.,100 mL) was added to quench the reaction. The resulting slurry was further extracted with additional EtOAc (40 mL x 3), the combined organic layer was dried over anhydrous Na_2SO_4, filtered and concentrated, the residue was purified by preparative TLC to afford **chejuenolide A** (10.0 mg, 60% yield) as a white solid.

Data for **chejuenolide A**: $[\alpha]_D^{25}$ = +222.8 (*c* = 0.20, MeOH); **^{1}H NMR** (500 MHz, CD_3OD) δ 6.71 (dd, *J* = 10.1, 5.2 Hz, 1H), 6.05 (d, *J* = 15.5 Hz, 1H), 5.75 (d, *J* = 15.8 Hz, 1H), 5.43 (dd, *J* = 15.6, 5.2 Hz, 1H), 5.36 (dd, *J* = 15.8, 7.7 Hz, 1H), 5.19 (t, *J* = 7.8 Hz, 1H), 5.10 (d, *J* = 10.4 Hz, 1H), 4.99 (t, *J* = 10.3 Hz, 1H), 4.52 (d, *J* = 4.6 Hz, 1H), 4.05 (ddd, *J* = 10.4, 7.8, 4.7 Hz, 1H), 3.42 (dq, *J* = 10.4, 6.9 Hz, 1H), 2.73 (ddd, *J* = 13.7, 10.3, 3.2 Hz, 1H), 2.53 – 2.47 (m, 2H), 2.18 (ddd, *J* = 13.1, 10.3, 9.1 Hz, 1H), 1.94 (s, 3H), 1.72 (s, 3H), 1.69 (d, *J* = 1.2 Hz, 3H), 1.60 (s, 3H), 1.00 (d, *J* = 6.8 Hz, 3H) ppm; **^{13}C NMR** (125 MHz,

CD_3OD) δ 205.7, 172.4, 140.0, 139.2, 136.8, 135.8, 135.4, 135.3, 131.6, 131.3, 129.6, 127.7, 74.8, 71.3, 51.0, 44.9, 37.4, 36.7, 22.7, 16.1, 13.6, 12.8, 12.5 ppm; **IR** (thin film): υ_{max} = 3291, 2932, 2860, 2394, 1659, 1267, 1123, 1029 cm^{-1}; **HRMS-ESI** (*m/z*): calcd. For $C_{23}H_{34}NO_4$ $[M + H]^+$: 388.2482, found: 388.2485.

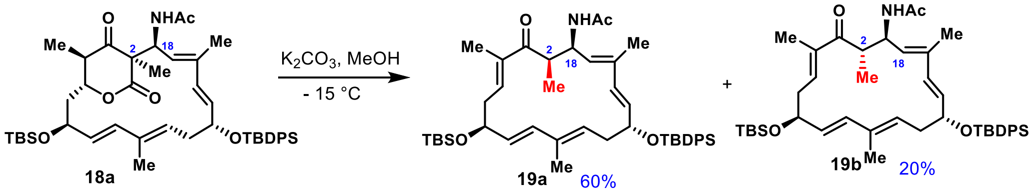

To a solution of **18a** (25 mg, 0.03 mmol) in MeOH (5 mL) was added K_2CO_3 (44 mg, 0.32 mmol, 10 equiv.) at -15 ℃, the resulting mixture was stirred for 8 hours at -15 ℃ before it was quenched by saturated NH_4Cl (*aq*., 20 mL). The mixture was warmed to ambient temperature and was extracted with CH_2Cl_2 (20 mL x 3), the combined organic layer was dried over anhydrous Na_2SO_4, filtered and concentrated, the residue was purified by preparative TLC to give compound **19a** (15 mg, 60% yield) as colorless oil and **19b** (4,5 mg, 20% yield) as colorless oil.

Data for **19a:** $[\alpha]_D^{25}$ = +231.1 (*c* = 0.45, $CHCl_3$); **¹H NMR** (400 MHz, $CDCl_3$) δ 7.68 – 7.62 (m, 4H), 7.43 – 7.32 (m, 6H), 6.43 (dd, *J* = 8.0, 6.0 Hz, 1H), 5.86 (d, *J* = 15.5 Hz, 1H), 5.45 (m, 2H), 5.27 (d, *J* = 6.7 Hz, 1H), 5.22 (dd, *J* = 15.5, 5.4 Hz, 2H), 5.03 – 4.96 (m, 2H), 4.91 (d, *J* = 9.5 Hz, 1H), 4.44 (brs, 1H), 4.13 – 4.05 (m, 1H), 3.22 (dq, *J* = 9.7, 6.8 Hz, 1H), 2.54 (ddd, *J* = 12.5, 9.5, 3.1 Hz, 1H), 2.42 – 2.29 (m, 2H), 2.21 (dt, *J* = 12.9, 9.3 Hz, 1H), 1.98 (s, 3H), 1.70 (s, 3H), 1.69 (s, 3H), 1.32 (s, 3H), 1.04 – 1.3 (m, 11 H), 0.91 (s, 9H), 0.07 (s, 3H), 0.04 (s, 3H) ppm; **¹³C NMR** (100 MHz, $CDCl_3$) δ 203.7, 169.3, 138.7, 136.7, 136.0, 135.9, 135.8, 134.6, 134.5, 134.3, 134.2, 131.1, 129.7, 129.7, 129.2, 128.6, 127.7, 127.6, 127.1, 77.2, 75.4, 71.8, 50.2, 44.9, 37.3, 36.6, 27.1, 26.1, 23.7, 19.3, 18.4, 15.8, 13.1, 12.9, 12.6, -4.4, -4.6 ppm; **IR** (thin film): υ_{max} = 3278, 3070, 3048, 2955, 2929, 2856, 1662,

1551, 1481, 1462, 1389 cm^{-1}; **HRMS-ESI** (*m/z*): calcd. For $C_{45}H_{66}NO_4Si_2$ [M + H]$^+$:740.4525, found: 740.4521.

Data for **19b**: $[\alpha]_D^{25}$ = +18.9 (*c* = 0.2, $CHCl_3$); ^{1}**H NMR** (400 MHz, $CDCl_3$) δ 7.69 – 7.61 (m, 4H), 7.41 – 7.31 (m, 6H), 6.26 (dd, J = 7.6, 5.6 Hz, 1H), 5.95 (d, J = 15.5 Hz, 1H), 5.47 (dd, J = 15.8, 7.9 Hz, 1H), 5.39 (d, J = 15.8 Hz, 1H), 5.28 (dd, J = 16.3, 4.9 Hz, 1H), 5.00 (t, J = 8.1 Hz, 1H), 4.90 (s, 1H), 4.89 (s, 1H), 4.42 – 4.38 (m, 1H), 4.04 (ddd, J = 10.1, 8.0, 5.2 Hz, 1H), 3.25 – 3.23 (m, 1H), 2.61 – 2.53 (m, 1H), 2.48 – 2.23 (m, 3H), 2.01 (s, 3H), 1.79 (s, 3H), 1.74 (s, 3H), 1.37 (s, 3H), 1.08 (d, J = 7.1 Hz, 3H), 1.04 (s, 9H), 0.87 (s, 9H), 0.04 (s, 3H), 0.02 (s, 3H) ppm; ^{13}**C NMR** (100 MHz, $CDCl_3$) δ 203.7, 169.3, 138.7, 136.7, 136.0, 135.9, 135.8, 134.6, 134.5, 134.3, 134.2, 131.1, 129.7, 129.7, 129.2, 128.6, 127.7, 127.6, 127.1, 77.2, 75.4, 71.8, 50.2, 44.9, 37.3, 36.6, 27.1, 26.1, 23.7, 19.3, 18.4, 15.8, 13.1, 12.9, 12.6, -4.4, -4.6 ppm; **IR** (thin film): υ_{max} = 3408, 3070, 3050, 2955, 2929, 2856, 1675, 1656, 1502, 1471, 1462 cm^{-1}; **HRMS-ESI** (*m/z*): calcd. For $C_{45}H_{66}NO_4Si_2$ [M + H]$^+$:740.4525, found: 740.4529.

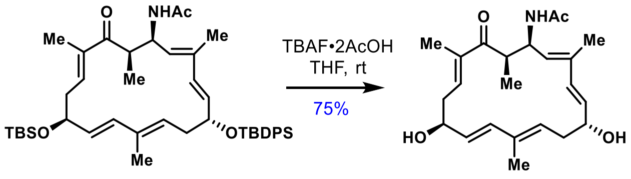

To a solution of **27** (50 mg, 0.068 mmol) in dry THF (2.0 mL) was added TBAF-2AcOH (2.0 mL, 1 M in THF, 30 equiv.), the resulting mixture was stirred for 6 days at room temperature and quenched by saturated NH_4Cl (*aq*., 10 mL). The resulting mixture was extracted with EtOAc (10 mL x 3), the combined organic layer was dried by anhydrous Na_2SO_4, filtered and concentrated, the residue was purified by preparative TLC (CH_2Cl_2/MeOH = 10/1) to afford **chejuenolide A** (19.6 mg, 70% yield) as white solid.

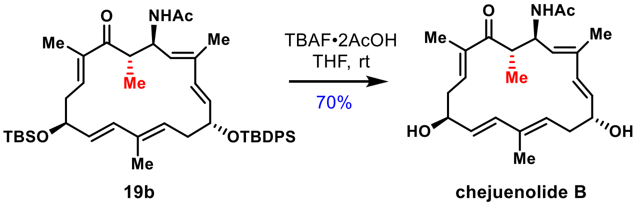

To a solution of **28** (14 mg, 0.02 mmol) in dry THF (1.4 mL) was added TBAF-2AcOH (0.57 mL, 1 M in THF, 30 equiv.), the resulting mixture was stirred for 4 days at room temperature and quenched by phosphate buffer (*aq*., pH = 7, 20 mL). The resulting mixture was extracted with EtOAc (10 mL x 3), the combined organic layer was dried by anhydrous Na_2SO_4, filtered and concentrated, the residue was purified by preparative TLC (CH_2Cl_2/MeOH = 15/1 v/v) to give **chejuenolide B** (5.0 mg, 70% yield) as white solid.

Data for **Chejuenolide B**: $[\alpha]_D^{25}$ = −8.2 (*c* = 0.37, MeOH); ^{1}**H NMR** (400 MHz, CD_3OD) δ 6.48 (dd, *J* = 7.8, 6.4Hz, 1H), 6.14 (d, *J* = 15.7 Hz, 1H), 5.71 (d, *J* = 15.7 Hz, 1H), 5.54 (dd, *J* = 15.7, 5.3 Hz, 1H), 5.43 (dd, *J* = 15.6, 8.5 Hz, 1H), 5.24 (t, *J* = 8.2 Hz, 1H), 5.06 (d, *J* = 9.9 Hz, 1H), 4.91 (dd, *J* = 9.9, 3.5 Hz, 1H), 4.48 (brs, 1H), 4.05 (ddd, *J* = 10.2, 8.9, 5.1 Hz, 1H), 3.42 (qd, *J* = 7.2, 3.7 Hz, 1H), 2.71 – 2.63 (m, 1H), 2.57 – 2.47 (m, 2H), 2.25 (dt, *J* = 12.6, 10.4 Hz, 1H)), 2.00 (s, 3H), 1.81 (brs, 6H), 1.65 (s, 3H), 1.08 (d, *J* = 7.1 Hz, 3H) ppm; ^{13}**C NMR** (100 MHz, CD_3OD) δ 208.9, 172.5, 140.2, 140.2, 136.4, 136.0, 135.9, 135.8, 131.6, 131.3, 128.9, 128.8, 75.1, 71.9, 51.5, 43.1, 37.8, 36.9, 22.9, 16.3, 13.1, 12.5, 12.3 ppm; **IR** (thin film): υ_{max} = 3416, 2982, 2924, 2858, 2814, 2554, 1645, 1621, 1504, 1545 cm^{-1}; **HRMS-ESI** (*m/z*): calcd. For $C_{23}H_{34}NO_4$ $[M + H]^+$: 388.2482, found: 388.2479.

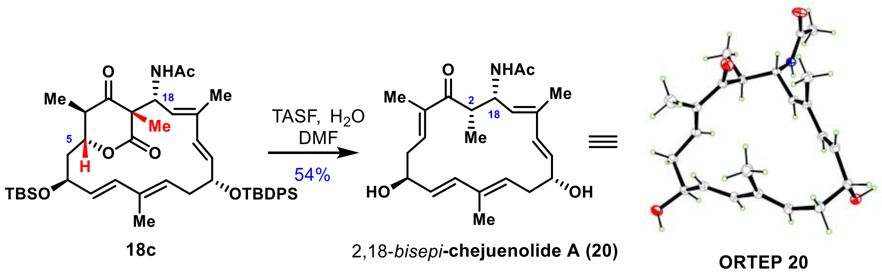

To a solution of macrocycle **18c** (34 mg, 0.04 mmol) in DMF (8 mL) was added TASF (180 mg, 0.65 mmol) and H_2O (32 µL, 1.7 mmol) dissolved in DMF (3 mL). The resulting mixture was stirred for 13 hours at room temperature, diluted with EtOAc (30 mL) and cooled to 0 ℃, during which time a mixture of brine (10 mL) and phosphate buffer (*aq*.,100

mL) was added to quench the reaction. The resulting slurry was further extracted with additional EtOAc (40 mL x 3), the combined organic layer was dried over anhydrous Na_2SO_4, filtered and concentrated, the residue was purified by preparative TLC to give 2,18-*bisepi*-**chejuenolide A** (10 mg, 60% yield) as a white solid.

Data for 2,18-*bisepi*-**chejuenolide A**: $[\alpha]_D^{25}$ = −262.5 (*c* = 0.28, MeOH); **^{1}H NMR** (500 MHz, CD_3OD) δ 6.54 (d, *J* = 9.4, 6.2 Hz, 1H), 6.07 (d, *J* = 15.5 Hz, 1H), 6.02 (d, *J* = 15.9 Hz, 1H), 5.52 (dd, *J* = 15.9, 4.7 Hz, 1H), 5.48 (dd, *J* = 9.9, 5.2 Hz, 1H), 5.35 (dd, *J* = 15.5, 8.3 Hz, 1H), 5.05 (t, *J* = 10.0 Hz, 1H), 5.01 (t, *J* = 10.0 Hz, 1H), 4.52 (d, *J* = 4.1 Hz, 1H), 4.26 (td, *J* = 8.8, 5.4 Hz, 1H), 3.40 (dq, *J* = 9.8, 6.8 Hz, 1H), 2.65 – 2.55 (m, 2H), 2.46 – 2.33 (m, 2H), 1.94 (s, 3H), 1.72 (s, 3H), 1.65 (d, *J* = 0.7 Hz, 3H), 1.53 (s, 3H), 0.99 (d, *J* = 6.9 Hz, 3H) ppm; **^{13}C NMR** (125 MHz, CD_3OD) δ 205.4, 172.3, 139.7, 139.0, 138.1, 135.9, 135.7, 135.5, 130.6, 130.3, 129.1, 128.7, 73.6, 70.5, 50.9, 49.0, 44.8, 38.5, 35.1, 22.8, 16.4, 13.2, 12.9, 12.6 ppm; **IR** (thin film): υ_{max} = 3365, 2960, 2922, 2852, 1658, 1650, 1637, 1547, 1454, 1373, 1286 cm^{-1}; **HRMS-ESI** (*m/z*): calcd. For $C_{23}H_{34}NO_4$ $[M + H]^+$: 388.2482, found: 388.2478.

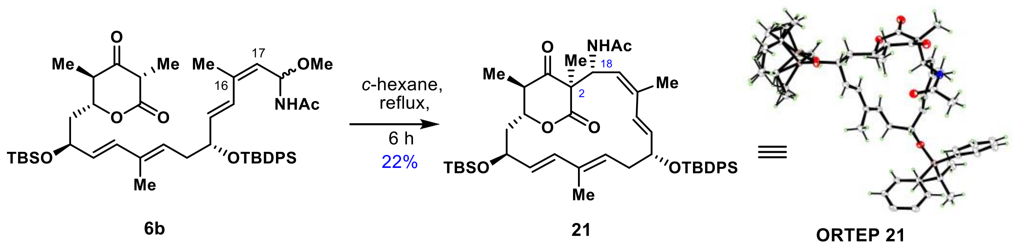

A solution of 1:1 diastereomeric mixture of *N*,*O*-acetal **6b** (850 mg, 1.0 mmol) in toluene (10 mL) was added to a refluxing cyclohexane (1000 mL) by cannula transfer. The reaction mixture was stirred vigorously at reflux for 6 hours until the complete disappearance of **6b** (monitored by TLC). The solution was cooled to ambient temperature and concentrated at vacuum to give a foam residue, which was purified by preparative TLC (petroleum ether / EtOAc = 2/1) to afford the macrocycle **21** (180 mg, 22% yield) as a white foam.

Data for **21**: $[\alpha]_D^{25}$ = +9.2 (*c* = 0.69, $CDCl_3$); **^{1}H NMR** (400 MHz, $CDCl_3$) δ 7.70 – 7.57 (m, 4H), 7.43 – 7.29 (m, 6H), 6.07 (d, *J* = 15.6 Hz, 1H), 5.70 (dd, *J* = 15.7, 8.3 Hz, 1H), 5.54

(d, J = 15.6 Hz, 1H), 5.45 (d, J = 7.5 Hz, 1H), 5.38 (d, J = 9.1 Hz, 1H), 5.30 (dd, J = 15.6, 8.9 Hz, 1H), 5.10 (t, J = 8.2 Hz, 1H), 5.02 (t, J = 8.2 Hz, 1H), 4.82 (td, J = 10.9, 2.7 Hz, 1H), 4.46 (td, J = 9.4, 5.1 Hz, 1H), 4.00 (ddd, J = 10.1, 8.3, 5.8Hz, 1H), 2.48 (ddd, J = 12.5, 9.4, 5.8Hz, 1H), 2.18 (qd, J = 11.1, 6.8 Hz, 1H), 2.06 (qd, J = 12.1, 7.3 Hz, 1H), 1.97 – 1.83 (m, 1H), 1.78 (s, 3H), 1.76 (s, 3H), 1.37 (s, 3H), 1.29 (3, 1H), 1.12 (d, J = 6.8 Hz, 3H), 1.03 (s, 9H), 0.84 (s, 9H), 0.02 (s, 3H), -0.01 (s, 3H) ppm; **^{13}C NMR** (100 MHz, $CDCl_3$) δ 204.9, 170.9, 168.6, 139.3, 137.2, 136.0, 135.9, 135.8, 134.8, 134.3, 134.1, 129.8, 129.7, 129.0, 128.3, 127.7, 127.6, 126.3, 120.9, 76.1, 75.7, 71.7, 59.6, 47.6, 46.6, 42.0, 35.7, 27.1, 26.0, 22.9, 20.2, 19.4, 18.3, 13.7, 11.8, 10.9, -3.9, -4.5 ppm; **IR** (thin film): υ_{max} = 3353, 3073, 2961, 2929, 2857, 1743, 1712, 1680, 1528, 1293, 1259 cm^{-1}; **HRMS-ESI** (*m/z*): calcd. For $C_{46}H_{65}O_6NNaSi_2$ $[M + Na]^+$: 806.4243, found: 806.4251.

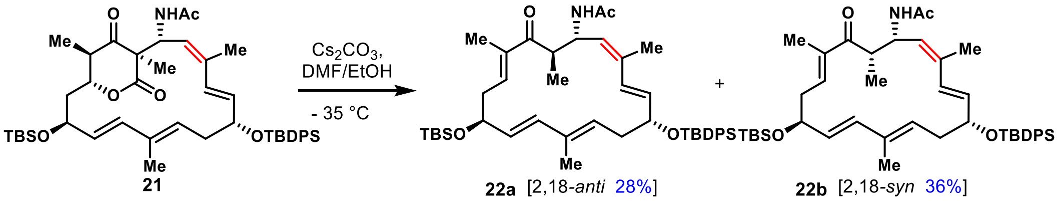

To a solution of macrocycle **21** (100 mg, 0.13 mmol) in DMF/EtOH (20 ml/5 mL) was added Cs_2CO_3 (458 mg, 1.28 mmol) at – 35 ℃, the resulting mixture was stirred for 4 hours at – 35 ℃. The reaction was quenched by saturated NH_4Cl (*aq*., 30 mL), diluted by H_2O (100 mL), the resulting mixture was extracted with ether/petroleum ether (25 mL/25 mL x 3), the combined organic layer was dried over anhydrous Na_2SO_4, filtered and concentrated, the residue was purified by preparative TLC (ether/petroleum ether = 1/1 v/v) to give **22a** (26 mg, 28% yield) as a white foam, **22b** (34 mg, 36% yield) as a white foam, respectively.

Data for **22a**: $[\alpha]_D^{25}$ = +242.4 (*c* = 0.90, $CHCl_3$); **^{1}H NMR** (400 MHz, $CDCl_3$) δ 7.72 – 7.64 (m, 4H), 7.47 – 7.39 (m, 6H), 7.06 (d, J = 9.4 Hz, 1H), 6.58 (t, J = 5.1 Hz, 1H), 6.25 (d, J = 15.3 Hz, 1H), 5.87 (d, J = 15.5 Hz, 1H), 5.51 (dd, J = 15.5, 8.7 Hz, 1H), 5.30 (dd, J = 15.5, 3.4 Hz, 1H), 5.13 (dd, J = 10.4, 5.9 Hz, 1H), 5.08 (d, J = 8.4 Hz, 1H), 4.88 (td, J = 8.9, 3.3 Hz, 1H), 4.56 (brs, 1H), 4.13 (td, J = 10.3, 4.2 Hz, 1H), 3.16 (qfd, J = 7.2, 3.8 Hz, 1H), 2.53 – 2.32 (m, 4H), 2.01 (s, 3H), 1.64 (s, 2H), 1.61 (s, 3H), 1.53 (s, 3H), 1.11 (d, J =

7.3 Hz, 3H), 1.06 (s, 9H), 0.88 (s, 9H), 0.03 (s, 3H), 0.03 (s, 3H) ppm; **^{13}C NMR** (100 MHz, $CDCl_3$) δ 203.4, 169.1, 138.3, 137.2, 136.0, 136.0, 135.6, 134.9, 134.6, 134.2, 134.1, 134.0, 129.7, 129.3, 128.2, 127.7, 127.7, 127.2, 126.7, 75.5, 74.1, 46.8, 41.3, 38.2, 36.4, 27.1, 26.0, 23.5, 20.7, 19.4, 18.3, 12.5, 12.1, 11.3, -4.1, -4.6 ppm; **IR** (thin film): υ_{max} = 3424, 3078, 3042, 2955, 2930, 2891, 2857, 1658, 1503, 1472, 1111 cm^{-1}; **HRMS-ESI** (*m/z*): calcd. For $C_{45}H_{65}NO_4NaSi_2$ $[M + Na]^+$: 762.4344, found: 762.4347.

Data for **22b**: $[\alpha]_D^{25}$ = −41.8 (*c* = 0.80, $CHCl_3$); **^{1}H NMR** (400 MHz, $CDCl_3$) δ 7.72 – 7.61 (m, 4H), 7.38 (m, 6H), 6.16 (dd, *J* = 6.5, 4.7Hz, 1H), 6.04 (d, *J* = 5.6 Hz, 1H), 6.00 (d, *J* = 5.6 Hz, 1H), 5.81 (d, *J* = 7.2 Hz, 1H), 5.64 (dd, *J* = 15.7, 8.2 Hz, 1H), 5.32 (dd, *J* = 15.6, 8.0 Hz 1H), 5.27 (d, *J* = 8.9 Hz, 1H), 5.11 (t, *J* = 8.1 Hz, 1H), 4.79 (td, *J* = 10.1, 3.2 Hz, 1H), 4.21 (td, *J* = 12.0, 4.2 Hz 1H), 4.09 (dd, *J* = 10.4, 4.9 Hz, 1H), 3.20 (qd, *J* = 6.9, 3.2 Hz, 1H), 2.54 (td, *J* = 17.1, 9.6 Hz, 1H), 2.49 – 2.37 (m, 2H), 2.13 (td, *J* = 11.0, 7.9 Hz, 1H), 1.86 (s, 3H), 1.79 (s, 3H), 1.71 (s, 3H), 1.27 (s, 3H), 1.08 (d, *J* = 6.9.0 Hz, 3H), 1.05 (s, 9H), 0.86 (s, 9H), 0.03 (s, 3H), 0.00 (s, 3H) ppm; **^{13}C NMR** (100 MHz, $CDCl_3$) δ 206.6, 169.6, 141.4, 136.9, 136.1, 136.0, 134.7, 134.4, 134.3, 133.9, 133.5, 131.9, 130.0, 130.0, 129.9, 128.3, 127.8, 127.7, 127.3, 126.1, 76.2, 69.0, 49.3, 43.6, 37.4, 36.2, 27.1, 25.9, 23.7, 20.2, 19.3, 18.3, 17.1, 12.8, 11.3, -4.7, -4.8 ppm; **IR** (thin film): υ_{max} = 3300, 3076, 3044, 2955, 2929, 2890, 2857, 2247, 1660, 1472, 1427, 1112, 1066 cm^{-1}; **HRMS-ESI** (*m/z*): calcd. For $C_{45}H_{65}NO_4NaSi_2$ $[M + Na]^+$: 762.4344, found: 762.4347.

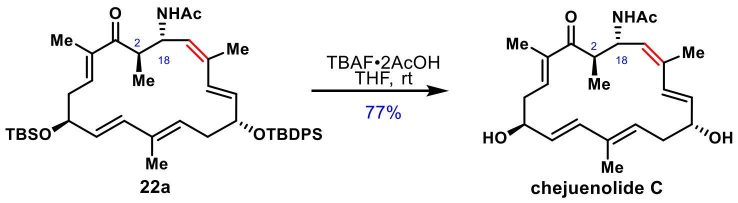

To a solution of **22a** (26 mg, 0.035 mmol) in dry THF (3 mL) was added TBAF-2AcOH (0.7 mL, 1 M in THF, 20 equiv.), the resulting mixture was stirred for 5 days at room temperature and quenched by saturated NH_4Cl (*aq*., 20 mL). The resulting mixture was extracted with EtOAc (20 mL x 3), the combined organic layer was dried by anhydrous Na_2SO_4, filtered and concentrated, the residue was purified by preparative TLC (CH_2Cl_2/MeOH = 10/1) to give **chejuenolide C** (10.5 mg, 77% yield) as a white solid.

Data for **chejuenolide C**: $[\alpha]_D^{25}$ = +156.8 (c = 0.2, MeOH); **^{1}H NMR** (500 MHz, CD_3OD) δ 6.85 (t, J = 6.5 Hz, 1H), 6.30 (d, J = 15.7 Hz, 1H), 6.20(d, J = 15.5 Hz, 1H), 5.55 (dd, J = 15.8, 8.6 Hz, 1H), 5.52 (dd, J = 15.7, 4.8 Hz, 1H), 5.36 (dd, J = 9.3, 7.1 Hz, 1H), 5.22 (d, J = 8.0 Hz, 1H), 4.94 (td, J = 10.5, 5.1 Hz, 1H), 4.48 (brs, 1H), 4.11 (ddd, J = 10.9, 8.6, 4.4 Hz, 1H), 3.47 (qd, J = 7.0, 4.1 Hz, 1H), 2.63 (ddd, J = 16.1, 7.9, 3.0 Hz, 1H), 2.57 – 2.47 (m, 2H), 2.32 (dt, J = 12.7, 10.5 Hz, 1H), 1.98 (s, 3H), 1.73 (s, 3H), 1.71 (s, 3H), 1.67 (s, 3H), 1.12 (d, J = 7.1 Hz, 3H) ppm; **^{13}C NMR** (125 MHz, CD_3OD) δ 205.8, 172.4, 141.7, 138.6, 136.3, 135.7, 135.0, 134.5, 129.6, 128.5, 128.4, 128.2, 75.1, 70.4, 50.3, 44.4, 37.0, 36.7, 22.7, 20.1, 14.7, 12.9, 11.4 ppm; **IR** (thin film): υ_{max} = 3362, 2978, 2925, 2854, 1724, 1651, 1519, 1454, 1377, 1272, 1030, 963 cm^{-1}; **HRMS-ESI** (m/z): calcd. For $C_{23}H_{33}O_4NNa$ $[M + Na]^+$: 410.2302, found: 410.2302.

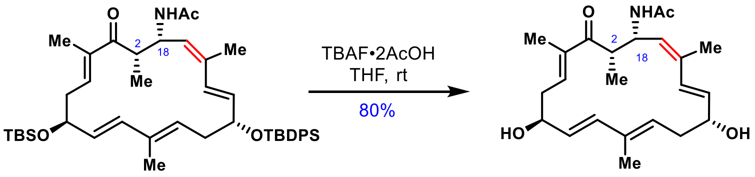

To a solution of **22b** (34 mg, 0.046 mmol) in dry THF (3 mL) was added TBAF-2AcOH (1 mL, 1 M in THF, 22 equiv.), the resulting mixture was stirred for 5 days at room temperature and quenched by saturated NH_4Cl (*aq*., 20 mL). The resulting mixture was extracted with EtOAc (20 mL x 3), the combined organic layer was dried by anhydrous Na_2SO_4, filtered and concentrated, the residue was purified by preparative TLC (CH_2Cl_2/MeOH = 10/1 v/v) to give 2-*epi*-**chejuenolide C** (**23**) (14 mg, 80% yield) as white solid.

Data for 2-*epi*-**chejuenolide C** (**23**): $[\alpha]_D^{25}$ = −45.2 (c = 0.35, MeOH); **^{1}H NMR** (500 MHz, CD_3OD) δ 6.37 – 6.35 (m, 1H), 6.23 (d, J = 15.7 Hz, 1H), 6.14 (d, J = 15.7 Hz, 1H), 5.57 (d, J = 7.8 Hz, 1H), 5.54 (d, J = 7.8 Hz, 1H), 5.47 (d, J = 8.4 Hz, 1H), 5.30 (t, J = 8.1 Hz, 1H), 5.01 (dd, J = 8.5, 2.6 Hz, 1H), 4.33 (ddd, J = 10.9, 7.6, 4.2 Hz 1H), 4.02 (ddd, J = 10.8, 8.4, 4.9 Hz 1H), 3.36 (qd, J = 6.8, 2.8 Hz, 1H), 2.70 – 2.58 (m, 2H), 2.57 – 2.50 (m, 1H), 2.16 (ddd, J = 12.4, 11.1, 8.7 Hz, 1H), 1.83 (s, 3H), 1.82 (d, J = 0.7 Hz,

3H), 1.75 (s, 3H), 1.72 (d, J = 0.8 Hz, 3H), 1.09 (d, J = 6.8 Hz, 3H) ppm; **^{13}C NMR** (125 MHz, CD_3OD) δ 204.7, 171.5, 139.4, 137.9, 137.5, 136.2, 136.1, 134.4, 129.4, 129.3, 128.8, 127.9, 75.0, 73.1, 47.7, 43.4, 37.6, 36.6, 22.4, 20.1, 12.8, 12.7, 10.2 ppm; **IR** (thin film): υ_{max} = 3358, 2923, 2853, 1652, 1534, 1451, 1385, 1292, 1113, 1029, 963 cm^{-1}; **HRMS-ESI** (*m/z*): calcd. For $C_{23}H_{33}O_4NNa$ $[M + Na]^+$: 410.2302, found: 410.2304.

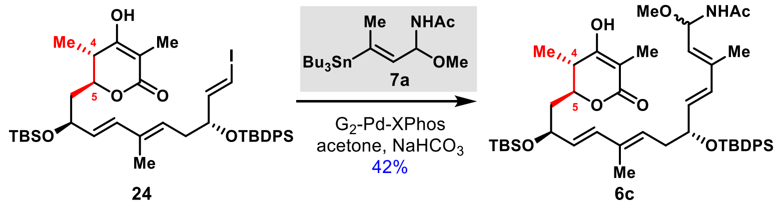

To a solution of **24** (1.1 g, 1.37 mmol) and *N,O*-**acetal** (**7a**) (893 mg, 2.06 mmol) in acetone (100 mL) under argon atmosphere was added $NaHCO_3$ (231 mg, 2.75 mmol) and Pd-G_2-XPhos (432 mg, 0.55 mmol), the resulting mixture was stirred for 2 days at room temperature and quenched by saturated KF (*aq*., 30 mL), and brine (50 mL), diluted with H_2O (100 mL), the resulting mixture was extracted by ether (50 mL x 3), the combined organic layer was dried over anhydrous Na_2SO_4, filtered and concentrated, the residue was purified by column chromatography on silica (petroleum ether/acetone = 5/1 to 3/1) to afford the target compound **6c** (470 mg, 42%) as a yellow foam, which was unstable, submitted to next step directly.

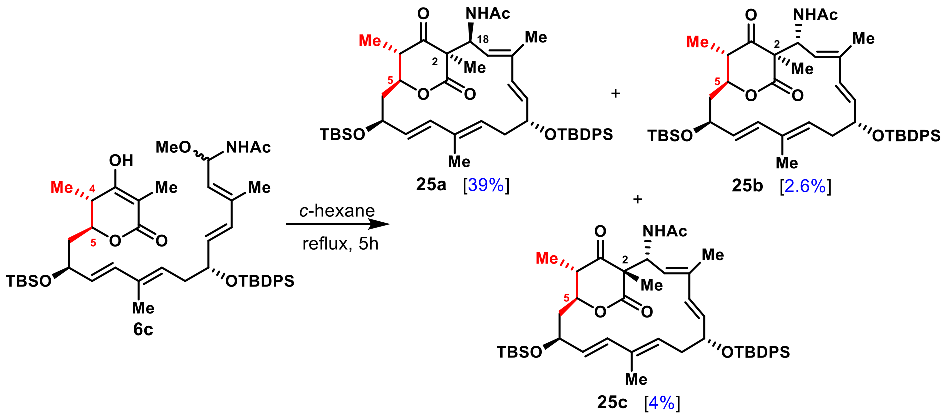

A solution of 1:1 diastereomeric mixture of *N*,*O*-acetal **6c** (470 mg, 0.58 mmol) in toluene (5 mL) was added to a refluxing cyclohexane (1000 mL) by cannula transfer. The reaction

mixture was stirred vigorously at reflux for 6 hours until the complete disappearance of starting material (monitored by TLC). The solution was cooled to ambient temperature and concentrated at vacuum to give a foam residue, which was purified by PTLC (petroleum ether/ethyl acetate = 2/1) to give the macrocycles **25a** (175 mg, 39% yield) as a yellow foam, **25b** (12 mg, 2.6% yield) as a yellow foam, **25c** (18 mg, 4% yield) as a yellow foam, respectively.

Data for **25a**: $[\alpha]_D^{25}$ = +240.0 (*c* = 0.33, $CHCl_3$); **^{1}H NMR** (500 MHz, $CDCl_3$) δ 7.69 – 7.61 (m, 4H), 7.44 – 7.31 (m, 6H), 6.63 (d, *J* = 10.0 Hz, 1H), 6.04 (dd, *J* = 15.5, 2.1 Hz, 1H), 5.77 (dd, *J* = 15.5, 2.8 Hz, 1H), 5.56 (d, *J* = 15.5, 1H), 5.55 (dd, *J* = 18.9, 2.8 Hz, 1H), 5.33 (d, *J* = 15.5 Hz, 1H), 4.96 (dd, *J* = 11.0, 5.8 Hz, 1H), 4.63 (brs, 1H), 4.60 (d, *J* = 11.2 Hz, 1H), 4.37 (dt, *J* = 11.8, 3.2 Hz, 1H), 4.03 (ddd, *J* = 11.9, 8.2, 4.1 Hz, 1H), 2.72 (dq, *J* = 13.2, 6.6 Hz, 1H), 2.44 (dt, *J* = 12.5, 11.0 Hz,1H), 2.35 (ddd, *J* = 16.0, 4.4, 3.4 Hz, 1H), 2.25 (dt, *J* = 13.0, 4.4 Hz, 1H), 2.10 (dt, *J* = 16.0, 3.1 Hz, 1H), 2.01 (s, 3H), 1.85 (d, *J* = 1.1 Hz, 3H), 1.48 (s, 1H), 1.36 (s, 3H), 1.25 (d, *J* = 6.6 Hz, 3H), 1.05 (s, 9H), 0.92 (s, 9H), 0.06 (s, 3H) , 0.04 (s, 3H) ppm; **^{13}C NMR** (125 MHz, $CDCl_3$) δ 213.2, 170.1, 169.3, 138.7, 136.3, 135.9, 135.8, 134.4, 134.1, 133.6, 131.7, 131.3, 129.5, 128.3, 127.5, 127.4, 125.9, 124.8, 77.7, 76.1, 69.9, 57.1, 51.1, 46.7, 37.7, 37.3, 29.7, 27.0, 26.1, 23.6, 20.6, 19.2, 18.6, 12.9, 12.7, 11.5, -5.0, -5.1 ppm; **IR** (thin film): υ_{max} = 3428, 3074, 3052, 2968, 2929, 2857, 1753, 1705, 1677, 1500, 1373 cm^{-1}; **HRMS-ESI** (*m/z*): calcd. For $C_{46}H_{66}NO_6Si_2$ $[M + H]^+$: 784.4423, found: 784.4436.

Data for **25b**: $[\alpha]_D^{25}$ = −64.6 (*c* = 0.45, $CHCl_3$) **^{1}H NMR** (400 MHz, $CDCl_3$) δ 7.70 – 7.59 (m, 4H), 7.43 – 7.32 (m, 6H), 6.82 (d, J = 10.0 Hz, 1H), 6.23 (d, J = 15.2 Hz, 1H), 6.20 (d, J = 15.2 Hz, 1H), 5.61 (dd, J = 4.6, 4.0 Hz, 1H), 5.51 (dd, J = 15.6, 8.9 Hz, 1H), 5.40 – 5.34 (m, 2H), 5.19 (d, J = 10.0 Hz, 1H), 4.59 (td, J = 9.5, 3.8 Hz, 1H), 4.54 (d, J = 3.8 Hz, 1H), 4.31(dd, J = 12.2, 4.6 Hz, 1H), 2.48 (dq, J = 12.2, 6.5 Hz, 1H), 2.36 (ddd, J = 15.0, 4.8, 4.8 Hz, 1H), 2.17 – 2.13 (m, 2H), 2.06 (s, 2H), 2.01 (dd, J = 11.2, 3.8 Hz, 1H), 1.54 (s, 3H), 1.49 (s, 3H), 1.39 (s, 3H), 1.11 (s, 9H), 1.04 (d, J = 6.5 Hz, 3H), 0.89 (s, 9H), 0.11 (s, 3H), 0.05 (s, 3H) ppm; **^{13}C NMR** (100 MHz, $CDCl_3$) δ 206.5, 174.5, 169.7, 137.1, 137.0, 136.0, 134.5, 134.2, 133.6, 133.5, 131.0, 130.3, 129.9, 129.8, 128.3, 127.8, 127.7, 125.4, 77.1, 70.7, 70.2, 58.3, 51.2, 46.0, 37.5, 35.1, 27.2, 26.0, 23.8, 21.0, 19.5, 18.3, 13.6, 12.6,

9.9, -3.9, -4.5 ppm; **IR** (thin film): υ_{max} = 3429, 3070, 2927, 2856, 1744, 1714, 1681, 1500, 1427, 1374, 1317 cm^{-1}; **HRMS-ESI** (*m/z*): calcd. For $C_{46}H_{66}NO_6Si_2$ $[M + H]^+$: 784.4423, found: 784.4434.

Data for **25c**: $[\alpha]_D^{25}$ = −252.8 (*c* = 0.25, $CHCl_3$); **^{1}H NMR** (400 MHz, $CDCl_3$) δ 7.74 – 7.57 (m, 4H), 7.47 – 7.31 (m, 6H), 6.85 (d, *J* = 10.1 Hz, 1H), 6.12 (d, *J* = 15.6 Hz, 1H), 6.04 (d, *J* = 15.5 Hz, 1H), 5.59 (dd, *J* = 10.9, 5.0 Hz, 1H), 5.48 (dd, *J* = 15.6, 3.8 Hz, 1H), 5.46 (t, *J* = 8.6 Hz, 1H), 5.43 (dd, *J* = 15.6, 2.7 Hz, 1H), 4.97 (d, *J* = 10.7 Hz, 1H), 4.62 (brs, 1H), 4.03 (td, J = 10.0, 5.1 Hz, 1H), 3.50 (ddd, *J* = 11.8, 5.0, 3.0 Hz, 1H), 2.80 (qd, *J* = 12.0, 6.5 Hz, 1H), 2.25 (ddd, *J* = 14.7, 5.5, 2.9 Hz, 1H), 2.17 – 2.06 (m, 2H), 2.03 (s, 3H), 1.96 (ddd, *J* = 15.0, 10.2, 4.7 Hz, 1H), 1.74 (s, 3H), 1.52 (s, 3H), 1.49 (d, *J* = 6.2 Hz, 3H), 1.20 (t, *J* = 6.1 Hz, 3H), 1.12 (s, 9H), 0.87 (s, 9H), 0.05 (s, 3H), 0.02 (s, 3H) ppm; **^{13}C NMR** (100 MHz, $CDCl_3$) δ 210.7, 171.7, 169.5, 138.7, 137.3, 136.0, 135.9, 135.5, 134.3, 133.6, 133.2, 131.5, 129.9, 129.8, 128.1, 127.9, 127.8, 127.7, 124.1, 75.8, 74.5, 71.7, 59.1, 52.2, 45.5, 42.7, 34.4, 27.3, 26.0, 23.8, 23.5, 19.6, 18.2, 13.1, 12.7, 9.0, -3.8, -4.5 ppm; **IR** (thin film): υ_{max} = 3432, 3077, 3051, 2958, 2927, 2861, 1750, 1711, 1674, 1499, 1462, 1376 cm^{-1}; **HRMS-ESI** (*m/z*): calcd. For $C_{46}H_{66}NO_6Si_2$ $[M + H]^+$: 784.4423, found: 784.4436.

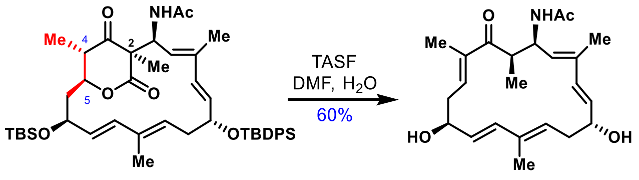

To a solution of macrocycle **25a** (34 mg, 0.04 mmol) in DMF (8 mL) was added TASF (180 mg, 0.65 mmol) and H_2O (32 µL, 1.7 mmol) dissolved in DMF (3 mL). The resulting mixture was stirred for 13 hours at room temperature, diluted with EtOAc (30 mL) and cooled to 0 ℃, during which time a mixture of brine (10 mL) and phosphate buffer (*aq.*,100 mL) was added to quench the reaction. The resulting slurry was further extracted with additional EtOAc (40 mL x 3), the combined organic layer was dried over anhydrous Na_2SO_4, filtered and concentrated, the residue was purified by preparative TLC (CH_2Cl_2/MeOH = 10/1) to afford **chejuenolide A** (**1**) (10 mg, 60% yield) as a white solid.

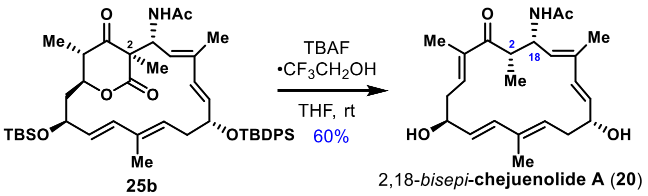

25b

2,18-*bisepi*-**chejuenolide A** (**20**)

To a solution of macrocycle **25b** (8 mg, 0.01 mmol) in THF (3 mL) was added TBAF-CF_3CH_2OH (0.6 mL, 1 M in THF) at room temperature and the resulting mixture was stirred for 2 days at room temperature. The reaction was quenched by phosphate buffer (*aq*., pH = 7, 5 mL), diluted with H_2O (5 mL), the resulting mixture was extracted with EtOAc (10 mL x 3), the combined organic layer was dried over anhydrous Na_2SO_4, filtered and concentrated, the residue was purified by preparative TLC (CH_2Cl_2/MeOH = 10/1) to afford 2,18-*bisepi*-**chejuenolide A** (**20**) (1.5 mg, 60% yield) as a white solid.

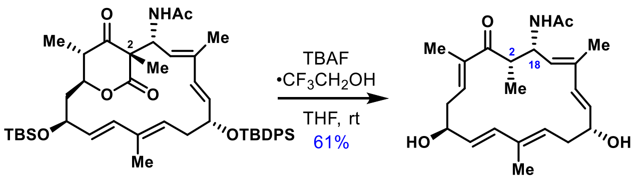

25c

2,18-*bisepi*-**chejuenolide A** (**20**)

To a solution of macrocycle **25c** (5 mg, 0.0064 mmol) in THF (2 mL) was added TBAF-CF_3CH_2OH (0.4 mL, 1 M in THF) at room temperature and the resulting mixture was stirred for 2 days at room temperature. The reaction was quenched by phosphate buffer (*aq*., pH = 7, 5 mL), diluted with H_2O (20 mL), the resulting mixture was extracted with EtOAc (10 mL x 4), the combined organic layer was dried over anhydrous Na_2SO_4, filtered and concentrated, the residue was purified by preparative TLC (CH_2Cl_2/MeOH = 9/1) to afford 2,18-*bisepi*-**chejuenolide A** (**20**) (1.5 mg, 61% yield) as a white solid.

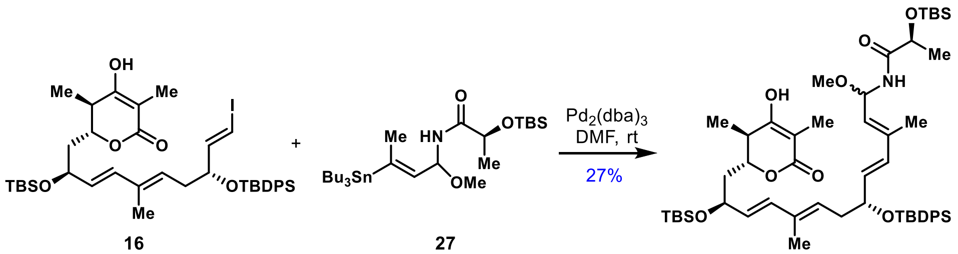

16 27 28

To a solution of **16** (160 mg, 0.2 mmol) and *N,O*-**acetal** (**27**) (231mg, 0.4 mmol) in DMF (8 mL) under argon atmosphere was added $Pd_2(dba)_3$ (73mg, 0.08 mmol), the resulting mixture was stirred for 2 days at room temperature and quenched by brine (100 mL), the resulting mixture was extracted by ether (100 mL x 3), the combined organic layer was washed with brine (50 mL), dried over anhydrous Na_2SO_4, filtered and concentrated, the residue was purified by column chromatography on silica (petroleum ether/acetone = 10/1 v/v) to give the target compound **28** (52 mg, 27% yield) as yellow foam, which was unstable, submitted to next step directly.

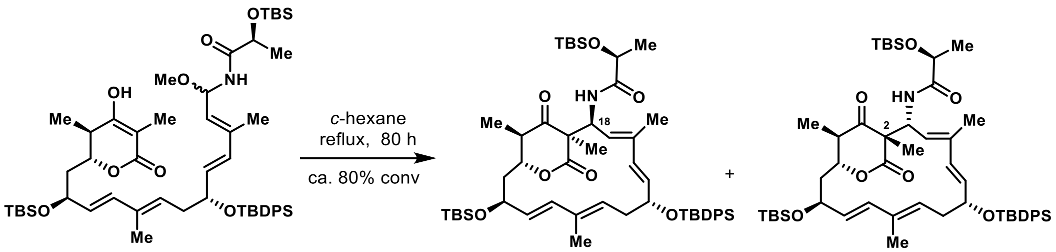

28 **29a** [22%] **29b** [11%]

A solution of 1:1 diastereomeric mixture of *N,O*-acetal **28** (50 mg, 0.05 mmol) in cyclohexane (5 mL) was added to a refluxing cyclohexane (100 mL) by cannula transfer. The reaction mixture was stirred vigorously at reflux for 80 hours (ca. 80% conversion). The solution was cooled to ambient temperature and concentrated at vacuum to give a foam residue, which was purified by PTLC (petroleum ether/acetone = 4/1) to give the macrocycles **29a** (21 mg, 22%) as a foam solid, **29b** (12 mg, 11% yield) as a yellow foam, respectively.

Data for **29a**: $[\alpha]_D^{25}$ = +33.3 (*c* = 0.25, $CHCl_3$); **^{1}H NMR** (400 MHz, $CDCl_3$) δ 7.96 (d, *J* = 10.2 Hz, 1H), 7.69 – 7.61 (m, 4H), 7.44 – 7.30 (m, 6H), 6.02 (d, *J* = 15.5 Hz, 1H), 5.55 (dd, *J* = 15.6, 8.7 Hz, 1H), 5.32 (t, *J* = 10.5f Hz, 1H), 5.28 (d, *J* = 15.5 Hz, 1H), 5.17 (dd, *J* = 15.5, 7.3 Hz, 1H), 5.05 (t, *J* = 8.4 Hz, 1H), 4.83 (d, *J* = 10.4 Hz, 1H), 4.44 (td, *J* = 7.8, 3.8

Hz, 1H), 4.26 (q, J = 6.6 Hz, 1H), 3.98 (td, J = 9.5, 6.4 Hz, 1H), 3.60 (t, J = 10.0 Hz, 1H), 2.68 (qd, J = 11.7, 6.4 Hz, 1H), 2.45 – 2.38 (m, 1H), 2.22 – 2.14 (m, 1H), 1.95 (ddd, J = 14.5, 10.4, 4.2 Hz, 1H), 1.84 – 1.78 (m, 1H), 1.76 (s, 3H), 1.46 (s, 3H), 1.38 (d, J = 6.7 Hz, 3H), 1.27 (s, 3H), 1.04 (s, 9H), 1.03 (d, J = 11.7 Hz, 3H), 0.96 (s, 9H), 0.84 (s, 9H), 0.12 (s, 3H), 0.09 (s, 3H), 0.01 (s, 3H), -0.02 (s, 3H) ppm; **^{13}C NMR** (100 MHz, $CDCl_3$) δ 209.2, 173.8, 172.3, 139.1, 137.0, 136.1, 136.0, 134.6, 134.4, 134.0, 133.9, 132.1, 129.8, 129.7, 129.4, 127.7, 127.6, 126.7, 125.4, 75.8, 75.4, 71.1, 70.0, 59.2, 51.7, 45.7, 41.9, 36.2, 27.1, 26.0, 25.9, 23.1, 22.0, 19.3, 18.3, 18.2, 12.9, 12.2, 8.9, -4.2, -4.7, -4.9 ppm; **IR** (thin film): υ_{max} = 3419, 3072, 2963, 2928, 2857, 1751, 1713, 1681, 1498, 1428 cm^{-1}; **HRMS-ESI** (*m/z*): calcd. For $C_{53}H_{82}O_7NSi_3$ $[M + H]^+$: 928.5394, found: 928.5397.

Data for **29b**: $[\alpha]_D^{25}$ = −122.1 (c = 0.67, $CDCl_3$); **^{1}H NMR** (400 MHz, $CDCl_3$) δ 7.84 (d, J = 10.3 Hz, 1H), 7.72 – 7.59 (m, 4H), 7.46 – 7.30 (m, 6H), 6.09 (d, J = 12.3 Hz, 1H), 6.05 (d, J = 12.2 Hz, 1H), 5.66 (dd, J = 15.5, 9.4 Hz, 1H), 5.55 (t, J = 4.1 Hz, 1H), 5.53 (t, J = 10.5 Hz, 1H), 5.46 (dd, J = 15.5, 3.8 Hz, 1H), 4.67 (d, J = 10.8 Hz, 1H), 4.59 (brs, 1H), 4.38 – 4.31 (m, 2H), 4.20 (q, J = 6.7 Hz, 1H), 2.53 (qd, J = 12.0, 6.7 Hz, 1H), 2.33 – 2.26 (m, 1H), 2.18 – 2.13 (m, 3H), 1.85 (s, 3H), 1.44 (s, 3H), 1.41 (d, J = 6.9 Hz, 3H), 1.40 (s, 3H), 1.31 (d, J = 6.7 Hz, 3H), 1.08 (s, 9H), 0.93 (s, 9H), 0.86 (s, 9H), 0.09 (s, 3H), 0.07 (s, 3H), 0.07 (s, 3H), 0.02 (s, 3H) ppm; **^{13}C NMR** (100 MHz, $CDCl_3$) δ 211.2, 174.3, 170.4, 139.1, 137.3, 136.5, 136.0, 135.9, 134.5, 134.2, 133.5, 130.3, 129.8, 129.8, 129.5, 127.7, 127.6, 127.3, 123.9, 75.8, 71.7, 71.0, 70.4, 57.2, 51.1, 46.3, 38.6, 35.0, 27.2, 26.0, 25.8, 22.4, 21.0, 19.5, 18.2, 18.2, 13.3, 12.6, 10.2, -3.6, -4.4, -4.7, -5.2 ppm; **IR** (thin film): υ_{max} = 3416, 3070, 2960, 2929, 2857, 1754, 1710, 1681, 1504, 1471 cm^{-1}; **HRMS-ESI** (*m/z*): calcd. For $C_{53}H_{82}O_7NSi_3$ $[M + H]^+$: 928.5394, found: 928.5399.

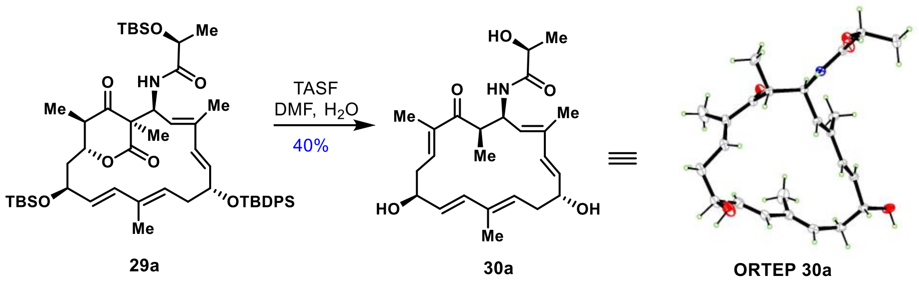

To a solution of macrocycle **29a** (14 mg, 0.015 mmol) in DMF (4 mL) was added TASF (62 mg, 0.23 mmol) and H_2O (10 μL, 0.56 mmol) dissolved in DMF (0.5 mL). The resulting mixture was stirred for 60 hours at room temperature and quenched by saturated NH_4Cl (*aq.*, 30 mL), the resulting mixture was extracted with EtOAc (20 mL x 4), the

combined organic layer was dried over anhydrous Na_2SO_4, filtered and concentrated, the residue was purified by preparative TLC (CH_2Cl_2/MeOH = 10/1) to afford **30a** (2.5 mg, 40% yield) as a white solid.

Data for **30a**: $[\alpha]_D^{25}$ = +92.7 (*c* = 0.10, MeOH); **^{1}H NMR** (500 MHz, CD_3OD) δ 6.72 (dd, *J* = 9.8, 4.9 Hz, 1H), 6.06 (d, *J* = 15.7 Hz, 1H), 5.76 (d, *J* = 15.8 Hz, 1H), 5.44 (dd, *J* = 15.6, 5.1 Hz, 1H), 5.37 (dd, *J* = 15.8, 7.8 Hz, 1H), 5.23 – 5.16 (m, 2H), 5.03 (t, *J* = 10.3 Hz, 1H), 4.53 (brs, 1H), 4.12 (q, *J* = 6.8 Hz, 1H), 4.05 (ddd, *J* = 10.6, 7.8, 4.6 Hz, 1H), 3.55 (dq, *J* = 10.5, 6.8 Hz, 1H), 2.73 (ddd, *J* = 13.8, 10.3, 3.1 Hz, 1H), 2.53 – 2.47 (m, 2H), 2.18 (ddd, *J* = 13.0, 10.2, 9.4 Hz, 1H), 1.73 (s, 3H), 1.70 (d, *J* = 1.1 Hz, 3H), 1.60 (s, 3H), 1.31 (t, *J* = 5.8 Hz, 3H), 0.99 (d, *J* = 6.8 Hz, 3H) ppm; **^{13}C NMR** (125 MHz, CD_3OD) δ 205.8, 177.1, 140.0, 139.2, 136.8, 135.8, 135.5, 135.3, 131.5, 131.3, 129.6, 127.7, 74.9, 71.4, 69.1, 50.6, 44.7, 37.4, 36.7, 21.2, 16.1, 13.6, 12.8, 12.5 ppm; **IR** (thin film): υ_{max} = 3407, 3349, 2920, 2850, 1651, 1642, 1526, 1366, 1277, 1123, 955 cm^{-1}; **HRMS-ESI** (*m/z*): calcd. For $C_{24}H_{36}NO_5$ $[M + H]^+$: 418.2588, found: 418.2583

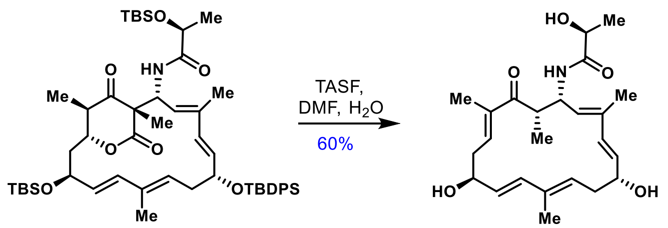

To a solution of macrocycle **29b** (15 mg, 0.016 mmol) in DMF (3 mL) was added TASF (66 mg, 0.24 mmol) and H_2O (15 µL, 0.83 mmol) dissolved in DMF (0.5 mL). The resulting mixture was stirred for 24 hours at room temperature and quenched by saturated NH_4Cl (*aq.*, 20 mL), the resulting mixture was extracted with EtOAc (30 mL x 4), the combined organic layer was dried over anhydrous Na_2SO_4, filtered and concentrated, the residue was purified by preparative TLC (CH_2Cl_2/MeOH = 10/1) to afford **30b** (4 mg, 60% yield) as a white solid.

Data for **30b**: $[\alpha]_D^{25}$ = −193.3 (*c* = 0.20, MeOH); **^{1}H NMR** (500 MHz, CD_3OD) δ 6.58 (dd, *J* = 8.9, 6.3 Hz, 1H), 6.08 (d, *J* = 15.5 Hz, 1H), 6.03 (d, *J* = 15.9 Hz, 1H), 5.52 (dd, *J* =

15.9, 4.6 Hz, 1H), 5.49 (dd, *J* = 10.2, 5.2 Hz, 1H), 5.35 (dd, *J* = 15.5, 8.3 Hz, 1H), 5.16 (d, *J* = 9.9 Hz, 1H), 5.07(t, *J* = 10.1 Hz, 1H), 4.53 – 4.50 (m, 1H), 4.27 (td, *J* = 8.7, 5.5 Hz, 1H), 4.09 (q, *J* = 6.8 Hz, 1H), 3.55 (dq, *J* = 10.2, 6.9 Hz, 1H), 2.65 – 2.57 (m, 2H), 2.46 – 2.34 (m, 2H), 1.72 (s, 3H), 1.66 (d, *J* = 1.2 Hz, 3H), 1.53 (s, 3H), 1.35 (d, *J* = 6.8Hz, 3H), 0.98 (d, *J* = 6.9 Hz, 3H) ppm; **^{13}C NMR** (125 MHz, CD_3OD) δ 205.5, 177.1, 139.7, 139.2, 138.1, 136.0, 135.7, 135.5, 130.6, 130.2, 129.1, 128.7, 73.6, 70.5, 69.3, 50.3, 44.6, 38.5, 35.1, 21.5, 16.3, 13.2, 12.9, 12.6 ppm; **IR** (thin film): υ_{max} = 3305, 3024, 2976, 2923, 2852, 1665, 1618, 1550, 1451, 1366, 1128 cm^{-1}; **HRMS-ESI** (*m/z*): calcd. For $C_{24}H_{36}NO_5$ $[M + H]^+$: 418.2588, found: 418.2584.

3. NMR Comparisons of the Reported O3P2 and Synthetic Stereoisomers

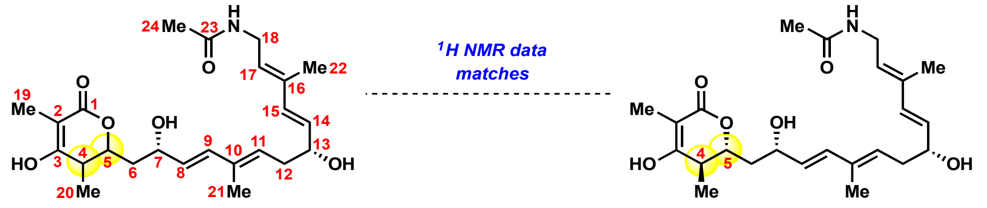

Table S1. Comparison of ^{1}H NMR data with the synthetic (4*R*,5*R*)-O3P2.

position	Reported O3P2 (Kim et al.[a]) ^{1}H NMR (500 MHz, CD_3OD) δ [ppm, mult, *J* (Hz)]	Synthetic 4*R*,5*R*-O3P2 (this work) ^{1}H NMR (400 MHz, CD_3OD) δ [ppm, mult, *J* (Hz)]	Δδ / ppm
19	1.72 (s, 3H)	1.72 (s, 3H)	0
4	2.48 (m, 1H)	2.50 (m, 1H)	+0.02
20	1.25 (d, *J* = 7.5 Hz, 3H)	1.25 (d, *J* = 7.2 Hz, 3H)	0
5	4.15 (m, 1H)	4.15 (m, 1H)	0
6α	1.77 (m, 1H)	1.78 (m, 1H)	+0.01
6β	2.08 (m, 1H)	2.08 (m, 1H)	0
7	4.29 (q, *J* = 7.0 Hz, 1H)	4.28 (q, *J* = 6.4 Hz, 1H)	−0.01
8	5.57 (dd, *J* = 15.5, 7.0 Hz, 1H)	5.56 (dd, *J* = 15.4, 7.6 Hz, 1H)	−0.01
9	6.30 (d, *J* = 15.5 Hz, 1H)	6.29 (d, *J* = 15.6 Hz, 1H)	−0.01
21	1.77 (s, 3H)	1.77 (s, 3H)	0
11	5.55 (t, *J* = 7.3 Hz, 1H)	5.54 (t, *J* = 7.2 Hz, 1H)	−0.01
12α	2.39 (m, 1H)	2.40 (m, 1H)	+0.01
12β	2.43 (m, 1H)	2.44 (m, 1H)	+0.01
13	4.17 (m, 1H)	4.17 (m, 1H)	0
14	5.70 (dd, *J* = 15.7, 6.7 Hz, 1H)	5.69 (dd, *J* = 15.6, 6.4 Hz, 1H)	−0.01
15	6.23 (d, *J* = 15.5 Hz, 1H)	6.23 (d, *J* = 15.2 Hz, 1H)	0
22	1.80 (s, 3H)	1.79 (s, 3H)	−0.01
17	5.46 (t, *J* = 6.8 Hz, 1H)	5.46 (t, *J* = 6.8 Hz, 1H)	0
18	3.90 (d, *J* = 7.0 Hz, 2H)	3.89 (d, *J* = 7.2 Hz, 2H)	−0.01
24	1.94 (s, 3H)	1.93 (s, 3H)	−0.01

[a]Kim, B. S.; Oh, H.; Ng, B.-G.; Han, J.-W. Biosynthetic Gene Cluster for Chejuenolide of Marine Microorganism *Hahella Chejuensis*. United States Patent, US 9394553 B2, 2016.

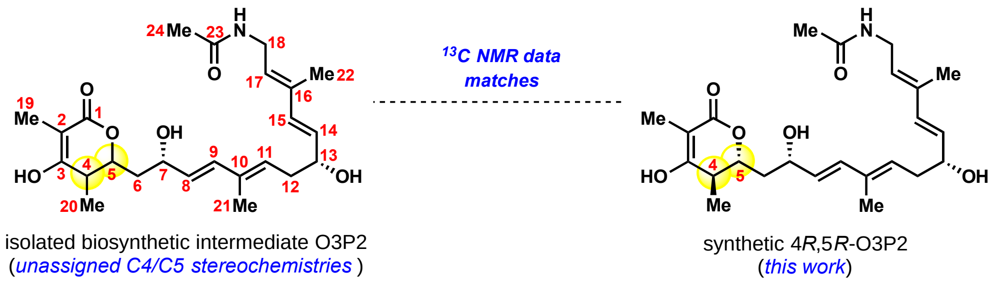

Table S2. Comparison of ^{13}C NMR data with the synthetic (4*R*,5*R*)-O3P2.

position	Reported O3P2 (Kim et al.[a]) ^{13}C NMR (125 MHz, CD_3OD) δ /ppm	Synthetic 4*R*,5*R*-O3P2 (this work) ^{13}C NMR (100 MHz, CD_3OD) δ /ppm	Δδ / ppm
1	169.5	170.6	+1.1
2	96.0	98.0	+2.0
19	7.2	8.6	+1.4
3	171.2	171.1	−0.1
4	37.0	38.0	+1.0
20	15.2	16.7	+1.5
5	78.0	79.4	+1.4
6	40.6	42.0	+1.4
7	69.4	70.8	+1.4
8	128.3	129.7	+1.4
9	136.0	137.4	+1.4
10	134.5	135,9	+1.4
21	11.2	12.6	+1.4
11	128.2	129.7	+1.5
12	36.2	37.6	+1.4
13	71.9	73.3	+1.4
14	130.9	132.3	+1.4
15	133.8	135.2	+1.4
16	135.5	136.9	+1.4
22	11.2	12.9	+1.7
17	126.9	128.3	+1.4
18	37.0	38.4	+1.4
23	171.6	173.0	+1.4
24	21.0	22.5	+1.5

[a]Kim, B. S.; Oh, H.; Ng, B.-G.; Han, J.-W. Biosynthetic Gene Cluster for Chejuenolide of Marine Microorganism *Hahella Chejuensis*. United States Patent, US 9394553 B2, 2016.

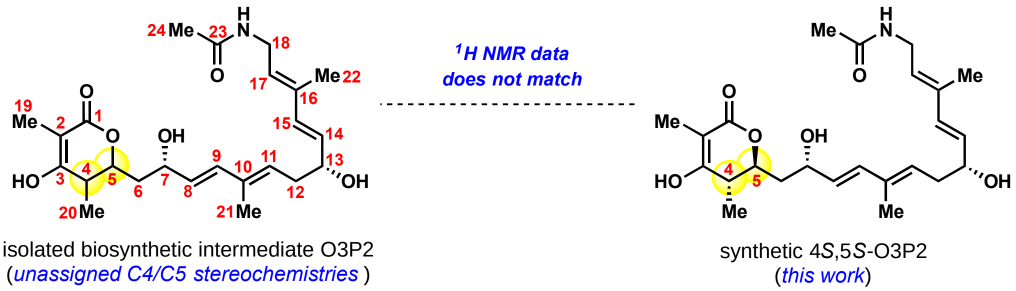

Table S3. Comparison of ^{1}H NMR data with the synthetic (4*S*,5*S*)-O3P2.

position	Reported O3P2 (Kim et al.[a]) ^{1}H NMR (500 MHz, CD_3OD) δ [ppm, mult, *J* (Hz)]	Synthetic 4*S*,5*S*-O3P2 (this work) ^{1}H NMR (400 MHz, CD_3OD) δ [ppm, mult, *J* (Hz)]	Δδ / ppm
19	1.72 (s, 3H)	1.72 (s, 3H)	0
4	2.48 (m, 1H)	2.46 (m, 1H)	−0.02
20	1.25 (d, *J* = 7.5 Hz, 3H)	1.27 (d, *J* = 7.2 Hz, 3H)	+0.02
5	4.15 (m, 1H)	4.36 (m, 1H)	+0.21
6α	1.77 (m, 1H)	1.71 (m, 1H)	−0.06
6β	2.08 (m, 1H)	1.90 (ddd, *J* = 14.4, 10.4, 3.2 Hz, 1H)	−0.18
7	4.29 (q, *J* = 7.0 Hz, 1H)	4.34 (m, 1H)	+0.05
8	5.57 (dd, *J* = 15.5, 7.0 Hz, 1H)	5.59 (dd, *J* = 15.6, 6.8 Hz, 1H)	+0.02
9	6.30 (d, *J* = 15.5 Hz, 1H)	6.25 (d, *J* = 15.6 Hz, 1H)	−0.05
21	1.77 (s, 3H)	1.74 (s, 3H)	−0.03
11	5.55 (t, *J* = 7.3 Hz, 1H)	5.51 (t, *J* = 7.6 Hz, 1H)	−0.04
12α	2.39 (m, 1H)	2.38 (m, 1H)	−0.01
12β	2.43 (m, 1H)	2.42 (m, 1H)	−0.01
13	4.17 (m, 1H)	4.16 (q, *J* = 6.4 Hz, 1H)	−0.01
14	5.70 (dd, *J* = 15.7, 6.7 Hz, 1H)	5.68 (dd, *J* = 15.6, 6.8 Hz, 1H)	−0.02
15	6.23 (d, *J* = 15.5 Hz, 1H)	6.22 (d, *J* = 15.6 Hz, 1H)	−0.01
22	1.80 (s, 3H)	1.79 (s, 3H)	−0.01
17	5.46 (t, *J* = 6.8 Hz, 1H)	5.46 (t, *J* = 6.8 Hz, 1H)	0
18	3.90 (d, *J* = 7.0 Hz, 2H)	3.89 (d, *J* = 6.8 Hz, 2H)	−0.01
24	1.94 (s, 3H)	1.93 (s, 3H)	−0.01

[a]Kim, B. S.; Oh, H.; Ng, B.-G.; Han, J.-W. Biosynthetic Gene Cluster for Chejuenolide of Marine Microorganism *Hahella Chejuensis*. United States Patent, US 9394553 B2, 2016.

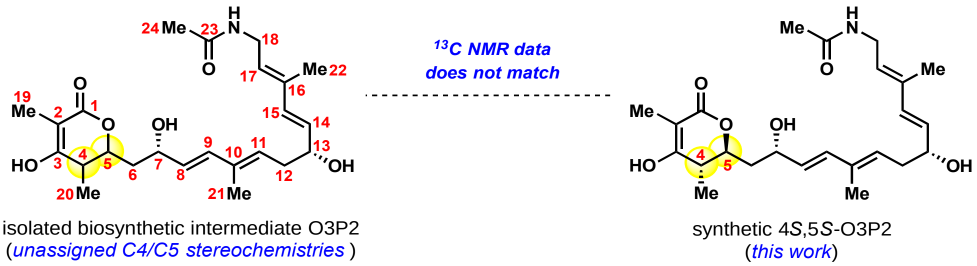

Table S4. Comparison of ^{13}C NMR data with the synthetic (4*S*,5*S*)-O3P2.

position	Reported O3P2 (Kim et al.[a]) ^{13}C NMR (125 MHz, CD$_3$OD) δ /ppm	Synthetic 4*S*,5*S*-O3P2 (this work) ^{13}C NMR (100 MHz, CD$_3$OD) δ /ppm	Δδ / ppm
1	169.5	170.7	+1.2
2	96.0	98.1	+2.1
19	7.2	8.6	+1.4
3	171.2	171.3	+0.1
4	37.0	38.5	+1.5
20	15.2	16.6	+1.4
5	78.0	78.8	+0.8
6	40.6	42.6	+2.0
7	69.4	69.5	+0.1
8	128.3	130.7	+2.4
9	136.0	136.0	0
10	134.5	135.9	+1.4
21	11.2	12.9	+1.7
11	128.2	129.3	+1.1
12	36.2	37.5	+1.3
13	71.9	73.3	+1.4
14	130.9	132.3	+1.4
15	133.8	135.2	+1.4
16	135.5	136.9	+1.4
22	11.2	12.6	+1.4
17	126.9	128.3	+1.4
18	37.0	38.4	+1.4
23	171.6	173.0	+1.4
24	21.0	22.5	+1.5

[a]Kim, B. S.; Oh, H.; Ng, B.-G.; Han, J.-W. Biosynthetic Gene Cluster for Chejuenolide of Marine Microorganism *Hahella Chejuensis*. United States Patent, US 9394553 B2, 2016.

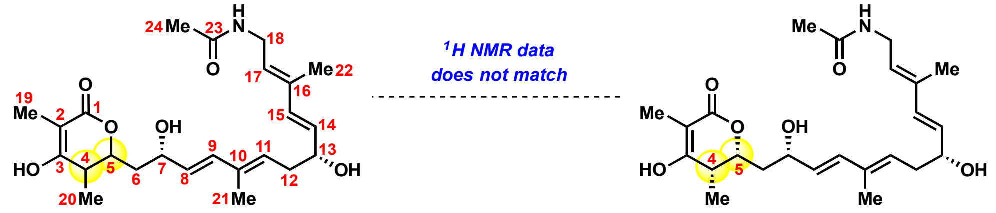

Table S5. Comparison of ^{1}H NMR data with the synthetic (4*S*,5*R*)-O3P2.

position	Reported O3P2 (Kim et al.[a])<br>^{1}H NMR (500 MHz, CD_3OD)<br>δ [ppm, mult, *J* (Hz)]	Synthetic 4*S*,5*R*-O3P2 (this work)<br>^{1}H NMR (500 MHz, CD_3OD)<br>δ [ppm, mult, *J* (Hz)]	Δδ / ppm
19	1.72 (s, 3H)	1.70 (s, 3H)	−0.02
4	2.48 (m, 1H)	2.40 (m, 1H)	−0.08
20	1.25 (d, *J* = 7.5 Hz, 3H)	1.12 (d, *J* = 7.0 Hz, 3H)	−0.13
5	4.15 (m, 1H)	4.37 (ddd, *J* = 8.5, 5.2, 3.2 Hz, 1H)	+0.22
6α	1.77 (m, 1H)	1.70 (m, 1H)	−0.07
6β	2.08 (m, 1H)	2.05 (ddd, *J* = 13.9, 8.6, 6.3 Hz, 1H)	−0.03
7	4.29 (q, *J* = 7.0 Hz, 1H)	4.29 (m, 1H)	0
8	5.57 (dd, *J* = 15.5, 7.0 Hz, 1H)	5.57 (dd, *J* = 15.7, 7.5 Hz, 1H)	0
9	6.30 (d, *J* = 15.5 Hz, 1H)	6.28 (d, *J* = 15.6 Hz, 1H)	−0.02
21	1.77 (s, 3H)	1.76 (d, *J* = 1.2 Hz, 3H)	−0.01
11	5.55 (t, *J* = 7.3 Hz, 1H)	5.54 (t, *J* = 7.4 Hz, 1H)	−0.01
12α	2.39 (m, 1H)	2.40 (m, 1H)	+0.01
12β	2.43 (m, 1H)	2.44 (m, 1H)	+0.01
13	4.17 (m, 1H)	4.16 (qd, *J* = 6.6, 1.2 Hz, 1H)	−0.01
14	5.70 (dd, *J* = 15.7, 6.7 Hz, 1H)	5.68 (dd, *J* = 15.7, 6.7 Hz, 1H)	−0.02
15	6.23 (d, *J* = 15.5 Hz, 1H)	6.23 (d, *J* = 15.7 Hz, 1H)	0
22	1.80 (s, 3H)	1.79 (d, *J* = 1.1 Hz, 3H)	−0.01
17	5.46 (t, *J* = 6.8 Hz, 1H)	5.46 (t, *J* = 6.9 Hz, 1H)	0
18	3.90 (d, *J* = 7.0 Hz, 2H)	3.89 (d, *J* = 7.0 Hz, 2H)	−0.01
24	1.94 (s, 3H)	1.93 (s, 3H)	−0.01

[a]Kim, B. S.; Oh, H.; Ng, B.-G.; Han, J.-W. Biosynthetic Gene Cluster for Chejuenolide of Marine Microorganism *Hahella Chejuensis*. United States Patent, US 9394553 B2, 2016.

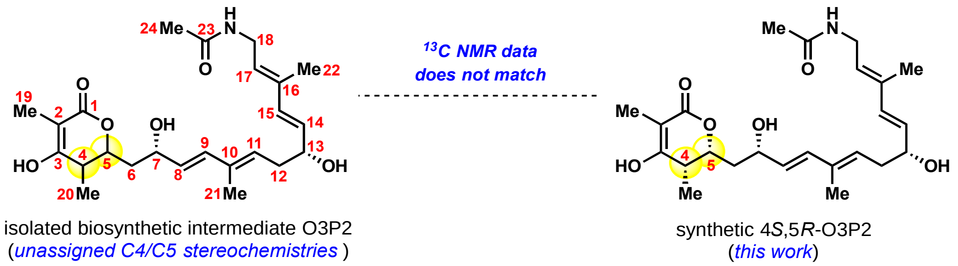

Table S6. Comparison of ^{13}C NMR data with the synthetic (4*S*,5*R*)-O3P2.

position	Reported O3P2 (Kim et al.[a]) ^{13}C NMR (125 MHz, CD_3OD) δ /ppm	Synthetic 4*S*,5*R*-O3P2 (this work) ^{13}C NMR (125 MHz, CD_3OD) δ /ppm	Δδ / ppm
1	169.5	172.2	+2.7
2	96.0	97.9	+1.9
19	7.2	8.7	+1.5
3	171.2	174.2	+3.0
4	37.0	37.5	+0.5
20	15.2	11.0	−4.2
5	78.0	76.4	−1.6
6	40.6	39.6	−1.0
7	69.4	70.6	+1.2
8	128.3	129.7	+1.4
9	136.0	137.5	+1.5
10	134.5	135.9	+1.4
21	11.2	12.9	+1.7
11	128.2	129.9	+1.7
12	36.2	37.6	+1.4
13	71.9	73.3	+1.4
14	130.9	132.3	+1.4
15	133.8	135.2	+1.4
16	135.5	136.9	+1.4
22	11.2	12.6	+1.4
17	126.9	128.3	+1.4
18	37.0	38.4	+1.4
23	171.6	173.0	+1.4
24	21.0	22.5	+1.5

[a]Kim, B. S.; Oh, H.; Ng, B.-G.; Han, J.-W. Biosynthetic Gene Cluster for Chejuenolide of Marine Microorganism *Hahella Chejuensis*. United States Patent, US 9394553 B2, 2016.

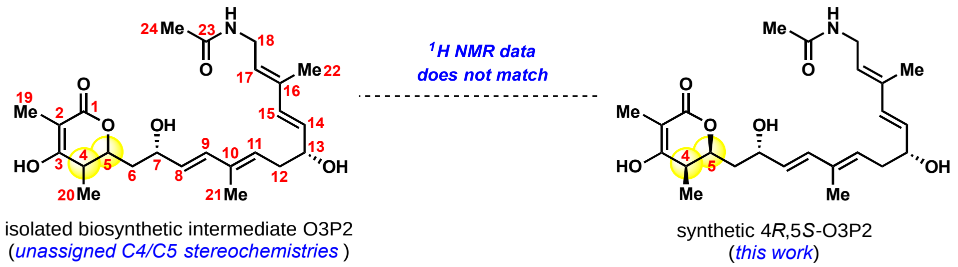

Table S7. Comparison of ^{1}H NMR data with the synthetic (4*R*,5*S*)-O3P2.

position	Reported O3P2 (Kim et al.[a]) ^{1}H NMR (500 MHz, CD_3OD) δ [ppm, mult, *J* (Hz)]	Synthetic 4*R*,5*S*-O3P2 (this work) ^{1}H NMR (500 MHz, CD_3OD) δ [ppm, mult, *J* (Hz)]	Δδ / ppm
19	1.72 (s, 3H)	1.71 (s, 3H)	−0.01
4	2.48 (m, 1H)	2.37 (m, 1H)	−0.11
20	1.25 (d, *J* = 7.5 Hz, 3H)	1.12 (d, *J* = 7.0 Hz, 3H)	−0.13
5	4.15 (m, 1H)	4.58 (dt, *J* = 9.7, 3.2 Hz, 1H)	+0.43
6α	1.77 (m, 1H)	1.62 (ddd, *J* = 14.0, 10.0, 3.3 Hz, 1H)	−0.15
6β	2.08 (m, 1H)	1.89 (ddd, *J* = 14.1, 9.6, 3.2 Hz, 1H)	−0.19
7	4.29 (q, *J* = 7.0 Hz, 1H)	4.36 (ddd, *J* = 10.0, 6.7, 3.1 Hz, 1H)	+0.07
8	5.57 (dd, *J* = 15.5, 7.0 Hz, 1H)	5.64 (dd, *J* = 15.7, 6.7 Hz, 1H)	+0.07
9	6.30 (d, *J* = 15.5 Hz, 1H)	6.28 (d, *J* = 15.7 Hz, 1H)	−0.02
21	1.77 (s, 3H)	1.76 (d, *J* = 1.3 Hz, 3H)	−0.01
11	5.55 (t, *J* = 7.3 Hz, 1H)	5.53 (t, *J* = 7.4 Hz, 1H)	−0.02
12α	2.39 (m, 1H)	2.40 (m, 1H)	+0.01
12β	2.43 (m, 1H)	2.44 (m, 1H)	+0.01
13	4.17 (m, 1H)	4.17 (app q, *J* = 6.6 Hz, 1H)	0
14	5.70 (dd, *J* = 15.7, 6.7 Hz, 1H)	5.69 (dd, *J* = 15.8, 6.7 Hz, 1H)	−0.01
15	6.23 (d, *J* = 15.5 Hz, 1H)	6.23 (d, *J* = 15.7 Hz, 1H)	0
22	1.80 (s, 3H)	1.79 (d, *J* = 1.4 Hz, 3H)	−0.01
17	5.46 (t, *J* = 6.8 Hz, 1H)	5.46 (t, *J* = 7.1 Hz, 1H)	0
18	3.90 (d, *J* = 7.0 Hz, 2H)	3.89 (d, *J* = 7.0 Hz, 2H)	−0.01
24	1.94 (s, 3H)	1.93 (s, 3H)	−0.01

[a]Kim, B. S.; Oh, H.; Ng, B.-G.; Han, J.-W. Biosynthetic Gene Cluster for Chejuenolide of Marine Microorganism *Hahella Chejuensis*. United States Patent, US 9394553 B2, 2016.

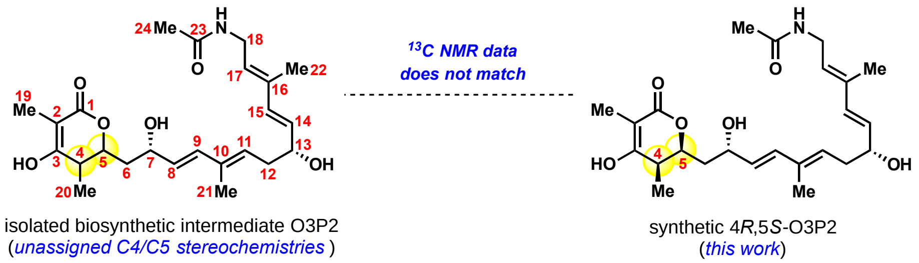

Table S8. Comparison of ^{13}C NMR data with the synthetic (4*R*,5*S*)-O3P2.

position	Reported O3P2 (Kim et al.[a]) ^{13}C NMR (125 MHz, CD_3OD) δ /ppm	Synthetic 4*R*,5*S*-O3P2 (this work) ^{13}C NMR (125 MHz, CD_3OD) δ /ppm	Δδ / ppm
1	169.5	172.2	+2.7
2	96.0	97.9	+1.9
19	7.2	8.7	+1.5
3	171.2	174.2	+3.0
4	37.0	38.1	+1.1
20	15.2	11.1	−4.1
5	78.0	75.8	−2.2
6	40.6	40.2	−0.4
7	69.4	69.2	−0.2
8	128.3	130.7	+2.4
9	136.0	136.1	+0.1
10	134.5	136.0	+1.5
21	11.2	12.9	+1.7
11	128.2	129.4	+1.2
12	36.2	37.5	+1.3
13	71.9	73.3	+1.4
14	130.9	132.3	+1.4
15	133.8	135.2	+1.4
16	135.5	136.9	+1.4
22	11.2	12.6	+1.4
17	126.9	128.3	+1.4
18	37.0	38.4	+1.4
23	171.6	173.0	+1.4
24	21.0	22.5	+1.5

[a]Kim, B. S.; Oh, H.; Ng, B.-G.; Han, J.-W. Biosynthetic Gene Cluster for Chejuenolide of Marine Microorganism *Hahella Chejuensis*. United States Patent, US 9394553 B2, 2016.

4. NMR Comparisons of the Reported and Synthetic chejuenolide A, B, and C

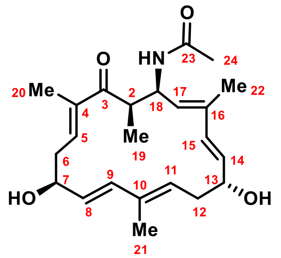

chejuenolide A

Comparison of ^{1}H-NMR of the Reported and Synthetic Chejuenolide A

position	Reported chejuenolide A (Oh et al.[a]) ^{1}H NMR (400 MHz, CD_3OD) δ [ppm, mult, *J* (Hz)]	Synthetic chejuenolide A (this work) ^{1}H NMR (500 MHz, CD_3OD) δ [ppm, mult, *J* (Hz)]	Δδ/ ppm
N-*H*	/	/	/
2	3.40(dq, *J* = 10.3 6.6Hz, 1H)	3.42 (dq, *J* = 10.4, 6.9 Hz, 1H)	+0.02
5	6.70 (dd, *J* = 10.3, 5.3 Hz, 1H)	6.71 (dd, *J* = 10.1, 5.2 Hz, 1H)	+0.01
6α	2.72 (ddd, *J* = 14.0, 10.3, 3.2 Hz, 1H)	2.73 (ddd, *J* = 13.7, 10.3, 3.2 Hz, 1H)	+0.01
6β	2.45 (m, 1H)	2.53 – 2.47 (m, 1H)	/
7	4.52 (brm, 1H)	4.52 (brm, 1H)	0
8	5.42 (dd, *J* = 15.7, 5.2 Hz, 1H)	5.43 (dd, *J* = 15.6, 5.2 Hz, 1H)	+0.01
9	6.04 (d, *J* = 15.7 Hz, 1H)	6.05 (d, *J* = 15.5 Hz, 1H)	+0.01
11	5.18 (t, *J* = 8.7 Hz, 1H)	5.19 (t, *J* = 7.8 Hz, 1H)	+0.01
12α	2.17 (ddd, *J* = 12.9, 10.6, 8.7 Hz, 1H)	2.18 (ddd, *J* = 13.1, 10.3, 9.1 Hz, 1H)	+0.01
12β	2.55 (m, 1H)	2.53 – 2.47 (m, 1H)	/
13	4.04 (ddd, *J* = 10.6, 8.0, 4.8 Hz, 1H)	4.05 (ddd, *J* = 10.4, 7.8, 4.7 Hz, 1H)	+0.01
14	5.36 (dd, *J* = 15.9, 8.0 Hz, 1H)	5.36 (dd, *J* = 15.8, 7.7 Hz, 1H)	0
15	5.74 (d, *J* = 15.9 Hz, 1H)	5.75 (d, *J* = 15.8 Hz, 1H)	+0.01
17	5.08 (d, *J* = 10.3 Hz, 1H)	5.10 (d, *J* = 10.4 Hz, 1H)	+0.02
18	4.98 (t, *J* = 10.3 Hz, 1H)	4.99 (t, *J* = 10.3 Hz, 1H)	+0.01
19	0.99 (d, *J* = 6.6 Hz, 3H)	1.00 (d, *J* = 6.8 Hz, 3H)	+0.01
20	1.71 (s, 3H)	1.72 (s, 3H)	+0.01
21	1.58 (s, 3H)	1.60 (s, 3H)	+0.02
22	1.68 (d, *J* = 1.1 Hz, 3H)	1.69 (d, *J* = 1.2 Hz, 3H)	+0.01
24	1.93 (s, 3H)	1.94 (s, 3H)	+0.01

[a] Choi, Y.-H.; Sohn, J.-H.; Lee, D.; Kim, J. K.; Kong, I. S.; Ahn, S. C.; Oh, H. Chejuenolides A and B, new macrocyclic tetraenes from the marine bacterium *Hahella chejuensis*. *Tetrahedron Lett*. **2008**, *49*, 7128.

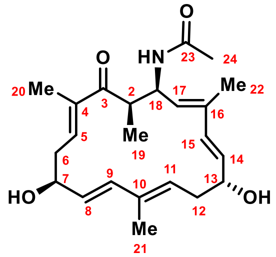

chejuenolide A

Comparison of ^{13}C-NMR of the Reported and Synthetic Chejuenolide A

position	Reported chejuenolide A (Oh et al.[a]) ^{13}C NMR (100 MHz, CD_3O) Δδ /ppm	Synthetic chejuenolide A (this work) ^{13}C NMR (125 MHz, CD_3OD) Δδ /ppm	Δδ / ppm
2	44.9	44.9	0
3	205.6	205.7	+0.1
4	140.0	140.0	0
5	139.2	139.2	0
6	37.4	37.4	0
7	71.3	71.3	0
8	129.6	129.6	0
9	135.7	135.8	+0.1
10	135.3	135.3	0
11	127.7	127.7	0
12	36.2	36.7	+0.5
13	74.8	74.8	0
14	131.3	131.3	0
15	136.7	136.8	+0.1
16	135.3	135.4	+0.1
17	131.6	131.6	0
18	51.0	51.0	0
19	16.1	16.1	0
20	12.5	12.5	0
21	13.6	13.6	0
22	12.8	12.8	0
23	172.4	172.4	0
24	22.7	22.8	+0.1

[a] Choi, Y.-H.; Sohn, J.-H.; Lee, D.; Kim, J. K.; Kong, I. S.; Ahn, S. C.; Oh, H. Chejuenolides A and B, new macrocyclic tetraenes from the marine bacterium *Hahella chejuensis*. *Tetrahedron Lett*. **2008**, *49*, 7128.

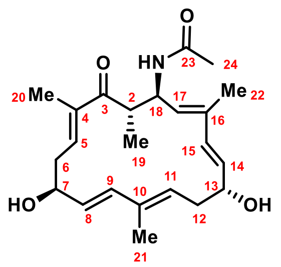

chejuenolide B

Comparison of ^{1}H-NMR of the Reported and Synthetic Chejuenolide B

position	Reported chejuenolide B (Oh et al.[a]) ^{1}H NMR (400 MHz, CD_3OD) δ [ppm, mult, *J* (Hz)]	Synthetic chejuenolide B (this work) ^{1}H NMR (400 MHz, CD_3OD) δ [ppm, mult, *J* (Hz)]	Δδ / ppm
N-*H*	/	/	/
2	3.43(qd, *J* = 7.0, 3.7Hz, 1H)	3.42 (qd, *J* = 7.2, 3.7 Hz, 1H)	-0.01
5	6.47 (brt, *J* = 7.7 Hz, 1H)	6.48 (dd, *J* = 7.8, 6.4Hz, 1H)	+0.01
6a	2.66 (dt, *J* = 15.4, 7.7 Hz, 1H)	2.71 – 2.63 (m, 1H)	/
6b	2.53 (m, 1H)	2.57 – 2.47 (m, 2H)	/
7	4.47(brm, 1H)	4.48 (brs, 1H)	+0.01
8	5.53 (dd, *J* = 15.8, 5.5 Hz, 1H)	5.54 (dd, *J* = 15.7, 5.3 Hz, 1H)	+0.01
9	6.13 (d, *J* = 15.7 Hz, 1H)	6.14 (d, *J* = 15.7 Hz, 1H)	+0.01
11	5.24 (dd, *J* = 9.5, 6.2Hz, 1H)	5.24 (t, *J* = 8.2 Hz, 1H)	0
12a	2.27 (dt, *J* = 12.4, 10.2 Hz, 1H)	2.25 (dt, *J* = 12.6, 10.4 Hz, 1H))	-0.02
12b	2.49 (m, 1H)	2.57 – 2.47 (m, 2H)	/
13	4.05 (ddd, *J* = 10.2, 8.8, 5.2 Hz, 1H)	4.05 (ddd, *J* = 10.2, 8.9, 5.1 Hz, 1H)	0
14	5.42 (dd, *J* = 15.8, 8.8 Hz, 1H)	5.43 (dd, *J* = 15.6, 8.5 Hz, 1H)	+0.01
15	5.70 (d, *J* = 15.8 Hz, 1H)	5.71 (d, *J* = 15.7 Hz, 1H)	+0.01
17	5.05 (d, *J* = 9.5 Hz, 1H)	5.06 (d, *J* = 9.9 Hz, 1H)	+0.01
18	4.90 (m, 1H)	4.91 (dd, *J* = 9.9, 3.5 Hz, 1H)	+0.01
19	1.07 (d, *J* = 7.0 Hz, 3H)	1.08 (d, *J* = 7.1 Hz, 3H)	+0.01
20	1.80 (s, 3H)	1.81 (s, 3H)	+0.01
21	1.64 (s, 3H)	1.65 (s, 3H)	+0.01
22	1.79 (d, *J* = 1.1 Hz, 3H)	1.81 (s, 3H)	+0.02
24	1.99 (s, 3H)	2.00 (s, 3H)	+0.01

[a] Choi, Y.-H.; Sohn, J.-H.; Lee, D.; Kim, J. K.; Kong, I. S.; Ahn, S. C.; Oh, H. Chejuenolides A and B, new macrocyclic tetraenes from the marine bacterium *Hahella chejuensis*. *Tetrahedron Lett*. **2008**, *49*, 7128.

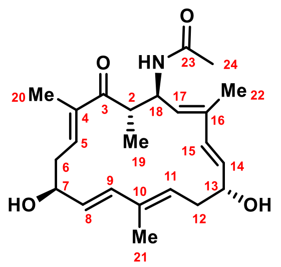

chejuenolide B

Comparison of ^{13}C-NMR of the Reported and Synthetic Chejuenolide B

position	Reported chejuenolide B (Oh et al.[a]) ^{13}C NMR (100 MHz, CD_3O) δ /ppm	Synthetic chejuenolide B (this work) ^{13}C NMR (100 MHz, CD_3OD) δ /ppm	Δδ / ppm
2	43.0	43.1	+0.1
3	208.9	208.9	0
4	140.18	140.19	+0.01
5	140.14	140.15	+0.01
6	37.8	37.8	0
7	71.9	71.9	0
8	128.9	128.9	0
9	136.1	136.0	-0.1
10	135.9	135.9	0
11	128.8	128.8	0
12	36.9	36.9	0
13	75.3	75.1	-0.2
14	131.6	131.6	0
15	136.4	136.4	0
16	135.8	135.8	0
17	131.3	131.3	0
18	51.5	51.5	0
19	16.3	16.3	0
20	12.5	12.5	0
21	13.1	13.1	0
22	12.3	12.3	0
23	172.5	172.5	0
24	22.9	22.9	0

[a] Choi, Y.-H.; Sohn, J.-H.; Lee, D.; Kim, J. K.; Kong, I. S.; Ahn, S. C.; Oh, H. Chejuenolides A and B, new macrocyclic tetraenes from the marine bacterium *Hahella chejuensis*. *Tetrahedron Lett*. **2008**, *49*, 7128.

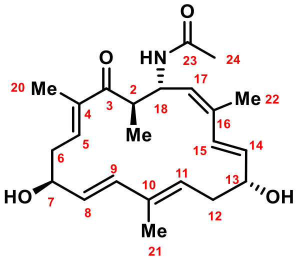

chejuenolide C

Comparison of ^{1}H-NMR of the Reported and Synthetic Chejuenolide C

position	Reported chejuenolide C (Oh et al.[a]) ^{1}H NMR (400 MHz, CD_3OD) δ [ppm, mult, *J* (Hz)]	Synthetic chejuenolide C (this work) ^{1}H NMR (500 MHz, CD_3OD) δ [ppm, mult, *J* (Hz)]	Δδ / ppm
N-*H*	/	/	/
2	3.47(qd, *J* = 7.0, 3.9Hz, 1H)	3.47 (qd, *J* = 7.0, 4.1 Hz, 1H)	0
5	6.84 (brt, *J* = 6.2 Hz, 1H)	6.85 (t, *J* = 6.5 Hz, 1H)	+0.01
6α	2.65 (ddd, *J* = 16.5, 8.1, 2.9 Hz, 1H)	2.63 (ddd, *J* = 16.1, 7.9, 3.0 Hz, 1H)	-0.02
6β	2.52 (m, 1H)	2.57 – 2.47 (m, 2H)	/
7	4.47 (brs, 1H)	4.48 (brs, 1H)	+0.01
8	5.51 (dd, *J* = 15.8, 5.5 Hz, 1H)	5.52 (dd, *J* = 15.7, 4.8 Hz, 1H)	+0.01
9	6.29 (d, *J* = 15.8 Hz, 1H)	6.30 (d, *J* = 15.7 Hz, 1H)	+0.01
11	5.35 (dd, *J* = 9.9, 7.3 Hz, 1H)	5.36 (dd, *J* = 9.3, 7.1 Hz, 1H)	+0.01
12α	2.31 (m, 1H)	2.32 (dt, *J* = 12.7, 10.5 Hz, 1H)	+0.01
12β	2.47(m, 1H)	2.57 – 2.47 (m, 2H)	/
13	4.10 (ddd, *J* = 11.0, 8.8, 4.4 Hz, 1H)	4.11 (ddd, *J* = 10.9, 8.6, 4.4 Hz, 1H)	+0.01
14	5.53 (dd, *J* = 15.8, 8.8 Hz, 1H)	5.55 (dd, *J* = 15.8, 8.6 Hz, 1H)	+0.02
15	6.19 (d, *J* = 15.8 Hz, 1H)	6.20 (d, *J* = 15.5 Hz, 1H)	+0.01
17	5.21 (d, *J* = 7.7 Hz, 1H)	5.22 (d, *J* = 8.0 Hz, 1H)	+0.01
18	4.92 (m, 1H)	4.94 (td, *J* = 10.5, 5.1 Hz, 1H)	+0.02
19	1.11 (d, *J* = 7.0 Hz, 3H)	1.12 (d, *J* = 7.1 Hz, 3H)	+0.01
20	1.69 (s, 3H)	1.71 (s, 3H)	+0.02
21	1.66 (s, 3H)	1.67 (s, 3H)	+0.01
22	1.71 (s, 3H)	1.73 (s, 3H)	+0.02
24	1.97 (s, 3H)	1.98 (s, 3H)	+0.01

[b] Seo, C.; Oh, H. Chejuenolide C: A new macrocyclic metabolite from the marine bacterium Hahella chejuensis. *Bull. Korean. Chem. Soc.* **2009**, *30*, 1181.

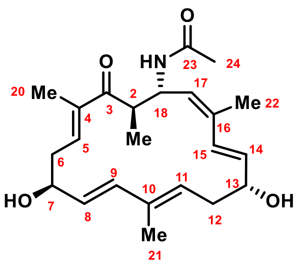

chejuenolide C

Comparison of ^{13}C-NMR of the Reported and Synthetic Chejuenolide C

position	Reported chejuenolide C (Oh et al.[b]) ^{13}C NMR (100 MHz, CD_3OD) δ [ppm, mult, *J* (Hz)]	Synthetic chejuenolide C (this work) ^{13}C NMR (125 MHz, CD_3OD) δ [ppm, mult, *J* (Hz)]	Δδ / ppm
2	44.4	44.4	0
3	205.9	205.8	-0.1
4	138.7	138.6	-0.1
5	141.7	141.7	0
6	36.7	36.7	0
7	70.5	70.4	-0.1
8	129.7	129.6	-0.1
9	135.8	135.7	-0.1
10	136.3	136.3	0
11	128.5	128.5	0
12	37.1	37.0	-0.1
13	75.2	75.1	-0.1
14	134.6	134.5	-0.1
15	128.3	128.2	-0.1
16	135.1	135.0	-0.1
17	128.5	128.4	-0.1
18	50.3	50.3	0
19	14.7	14.7	0
20	11.5	11.4	-0.1
21	13.0	12.9	-0.1
22	20.1	20.1	0
23	172.5	172.4	-0.1
24	22.7	22.7	0

[b]Seo, C.; Oh, H. Chejuenolide C: A new macrocyclic metabolite from the marine bacterium Hahella chejuensis. *Bull. Korean. Chem. Soc.* **2009**, *30*, 1181.

5. X-Ray Crystallographic Information

X-Ray Crystallographic Information for **18a**: CCDC 2205570

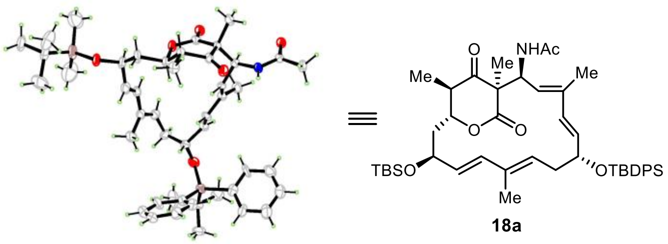

18a

Identification code	mj20390_0m	
Empirical formula	C46 H65 N O6 Si2	
Formula weight	784.17	
Temperature	193.01 K	
Wavelength	1.34139 Å	
Crystal system	Monoclinic	
Space group	P 1 21 1	
Unit cell dimensions	a = 16.4420(14) Å	a= 90°.
	b = 10.9818(8) Å	b= 99.492(4)°.
	c = 18.9303(18) Å	g = 90°.
Volume	3371.3(5) $Å^3$	
Z	2	
Density (calculated)	0.772 Mg/m^3	
Absorption coefficient	0.464 mm^{-1}	
F(000)	848	
Crystal size	0.1 x 0.08 x 0.06 mm^3	
Theta range for data collection	3.387 to 55.169°.	
Index ranges	-20<=h<=19, -6<=k<=13, -23<=l<=23	
Reflections collected	30598	
Independent reflections	9494 [R(int) = 0.1049]	
Completeness to theta = 53.594°	98.7 %	
Absorption correction	Semi-empirical from equivalents	
Max. and min. transmission	0.7508 and 0.4730	
Refinement method	Full-matrix least-squares on F^2	
Data / restraints / parameters	9494 / 48 / 497	
Goodness-of-fit on F^2	0.936	
Final R indices [I>2sigma(I)]	R1 = 0.0763, wR2 = 0.2103	
R indices (all data)	R1 = 0.1204, wR2 = 0.2474	
Absolute structure parameter	0.10(5)	
Extinction coefficient	n/a	
Largest diff. peak and hole	0.257 and -0.295 e.$Å^{-3}$	

X-Ray Crystallographic Information for **chejuenolide A**: CCDC 2205487

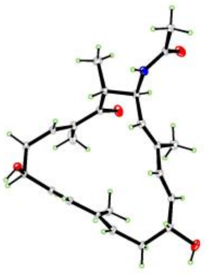

≡

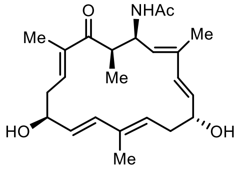

chejuenolide A (**1**)

Identification code	mj20029_0m	
Empirical formula	C23 H37 N O6	
Formula weight	423.53	
Temperature	170.03 K	
Wavelength	1.34139 Å	
Crystal system	Monoclinic	
Space group	P 1 21 1	
Unit cell dimensions	a = 6.23890(10) Å	a= 90°.
	b = 12.9682(3) Å	b= 91.0320(10)°.
	c = 30.8833(6) Å	g = 90°.
Volume	2498.28(9) $Å^3$	
Z	4	
Density (calculated)	1.126 Mg/m^3	
Absorption coefficient	0.421 mm^{-1}	
F(000)	920	
Crystal size	0.12 x 0.06 x 0.006 mm^3	
Theta range for data collection	3.216 to 54.880°.	
Index ranges	-7<=h<=7, -15<=k<=15, -37<=l<=37	
Reflections collected	27046	
Independent reflections	9442 [R(int) = 0.0518]	
Completeness to theta = 53.594°	99.9 %	
Absorption correction	Semi-empirical from equivalents	
Max. and min. transmission	0.7508 and 0.5964	
Refinement method	Full-matrix-block least-squares on F^2	
Data / restraints / parameters	9442 / 1 / 567	
Goodness-of-fit on F^2	0.703	
Final R indices [I>2sigma(I)]	R1 = 0.0576, wR2 = 0.1654	
R indices (all data)	R1 = 0.0699, wR2 = 0.1832	
Absolute structure parameter	0.09(10)	
Extinction coefficient	n/a	
Largest diff. peak and hole	0.877 and -0.418 e.$Å^{-3}$	

X-Ray Crystallographic Information for 2,18-*bisepi*-**chejuenolide A**: CCDC 2205571

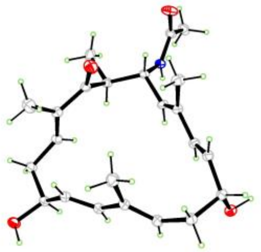

≡

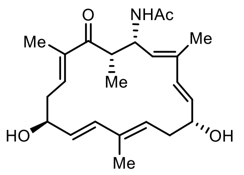

2,18-*bisepi*-**chejuenolide A** (**20**)

Identification code	t
Empirical formula	C23 H33 N O4
Formula weight	387.50
Temperature	297(2) K
Wavelength	1.54178 Å
Crystal system	Orthorhombic
space group	P2(1)2(1)2(1)
Unit cell dimensions	a = 6.1579(6) Å alpha = 90 deg.
	b = 12.7279(11) Å beta = 90 deg.
	c = 31.194(2) Å gamma = 90 deg.
Volume	2444.9(4) A^3
Z	4
Calculated density	1.053 Mg/m^3
Absorption coefficient	0.569 mm^-1
F(000)	840
Crystal size	0.200 x 0.120 x 0.100 mm
Theta range for data collection	2.833 to 65.183 deg.
Limiting indices	-7<=h<=7, -14<=k<=14, -36<=l<=26
Reflections collected / unique	16386 / 4058 [R(int) = 0.0387]
Completeness to theta = 65.183	97.9 %
Refinement method	Full-matrix least-squares on F^2
Data / restraints / parameters	4058 / 0 / 264
Goodness-of-fit on F^2	1.037
Final R indices [I>2sigma(I)]	R1 = 0.0401, wR2 = 0.1047
R indices (all data)	R1 = 0.0493, wR2 = 0.1119
Absolute structure parameter	0.19(11)
Extinction coefficient	n/a
Largest diff. peak and hole	0.224 and -0.157 e.A^-3

X-Ray Crystallographic Information for **30a**: CCDC 2205489

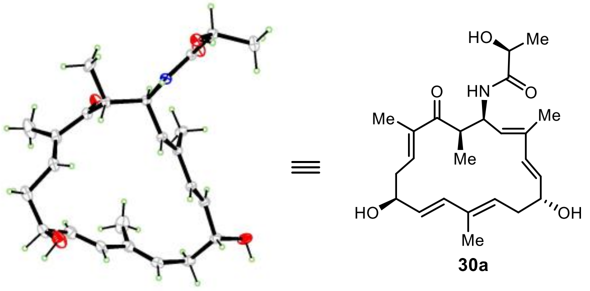

30a

Identification code	a_a
Empirical formula	C24 H35 N O5
Formula weight	417.53
Temperature	297(2) K
Wavelength	1.54178 A
Crystal system	Triclinic
space group	P1
Unit cell dimensions	a = 5.80670(10) Å alpha = 69.1710(10) deg.
	b = 9.8743(2) Å beta = 82.6340(10) deg.
	c = 11.4837(3) Å gamma = 77.5620(10) deg.
Volume	600.00(2) A^3
Z	1
Calculated density	1.156 Mg/m^3
Absorption coefficient	0.646 mm^-1
F(000)	226
Crystal size	0.180 x 0.160 x 0.150 mm
Theta range for data collection	4.125 to 68.112 deg.
Limiting indices	-6<=h<=6, -11<=k<=11, -13<=l<=13
Reflections collected / unique	17002 / 4227 [R(int) = 0.0275]
Completeness to theta = 67.679	99.5 %
Refinement method	Full-matrix least-squares on F^2
Data / restraints / parameters	4227 / 3 / 286
Goodness-of-fit on F^2	1.000
Final R indices [I>2sigma(I)]	R1 = 0.0329, wR2 = 0.0885
R indices (all data)	R1 = 0.0349, wR2 = 0.0912
Absolute structure parameter	0.08(6)
Extinction coefficient	n/a
Largest diff. peak and hole	0.121 and -0.104 e.A^-3

X-Ray Crystallographic Information for **21**: CCDC 2205490

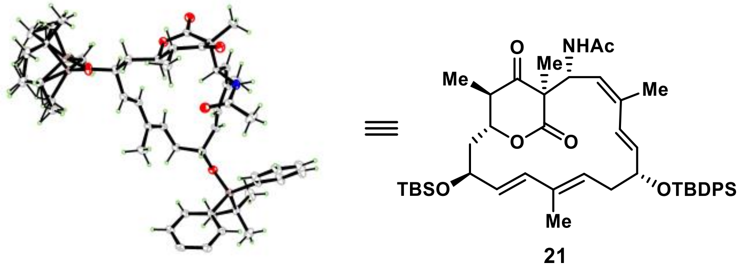

21

Identification code	a_a
Empirical formula	$C_{95}H_{136}N_2O_{13}Si_4$
Formula weight	1626.41
Temperature/K	173.0
Crystal system	monoclinic
Space group	C2
Unit cell dimensions	a = 24.1276(6) Å alpha = 90 deg.
	b = 9.7443(2) Å beta = 100.0630(10) deg.
	c = 41.7915(11) Å gamma = 90 deg.
Volume/Å^3	9674.3(4)
Z	4
ρ_{calc}g/cm^3	1.117
μ/mm^{-1}	1.026
F(000)	3520.0
Crystal size/mm^3	0.26 × 0.1 × 0.06
Radiation	CuKα (λ = 1.54178)
2Θ range for data collection/°	6.444 to 136.544
Index ranges	-28 ≤ h ≤ 28, -11 ≤ k ≤ 11, -50 ≤ l ≤ 46
Reflections collected	85170
Independent reflections	17651 [R_{int} = 0.0588, R_{sigma} = 0.0447]
Data/restraints/parameters	17651/713/1341
Goodness-of-fit on F^2	1.062
Final R indexes [I>=2σ (I)]	R_1 = 0.0499, wR_2 = 0.1369
Final R indexes [all data]	R_1 = 0.0573, wR_2 = 0.1423
Largest diff. peak/hole / e Å^{-3}	0.90/-0.40
Flack parameter	0.055(10

6. ^{1}H and ^{13}C NMR Spectra of Compounds

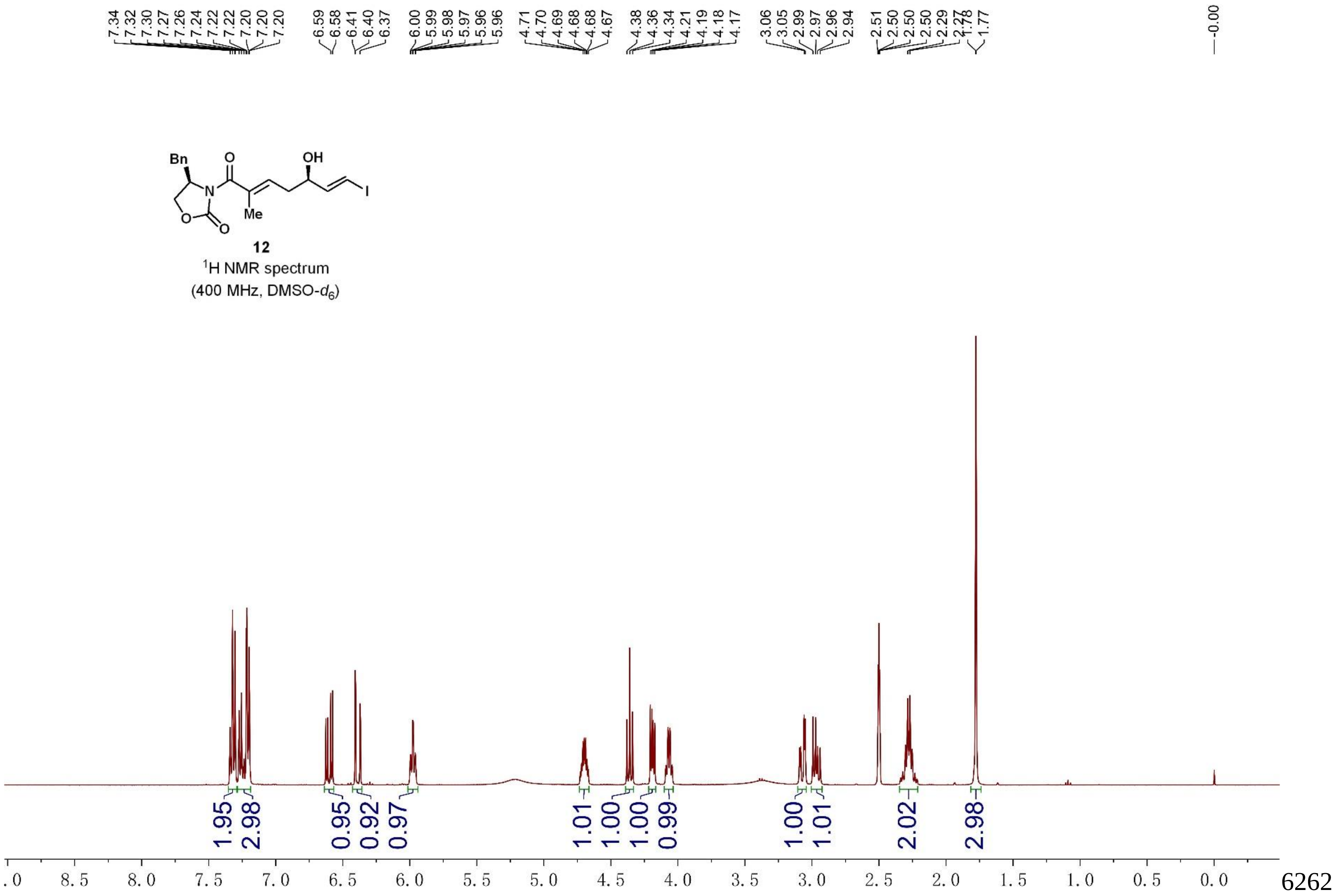

12
¹H NMR spectrum
(400 MHz, DMSO-d6)

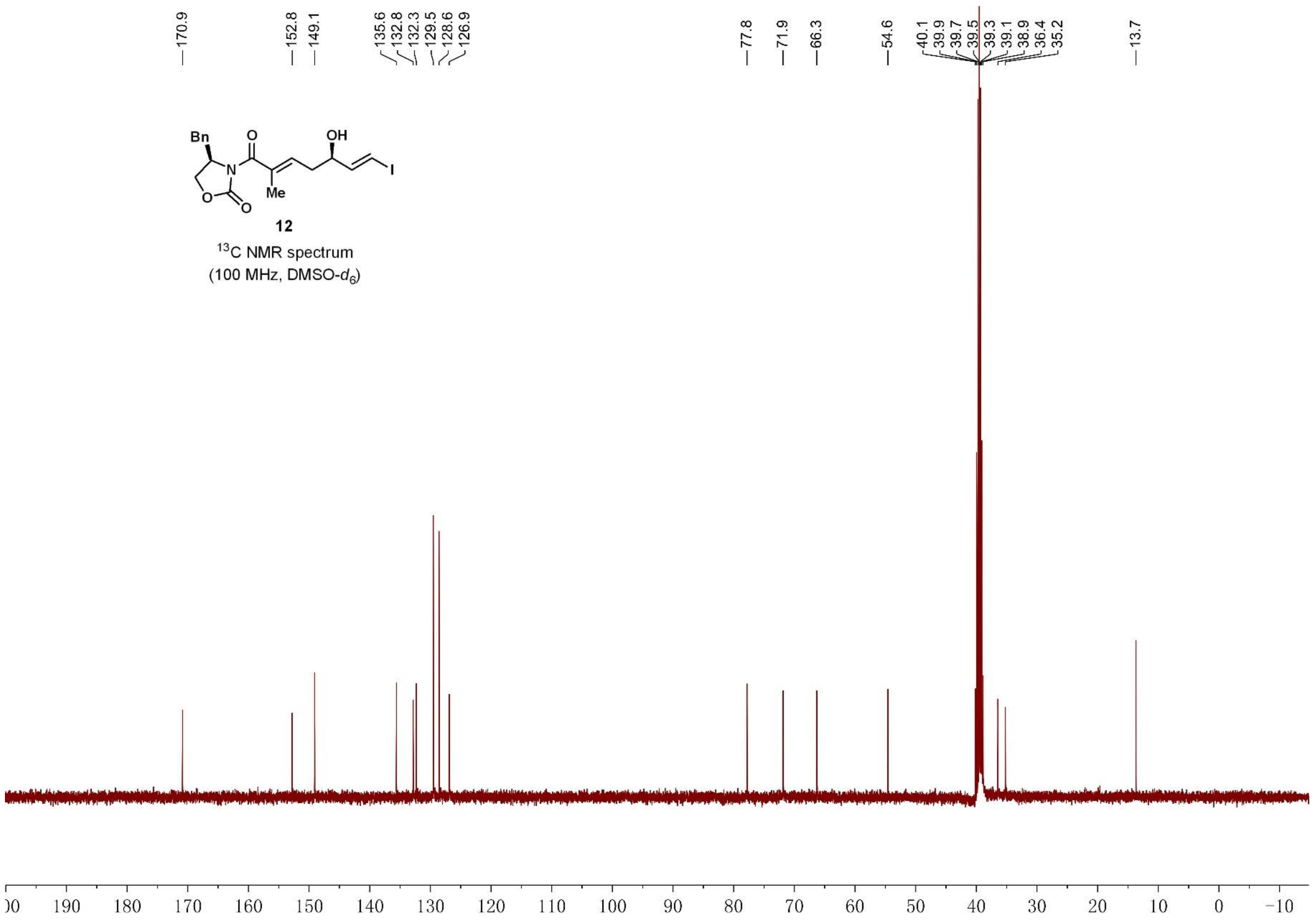

12

^{13}C NMR spectrum
(100 MHz, DMSO-d_6)

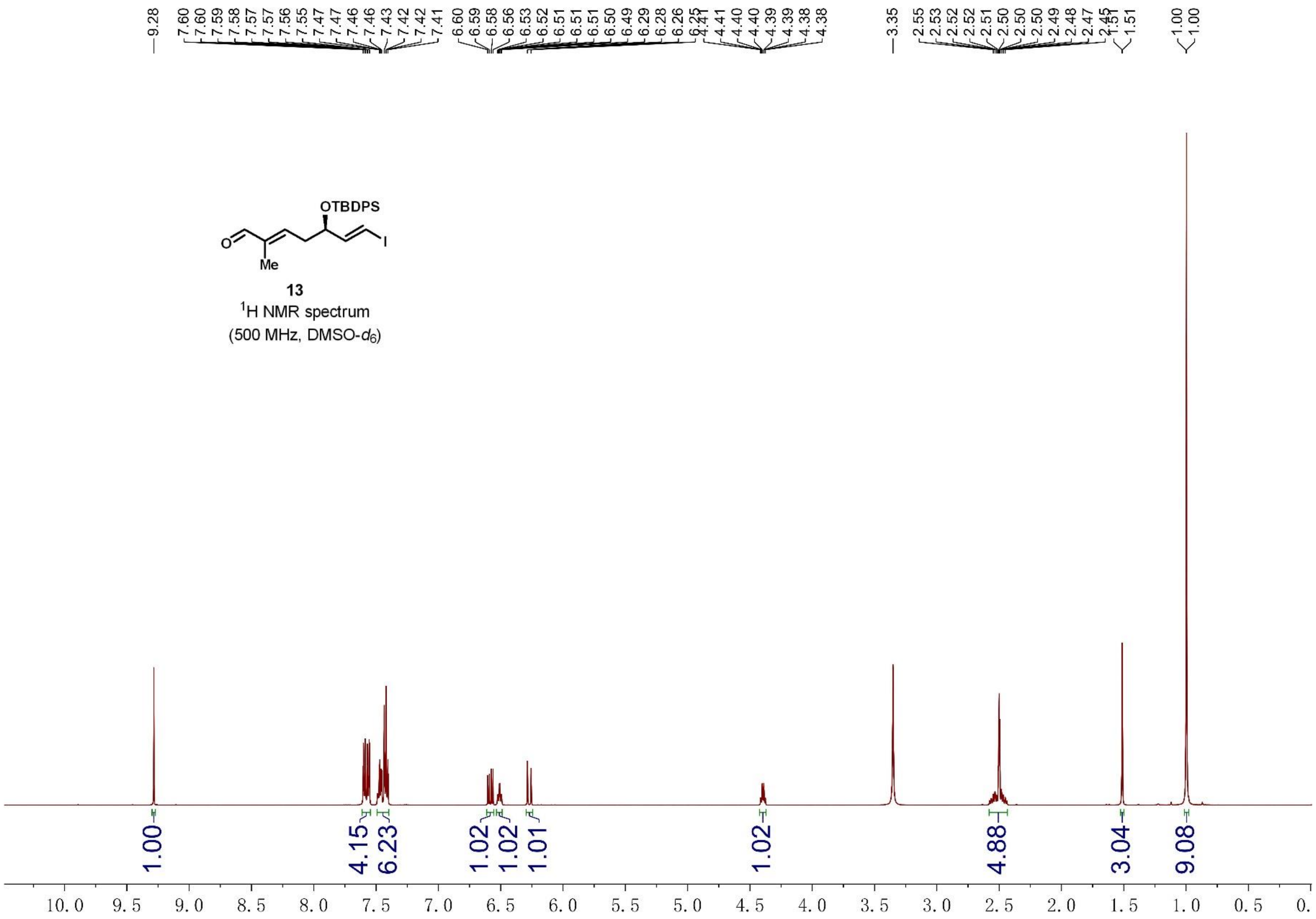

13
^1H NMR spectrum
(500 MHz, DMSO-d_6)

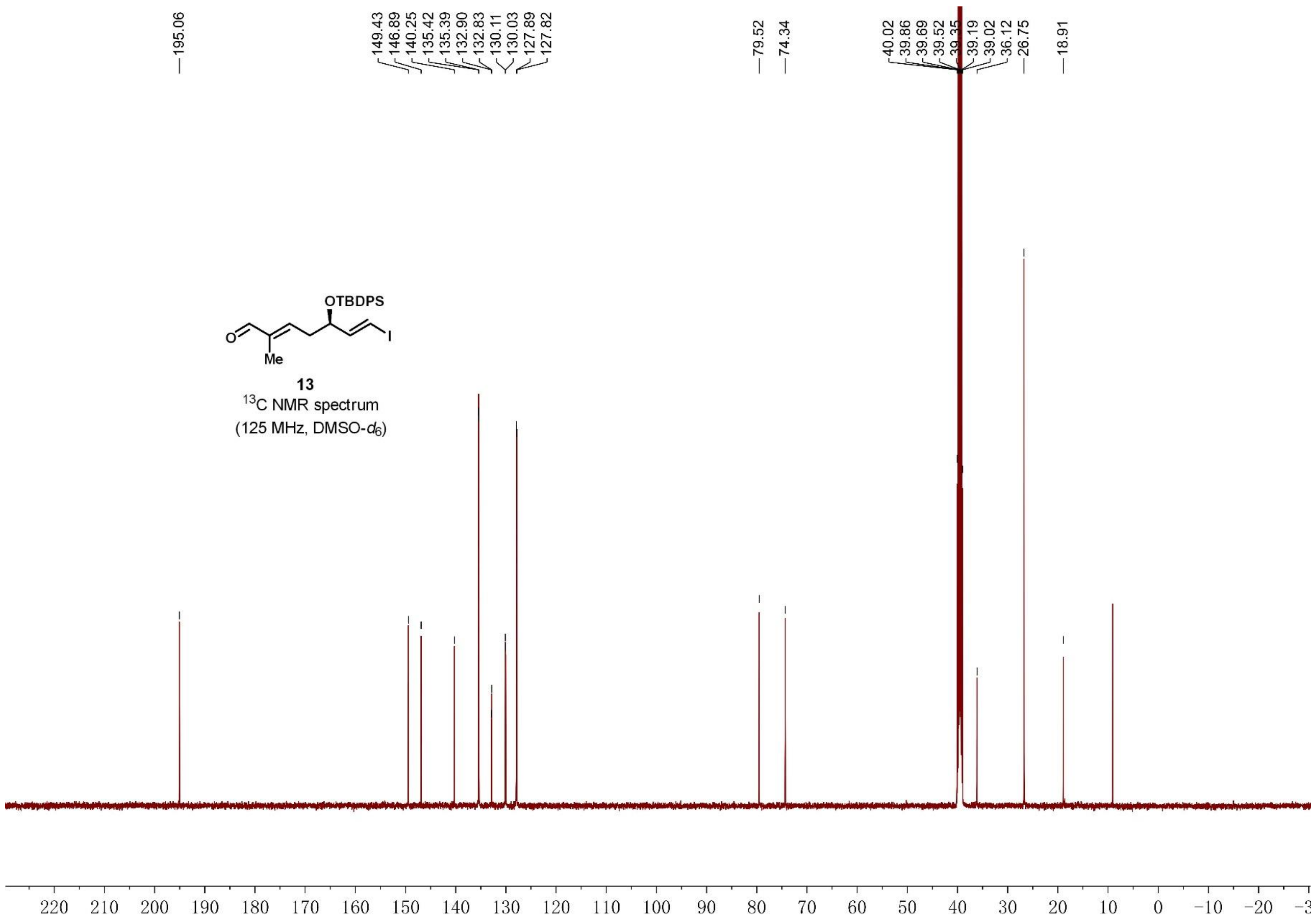
195.06
149.43
146.89
140.25
135.42
135.39
132.90
132.83
130.11
130.03
127.89
127.82
79.52
74.34
40.02
39.86
39.69
39.52
39.35
39.19
39.02
36.12
26.75
18.91
OTBDPS
O
I
Me
13
^{13}C NMR spectrum
(125 MHz, DMSO-d_6)
220 210 200 190 180 170 160 150 140 130 120 110 100 90 80 70 60 50 40 30 20 10 0 −10 −20

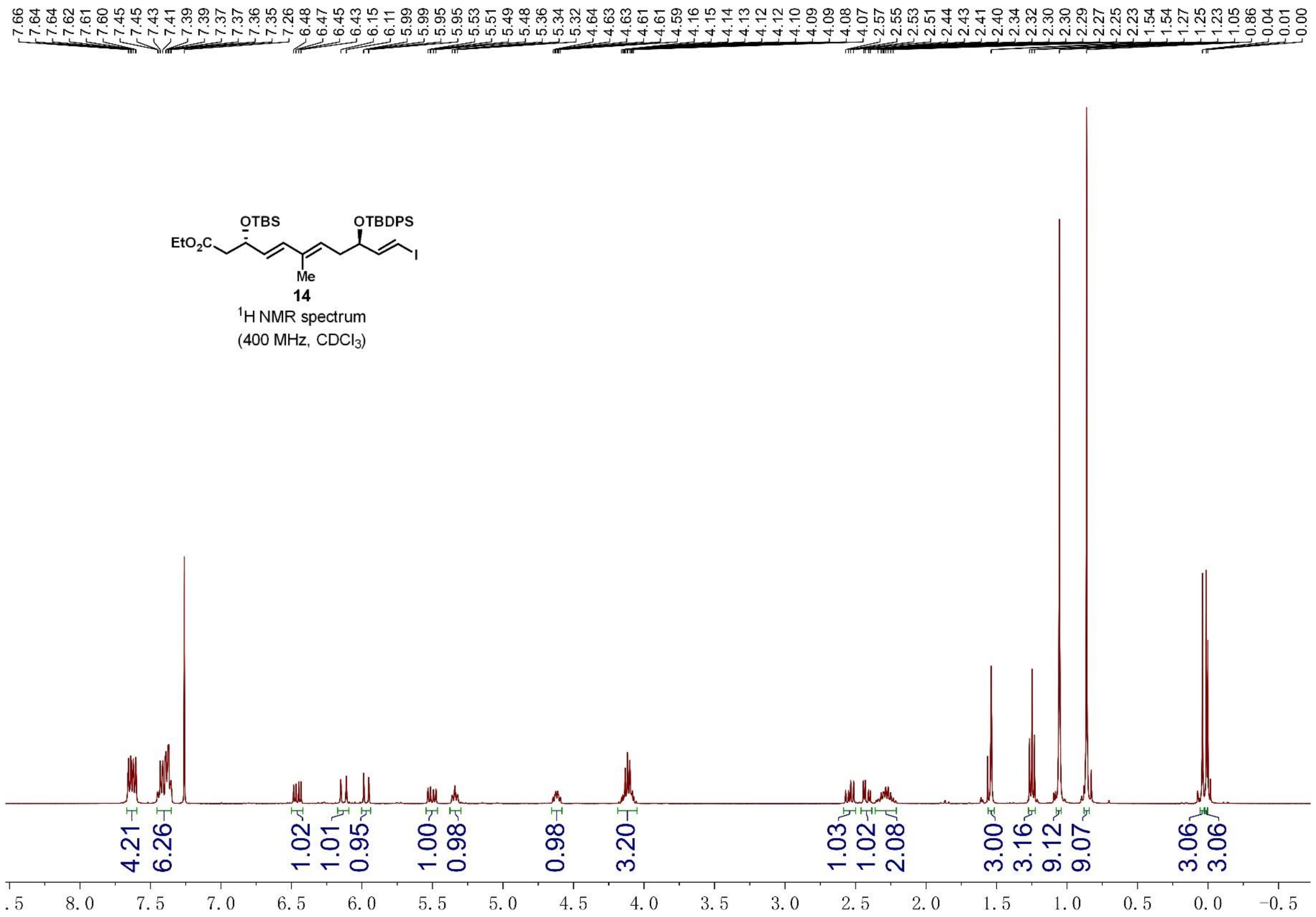
OTBS
OTBDPS
EtO2C
Me
I
14
1H NMR spectrum
(400 MHz, CDCl3)
4.21
6.26
1.02
1.01
0.95
1.00
0.98
0.98
3.20
1.03
1.02
2.08
3.00
3.16
9.12
9.07
3.06
3.06
8.0
7.5
7.0
6.5
6.0
5.5
5.0
4.5
4.0
3.5
3.0
2.5
2.0
1.5
1.0
0.5
0.0
-0.5

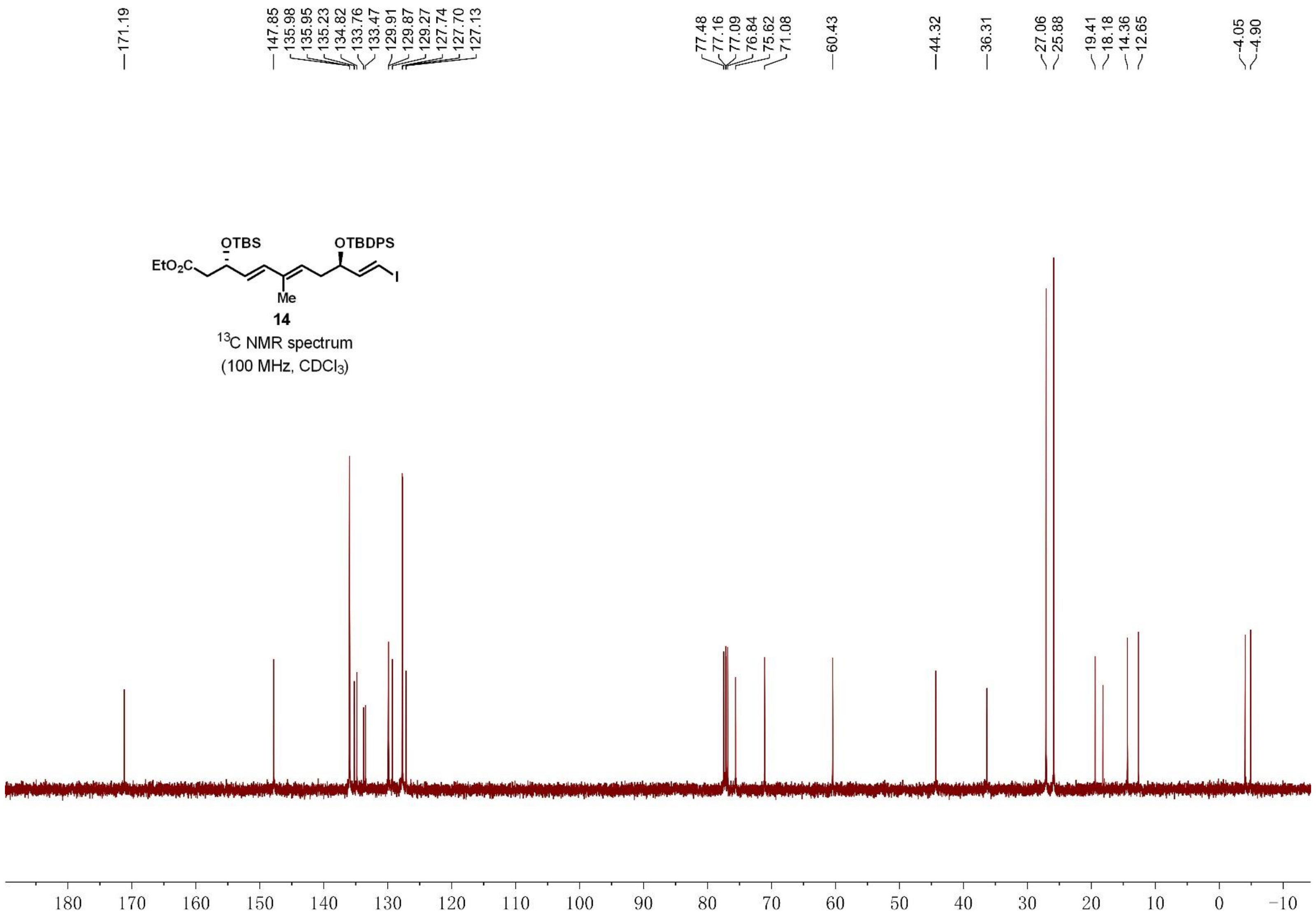
171.19
147.85
135.98
135.95
135.23
134.82
133.76
133.47
129.91
129.87
129.27
127.74
127.70
127.13
77.48
77.16
77.09
76.84
75.62
71.08
60.43
44.32
36.31
27.06
25.88
19.41
18.18
14.36
12.65
-4.05
-4.90
OTBS
OTBDPS
EtO_2C
I
Me
14
^{13}C NMR spectrum
(100 MHz, $CDCl_3$)
180 170 160 150 140 130 120 110 100 90 80 70 60 50 40 30 20 10 0 −10

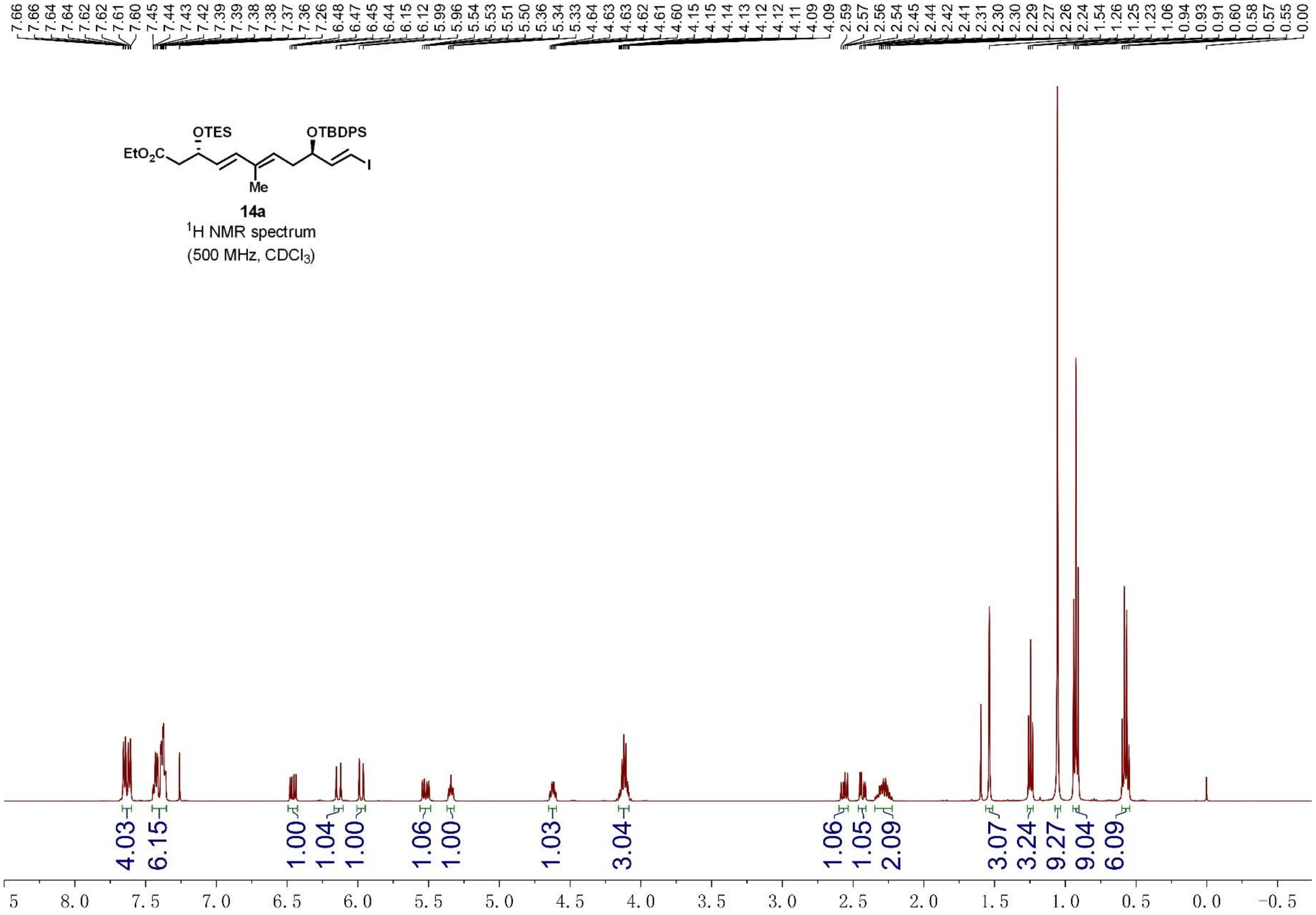

7.66 7.66 7.64 7.64 7.62 7.62 7.61 7.60 7.45 7.44 7.43 7.42 7.39 7.39 7.38 7.38 7.37 7.36 7.26 6.48 6.47 6.45 6.44 6.15 6.12 5.99 5.96 5.54 5.53 5.51 5.50 5.36 5.34 5.33 4.64 4.63 4.63 4.62 4.61 4.60 4.15 4.15 4.14 4.13 4.12 4.12 4.11 4.09 2.59 2.57 2.56 2.54 2.45 2.44 2.42 2.41 2.31 2.30 2.30 2.29 2.27 2.26 2.24 1.54 1.54 1.26 1.25 1.23 1.06 0.94 0.93 0.91 0.60 0.58 0.57 0.55 0.00
OTES
OTBDPS
EtO_2C
I
Me
14a
^1H NMR spectrum
(500 MHz, $CDCl_3$)
4.03 6.15 1.00 1.04 1.00 1.06 1.00 1.03 3.04 1.06 1.05 2.09 3.07 3.24 9.27 9.04 6.09
5 8.0 7.5 7.0 6.5 6.0 5.5 5.0 4.5 4.0 3.5 3.0 2.5 2.0 1.5 1.0 0.5 0.0 −0.5

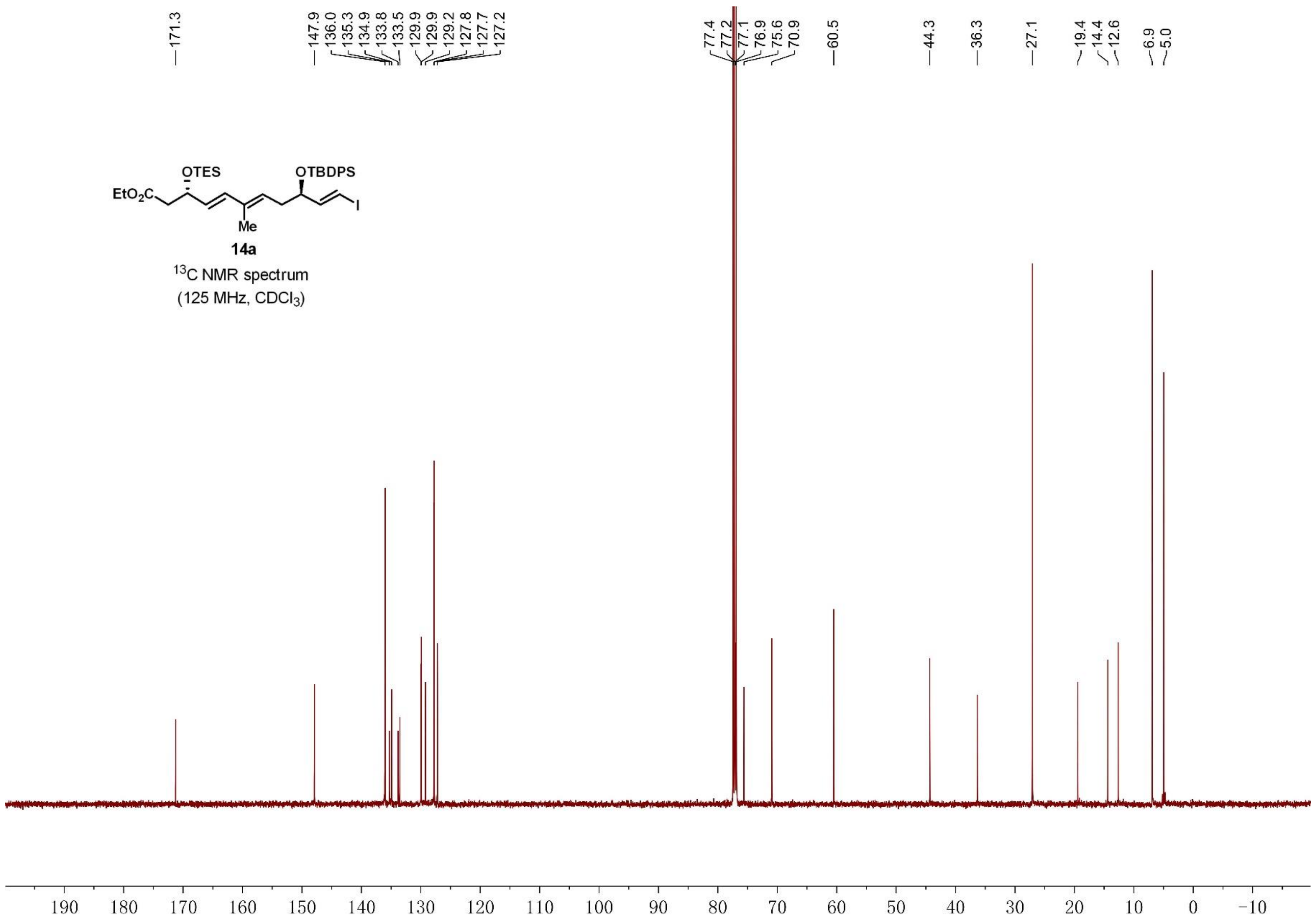
171.3
147.9
136.0
135.3
134.9
133.8
133.5
129.9
129.9
129.2
127.8
127.7
127.2
77.4
77.2
77.1
76.9
75.6
70.9
60.5
44.3
36.3
27.1
19.4
14.4
12.6
6.9
5.0
OTES
OTBDPS
EtO_2C
Me
I
14a
^{13}C NMR spectrum
(125 MHz, $CDCl_3$)
190 180 170 160 150 140 130 120 110 100 90 80 70 60 50 40 30 20 10 0 −10

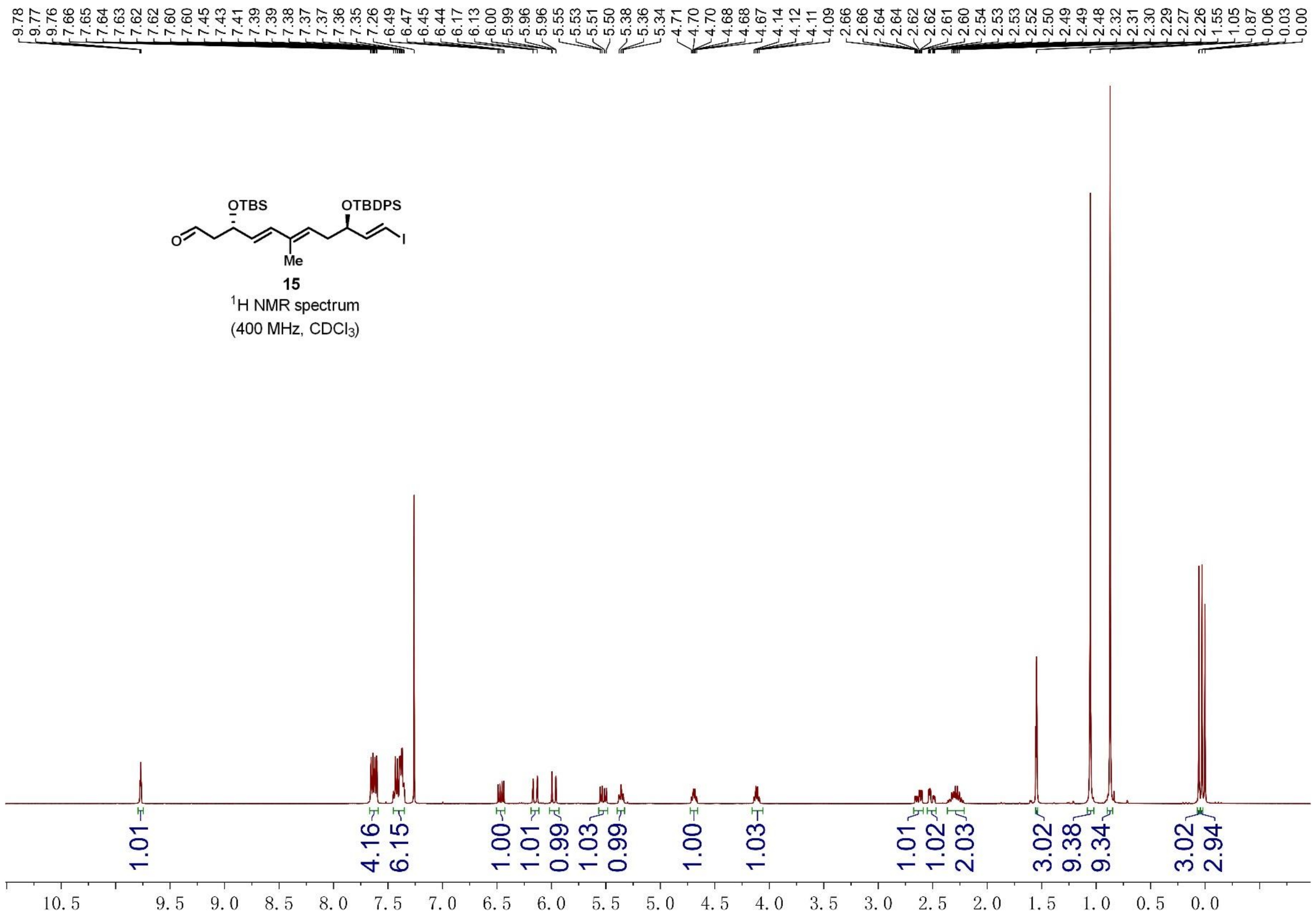

15
^1H NMR spectrum
(400 MHz, $CDCl_3$)

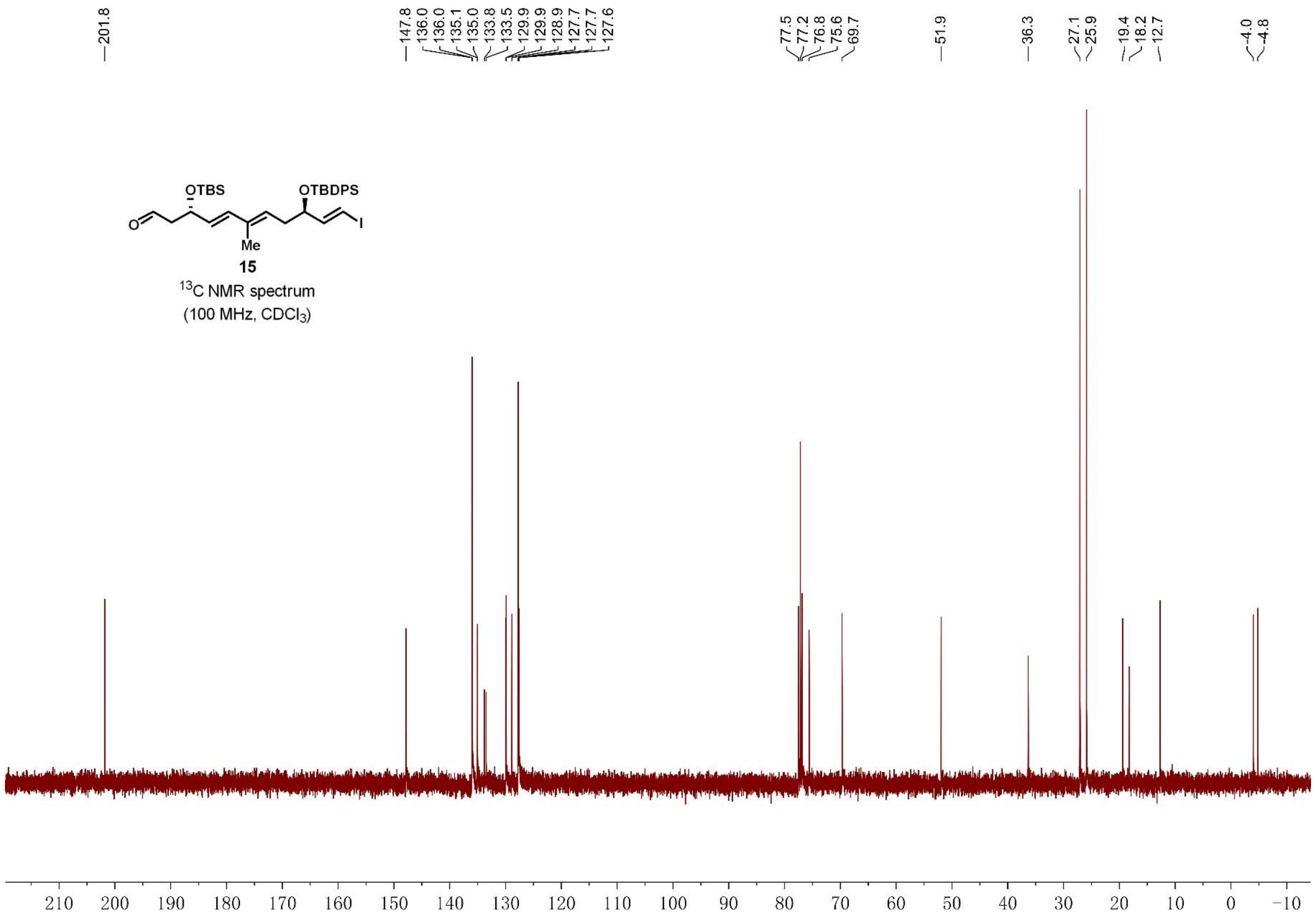
201.8
147.8
136.0
136.0
135.1
135.0
133.8
133.5
129.9
129.9
128.9
127.7
127.7
127.6
77.5
77.2
76.8
75.6
69.7
51.9
36.3
27.1
25.9
19.4
18.2
12.7
-4.0
-4.8
OTBS
OTBDPS
O
I
Me
15
^{13}C NMR spectrum
(100 MHz, CDCl$_3$)
210
200
190
180
170
160
150
140
130
120
110
100
90
80
70
60
50
40
30
20
10
0
−10

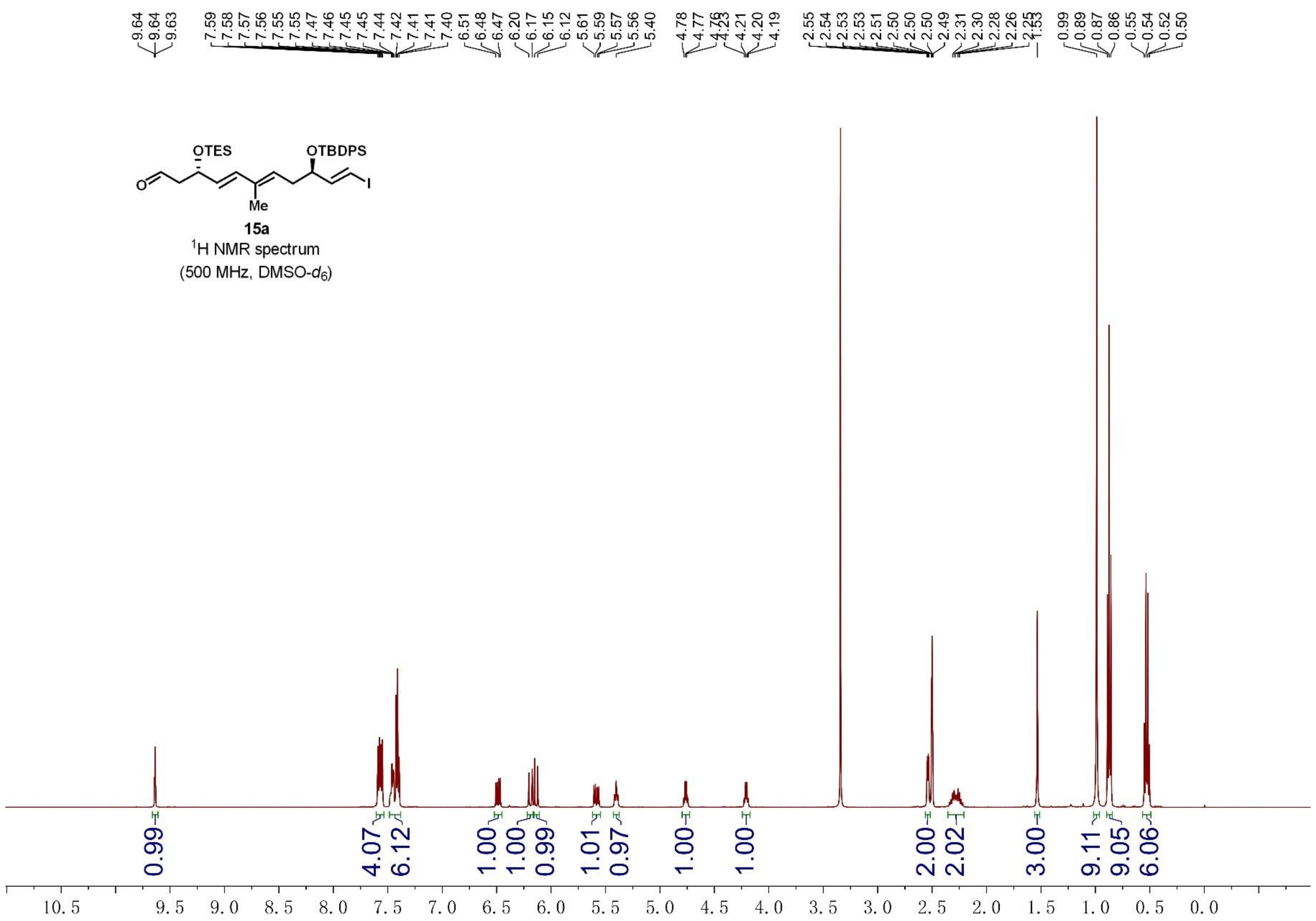
OTES
OTBDPS
Me
15a
^{1}H NMR spectrum
(500 MHz, DMSO-d_6)
10.5 9.5 9.0 8.5 8.0 7.5 7.0 6.5 6.0 5.5 5.0 4.5 4.0 3.5 3.0 2.5 2.0 1.5 1.0 0.5 0.0

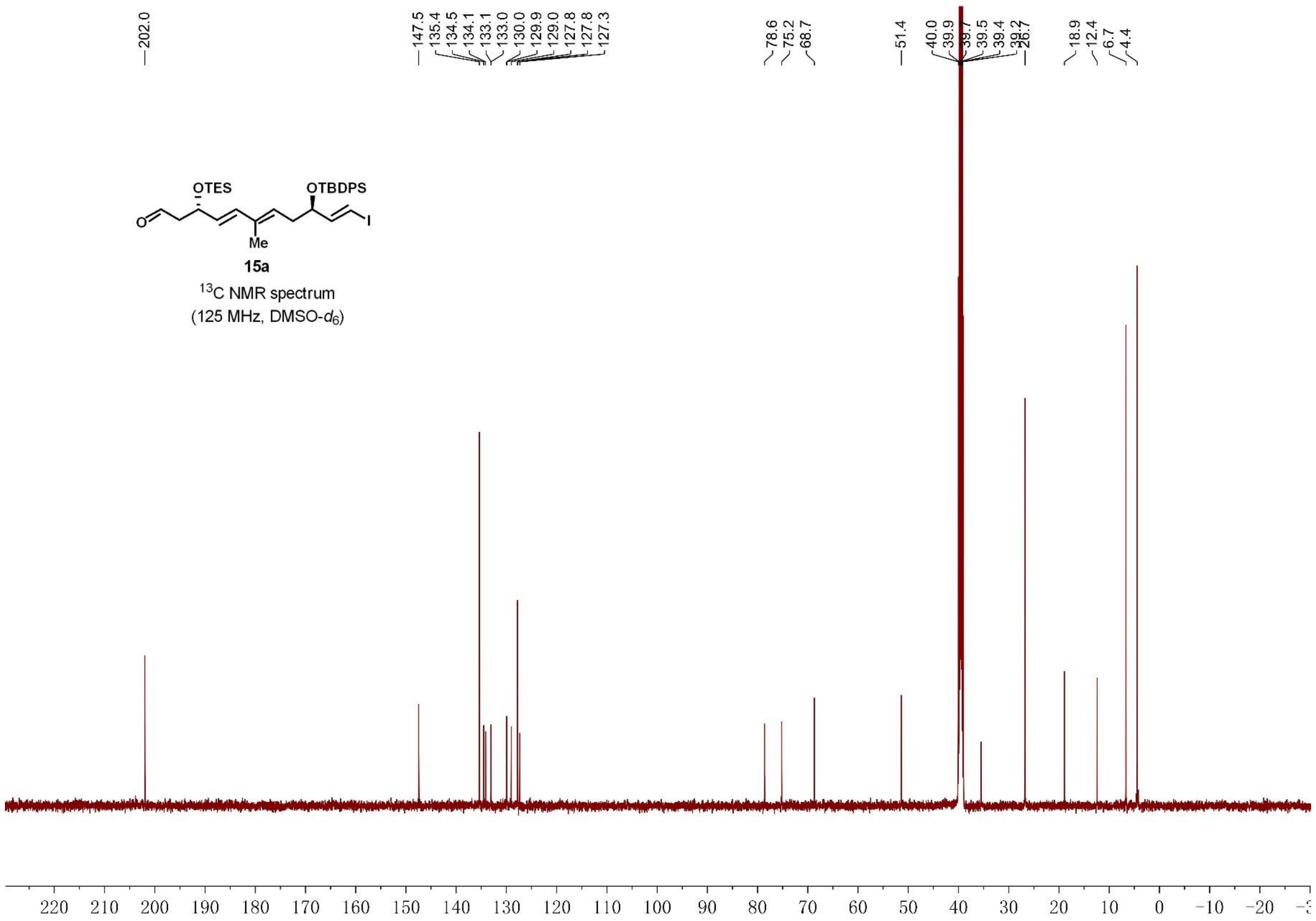
OTES
OTBDPS
O
I
Me
15a
^{13}C NMR spectrum
(125 MHz, DMSO-d_6)
202.0
147.5
135.4
134.5
134.1
133.1
133.0
130.0
129.9
129.0
127.8
127.8
127.3
78.6
75.2
68.7
51.4
40.0
39.9
39.7
39.5
39.4
39.2
26.7
18.9
12.4
6.7
4.4
220 210 200 190 180 170 160 150 140 130 120 110 100 90 80 70 60 50 40 30 20 10 0 −10 −20

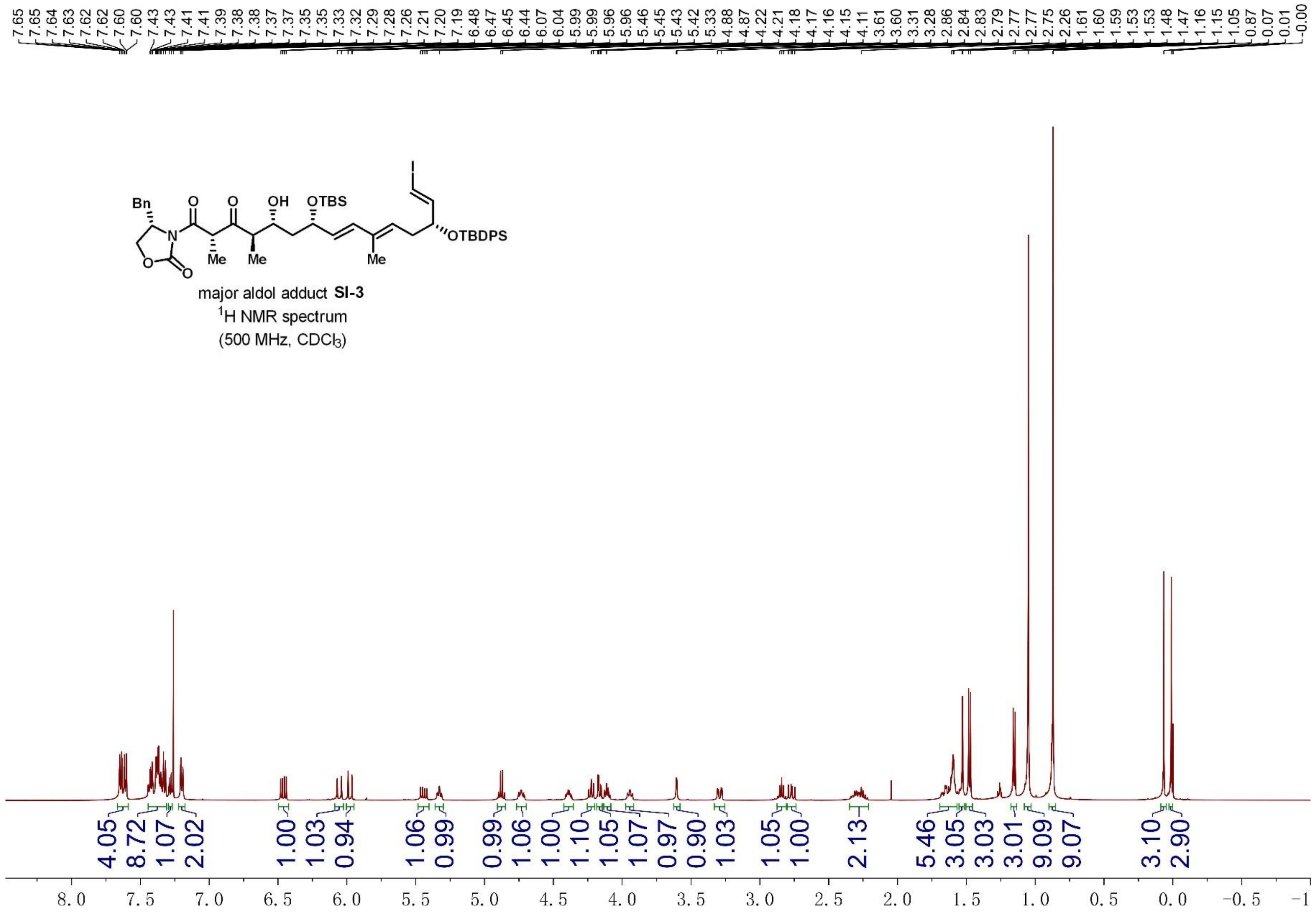
Bn
OH
OTBS
OTBDPS
Me
Me
Me
major aldol adduct **SI-3**
^{1}H NMR spectrum
(500 MHz, $CDCl_3$)

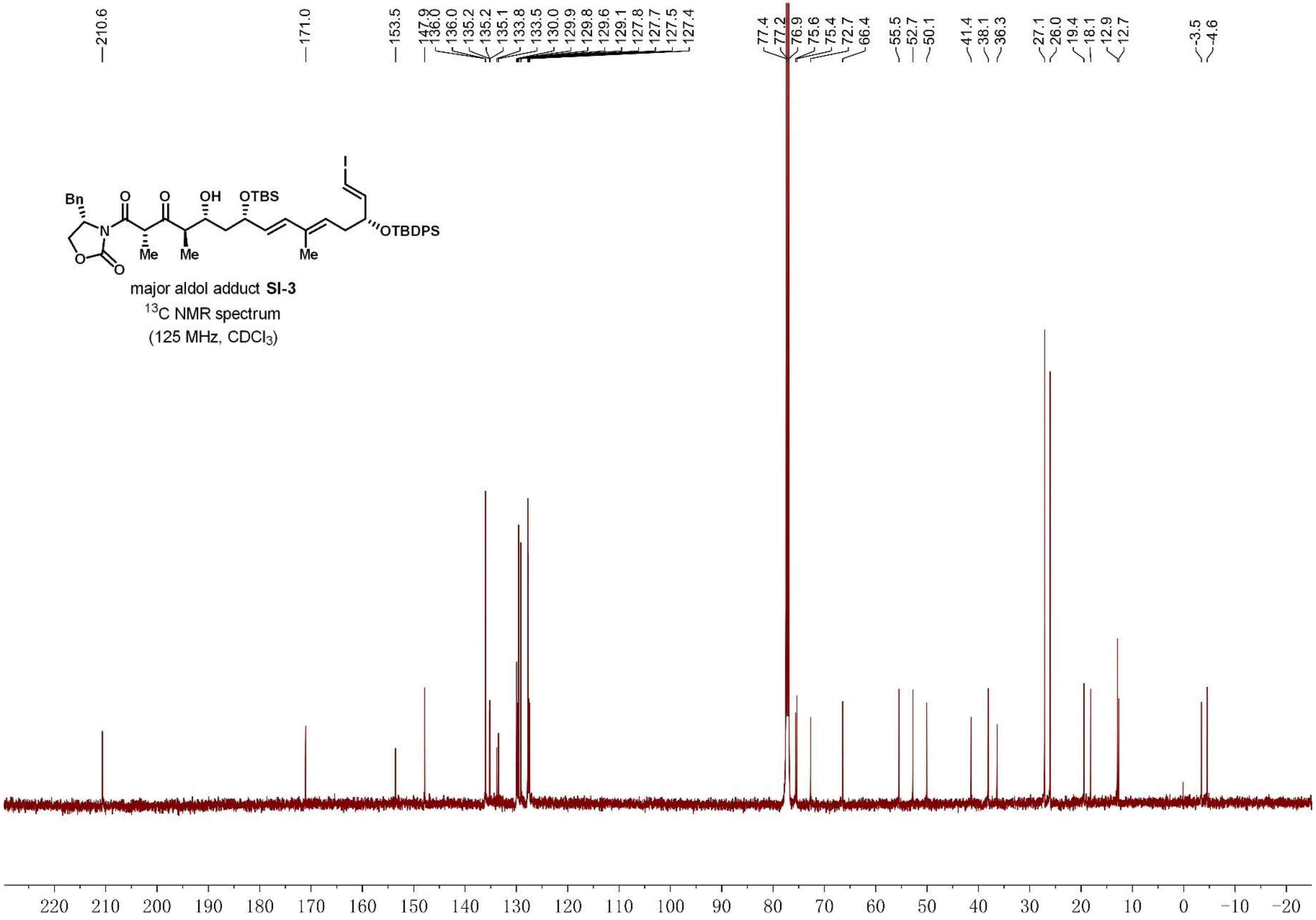

major aldol adduct **SI-3**
^{13}C NMR spectrum
(125 MHz, $CDCl_3$)

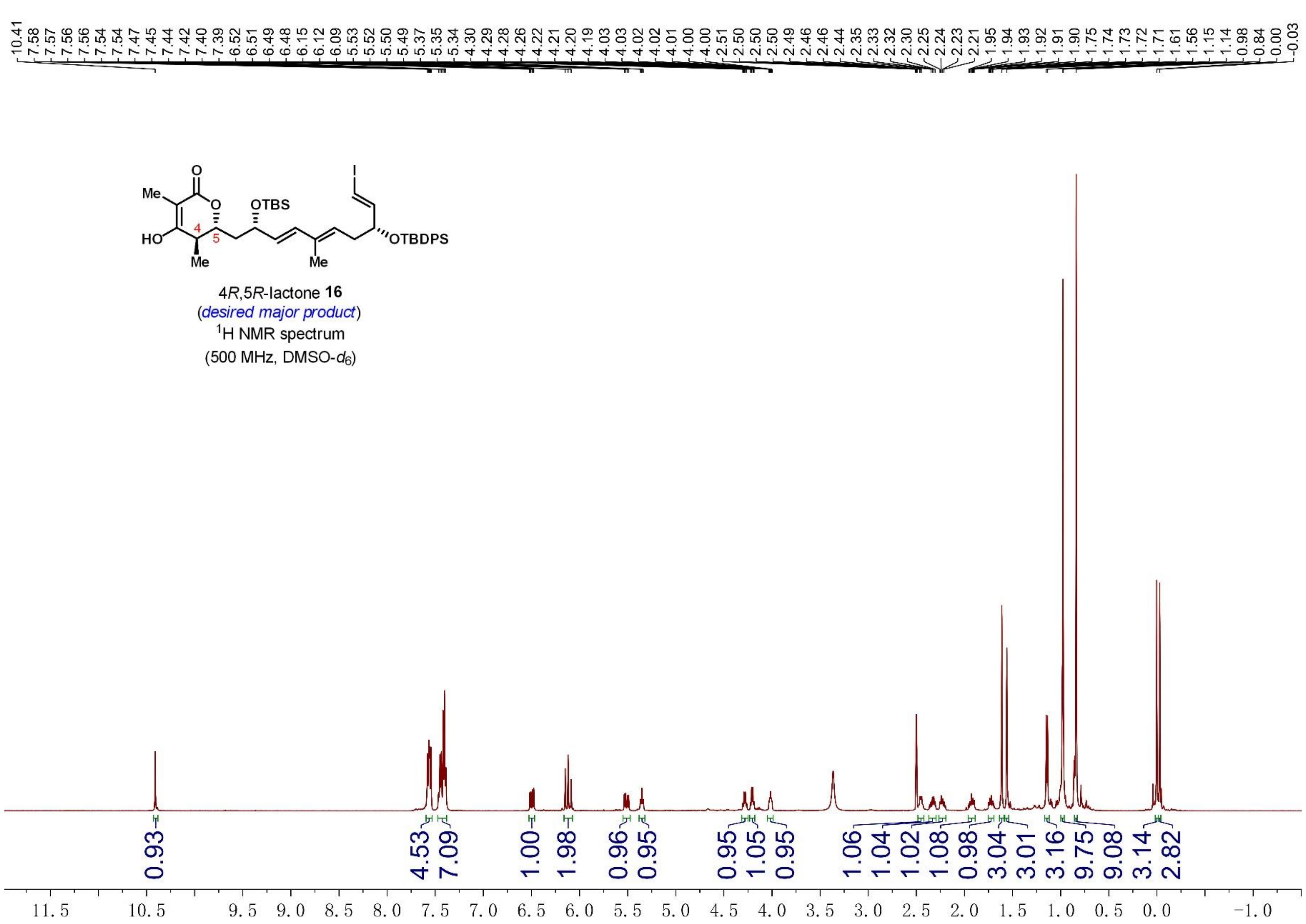

4*R*,5*R*-lactone **16**
(*desired major product*)
^{1}H NMR spectrum
(500 MHz, DMSO-d_6)

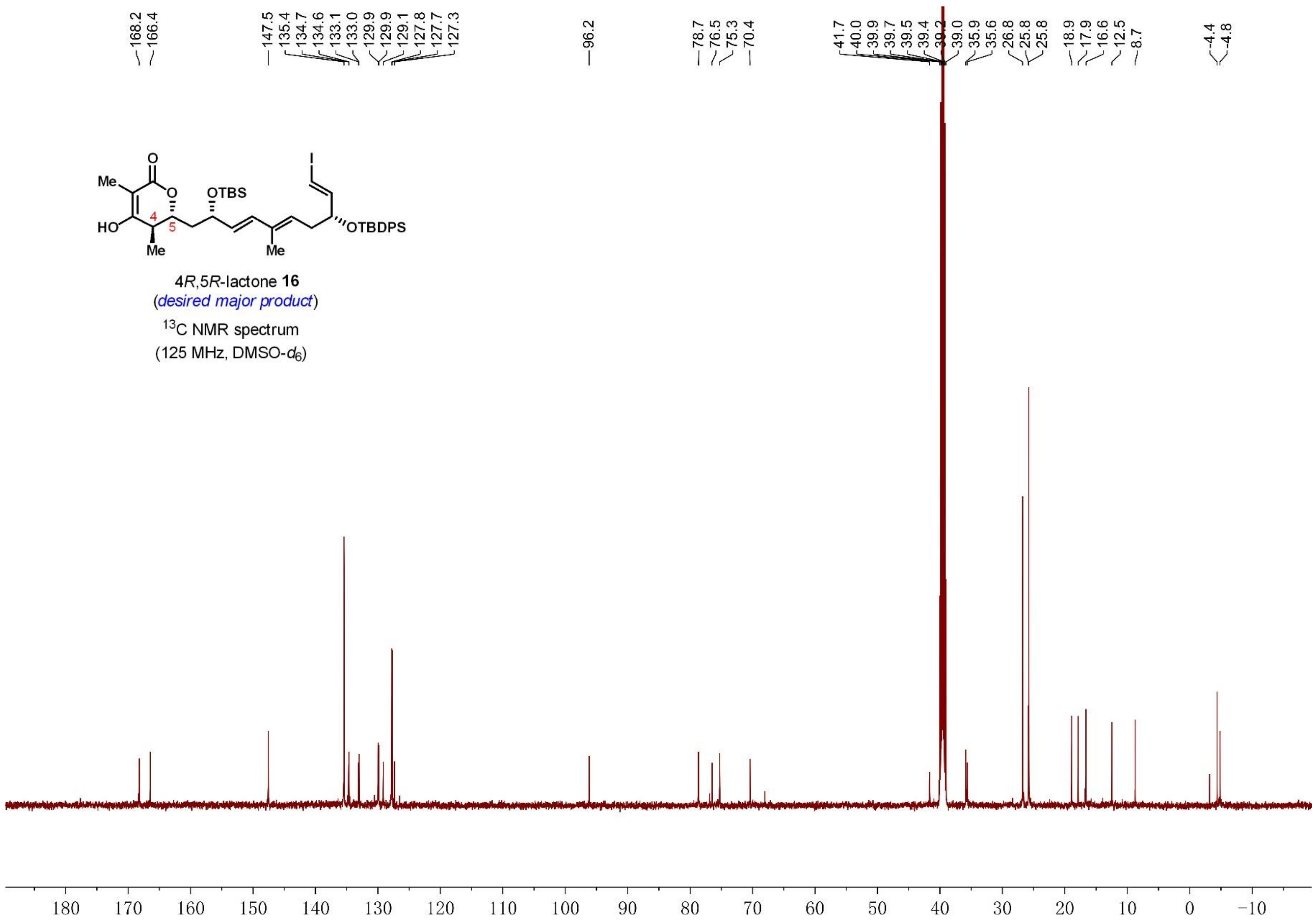

4*R*,5*R*-lactone **16**
(*desired major product*)
^{13}C NMR spectrum
(125 MHz, DMSO-d_6)

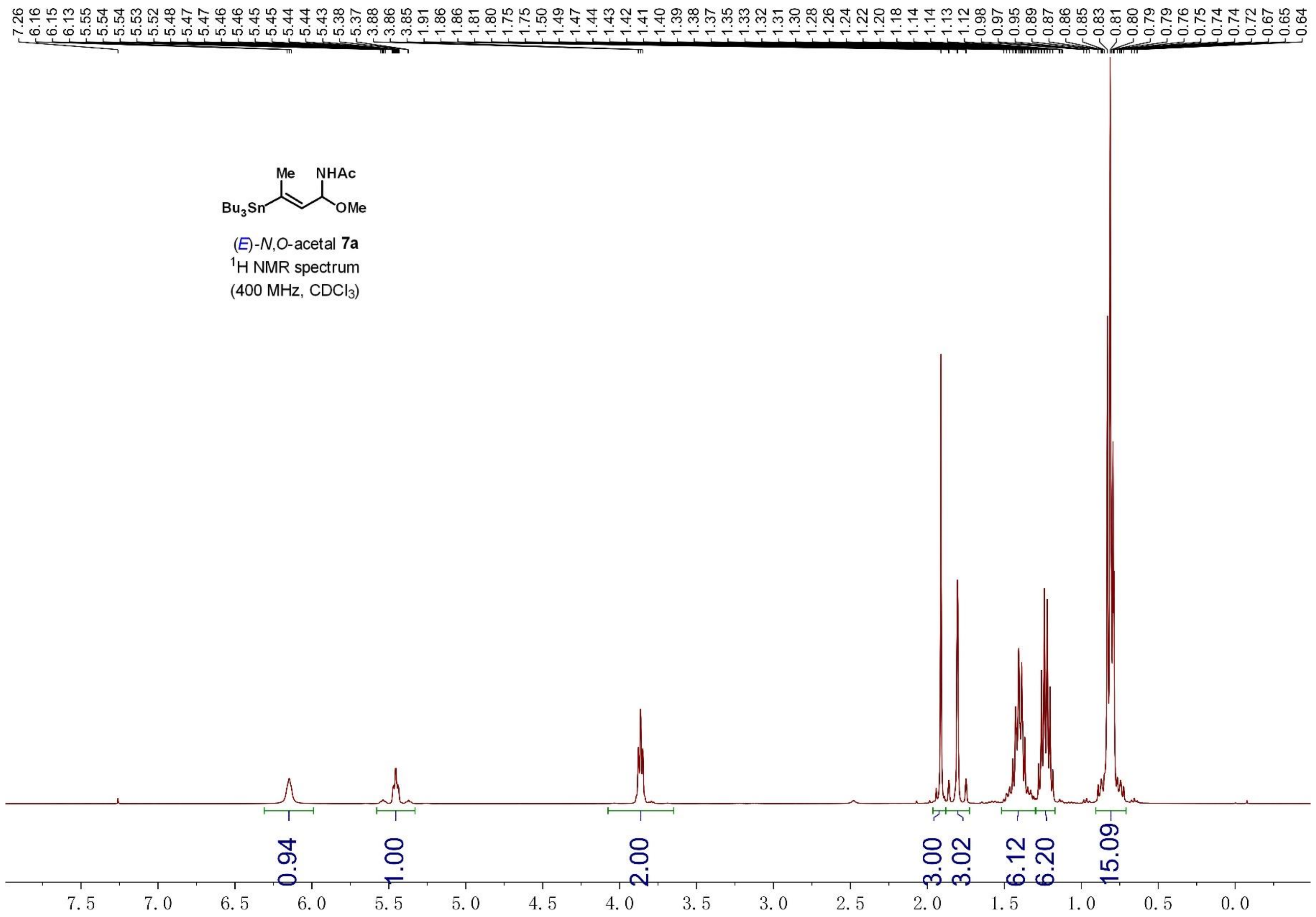
Me
NHAc
Bu3Sn
OMe
(E)-N,O-acetal 7a
1H NMR spectrum
(400 MHz, CDCl3)
0.94
1.00
2.00
3.00
3.02
6.12
6.20
15.09

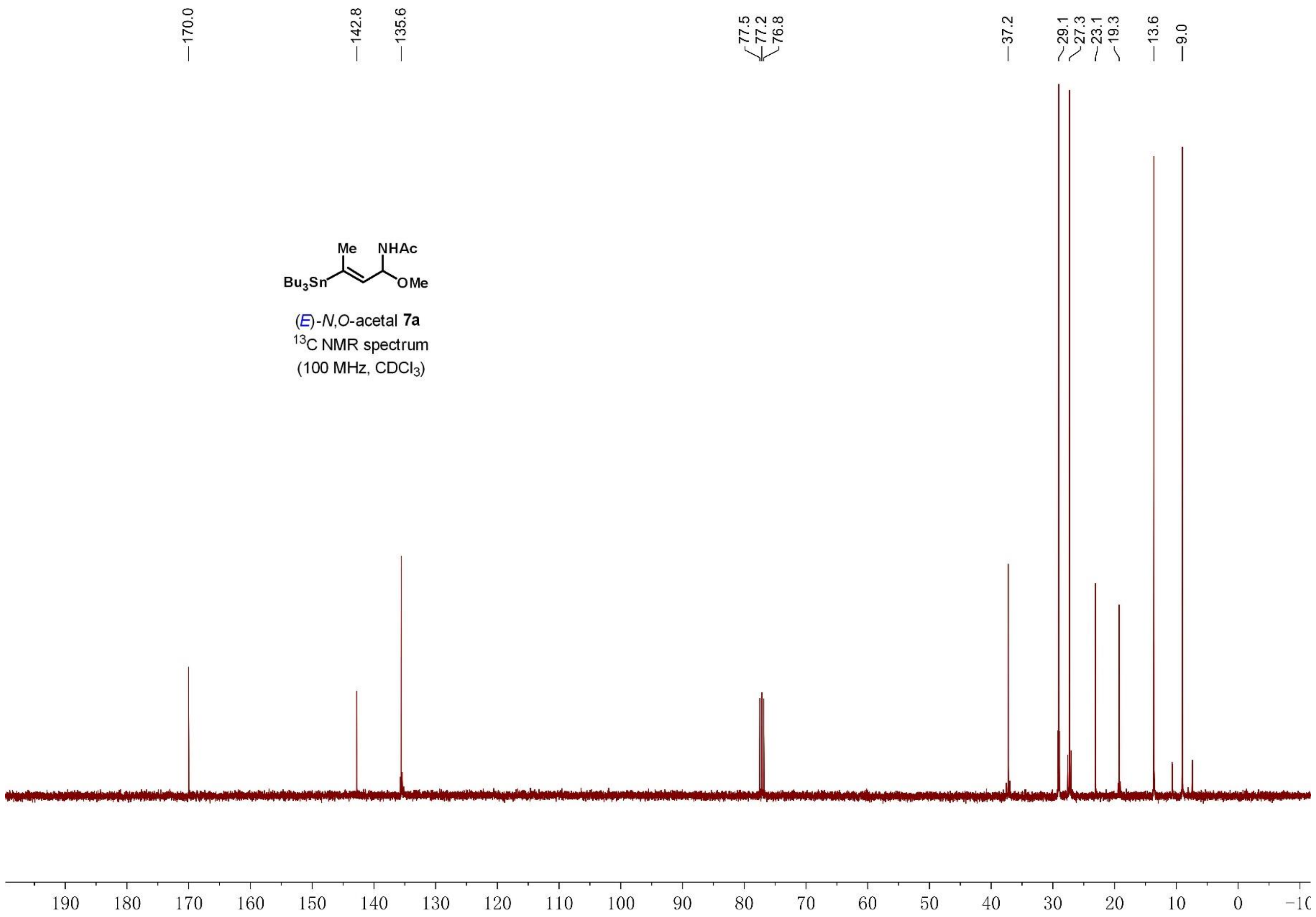
170.0
142.8
135.6
77.5
77.2
76.8
37.2
29.1
27.3
23.1
19.3
13.6
9.0
Me
NHAc
Bu3Sn
OMe
(E)-N,O-acetal 7a
13C NMR spectrum
(100 MHz, CDCl3)
190
180
170
160
150
140
130
120
110
100
90
80
70
60
50
40
30
20
10
0
−10

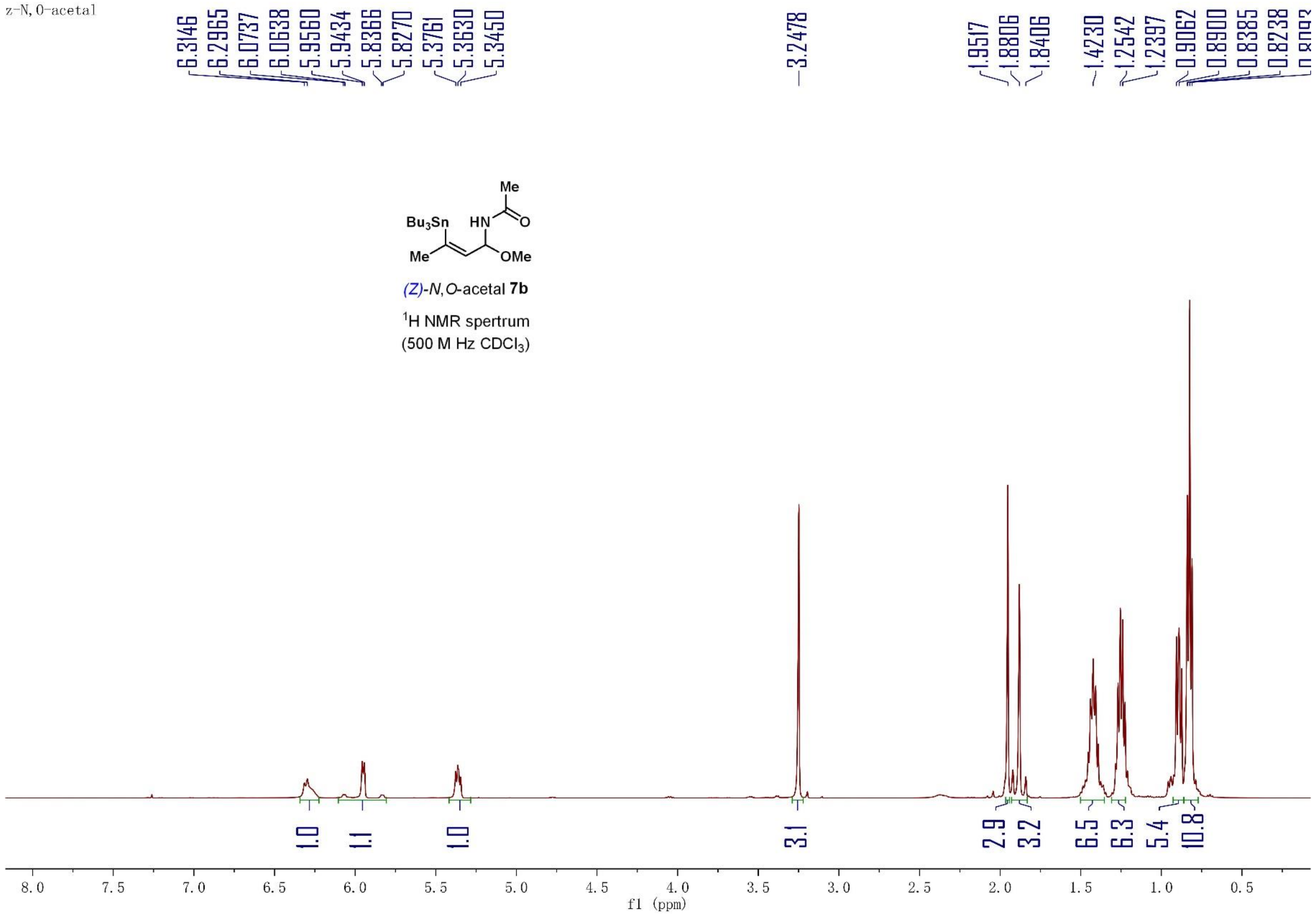
z-N, O-acetal
6.3146
6.2965
6.0737
6.0638
5.9560
5.9434
5.8366
5.8270
5.3761
5.3630
5.3450
3.2478
1.9517
1.8806
1.8406
1.4230
1.2542
1.2397
0.9062
0.8900
0.8385
0.8238
Me
Bu3Sn
HN
O
Me
OMe
(Z)-N,O-acetal 7b
1H NMR spertrum
(500 M Hz CDCl3)
1.0
1.1
1.0
3.1
2.9
3.2
6.5
6.3
5.4
10.8
8.0
7.5
7.0
6.5
6.0
5.5
5.0
4.5
4.0
3.5
3.0
2.5
2.0
1.5
1.0
0.5
f1 (ppm)

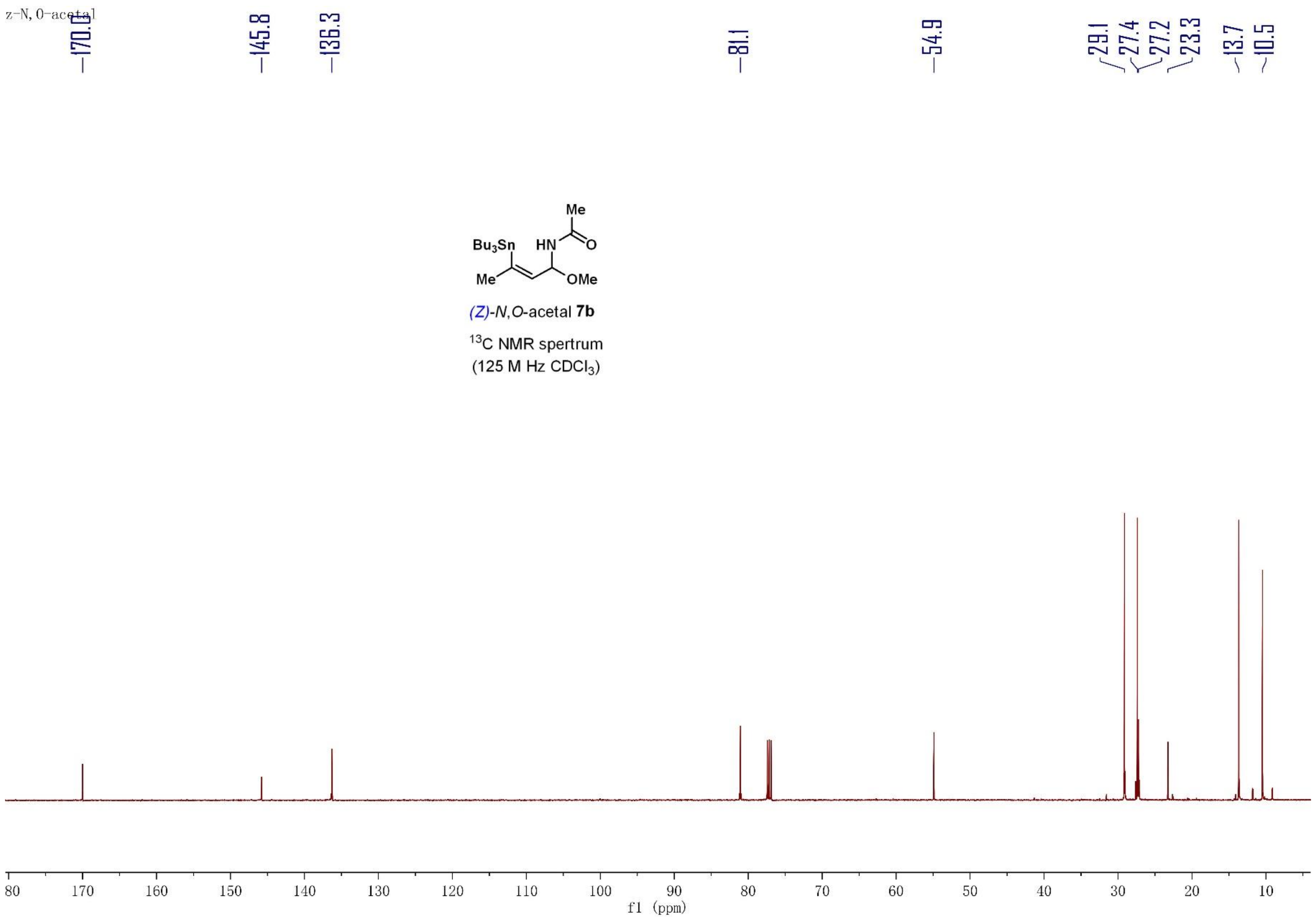
z-N,O-acetal
170.0
145.8
136.3
81.1
54.9
29.1
27.4
27.2
23.3
13.7
10.5
Me
Bu3Sn
HN
O
Me
OMe
(Z)-N,O-acetal 7b
13C NMR spertrum
(125 M Hz CDCl3)
80
170
160
150
140
130
120
110
100
90
80
70
60
50
40
30
20
10
f1 (ppm)

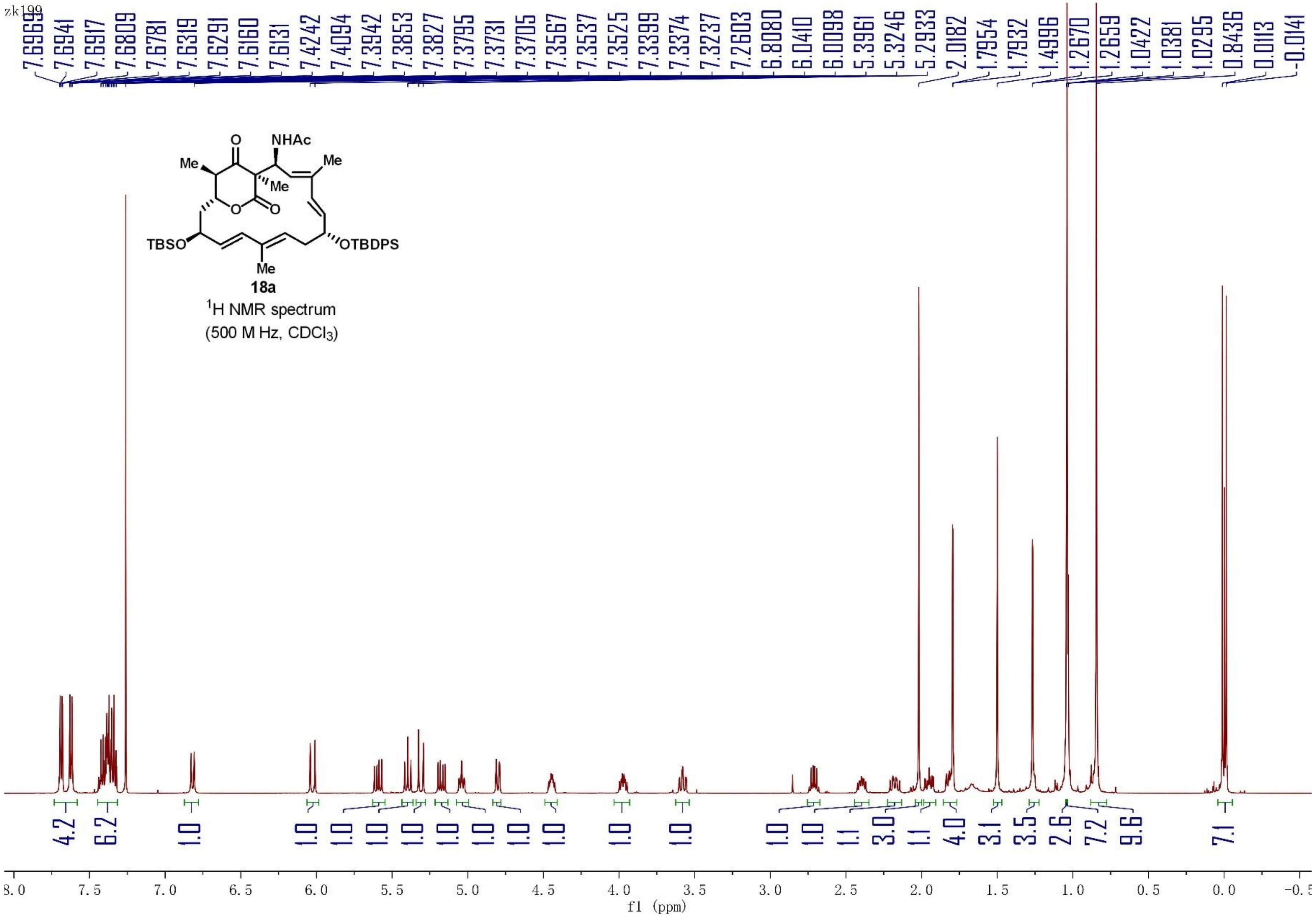
zk199
7.6969
7.6941
7.6917
7.6809
7.6781
7.6319
7.6291
7.6160
7.6131
7.4242
7.4094
7.3942
7.3853
7.3827
7.3795
7.3731
7.3705
7.3567
7.3537
7.3525
7.3399
7.3374
7.3237
7.2603
6.8080
6.0410
6.0098
5.3961
5.3246
5.2933
2.0182
1.7954
1.7932
1.4996
1.2670
1.2659
1.0422
1.0381
1.0295
0.8436
0.0113
-0.0141
NHAc
Me
Me
O
TBSO
OTBDPS
18a
^{1}H NMR spectrum
(500 M Hz, CDCl$_3$)
4.2
6.2
1.0
1.0
1.0
1.0
1.0
1.0
1.0
1.0
1.0
1.0
1.0
1.0
1.0
1.1
3.0
1.1
4.0
3.1
3.5
2.6
7.2
9.6
7.1
8.0
7.5
7.0
6.5
6.0
5.5
5.0
4.5
4.0
3.5
3.0
2.5
2.0
1.5
1.0
0.5
0.0
-0.5
f1 (ppm)

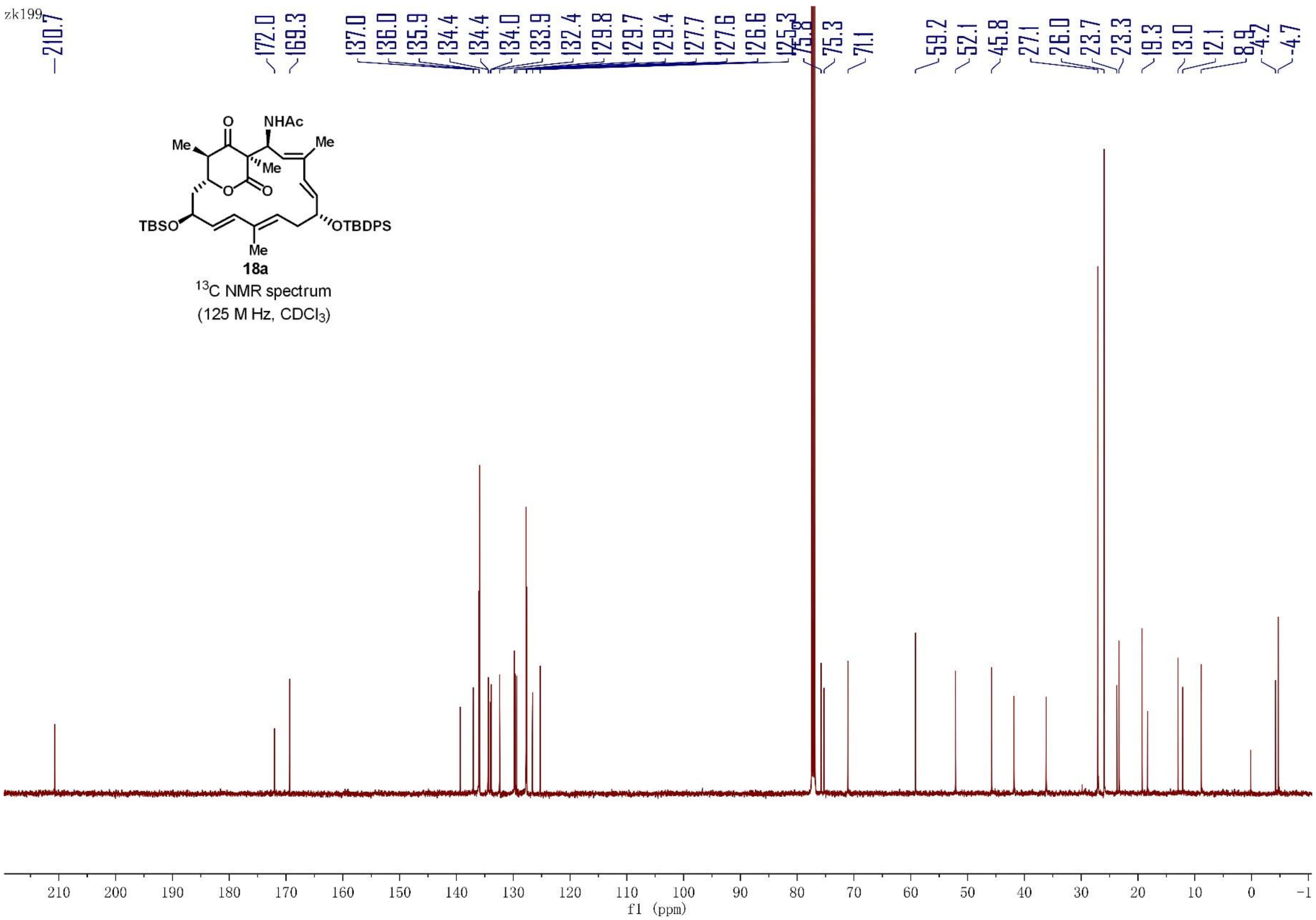
zk199
210.7
172.0
169.3
137.0
136.0
135.9
134.4
134.4
134.0
133.9
132.4
129.8
129.7
129.4
127.7
127.6
126.6
125.3
75.8
75.3
71.1
59.2
52.1
45.8
27.1
26.0
23.7
23.3
19.3
13.0
12.1
8.9
-4.2
-4.7
NHAc
Me
Me
Me
O
O
O
TBSO
OTBDPS
Me
18a
^{13}C NMR spectrum
(125 M Hz, CDCl$_3$)
210 200 190 180 170 160 150 140 130 120 110 100 90 80 70 60 50 40 30 20 10 0 −1
f1 (ppm)

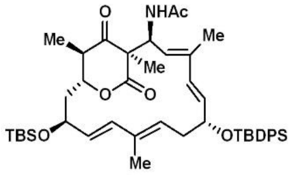

18a
H-H COSY spectrum
(500 M Hz, $CDCl_3$)

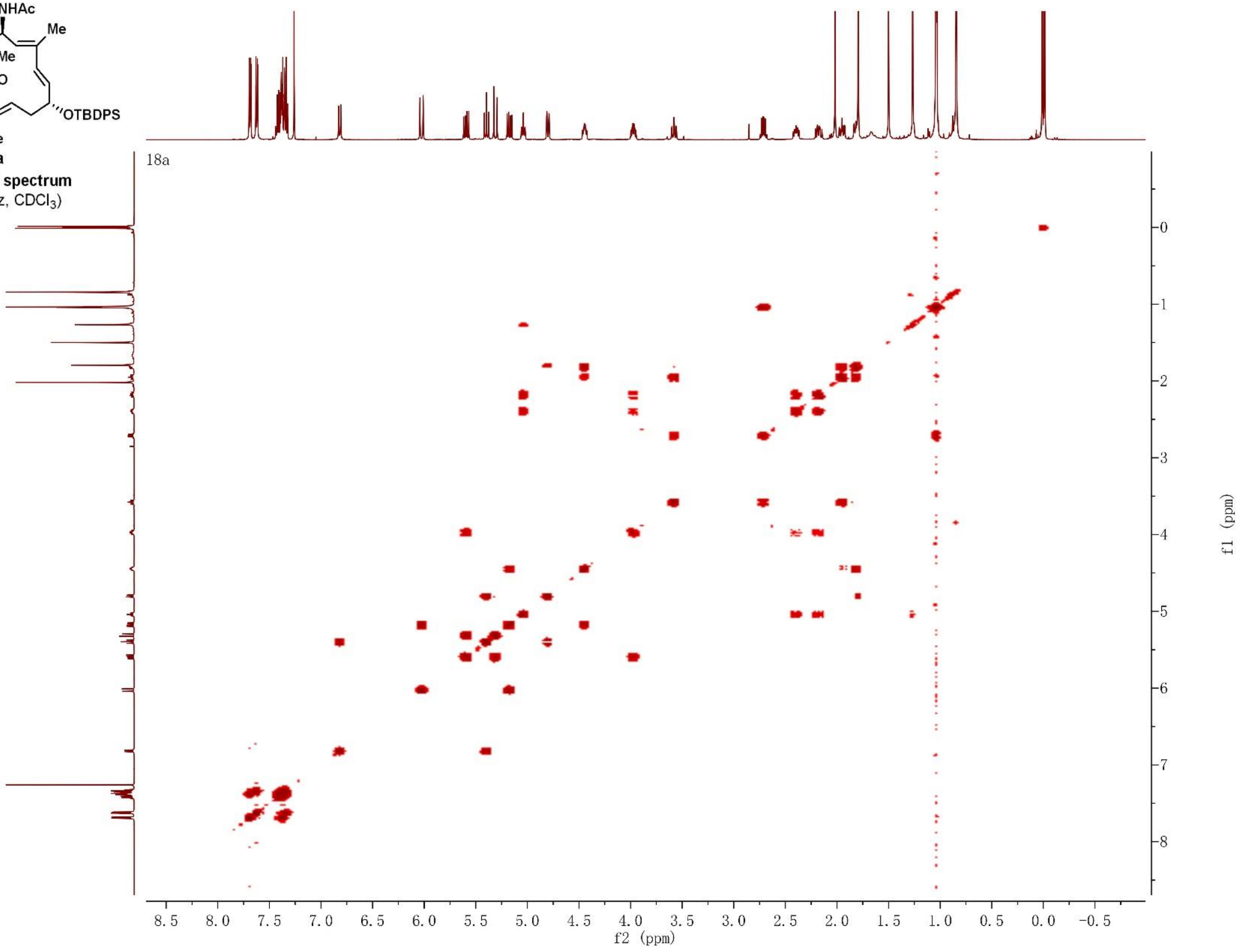

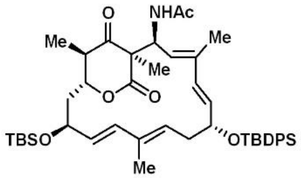

18a

ROESY spectrum
(500 M Hz, $CDCl_3$)

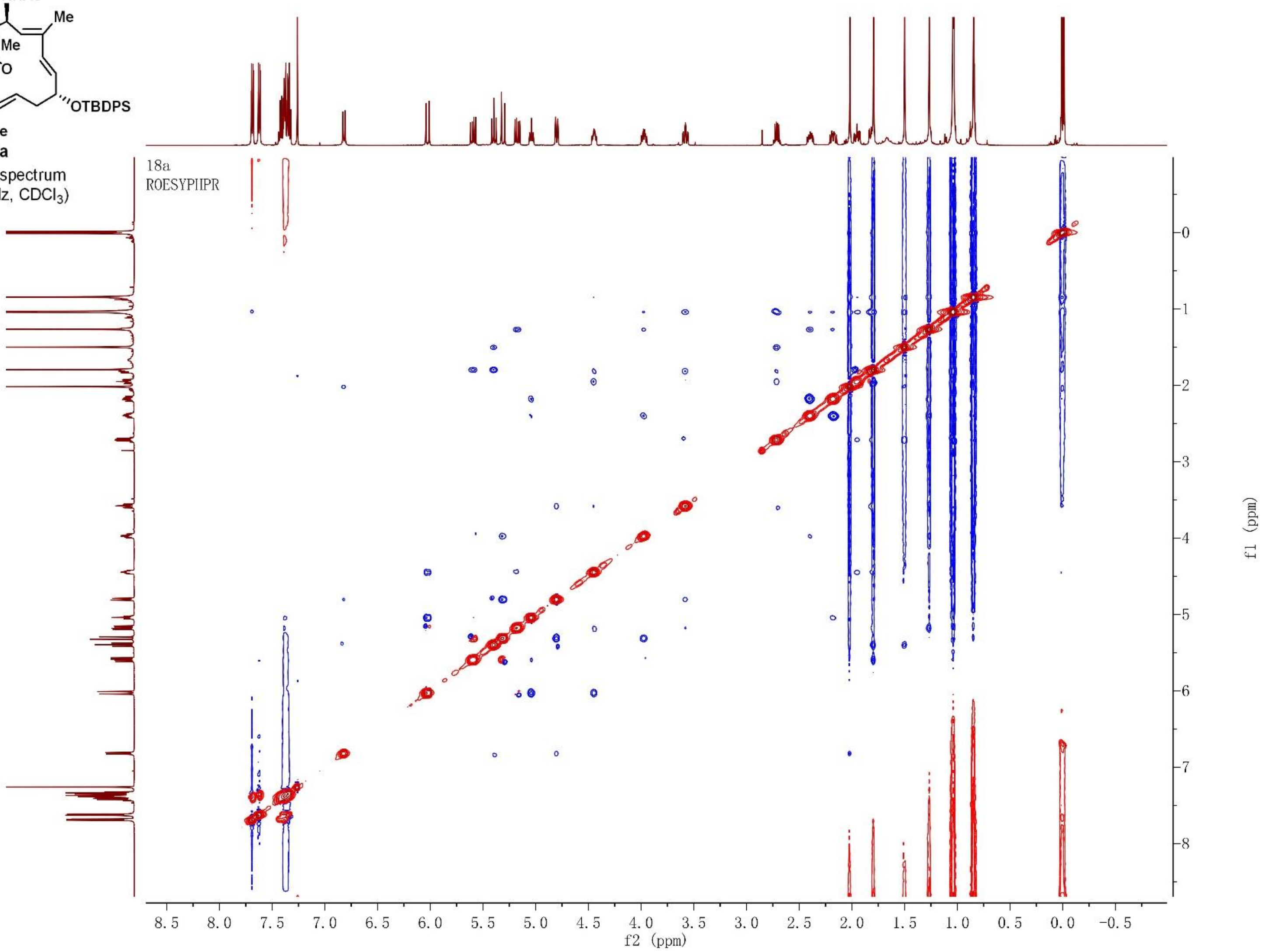

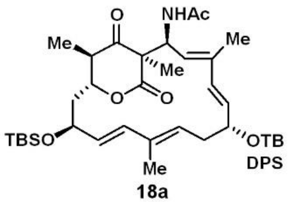

18a

C-H HSQC spectrum
(500 M Hz, $CDCl_3$)

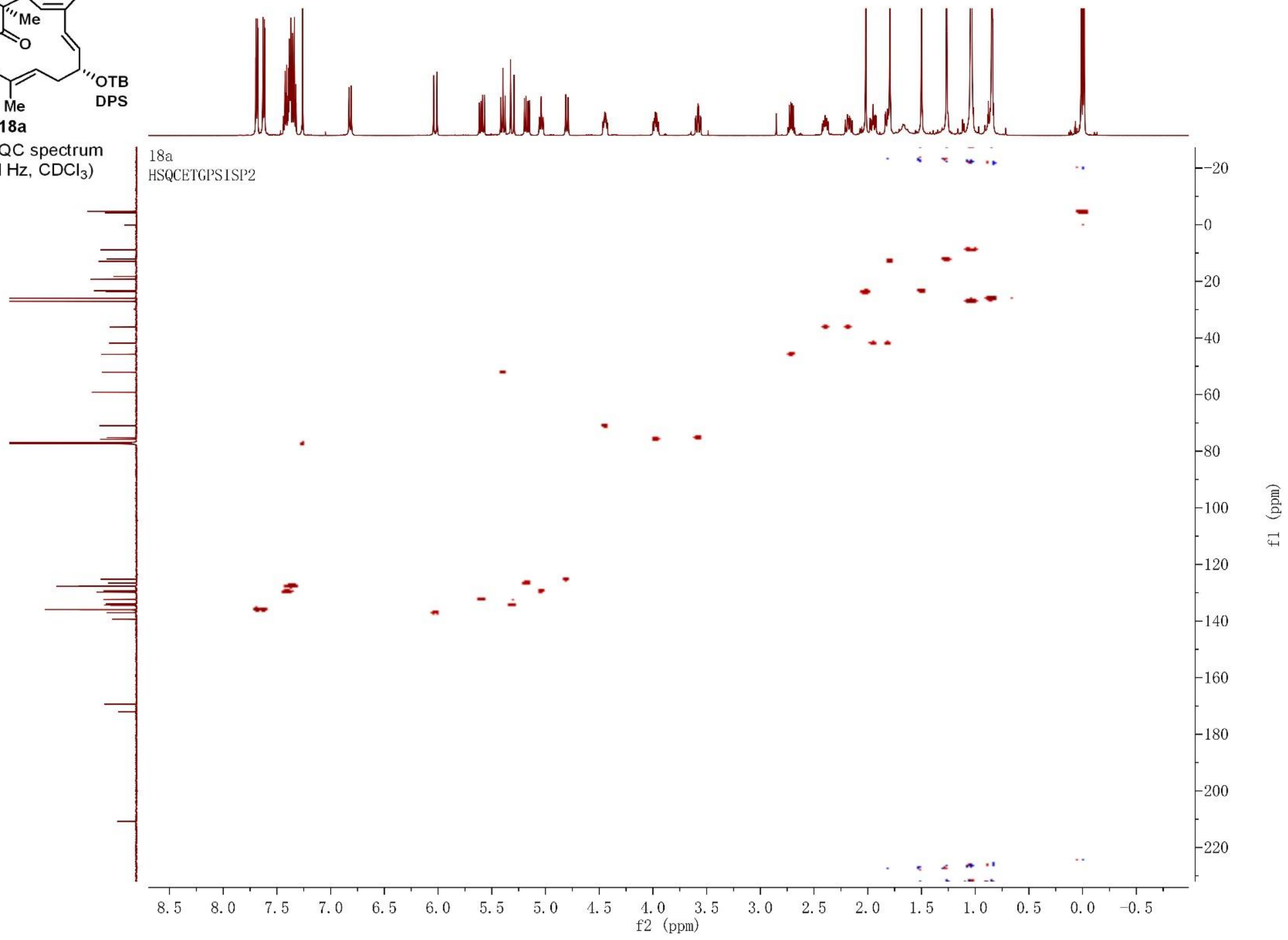

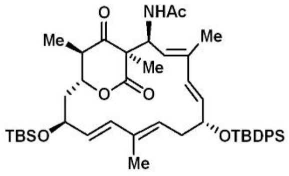

18a

C-H HMBC spectrum
(500 M Hz, $CDCl_3$)

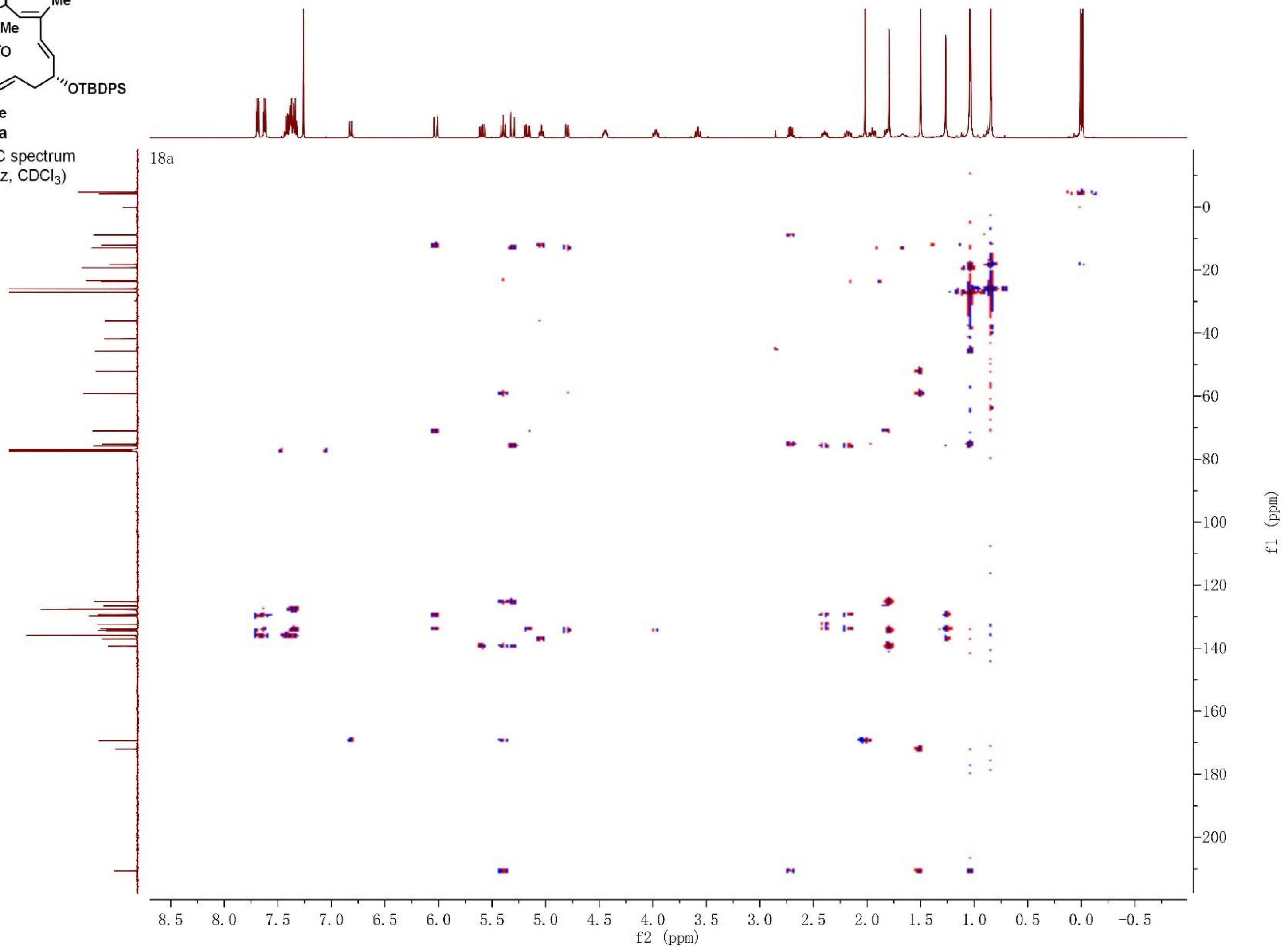

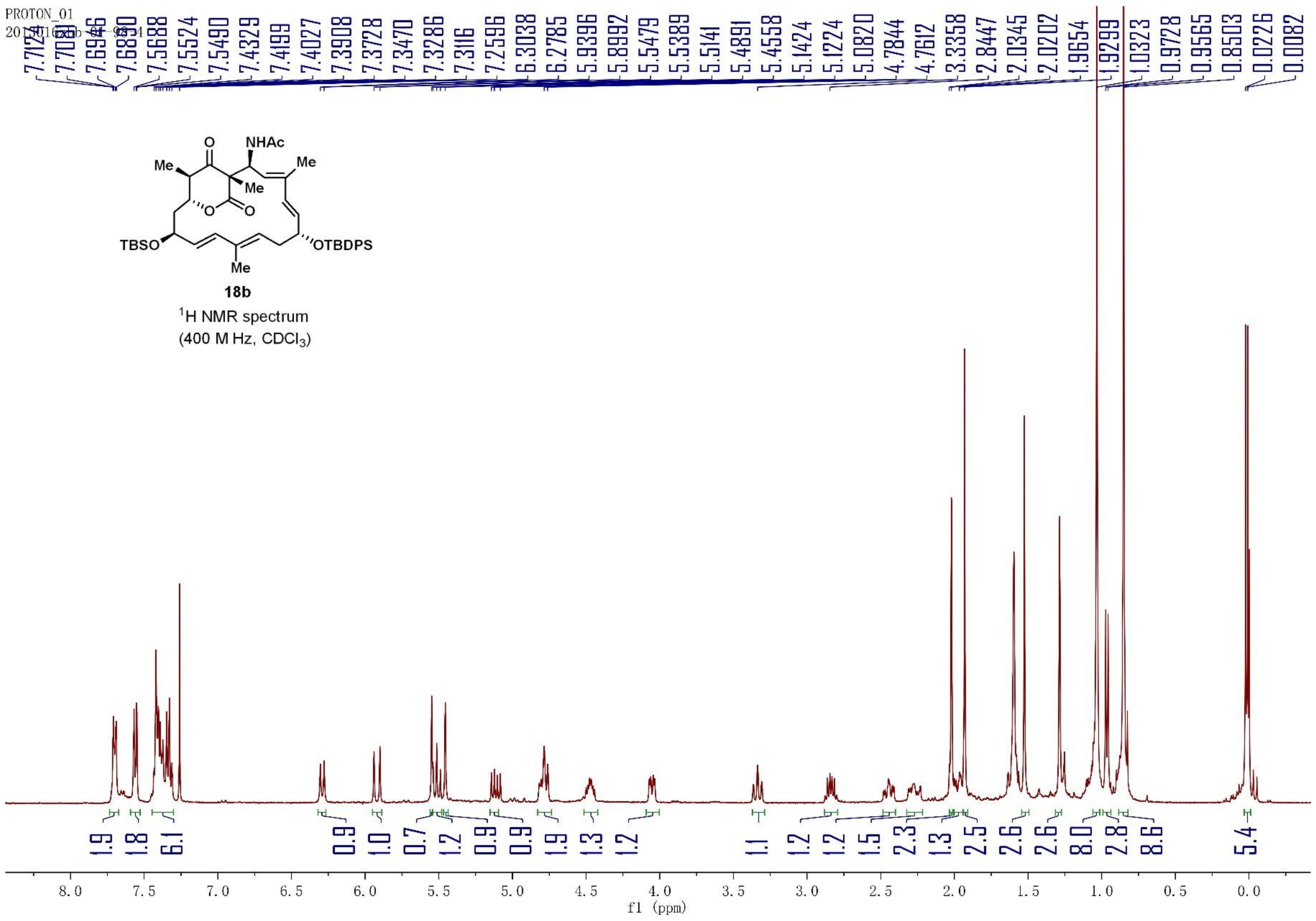
PROTON_01
NHAc
Me
Me
Me
O
O
O
TBSO
OTBDPS
Me
18b
1H NMR spectrum
(400 M Hz, CDCl3)
f1 (ppm)

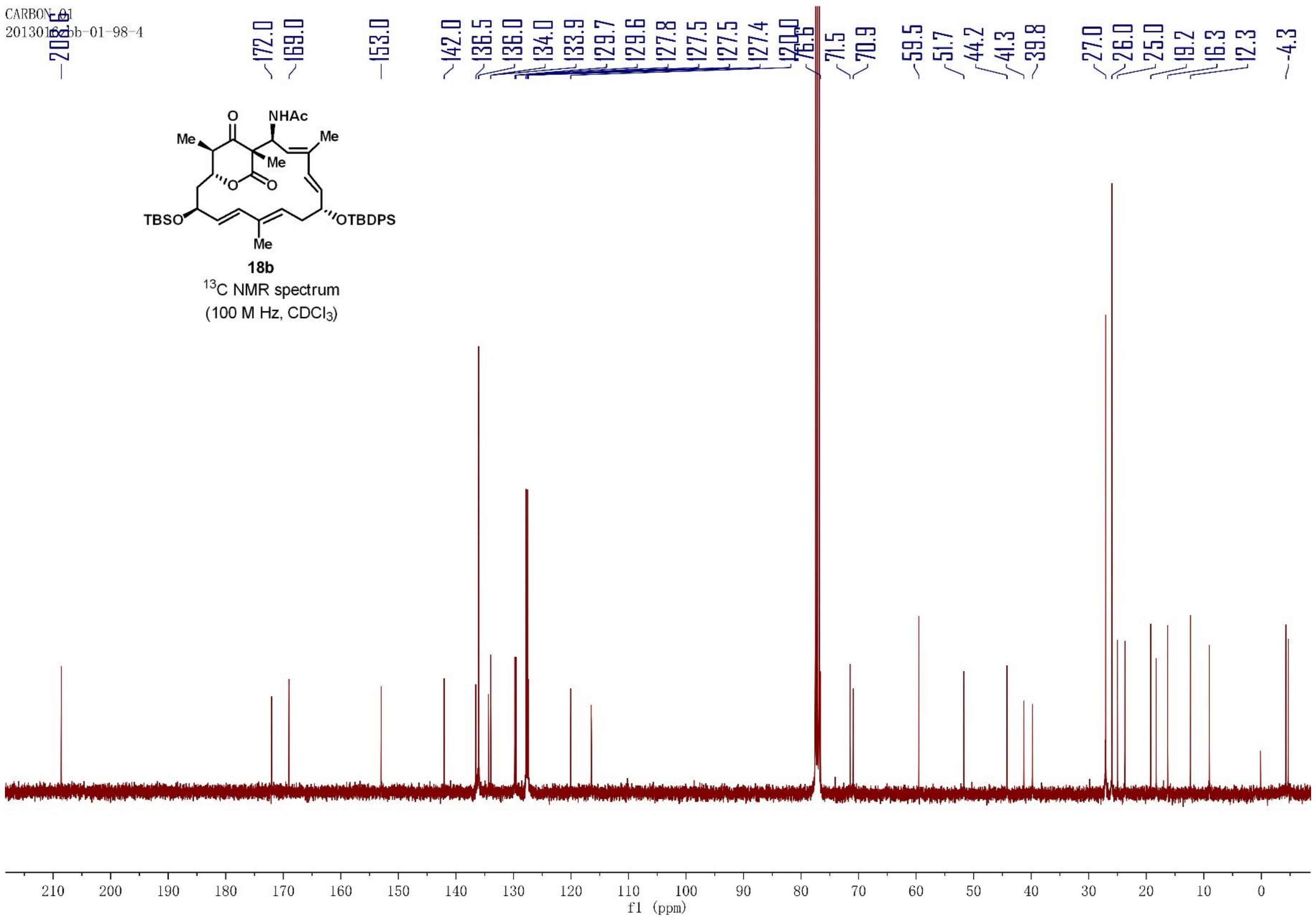

18b

^{13}C NMR spectrum

(100 M Hz, $CDCl_3$)

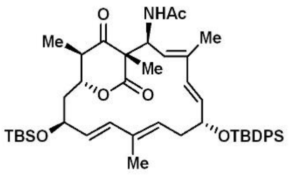

18b

H-H COSY spectrum

(400 M Hz, $CDCl_3$)

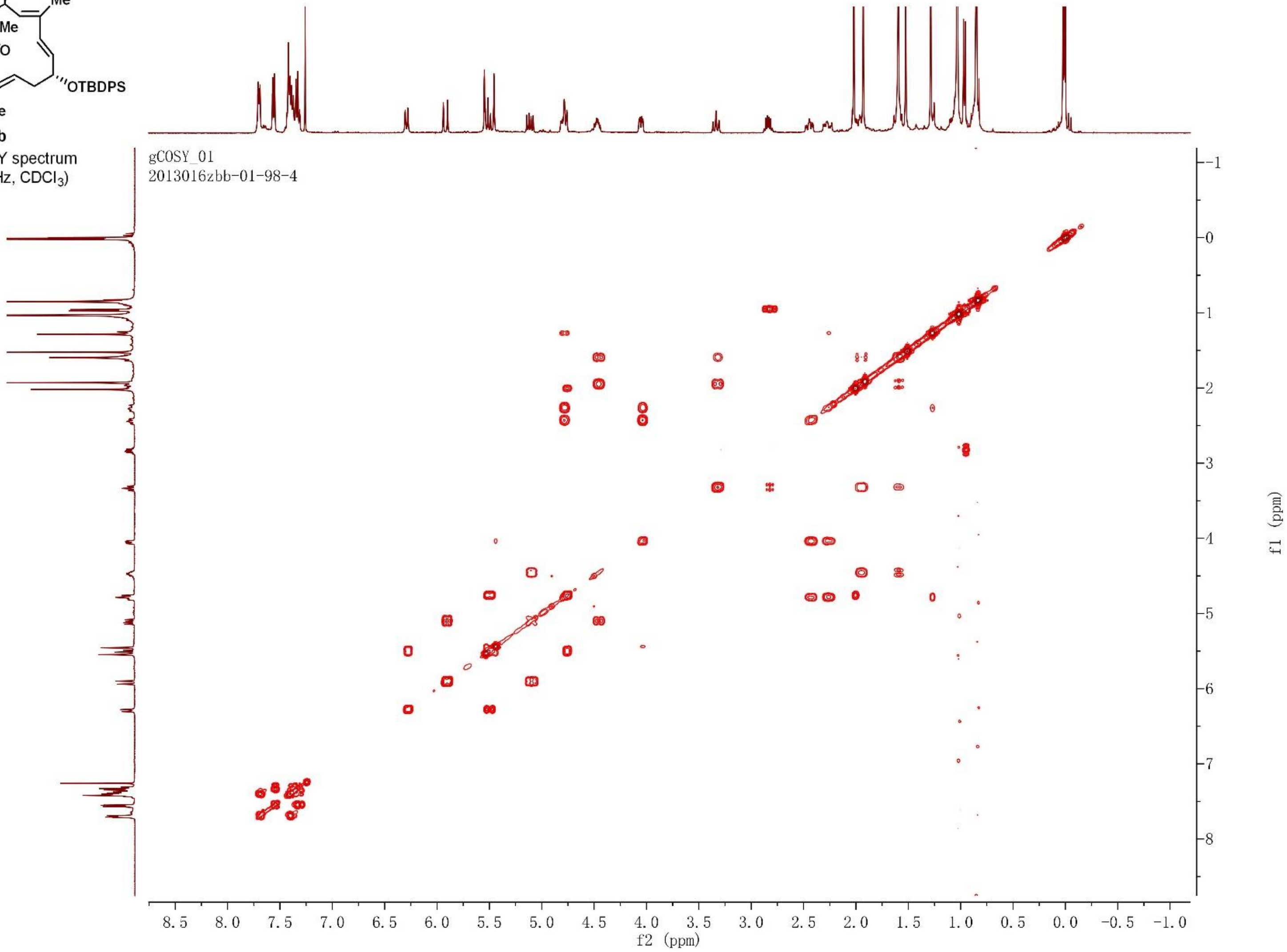

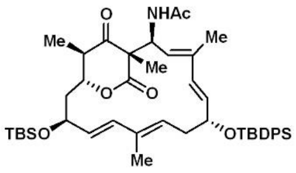

18b

NOESY spectrum
(400 M Hz, $CDCl_3$)

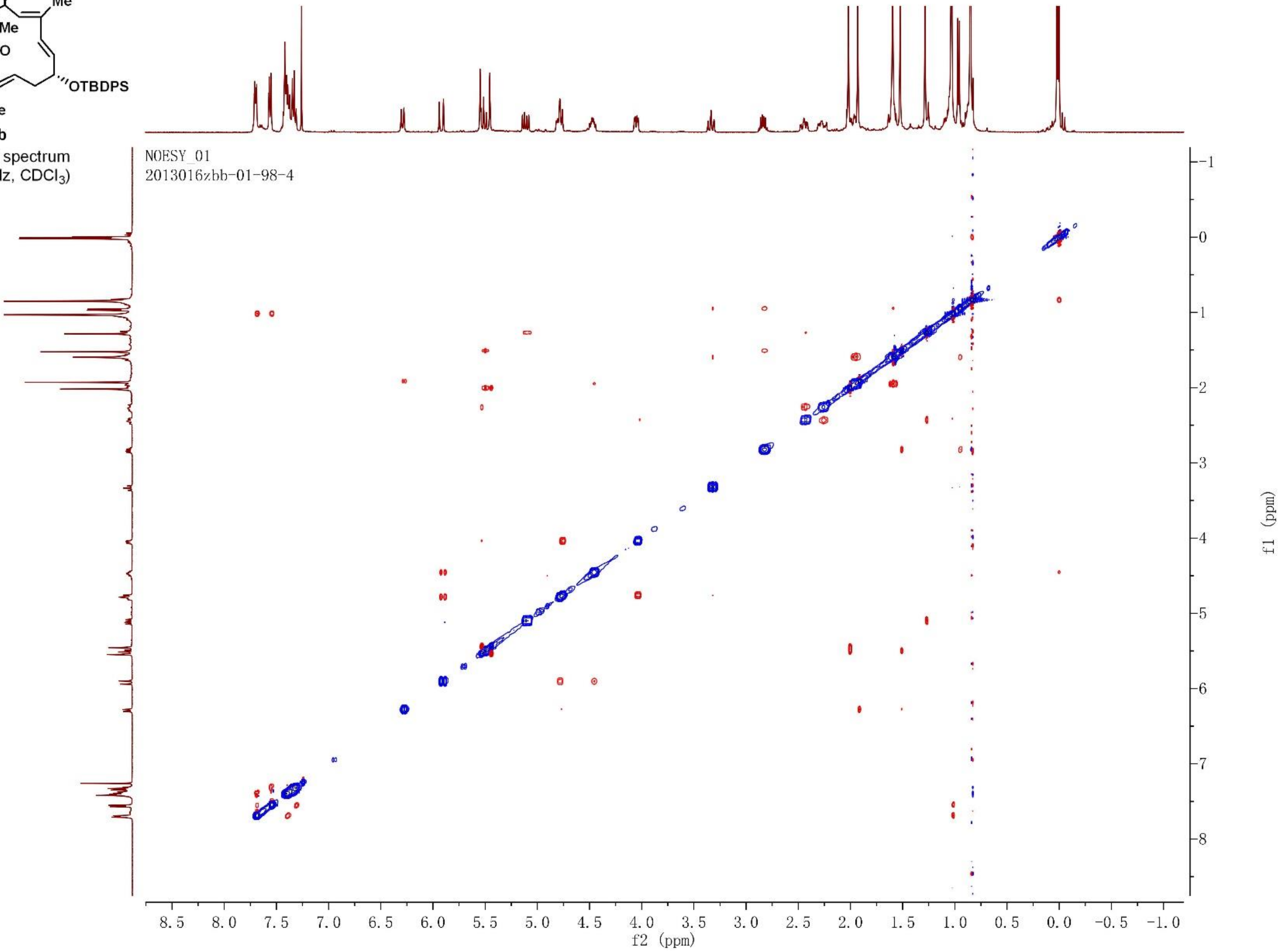

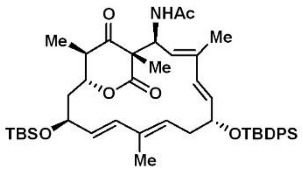

18b

C-H HSQC spectrum
(400 M Hz, $CDCl_3$)

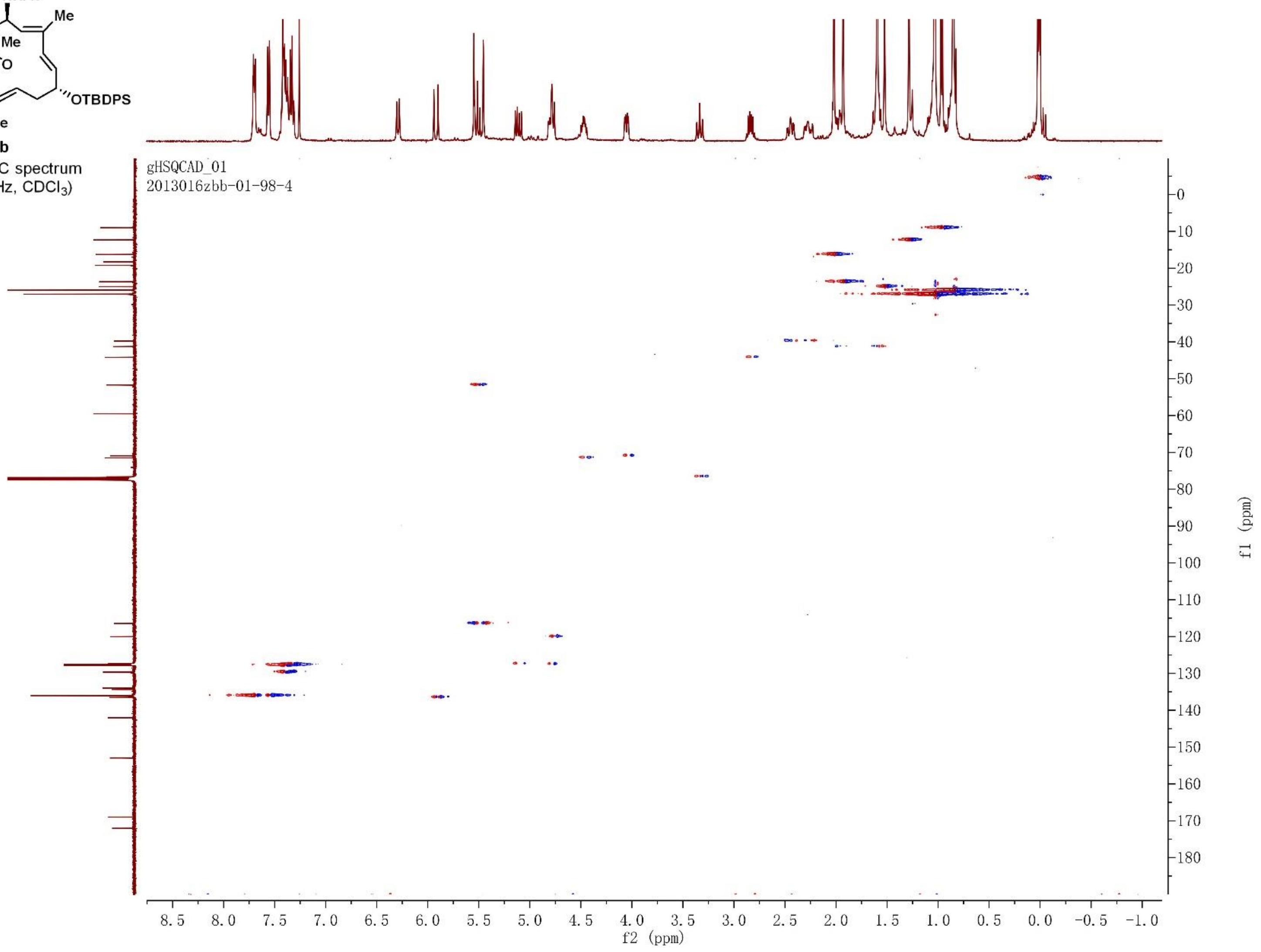

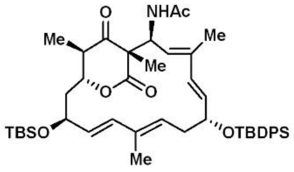

18b

C-H HMBC spectrum
(400 M Hz, $CDCl_3$)

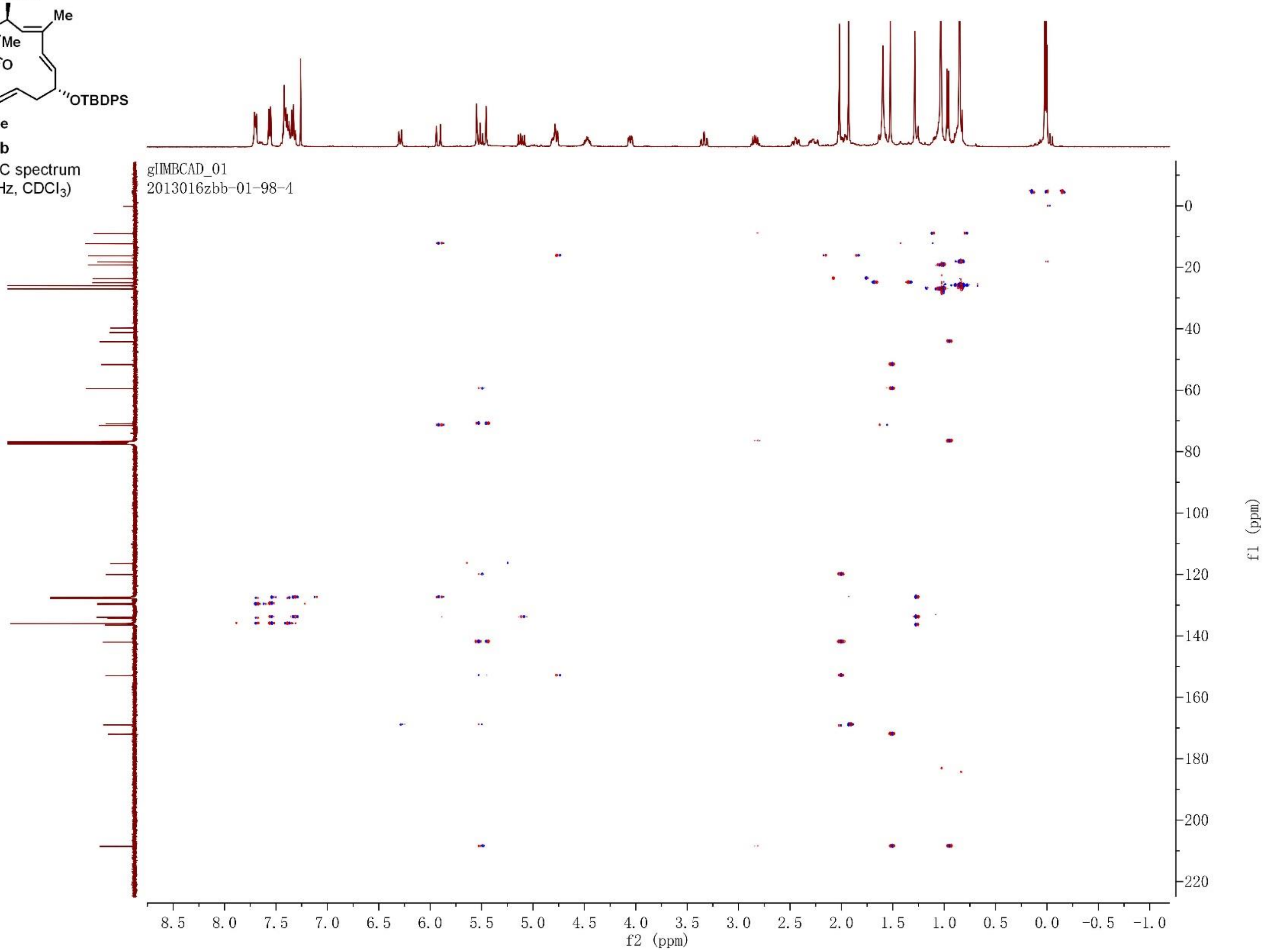

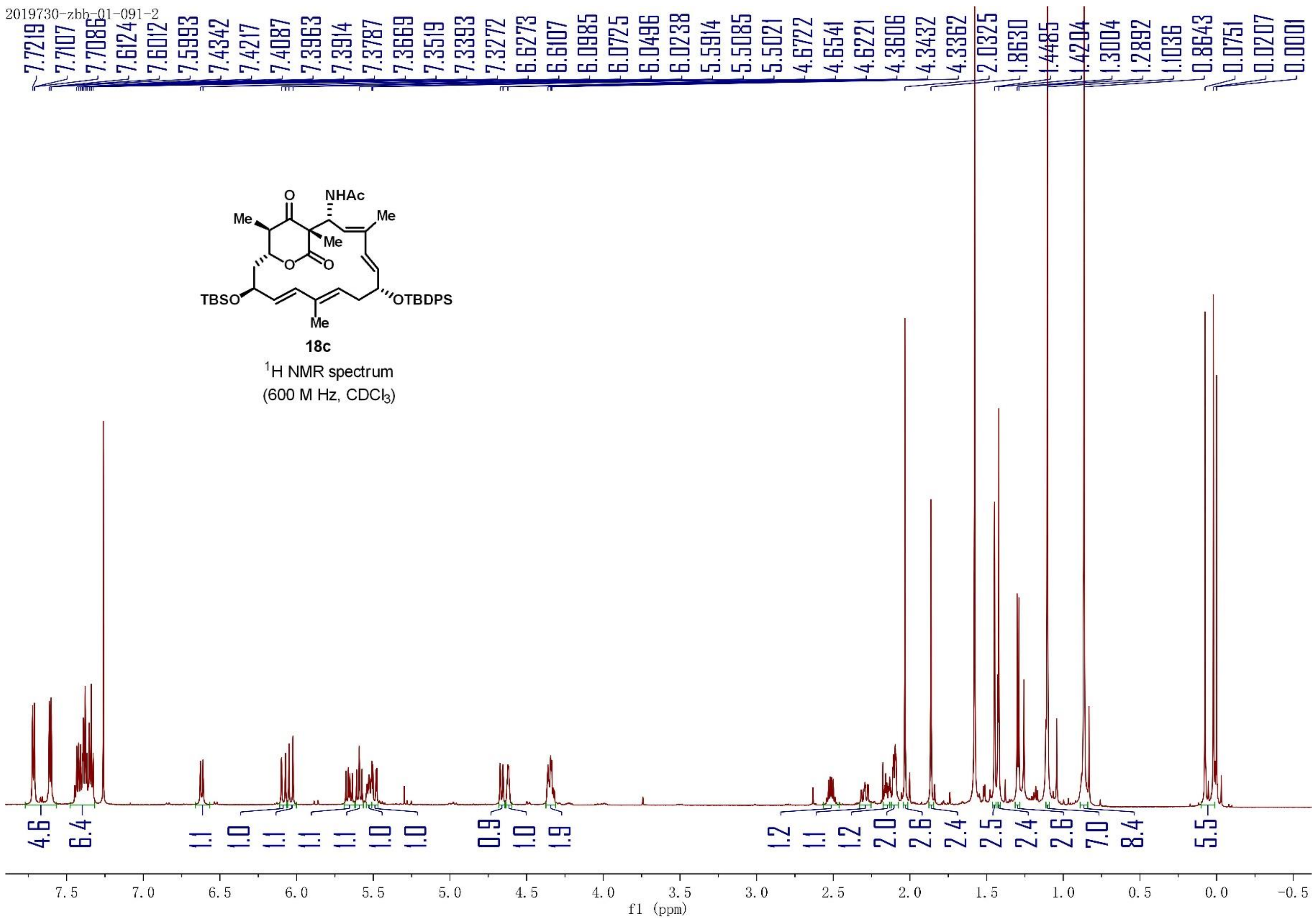

2019730-zbb-01-091-2
7.7219
7.7107
7.7086
7.6124
7.6012
7.5993
7.4342
7.4217
7.4087
7.3963
7.3914
7.3787
7.3669
7.3519
7.3393
7.3272
6.6273
6.6107
6.0985
6.0725
6.0496
6.0238
5.5914
5.5085
5.5021
4.6722
4.6541
4.6221
4.3606
4.3432
4.3362
2.0325
1.8630
1.4485
1.4204
1.3004
1.2892
1.1036
0.8643
0.0751
0.0207
0.0001
NHAc
Me
O
TBSO
OTBDPS
18c
^{1}H NMR spectrum
(600 M Hz, $CDCl_3$)
4.6
6.4
1.1
1.0
1.1
1.1
1.1
1.0
1.0
0.9
1.0
1.9
1.2
1.1
1.2
2.0
2.6
2.4
2.5
2.4
2.6
7.0
8.4
5.5
f1 (ppm)

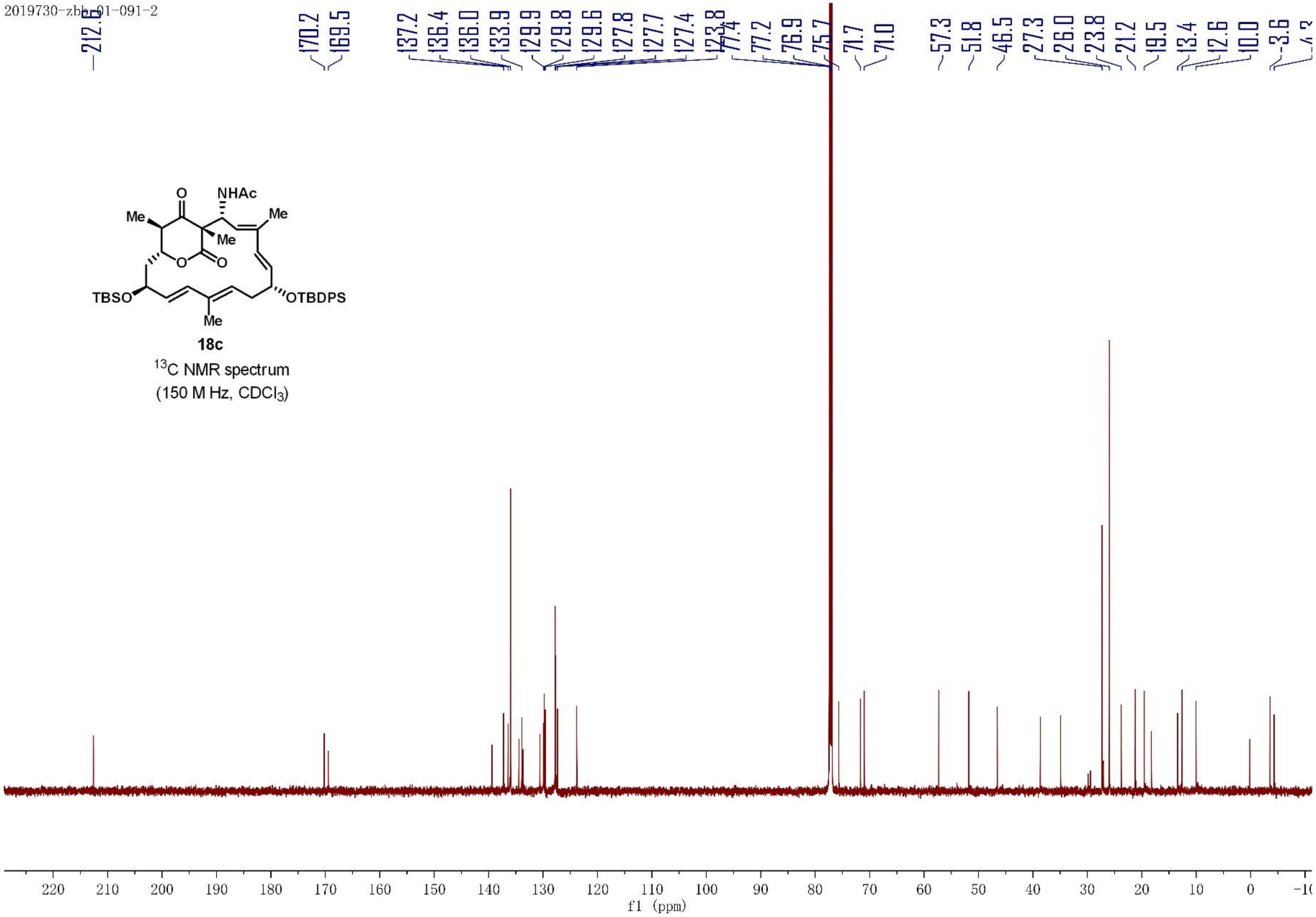
2019730-zbb-01-091-2
212.6
170.2
169.5
137.2
136.4
136.0
133.9
129.9
129.8
129.6
127.8
127.7
127.4
123.8
77.4
77.2
76.9
75.7
71.7
71.0
57.3
51.8
46.5
27.3
26.0
23.8
21.2
19.5
13.4
12.6
10.0
-3.6
NHAc
Me
Me
Me
O
O
O
TBSO
OTBDPS
Me
18c
^{13}C NMR spectrum
(150 M Hz, $CDCl_3$)
220
210
200
190
180
170
160
150
140
130
120
110
100
90
80
70
60
50
40
30
20
10
0
f1 (ppm)

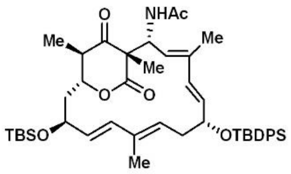

18c

H-H COSY spectrum
(600 M Hz, $CDCl_3$)

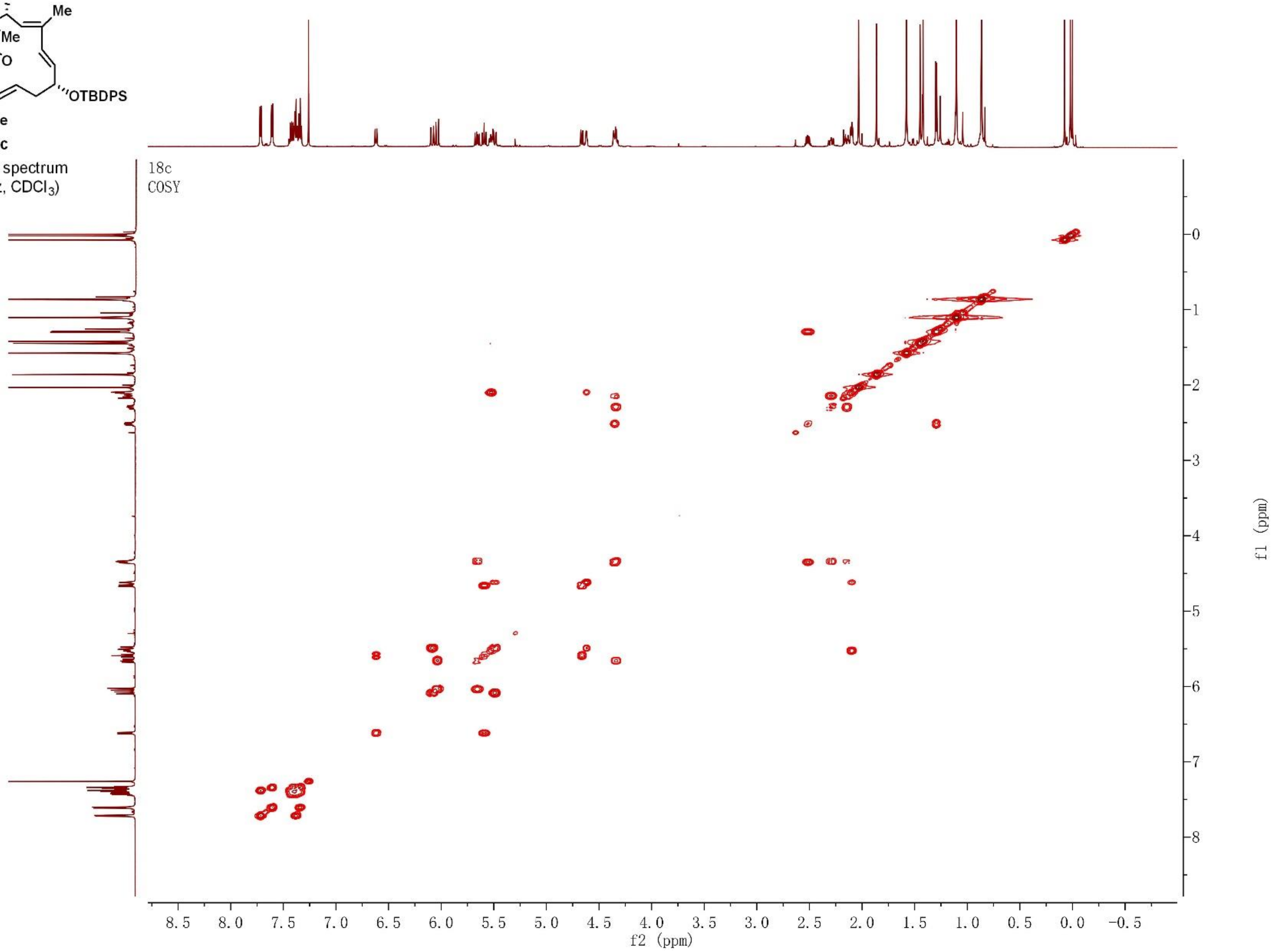

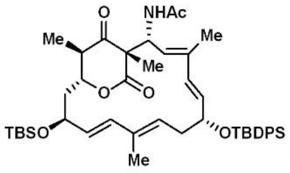

18c

ROESY spectrum
(600 M Hz, $CDCl_3$)

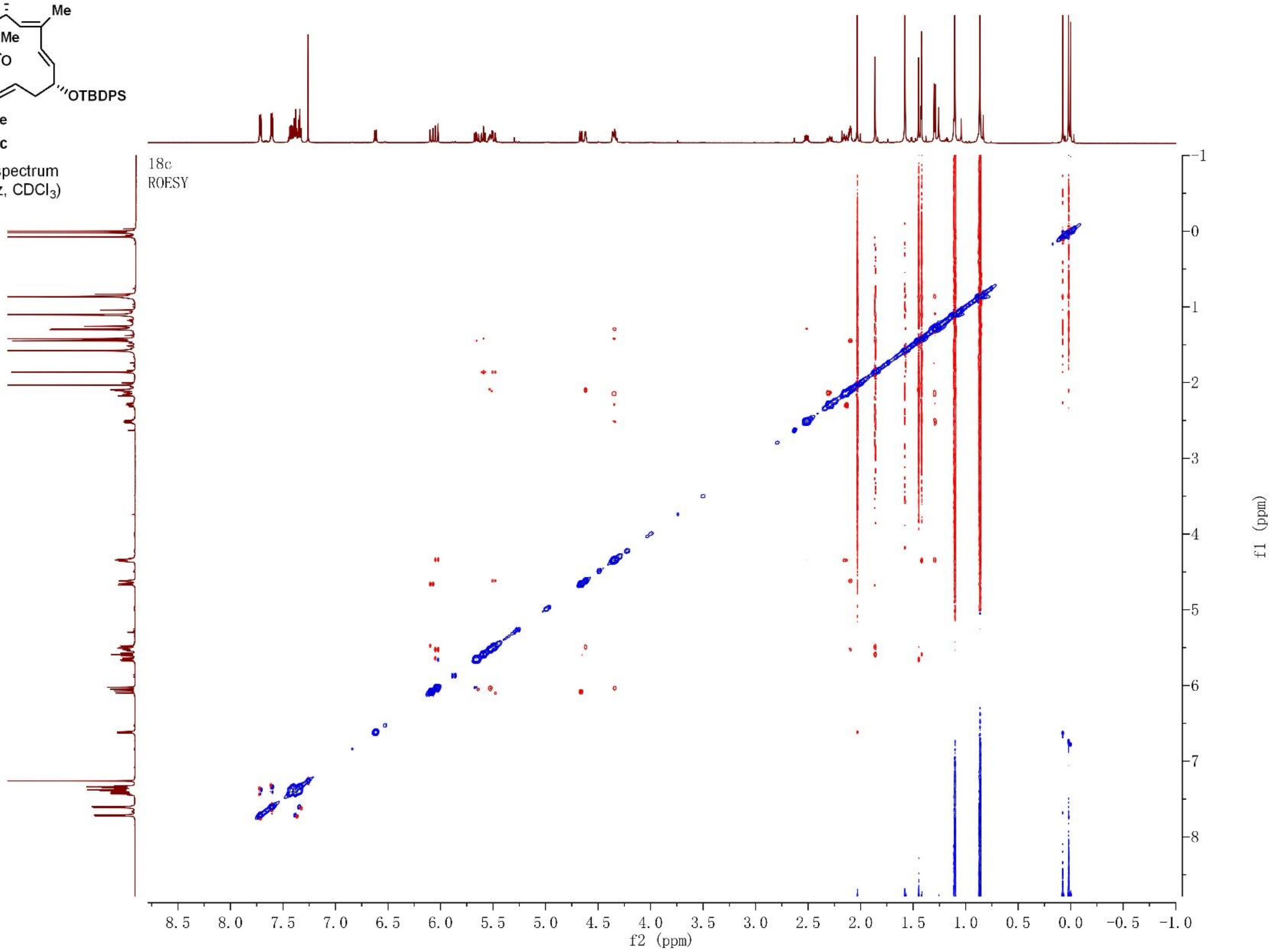

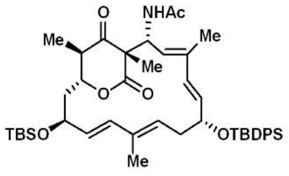

18c

C-H HSQC spectrum
(600 M Hz, $CDCl_3$)

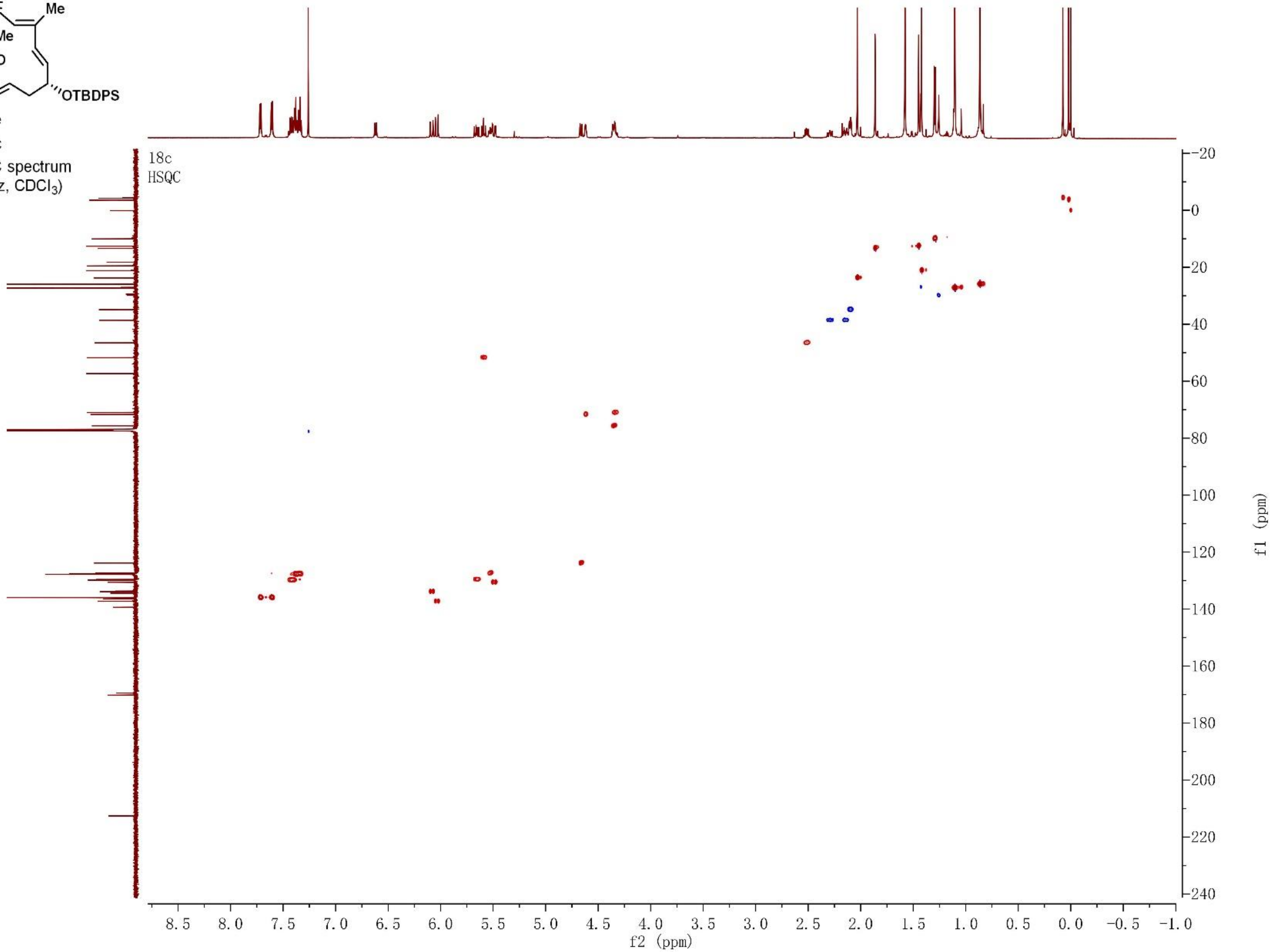

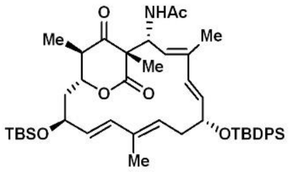

18c

C-H HMBC spectrum
(600 M Hz, $CDCl_3$)

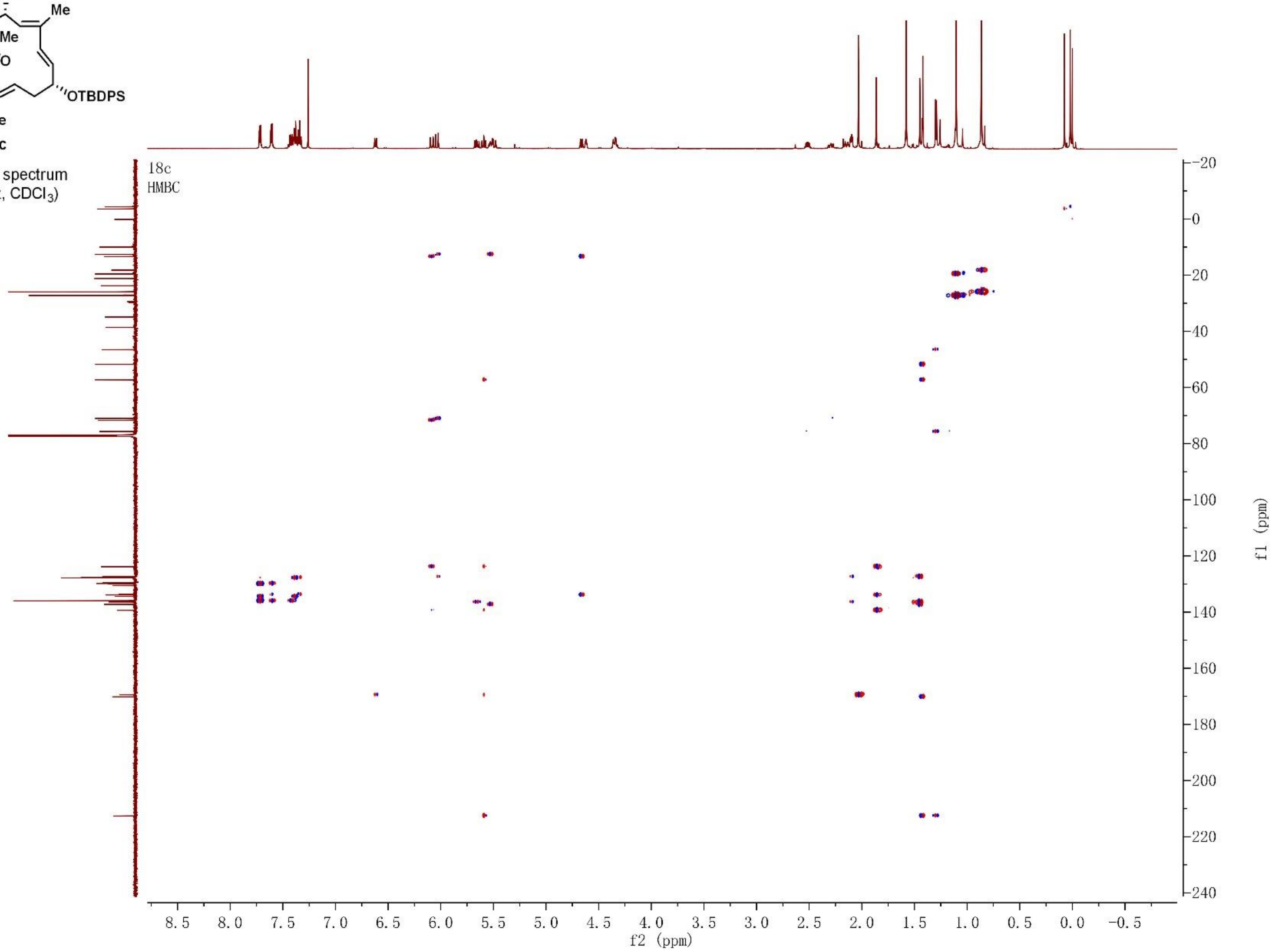

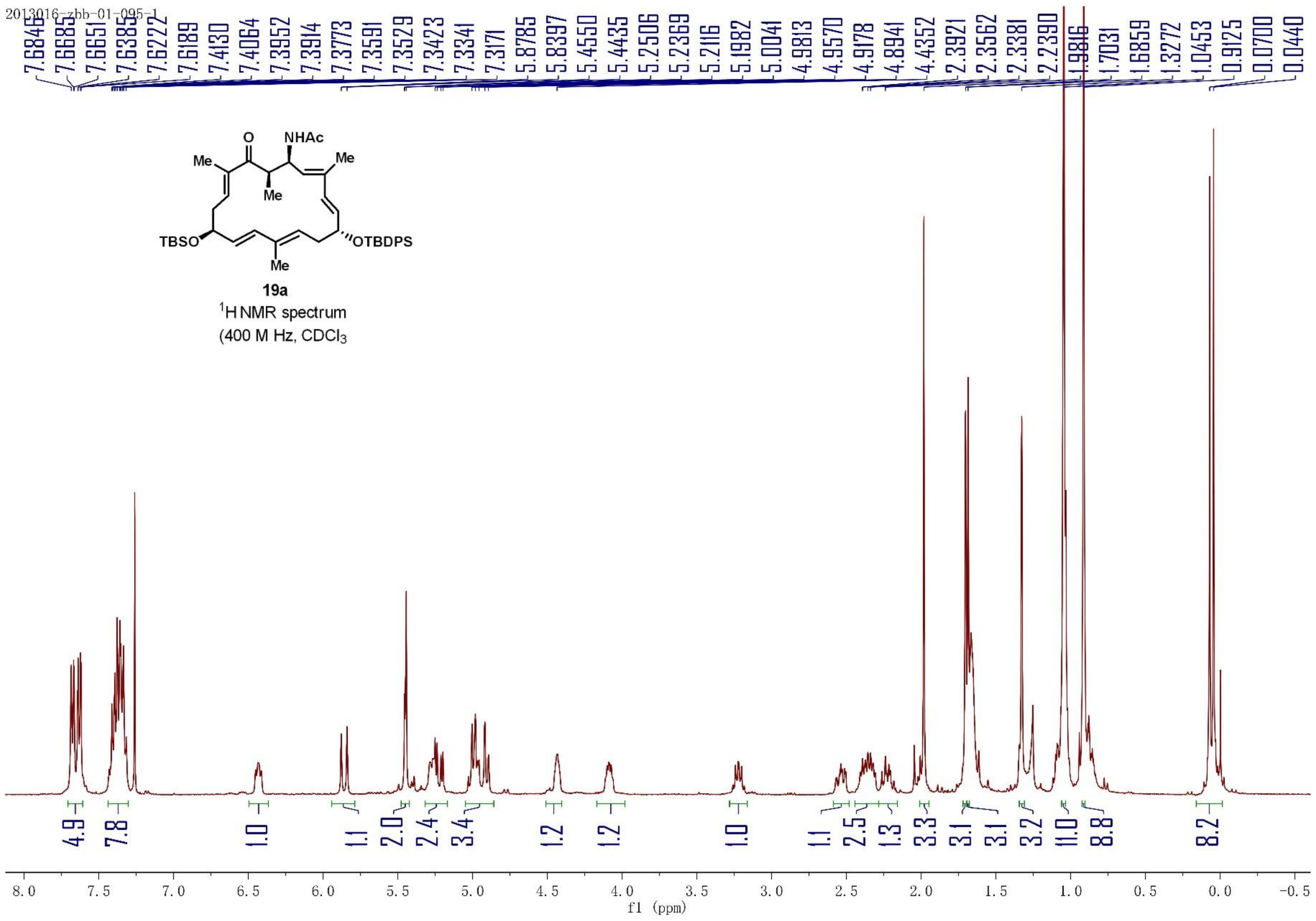
2013016-zbb-01-095-1
NHAc
Me
TBSO
OTBDPS
19a
^{1}H NMR spectrum
(400 M Hz, CDCl$_3$
f1 (ppm)

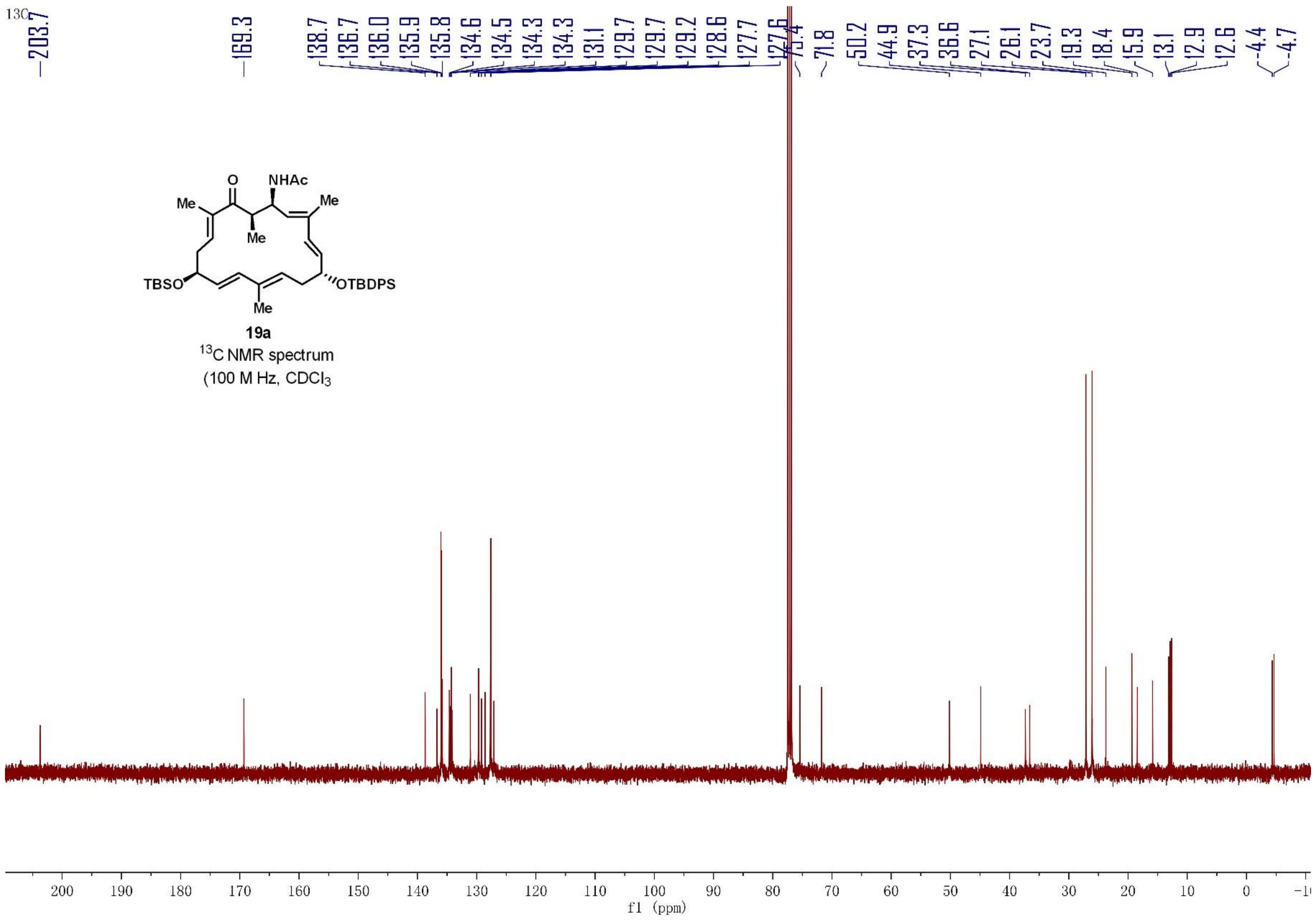

13C
203.7
169.3
138.7
136.7
136.0
135.9
135.8
134.6
134.5
134.3
134.3
131.1
129.7
129.7
129.2
128.6
127.7
127.6
75.4
71.8
50.2
44.9
37.3
36.6
27.1
26.1
23.7
19.3
18.4
15.9
13.1
12.9
12.6
-4.4
-4.7
O
NHAc
Me
Me
Me
TBSO
OTBDPS
Me
19a
^{13}C NMR spectrum
(100 M Hz, CDCl$_3$
200 190 180 170 160 150 140 130 120 110 100 90 80 70 60 50 40 30 20 10 0
f1 (ppm)

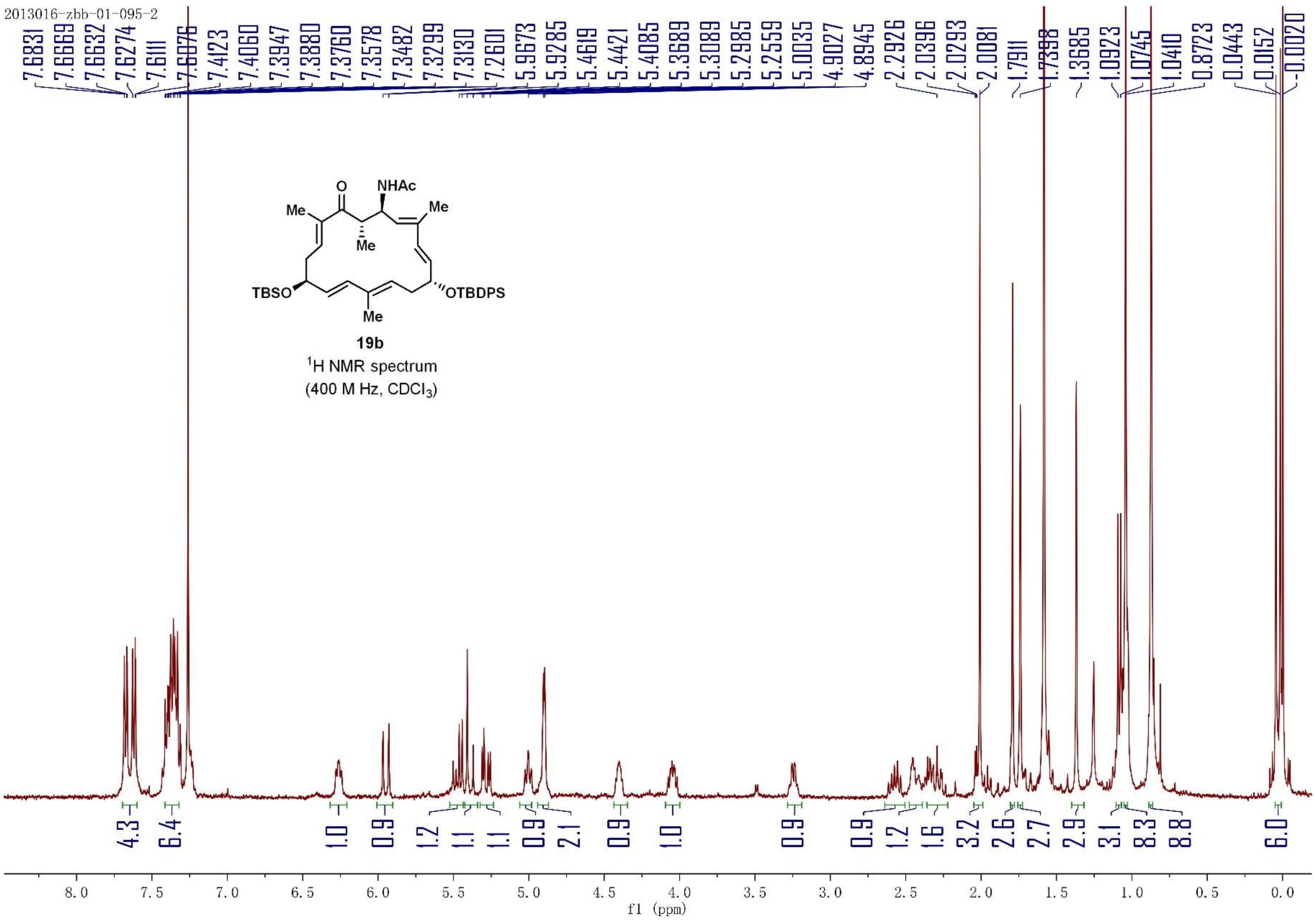
2013016-zbb-01-095-2
NHAc
Me
Me
Me
TBSO
OTBDPS
Me
19b
^{1}H NMR spectrum
(400 M Hz, CDCl$_3$)
f1 (ppm)

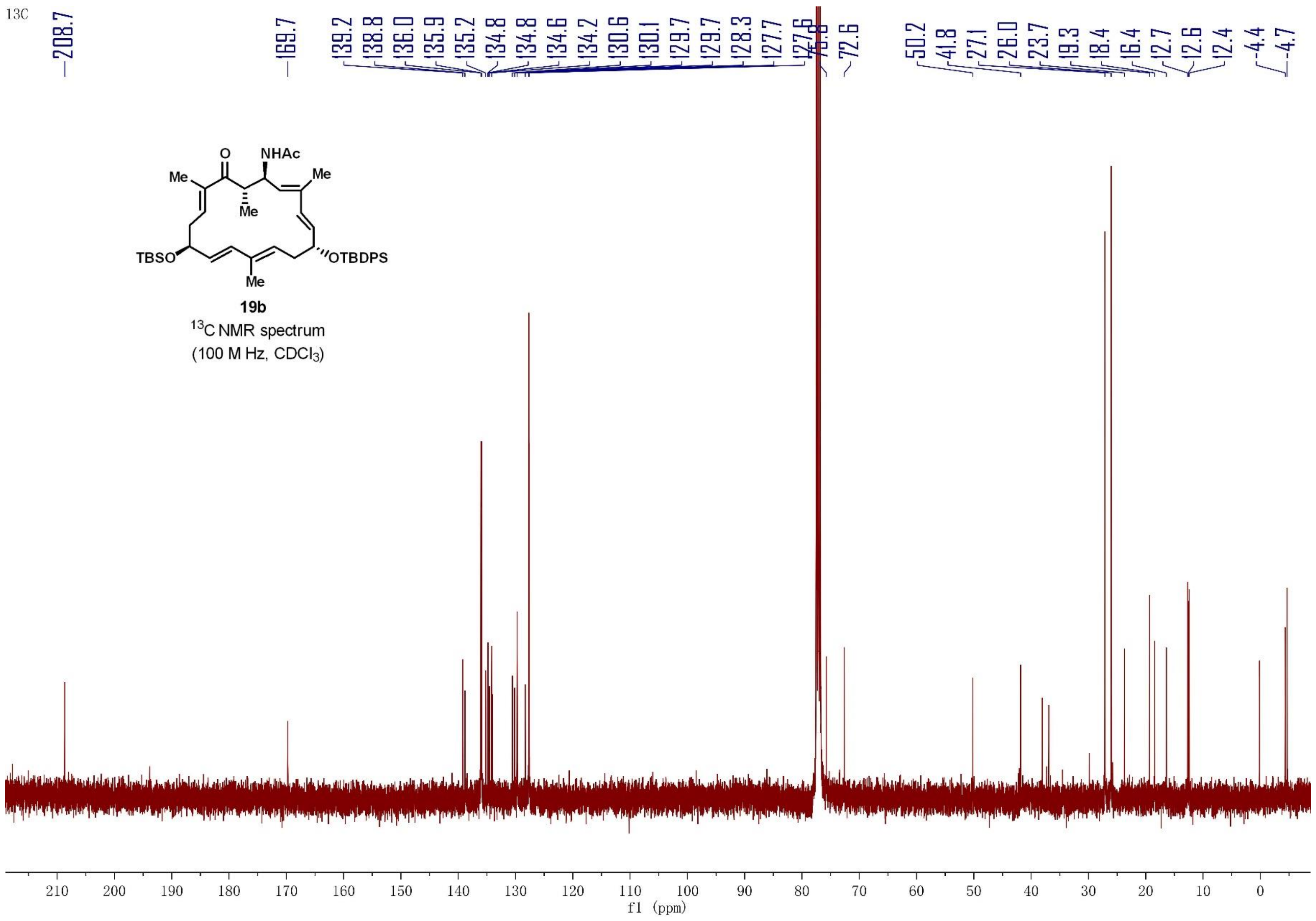
13C
208.7
169.7
139.2
138.8
136.0
135.9
135.2
134.8
134.8
134.6
134.2
130.6
130.1
129.7
129.7
128.3
127.7
127.6
72.6
50.2
41.8
27.1
26.0
23.7
19.3
18.4
16.4
12.7
12.6
12.4
-4.4
-4.7
O
NHAc
Me
Me
Me
TBSO
OTBDPS
Me
19b
13C NMR spectrum
(100 M Hz, CDCl3)
210
200
190
180
170
160
150
140
130
120
110
100
90
80
70
60
50
40
30
20
10
0
f1 (ppm)

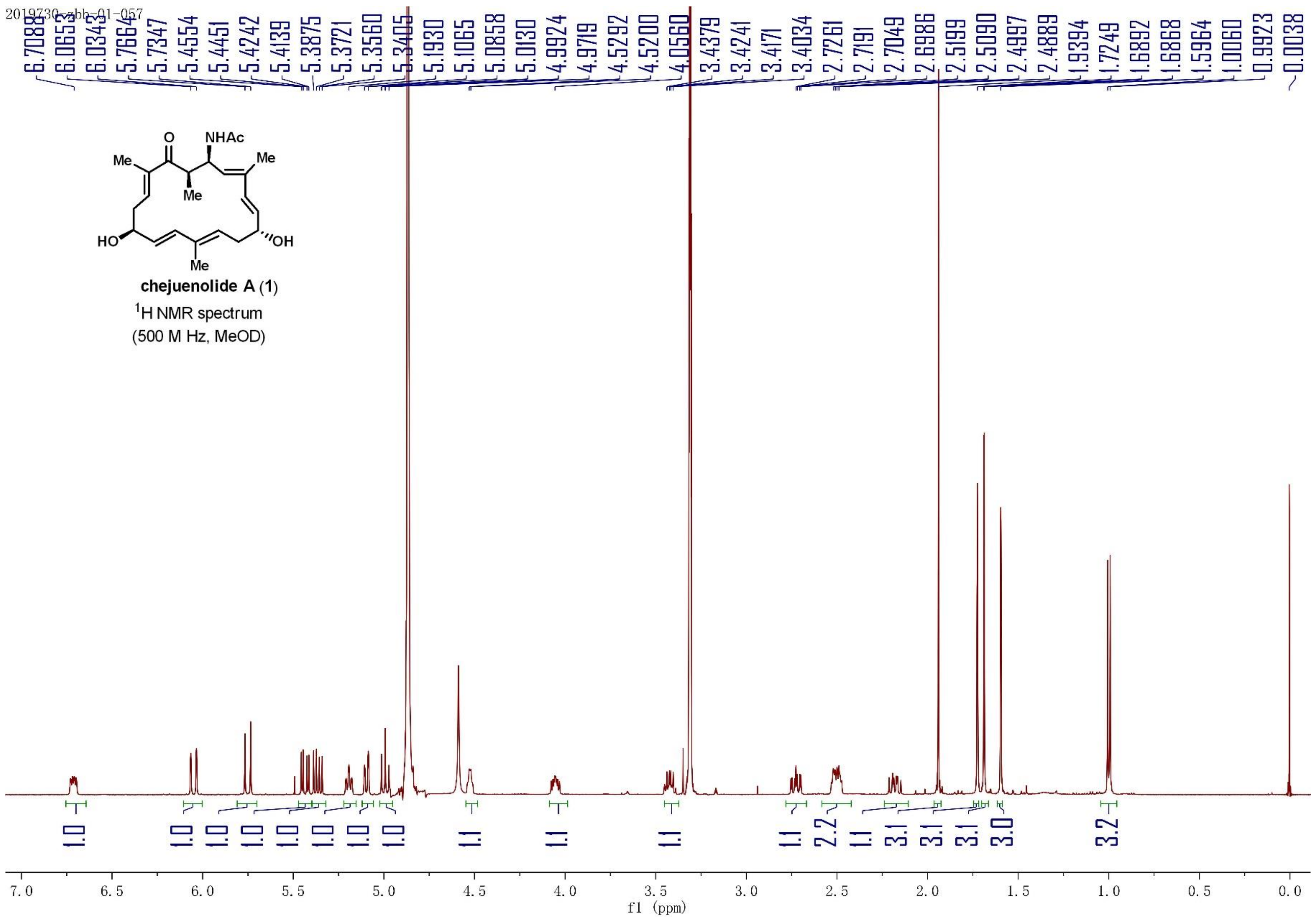

2019730-zbb-01-057
chejuenolide A (1)
1H NMR spectrum
(500 M Hz, MeOD)
f1 (ppm)

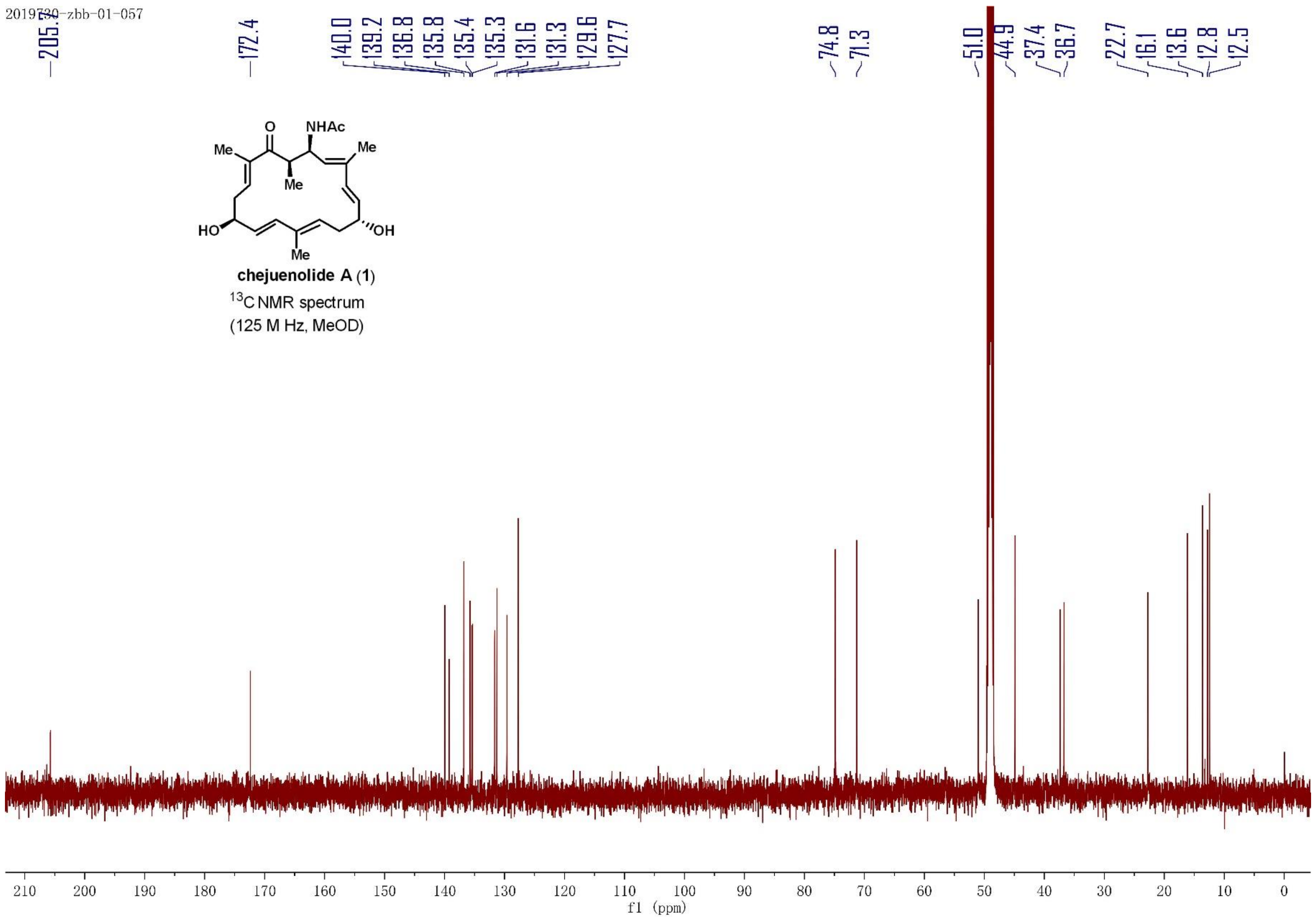
2019730-zbb-01-057
205.1
172.4
140.0
139.2
136.8
135.8
135.4
135.3
131.6
131.3
129.6
127.7
74.8
71.3
51.0
44.9
37.4
36.7
22.7
16.1
13.6
12.8
12.5
O
NHAc
Me
Me
Me
HO
OH
Me
chejuenolide A (1)
13C NMR spectrum
(125 M Hz, MeOD)
210
200
190
180
170
160
150
140
130
120
110
100
90
80
70
60
50
40
30
20
10
0
f1 (ppm)

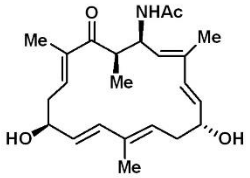

chejuenolide A (1)

H-H COSY spectrum
(500 M Hz, MeOD)

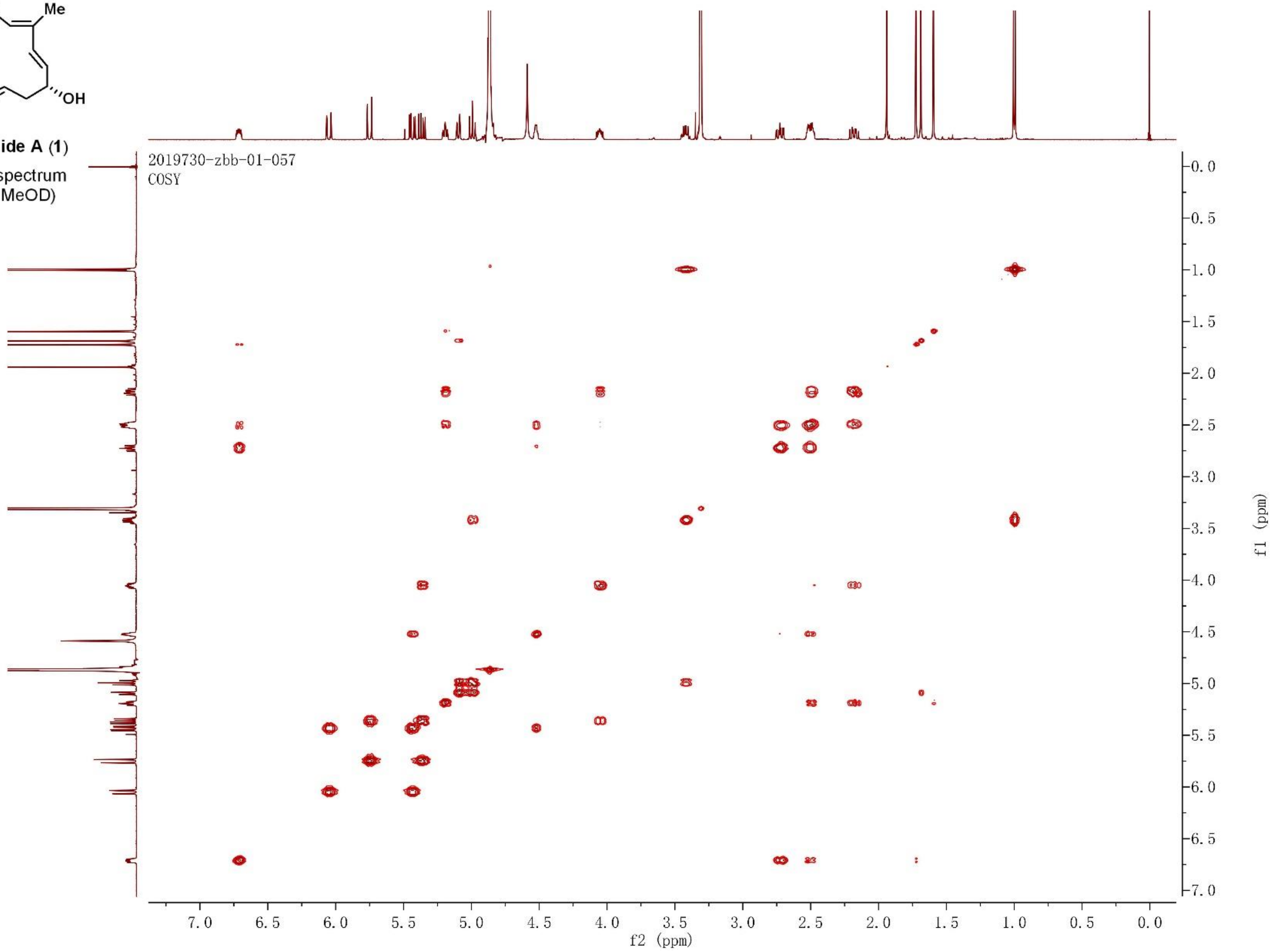

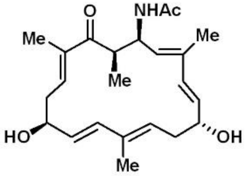

chejuenolide A (1)

NOESY spectrum
(500 M Hz, MeOD)

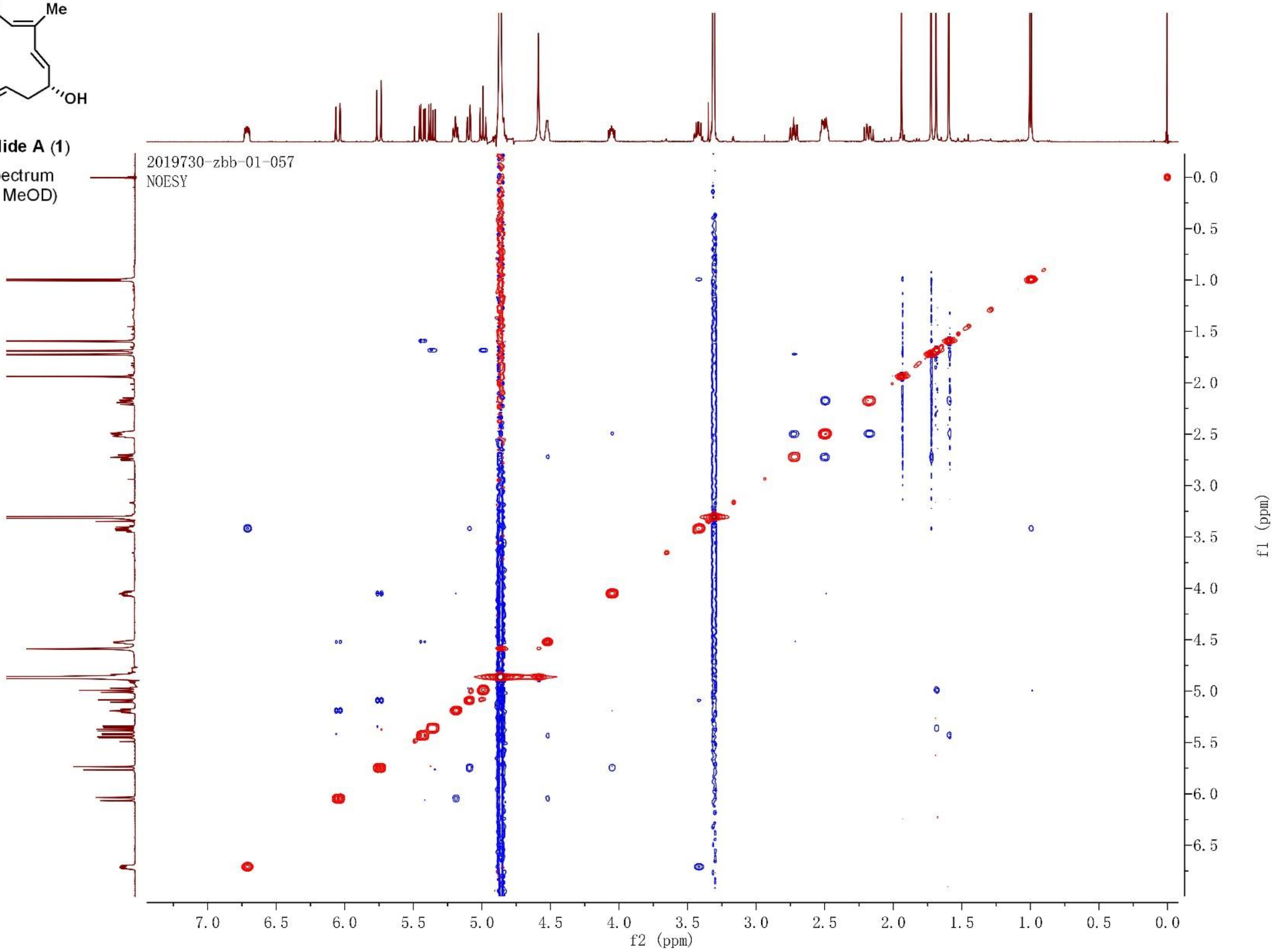

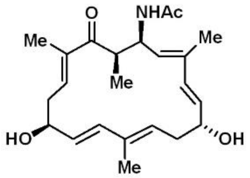

chejuenolide A (1)

C-H HSQC spectrum
(500 M Hz, MeOD)

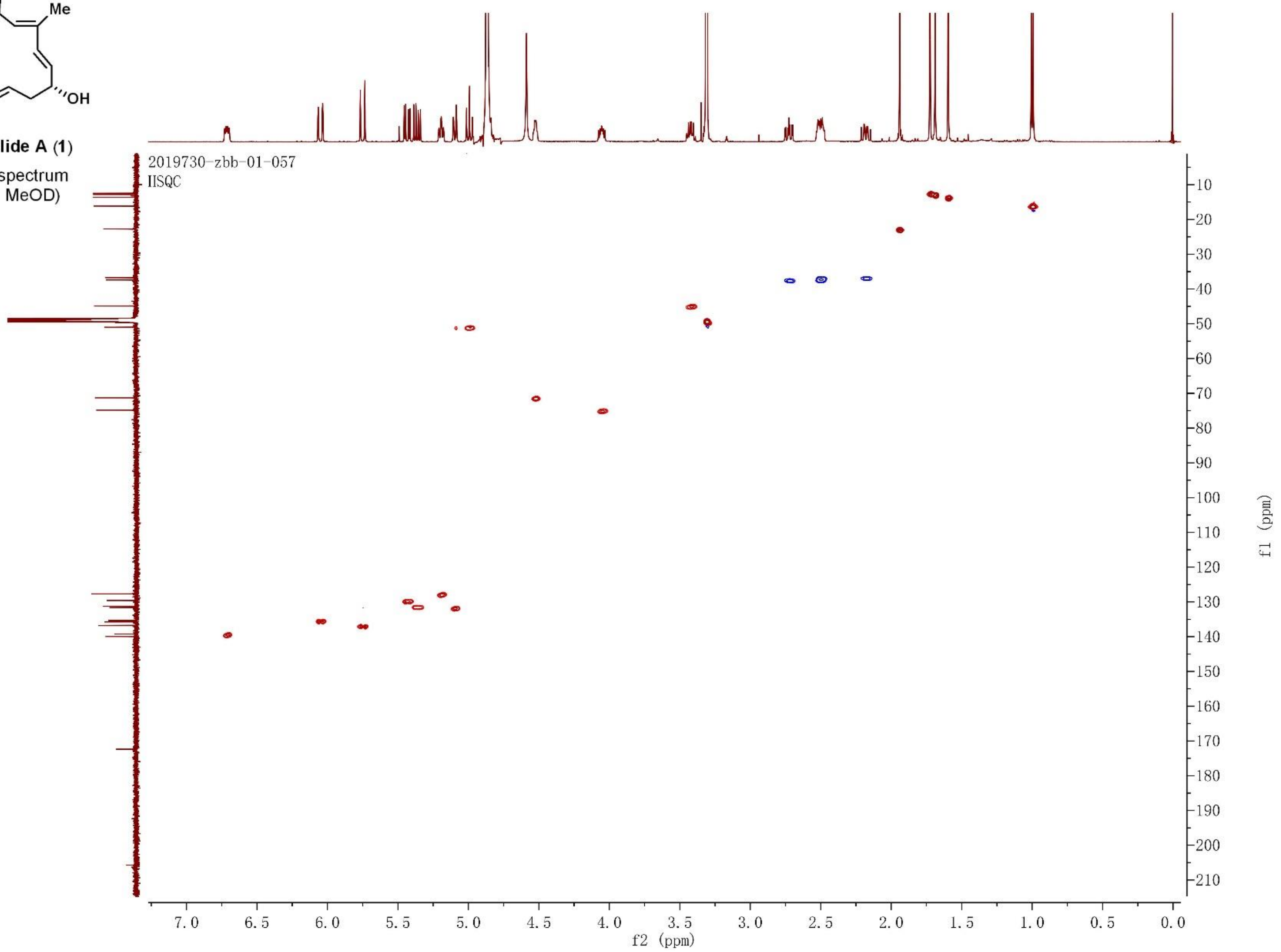

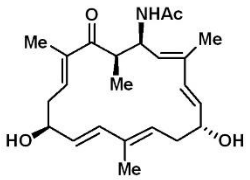

chejuenolide A (1)

C-H HMBC spectrum
(500 M Hz, MeOD)

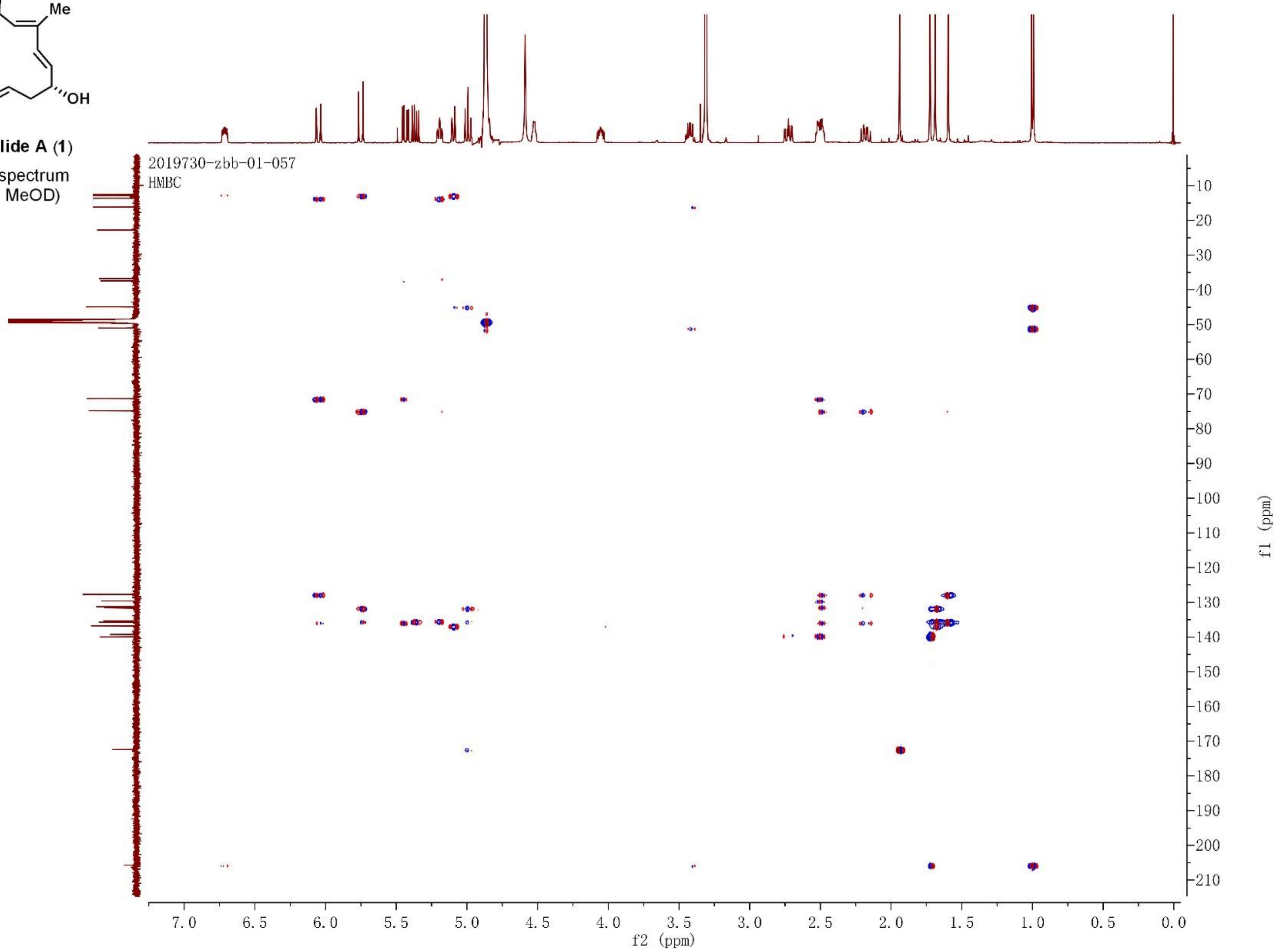

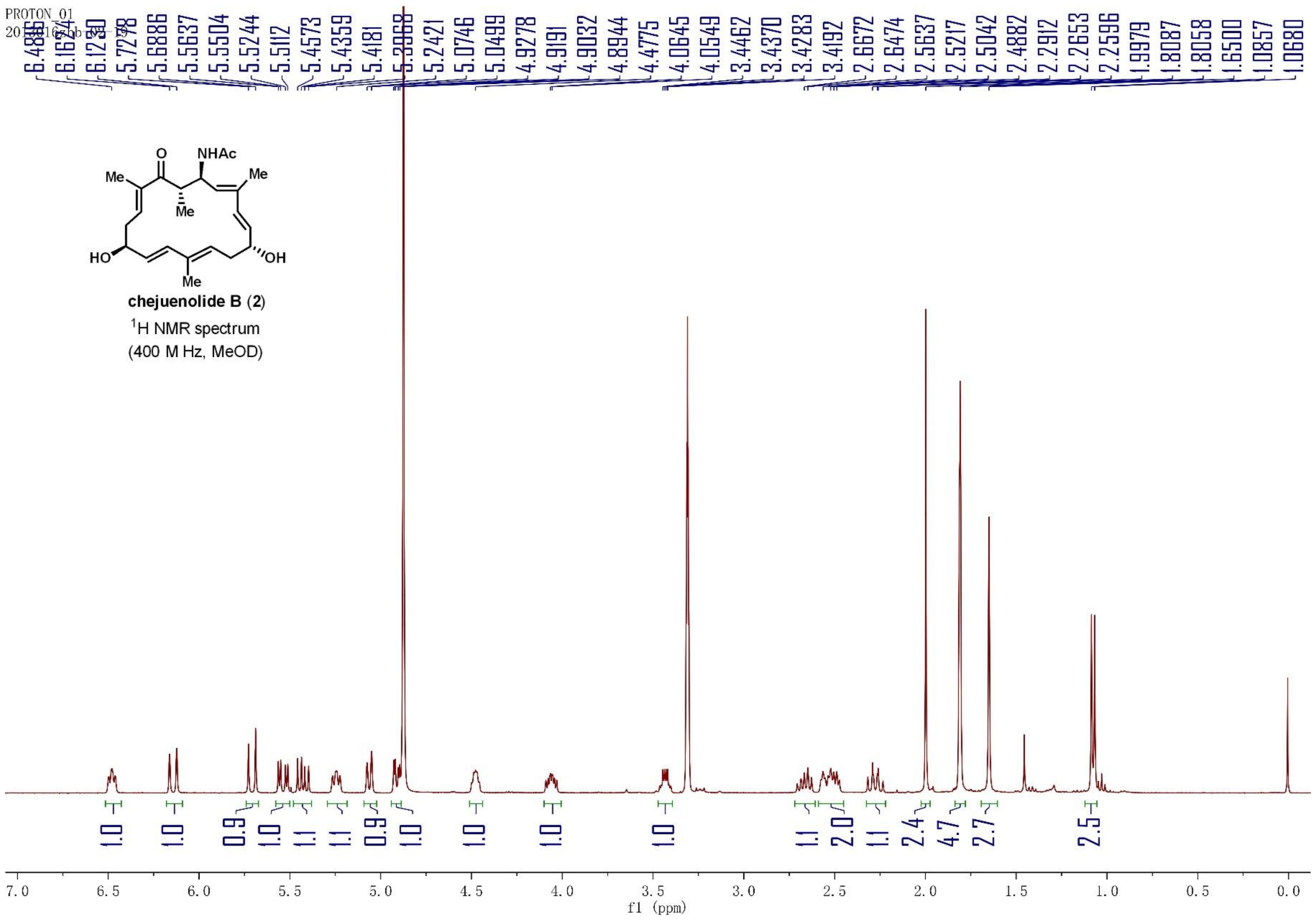
PROTON_01
6.4885
6.1624
6.1230
5.7278
5.6886
5.5637
5.5504
5.5244
5.5112
5.4573
5.4359
5.4181
5.3968
5.2421
5.0746
5.0499
4.9278
4.9191
4.9032
4.8944
4.4775
4.0645
4.0549
3.4462
3.4370
3.4283
3.4192
2.6672
2.6474
2.5637
2.5217
2.5042
2.4882
2.2912
2.2653
2.2596
1.9979
1.8087
1.8058
1.6500
1.0857
1.0680
O
NHAc
Me
Me
Me
HO
OH
Me
chejuenolide B (2)
1H NMR spectrum
(400 M Hz, MeOD)
1.0
1.0
0.9
1.0
1.1
1.1
0.9
1.0
1.0
1.0
1.0
1.1
2.0
1.1
2.4
4.7
2.7
2.5
7.0
6.5
6.0
5.5
5.0
4.5
4.0
3.5
3.0
2.5
2.0
1.5
1.0
0.5
0.0
f1 (ppm)

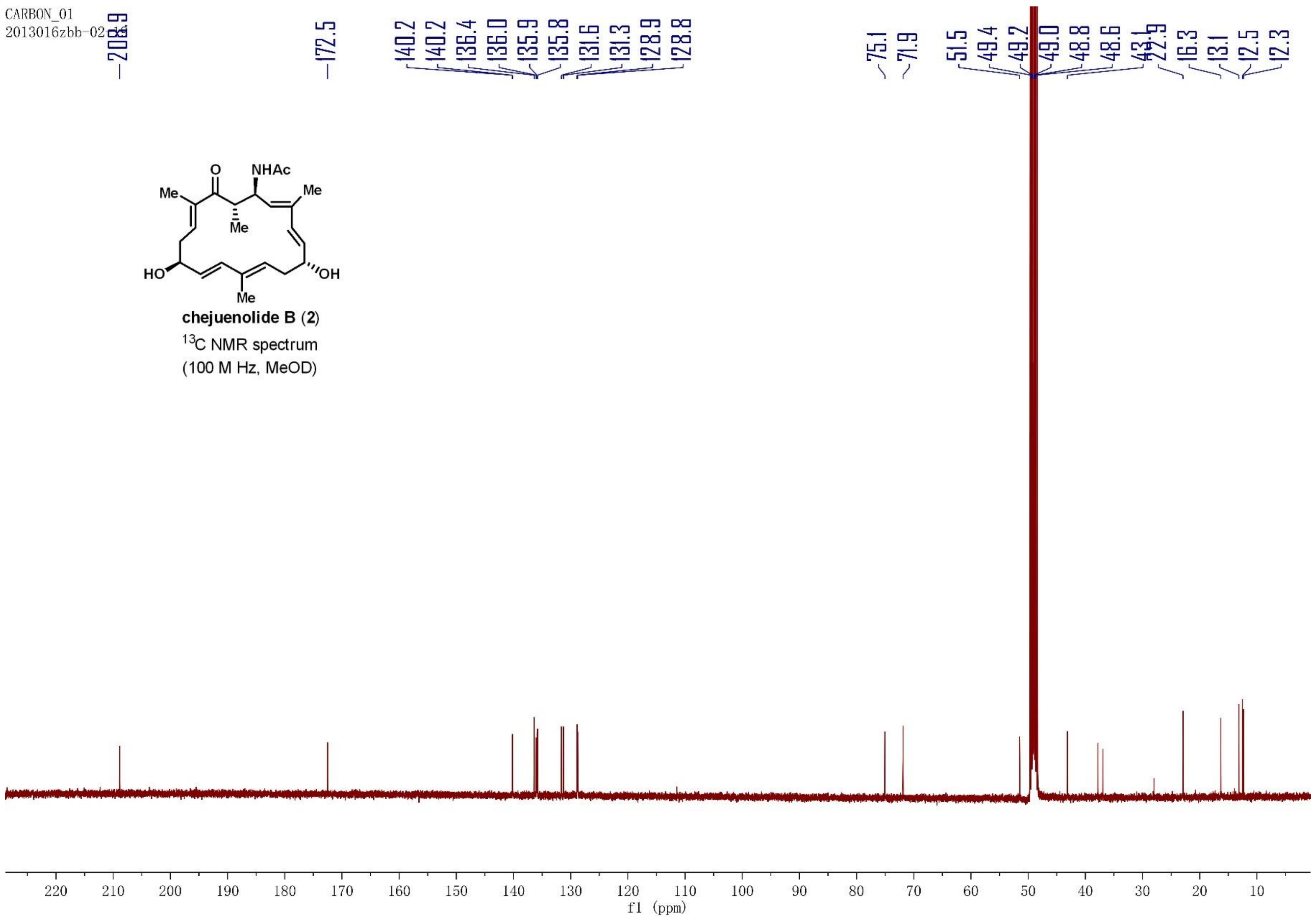

chejuenolide B (2)

^{13}C NMR spectrum

(100 M Hz, MeOD)

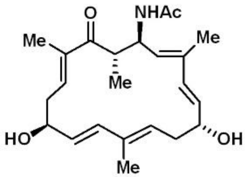

chejuenolide B (**2**)

H-H COSY spectrum
(400 M Hz, MeOD)

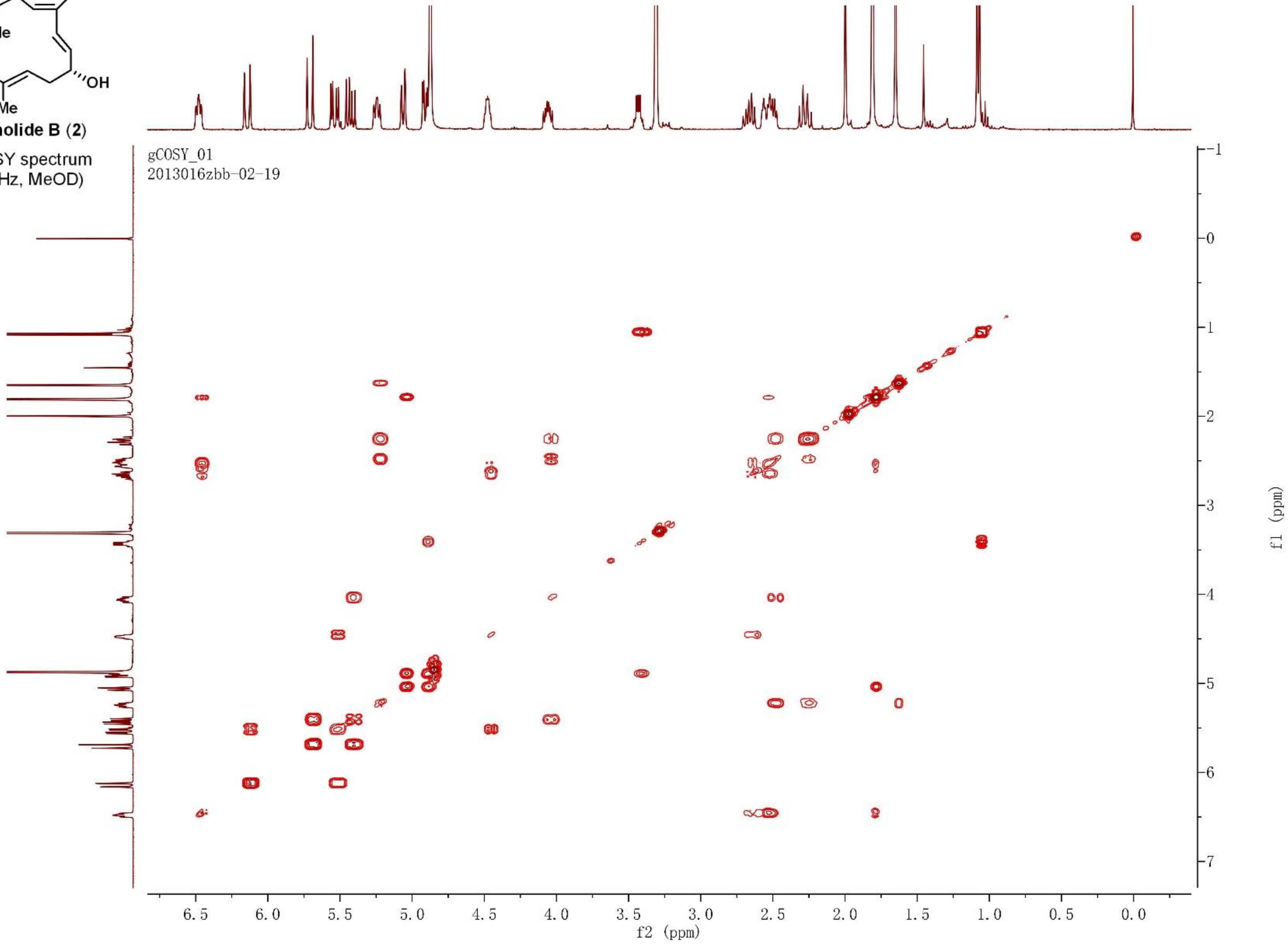

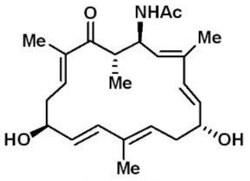

chejuenolide B (**2**)

NOESY spectrum
(400 M Hz, MeOD)

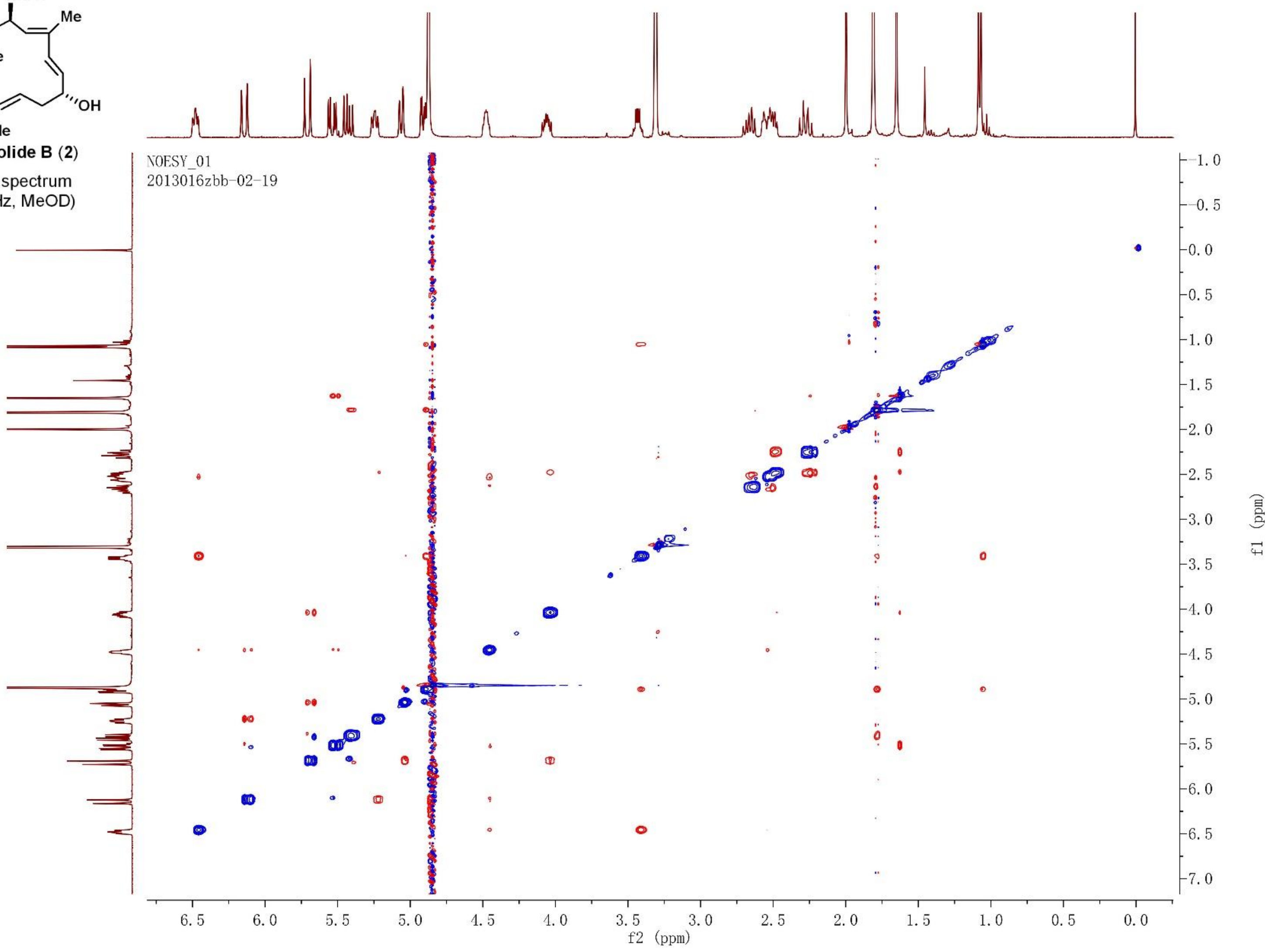

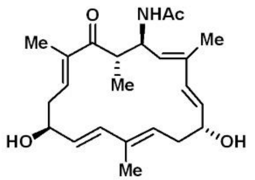

chejuenolide B (2)

C-H HSQC spectrum
(400 M Hz, MeOD)

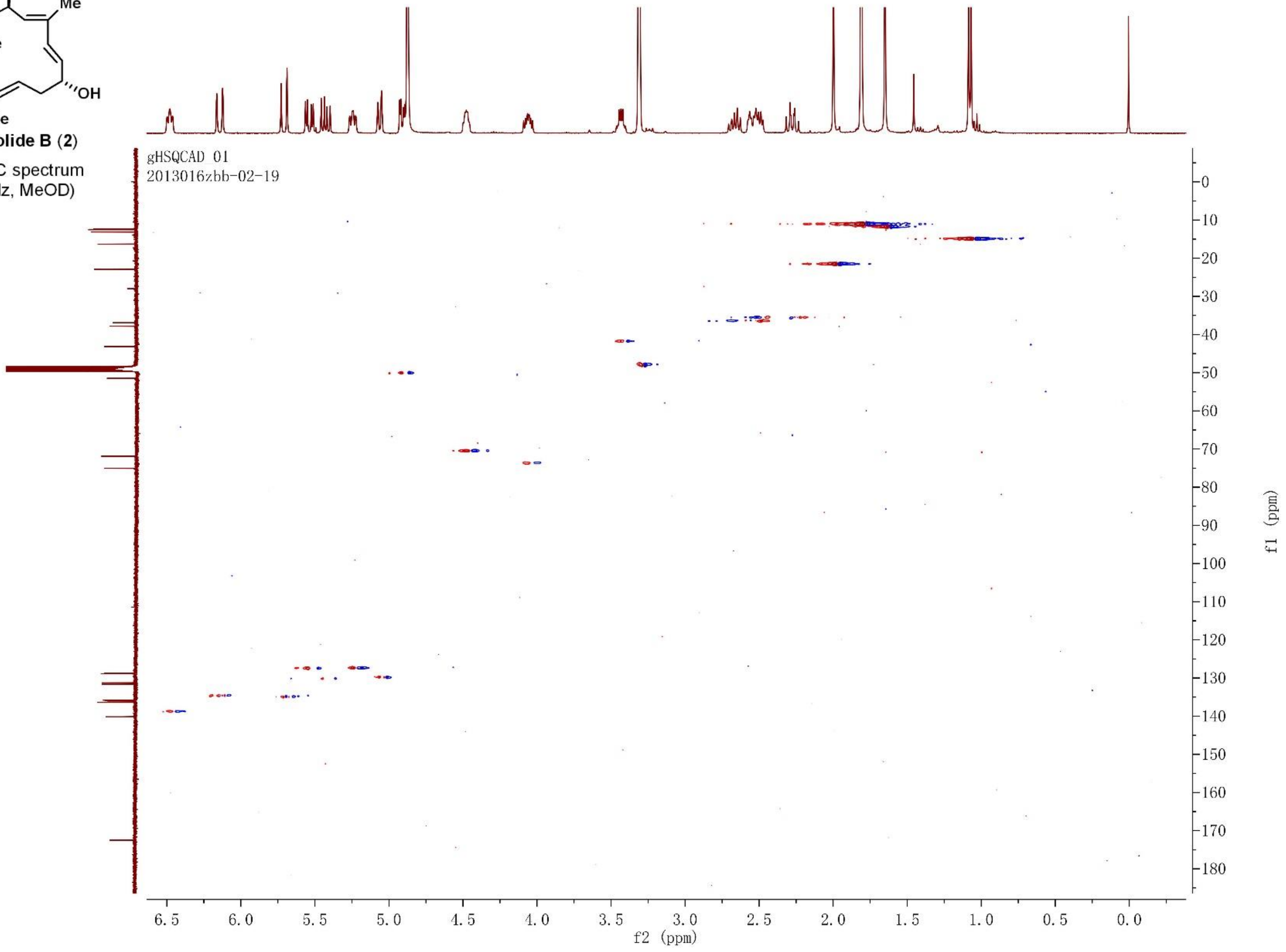

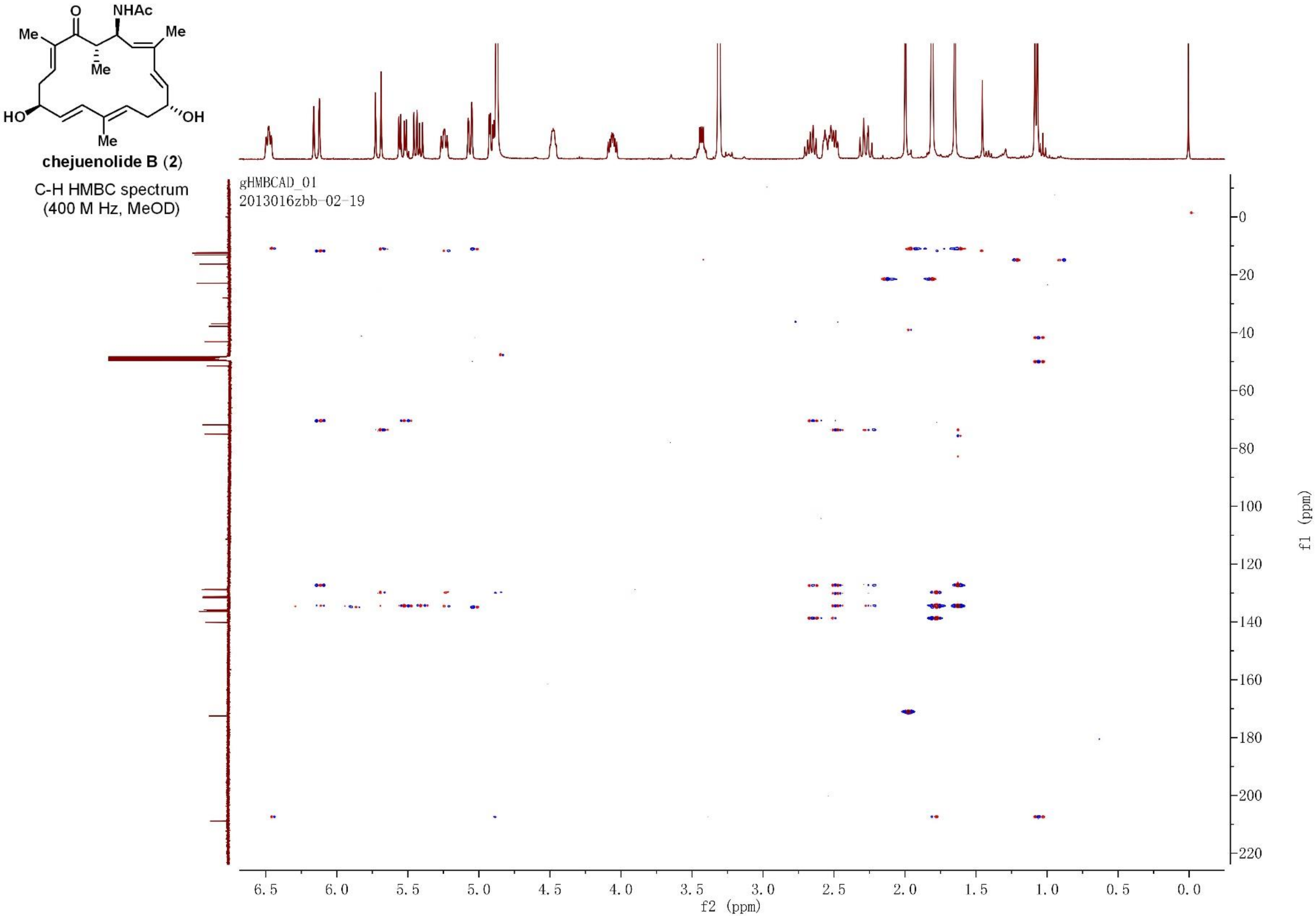

chejuenolide B (2)

C-H HMBC spectrum
(400 M Hz, MeOD)

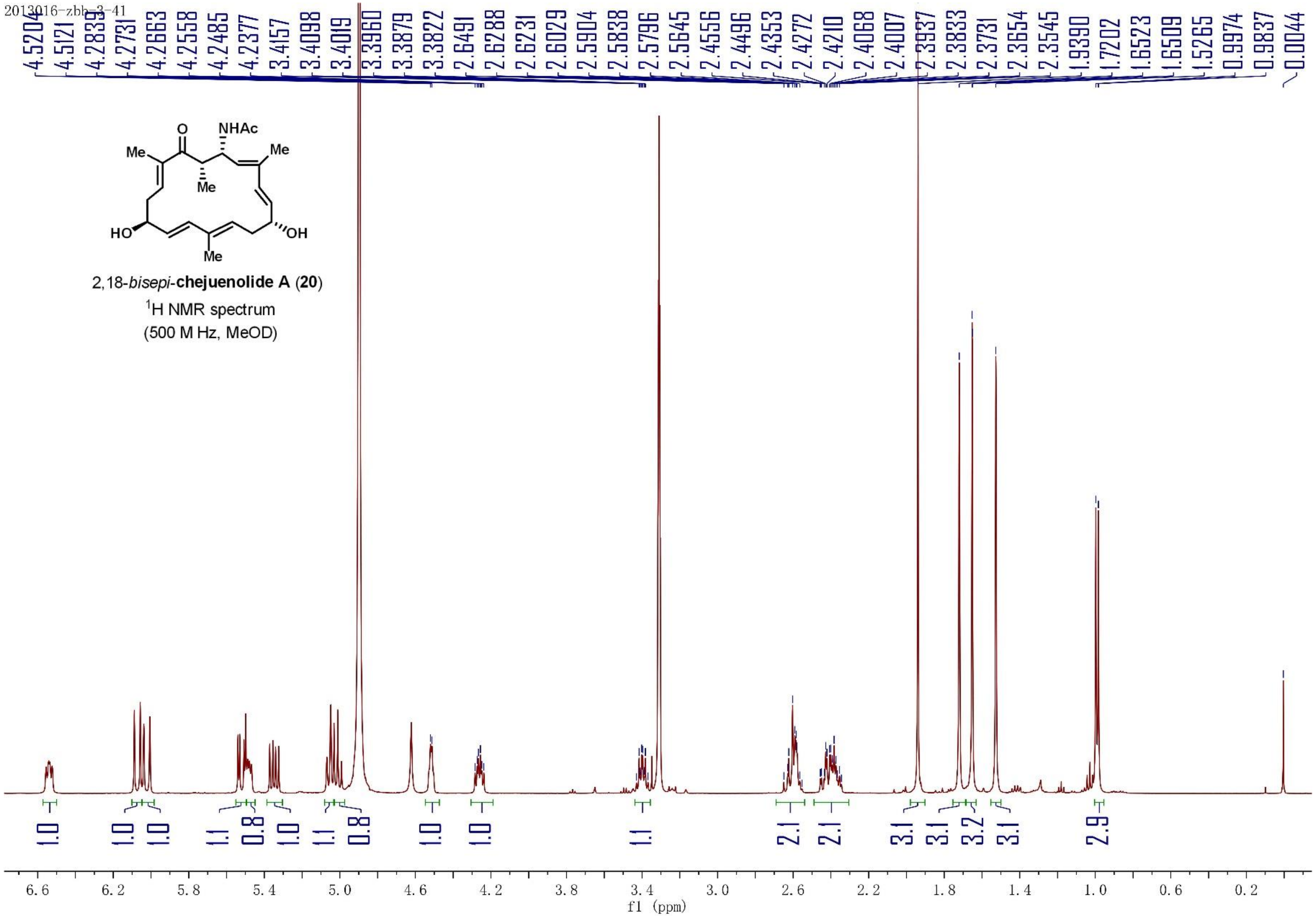

2,18-*bisepi*-**chejuenolide A** (**20**)

^{1}H NMR spectrum

(500 M Hz, MeOD)

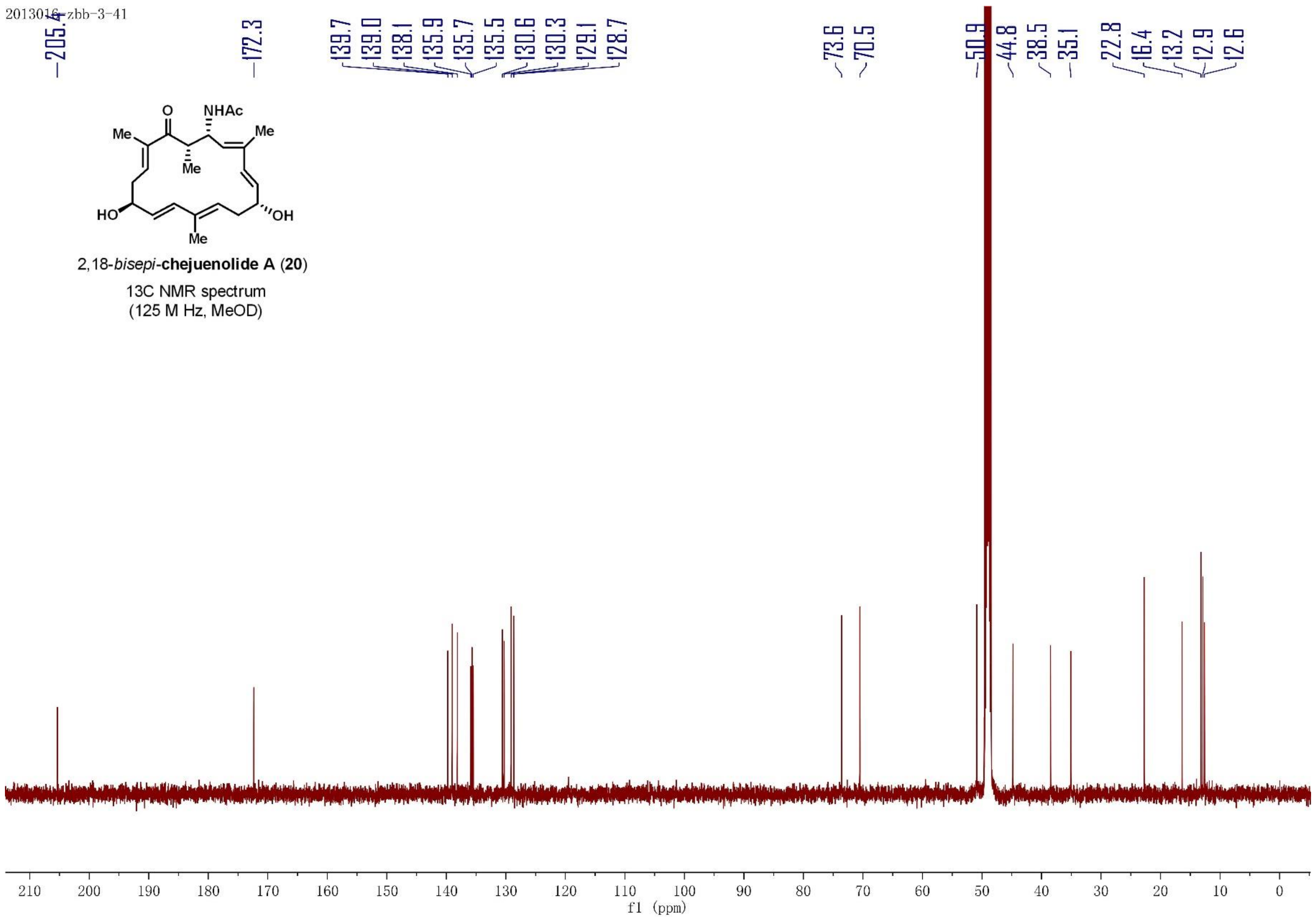
2013016-zbb-3-41
205.4
172.3
139.7
139.0
138.1
135.9
135.7
135.5
130.6
130.3
129.1
128.7
73.6
70.5
50.9
44.8
38.5
35.1
22.8
16.4
13.2
12.9
12.6
O
NHAc
Me
Me
Me
HO
OH
Me
2,18-*bisepi*-**chejuenolide A** (**20**)
13C NMR spectrum
(125 M Hz, MeOD)
210 200 190 180 170 160 150 140 130 120 110 100 90 80 70 60 50 40 30 20 10 0
f1 (ppm)

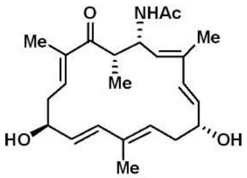

2,18-*bisepi*-**chejuenolide A** (**20**)

H-H COSY spectrum
(500 M Hz, MeOD)

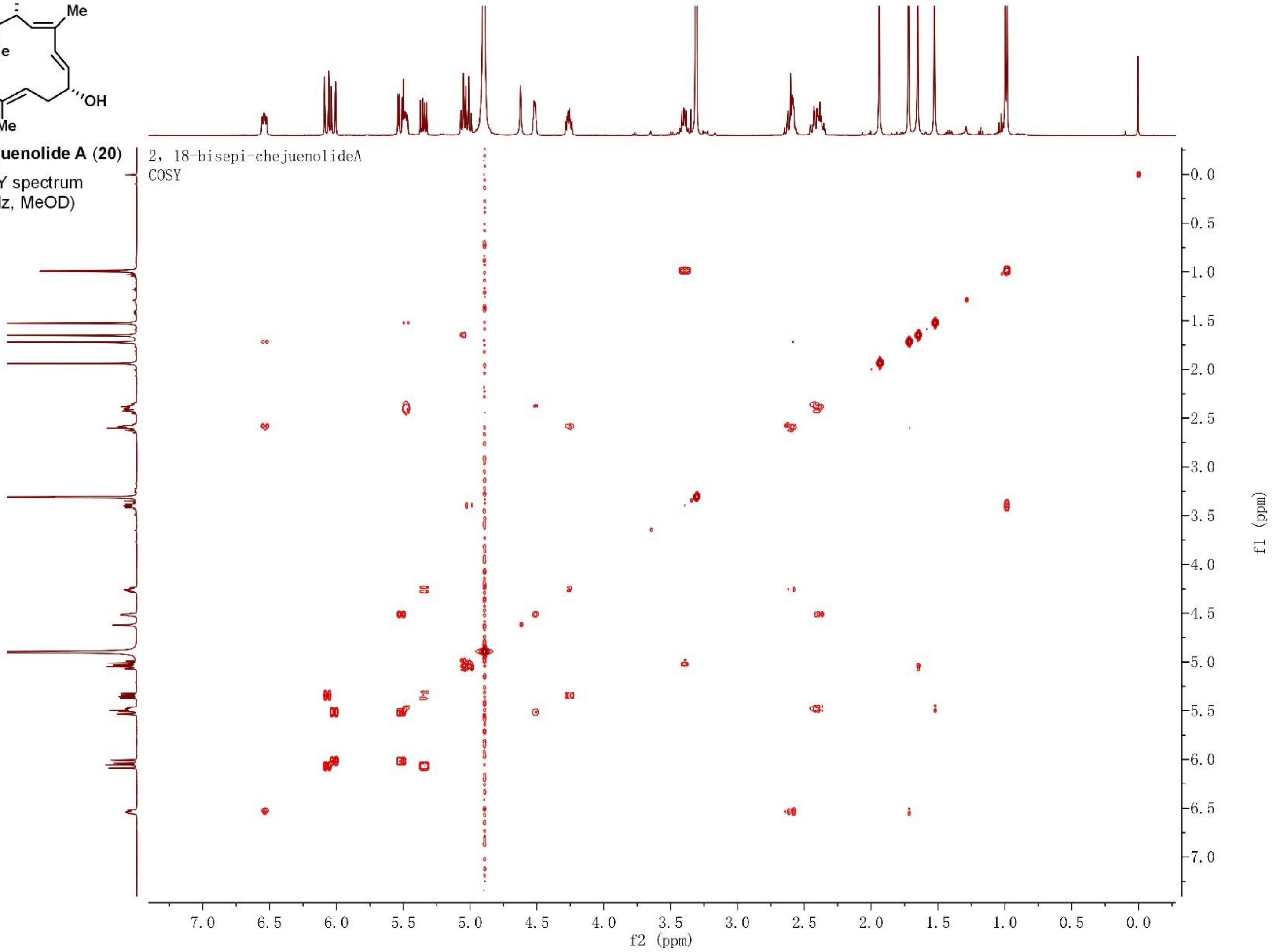

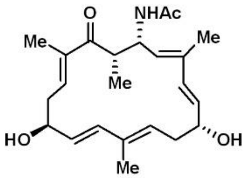

2,18-*bisepi*-**chejuenolide A** (**20**)

NOESY spectrum
(500 M Hz, MeOD)

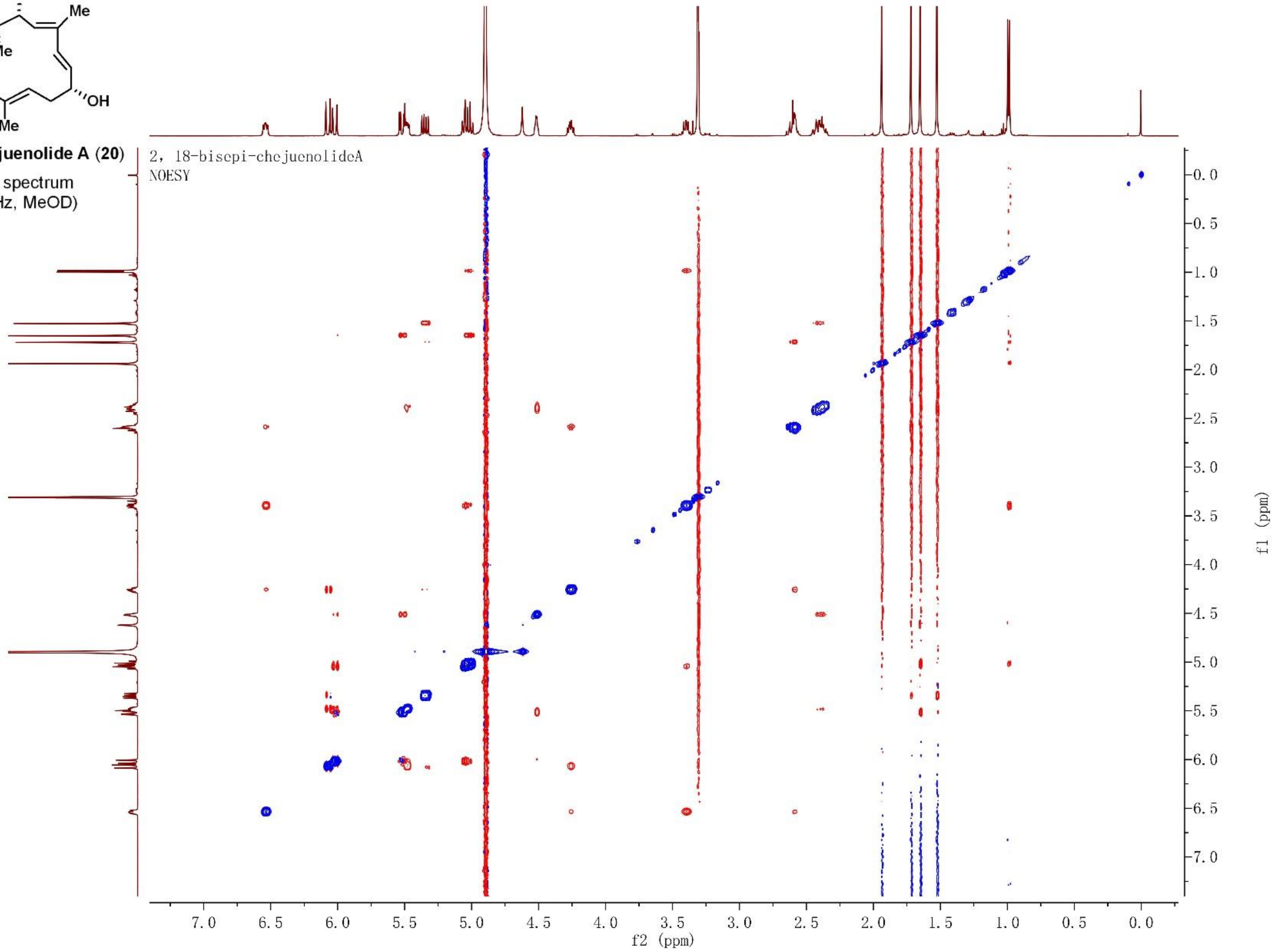

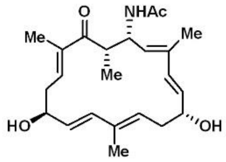

2,18-*bisepi*-**chejuenolide A** (**20**)

C-H HSQC spectrum
(500 M Hz, MeOD)

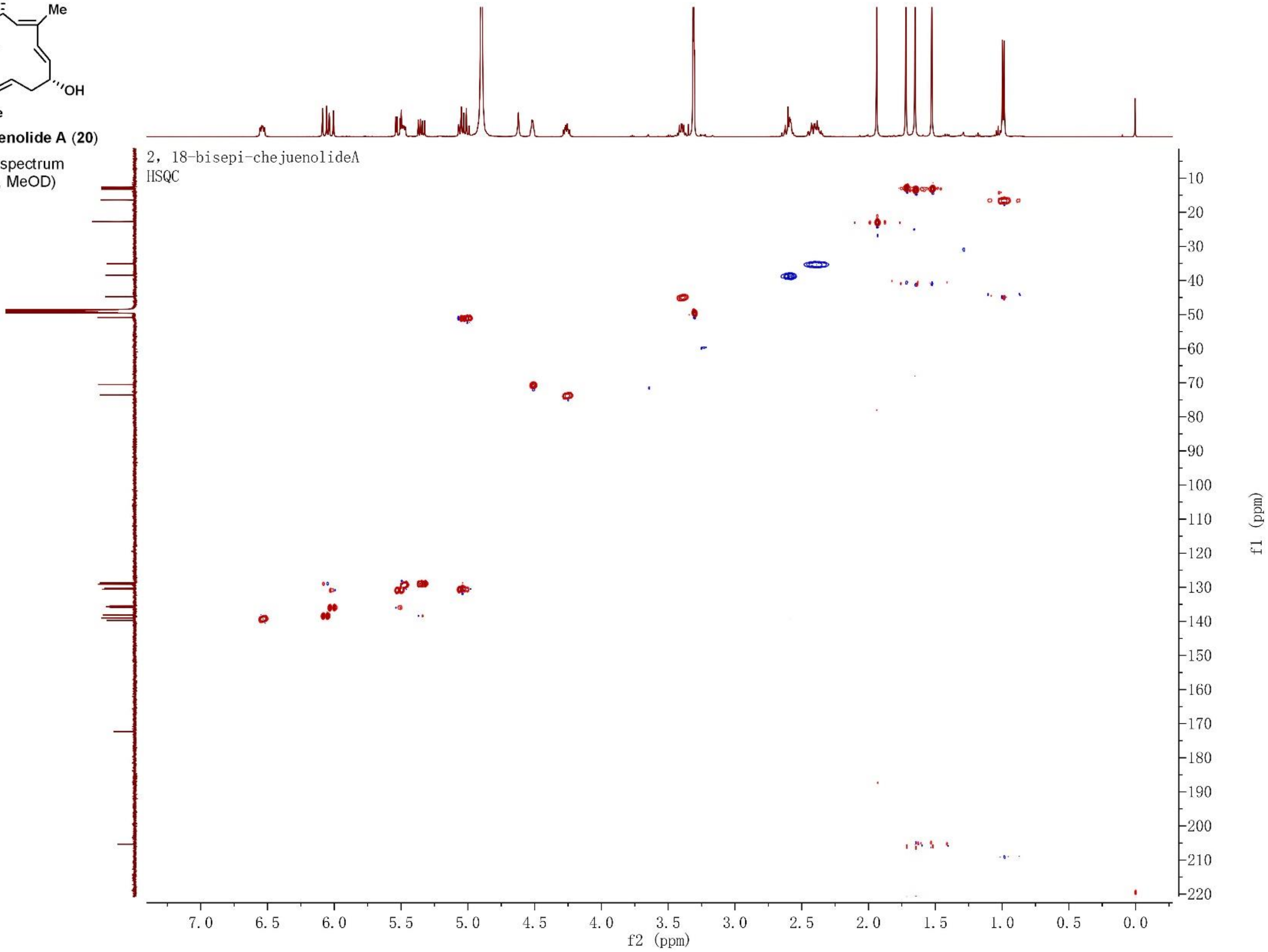

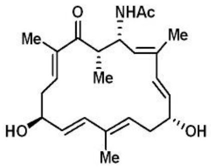

2,18-*bisepi*-**chejuenolide A** (**20**)

C-H HMBC spectrum
(500 M Hz, MeOD)

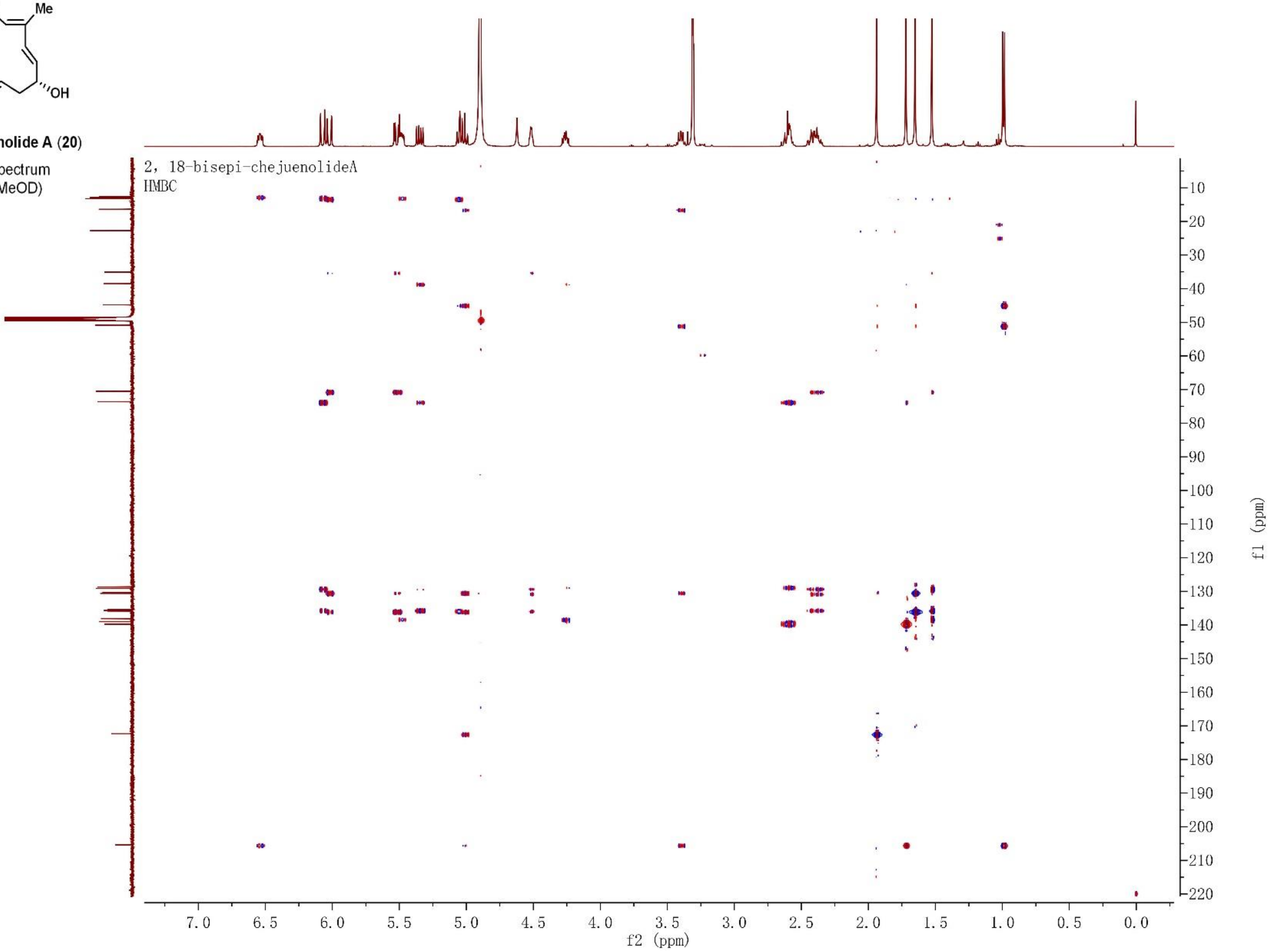

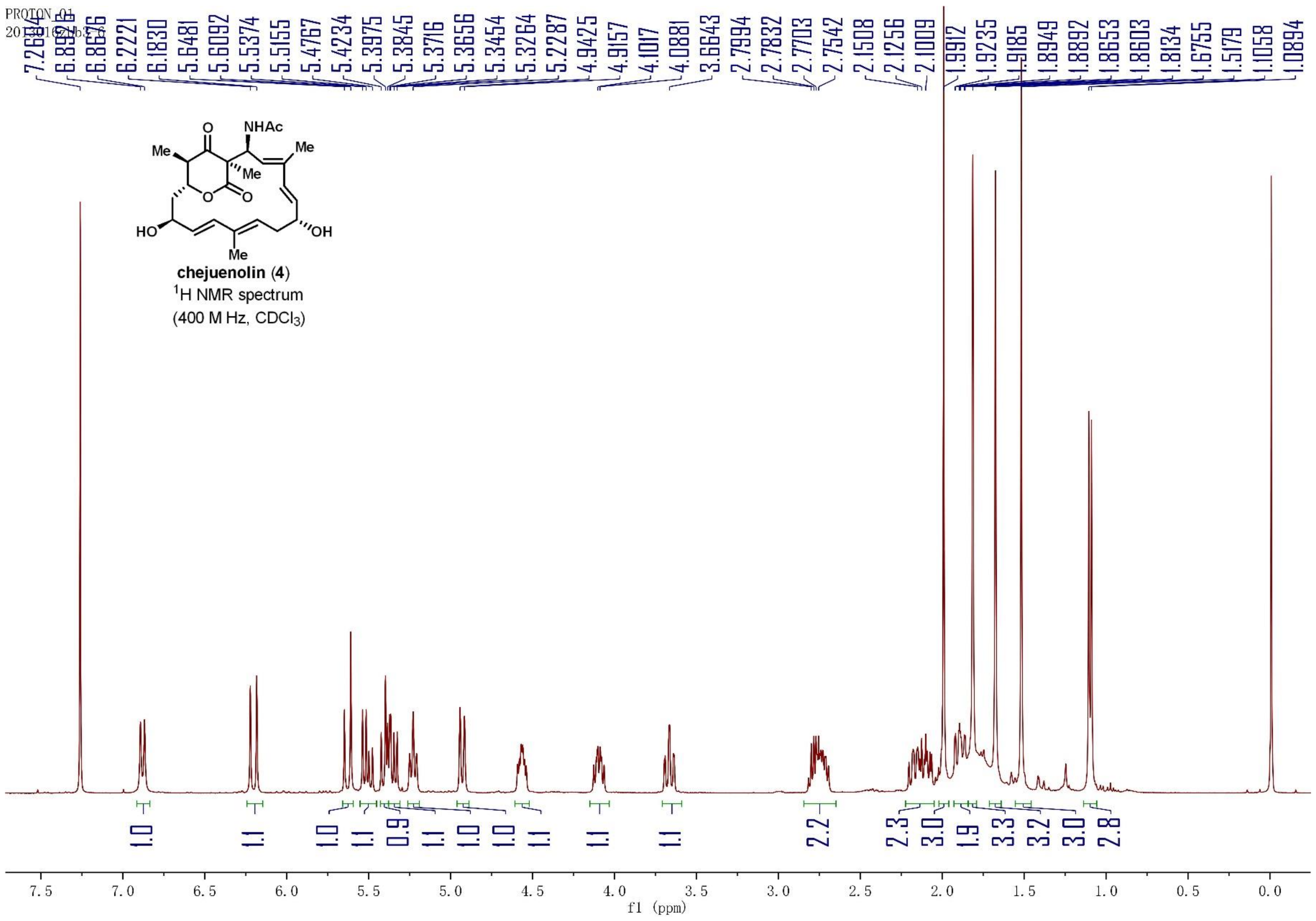

chejuenolin (4)
1H NMR spectrum
(400 M Hz, CDCl3)
f1 (ppm)

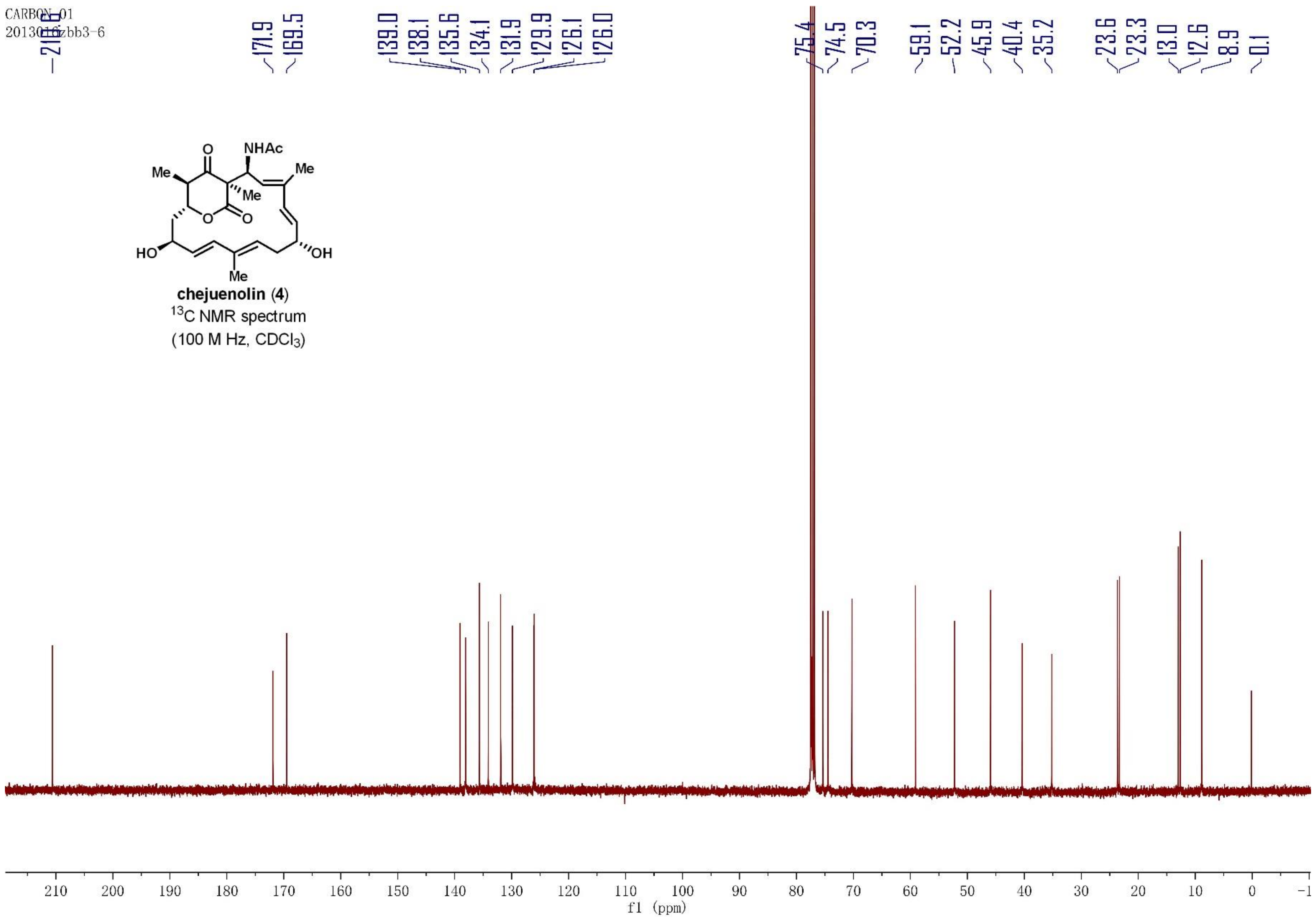
CARBON_01
20130116zbb3-6
210.6
171.9
169.5
139.0
138.1
135.6
134.1
131.9
129.9
126.1
126.0
75.4
74.5
70.3
59.1
52.2
45.9
40.4
35.2
23.6
23.3
13.0
12.6
8.9
0.1
NHAc
Me
Me
Me
O
O
O
HO
OH
Me
chejuenolin (4)
^{13}C NMR spectrum
(100 M Hz, $CDCl_3$)
210 200 190 180 170 160 150 140 130 120 110 100 90 80 70 60 50 40 30 20 10 0 -1
f1 (ppm)

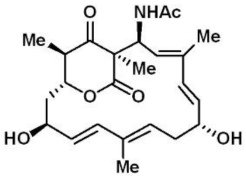

chejuenolin (4)
H-H COSY spectrum
(400 M Hz, $CDCl_3$)

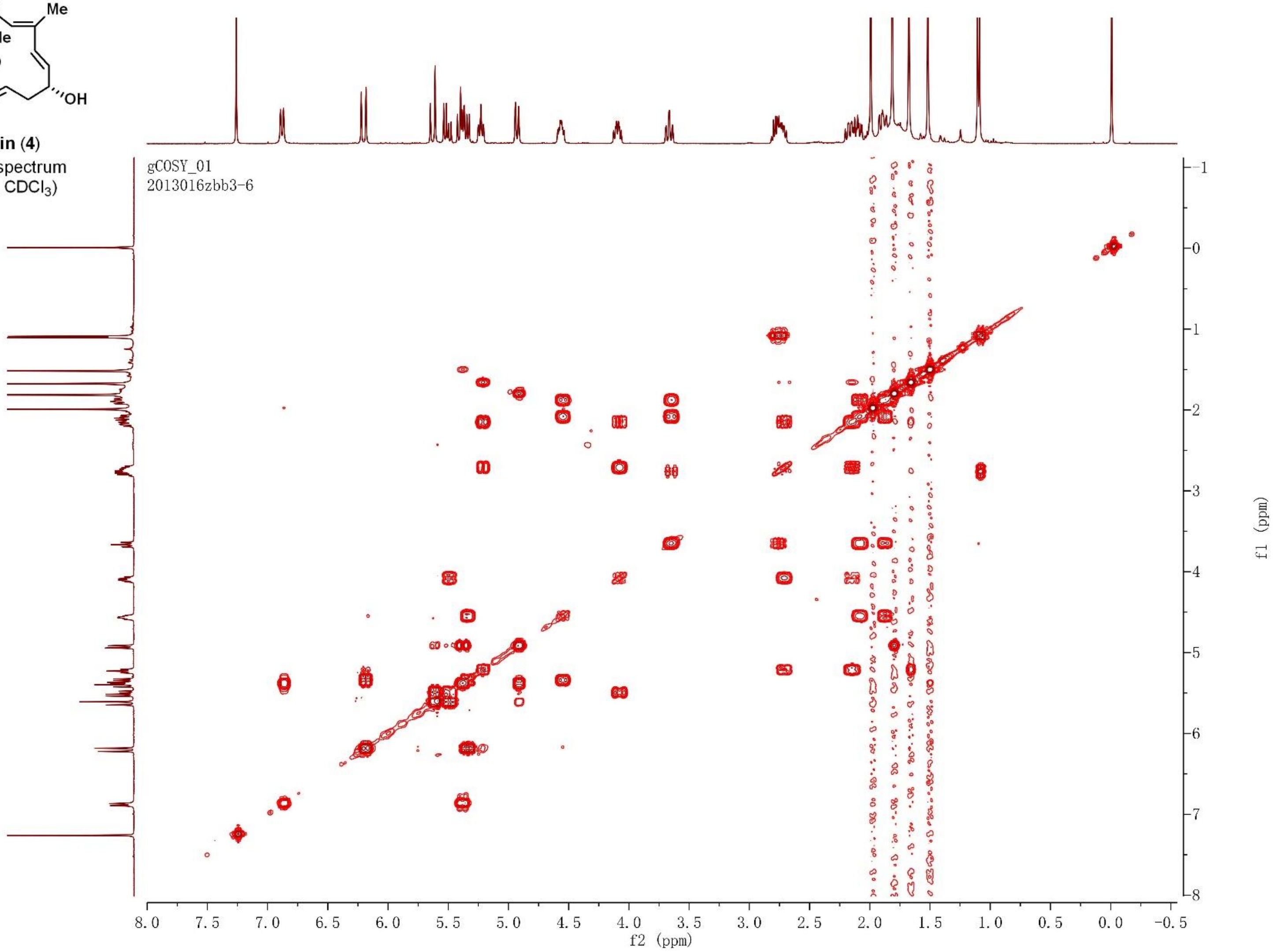

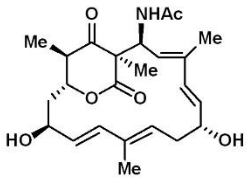

chejuenolin (4)
NOESY spectrum
(400 M Hz, $CDCl_3$)

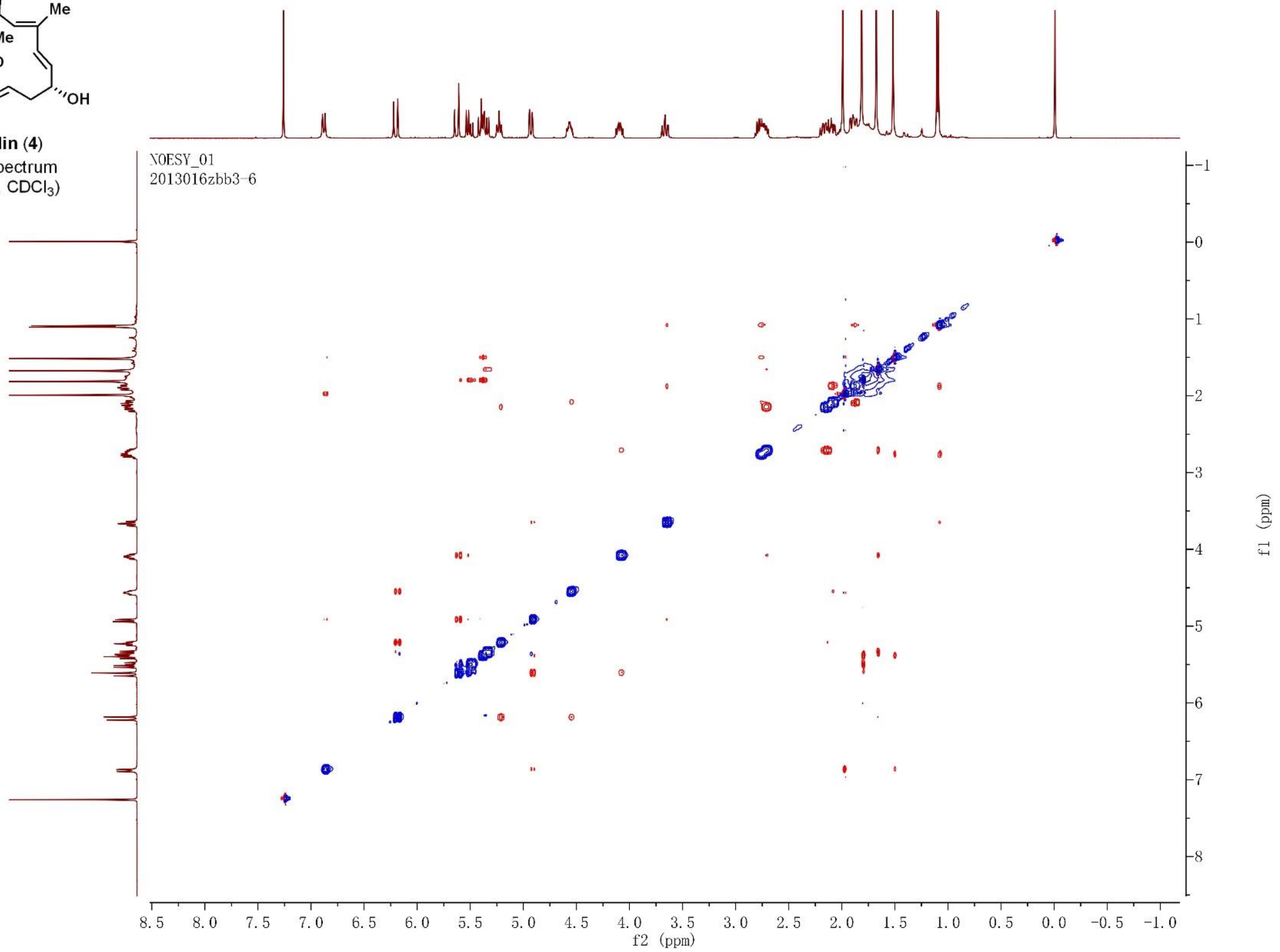

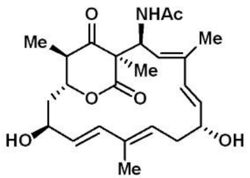

chejuenolin (4)
C-H HSQC spectrum
(400 M Hz, $CDCl_3$)

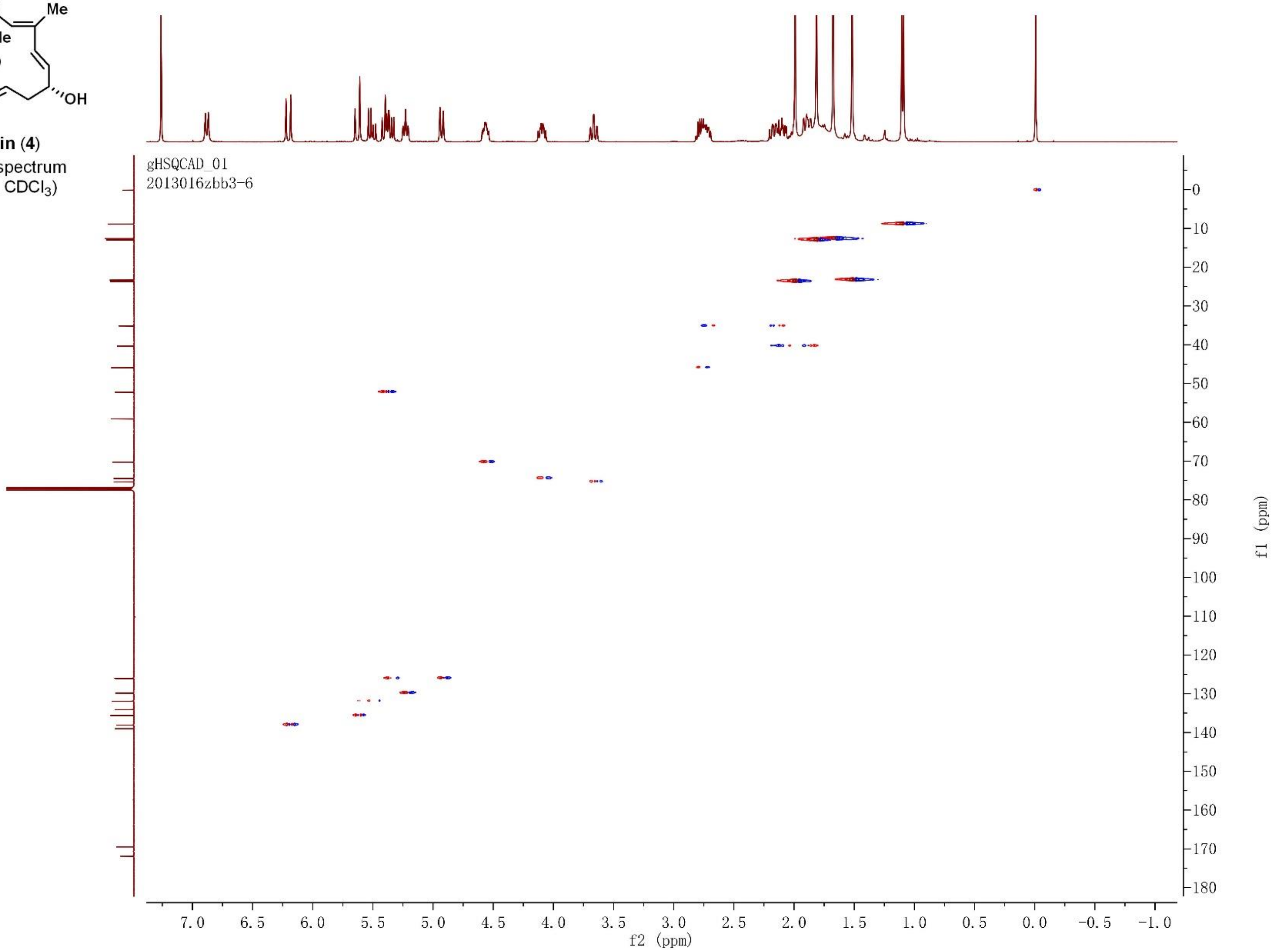

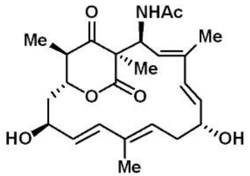

chejuenolin (4)
C-H HMBC spectrum
(400 M Hz, $CDCl_3$)

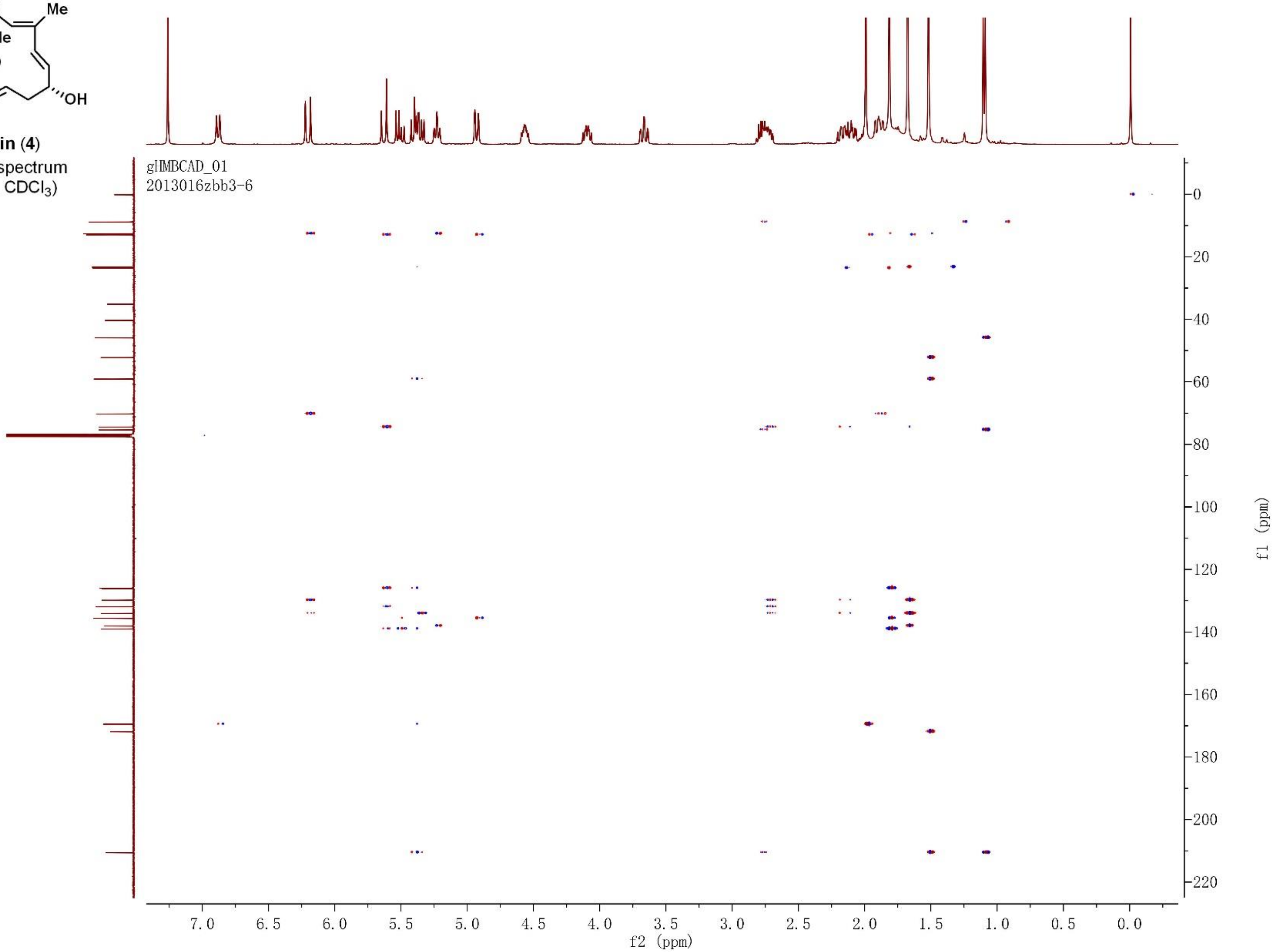

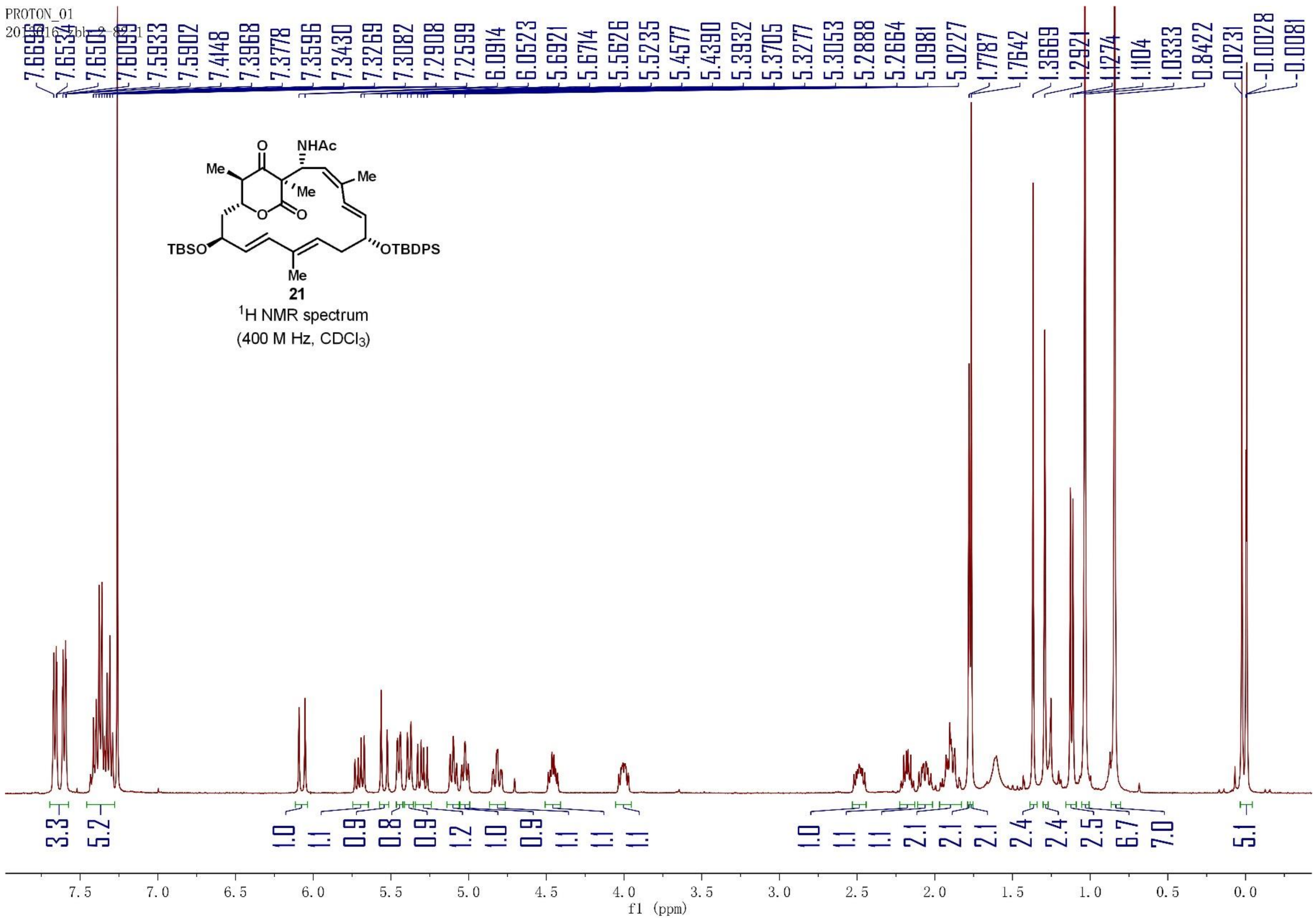

21

^{1}H NMR spectrum

(400 M Hz, $CDCl_3$)

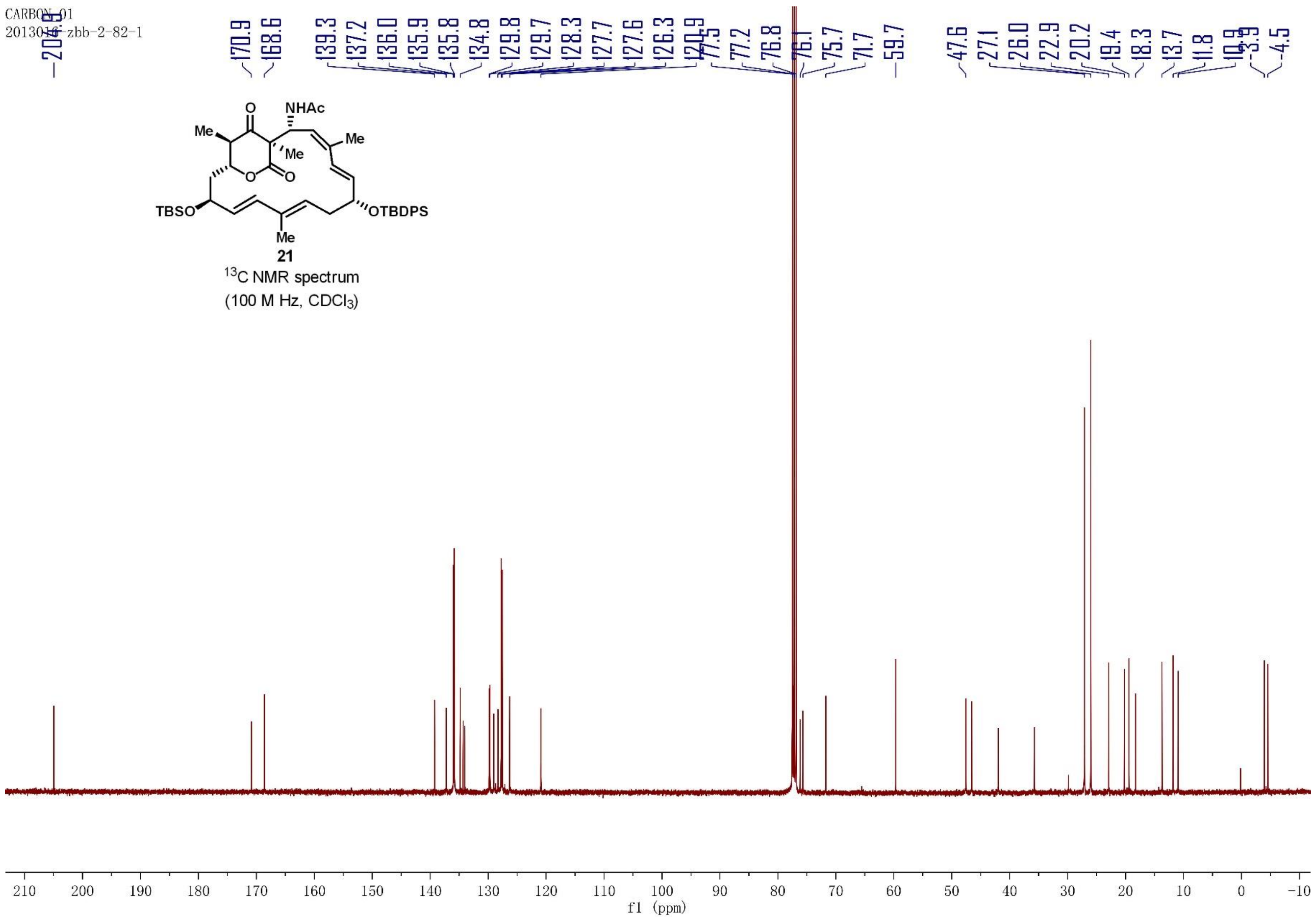
CARBON_01
2013016-zbb-2-82-1
204.9
170.9
168.6
139.3
137.2
136.0
135.9
135.8
134.8
129.8
129.7
128.3
127.7
127.6
126.3
120.9
77.5
77.2
76.8
76.1
75.7
71.7
59.7
47.6
27.1
26.0
22.9
20.2
19.4
18.3
13.7
11.8
10.9
-3.9
-4.5
NHAc
Me
TBSO
OTBDPS
21
^{13}C NMR spectrum
(100 M Hz, CDCl$_3$)
210
200
190
180
170
160
150
140
130
120
110
100
90
80
70
60
50
40
30
20
10
0
−10
f1 (ppm)

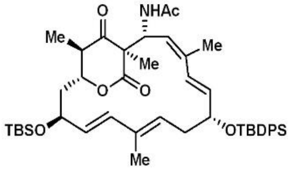

21

H-H COSY spectrum
(400 M Hz, $CDCl_3$)

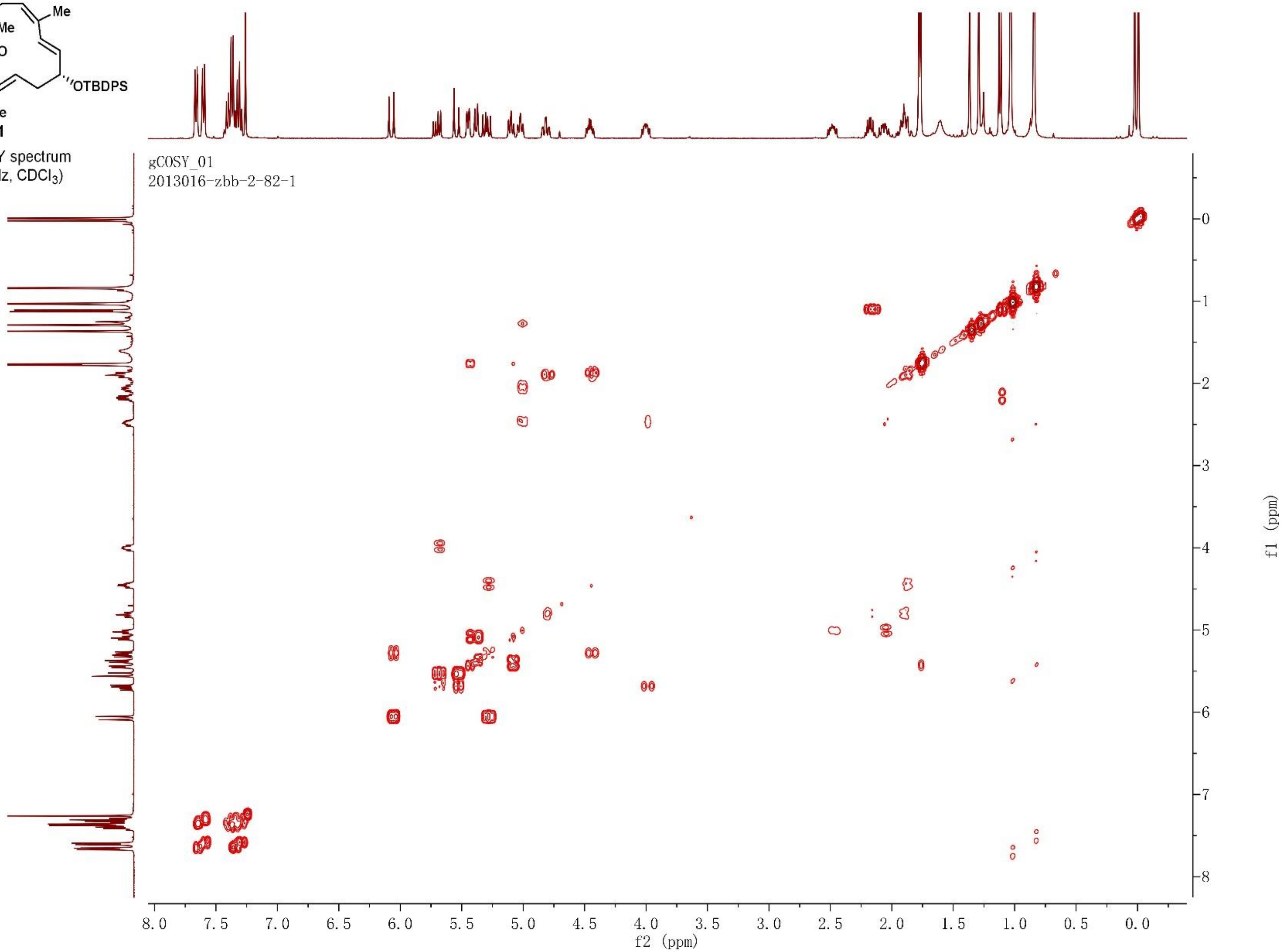

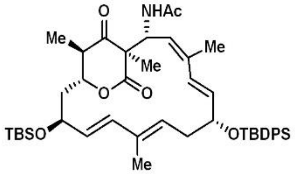

21

NOESY spectrum
(400 M Hz, $CDCl_3$)

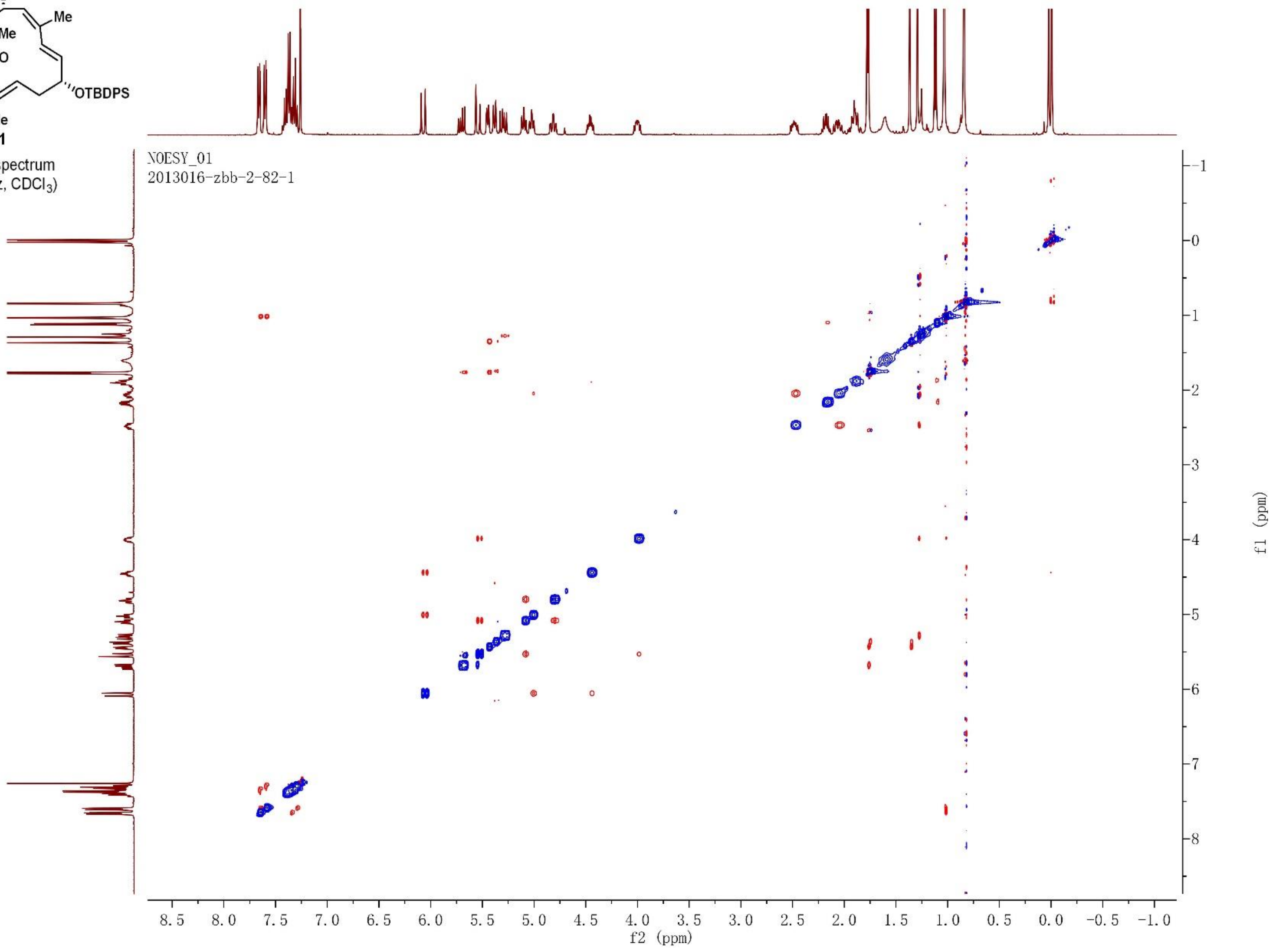

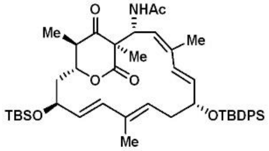

21

C-H HSQC spectrum

(400 M Hz, $CDCl_3$)

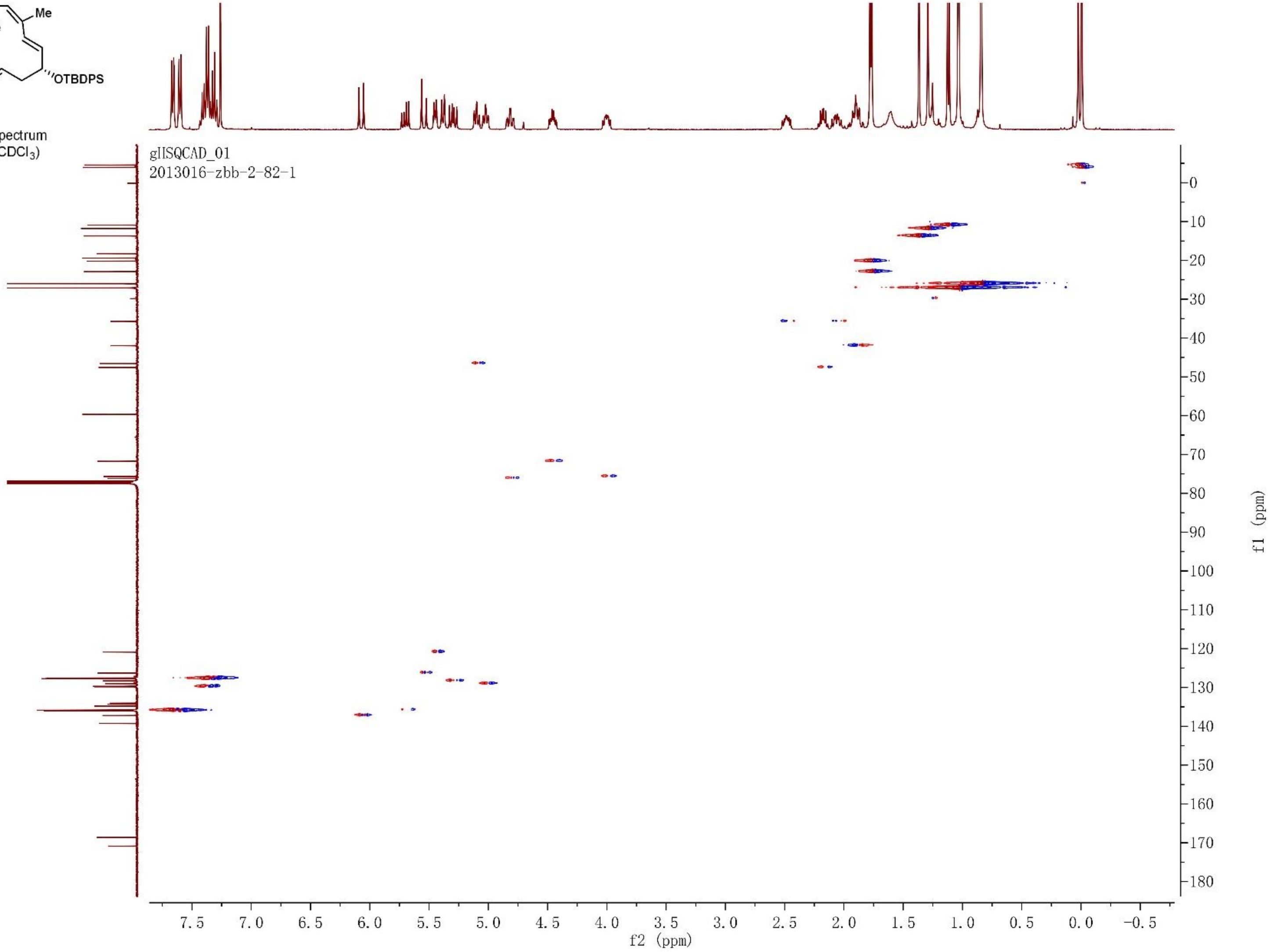

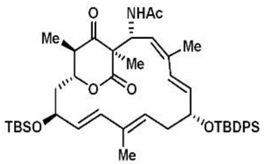

21

C-H HMBC spectrum
(400 M Hz, $CDCl_3$)

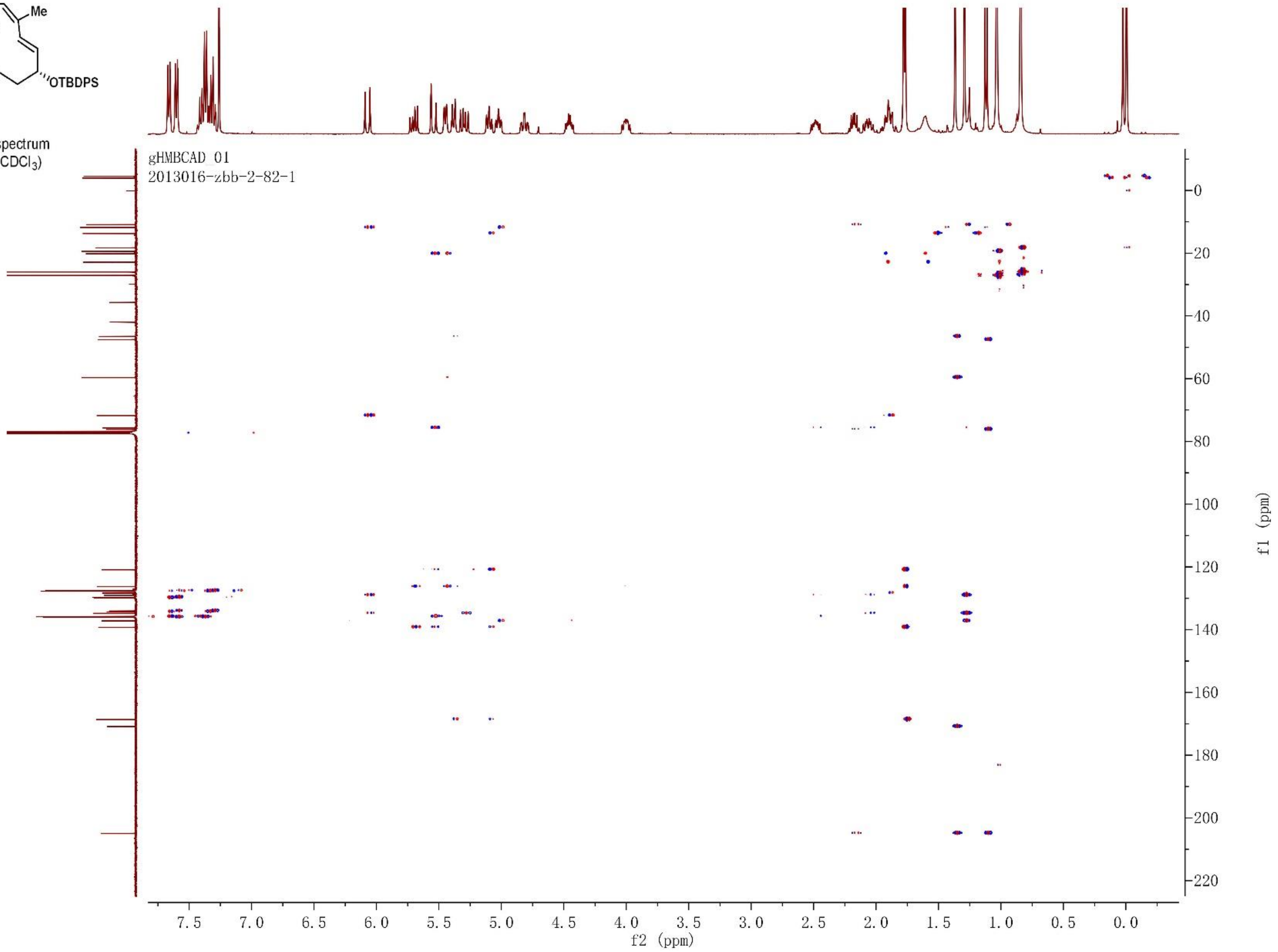

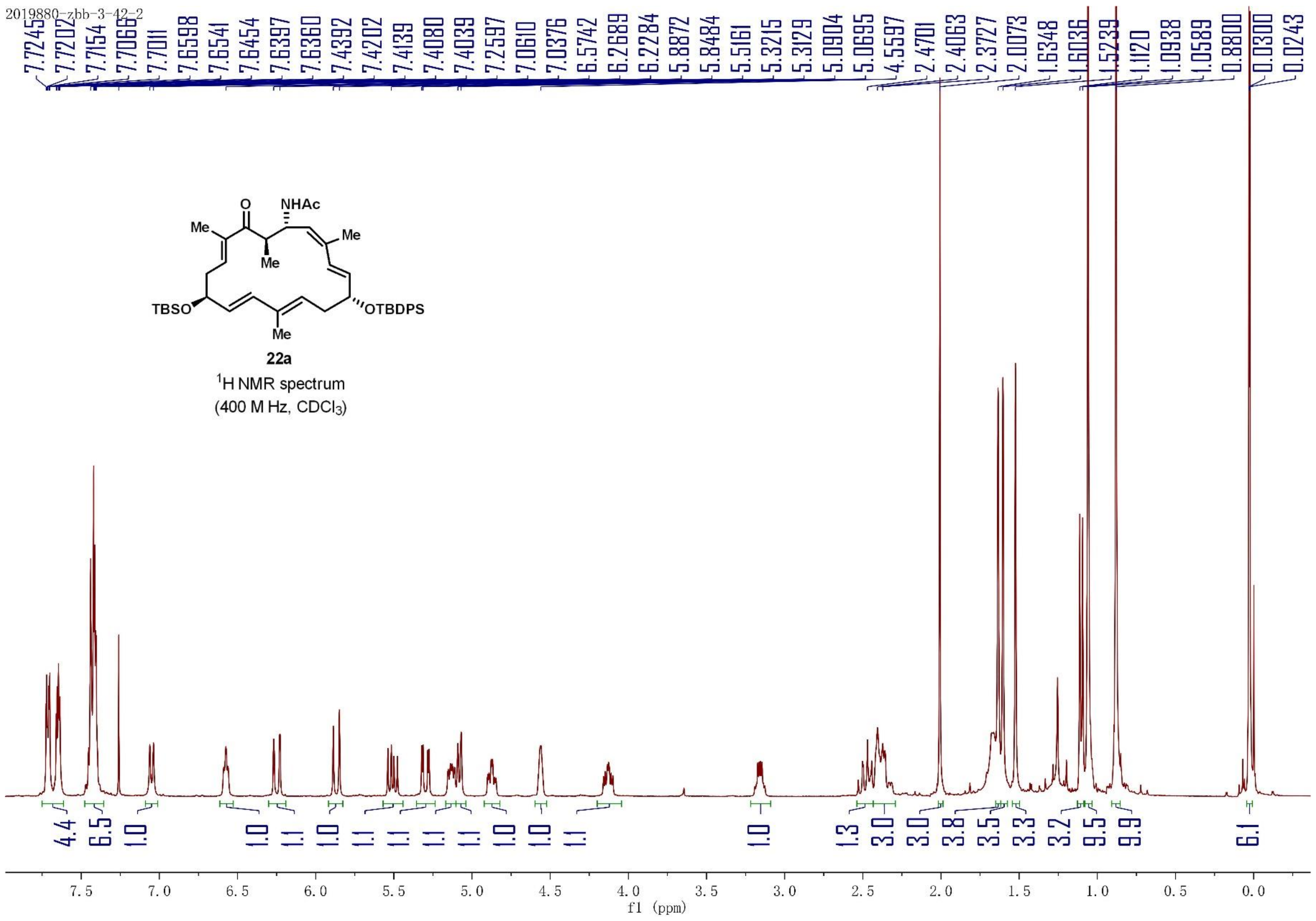
2019880-zbb-3-42-2
7.7245
7.7202
7.7154
7.7066
7.7011
7.6598
7.6541
7.6454
7.6397
7.6360
7.4392
7.4202
7.4139
7.4080
7.4039
7.2597
7.0610
7.0376
6.5742
6.2689
6.2284
5.8872
5.8484
5.5161
5.3215
5.3129
5.0904
5.0695
4.5597
2.4701
2.4063
2.3727
2.0073
1.6348
1.6036
1.5239
1.1120
1.0938
1.0589
0.8800
0.0300
0.0243
O
NHAc
Me
Me
Me
TBSO
OTBDPS
Me
22a
^{1}H NMR spectrum
(400 M Hz, CDCl$_3$)
4.4
6.5
1.0
1.0
1.1
1.0
1.1
1.1
1.1
1.1
1.0
1.0
1.1
1.0
1.3
3.0
3.0
3.8
3.5
3.3
3.2
9.5
9.9
6.1
f1 (ppm)

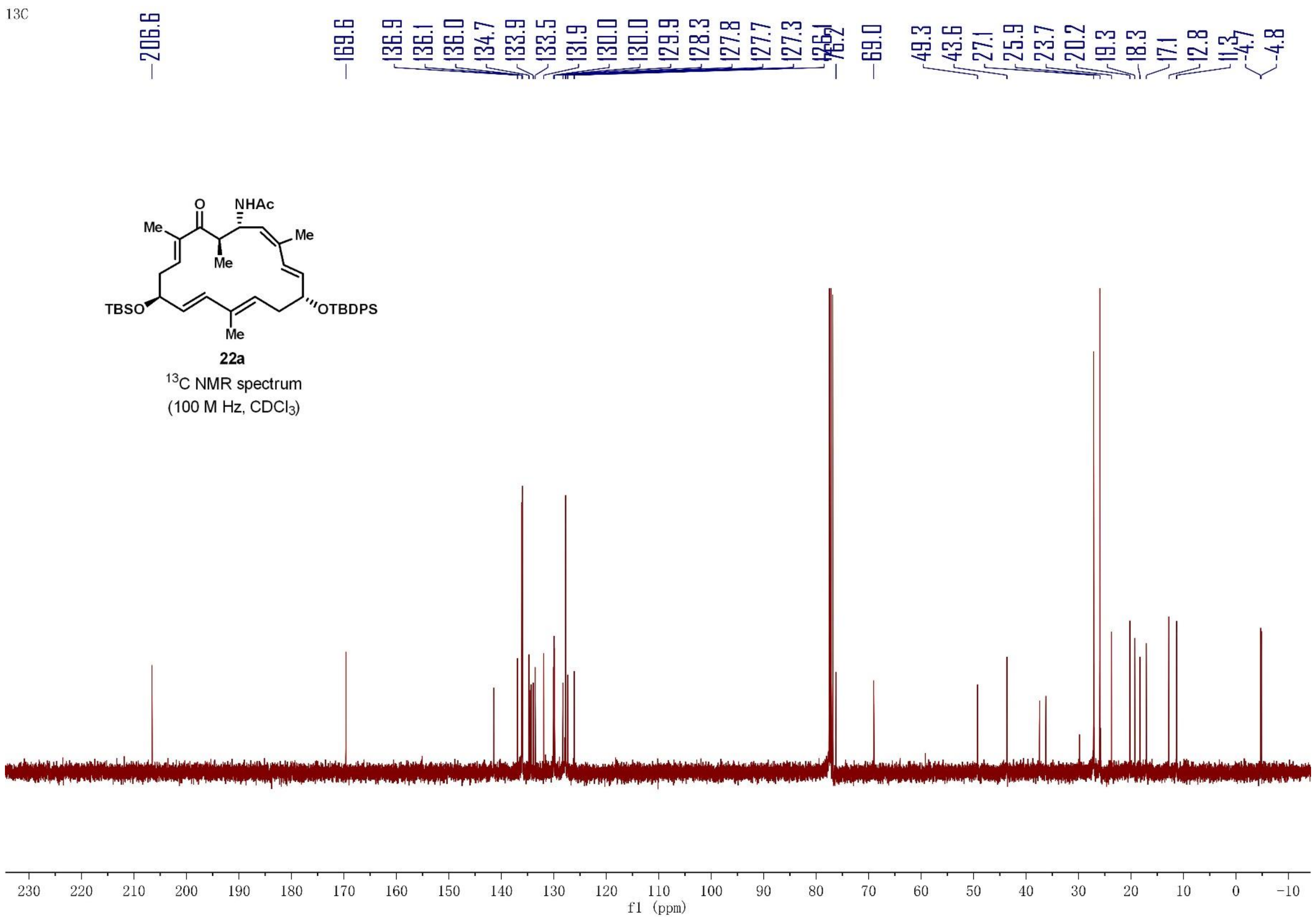
13C
206.6
169.6
136.9
136.1
136.0
134.7
133.9
133.5
131.9
130.0
130.0
129.9
128.3
127.8
127.7
127.3
69.0
49.3
43.6
27.1
25.9
23.7
20.2
19.3
18.3
17.1
12.8
11.3
-4.7
-4.8
O
NHAc
Me
Me
Me
TBSO
OTBDPS
Me
22a
13C NMR spectrum
(100 M Hz, CDCl3)
230 220 210 200 190 180 170 160 150 140 130 120 110 100 90 80 70 60 50 40 30 20 10 0 -10
f1 (ppm)

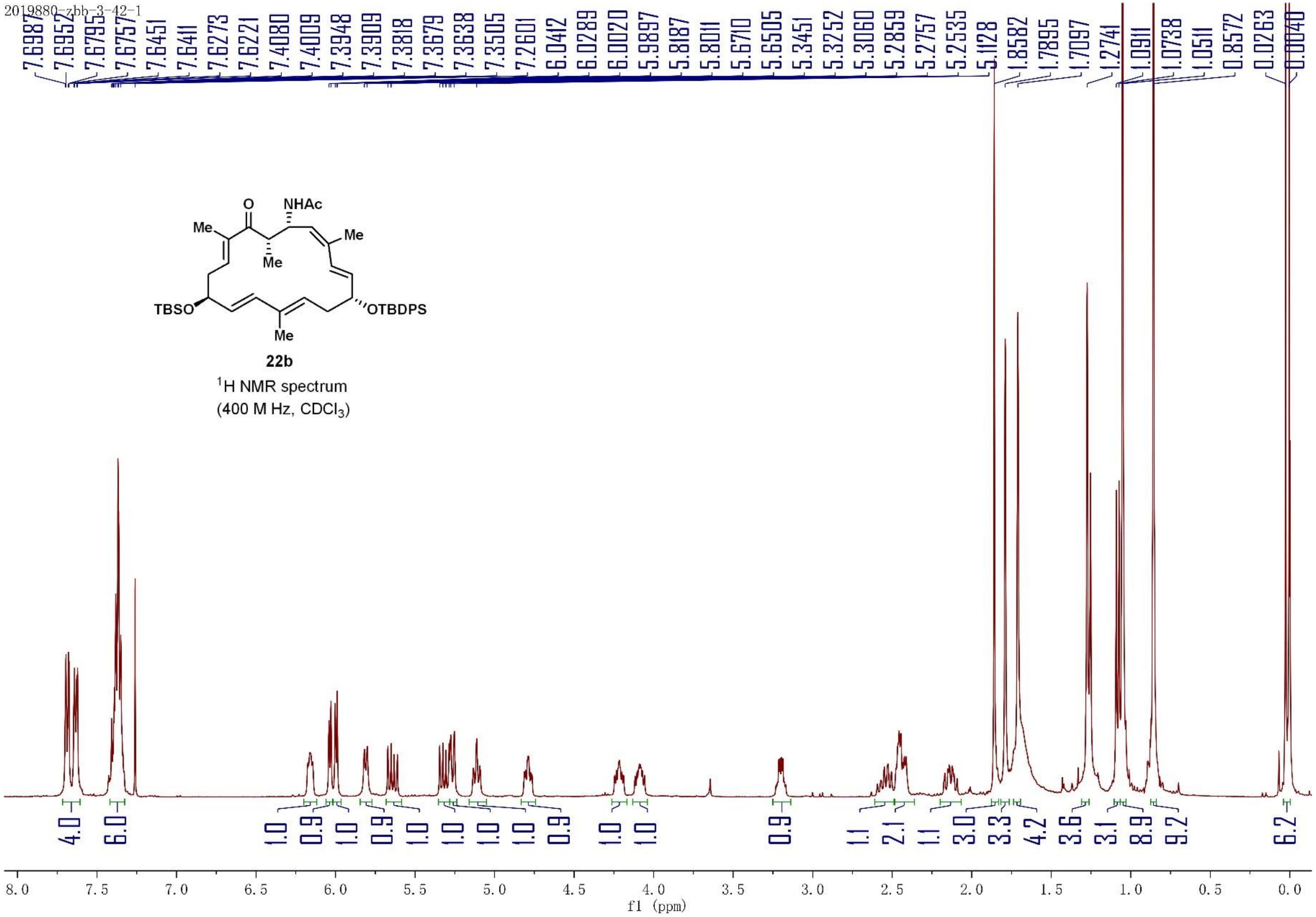
2019880-zbb-3-42-1
7.6987 7.6952 7.6795 7.6757 7.6451 7.6411 7.6273 7.6221 7.4080 7.4009 7.3948 7.3909 7.3818 7.3679 7.3638 7.3505 7.2601 6.0412 6.0289 6.0020 5.9897 5.8187 5.8011 5.6710 5.6505 5.3451 5.3252 5.3060 5.2859 5.2757 5.2535 5.1128 1.8582 1.7895 1.7097 1.2741 1.0911 1.0738 1.0511 0.8572 0.0263 0.0040
O
NHAc
Me
Me
Me
TBSO
OTBDPS
Me
22b
^{1}H NMR spectrum
(400 M Hz, $CDCl_3$)
4.0 6.0 1.0 0.9 1.0 0.9 1.0 1.0 1.0 1.0 0.9 1.0 1.0 0.9 1.1 2.1 1.1 3.0 3.3 4.2 3.6 3.1 8.9 9.2 6.2
8.0 7.5 7.0 6.5 6.0 5.5 5.0 4.5 4.0 3.5 3.0 2.5 2.0 1.5 1.0 0.5 0.0
f1 (ppm)

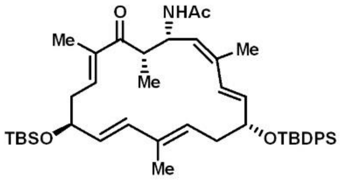

22b

^{13}C NMR spectrum

(100 M Hz, $CDCl_3$)

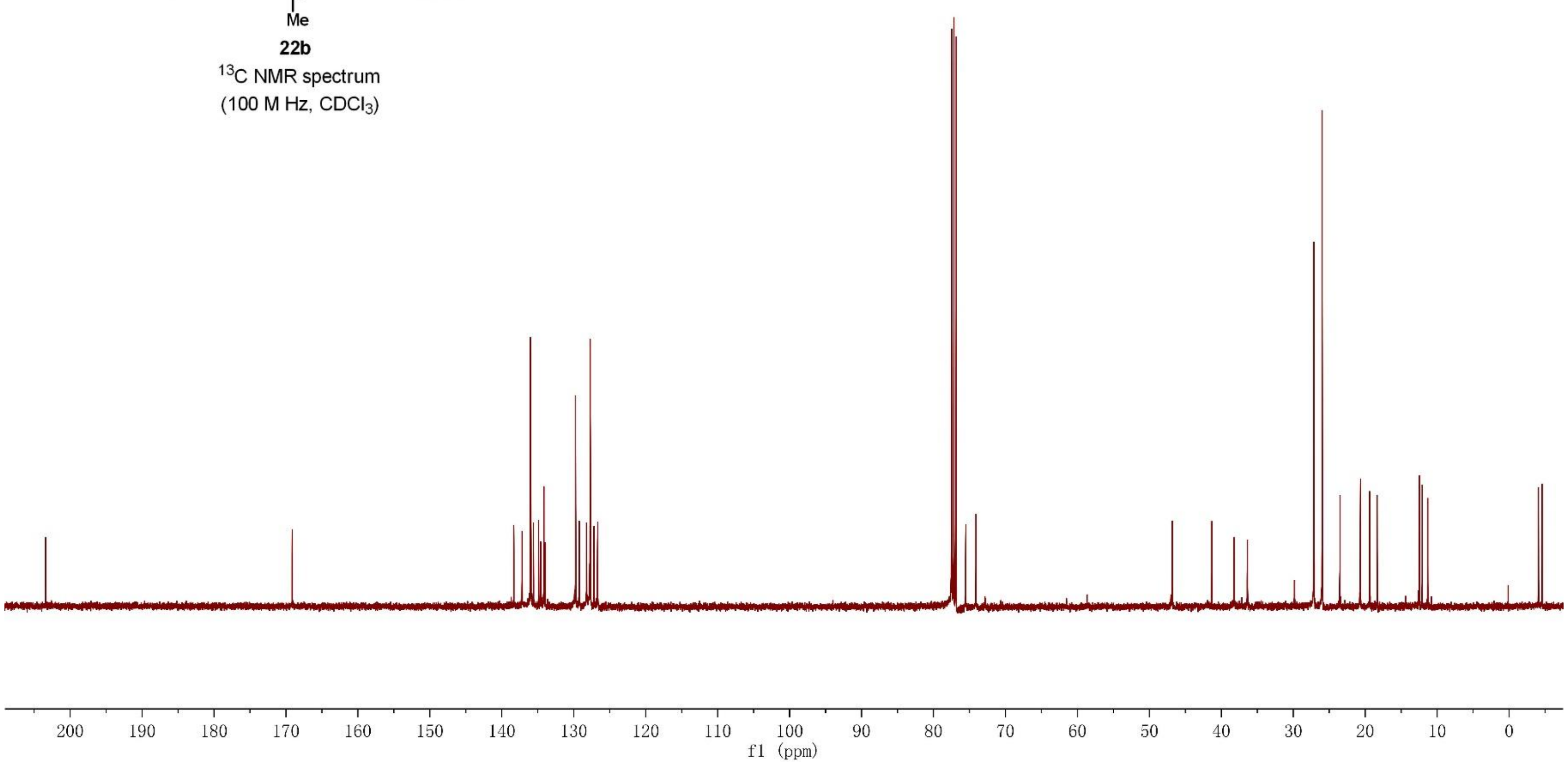

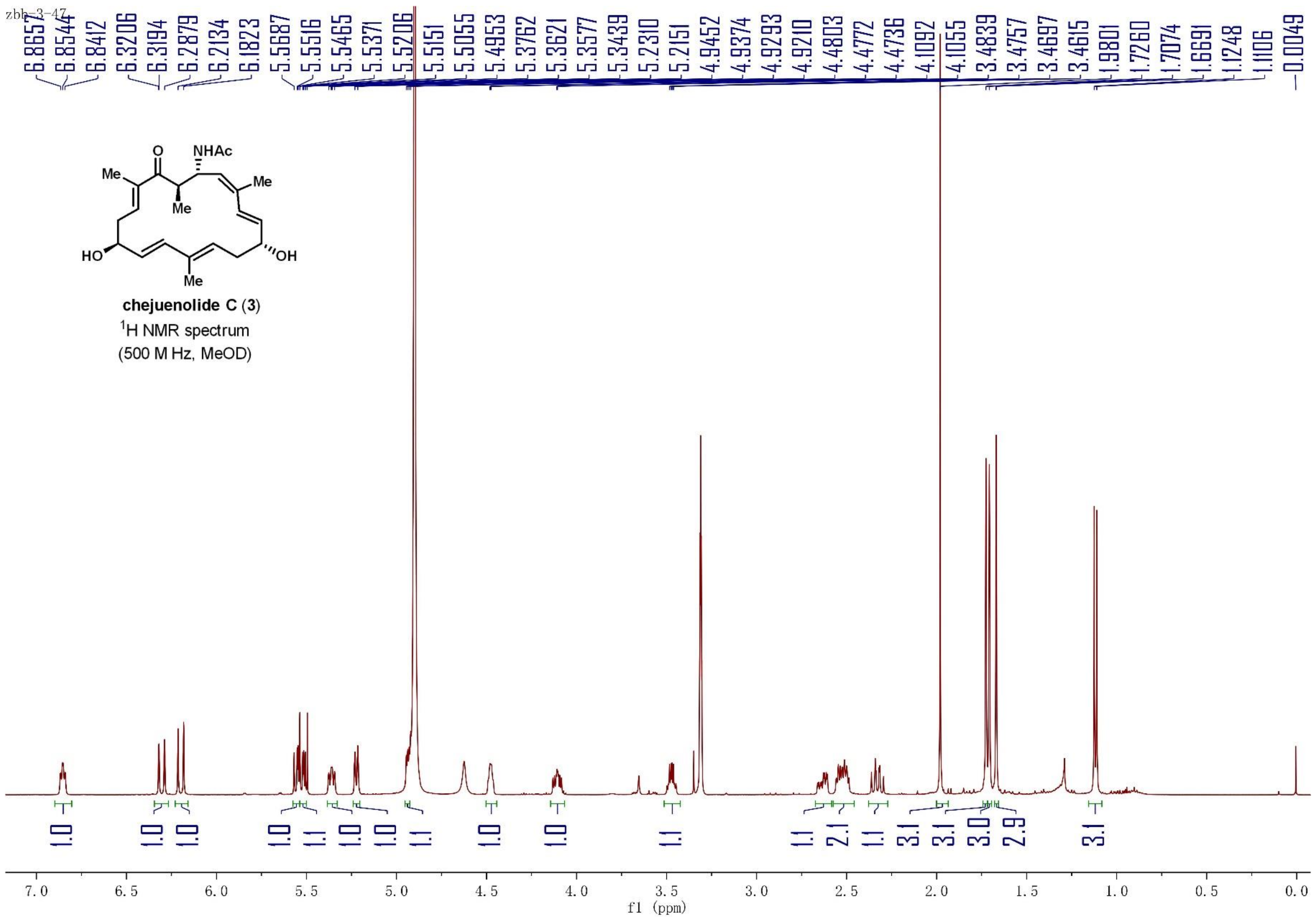
zbb-3-47
NHAc
Me
Me
Me
HO
OH
Me
chejuenolide C (3)
1H NMR spectrum
(500 M Hz, MeOD)
f1 (ppm)

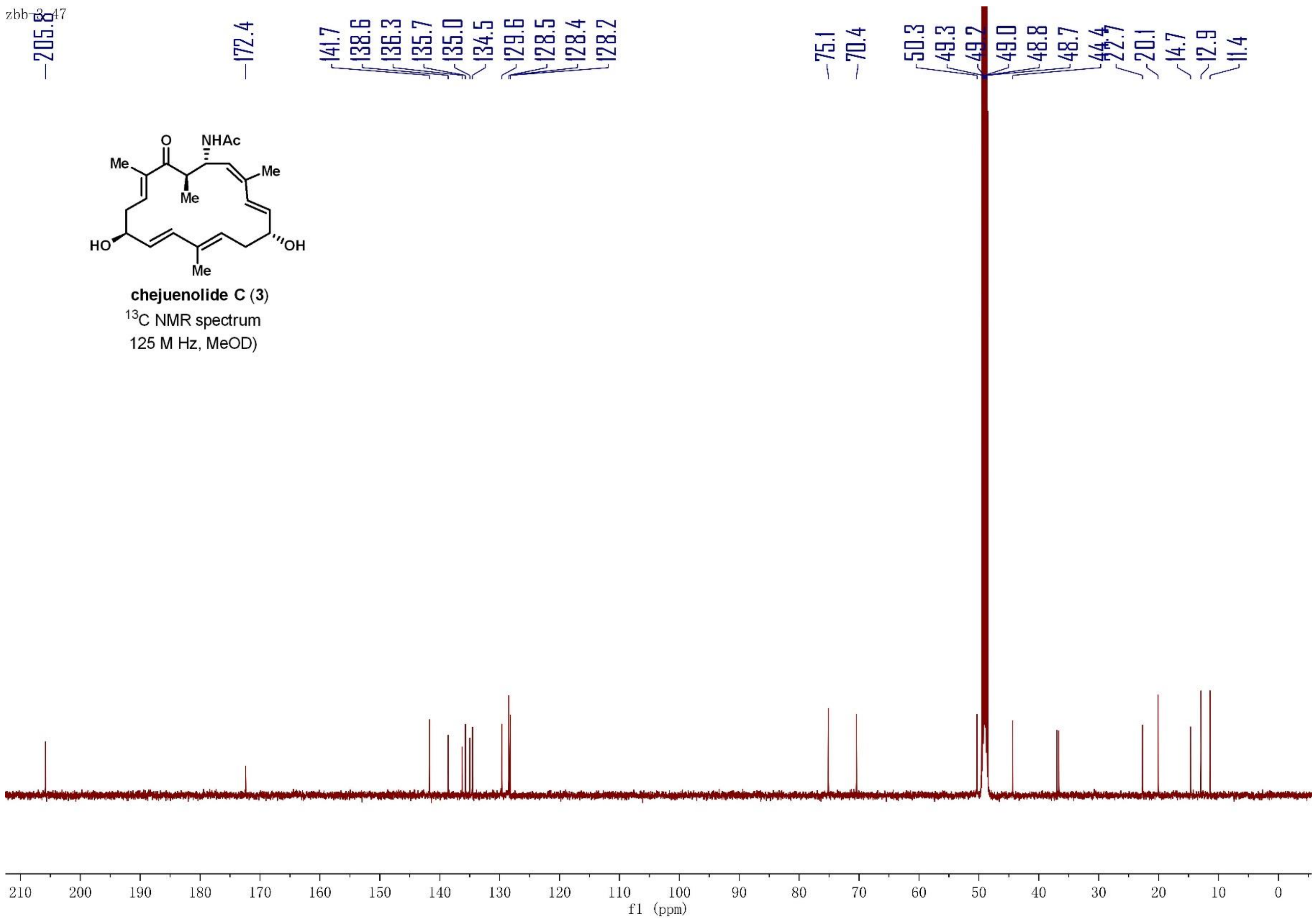

chejuenolide C (**3**)
^{13}C NMR spectrum
125 M Hz, MeOD)

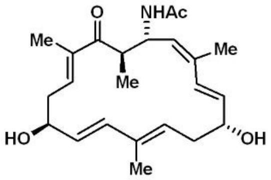

chejuenolide C (3)
H-H COSY spectrum
(500 M Hz, MeOD)

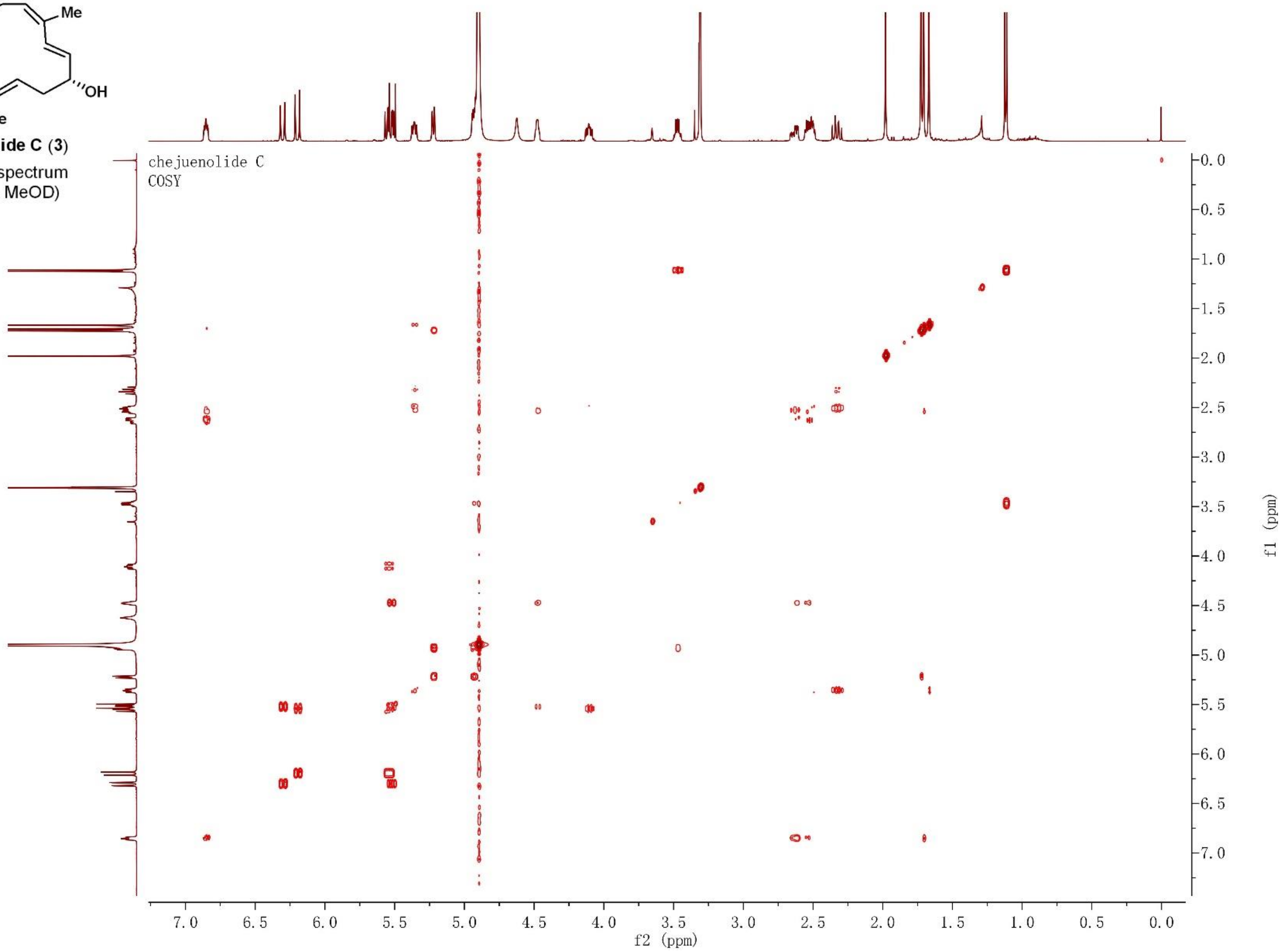

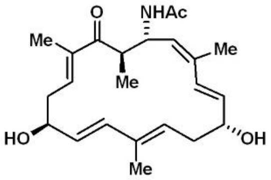

chejuenolide C (**3**)
NOESY spectrum
(500 M Hz, MeOD)

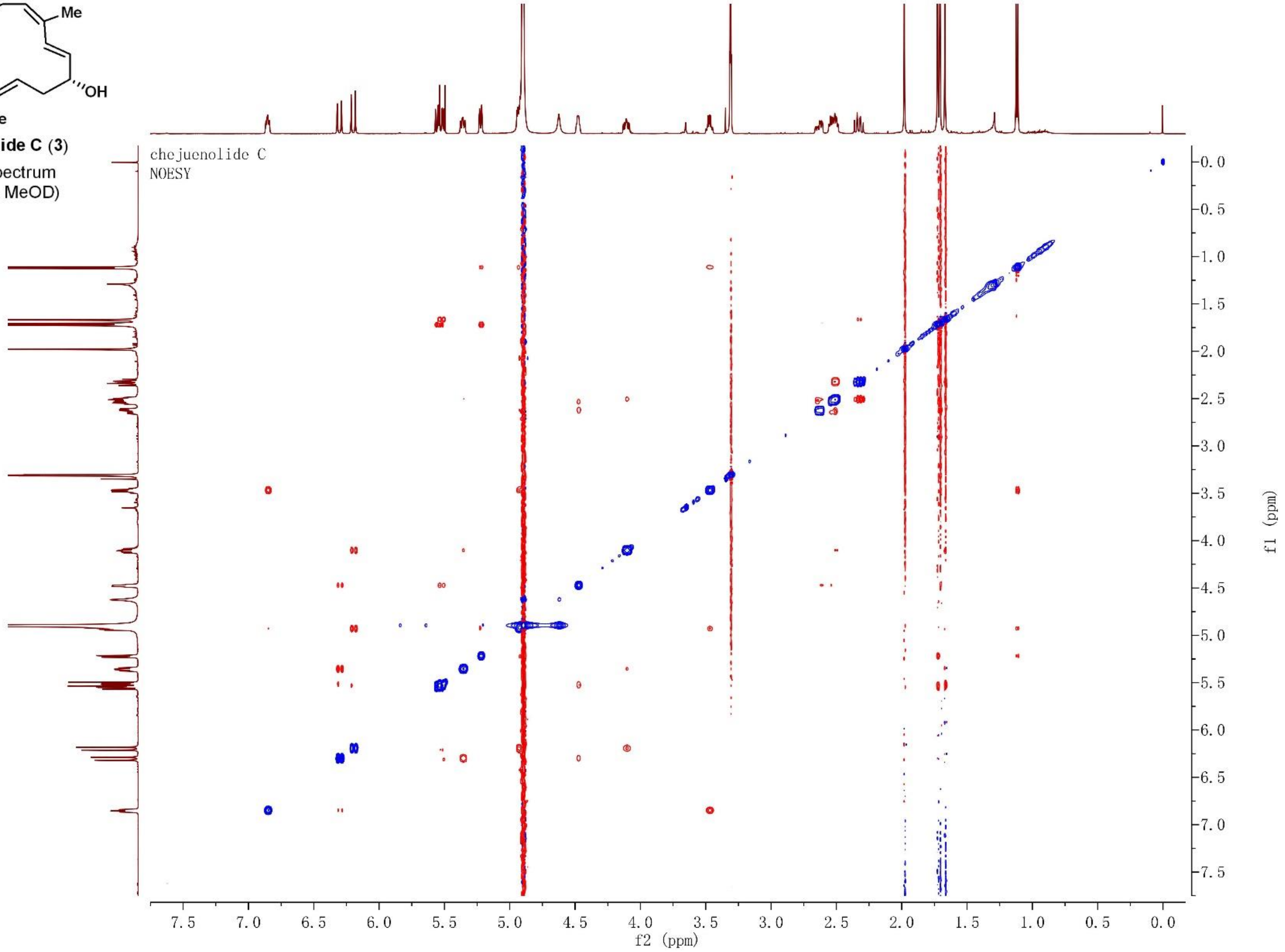

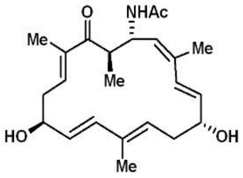

chejuenolide C (3)

C-H HSQC spectrum
(500 M Hz, MeOD)

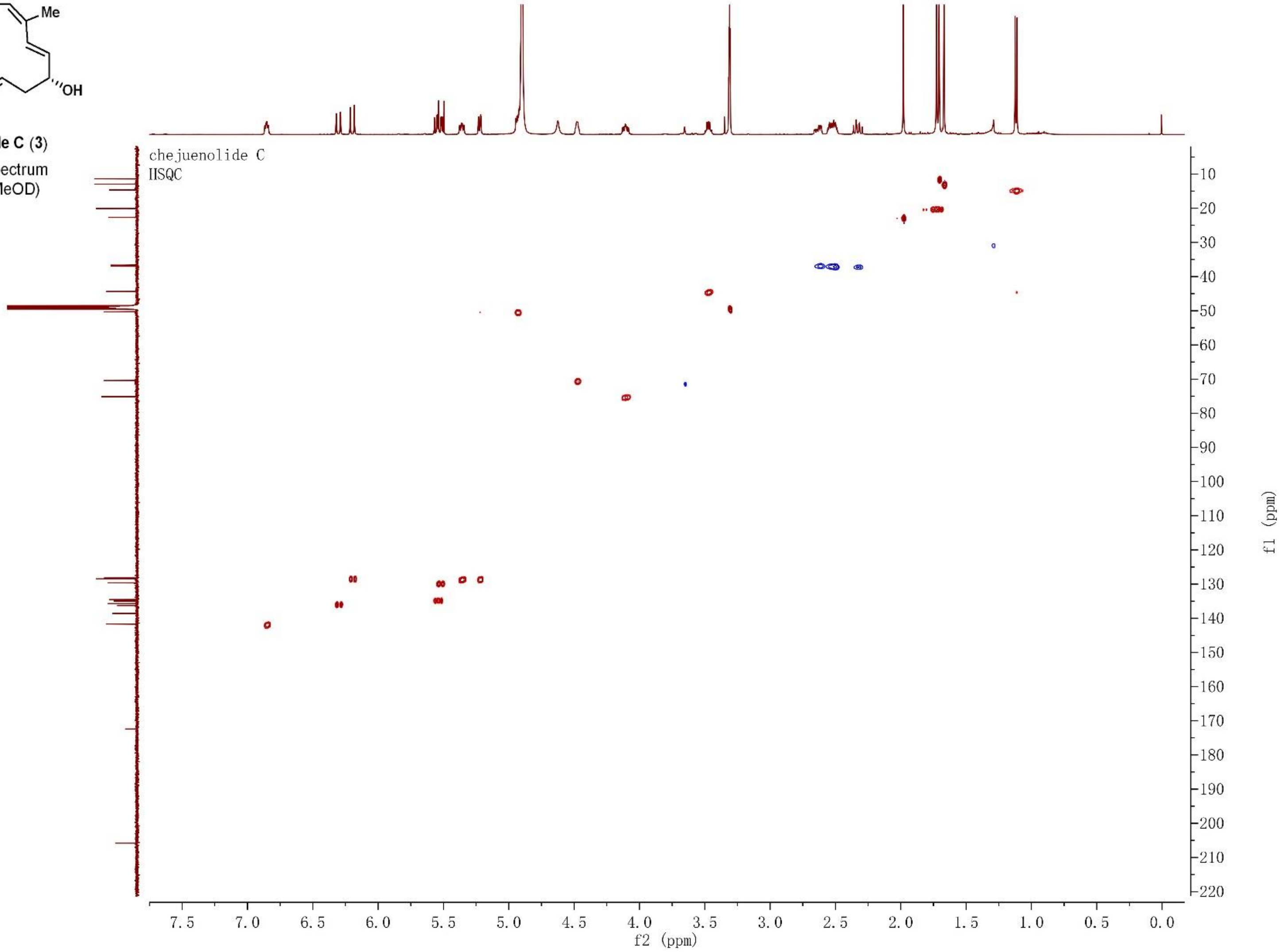

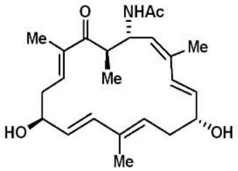

chejuenolide C (3)
C-H HMBC spectrum
(500 M Hz, MeOD)

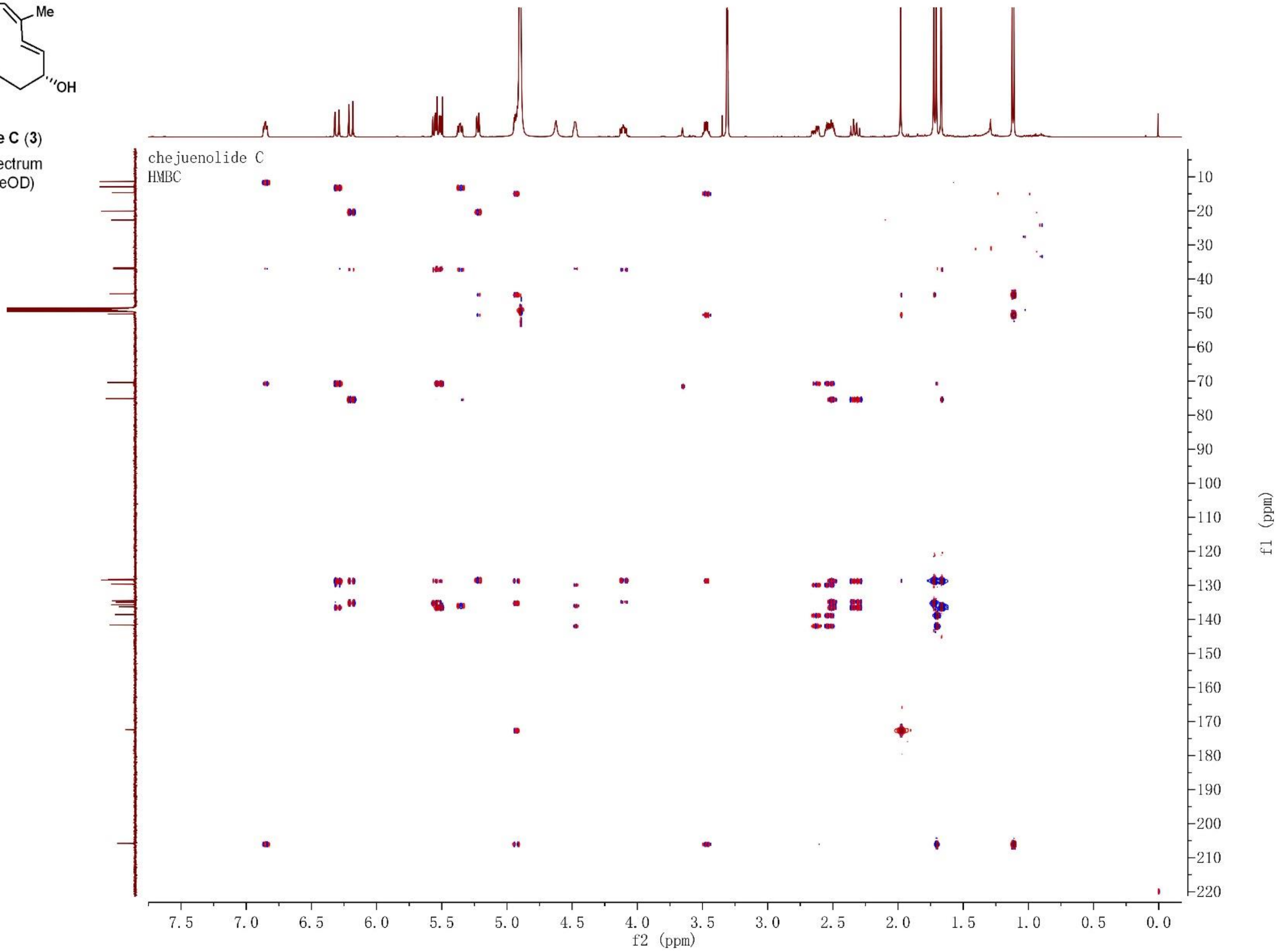

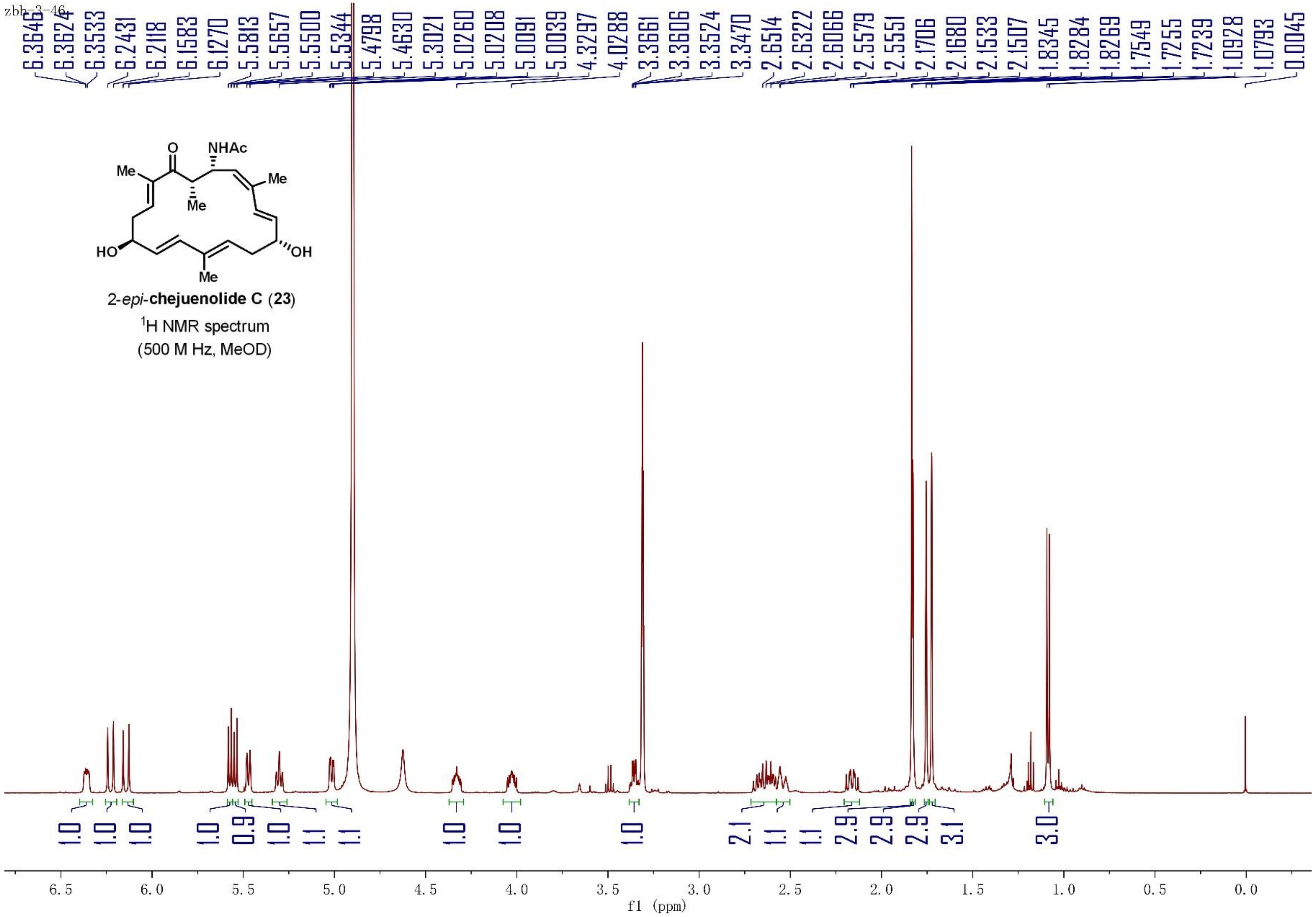
zbh-3-46
6.3646
6.3624
6.3533
6.2431
6.2118
6.1583
6.1270
5.5813
5.5657
5.5500
5.5344
5.4798
5.4630
5.3021
5.0260
5.0208
5.0091
5.0039
4.3297
4.0288
3.3661
3.3606
3.3524
3.3470
2.6514
2.6322
2.6066
2.5579
2.5551
2.1706
2.1680
2.1533
2.1507
1.8345
1.8284
1.8269
1.7549
1.7255
1.7239
1.0928
1.0793
0.0045
O
NHAc
Me
Me
Me
HO
OH
Me
2-*epi*-**chejuenolide C** (**23**)
¹H NMR spectrum
(500 M Hz, MeOD)
1.0
1.0
1.0
1.0
0.9
1.0
1.1
1.1
1.0
1.0
1.0
2.1
1.1
1.1
2.9
2.9
2.9
3.1
3.0
6.5
6.0
5.5
5.0
4.5
4.0
3.5
3.0
2.5
2.0
1.5
1.0
0.5
0.0
f1 (ppm)

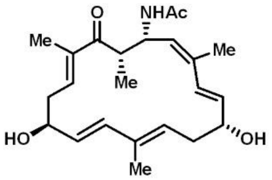

2-*epi*-**chejuenolide C** (**23**)

^{13}C NMR spectrum

(125 M Hz, MeOD)

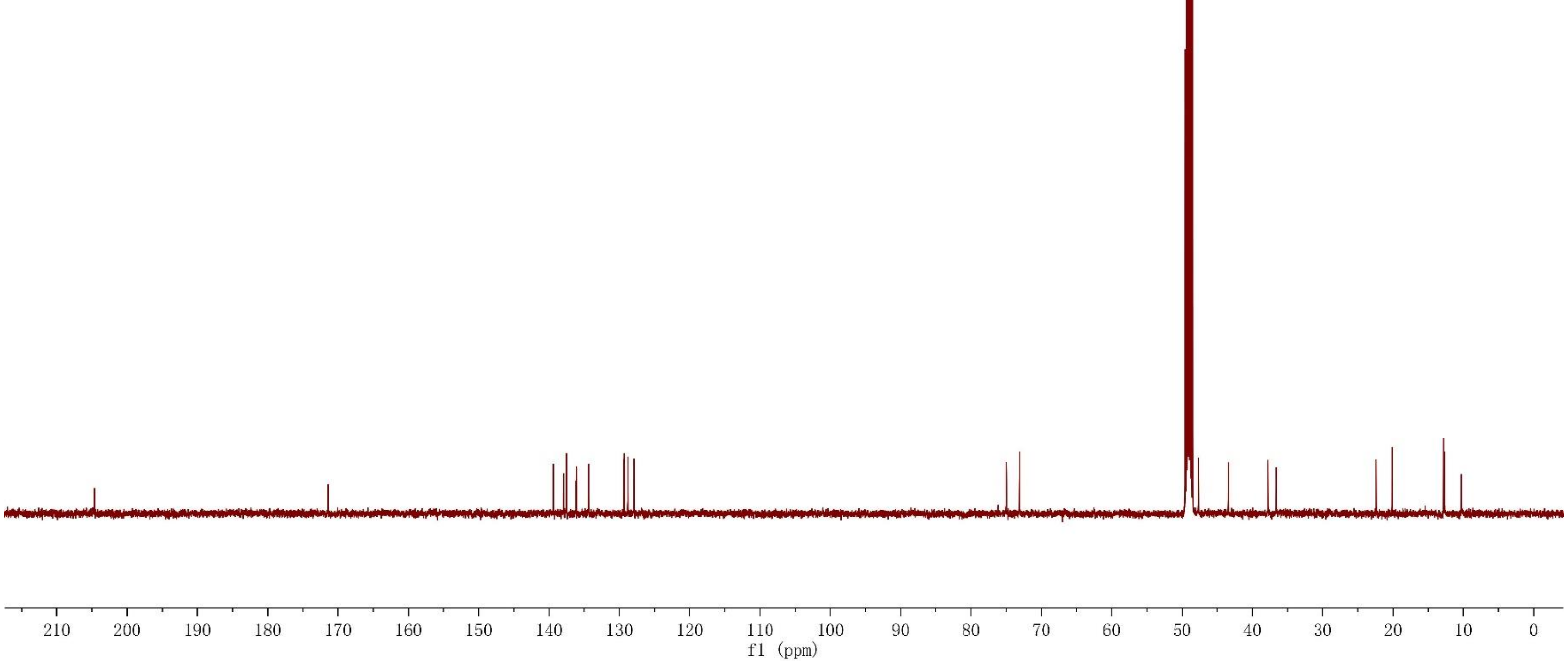

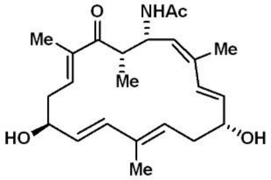

2-*epi*-**chejuenolide C** (**23**)

H-H COSY spectrum
(500 M Hz, MeOD)

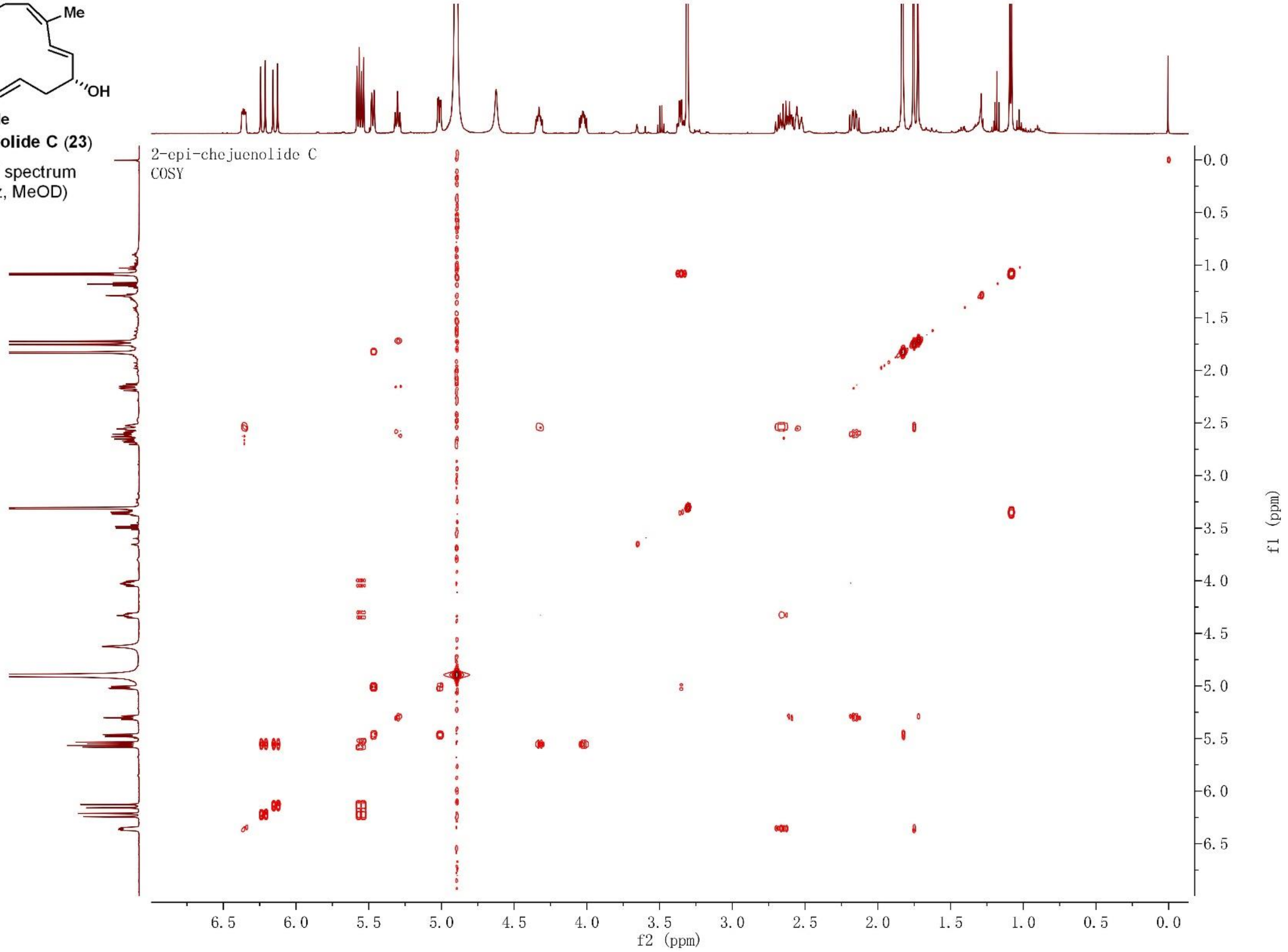

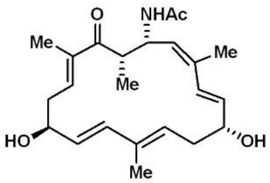

2-*epi*-**chejuenolide C** (**23**)

NOESY spectrum
(500 M Hz, MeOD)

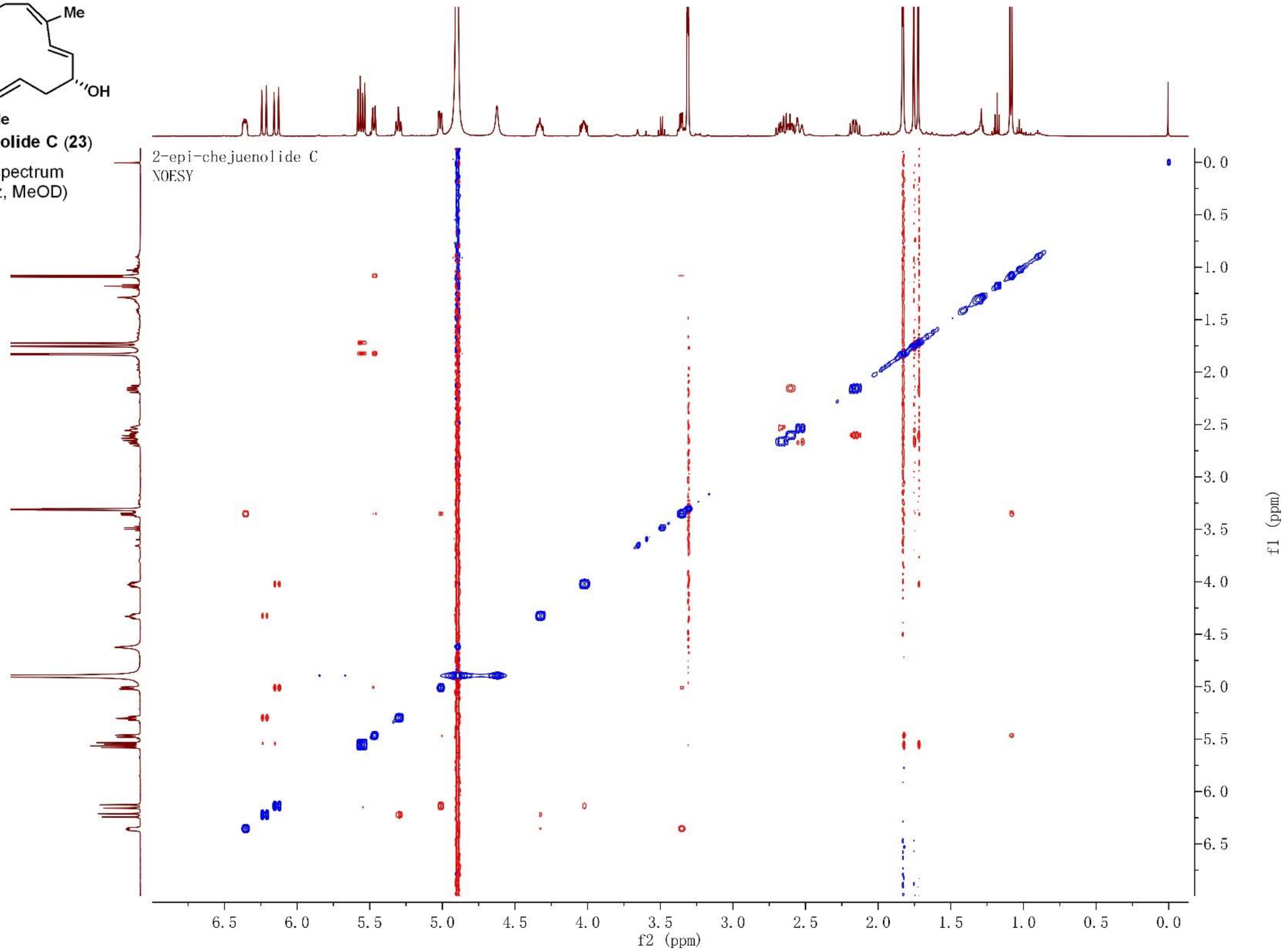

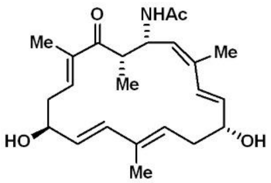

2-*epi*-**chejuenolide C** (**23**)

C-H HSQC spectrum
(500 M Hz, MeOD)

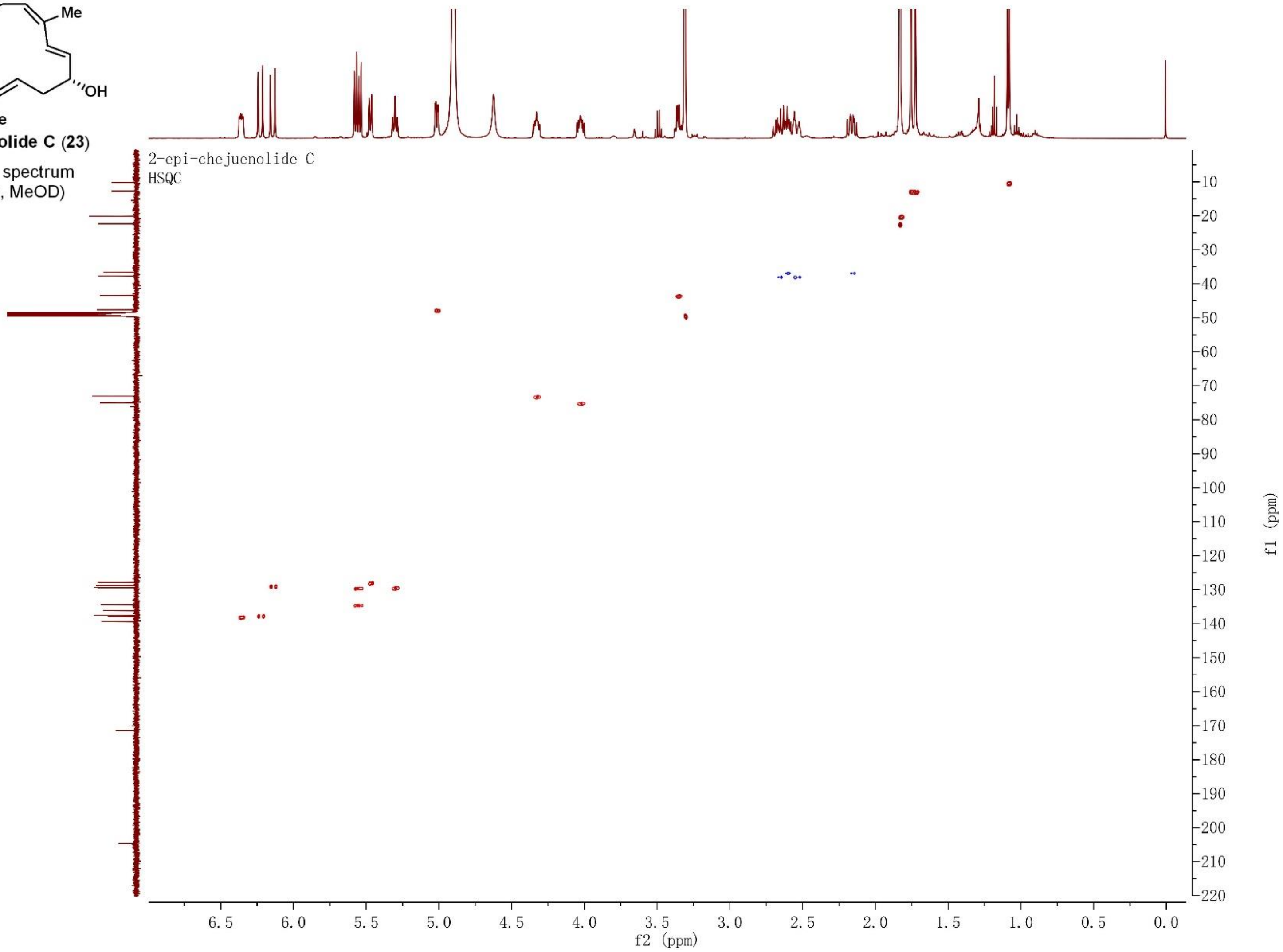

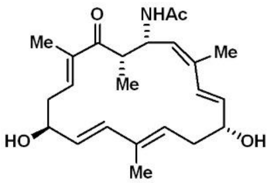

2-*epi*-**chejuenolide C** (**23**)

C-H HMBC spectrum
(500 M Hz, MeOD)

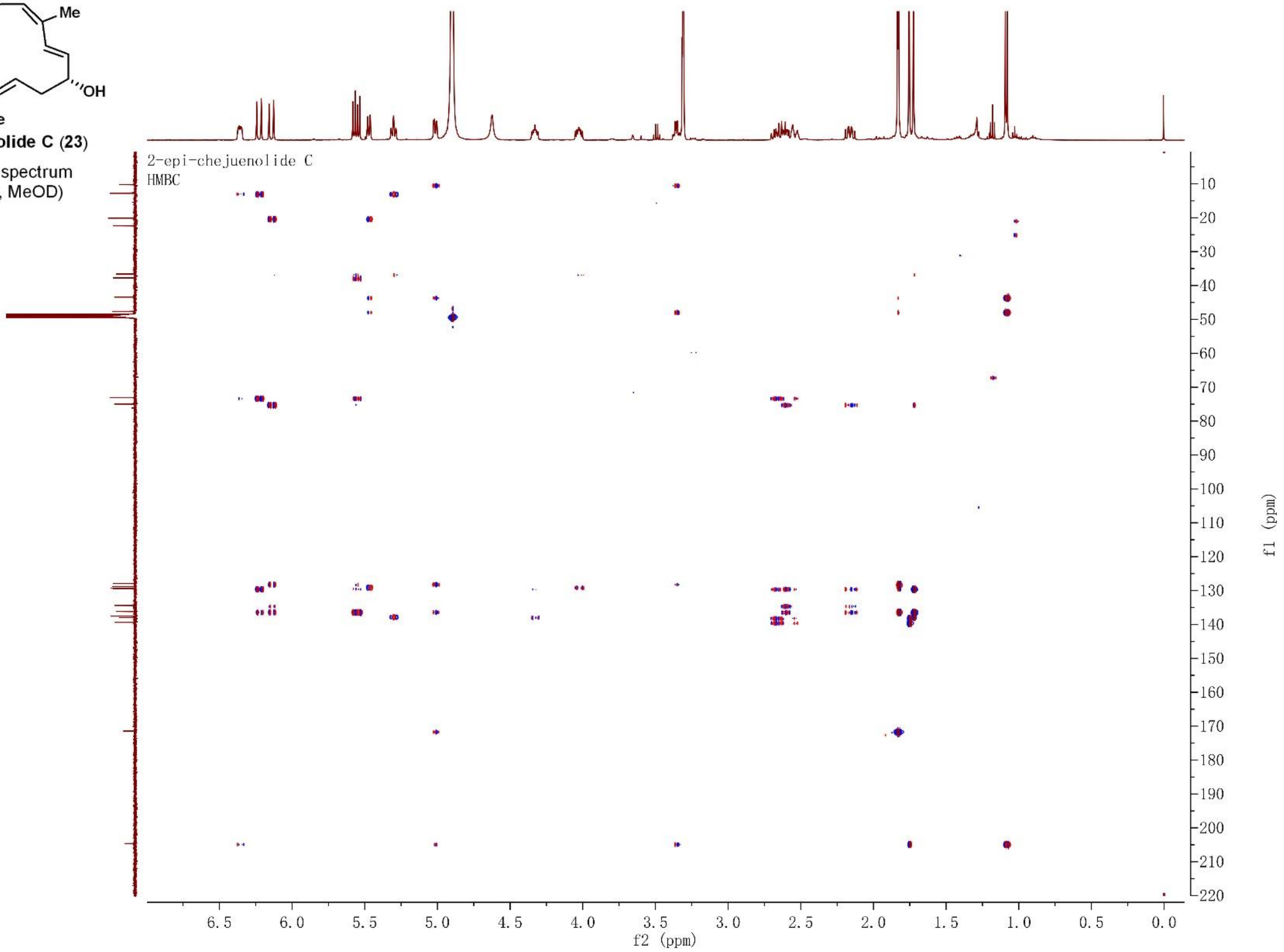

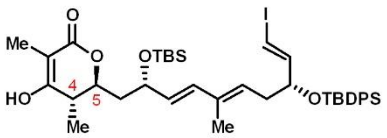

4*S*,5*S*-lactone **24**
(*undesired minor product*)
^{1}H NMR spectrum
(400 MHz, DMSO-d_6)

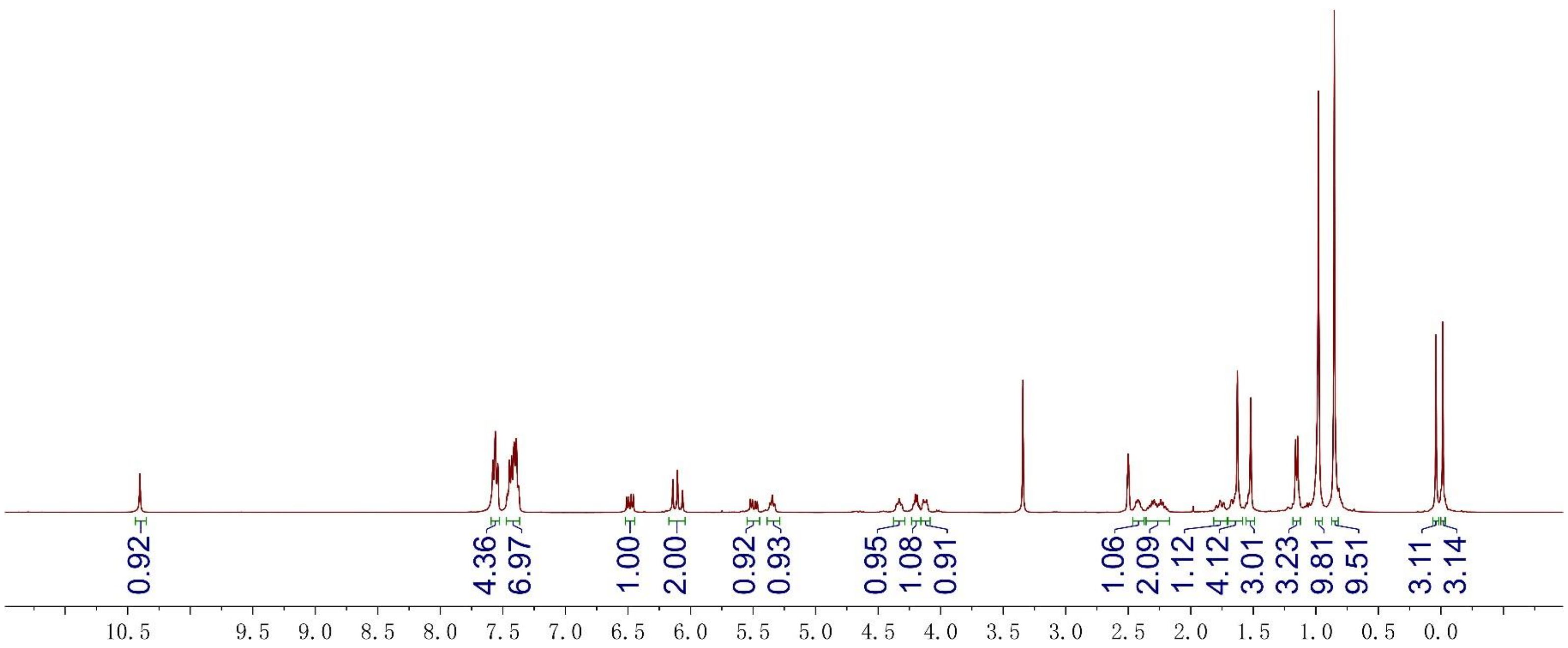

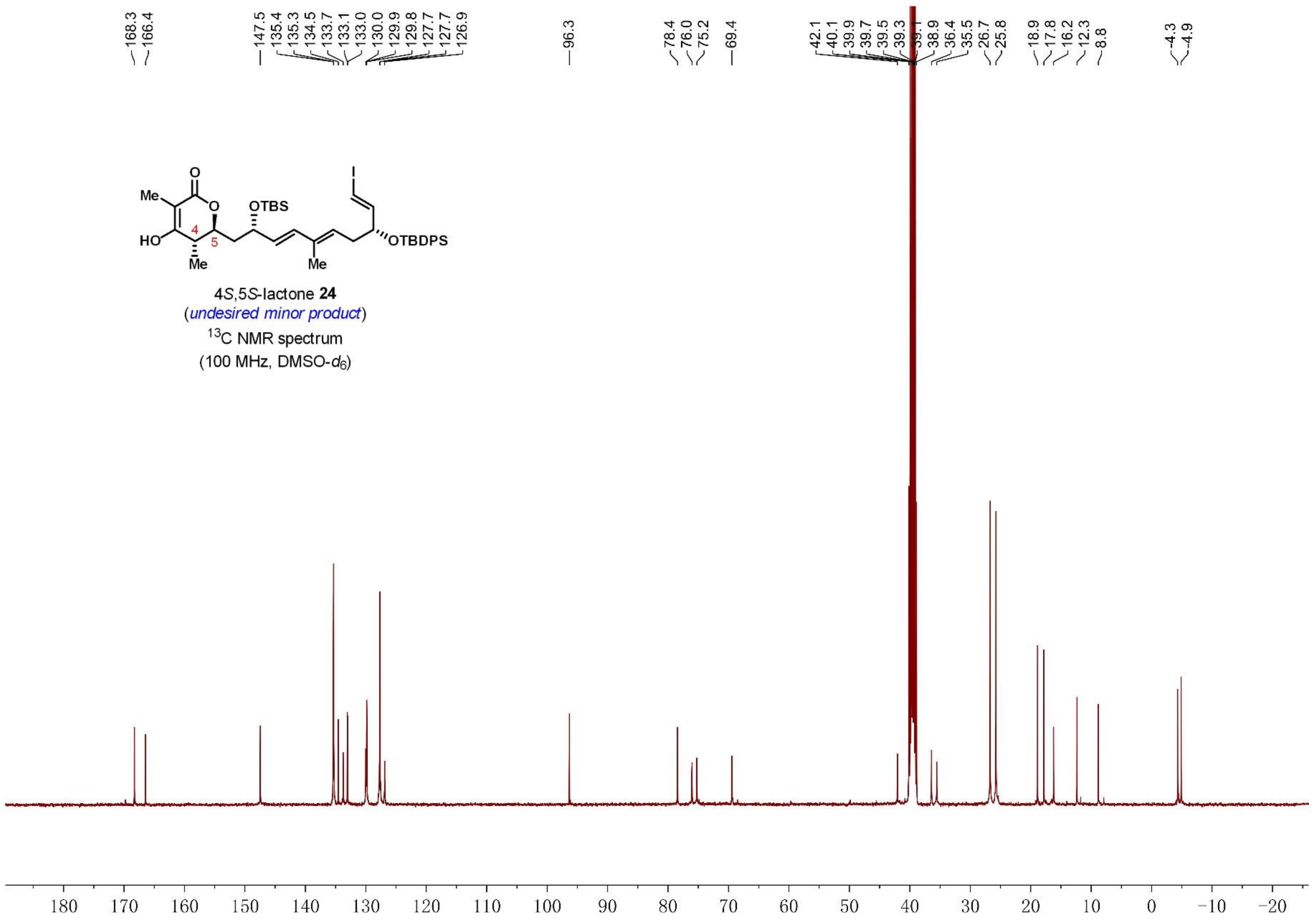
168.3
166.4
147.5
135.4
135.3
134.5
133.7
133.1
133.0
130.0
129.9
129.8
127.7
127.7
126.9
96.3
78.4
76.0
75.2
69.4
42.1
40.1
39.9
39.7
39.5
39.3
39.1
38.9
36.4
35.5
26.7
25.8
18.9
17.8
16.2
12.3
8.8
-4.3
-4.9
Me
O
OTBS
I
HO
4
5
Me
Me
OTBDPS
4S,5S-lactone 24
(undesired minor product)
13C NMR spectrum
(100 MHz, DMSO-d6)
180
170
160
150
140
130
120
110
100
90
80
70
60
50
40
30
20
10
0
−10
−20

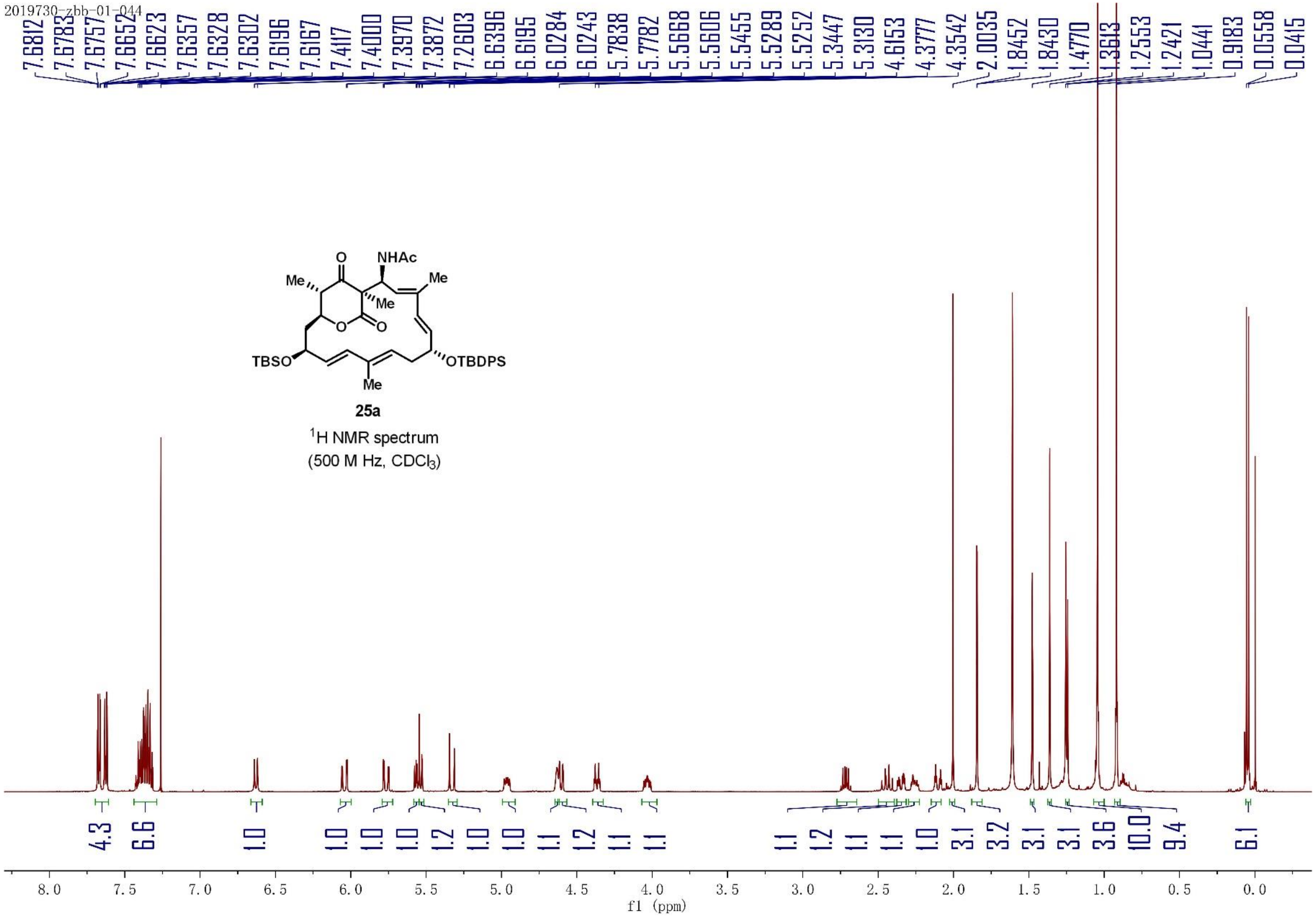

25a

^{1}H NMR spectrum

(500 M Hz, $CDCl_3$)

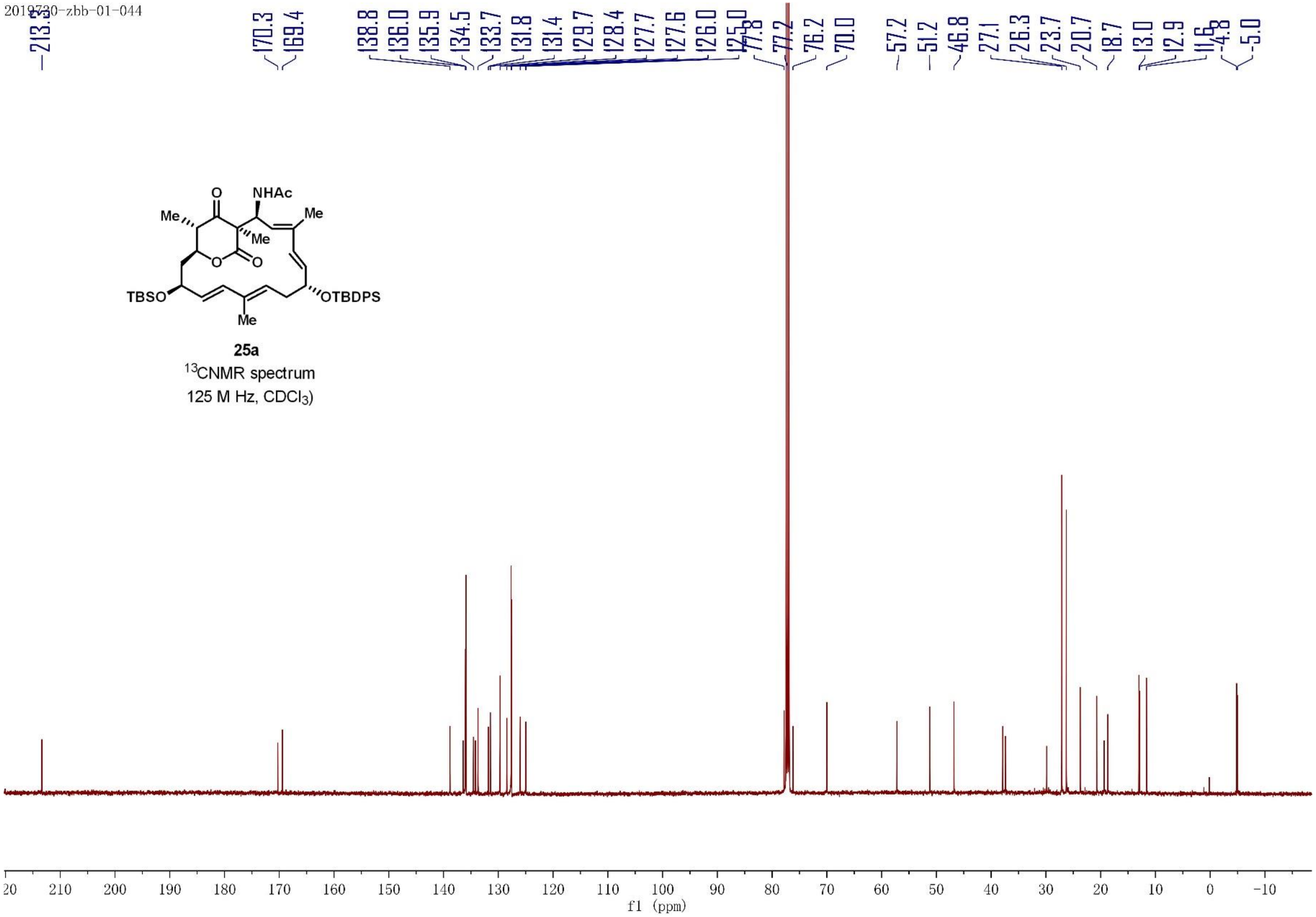
2019730-zbb-01-044
213.3
170.3
169.4
138.8
136.0
135.9
134.5
133.7
131.8
131.4
129.7
128.4
127.7
127.6
126.0
125.0
77.5
77.2
76.2
70.0
57.2
51.2
46.8
27.1
26.3
23.7
20.7
18.7
13.0
12.9
11.6
-4.8
-5.0
O
NHAc
Me
Me
Me
O
O
TBSO
OTBDPS
Me
25a
13CNMR spectrum
125 M Hz, CDCl3)
20 210 200 190 180 170 160 150 140 130 120 110 100 90 80 70 60 50 40 30 20 10 0 -10
f1 (ppm)

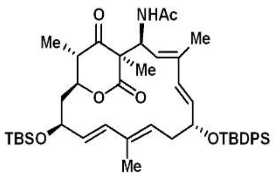

25a

H-H COSY spectrum
(500 M Hz, $CDCl_3$)

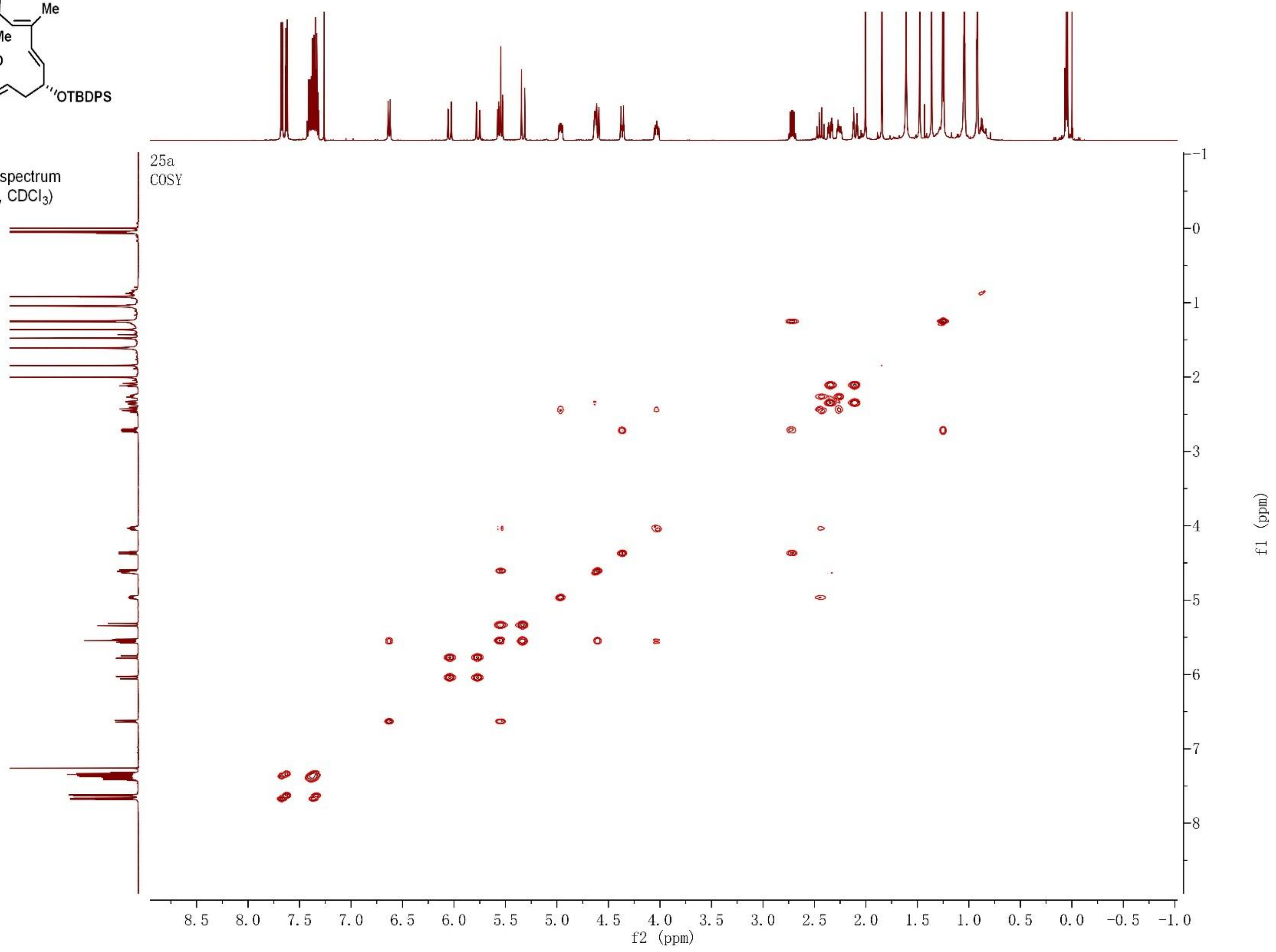

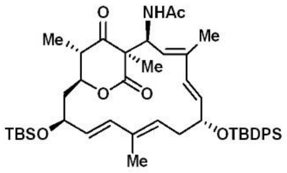

25a

NOESY spectrum
(500 M Hz, $CDCl_3$)

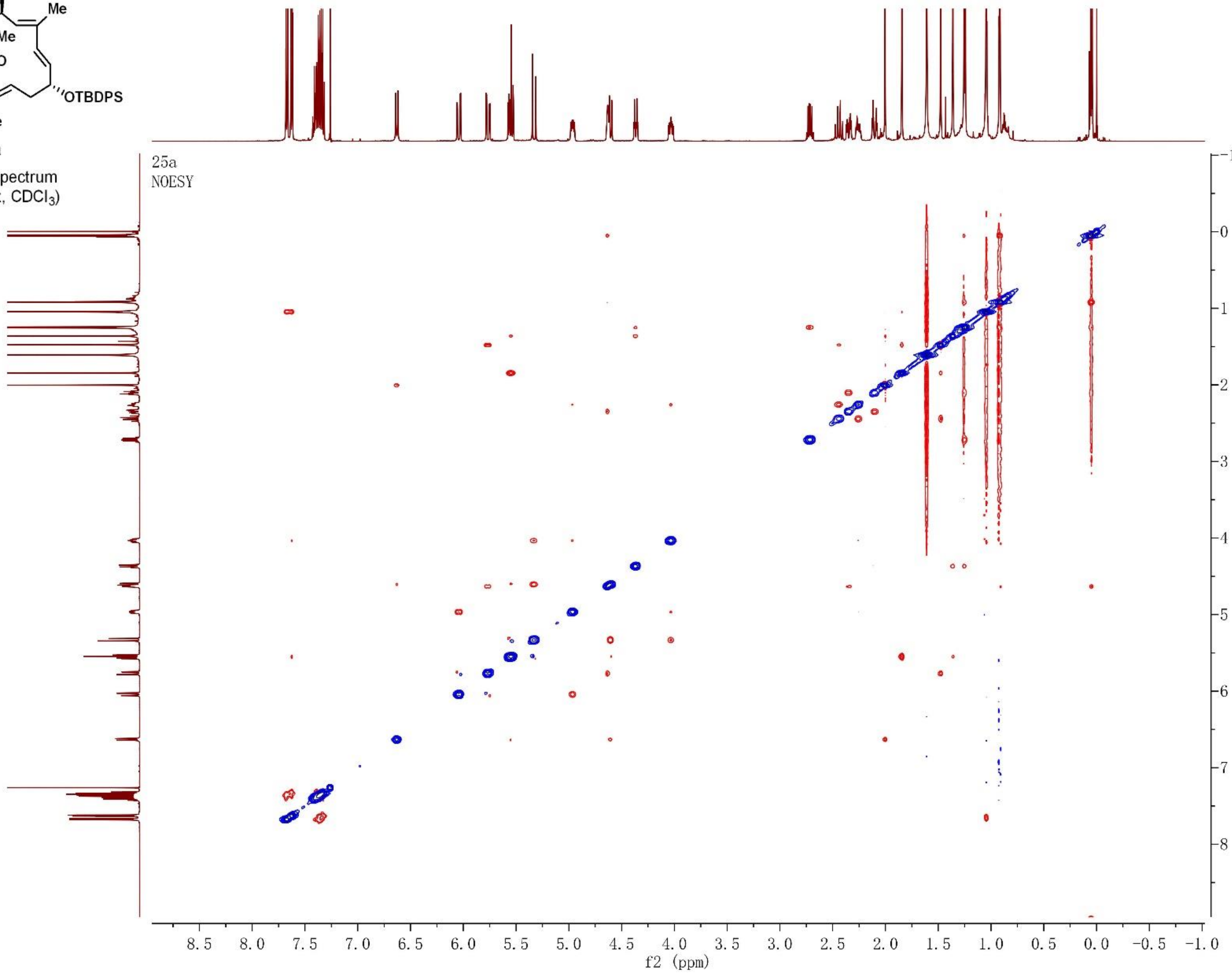

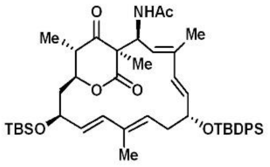

25a

C-H HSQC spectrum
(500 M Hz, $CDCl_3$)

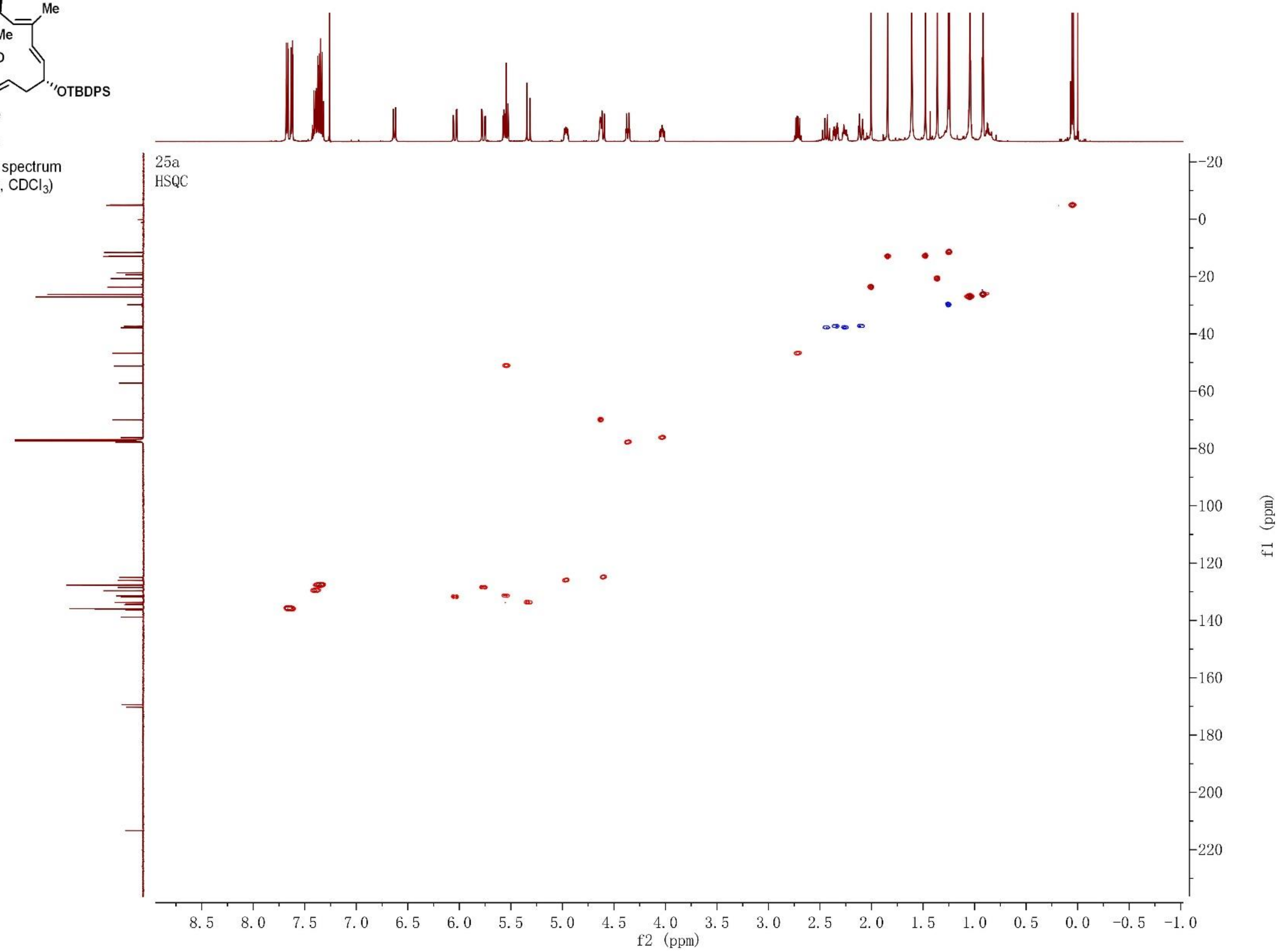

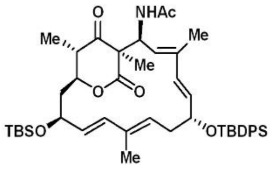

25a

C-H HMBC spectrum
(500 M Hz, $CDCl_3$)

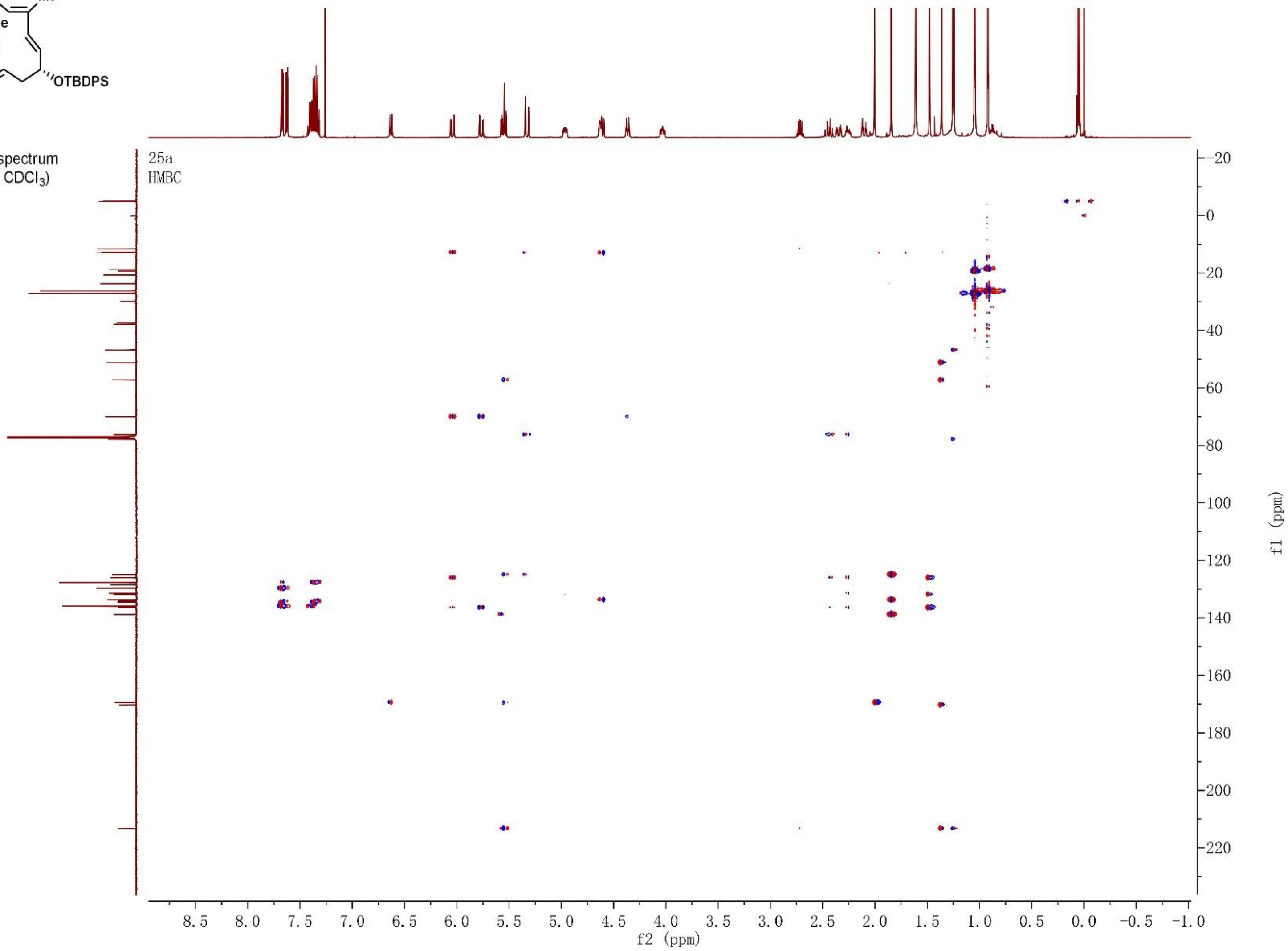

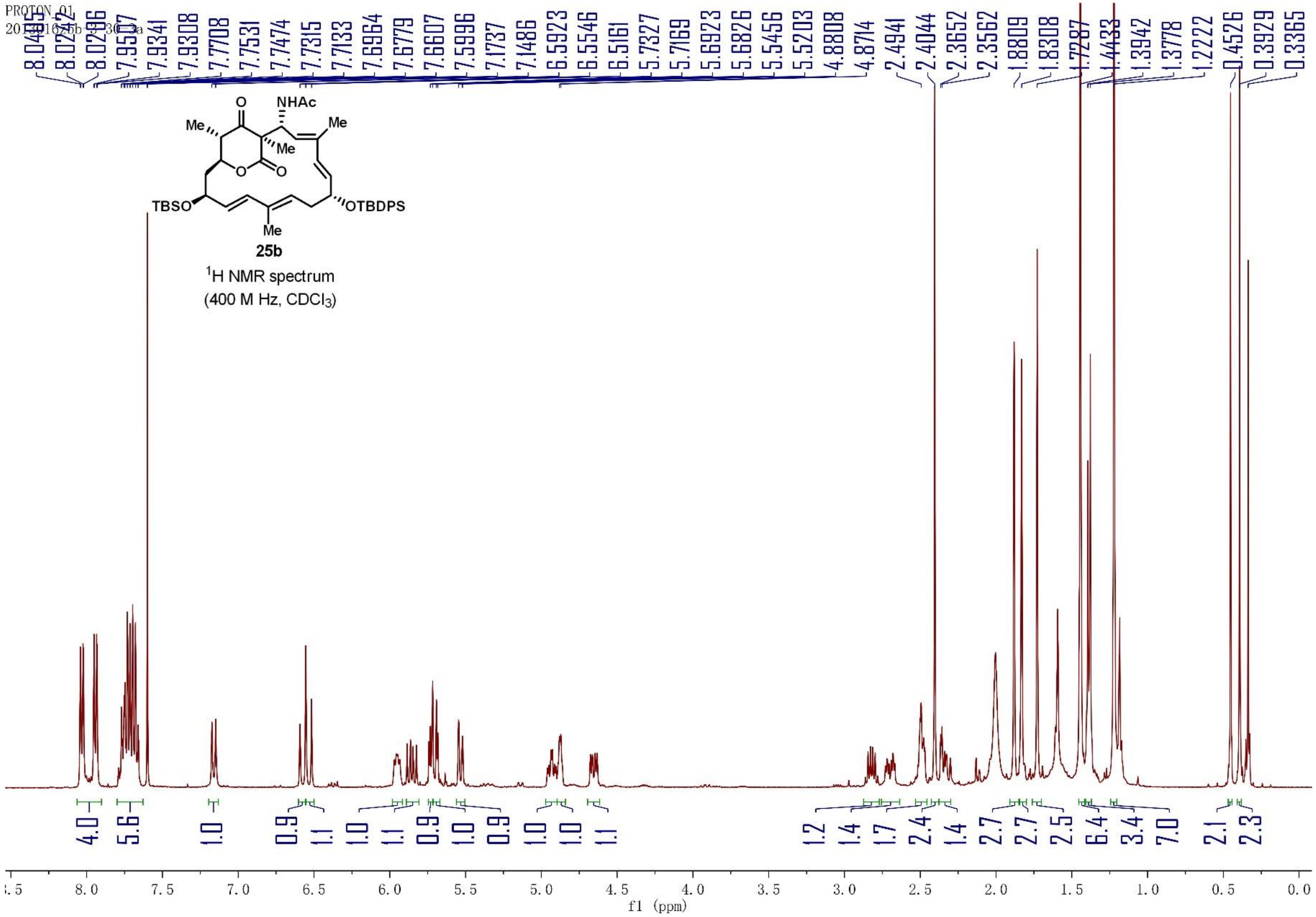

PROTON_01
25b
1H NMR spectrum
(400 M Hz, CDCl3)
f1 (ppm)

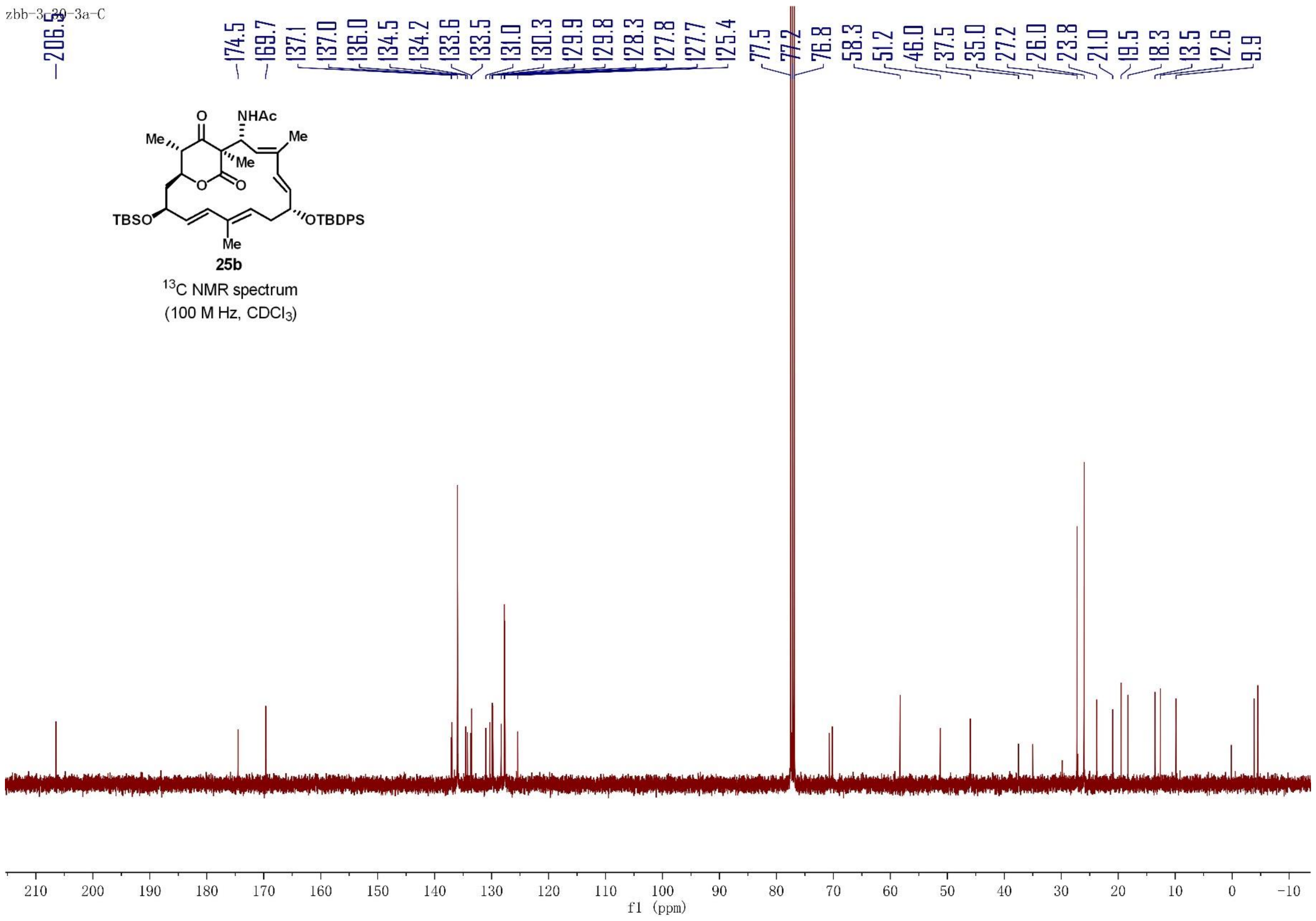

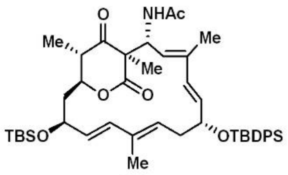

25b

H-H COSY spectrum
(400 M Hz, $CDCl_3$)

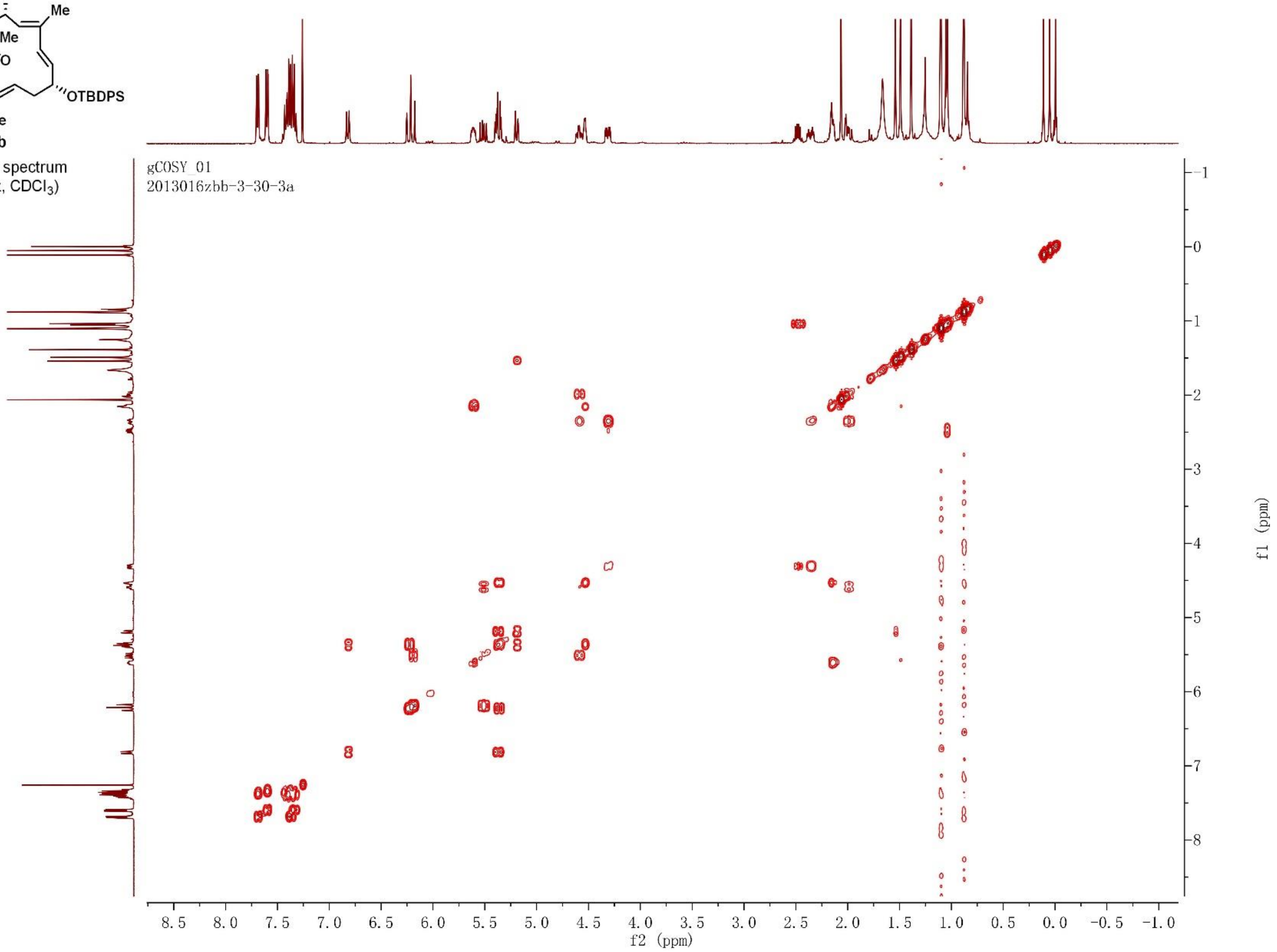

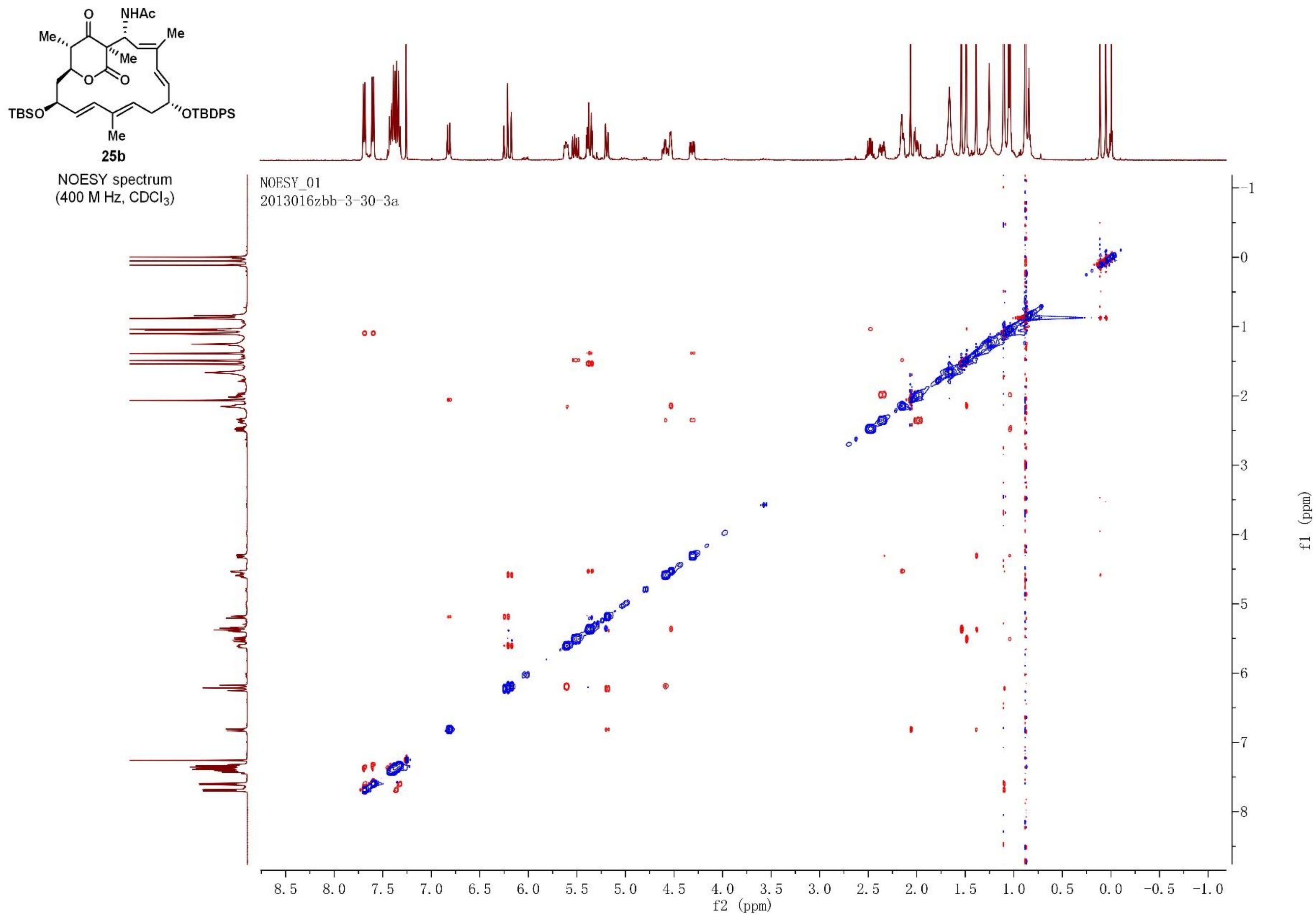
NHAc
Me
Me
TBSO
OTBDPS
25b
NOESY spectrum
(400 M Hz, CDCl3)
NOESY_01
2013016zbb-3-30-3a
f2 (ppm)
f1 (ppm)

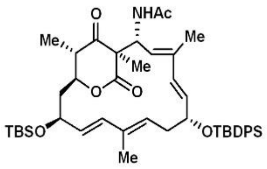

25b

C-H HSQC spectrum
(400 M Hz, $CDCl_3$)

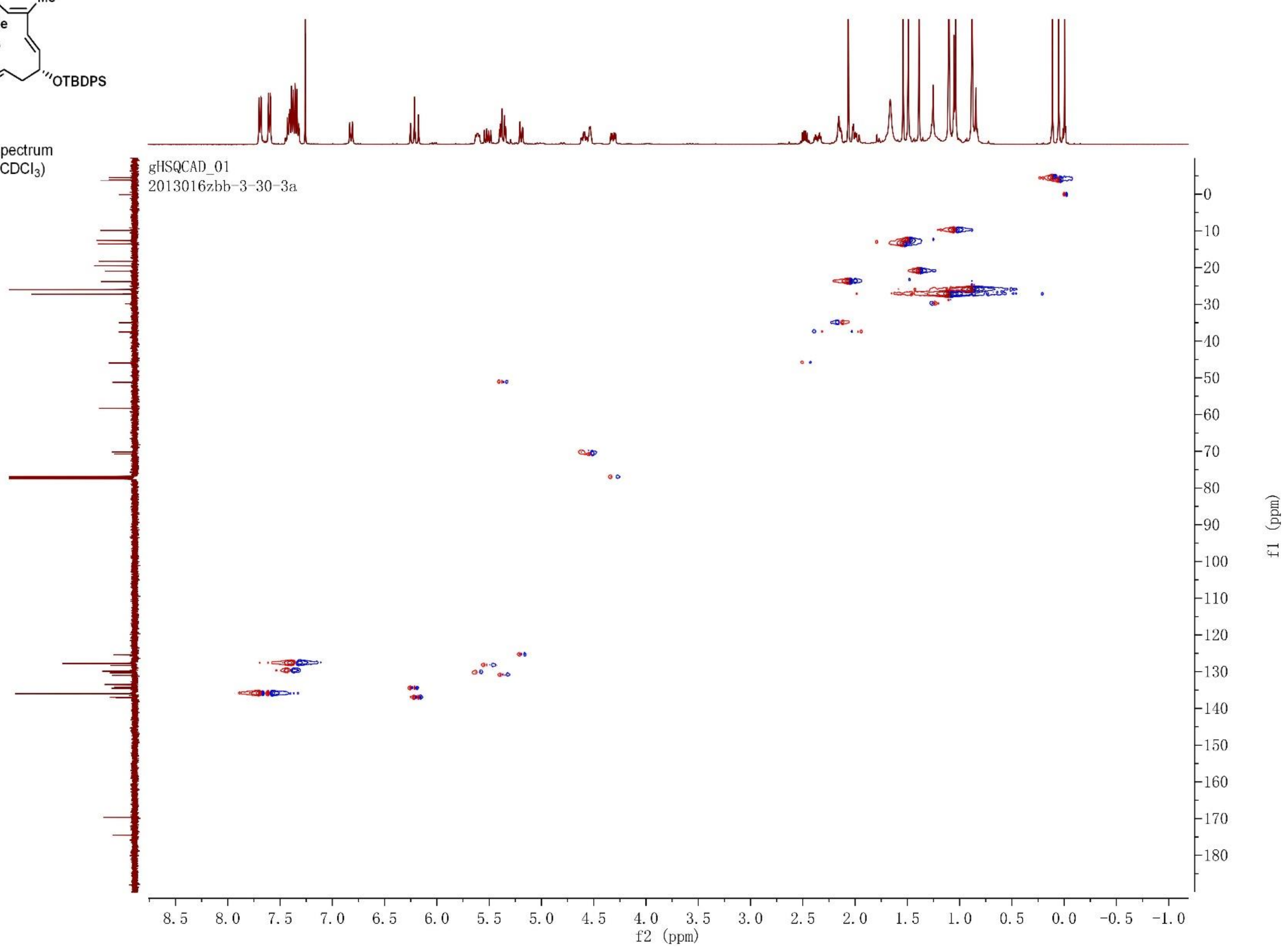

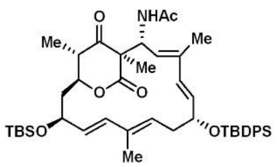

25b

C-H HMBC spectrum
(400 M Hz, $CDCl_3$)

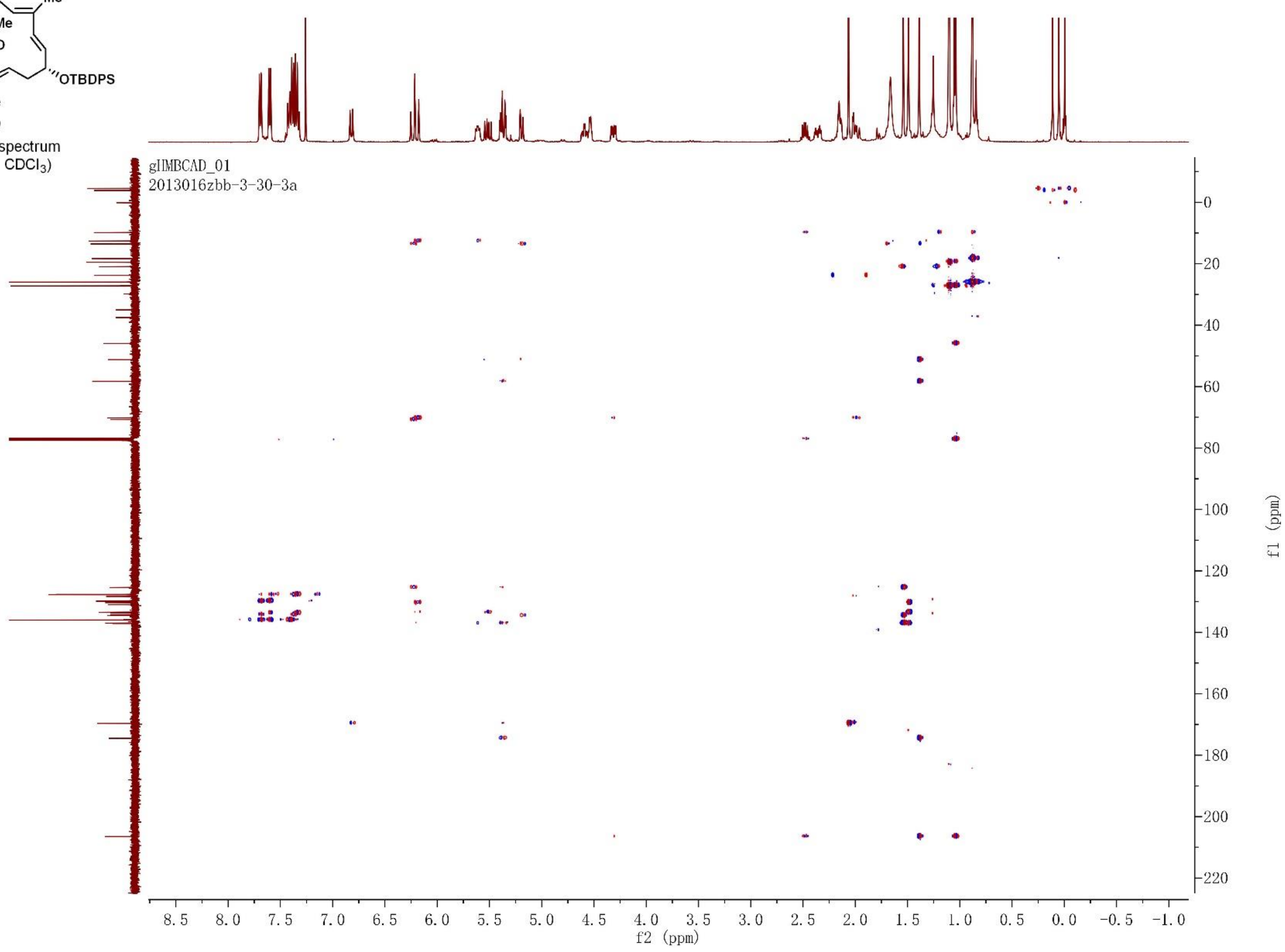

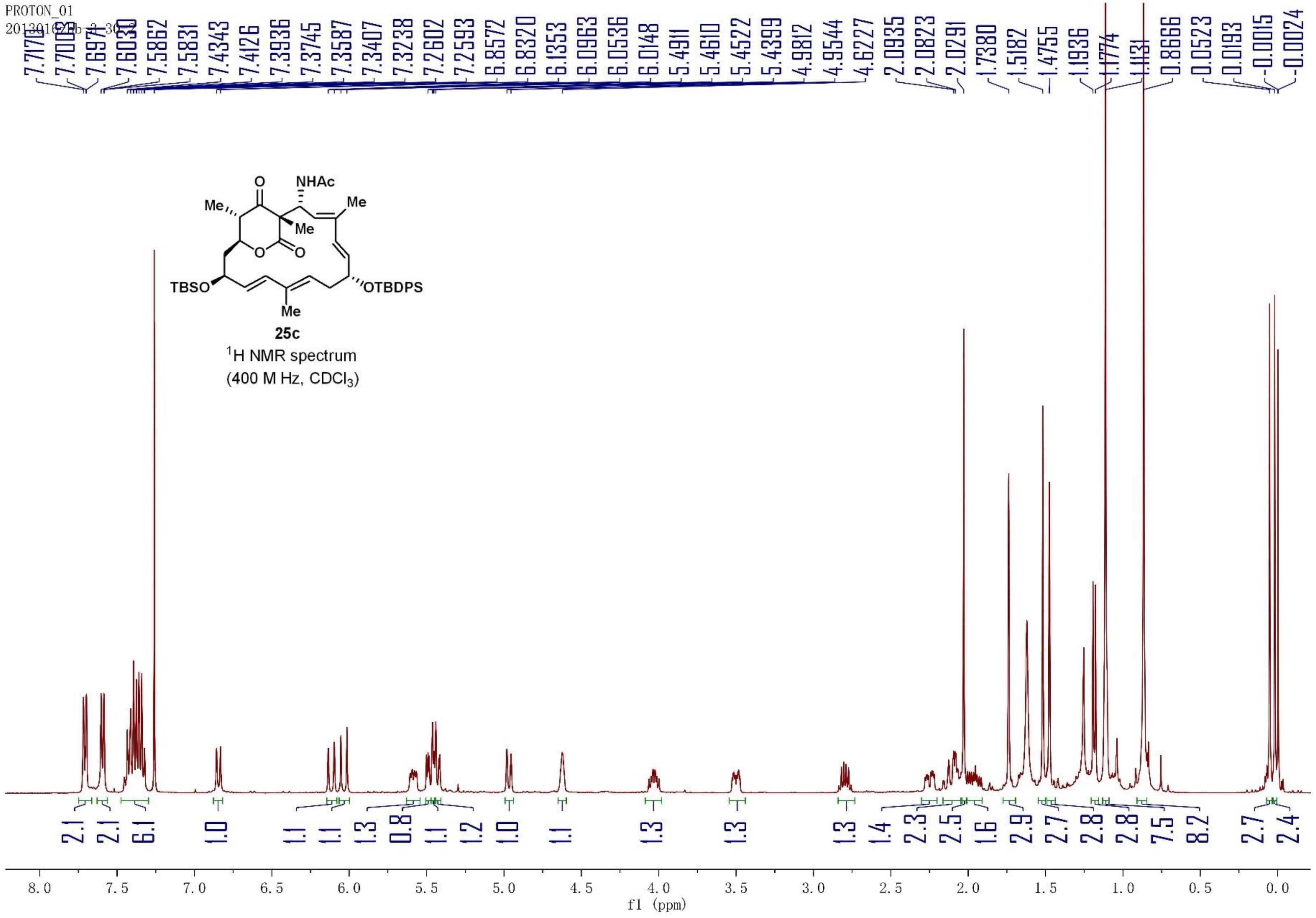
PROTON_01
25c
^{1}H NMR spectrum
(400 M Hz, CDCl$_3$)
f1 (ppm)

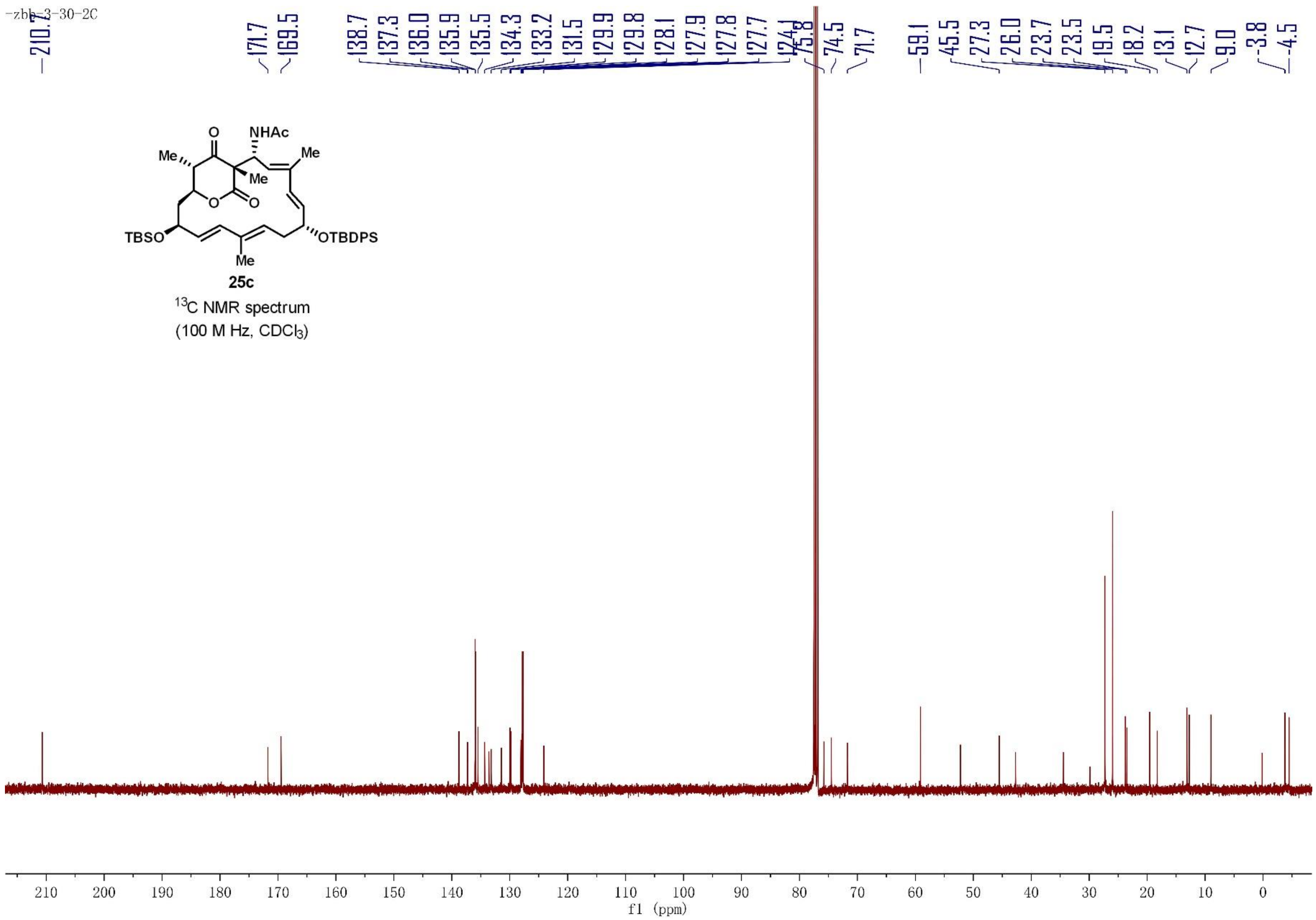

25c

^{13}C NMR spectrum

(100 M Hz, $CDCl_3$)

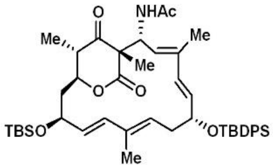

25c

H-H COSY spectrum
(400 M Hz, $CDCl_3$)

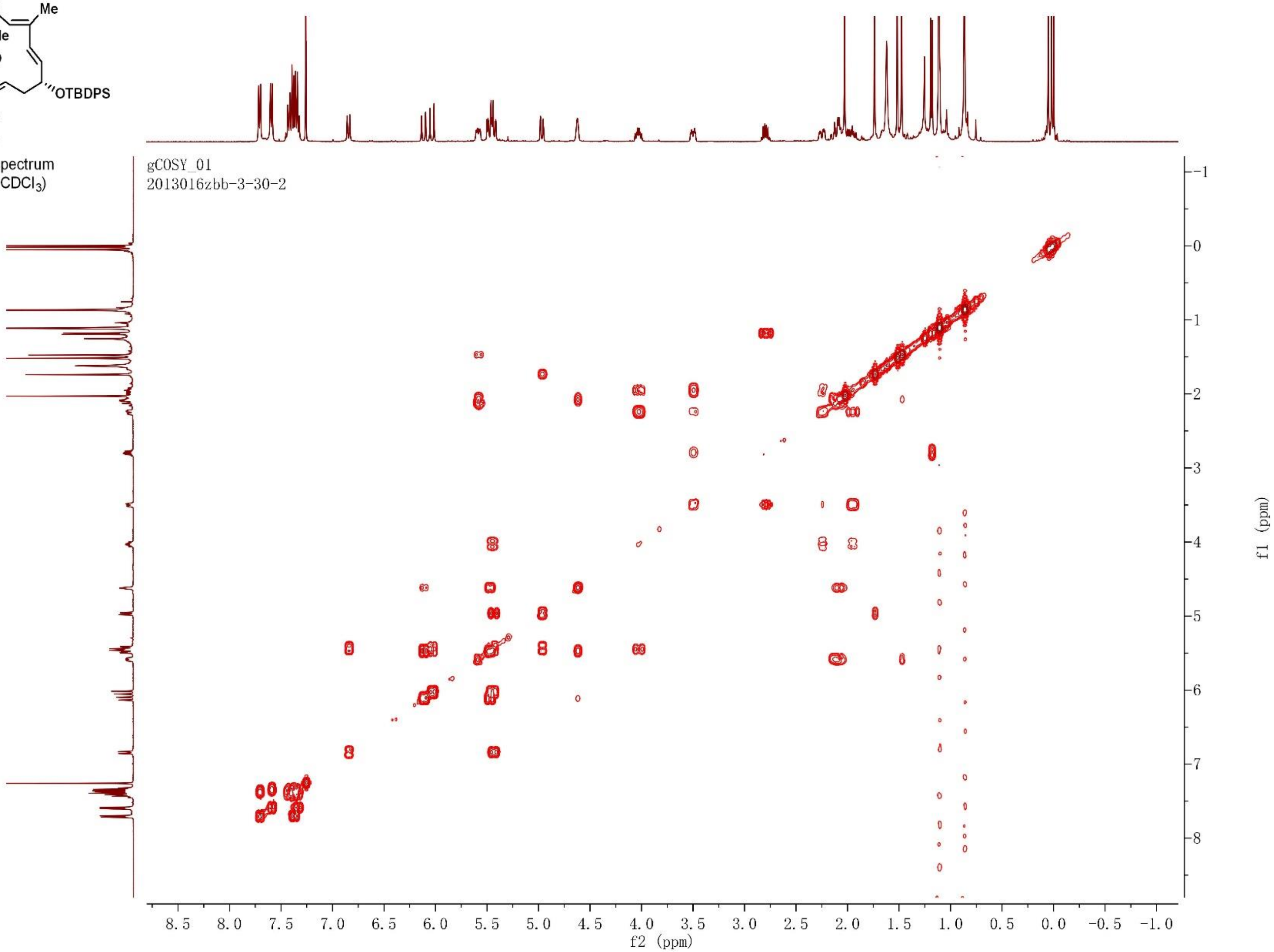

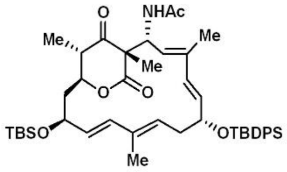

25c

NOESY spectrum
(400 M Hz, $CDCl_3$)

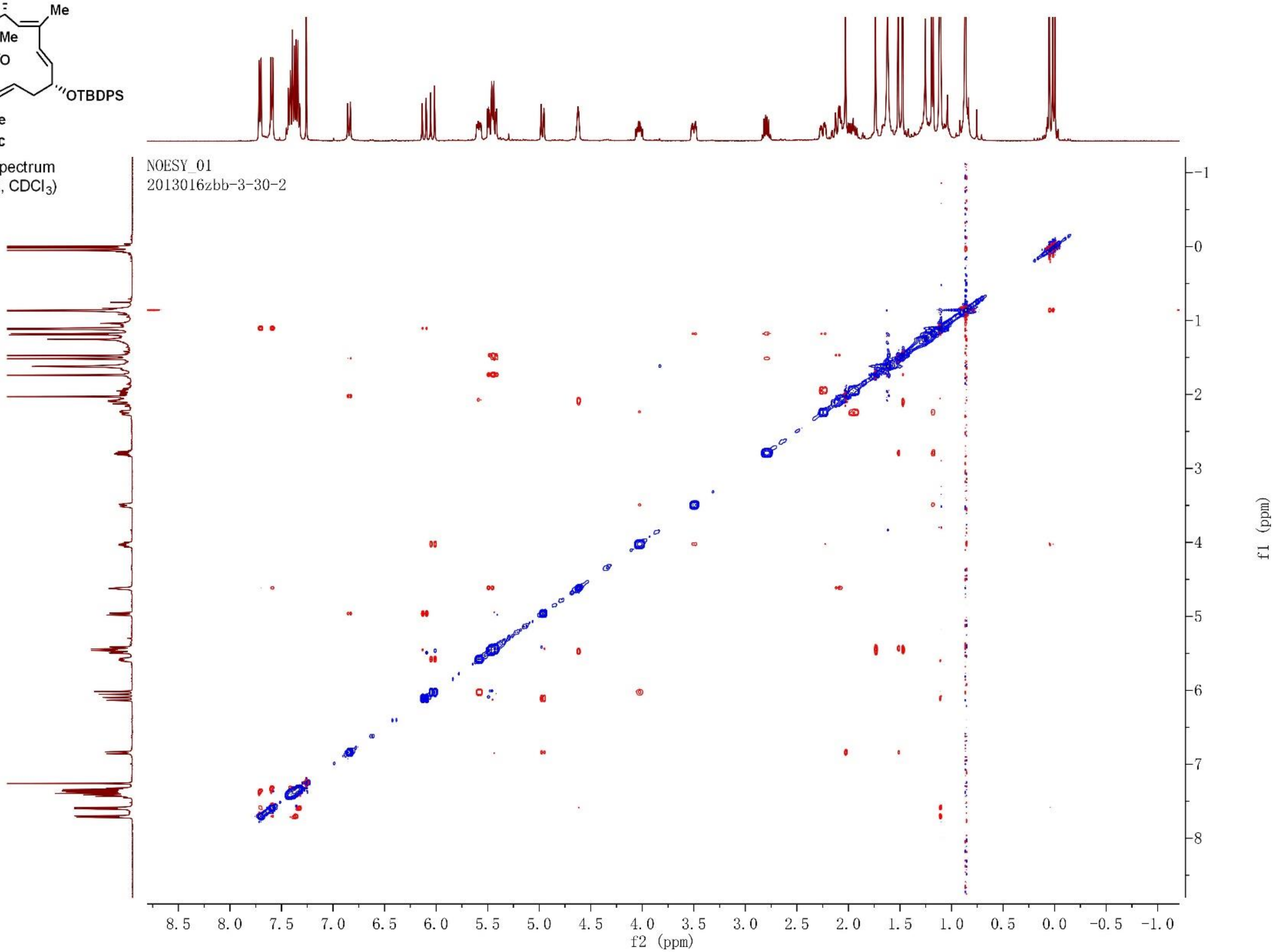

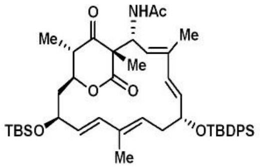

25c

C-H HSQC spectrum
(400 M Hz, $CDCl_3$)

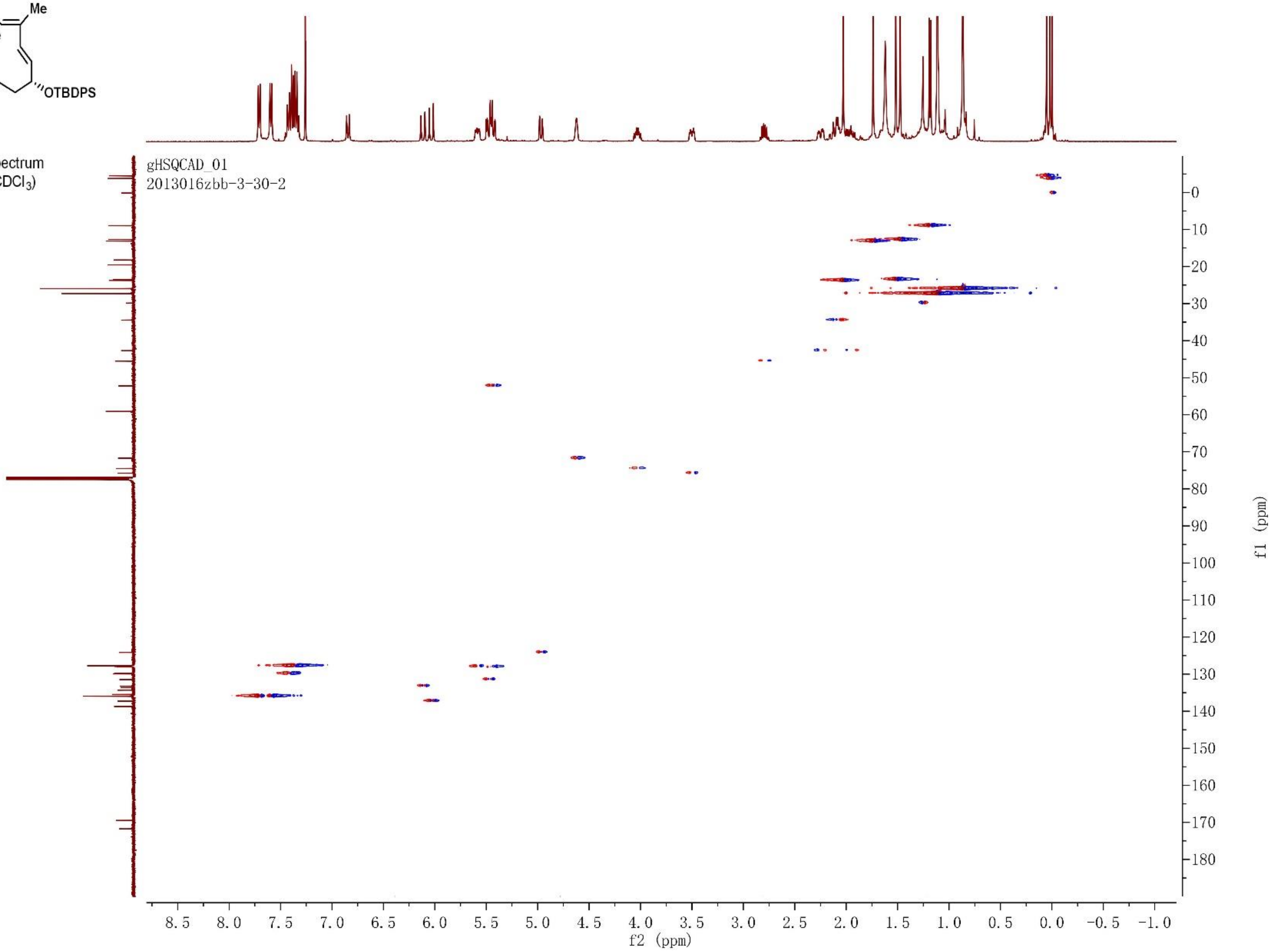

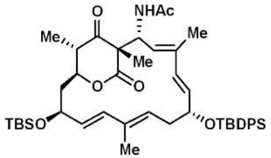

25c

C-H HMBC spectrum
(400 M Hz, $CDCl_3$)

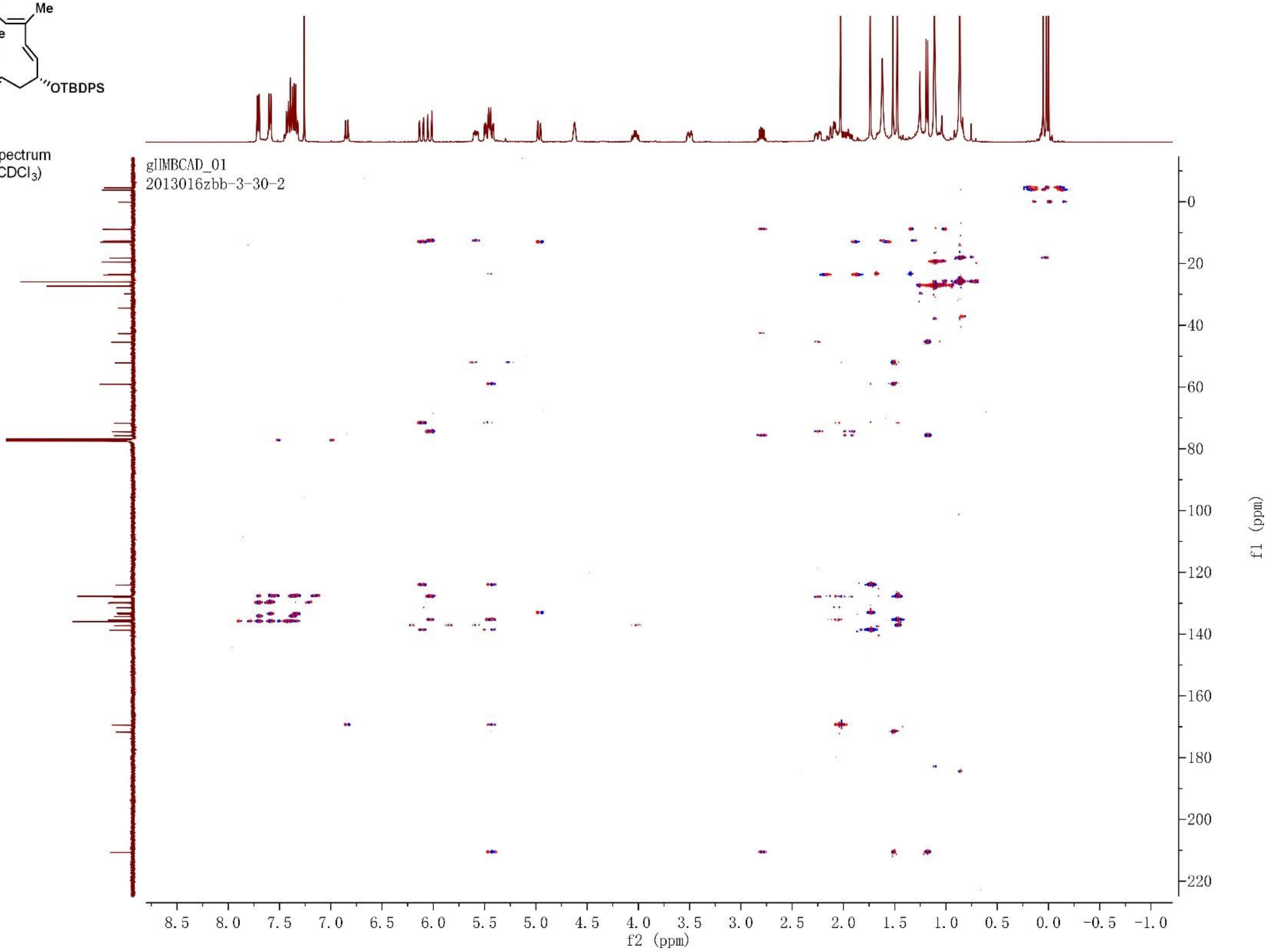

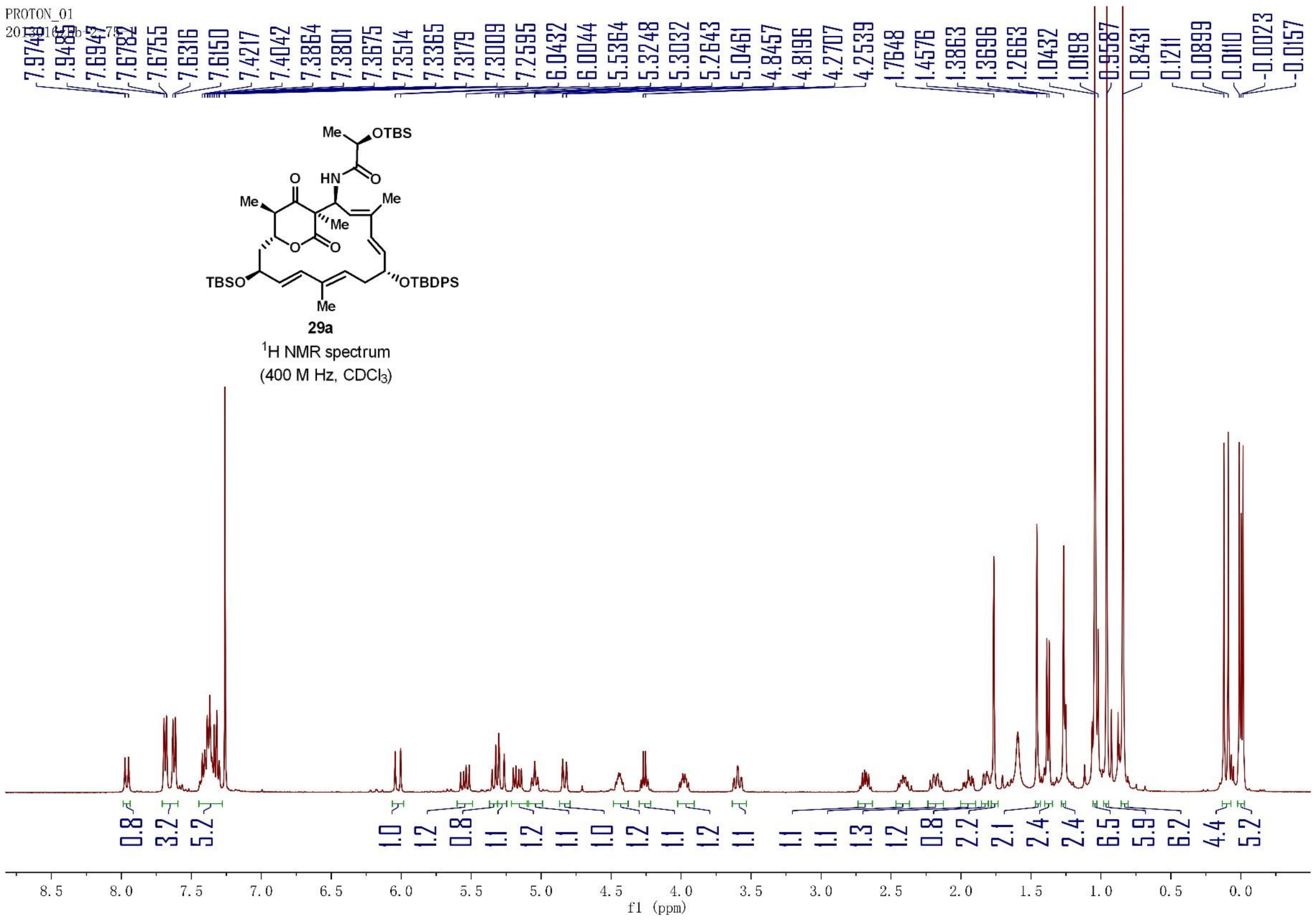

29a

^{1}H NMR spectrum

(400 M Hz, $CDCl_3$)

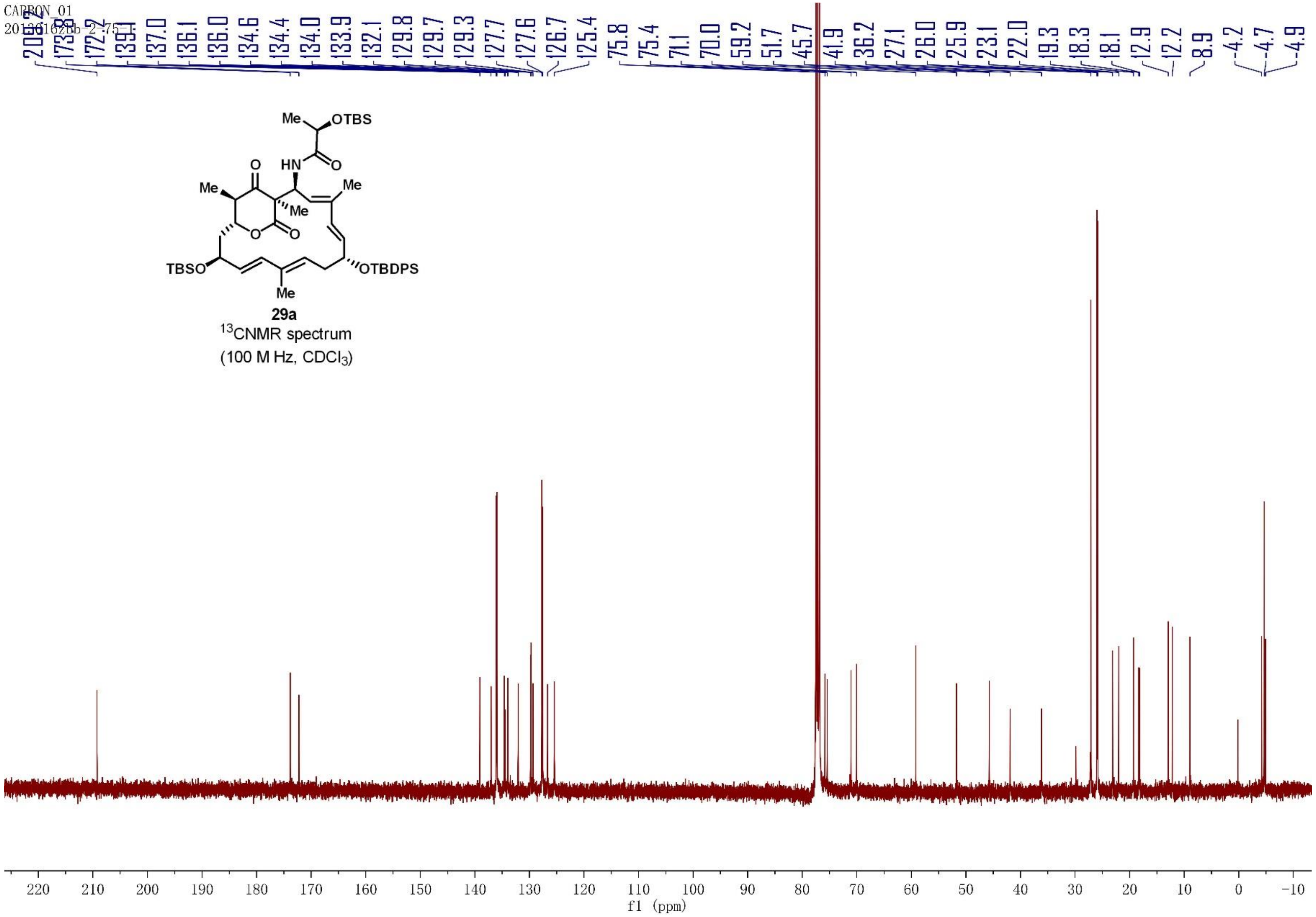

CARBON_01
2013061 6zbb-2-75-1
173.8
172.2
139.1
137.0
136.1
136.0
134.6
134.4
134.0
133.9
132.1
129.8
129.7
129.3
127.7
127.6
126.7
125.4
75.8
75.4
71.1
70.0
59.2
51.7
45.7
41.9
36.2
27.1
26.0
25.9
23.1
22.0
19.3
18.3
18.1
12.9
12.2
8.9
-4.2
-4.7
-4.9
Me
OTBS
HN
O
Me
Me
TBSO
OTBDPS
29a
^{13}CNMR spectrum
(100 M Hz, $CDCl_3$)
220 210 200 190 180 170 160 150 140 130 120 110 100 90 80 70 60 50 40 30 20 10 0 −10
f1 (ppm)

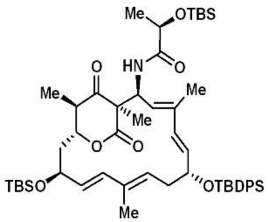

29a

H-H COSY spectrum
(400 M Hz, $CDCl_3$)

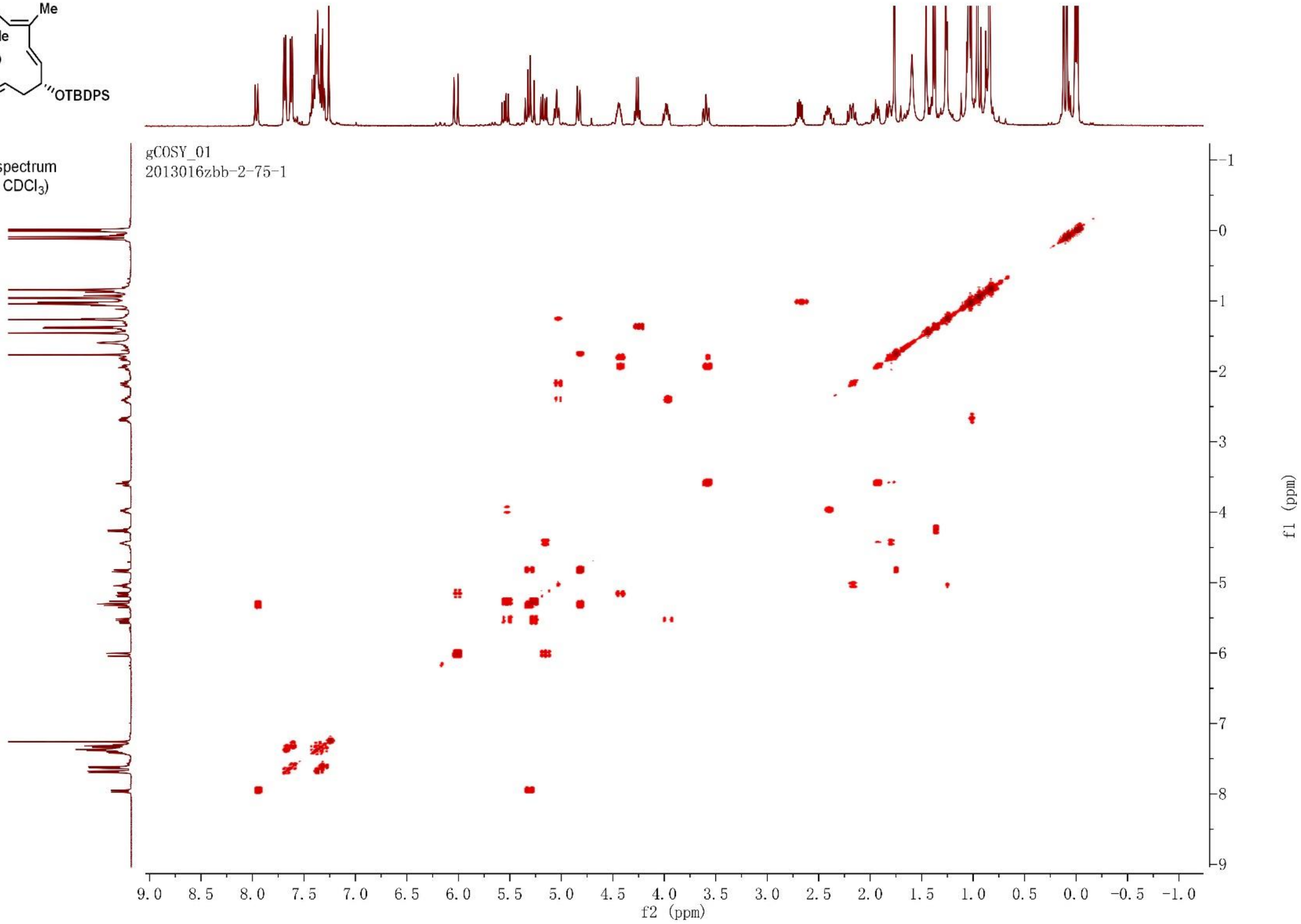

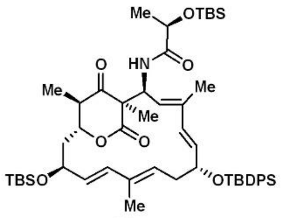

29a

NOESY spectrum
(400 M Hz, $CDCl_3$)

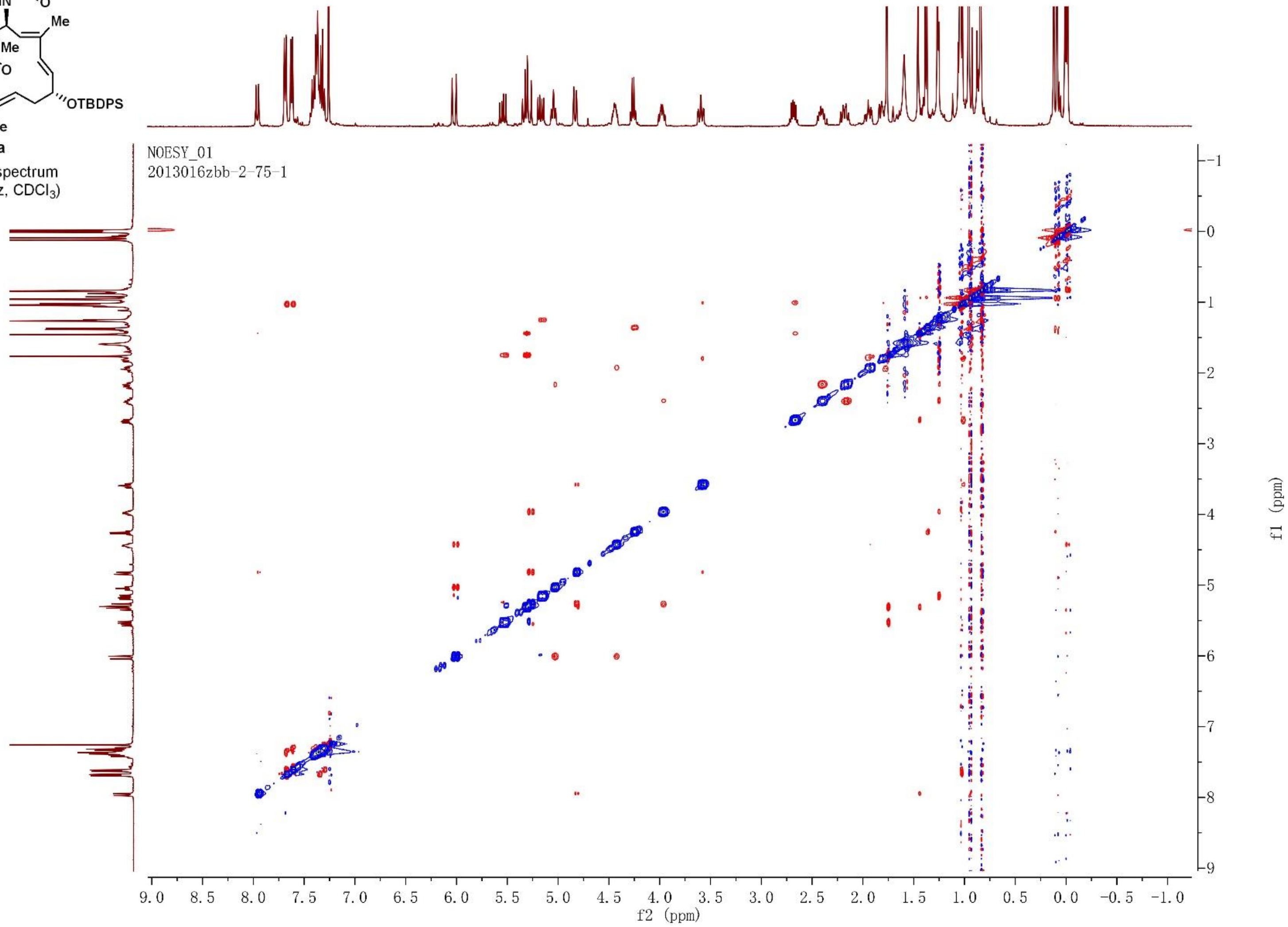

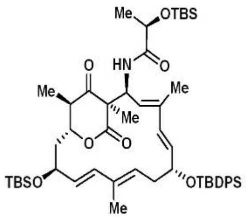

29a

C-H HSQC spectrum
(400 M Hz, $CDCl_3$)

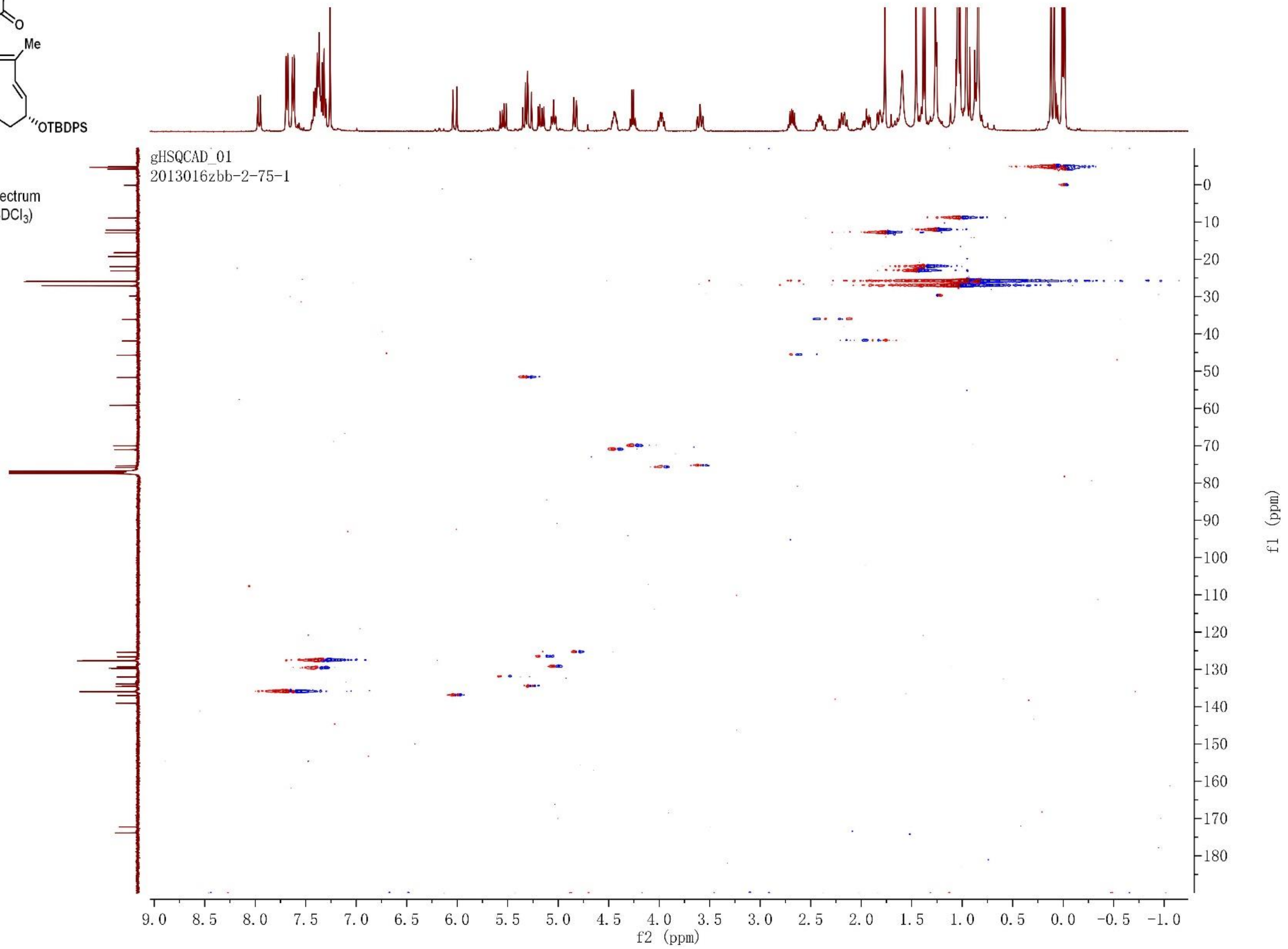

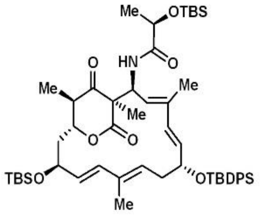

29a

C-H HMBC spectrum
(400 M Hz, $CDCl_3$)

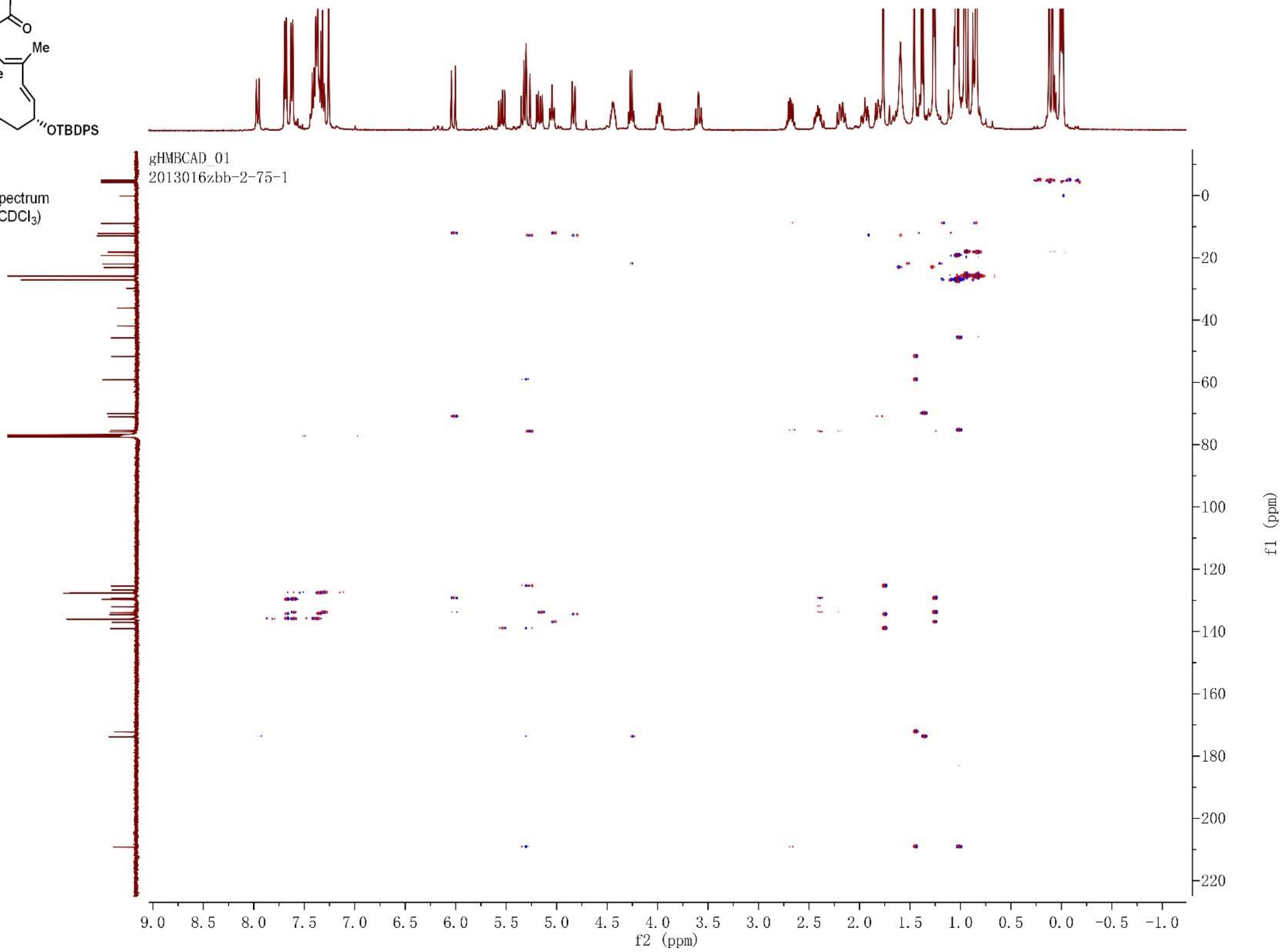

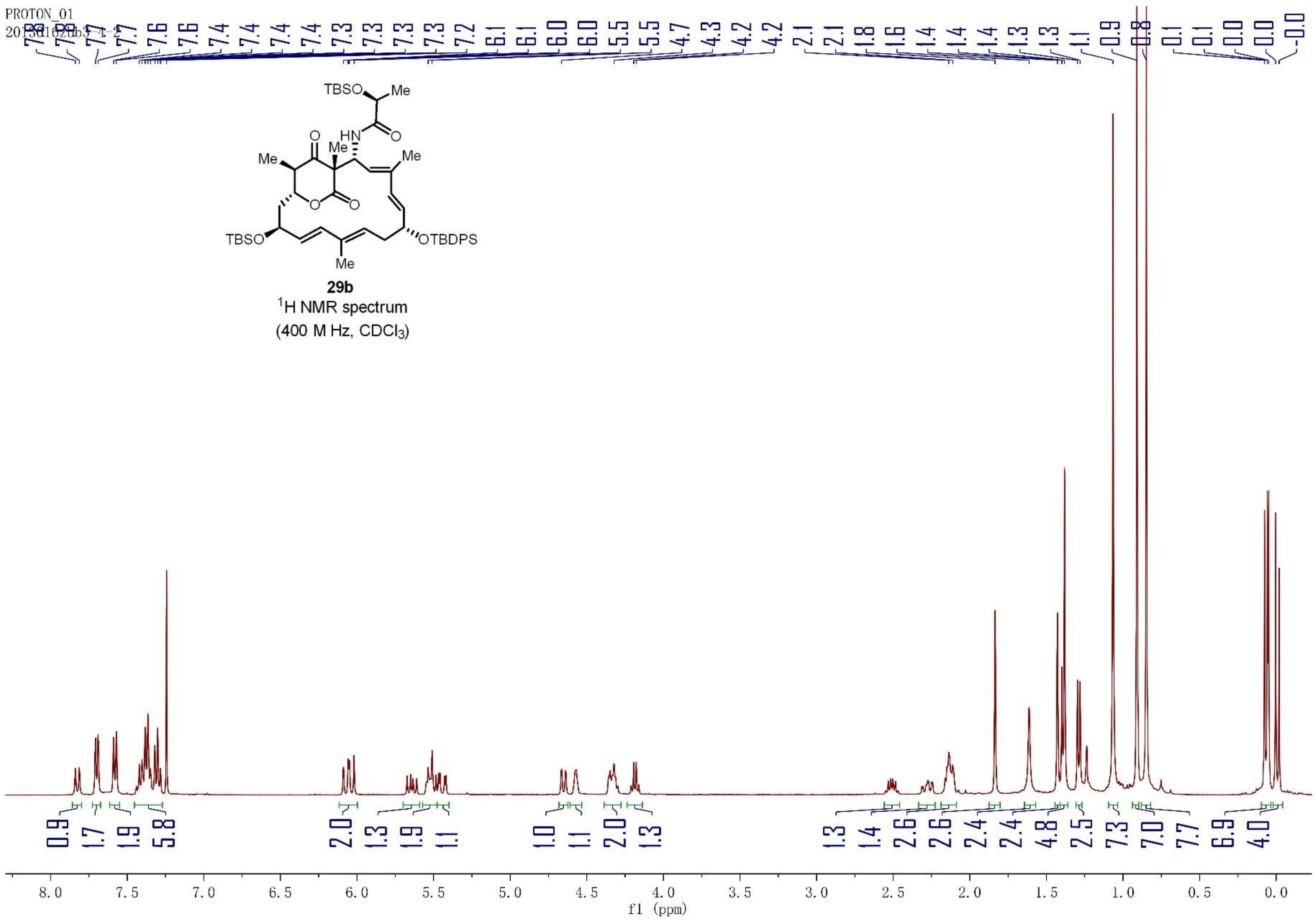
PROTON_01
TBSO
Me
HN
O
Me
Me
O
Me
O
O
TBSO
OTBDPS
Me
29b
^{1}H NMR spectrum
(400 M Hz, CDCl$_3$)
8.0
7.5
7.0
6.5
6.0
5.5
5.0
4.5
4.0
3.5
3.0
2.5
2.0
1.5
1.0
0.5
0.0
f1 (ppm)

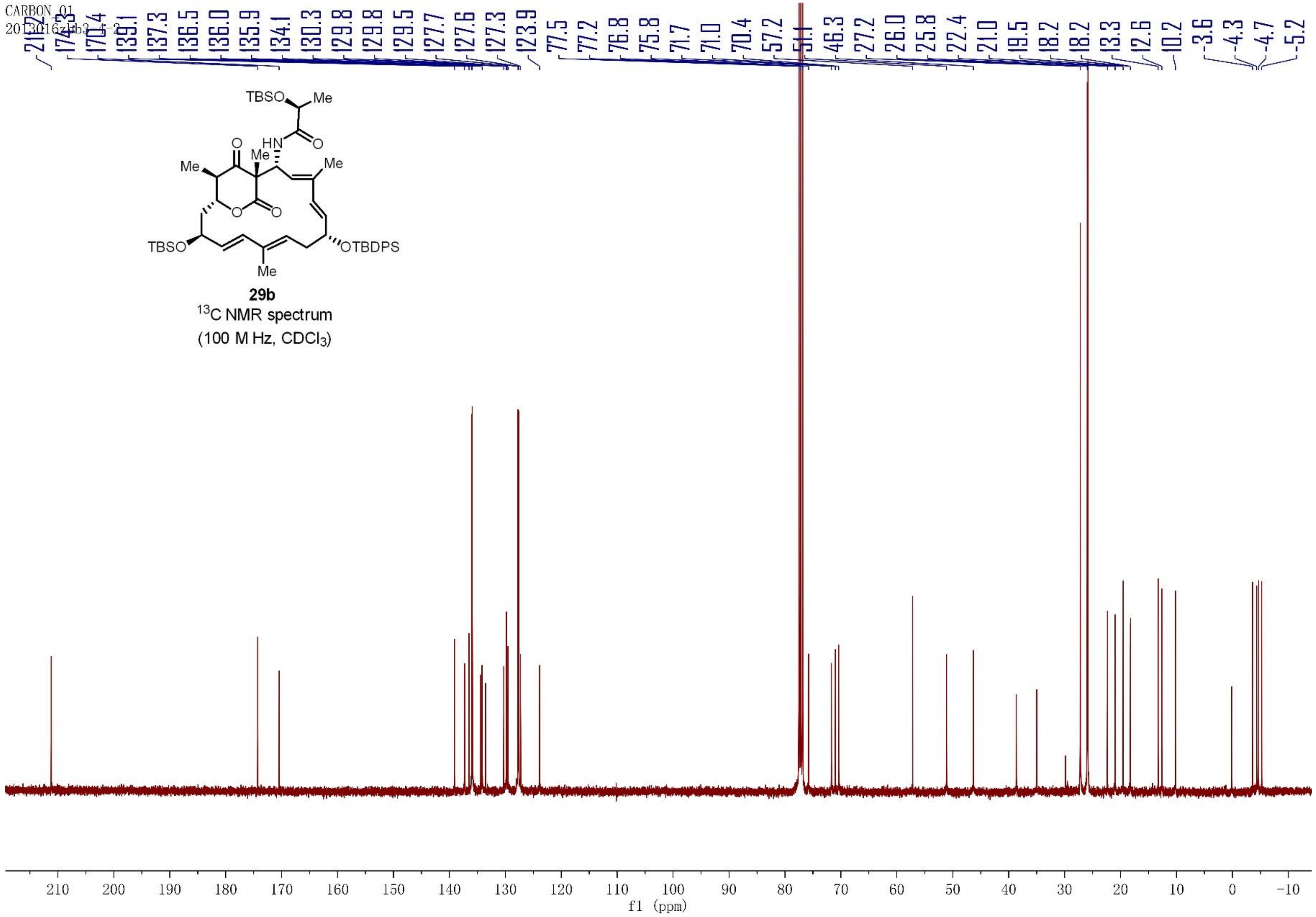
CARBON_01
2013016zfb3-4-2
211.2
174.3
170.4
139.1
137.3
136.5
136.0
135.9
134.1
130.3
129.8
129.8
129.5
127.7
127.6
127.3
123.9
77.5
77.2
76.8
75.8
71.7
71.0
70.4
57.2
51.1
46.3
27.2
26.0
25.8
22.4
21.0
19.5
18.2
18.2
13.3
12.6
10.2
-3.6
-4.3
-4.7
-5.2
TBSO
Me
O
HN
O
Me
Me
Me
O
O
TBSO
OTBDPS
Me
29b
^{13}C NMR spectrum
(100 M Hz, CDCl$_3$)
210
200
190
180
170
160
150
140
130
120
110
100
90
80
70
60
50
40
30
20
10
0
-10
f1 (ppm)

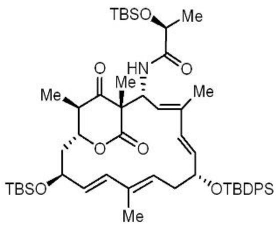

29b
H-H COSY spectrum
(400 M Hz, $CDCl_3$)

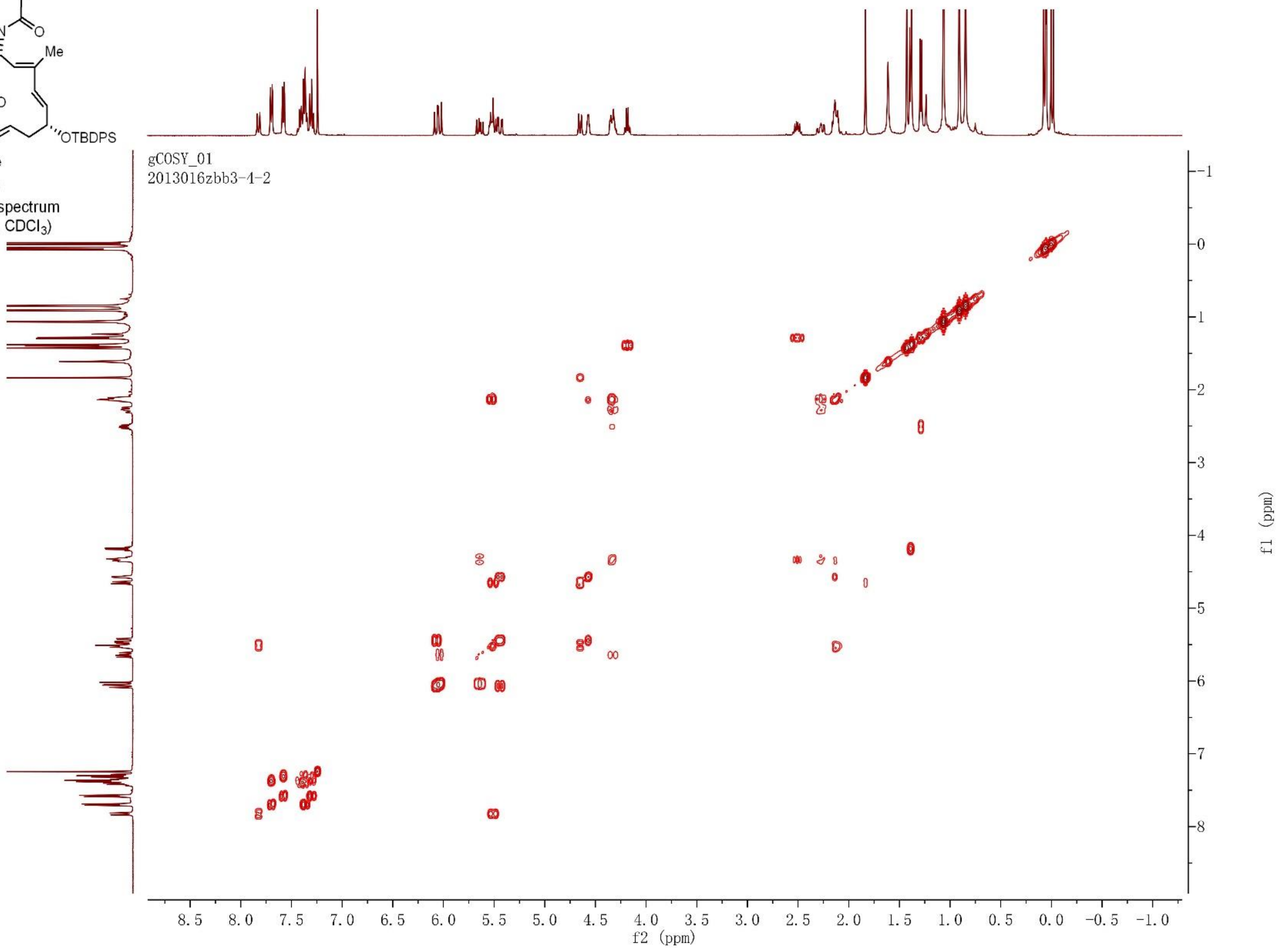

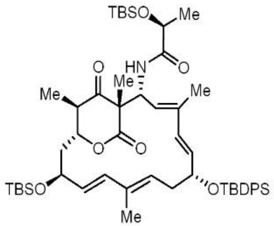

29b
NOESY spectrum
(400 M Hz, $CDCl_3$)

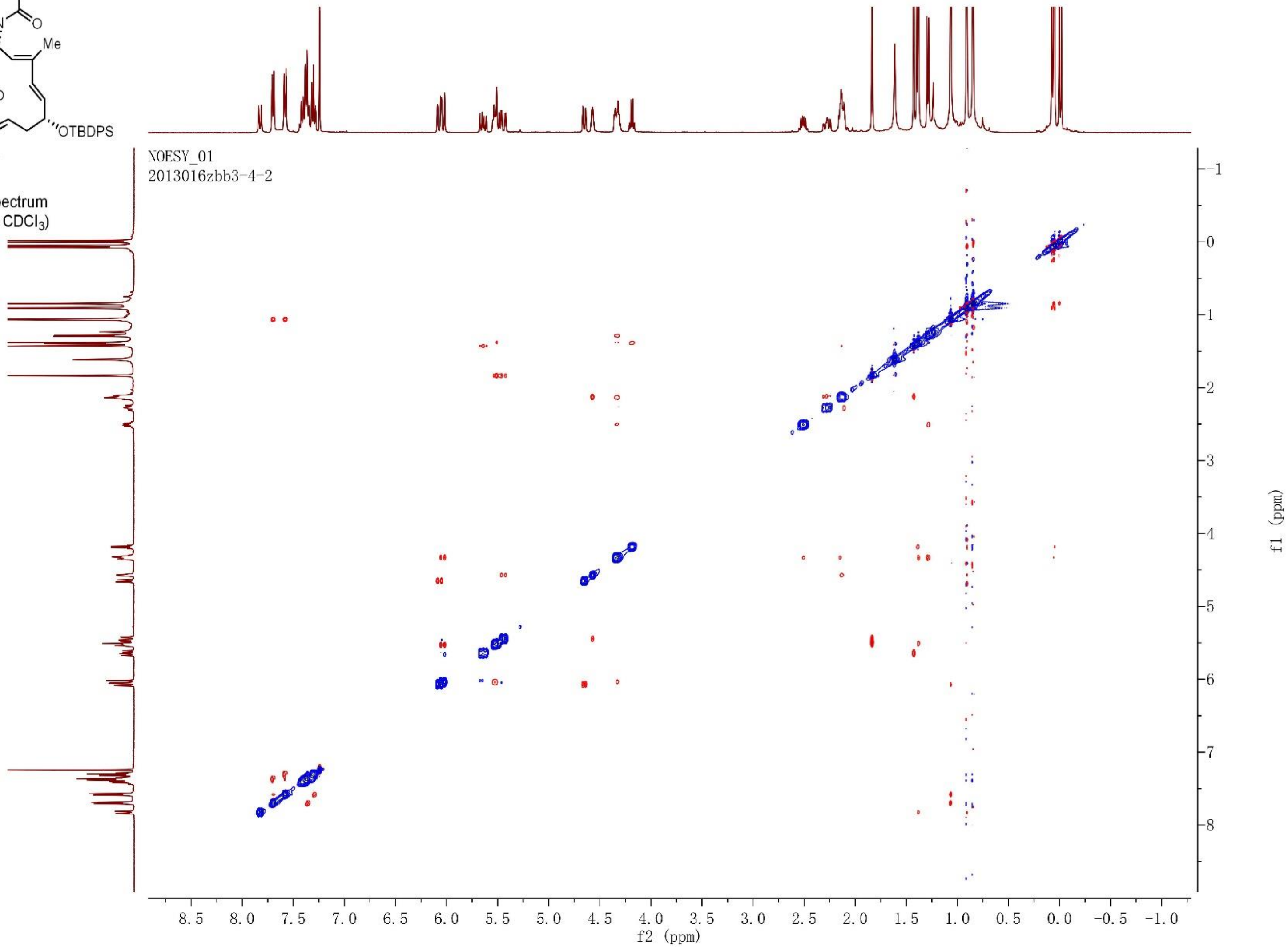

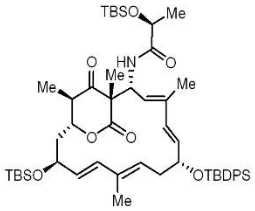

29b

C-H HSQC spectrum

(400 M Hz, $CDCl_3$)

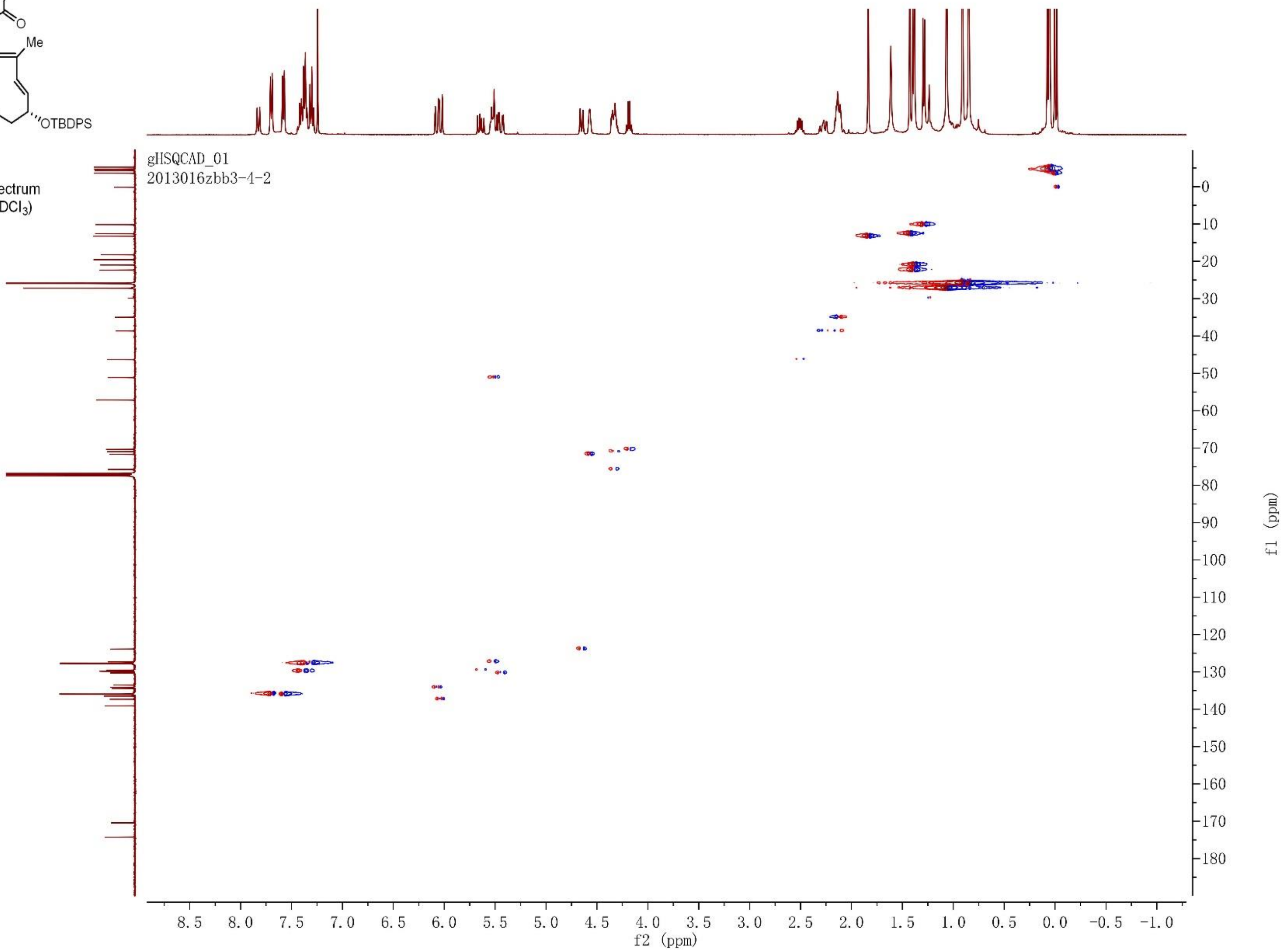

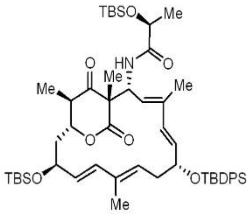

29b

C-H HMBC spectrum

(400 M Hz, $CDCl_3$)

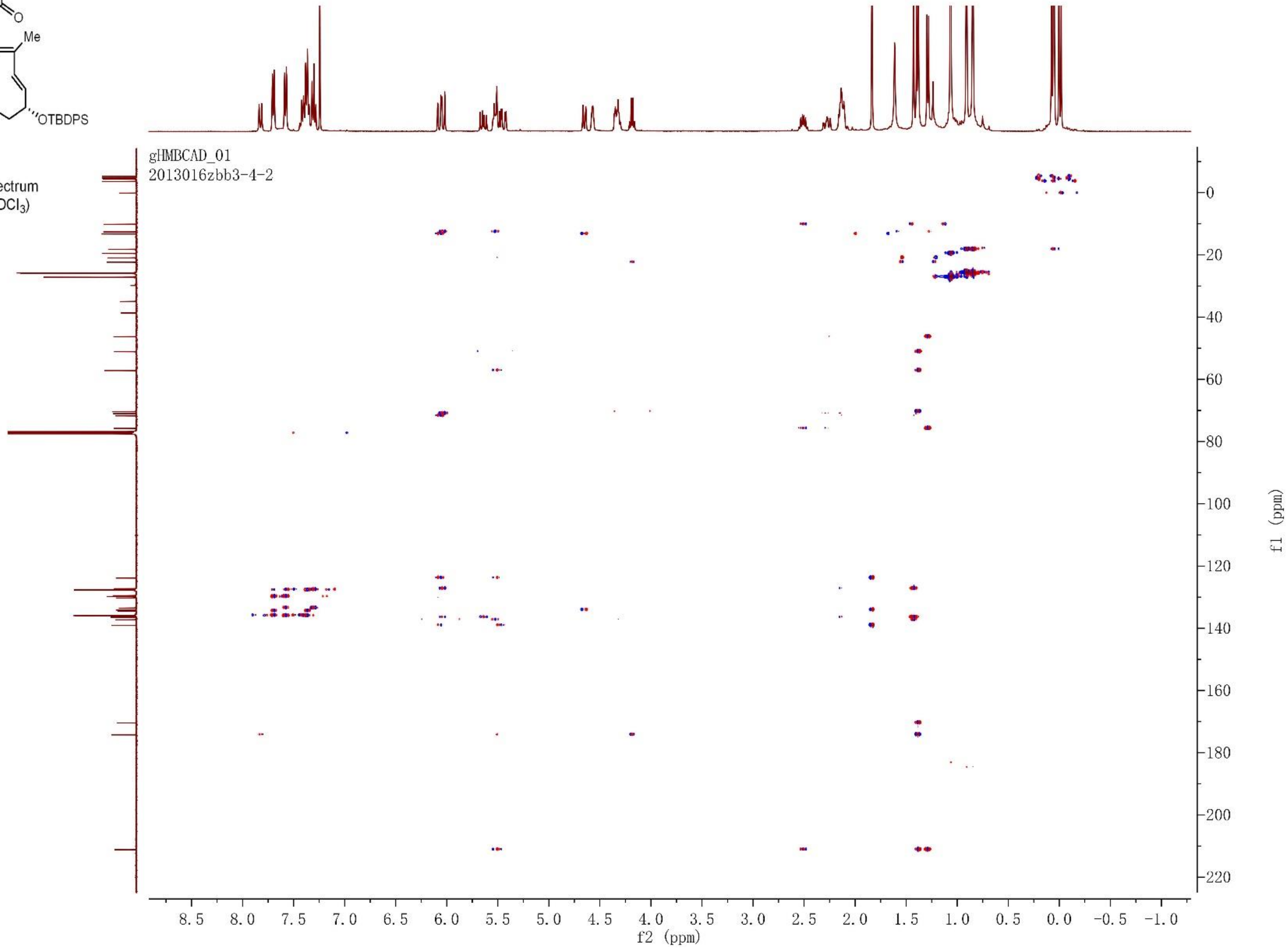

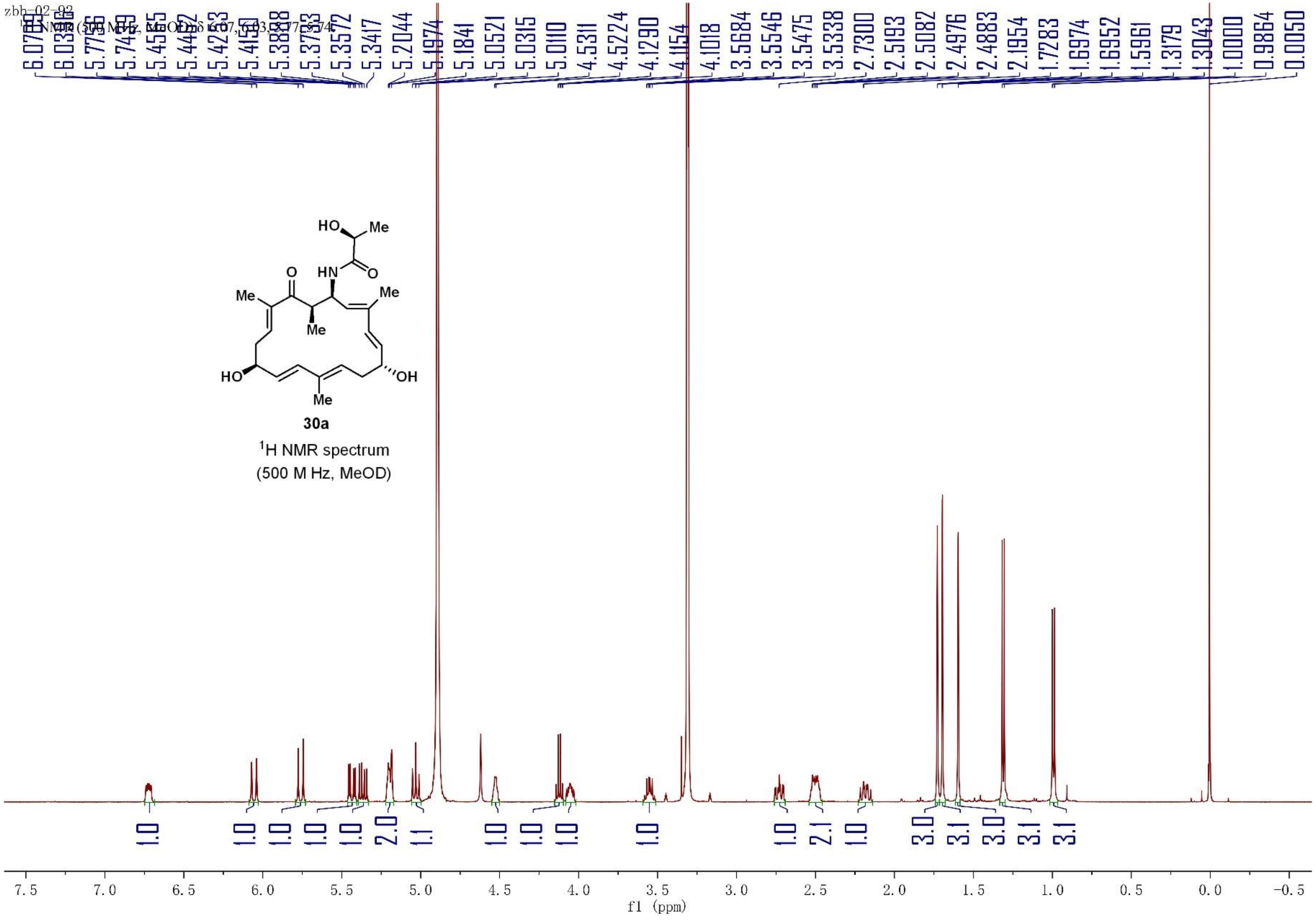

30a

^{1}H NMR spectrum

(500 M Hz, MeOD)

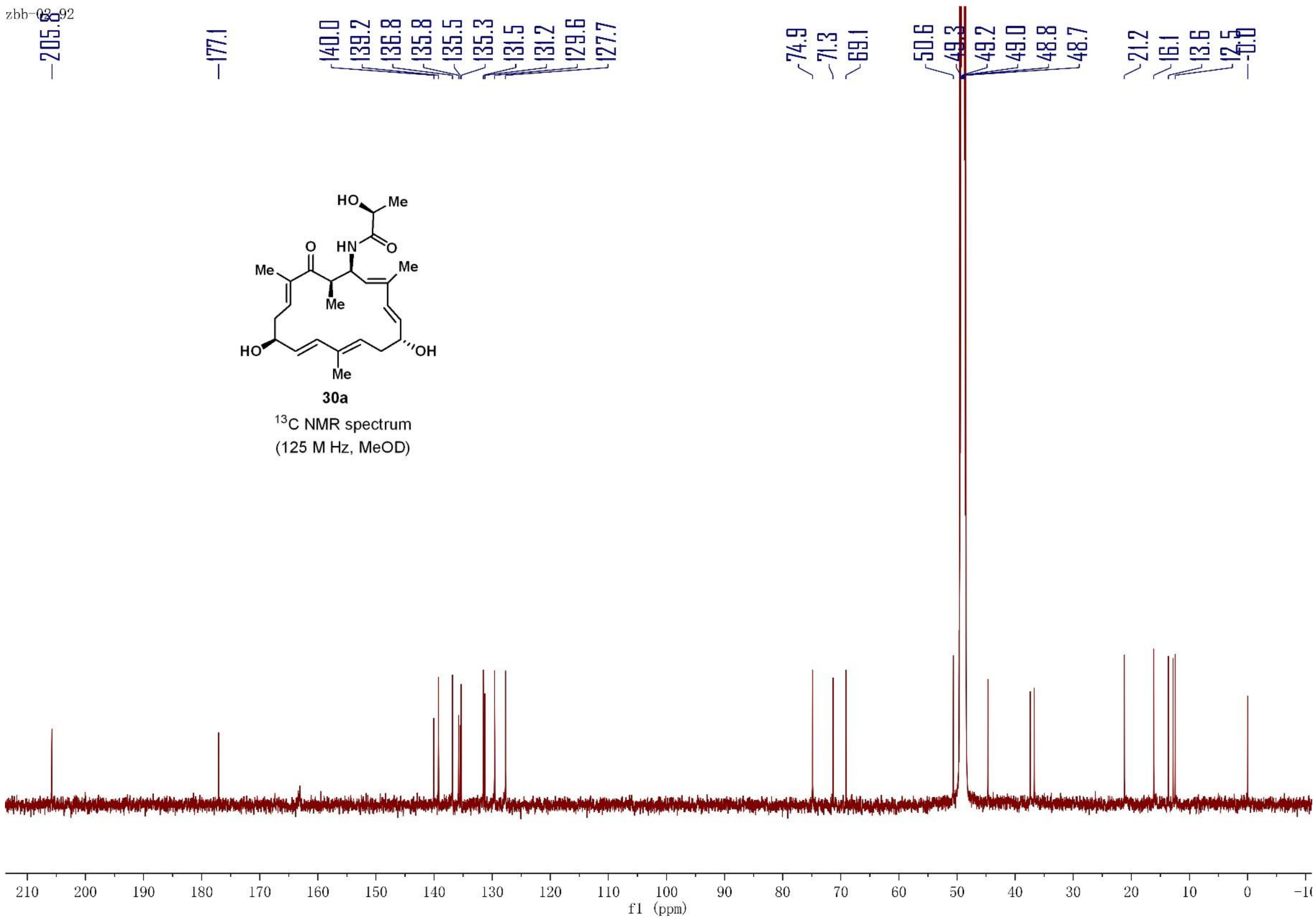

30a

^{13}C NMR spectrum

(125 M Hz, MeOD)

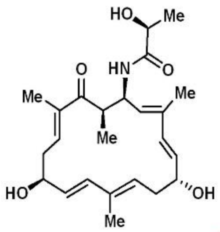

30a

H-H COSY spectrum
(500 M Hz, MeOD)

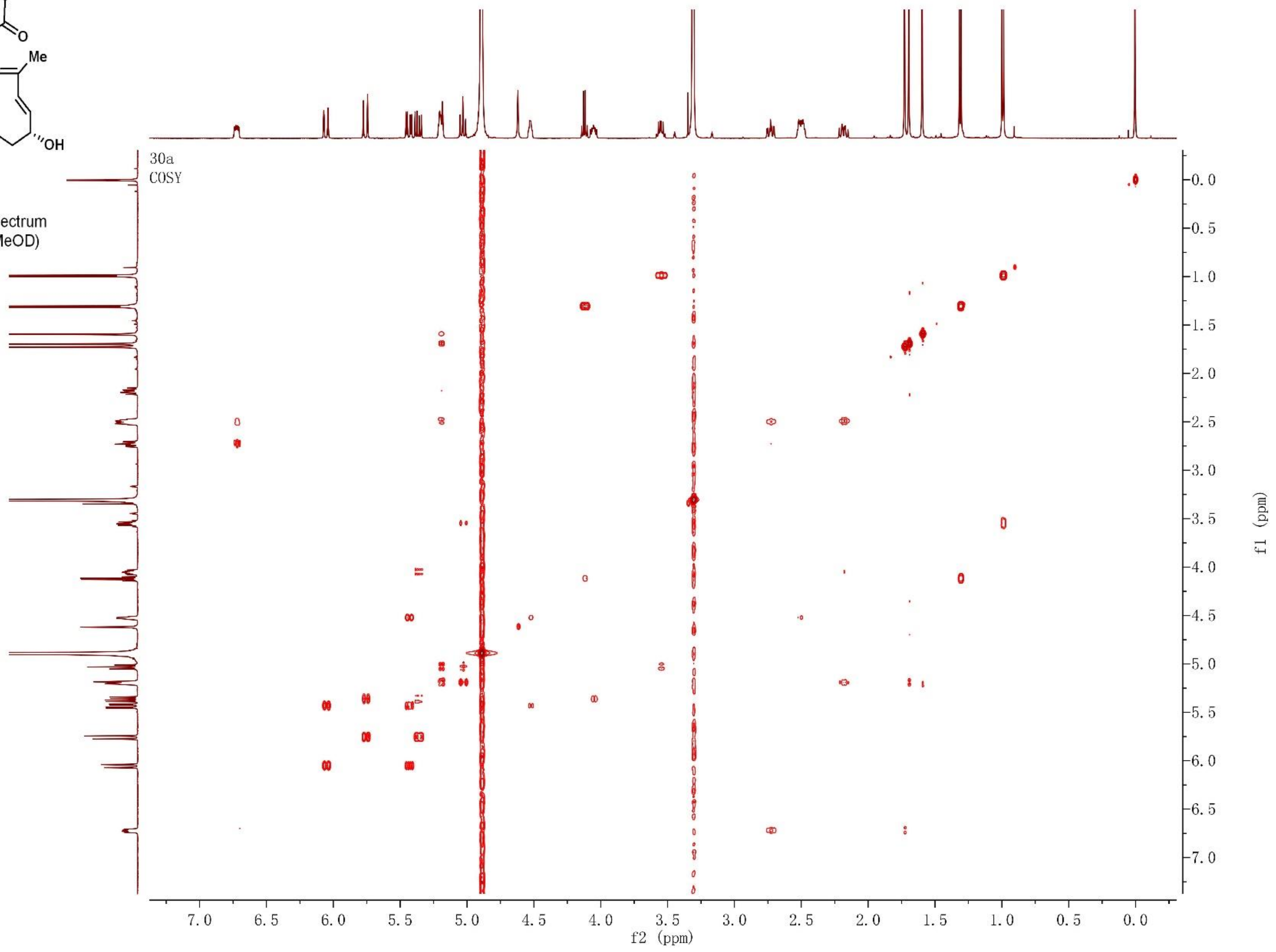

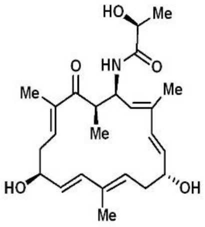

30a

NOESY spectrum
(500 M Hz, MeOD)

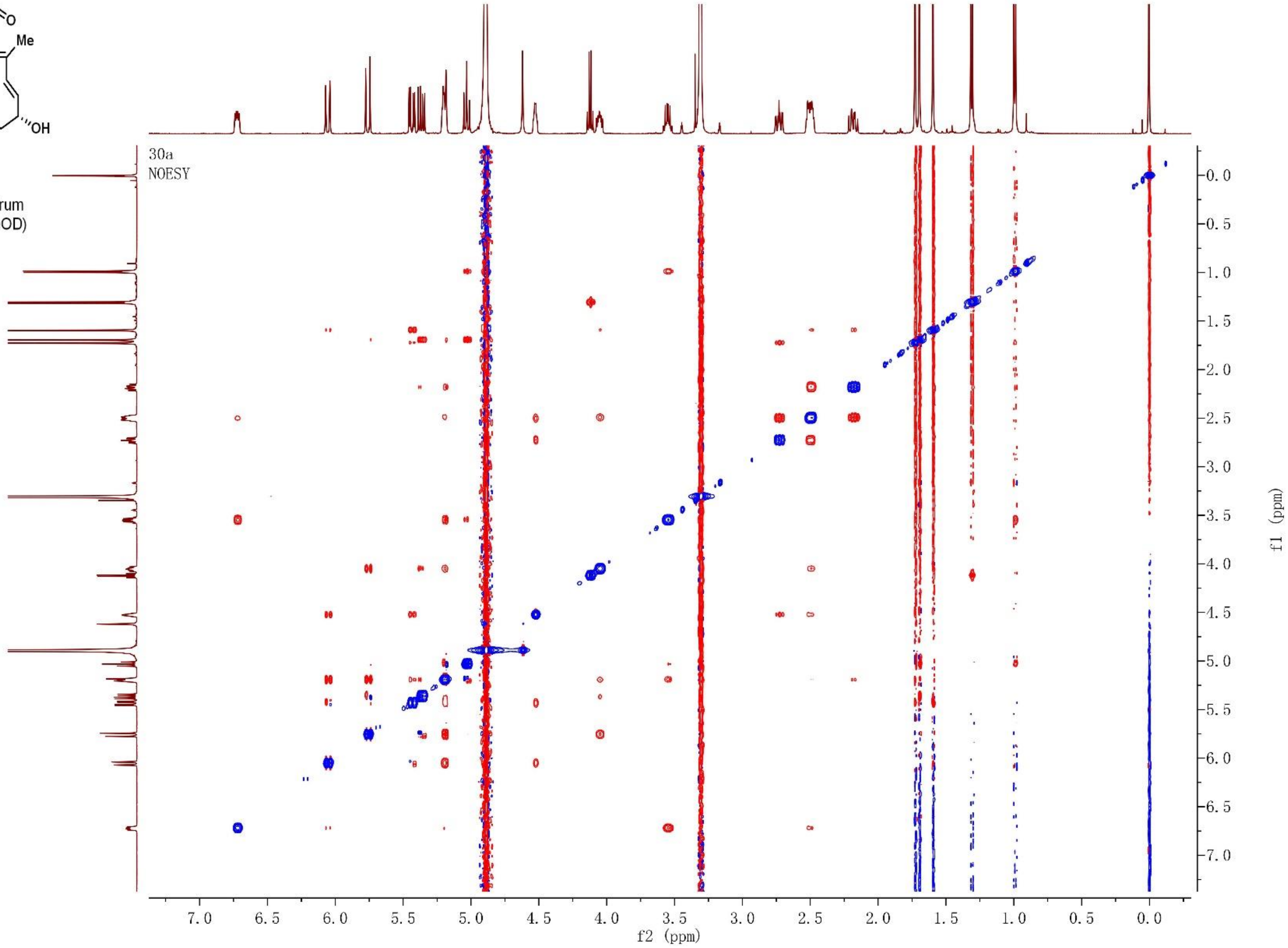

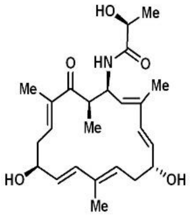

30a

C-H HSQC spectrum
(500 M Hz, MeOD)

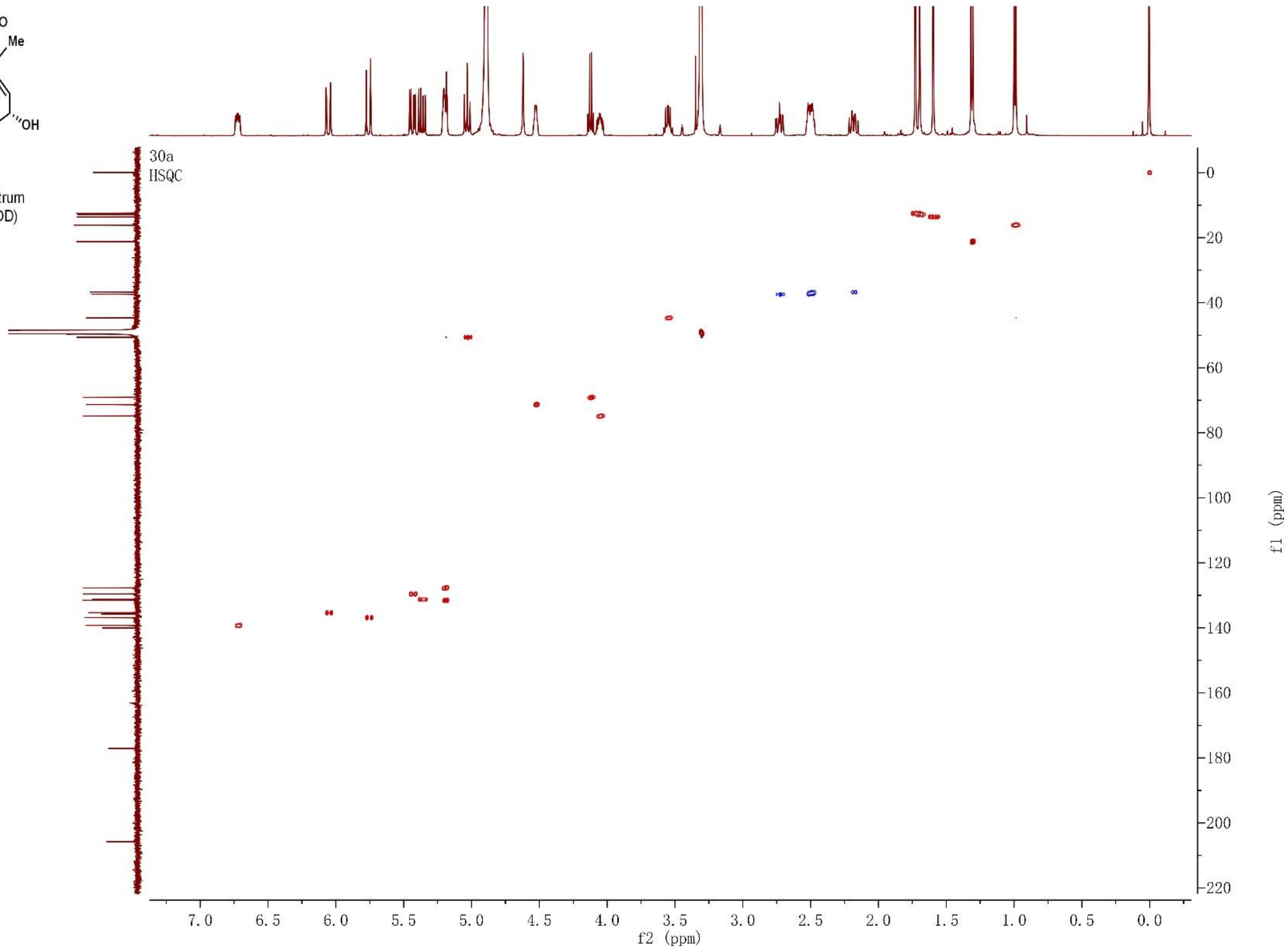

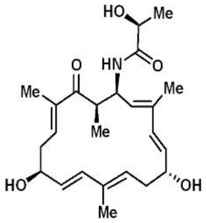

30a

C-H HMBC spectrum
(500 M Hz, MeOD)

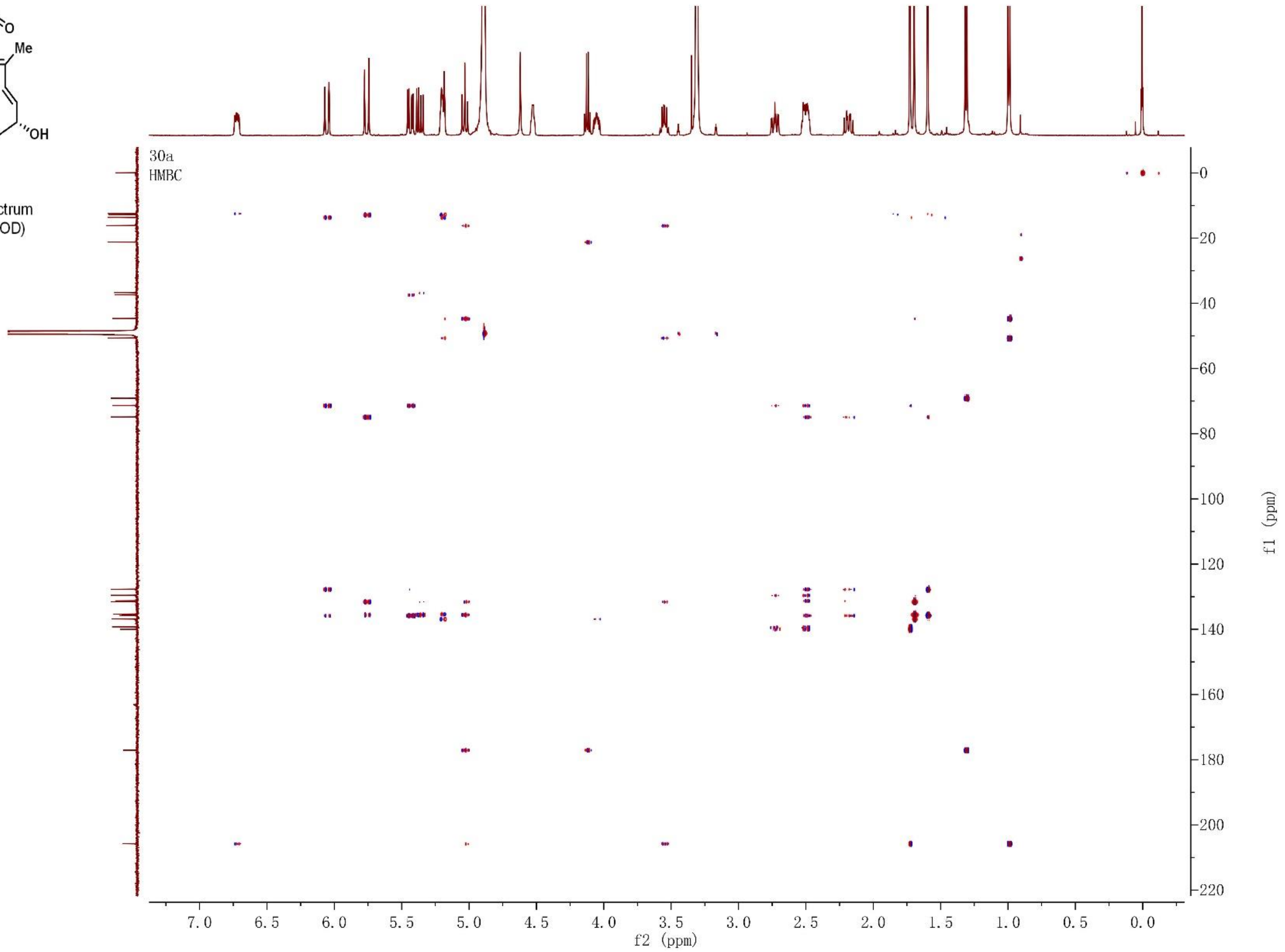

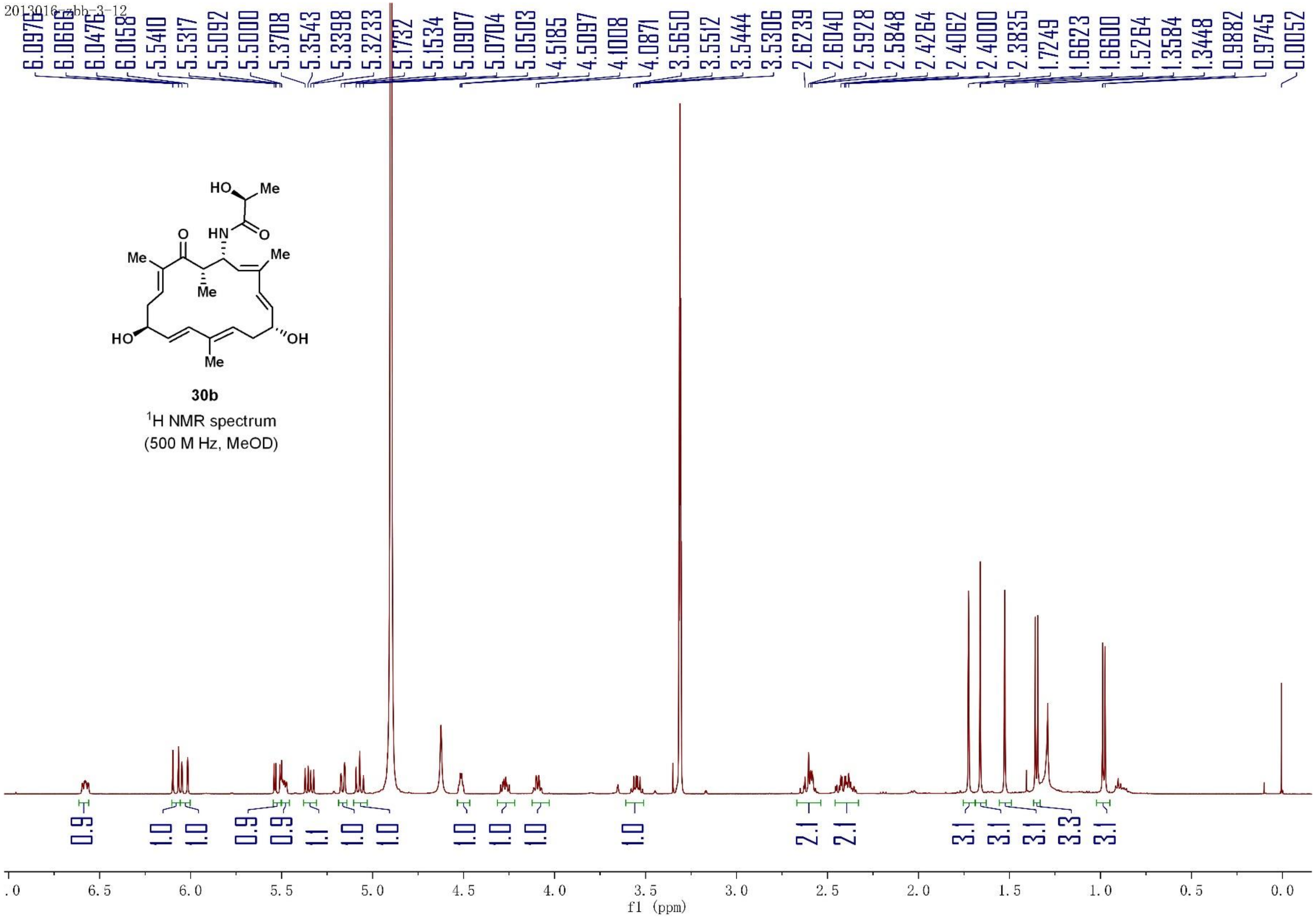

30b

^{1}H NMR spectrum

(500 M Hz, MeOD)

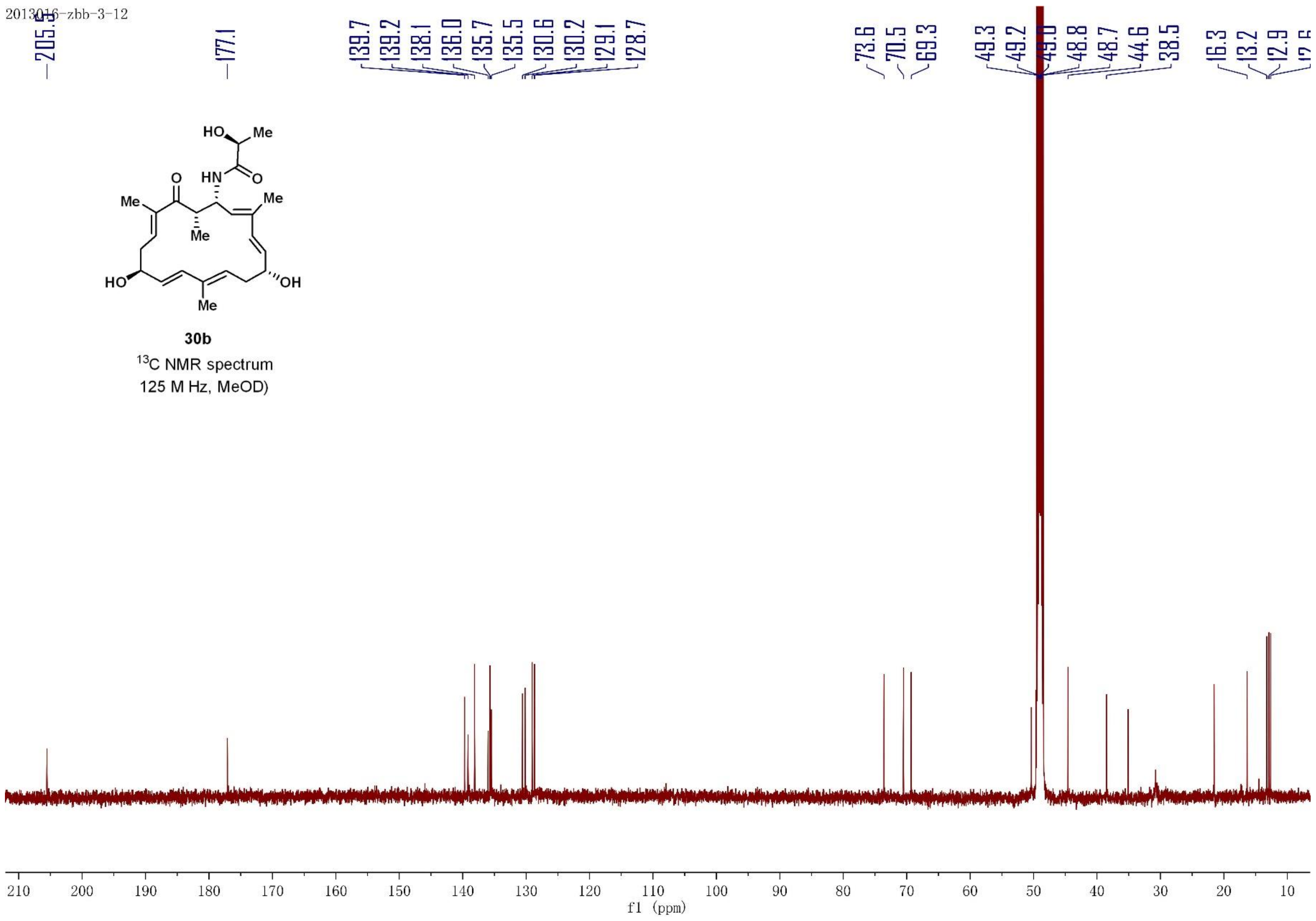

30b

^{13}C NMR spectrum

125 M Hz, MeOD)

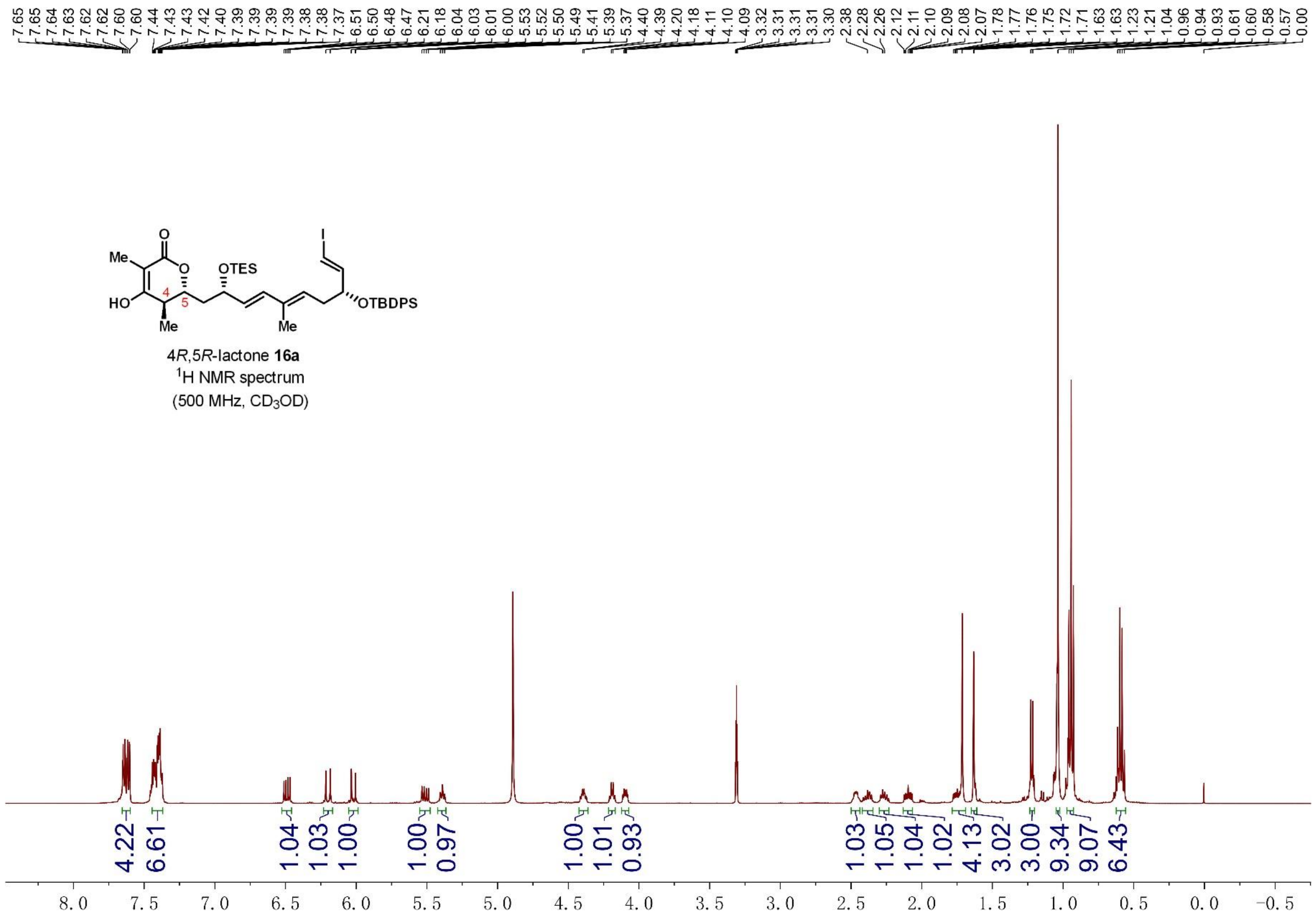
4R,5R-lactone 16a
1H NMR spectrum
(500 MHz, CD3OD)
OTES
OTBDPS
HO
Me
I

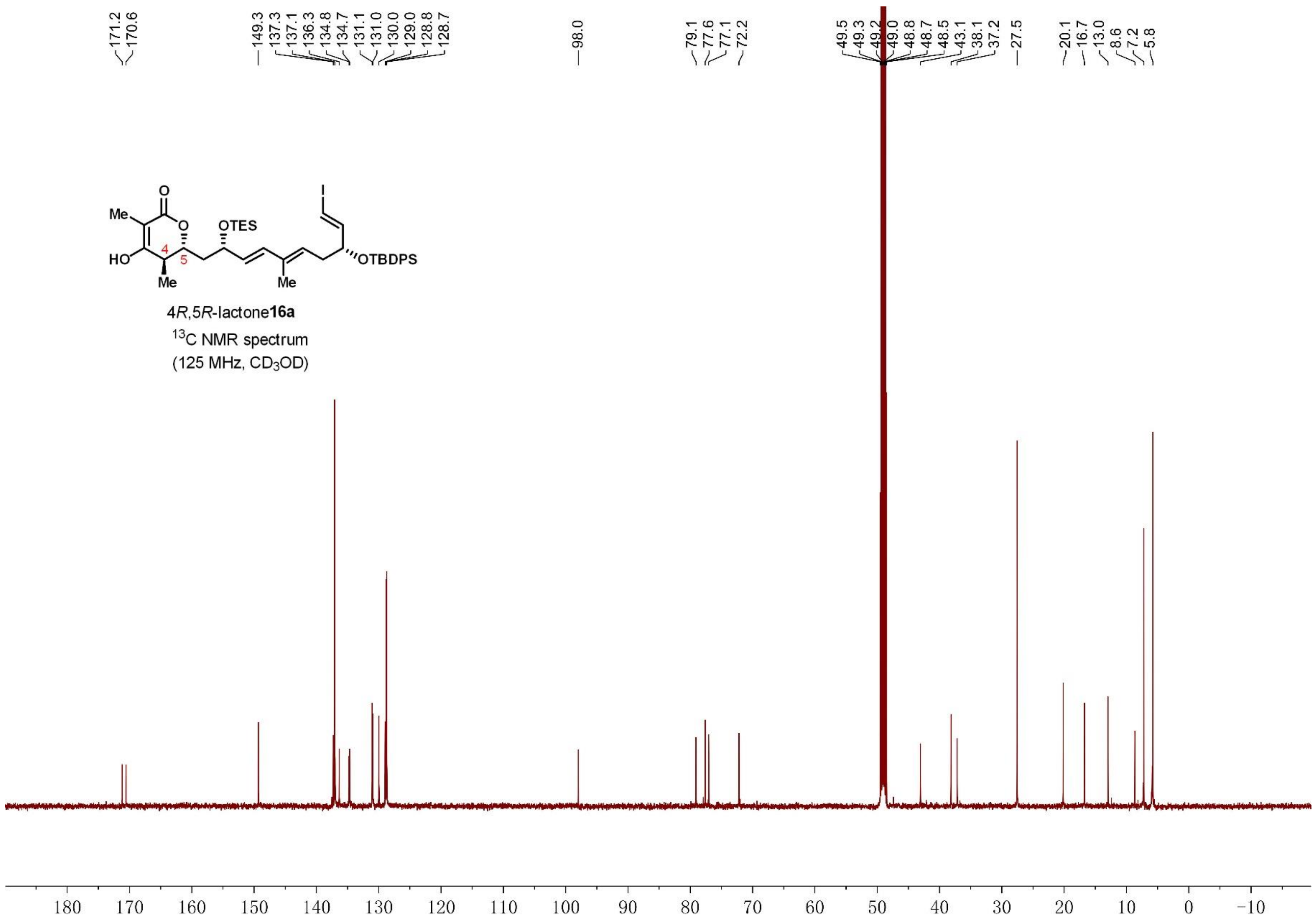
171.2
170.6
149.3
137.3
137.1
136.3
134.8
134.7
131.1
131.0
130.0
129.0
128.8
128.7
98.0
79.1
77.6
77.1
72.2
49.5
49.3
49.2
49.0
48.8
48.7
48.5
43.1
38.1
37.2
27.5
20.1
16.7
13.0
8.6
7.2
5.8
4R,5R-lactone16a
13C NMR spectrum
(125 MHz, CD3OD)
180
170
160
150
140
130
120
110
100
90
80
70
60
50
40
30
20
10
0
−10

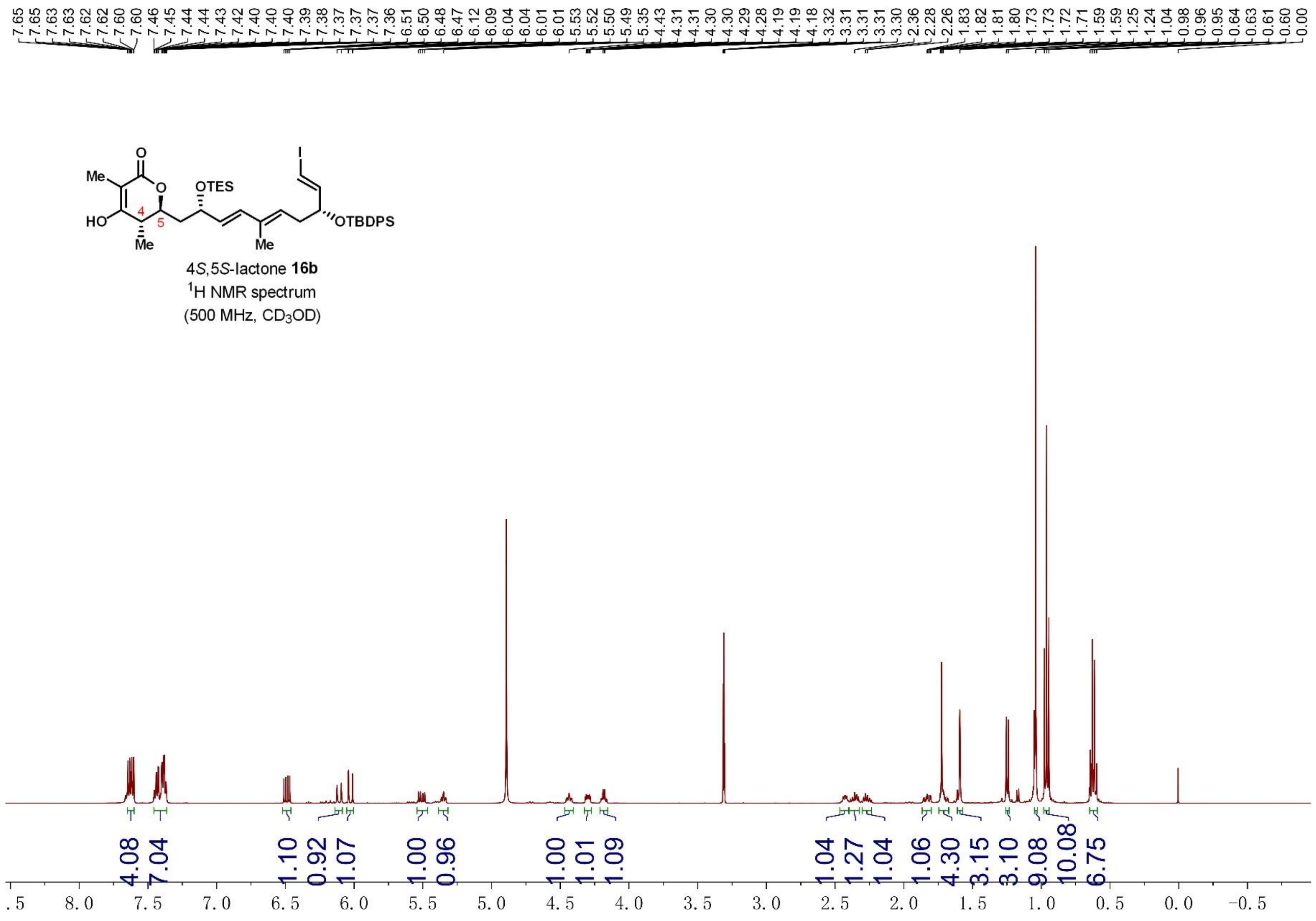
4S,5S-lactone 16b
1H NMR spectrum
(500 MHz, CD3OD)
OTES
OTBDPS
Me
HO
I

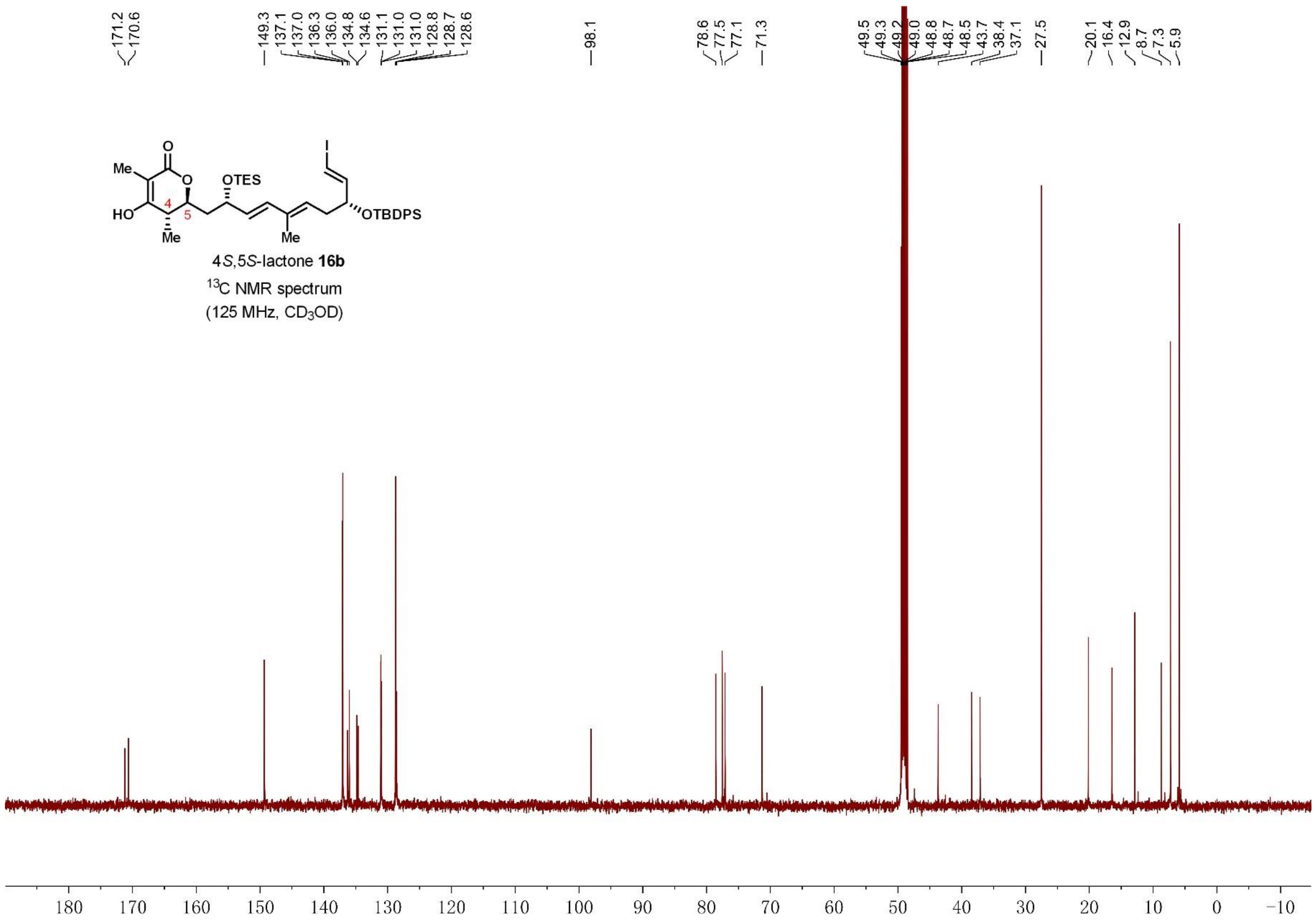
171.2
170.6
149.3
137.1
137.0
136.3
136.0
134.8
134.6
131.1
131.0
131.0
128.8
128.7
128.6
98.1
78.6
77.5
77.1
71.3
49.5
49.3
49.2
49.0
48.8
48.7
48.5
43.7
38.4
37.1
27.5
20.1
16.4
12.9
8.7
7.3
5.9
Me
O
OTES
I
HO
4
5
Me
Me
OTBDPS
4S,5S-lactone 16b
13C NMR spectrum
(125 MHz, CD3OD)
180
170
160
150
140
130
120
110
100
90
80
70
60
50
40
30
20
10
0
−10

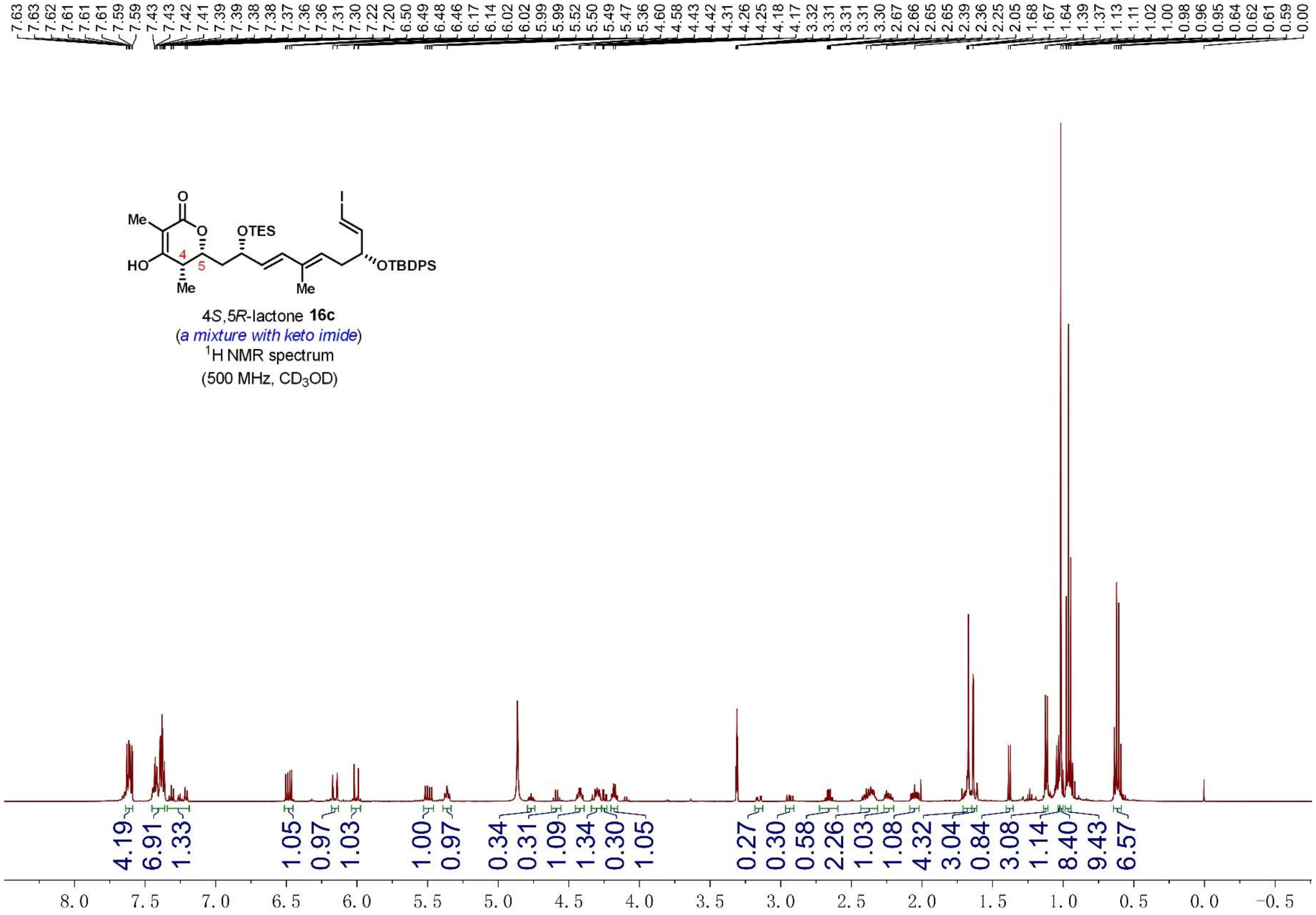
Me
O
O
OTES
HO
4
5
Me
Me
I
OTBDPS
4S,5R-lactone 16c
(a mixture with keto imide)
1H NMR spectrum
(500 MHz, CD3OD)
4.19
6.91
1.33
1.05
0.97
1.03
1.00
0.97
0.34
0.31
1.09
1.34
0.30
1.05
0.27
0.30
0.58
2.26
1.03
1.08
4.32
3.04
0.84
3.08
1.14
8.40
9.43
6.57
8.0
7.5
7.0
6.5
6.0
5.5
5.0
4.5
4.0
3.5
3.0
2.5
2.0
1.5
1.0
0.5
0.0
-0.5

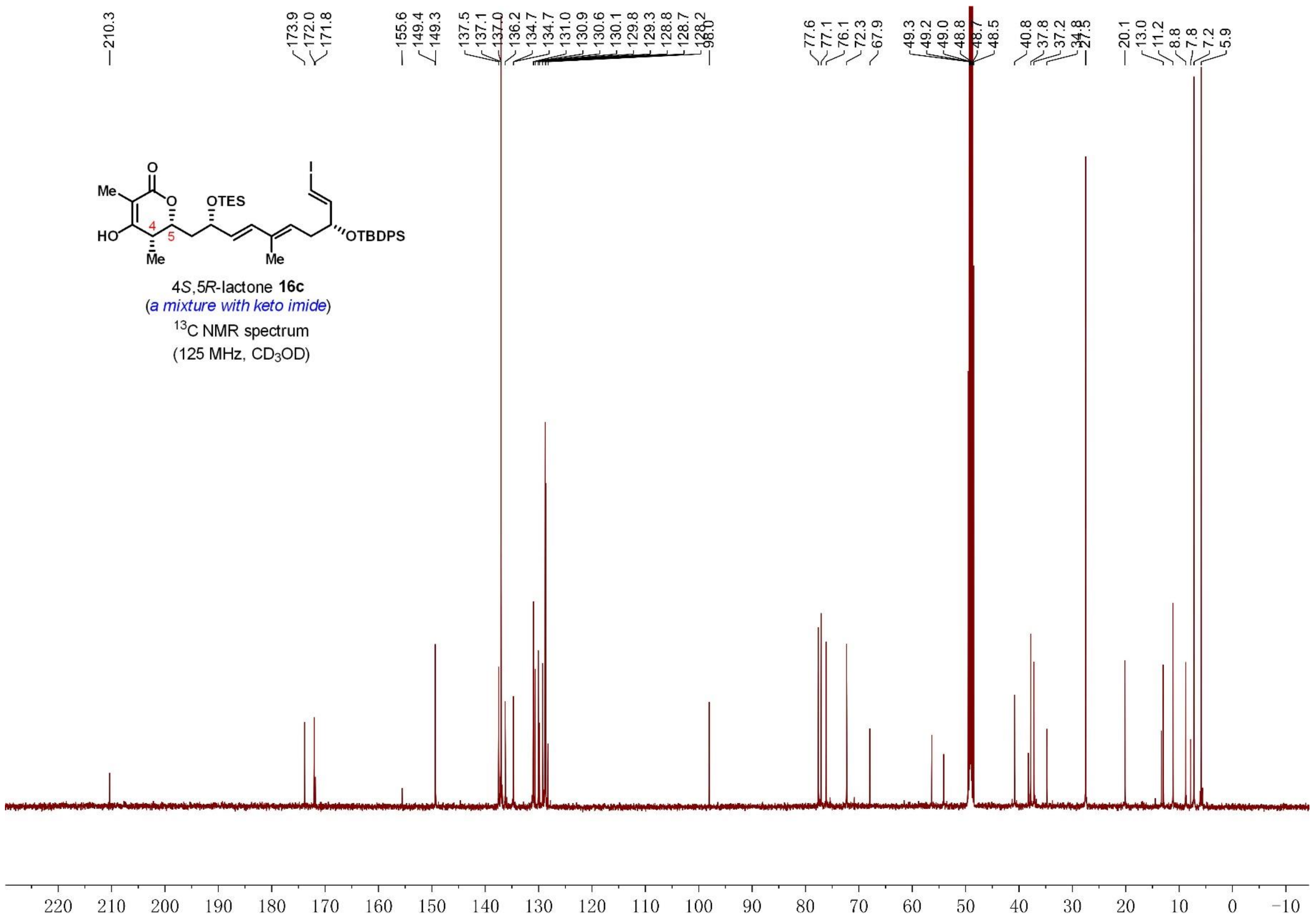
210.3
173.9
172.0
171.8
155.6
149.4
149.3
137.5
137.1
137.0
136.2
134.7
134.7
131.0
130.9
130.6
130.1
129.8
129.3
128.8
128.7
128.2
98.0
77.6
77.1
76.1
72.3
67.9
49.3
49.2
49.0
48.8
48.7
48.5
40.8
37.8
37.2
34.8
27.5
20.1
13.0
11.2
8.8
7.8
7.2
5.9
Me
O
O
OTES
I
HO
4
5
OTBDPS
Me
Me
4S,5R-lactone 16c
(a mixture with keto imide)
13C NMR spectrum
(125 MHz, CD3OD)
220
210
200
190
180
170
160
150
140
130
120
110
100
90
80
70
60
50
40
30
20
10
0
−10

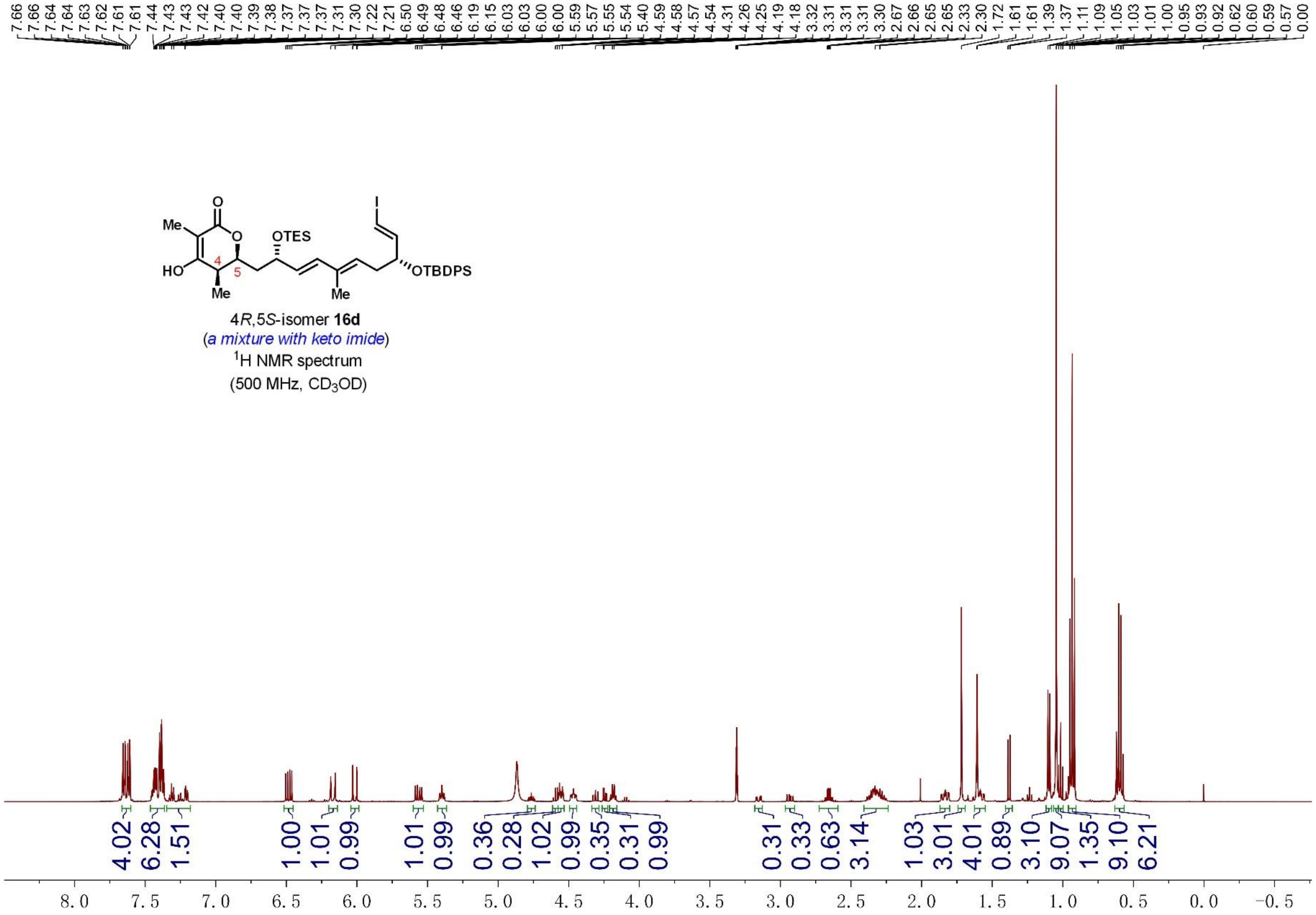
Me
HO
Me
OTES
Me
I
OTBDPS
4R,5S-isomer 16d
(a mixture with keto imide)
1H NMR spectrum
(500 MHz, CD3OD)

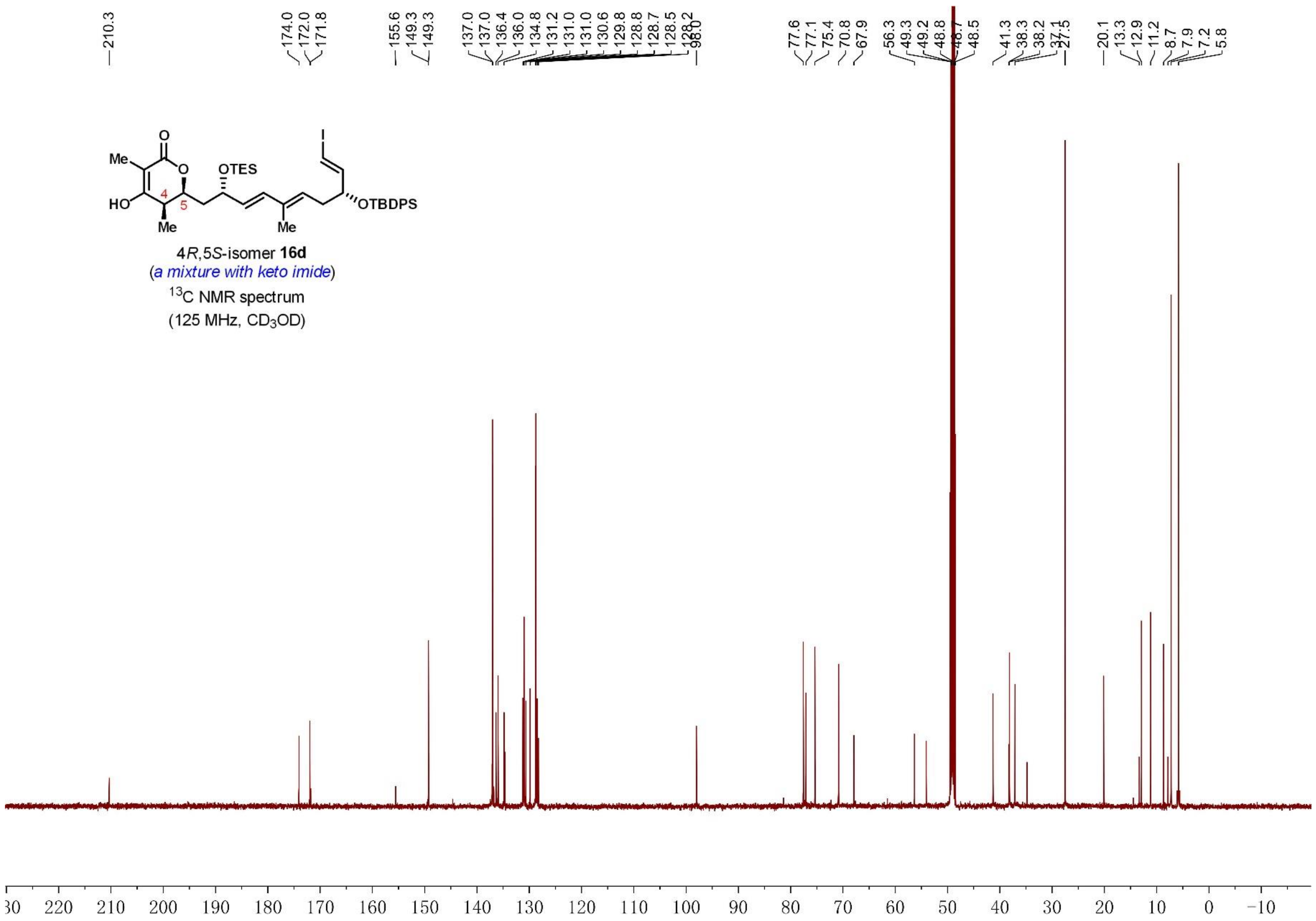

210.3
174.0
172.0
171.8
155.6
149.3
149.3
137.0
137.0
136.4
136.0
134.8
131.2
131.0
131.0
130.6
129.8
128.8
128.7
128.5
128.2
98.0
77.6
77.1
75.4
70.8
67.9
56.3
49.3
49.2
48.8
48.7
48.5
41.3
38.3
38.2
37.1
27.5
20.1
13.3
12.9
11.2
8.7
7.9
7.2
5.8
Me
O
OTES
HO
4
5
Me
Me
I
OTBDPS
4R,5S-isomer 16d
(a mixture with keto imide)
13C NMR spectrum
(125 MHz, CD3OD)
30
220
210
200
190
180
170
160
150
140
130
120
110
100
90
80
70
60
50
40
30
20
10
0
−10

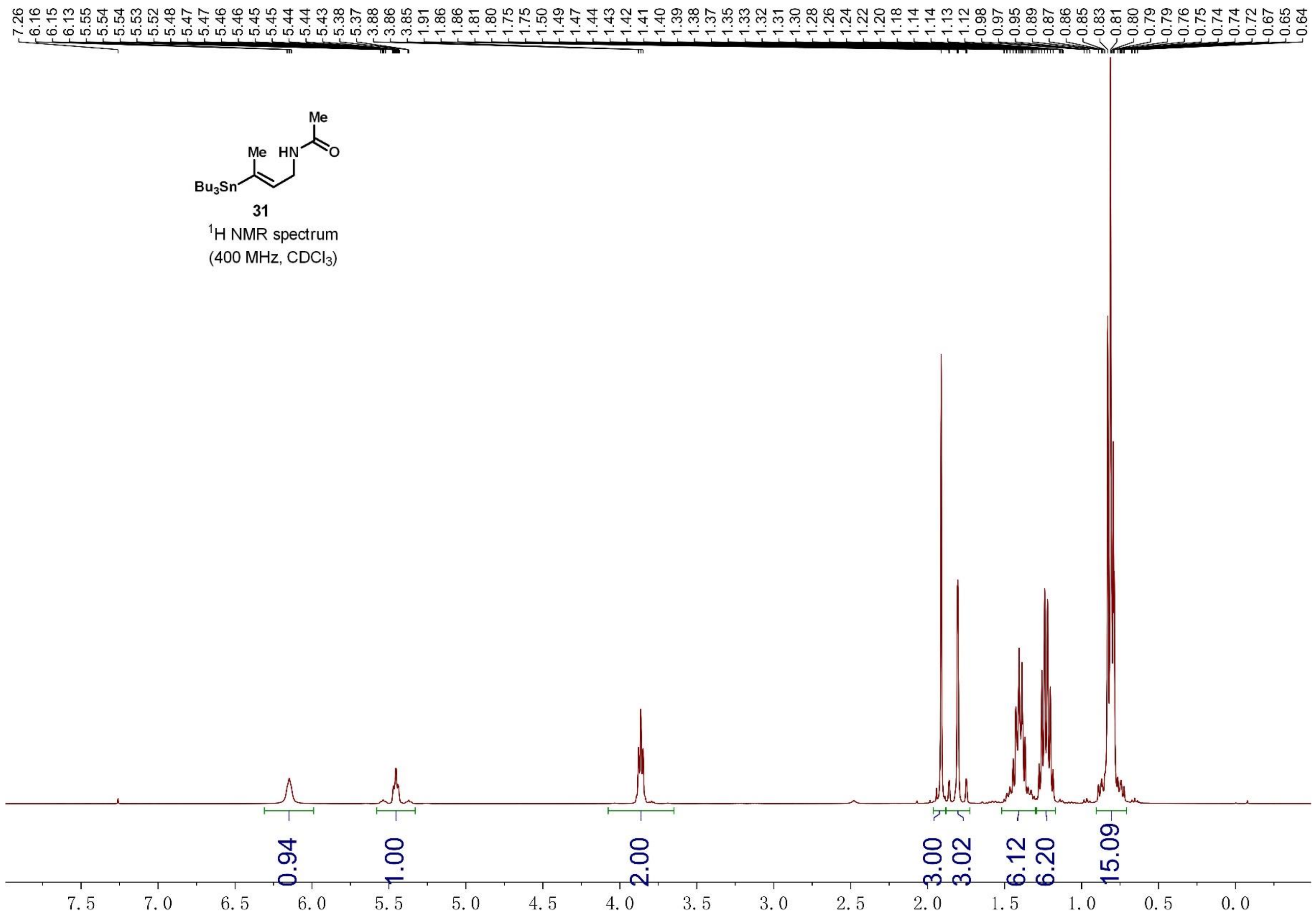
Me
HN
O
Me
Bu3Sn
31
1H NMR spectrum
(400 MHz, CDCl3)
0.94
1.00
2.00
3.00
3.02
6.12
6.20
15.09
7.5
7.0
6.5
6.0
5.5
5.0
4.5
4.0
3.5
3.0
2.5
2.0
1.5
1.0
0.5
0.0

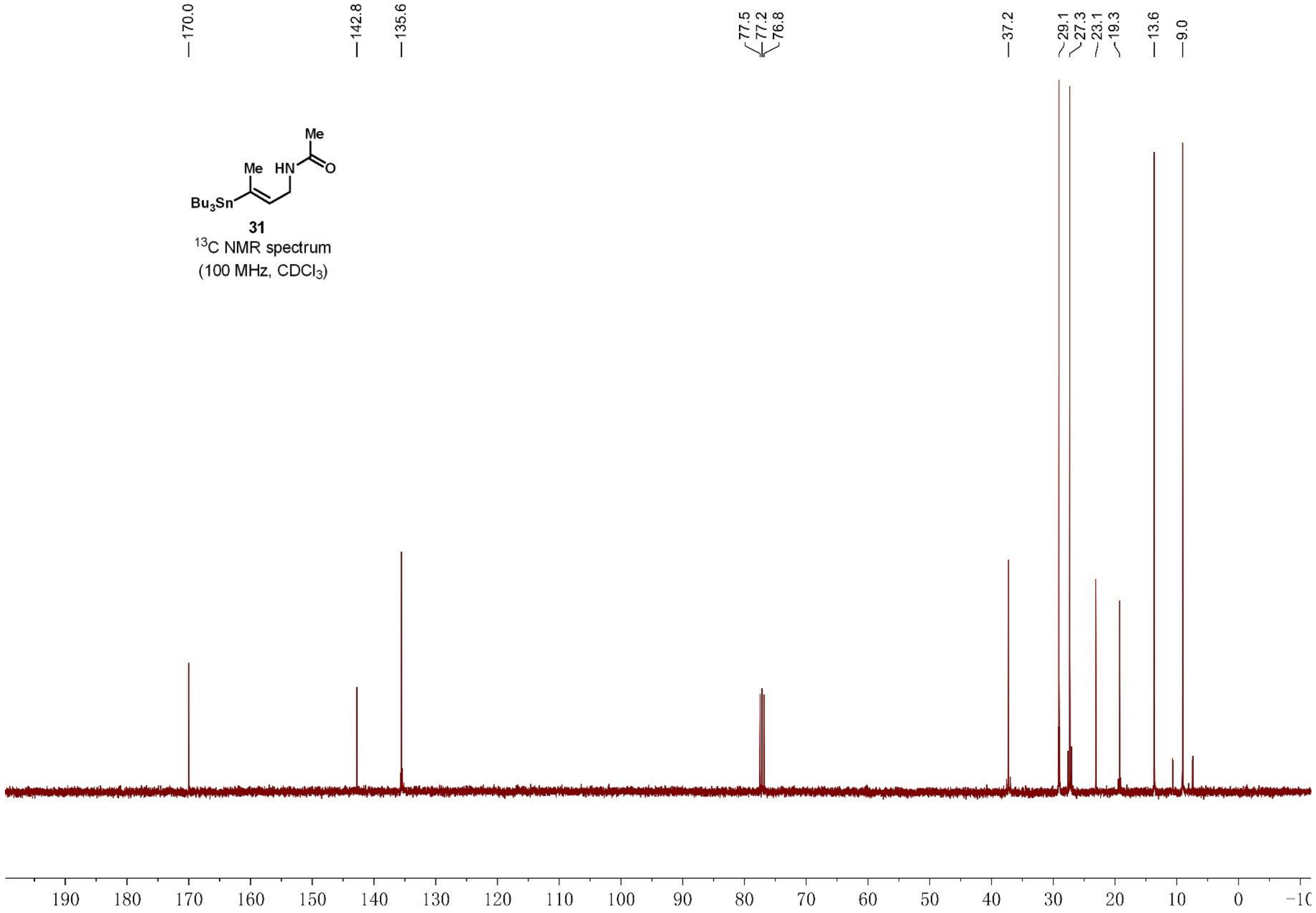

170.0
142.8
135.6
77.5
77.2
76.8
37.2
29.1
27.3
23.1
19.3
13.6
9.0
Me
Me
HN
O
Bu3Sn
31
13C NMR spectrum
(100 MHz, CDCl3)
190
180
170
160
150
140
130
120
110
100
90
80
70
60
50
40
30
20
10
0
−10

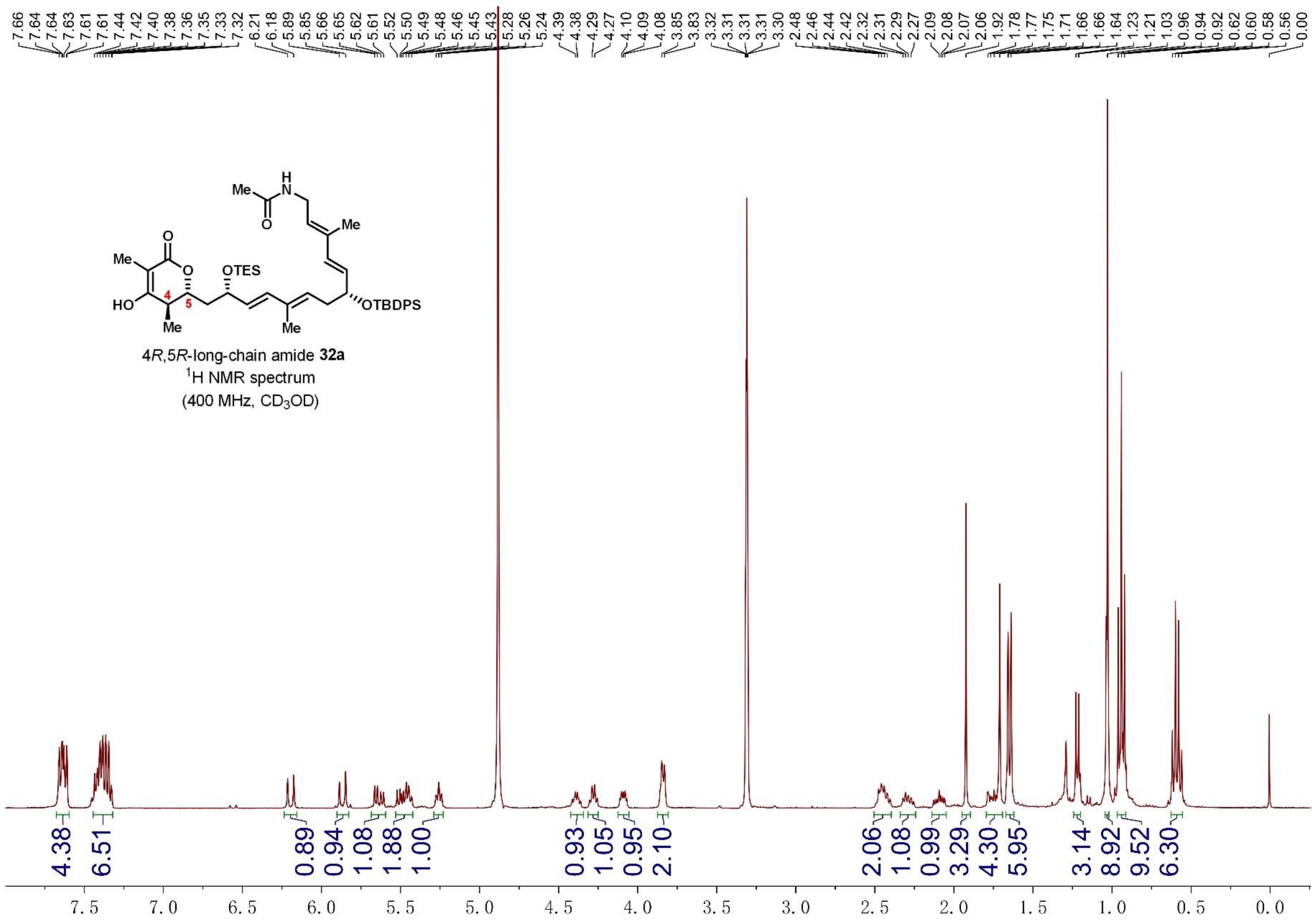

4*R*,5*R*-long-chain amide **32a**
^{1}H NMR spectrum
(400 MHz, CD_3OD)

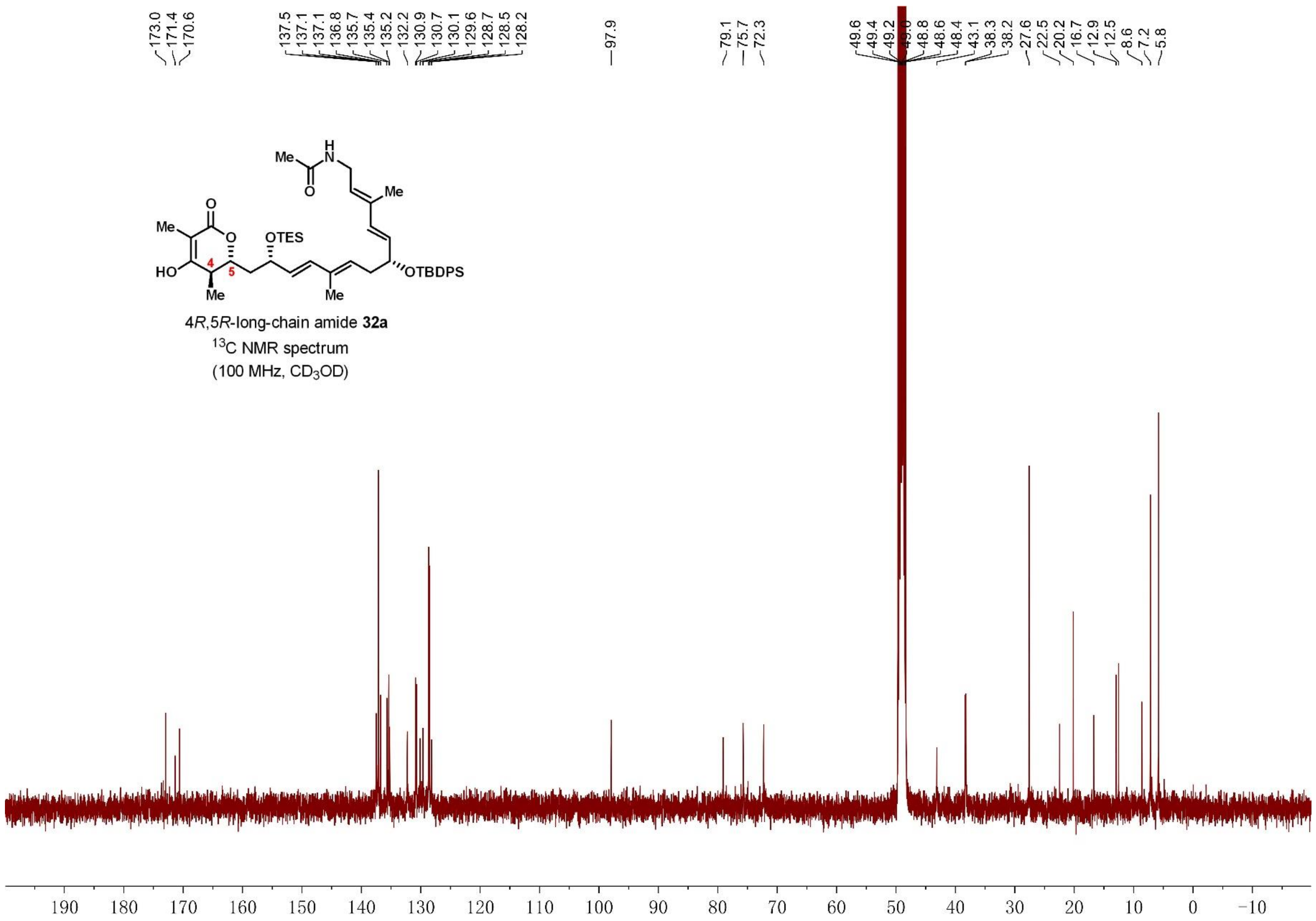

173.0
171.4
170.6
137.5
137.1
137.1
136.8
135.7
135.4
135.2
132.2
130.9
130.7
130.1
129.6
128.7
128.5
128.2
97.9
79.1
75.7
72.3
49.6
49.4
49.2
48.8
48.6
48.4
43.1
38.3
38.2
27.6
22.5
20.2
16.7
12.9
12.5
8.6
7.2
5.8
Me
H
N
O
Me
O
Me
O
OTES
HO
4
5
Me
Me
OTBDPS
4*R*,5*R*-long-chain amide **32a**
^{13}C NMR spectrum
(100 MHz, CD_3OD)
190
180
170
160
150
140
130
120
110
100
90
80
70
60
50
40
30
20
10
0
−10

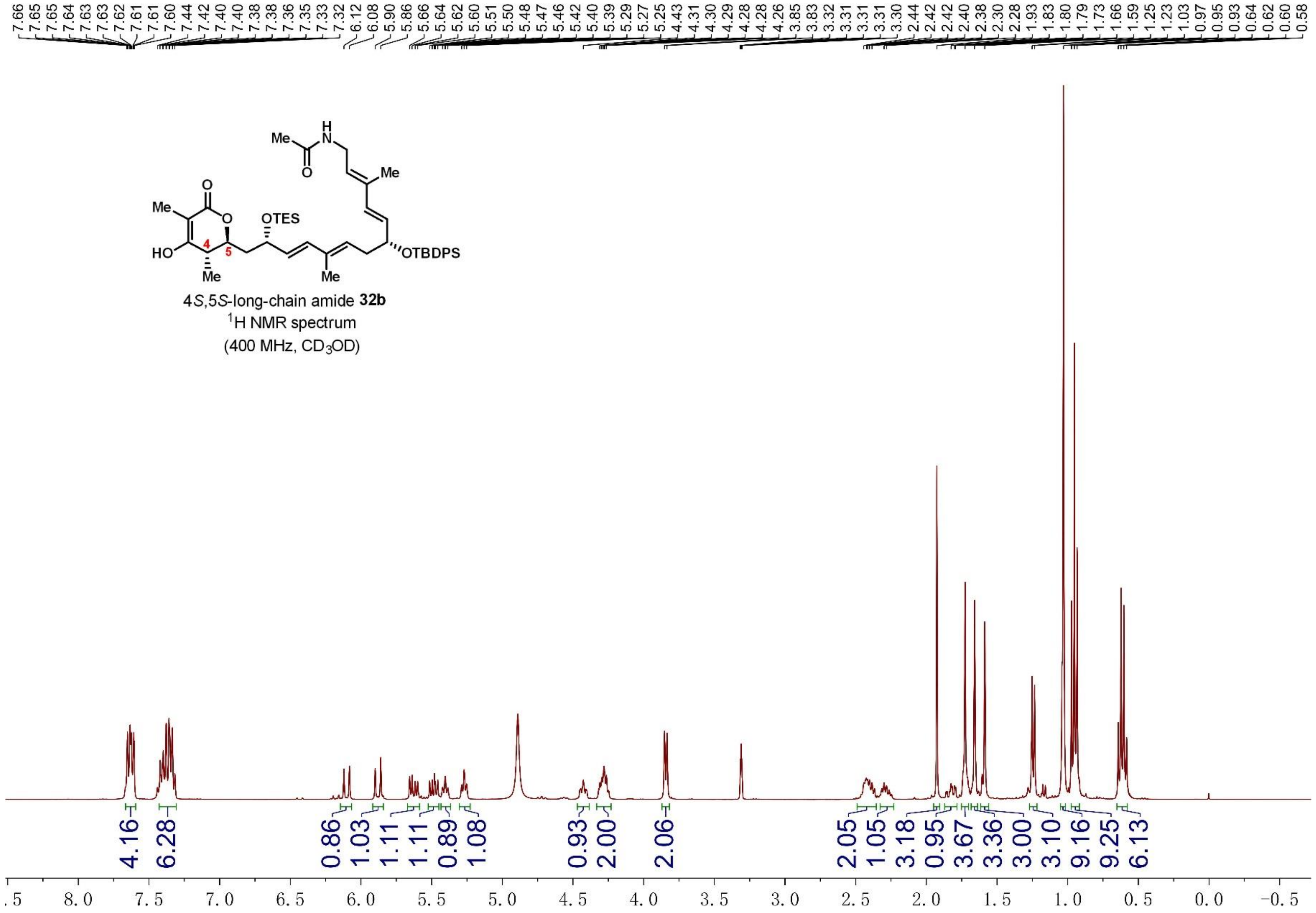
4S,5S-long-chain amide 32b
1H NMR spectrum
(400 MHz, CD3OD)

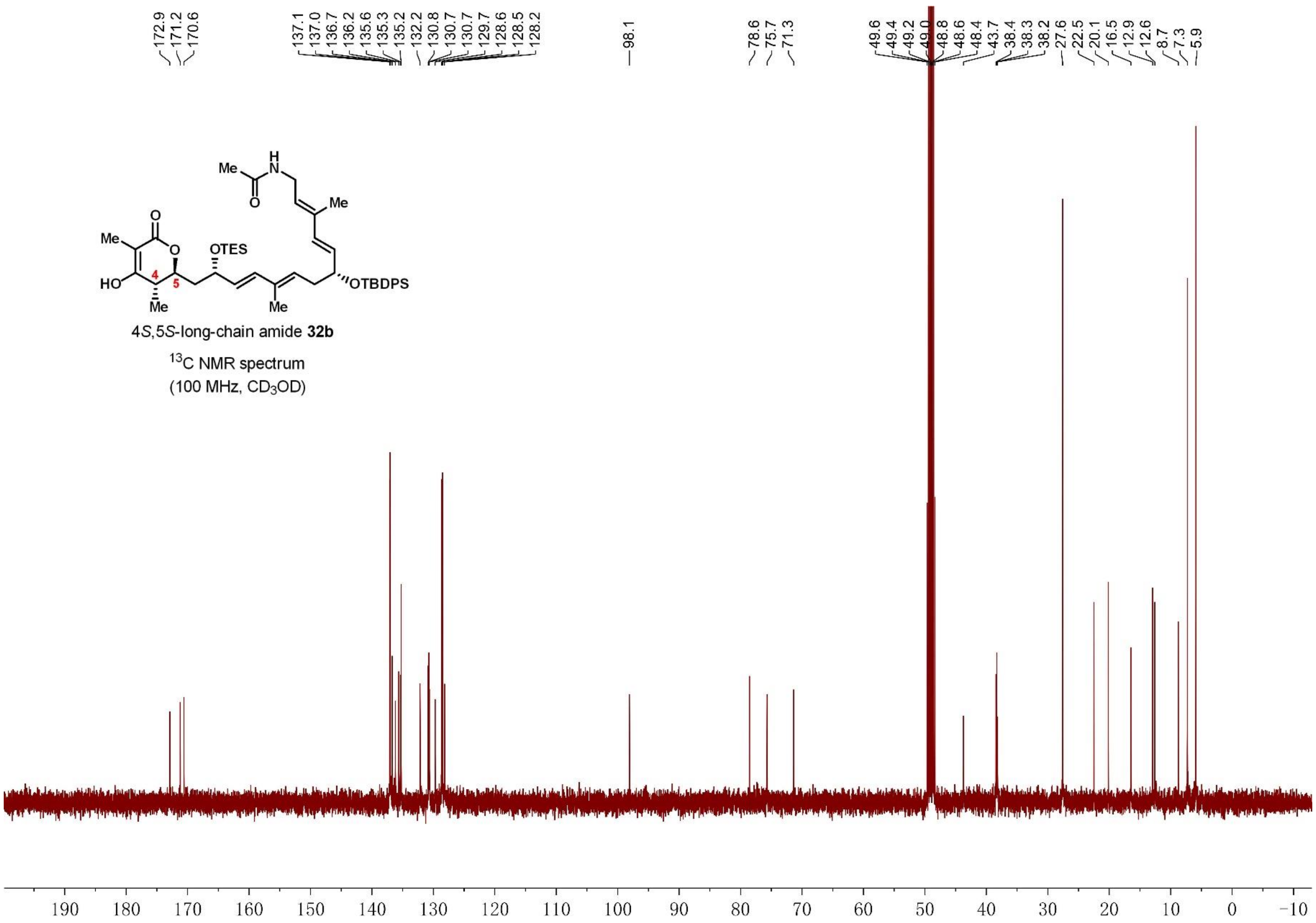

4*S*,5*S*-long-chain amide **32b**

^{13}C NMR spectrum

(100 MHz, CD_3OD)

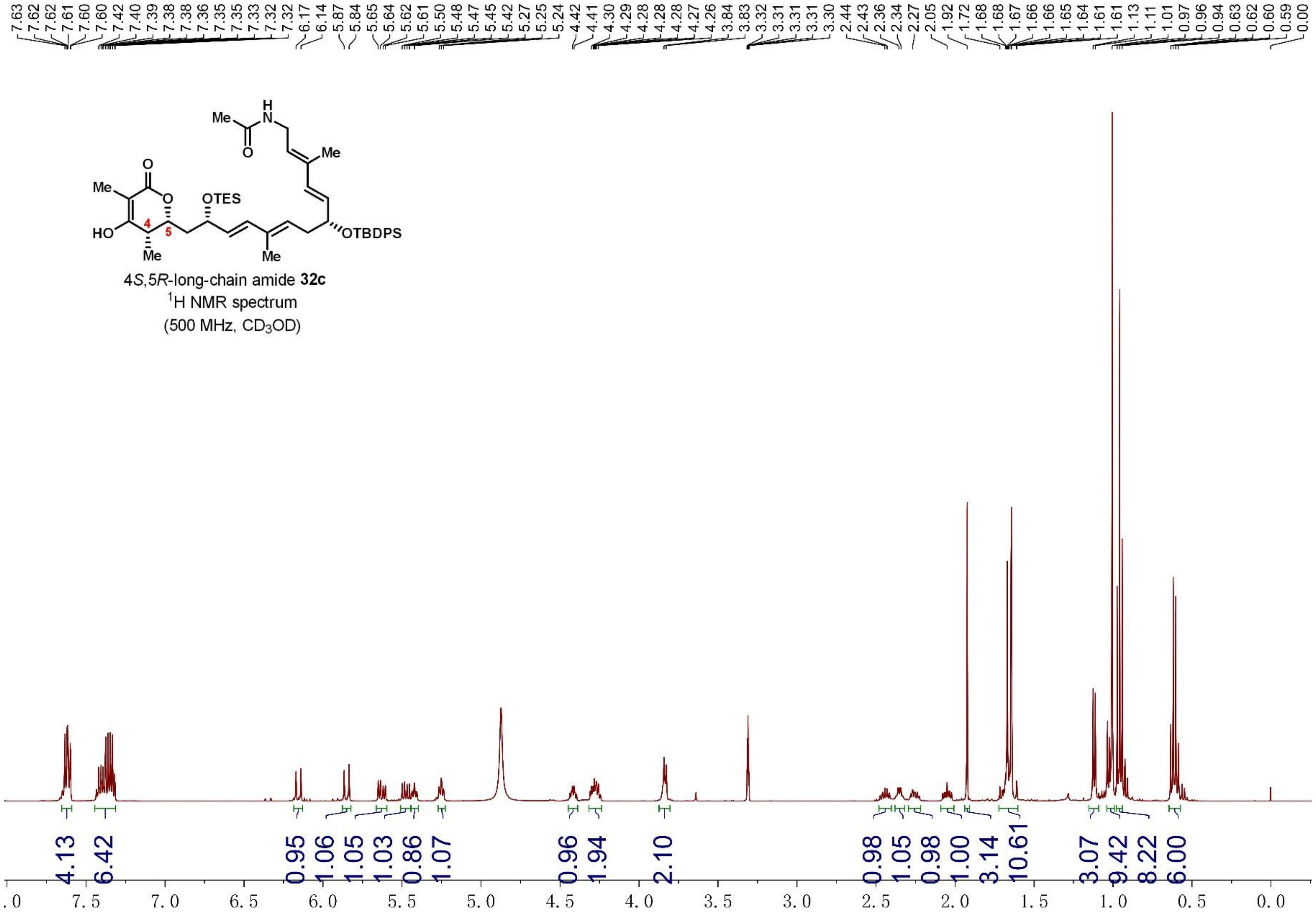

4*S*,5*R*-long-chain amide **32c**
^{1}H NMR spectrum
(500 MHz, CD_3OD)

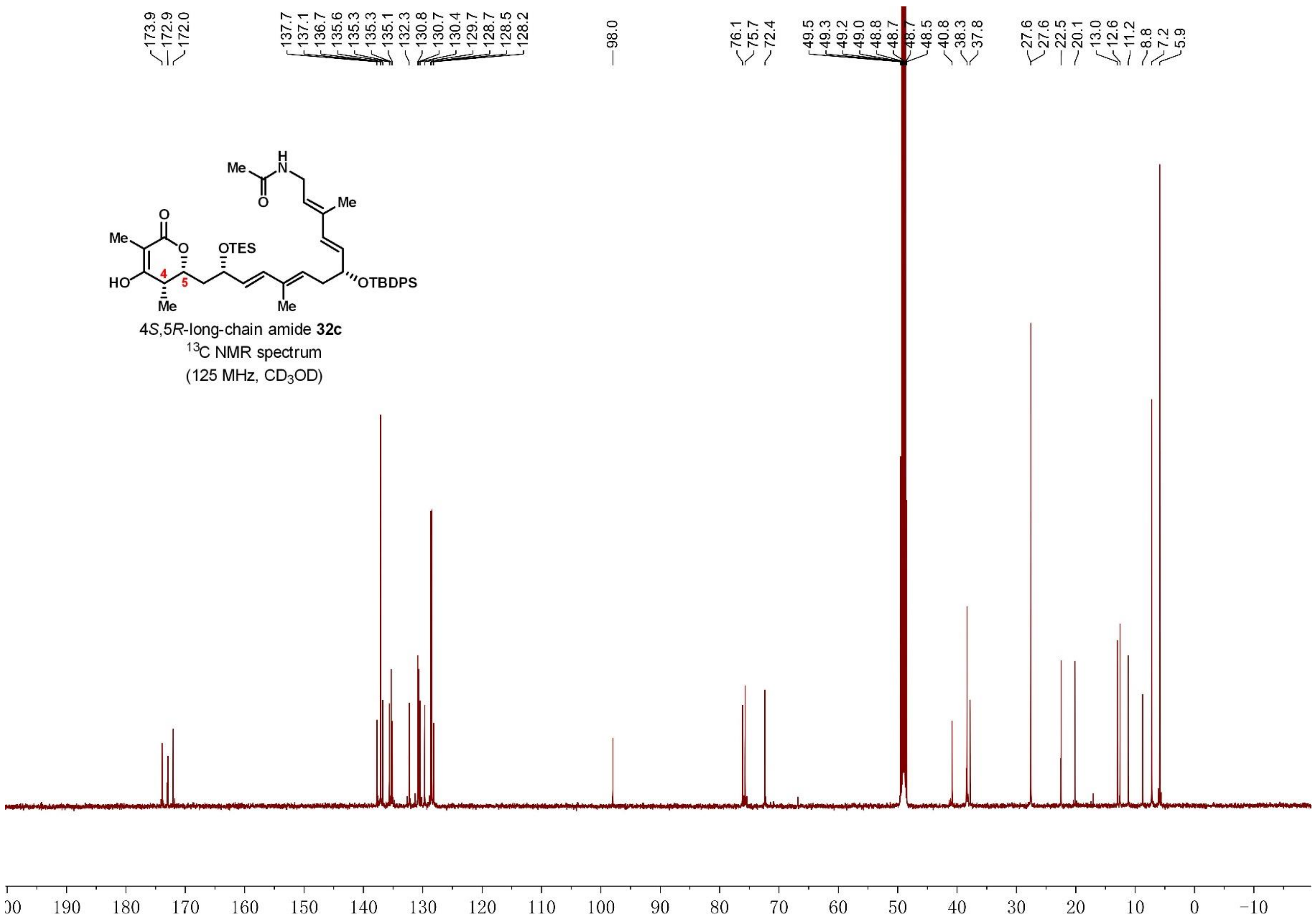

173.9
172.9
172.0
137.7
137.1
136.7
135.6
135.3
135.3
135.1
132.3
130.8
130.7
130.4
129.7
128.7
128.5
128.2
98.0
76.1
75.7
72.4
49.5
49.3
49.2
49.0
48.8
48.7
48.7
48.5
40.8
38.3
37.8
27.6
27.6
22.5
20.1
13.0
12.6
11.2
8.8
7.2
5.9
Me
H
N
O
OTES
OTBDPS
HO
4
5
4S,5R-long-chain amide 32c
13C NMR spectrum
(125 MHz, CD3OD)

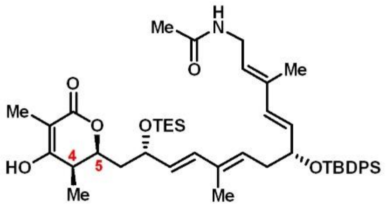

4*R*,5*S*-long-chain amide **32d**
^{1}H NMR spectrum
(500 MHz, CD_3OD)

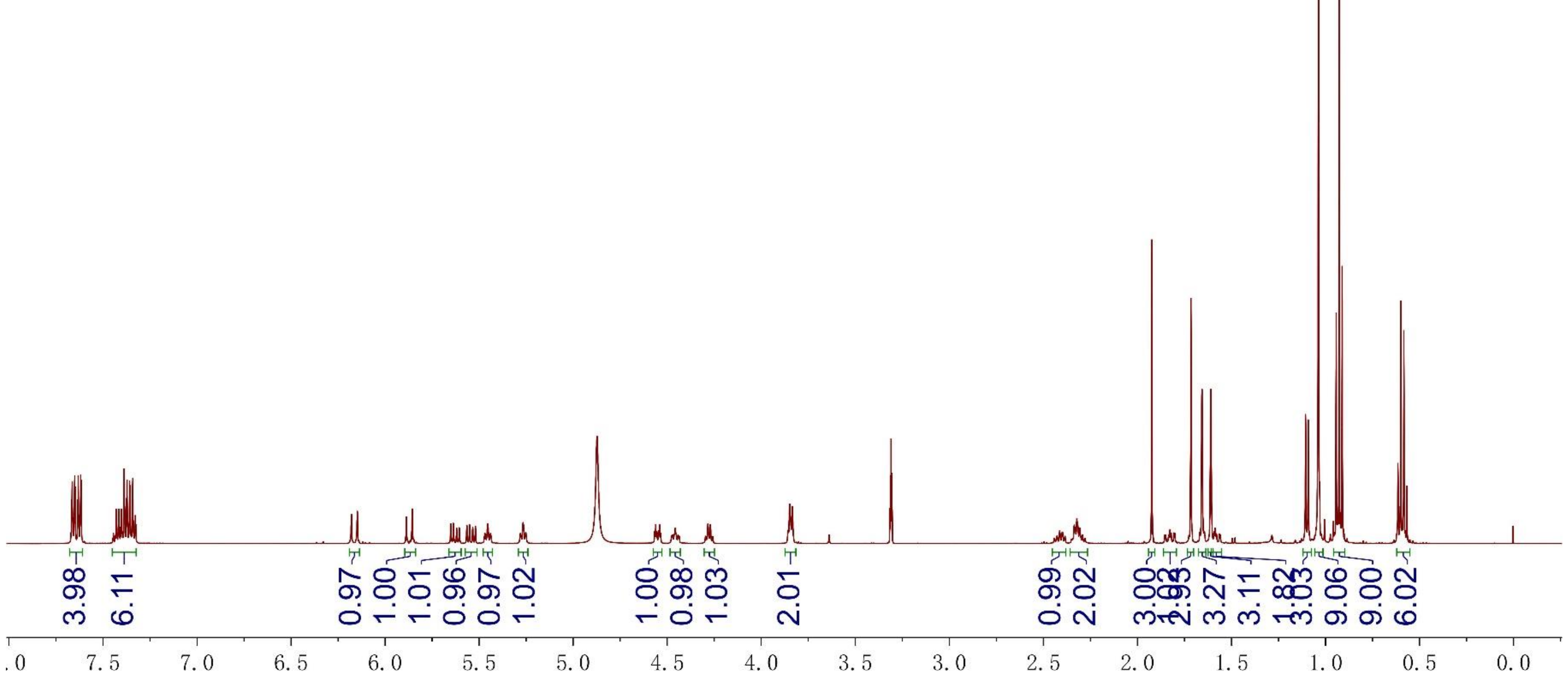

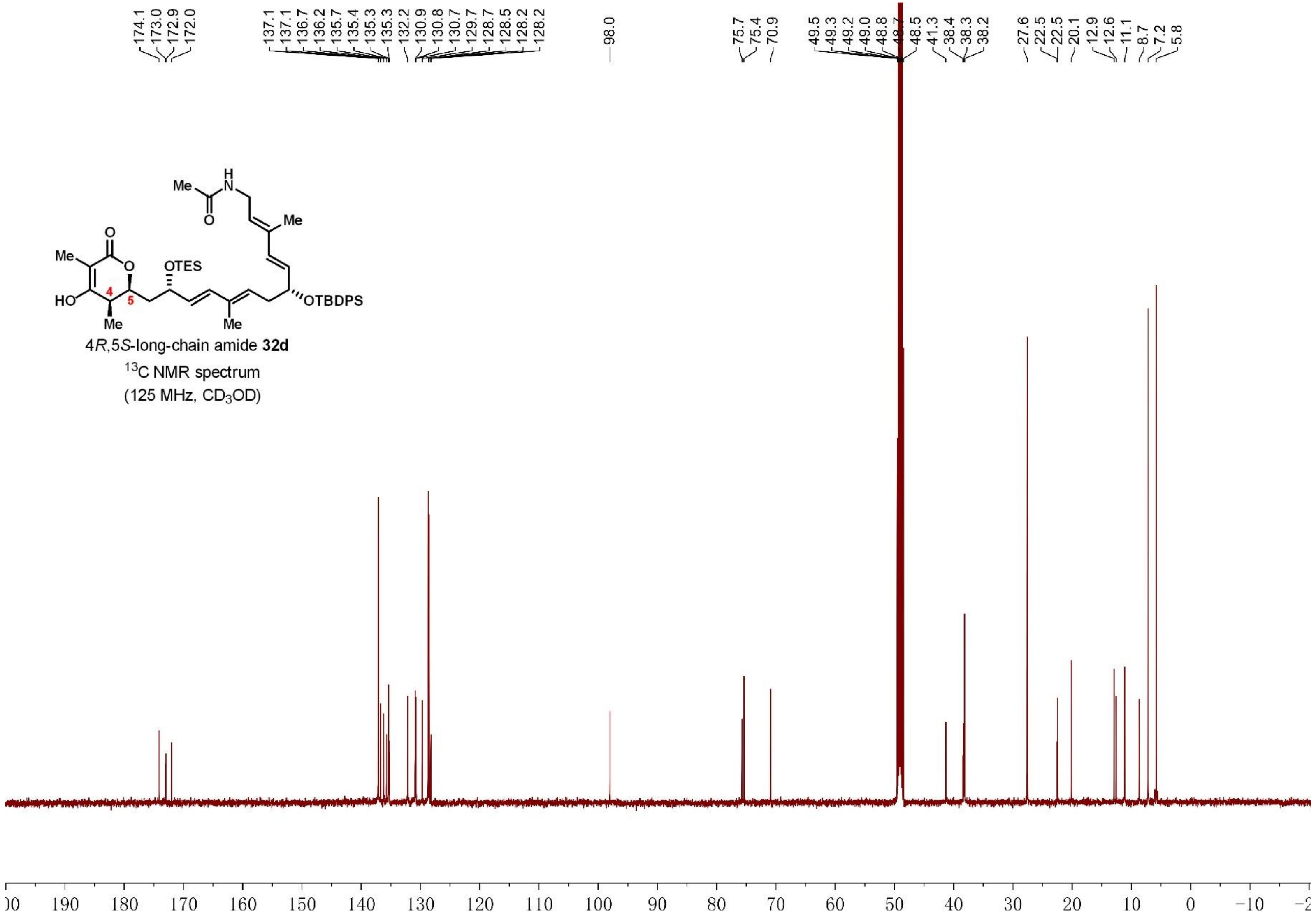
174.1
173.0
172.9
172.0
137.1
137.1
136.7
136.2
135.7
135.4
135.3
135.3
132.2
130.9
130.8
130.7
129.7
128.7
128.5
128.2
128.2
98.0
75.7
75.4
70.9
49.5
49.3
49.2
49.0
48.8
48.7
48.5
41.3
38.4
38.3
38.2
27.6
22.5
22.5
20.1
12.9
12.6
11.1
8.7
7.2
5.8
Me
N
H
O
Me
O
O
Me
OTES
HO
4
5
Me
Me
OTBDPS
4*R*,5*S*-long-chain amide **32d**
^{13}C NMR spectrum
(125 MHz, CD_3OD)
190
180
170
160
150
140
130
120
110
100
90
80
70
60
50
40
30
20
10
0
−10

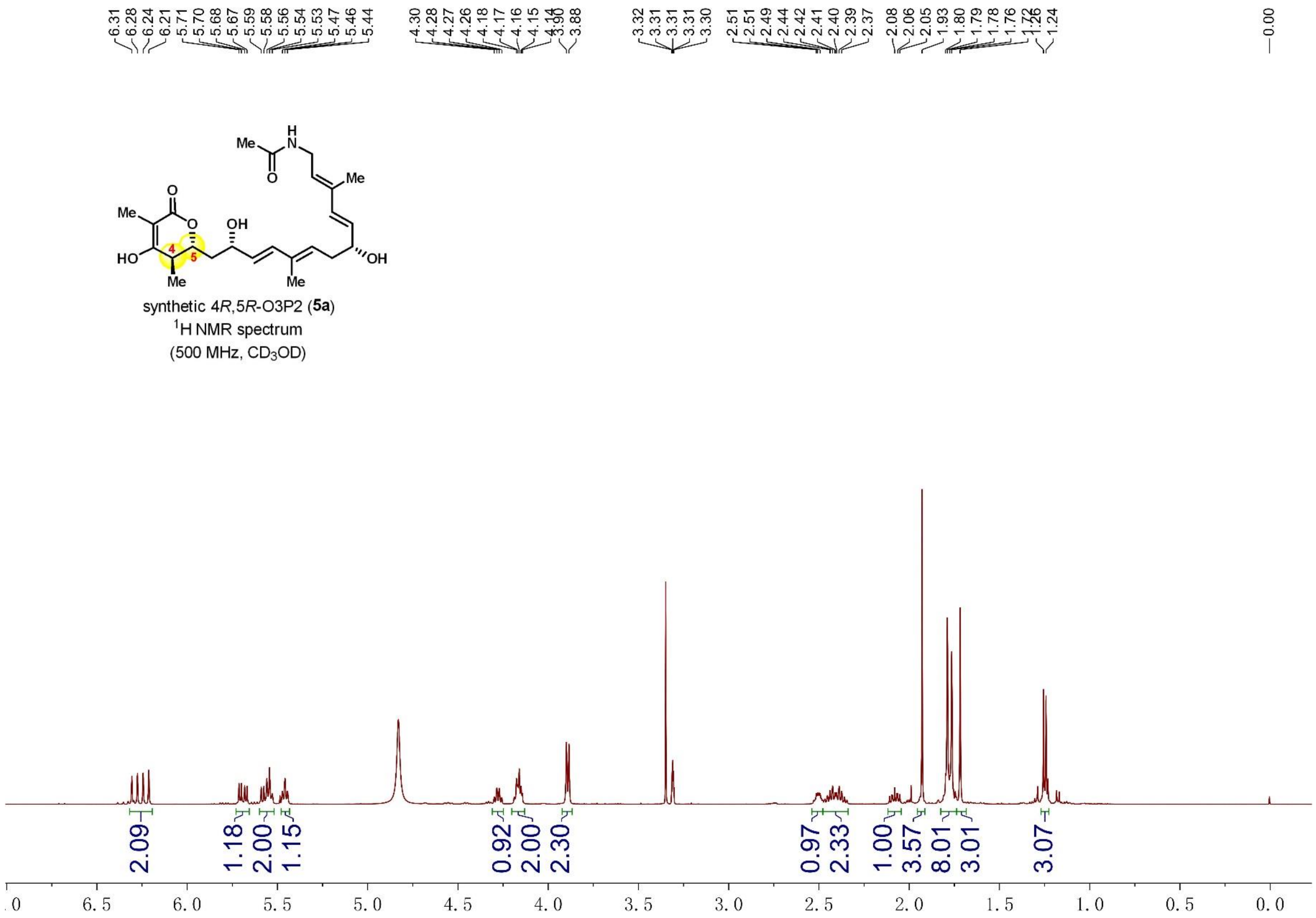

synthetic 4*R*,5*R*-O3P2 (**5a**)
^{1}H NMR spectrum
(500 MHz, CD_3OD)

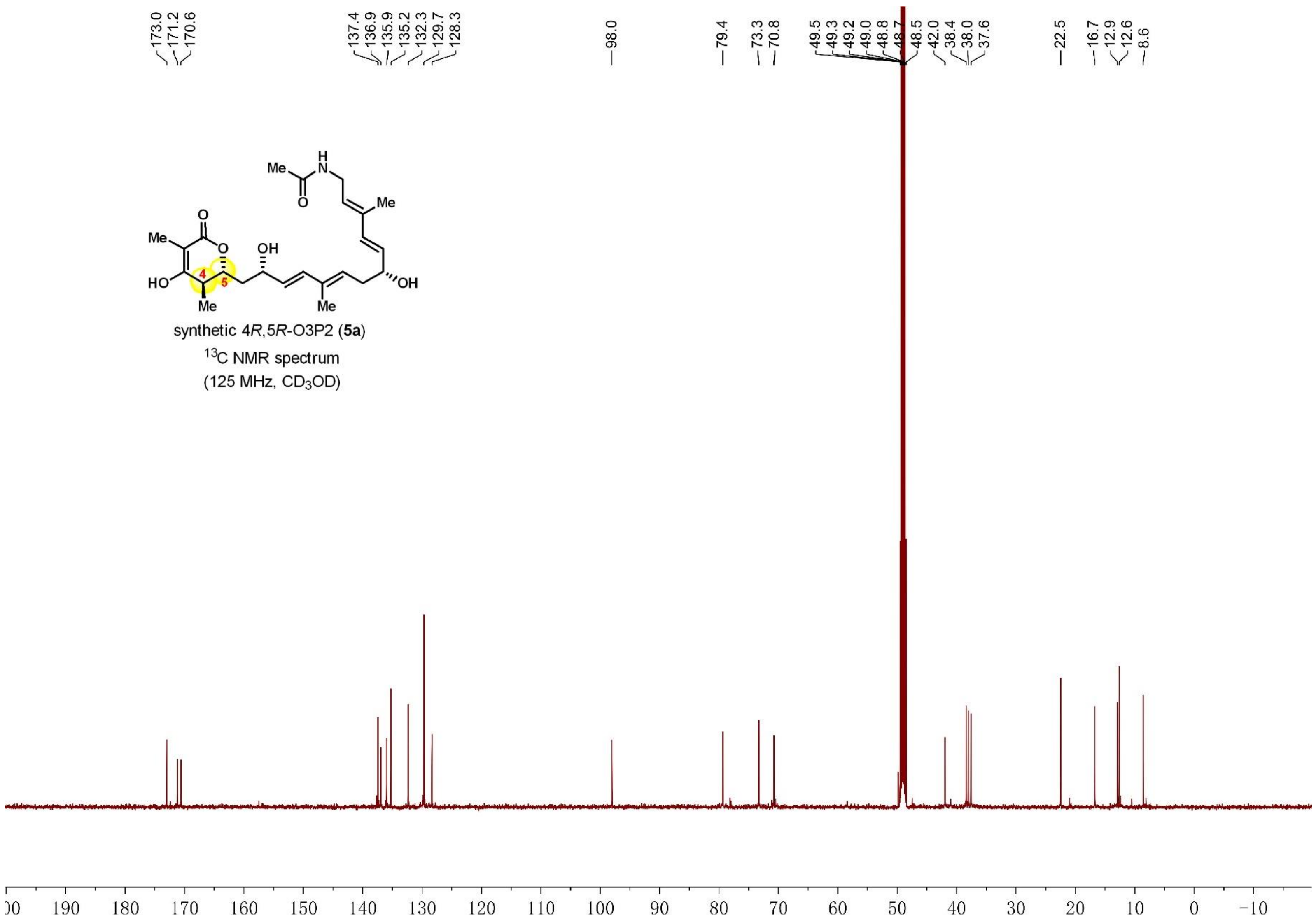
173.0
171.2
170.6
137.4
136.9
135.9
135.2
132.3
129.7
128.3
98.0
79.4
73.3
70.8
49.5
49.3
49.2
49.0
48.8
48.7
48.5
42.0
38.4
38.0
37.6
22.5
16.7
12.9
12.6
8.6
Me
H
N
O
Me
O
O
Me
OH
HO
4
5
Me
Me
OH
synthetic 4R,5R-O3P2 (5a)
13C NMR spectrum
(125 MHz, CD3OD)
190
180
170
160
150
140
130
120
110
100
90
80
70
60
50
40
30
20
10
0
−10

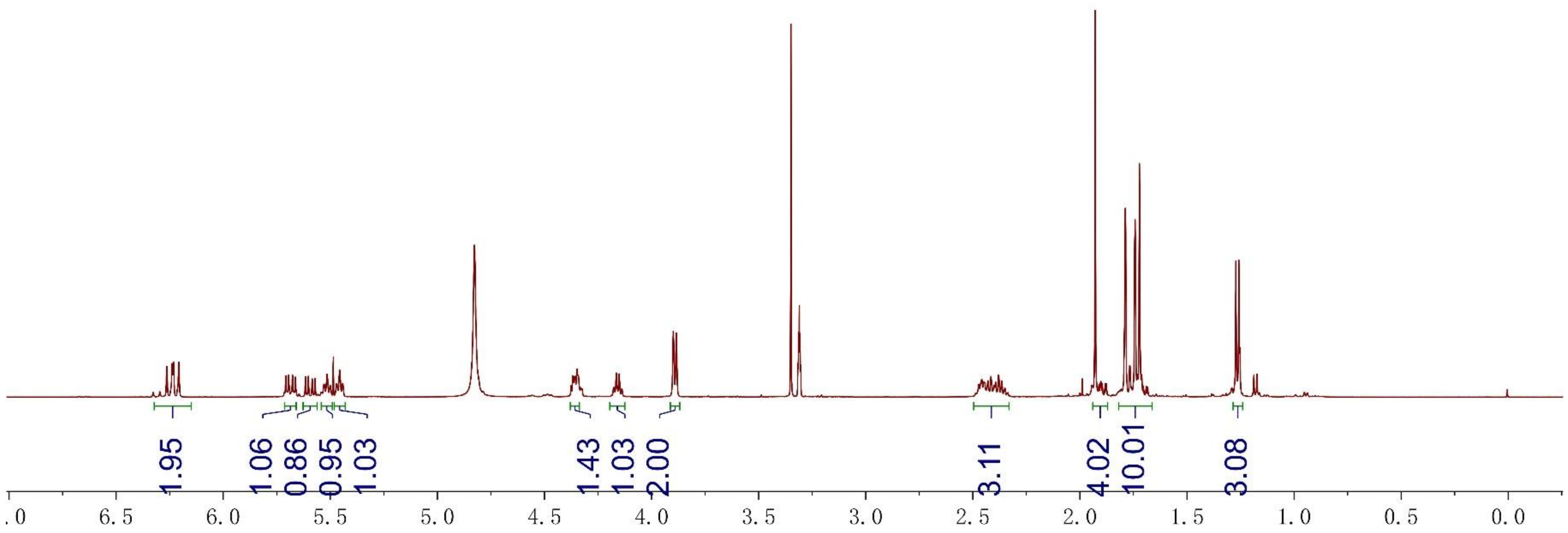

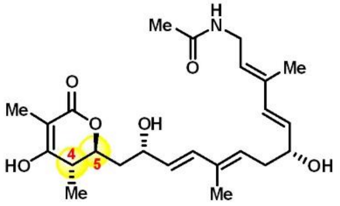

synthetic 4*S*,5*S*-O3P2 (**5b**)
^{1}H NMR spectrum
(500 MHz, CD_3OD)

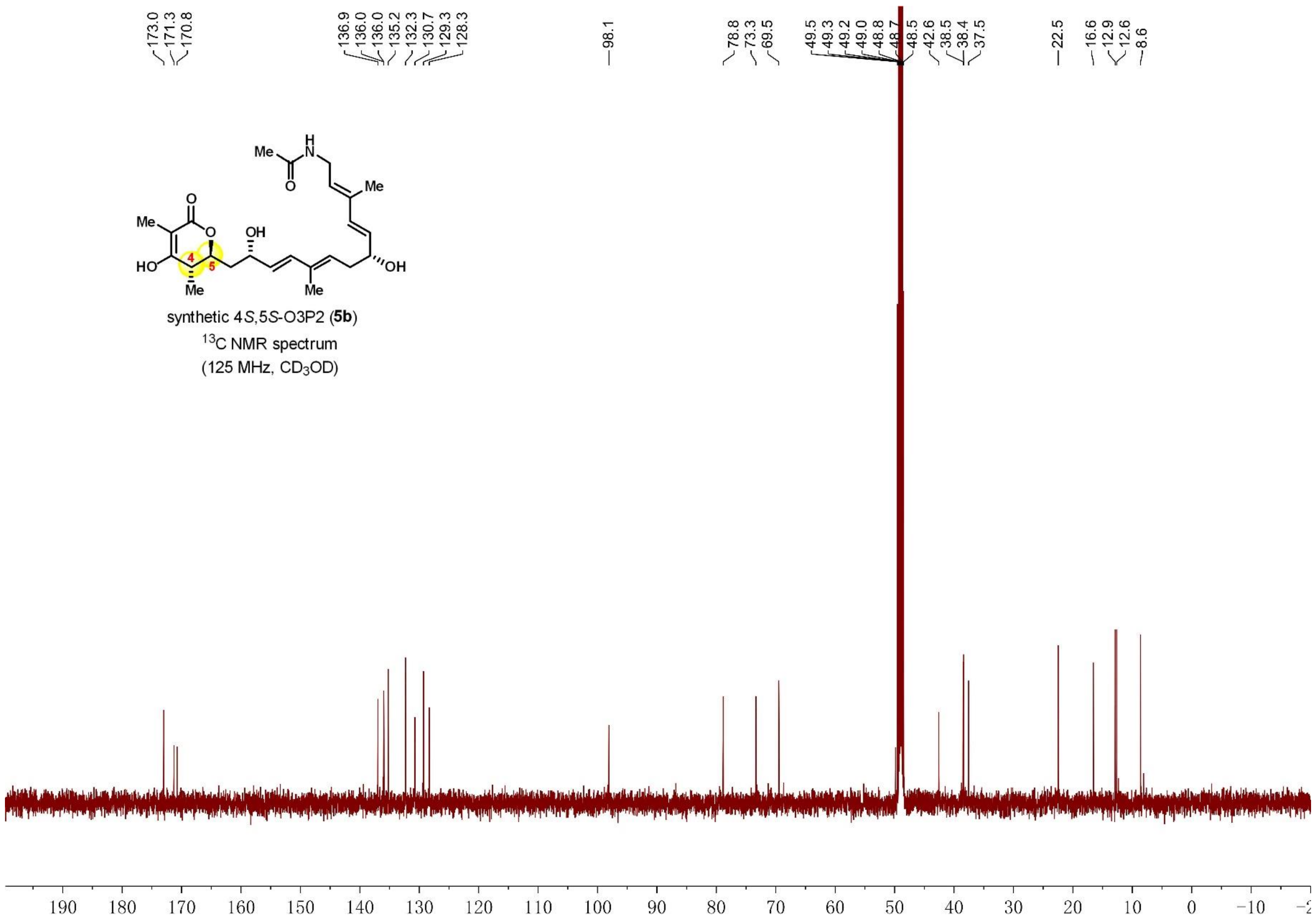
173.0
171.3
170.8
136.9
136.0
136.0
135.2
132.3
130.7
129.3
128.3
98.1
78.8
73.3
69.5
49.5
49.3
49.2
49.0
48.8
48.7
48.5
42.6
38.5
38.4
37.5
22.5
16.6
12.9
12.6
8.6
synthetic 4S,5S-O3P2 (5b)
13C NMR spectrum
(125 MHz, CD3OD)
190 180 170 160 150 140 130 120 110 100 90 80 70 60 50 40 30 20 10 0 −10

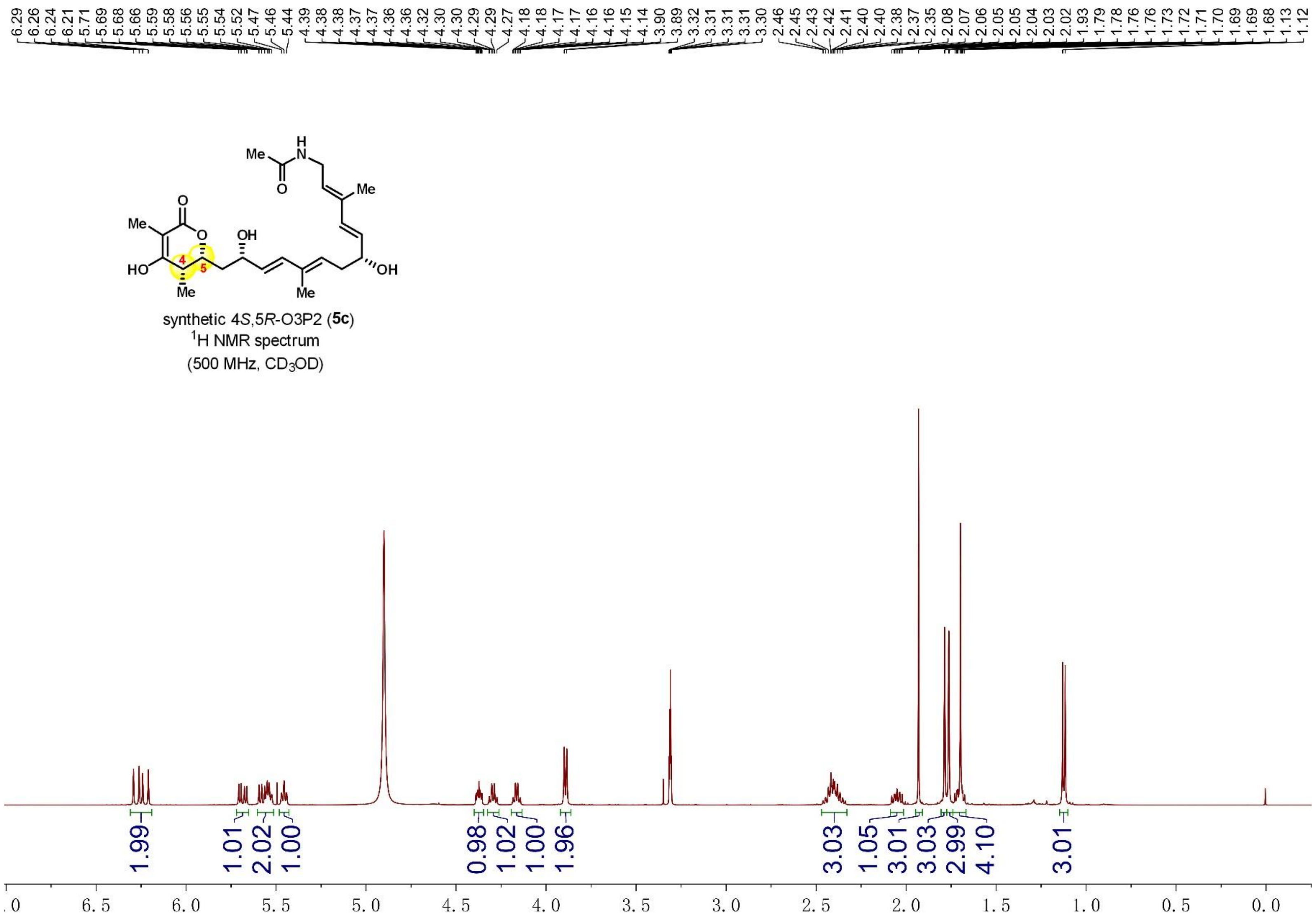
synthetic 4S,5R-O3P2 (5c)
1H NMR spectrum
(500 MHz, CD3OD)
1.99
1.01
2.02
1.00
0.98
1.02
1.00
1.96
3.03
1.05
3.01
3.03
2.99
4.10
3.01
6.5
6.0
5.5
5.0
4.5
4.0
3.5
3.0
2.5
2.0
1.5
1.0
0.5
0.0

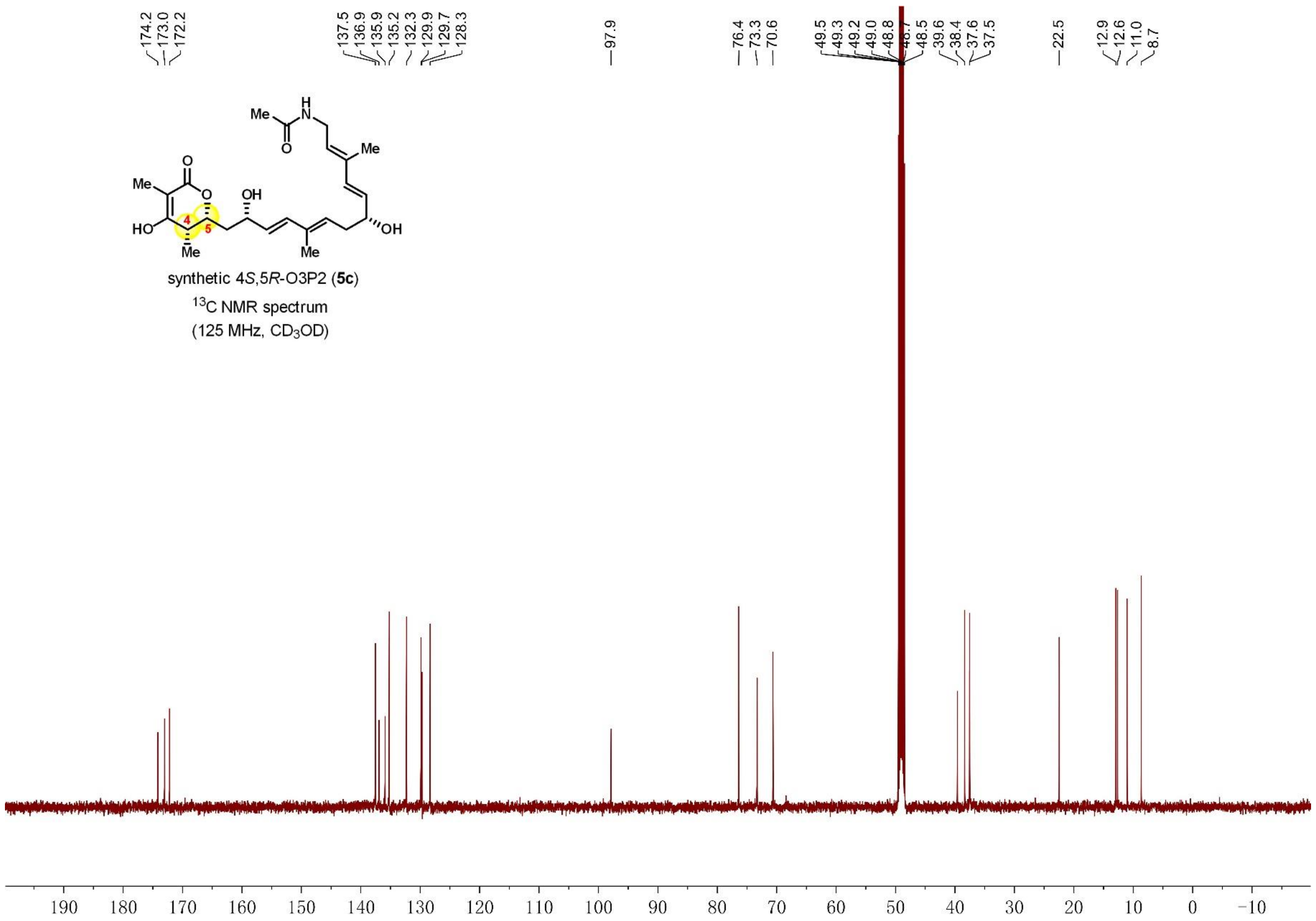
174.2
173.0
172.2
137.5
136.9
135.9
135.2
132.3
129.9
129.7
128.3
97.9
76.4
73.3
70.6
49.5
49.3
49.2
49.0
48.8
48.7
48.5
39.6
38.4
37.6
37.5
22.5
12.9
12.6
11.0
8.7
synthetic 4S,5R-O3P2 (5c)
13C NMR spectrum
(125 MHz, CD3OD)
190 180 170 160 150 140 130 120 110 100 90 80 70 60 50 40 30 20 10 0 −10

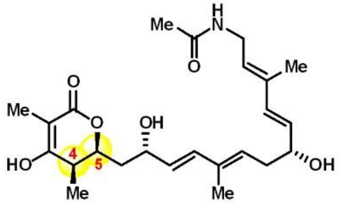

synthetic 4*R*,5*S*-O3P2 (**5d**)
^{1}H NMR spectrum
(500 MHz, CD_3OD)

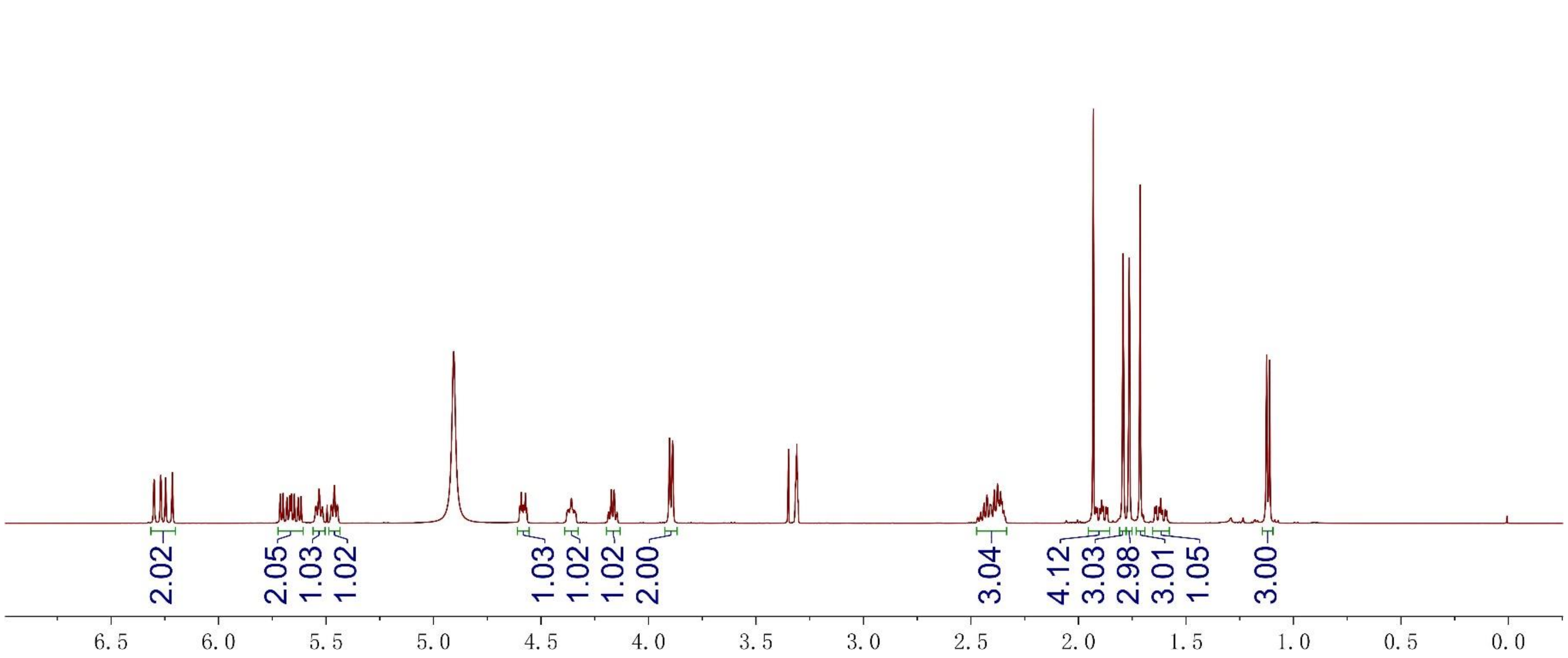

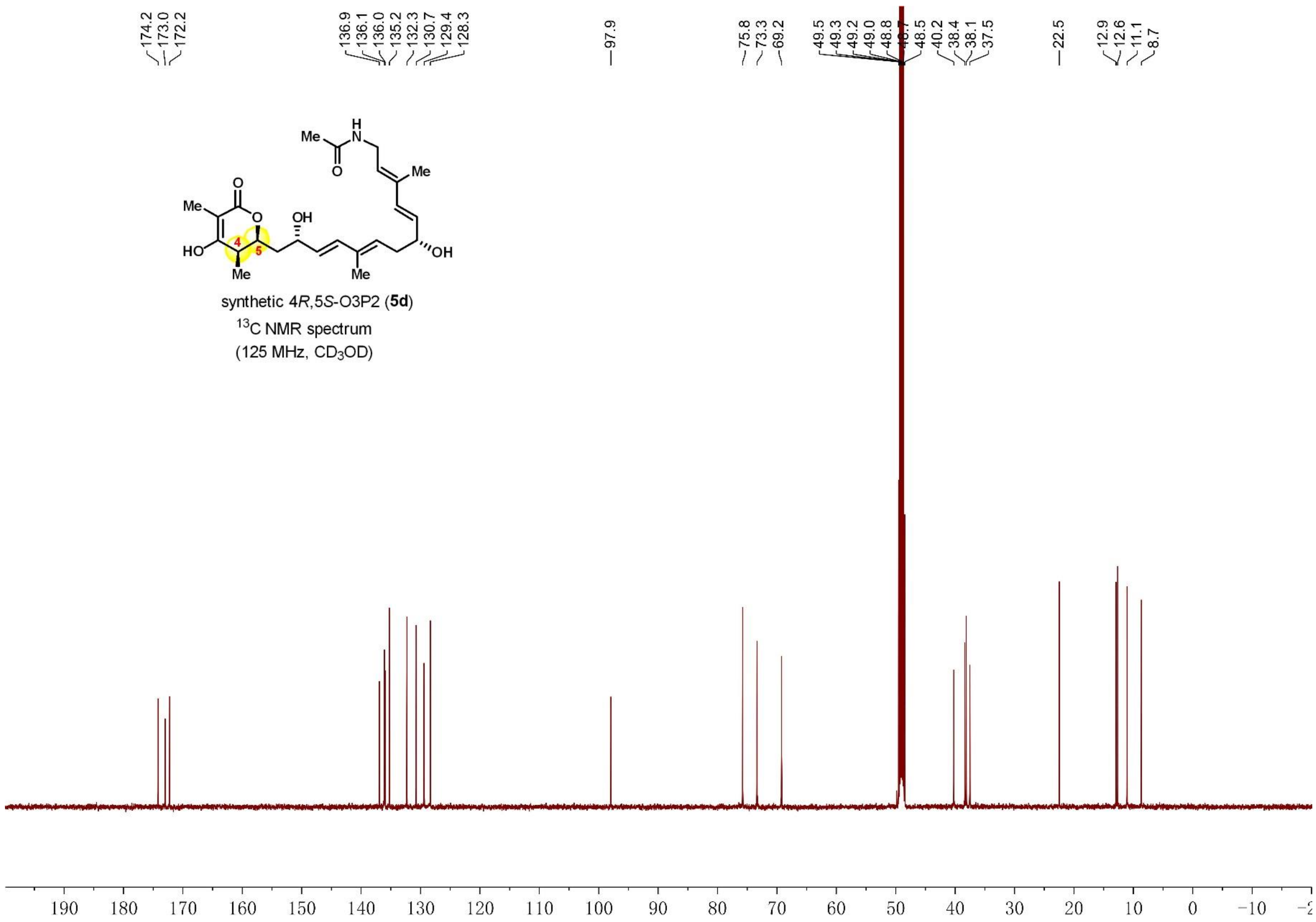
174.2
173.0
172.2
136.9
136.1
136.0
135.2
132.3
130.7
129.4
128.3
97.9
75.8
73.3
69.2
49.5
49.3
49.2
49.0
48.8
48.7
48.5
40.2
38.4
38.1
37.5
22.5
12.9
12.6
11.1
8.7
Me
H
N
O
OH
HO
4
5
synthetic 4R,5S-O3P2 (5d)
13C NMR spectrum
(125 MHz, CD3OD)
190
180
170
160
150
140
130
120
110
100
90
80
70
60
50
40
30
20
10
0
−10
